# Supplementary material for: Ambiphilic cross-coupling with aryl-bismuth reagents
Source: Nature. 2026 Apr 9;654(8117):92–9. doi: 10.1038/s41586-026-10486-8 (PMC13233618; doi:10.1038/s41586-026-10486-8)

nature portfolio

https://doi.org/10.1038/s41586-026-10486-8

Supplementary information

Ambiphilic cross-coupling with aryl-bismuth reagents

In the format provided by the
authors and unedited

Supplementary Information

Ambiphilic cross-coupling with aryl-bismuth reagents

Byeongdo Roh, Benedict A. Williams and Josep Cornella*

Max-Planck-Institut für Kohlenforschung, Kaiser-Wilhelm-Platz 1, Mülheim an der Ruhr, 45470, Germany

*Corresponding author: Josep Cornella, cornella@kofo.mpg.de.

Table of contents

1. General Experimental Details 4

2. Procedures for the Preparation of Starting Materials 5

Table S1. Coupling partners: aryl iodides and aryl boron derivatives 5

Table S2. Aryl-bismuth compounds and aryl thianthrenium salts 13

3. Optimization of Reaction Conditions. 26

Table S3. Ambiphilic cross-coupling of aryl-bismuth with aryl iodide 26

Figure S1. Evaluation of other electrophilic partners 27

Figure S2. Evaluation of the counteranion in ambiphiles acting as nucleophiles 27

Figure S3. Control experiments on the origin of aryl iodide-derived homocoupling products 28

Figure S4. Additive effect 29

Figure S5. Observation of aryl bismuth-derived coupling products 29

Figure S6. Systematic analysis of side product profiles across representative substrates 33

Table S4. Ambiphilic cross-coupling of aryl-bismuth with aryl-boron derivative 34

Figure S7. Evaluation of the counteranion in ambiphiles via anion exchange 35

Figure S8. Evaluation of the counteranion in ambiphiles acting as electrophiles 35

Figure S9. Preliminary ambiphilic reactivity of other aryl-bismuth compounds 36

Table S5. Current limitations in the substrate scope of ambiphilic cross-coupling 37

4. General Procedures for Ambiphilic Cross-Coupling 39

4.1. Ambiphilic cross-coupling of aryl-bismuths with aryl iodides (General Procedure F) 39

4.2. Ambiphilic cross-coupling of aryl-bismuths with aryl-boron derivatives (General Procedure G) 39

5. Synthesis and Characterization of the Products 40

5.1. Ambiphilic cross-coupling of aryl-bismuths with aryl iodides 40

5.2. Ambiphilic cross-coupling of aryl-bismuths with aryl boron derivatives 46

5.3. Ambiphilic cross-coupling with structurally diverse aryl-bismuth reagents 52

5.4. Practical synthetic usage of ambiphilic coupling: case studies 63

Figure S10. Price comparison of aryl boron derivatives and electron-rich 2-haloheteroarenes from commercial vendors 63

6. Mechanistic Investigations ... 66

Figure S11. Control experiment using **OAC-1** as the precatalyst ... 66

Figure S12. Control experiment employing **OAC-1** as a stoichiometric electrophile ... 67

Figure S13. ^{19}F NMR spectra of reaction mixtures for entries 2 and 7 ... 67

Figure S14. Structures of products observed by GC/MS and reported phosphonium salts for comparison ... 68

Figure S15. Plausible mechanism in stoichiometric experiment of **OAC-1** with aryl-bismuth ... 68

Figure S16. Stoichiometric studies on the oxidative addition of ambiphilic aryl-bismuth ... 69

Figure S17. Oxidative addition complexes from the reaction of **ArBi(Br)-1** with **Pd(PPh$_3$)$_4$** ... 69

Figure S18. Independent synthesis of oxidative addition complex **OAC-2** and comparison of its ^{1}H NMR spectrum with that observed in the reaction of **ArBi(Br)-1** with **Pd(PPh$_3$)$_4$** ... 70

Figure S19. COSY spectrum of the reaction of **ArBi(Br)-1** with **Pd(PPh$_3$)$_4$** ... 71

Figure S20. HMBC (^{1}H–^{13}C) spectrum of the reaction of **ArBi(Br)-1** with **Pd(PPh$_3$)$_4$** ... 71

Figure S21. HMBC (^{1}H–^{31}P) spectrum of the reaction of **ArBi(Br)-1** with **Pd(PPh$_3$)$_4$** ... 72

Figure S22. HSQC (^{1}H–^{13}C) spectrum of the reaction of **ArBi(Br)-1** with **Pd(PPh$_3$)$_4$** ... 72

Figure S23. No oxidative addition complexes were observed from **ArBi(OTf)-1** or **ArBi(BF$_4$)-1** with **Pd(PPh$_3$)$_4$** ... 73

Figure S24. ^{1}H NMR spectrum of the phosphonium salt side product formed via C–P coupling ... 74

Figure S25. COSY spectrum of the phosphonium salt side product formed via C–P coupling ... 74

Figure S26. ^{13}C NMR spectrum of the phosphonium salt side product formed via C–P coupling ... 75

Figure S27. HMBC (^{1}H–^{13}C) spectrum of the phosphonium salt side product formed via C–P coupling ... 75

Figure S28. HSQC (^{1}H–^{13}C) spectrum of the phosphonium salt side product formed via C–P coupling ... 76

Figure S30. HMBC (^{1}H–^{31}P) spectrum of the phosphonium salt side product formed via C–P coupling ... 77

Figure S31. Stoichiometric experiment using **ArBi^{III}X** as the electrophile with stoichiometric **Pd(PPh$_3$)$_4$** ... 78

7. Convergent Synthesis of Ambiphilic *N*,*C*,*N*-Pincer Aryl-Bismuth Compounds ... 79

Figure S32. Overview of the synthesis of ambiphiles ... 79

8. References ... 85

9. NMR spectra for Isolated Products and Substrates ... 88

1. General Experimental Details

Unless otherwise stated, all manipulations were performed under argon using standard Schlenk-line techniques or in an argon-filled glovebox.

Instruments

NMR spectra were recorded on Bruker AVIII HD 300 MHz, Bruker AVIII HD 400 MHz, or Bruker AVNeo 600 MHz NMR spectrometers and calibrated using the residual undeuterated solvent (For ^{1}H NMR: $CHCl_3$ at δ 7.26 ppm, DMSO at δ 2.50 ppm, MeCN at δ 1.94 ppm, DMF at δ 2.92 ppm, and for ^{13}C NMR: $CHCl_3$ at δ 77.16 ppm, DMSO at δ 39.52 ppm, MeCN at δ 1.32 ppm, DMF at δ 29.76 ppm) as an internal reference. Chemical shifts (δ) are reported in parts per million (ppm) and coupling constants (*J*) are reported in hertz (Hz). The following abbreviations were used to explain multiplicities of NMR spectra: s = singlet, d = doublet, t = triplet, q = quartet, m = multiplet, br = broad. Mass spectra were recorded on Bruker ESQ 3000 for ESI-MS and Shimadzu GCMS-QP2010 system for GC-MS. Accurate mass measurements were performed on a Bruker APEX III FT-MS (7 T magnet) or a Finnigan MAT 95. The chromatographic purifications were performed by flash column chromatography using Merck silica gel 60 (40–63 µm) or by preparative thin-layer chromatography (pTLC) using Merck PLC Silica gel 60 F254, 1 mm, 20 × 20 cm (Sigma-Aldrich).

Solvents and reagents

All chemicals were purchased from commercial suppliers and used without further purification unless otherwise noted. Acetonitrile (MeCN), dimethylacetamide (DMA), and dimethylformamide (DMF) were obtained as anhydrous grade from Sigma-Aldrich and stored directly in the glovebox. Molecular sieves were activated at 200 °C under a high vacuum using Schlenk-line techniques for three days. Anhydrous MeCN-d_3 was prepared by distillation over CaH_2 and stored over activated 3 Å molecular sieves. Anhydrous DMF-d_7 was prepared by storing over activated 3 Å molecular sieves, followed by degassing through argon sparging for 1 h to remove volatile impurities. Anhydrous DMF-d_7 was kept over 3 Å molecular sieves for at least three days prior to use.

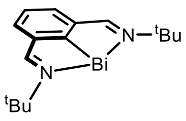

N,C,N-pincer bismuthinidene **Bi(I)**

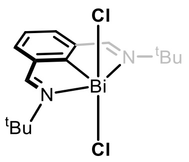

N,C,N-pincer bismuthinidene **Bi(III)·Cl₂**

Bismuth complexes such as *N*,*C*,*N*-pincer bismuthinidene **Bi(I)** and *N*,*C*,*N*-pincer bismuthinidene **Bi(III)·Cl₂** as shown above were synthesized according to a previously reported protocol.[29]

2. Procedures for the Preparation of Starting Materials

Table S1. Coupling partners: aryl iodides and aryl boron derivatives

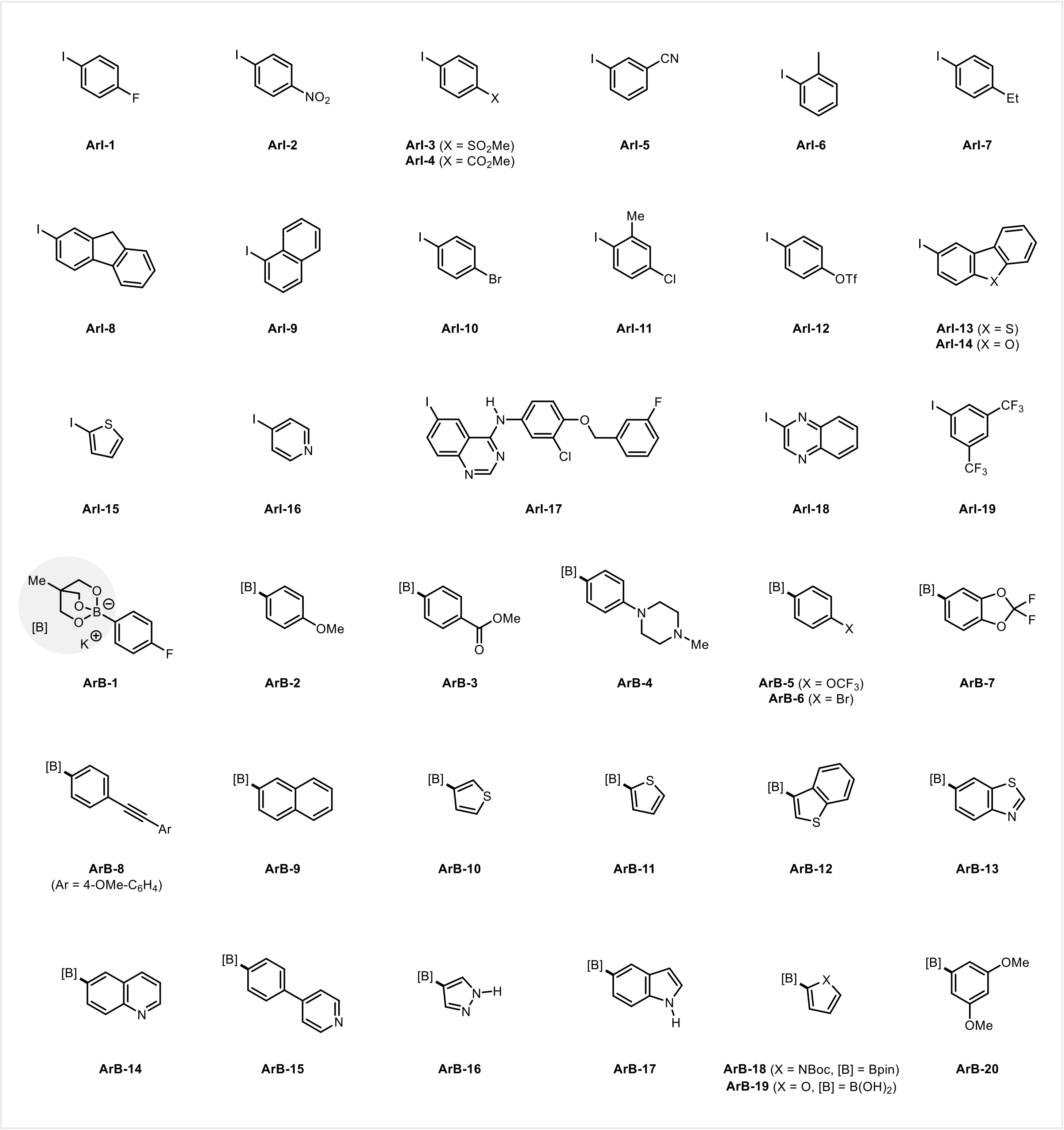

Aryl iodides (**ArI-1** to **ArI-19**) were purchased from commercial sources and used without further purification. Potassium (cyclic)triol borates (**ArB-1** to **ArB-17** and **ArB-20**) were prepared from the corresponding commercially available boronic acids or pinacol boronic esters according to the literature procedure (*General Procedure A*).[42] Aryl boronic acid derivatives (**ArB-18** and **ArB-19**) were purchased from commercial sources and used without further purification.

***General Procedure A* (GP-A)**

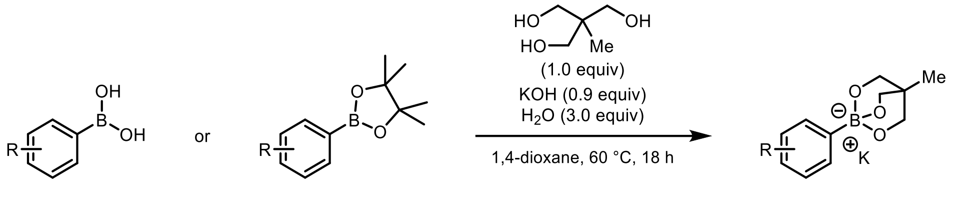

*Potassium cyclic triol borates **ArB-X** were prepared according to a slightly modified literature procedure.*[42]

A 25 mL round-bottom flask equipped with a magnetic stir bar was charged with the corresponding aryl boronic acid or boronic acid pinacol ester (3.00 mmol, 1.00 equiv.), 1,1,1-tris(hydroxymethyl)ethane (360 mg, 3.00 mmol, 1.00 equiv.), and finely ground potassium hydroxide (151 mg, 2.70 mmol, 0.900 equiv.). To this mixture, 1,4-dioxane (15 mL, 0.20 M) and H_2O (0.160 mL, 9.00 mmol, 3.00 equiv.) were added. The flask was sealed with a rubber septum and purged with argon using standard Schlenk techniques for 1 min. The reaction mixture was stirred at 60 °C overnight (approximately 16–18 h). After completion, pentane (10 mL) was added, and the resulting white precipitate was collected by filtration. If no precipitate was observed, the solvent was first evaporated and then pentane was added to induce precipitation. The filter cake was washed thoroughly with acetone (100 mL) and dried under high vacuum overnight, and subsequently used for ambiphilic cross-coupling. *Note: Due to similarities with the background signal, the ^{11}B NMR spectrum was not assigned.*

Potassium (4-fluorophenyl)triolborate (*Table S1*, ArB-1)

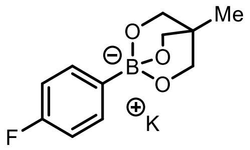

Following *General Procedure A* from the corresponding boronic acid, the desired product was obtained as a white solid.

^{1}H NMR (300 MHz, DMSO-*d*$_6$) δ 7.38 – 7.21 (m, 2H), 6.80 – 6.67 (m, 2H), 3.57 (s, 6H), 0.48 (s, 3H) ppm.
^{13}C NMR (75 MHz, DMSO-*d*$_6$) δ 160.5 (d, *J* = 237.5 Hz), 133.4 (d, *J* = 6.2 Hz), 111.8 (d, *J* = 17.9 Hz), 73.6, 34.4, 16.2 ppm.
^{19}F{^{1}H} NMR (282 MHz, DMSO-*d*$_6$) δ –119.92 ppm.
HRMS (ESI) calculated for $C_{11}H_{13}BFO_3$ [M–K]$^-$: 223.0947, found: 223.0949.

Potassium (4-methoxyphenyl)triolborate (*Table S1*, ArB-2)

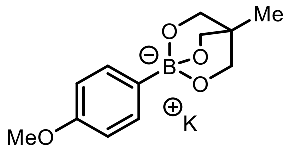

Following *General Procedure A* from the corresponding boronic acid, the desired product was obtained as a white solid.

^{1}H NMR (600 MHz, DMSO-d_6) δ 7.24 – 7.19 (m, 2H), 6.59 – 6.53 (m, 2H), 3.64 (s, 3H), 3.55 (s, 6H), 0.47 (s, 3H) ppm.
^{13}C NMR (151 MHz, DMSO-d_6) δ 156.7, 133.0, 111.2, 73.4, 54.4, 34.4, 16.3 ppm.
HRMS (ESI) calculated for $C_{12}H_{16}BO_4$ [M–K]$^-$: 235.1147, found: 235.1148.

Potassium (4-(methoxycarbonyl)phenyl)triolborate (*Table S1*, ArB-3)

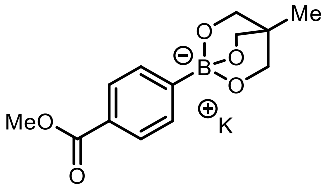

Following *General Procedure A* from the corresponding boronic acid pinacol ester, the desired product was obtained as a white solid.

^{1}H NMR (600 MHz, DMSO-d_6) δ 7.63 – 7.59 (m, 2H), 7.46 – 7.43 (m, 2H), 3.78 (s, 3H), 3.57 (s, 6H), 0.48 (s, 3H) ppm.
^{13}C NMR (151 MHz, DMSO-d_6) δ 167.4, 132.2, 126.5, 125.4, 73.7, 51.4, 34.5, 16.2 ppm.
HRMS (ESI) calculated for $C_{13}H_{16}BO_5$ [M–K]$^-$: 263.1096, found: 263.1097.

Potassium (4-(4-methylpiperazin-1-yl)phenyl)triolborate (*Table S1,* ArB-4)

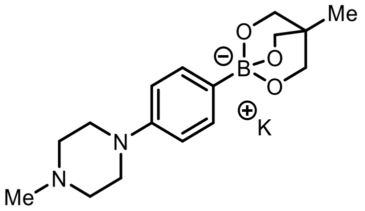

Following *General Procedure A* from the corresponding boronic acid, the desired product was obtained as a white solid.

^{1}H NMR (600 MHz, DMSO-d_6) δ 7.21 (d, J = 7.9 Hz, 2H), 6.63 (d, J = 8.2 Hz, 2H), 3.56 (s, 6H), 3.01 (t, J = 5.0 Hz, 4H), 2.42 (t, J = 5.0 Hz, 4H), 2.20 (s, 3H), 0.52 (s, 3H) ppm.
^{13}C NMR (151 MHz, DMSO-d_6) δ 133.0, 113.8, 72.4, 64.1, 54.9, 48.8, 45.8, 34.7, 16.5 ppm.
HRMS (ESI) calculated for $C_{12}H_{16}BO_4$ [M–K]$^-$: 303.1886, found: 303.1887.

Potassium (4-(trifluoromethoxy)phenyl)triolborate (*Table S1*, ArB-5)

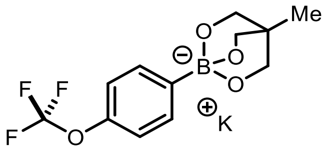

Following *General Procedure A* from the corresponding boronic acid pinacol ester, the desired product was obtained as a white solid.

^{1}H NMR (600 MHz, DMSO-*d$_6$*) δ 7.43 – 7.34 (m, 2H), 6.93 – 6.89 (m, 2H), 3.57 (s, 6H), 0.47 (s, 3H) ppm.
^{13}C NMR (151 MHz, DMSO-*d$_6$*) δ 146.1, 133.5, 120.3 (q, *J* = 254.09 Hz), 117.8, 73.6, 34.5, 16.2 ppm.
^{19}F{^{1}H} NMR (565 MHz, DMSO-*d$_6$*) δ –56.47 ppm.
HRMS (ESI) calculated for $C_{12}H_{13}BF_3O_4$ [M–K]$^-$: 289.0865, found: 289.0867.

Potassium (4-bromophenyl)triolborate (*Table S1*, ArB-6)

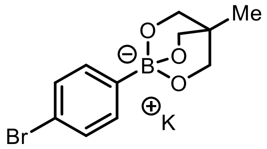

Following *General Procedure A* from the corresponding boronic acid pinacol ester, the desired product was obtained as a white solid. The spectral data are consistent with previously reported data within experimental error.[43]

^{1}H NMR (600 MHz, DMSO-*d$_6$*) δ 7.27 – 7.24 (m, 2H), 7.15 – 7.10 (m, 2H), 3.56 (s, 6H), 0.47 (s, 3H) ppm.
^{13}C NMR (151 MHz, DMSO-*d$_6$*) δ 134.5, 128.2, 117.7, 73.6, 34.5, 16.2 ppm.
HRMS (ESI) calculated for $C_{11}H_{13}BBrO_3$ [M–K]$^-$: 283.0147, found: 283.0148.

Potassium (2,2-difluorobenzo[d][1,3]dioxol-5-yl)triolborate (*Table S1*, ArB-7)

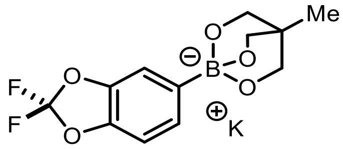

Following *General Procedure A* from the corresponding boronic acid, the desired product was obtained as a white solid.

^{1}H NMR (300 MHz, DMSO-*d$_6$*) δ 7.15 – 7.04 (m, 2H), 6.94 (d, *J* = 7.9 Hz, 1H), 3.57 (s, 6H), 0.47 (s, 3H) ppm.
^{13}C NMR (101 MHz, DMSO-*d$_6$*) δ 141.7, 140.2, 131.0 (t, *J* = 248.7 Hz), 127.0, 112.4, 107.2, 73.7, 34.5, 16.1 ppm.
^{19}F{^{1}H} NMR (282 MHz, DMSO-*d$_6$*) δ –49.44 ppm.
HRMS (ESI) calculated for $C_{12}H_{12}BF_2O_5$ [M–K]$^-$: 285.0751, found: 285.0754.

Potassium (4-((4-methoxyphenyl)ethynyl)phenyl)triolborate (*Table S1*, ArB-8)

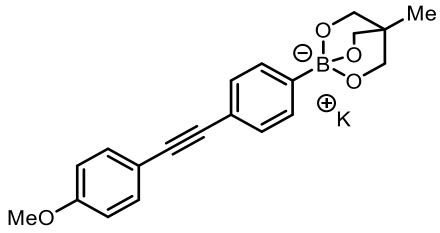

Following *General Procedure A* from the corresponding boronic acid pinacol ester, the desired product was obtained as a white solid.

^{1}H NMR (600 MHz, DMSO-d_6) δ 7.46 – 7.41 (m, 2H), 7.36 – 7.32 (m, 2H), 7.15 – 7.12 (m, 2H), 6.96 – 6.93 (m, 2H), 3.78 (s, 3H), 3.57 (s, 6H), 0.48 (s, 3H) ppm.
^{13}C NMR (101 MHz, DMSO-d_6) δ 159.0, 132.6, 132.3, 128.6, 117.8, 115.2, 114.3, 90.0, 87.2, 73.7, 55.2, 34.5, 16.2 ppm.
HRMS (ESI) calculated for $C_{20}H_{20}BO_4$ $[M–K]^-$: 335.1460, found: 333.1463.

Potassium (2-naphthyl)triolborate (*Table S1*, ArB-9)

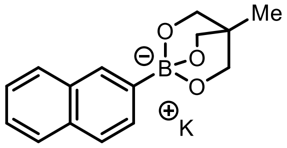

Following *General Procedure A* from the corresponding boronic acid pinacol ester, the desired product was obtained as a white solid. The spectral data are consistent with previously reported data within experimental error.[44]

^{1}H NMR (600 MHz, DMSO-d_6) δ 7.79 (s, 1H), 7.72 – 7.67 (m, 2H), 7.57 (dd, J = 8.0, 1.1 Hz, 1H), 7.51 (d, J = 8.1 Hz, 1H), 7.29 (d, J = 1.7 Hz, 2H), 3.62 (s, 6H), 0.52 (s, 3H) ppm.
^{13}C NMR (151 MHz, DMSO-d_6) δ 132.8, 131.9, 131.8, 130.5, 127.5, 127.0, 124.0, 123.4, 73.4, 34.7, 16.3 ppm.
HRMS (ESI) calculated for $C_{15}H_{16}BO_3$ $[M–K]^-$: 255.1198, found: 255.1200.

Potassium (thiophene-3-yl)triolborate (*Table S1*, ArB-10)

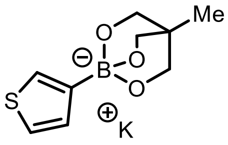

Following *General Procedure A* from the corresponding boronic acid, the desired product was obtained as a white solid. The spectral data are consistent with previously reported data within experimental error.[43]

^{1}H NMR (600 MHz, DMSO-d_6) δ 7.03 (dd, J = 4.7, 2.7 Hz, 1H), 6.94 (dd, J = 4.6, 1.0 Hz, 1H), 6.89 – 6.85 (m, 1H), 3.54 (s, 6H), 0.46 (s, 3H) ppm.
^{13}C NMR (151 MHz, DMSO-d_6) δ 132.9, 124.0, 121.1, 73.4, 34.4, 16.3 ppm.
HRMS (ESI) calculated for $C_9H_{12}BO_3S$ $[M–K]^-$: 211.0606, found: 211.0606.

Potassium (thiophene-2-yl)triolborate (*Table S1*, ArB-11)

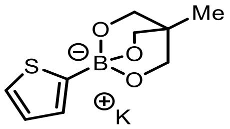

Following *General Procedure A* from the corresponding boronic acid, the desired product was obtained as a white solid.

^{1}H NMR (300 MHz, DMSO-d_6) δ 7.07 (dd, *J* = 4.6, 1.0 Hz, 1H), 6.80 (dd, *J* = 4.7, 3.2 Hz, 1H), 6.70 (dd, *J* = 3.2, 1.0 Hz, 1H), 3.54 (s, 6H), 0.46 (s, 3H) ppm.
^{13}C NMR (75 MHz, DMSO-d_6) δ 126.4, 125.9, 123.2, 73.6, 34.3, 16.1 ppm.
HRMS (ESI) calculated for $C_9H_{12}BO_3S$ [M–K]$^-$: 211.0606, found: 211.0606.

Potassium (benzo[b]thiophen-3-yl)triolborate (*Table S1*, ArB-12)

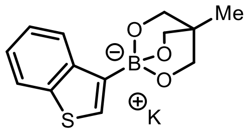

Following *General Procedure A* from the corresponding boronic acid, the desired product was obtained as an off-white solid.

^{1}H NMR (600 MHz, DMSO-d_6) δ 8.29 – 8.23 (m, 1H), 7.76 – 7.70 (m, 1H), 7.15 – 7.07 (m, 2H), 6.99 (s, 1H), 3.64 (s, 6H), 0.51 (s, 3H) ppm.
^{13}C NMR (151 MHz, DMSO-d_6) δ 145.4, 140.4, 127.6, 124.6, 122.0, 121.6, 121.1, 73.7, 34.6, 16.4 ppm.
HRMS (ESI) calculated for $C_{13}H_{14}BO_3S$ [M–K]$^-$: 261.0762, found: 261.0764.

Potassium (benzo[d]thiazol-6-yl)triolborate (*Table S1*, ArB-13)

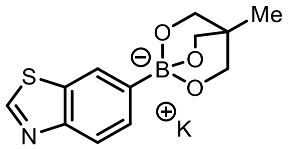

Following *General Procedure A* from the corresponding boronic acid pinacol ester, the desired product was obtained as a white solid.

^{1}H NMR (400 MHz, DMSO-d_6) δ 9.10 (s, 1H), 7.93 (s, 1H), 7.72 (d, *J* = 8.1 Hz, 1H), 7.53 (dd, *J* = 8.0, 1.0 Hz, 1H), 3.62 (s, 6H), 0.50 (s, 3H) ppm.
^{13}C NMR (101 MHz, DMSO-d_6) δ 152.1, 150.9, 131.6, 130.7, 124.6, 120.1, 73.8, 34.6, 16.2 ppm.
HRMS (ESI) calculated for $C_{12}H_{13}BNO_3S$ [M–K]$^-$: 262.0715, found: 262.0719.

Potassium (quinolin-6-yl)triolborate (*Table S1*, ArB-14)

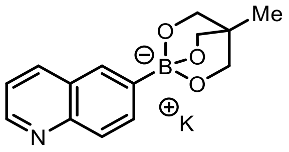

Following *General Procedure A* from the corresponding boronic acid, the desired product was obtained as a beige solid.

^{1}H NMR (600 MHz, DMSO-*d_6*) δ 9.22 (dd, *J* = 8.3, 1.8 Hz, 1H), 8.65 (dd, *J* = 4.1, 1.9 Hz, 1H), 7.62 (d, *J* = 7.4 Hz, 2H), 7.45 – 7.42 (m, 1H), 7.24 (dd, *J* = 8.4, 4.1 Hz, 1H), 3.70 (s, 6H), 0.56 (s, 3H) ppm.
^{13}C NMR (151 MHz, DMSO-*d_6*) δ 148.1, 148.0, 140.1, 131.9, 129.4, 128.0, 125.5, 118.3, 73.4, 34.8, 16.3 ppm.
HRMS (ESI) calculated for $C_{14}H_{15}BNO_3$ [M–K]$^-$: 256.1151, found: 256.1152.

Potassium (4-(pyridin-4-yl)phenyl)triolborate (*Table S1*, ArB-15)

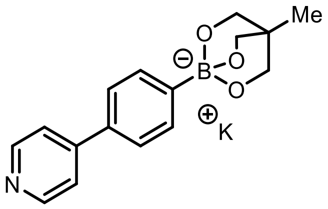

Following *General Procedure A* from the corresponding boronic acid, the desired product was obtained as an off-white solid.

^{1}H NMR (400 MHz, DMSO-*d_6*) δ 8.56 – 8.50 (m, 2H), 7.64 – 7.60 (m, 2H), 7.49 – 7.40 (m, 4H), 3.59 (s, 6H), 0.49 (s, 3H) ppm.
^{13}C NMR (101 MHz, DMSO-*d_6*) δ 150.0, 148.4, 133.0, 132.4, 123.8, 120.6, 73.7, 34.5, 16.3 ppm.
HRMS (ESI) calculated for $C_{16}H_{17}BNO_3$ [M–K]$^-$: 282.1307, found: 282.1312.

Potassium (1H-pyrazol-4-yl)triolborate (*Table S1*, ArB-16)

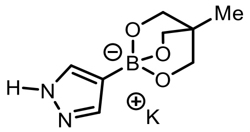

Following *General Procedure A* from the corresponding boronic acid pinacol ester, the desired product was obtained as an off-white solid.

^{1}H NMR (400 MHz, DMSO-*d_6*) δ 11.79 (brs, 1H), 7.05 (s, 2H), 3.52 (s, 6H), 0.45 (s, 3H) ppm.
^{13}C NMR (151 MHz, DMSO-*d_6*) δ 124.3, 73.3, 34.3, 16.4 ppm.
HRMS (ESI) calculated for $C_8H_{12}BN_2O_3$ [M–K]$^-$: 195.0947, found: 195.0949.

Potassium (1H-indol-5-yl)triolborate (*Table S1*, ArB-17)

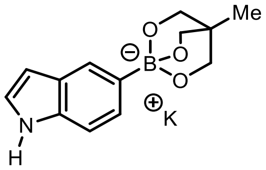

Following *General Procedure A* from the corresponding boronic acid, the desired product was obtained as a white solid.

^{1}H NMR (600 MHz, DMSO-d_6) δ 10.79 (brs, 1H), 7.54 (s, 1H), 7.17 (d, *J* = 7.5 Hz, 1H), 7.06 (d, *J* = 8.0 Hz, 1H), 7.02 – 6.98 (m, 1H), 6.18 (d, *J* = 3.0 Hz, 1H), 3.64 (s, 6H), 0.55 (s, 3H) ppm.
^{13}C NMR (151 MHz, DMSO-d_6) δ 135.0, 126.9, 126.3, 123.6, 122.9, 108.8, 100.5, 72.8, 34.7, 16.5 ppm.
HRMS (ESI) calculated for $C_{13}H_{15}BNO_3$ [M–K]$^-$: 244.1151, found: 244.1153.

Potassium (3,5-dimethoxyphenyl)triolborate (*Table S1*, ArB-20)

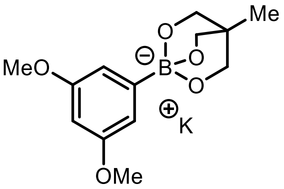

Following *General Procedure A* from the corresponding boronic acid pinacol ester, the desired product was obtained as a white solid.

^{1}H NMR (600 MHz, DMSO-d_6) δ 6.50 (d, *J* = 2.5 Hz, 2H), 6.02 (t, *J* = 2.5 Hz, 1H), 3.63 (s, 6H), 3.56 (s, 6H), 0.47 (s, 3H) ppm.
^{13}C NMR (151 MHz, DMSO-d_6) δ 158.6, 109.0, 97.6, 73.6, 54.5, 34.5, 16.3 ppm.
HRMS (ESI) calculated for $C_{13}H_{18}BO_5$ [M–K]$^-$: 265.1253, found: 265.1251.

Table S2. Aryl-bismuth compounds and aryl thianthrenium salts

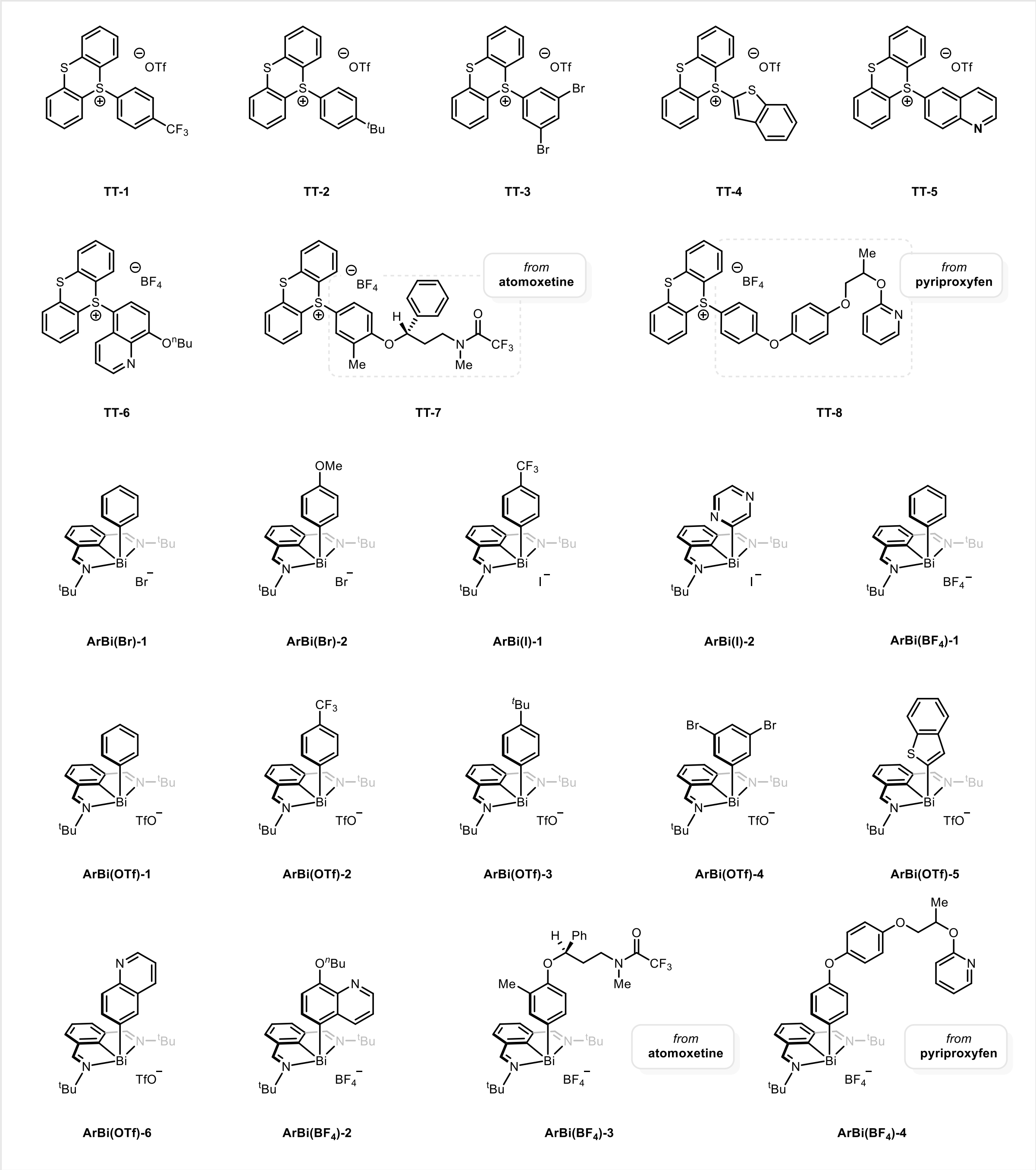

Aryl thianthrenium salts (**TT-1** to **TT-8**) were synthesized according to literature procedures.[45,46] Aryl-bismuth bromides (**ArBi(Br)-1** and **ArBi(Br)-2**) were prepared following *General Procedure B*. Aryl-bismuth iodides (**ArBi(I)-1** and **ArBi(I)-2**) were synthesized according to our previously reported procedure (*General Procedure C*).[32] **ArBi(BF$_4$)-1** and **ArBi(OTf)-1** were obtained by anion exchange of **ArBi(Br)-1** with the corresponding silver salts (*General Procedure D*). Aryl-bismuth triflates (**ArBi(OTf)-2** to **ArBi(OTf)-6**) and tetrafluoroborates (**ArBi(BF$_4$)-2** to **ArBi(BF$_4$)-4**) were prepared following our previously reported method (*General Procedure E*).[32]

***General Procedure B* (GP-B)**

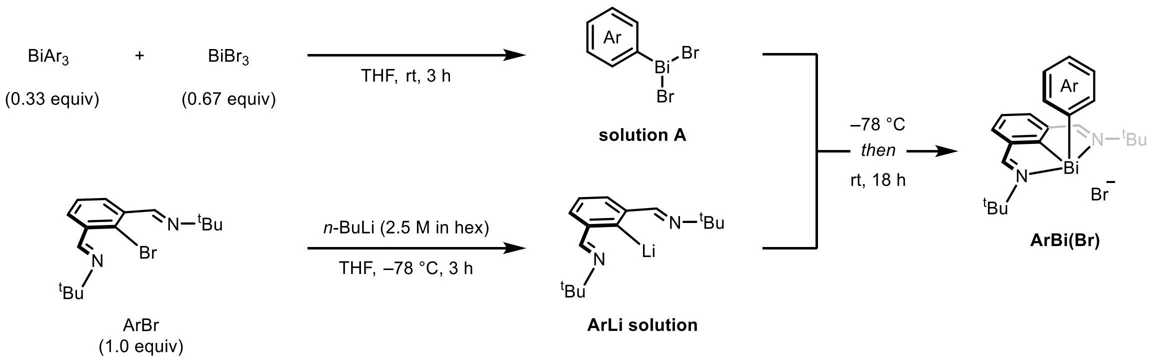

Aryl bromide (ArBr) as shown above was prepared according to a literature procedure.[34]

Solution A: A heat-gun-dried 250 mL Schlenk flask equipped with a Teflon-coated magnetic stir bar was charged with $BiBr_3$ (2.99 g, 6.66 mmol, 0.667 equiv.) in an argon-filled glovebox. After removal from the glovebox, anhydrous THF (45 mL) was added, and the mixture was stirred for 5 min to afford a homogeneous bright yellow solution. $BiAr_3$ (3.33 mmol, 0.333 equiv.) was then added in one portion at room temperature. The reaction mixture was stirred for 3 h and subsequently cooled to –78 °C prior to the addition of the aryllithium solution.

Aryllithium solution (ArLi): In a separate heat-gun dried 250 mL Schlenk flask equipped with a PTFE-coated magnetic stir bar, aryl bromide (3.23 g, 10.0 mmol, 1.00 equiv.) was added under a positive flow of argon. Dry THF (120 mL) was added to afford a homogeneous pale yellow solution, which was cooled to –78 °C. To this chilled solution, *n*-BuLi (4.100 mL, 2.5 M in hexanes, 10.25 mmol, 1.025 equiv.) was added dropwise, resulting in a color change to dark orange/brown. After complete addition, the reaction mixture was stirred at –78 °C for 3 h.

After 3 h, the ***aryllithium solution (ArLi)*** was transferred via cannula into ***Solution A*** in one portion at –78 °C, affording a yellow suspension. The reaction mixture was stirred at –78 °C for 10 min, then allowed to warm to room temperature and stirred for an additional 18 h. Upon completion, the suspension was filtered through a fritted funnel, and the solid was washed with THF (3 × 40 mL). The resulting solid was transferred to a Celite-packed funnel and further washed with $CHCl_3$. The combined filtrates were concentrated by rotary evaporation to a final volume of approximately 10 mL, and pentane was added to induce precipitation. The resulting precipitate was collected by filtration, washed with THF, and dried under high vacuum to afford the desired product as an off-white solid (2.160 g, 60%).

[(2,6-(^{t}BuNCH)$_2$C$_6$H$_3$)Bi(phenyl)(bromide)] (*Table S2*, ArBi(Br)-1)

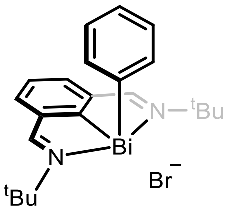

Following *General Procedure B*, the title compound **ArBi(Br)-1** was obtained from $BiBr_3$ (2.99 g, 6.66 mmol, 0.667 equiv.) and $BiPh_3$ (1.47 g, 3.33 mmol, 0.333 equiv.) in anhydrous THF (45 mL), together with an aryllithium solution generated from ArBr (3.23 g, 10.0 mmol, 1.00 equiv.) and *n*-BuLi (4.100 mL, 2.5 M in hexanes, 10.25 mmol, 1.025 equiv.) in anhydrous THF (120 mL), to give the product as an off-white solid (2.160 g, 60%).

^{1}H NMR (600 MHz, DMF-d_7) δ 10.18 (s, 2H), 8.57 (d, *J* = 7.6 Hz, 2H), 8.35 (dd, *J* = 8.0, 1.3 Hz, 2H), 8.16 (t, *J* = 7.5 Hz, 1H), 7.61 – 7.57 (m, 2H), 7.46 – 7.40 (m, 1H), 1.39 (s, 18H) ppm.

^{13}C NMR (151 MHz, DMF-d_7) δ 191.5, 181.6, 168.4, 149.2, 138.3, 136.9, 132.0, 130.5, 129.3, 61.5, 30.5 ppm.

HRMS (ESI) calculated for $C_{22}H_{28}BiN_2$ $[M–Br]^+$: 529.2051, found: 529.2049.

[(2,6-(^{t}BuNCH)$_2$C$_6$H$_3$)Bi(4-methoxyphenyl)(bromide)] (*Table S2*, ArBi(Br)-2)

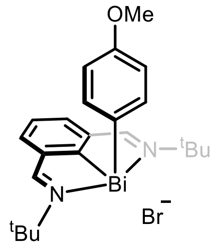

Following *General Procedure B*, the title compound **ArBi(Br)-2** was obtained from $BiBr_3$ (237 mg, 0.528 mmol, 0.667 equiv.) and $BiAr_3$ (140 mg, 0.264 mmol, 0.333 equiv.) in anhydrous THF (10 mL), together with an aryllithium solution generated from ArBr (256 mg, 0.792 mmol, 1.00 equiv.) and *n*-BuLi (0.320 mL, 2.5 M in hexanes, 0.808 mmol, 1.02 equiv.) in anhydrous THF (10 mL), to give the product as an off-white solid (180 mg, 36%).

^{1}H NMR (600 MHz, DMF-d_7) δ 10.09 (s, 2H), 8.52 (d, *J* = 7.5 Hz, 2H), 8.20 – 8.17 (m, 2H), 8.15 (t, *J* = 7.5 Hz, 1H), 7.10 – 7.06 (m, 2H), 3.75 (s, 3H), 1.37 (s, 18H) ppm.

^{13}C NMR (151 MHz, DMF-d_7) δ 190.3, 172.3, 168.2, 160.5, 149.1, 140.3, 136.9, 130.6, 117.9, 61.5, 55.1, 30.5 ppm.

HRMS (ESI) calculated for $C_{22}H_{30}BiN_2O$ $[M–Br]^+$: 559.2157, found: 559.2161.

***General Procedure C* (GP-C)**

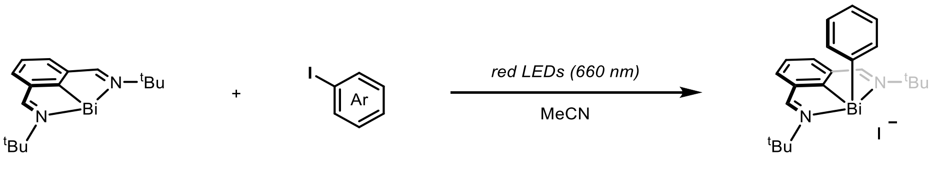

Aryl-bismuth reagents from aryl iodides were prepared according to a slightly modified literature procedure.[32]

An oven-dried 10 mL culture tube equipped with a Teflon-coated magnetic stir bar was charged with *N*,*C*,*N*-pincer bismuthinidene **Bi(I)** (1.0 equiv.) and the corresponding aryl iodide **ArI** (1.1–2.0 equiv.). Anhydrous, degassed acetonitrile was then added, and the tube was sealed with a screw cap under an argon atmosphere in a glovebox. After removal from the glovebox, the mixture was stirred under red-light irradiation. After the specified reaction time, the mixture was concentrated by rotary evaporation. The residue was re-dissolved in acetonitrile and filtered through a short pad of Celite. The filtrate was concentrated, and diethyl ether was added to induce precipitation. The precipitate was collected by filtration, washed with diethyl ether, and dried under high vacuum to give the desired product as a yellowish solid.

[(2,6-(^{t}BuNCH)$_2$C$_6$H$_3$)Bi(4-trifluoromethylphenyl)(iodide)] (*Table S2*, ArBi(I)-1)

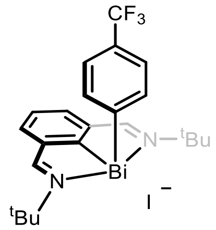

Following *General Procedure C*, the title compound **ArBi(I)-1** was obtained from *N*,*C*,*N*-pincer bismuthinidene **Bi(I)** (9.1 mg, 0.020 mmol, 1.0 equiv.) and 4-iodobenzotrifluoride (5.9 µL, 0.040 mmol, 2.0 equiv.) in anhydrous CD_3CN (0.6 mL) after 12 h of red LED irradiation (660 nm) (93% NMR yield). Alternatively, the title compound was obtained from *N*,*C*,*N*-pincer bismuthinidene **Bi(I)** (68 mg, 0.15 mmol, 1.0 equiv.) and 4-iodobenzotrifluoride (44 µL, 0.30 mmol, 2.0 equiv.) in anhydrous acetonitrile (2.0 mL) after 7 days of red LED irradiation, affording the product as a yellow solid (55 mg, 55%), which was subsequently used for ambiphilic cross-coupling. The spectral data are consistent with previously reported data within experimental error.[32]

^{1}H NMR (600 MHz, CD_3CN) δ 9.75 (s, 2H), 8.34 (d, *J* = 7.6 Hz, 2H), 8.26 (dt, *J* = 7.5, 0.8 Hz, 2H), 8.09 (t, *J* = 7.6 Hz, 1H), 7.84 – 7.64 (m, 2H), 1.30 (s, 18H) ppm.
^{13}C NMR (151 MHz, CD_3CN) δ 188.4, 183.7, 168.9, 149.5, 139.6, 137.7, 131.9, 131.4 (q, *J* = 32.4 Hz), 129.2 (q, *J* = 3.8 Hz), 125.4 (q, *J* = 284.1 Hz), 62.4, 31.0 ppm.
^{19}F{^{1}H} NMR (565 MHz, CD_3CN) δ –63.56 ppm.
HRMS (ESI) calculated for $C_{23}H_{37}BiF_2N_2$ $[M–I]^+$: 597.1925, found: 597.1930.

[(2,6-(^{t}BuNCH)$_2$C$_6$H$_3$)Bi(pyrazin-2-yl)(iodide)] (*Table S2*, ArBi(I)-2)

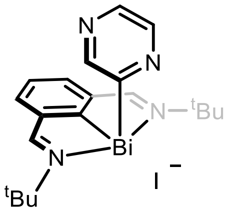

Following *General Procedure C*, the title compound **ArBi(I)-2** was obtained from *N*,*C*,*N*-pincer bismuthinidene **Bi(I)** (9.1 mg, 0.020 mmol, 1.0 equiv.) and 2-iodopyrazine (4.0 µL mg, 0.040 mmol, 2.0 equiv.) in anhydrous CD_3CN (0.6 mL) after 24 h of red LED irradiation (660 nm) (87% NMR yield). Alternatively, the title compound was obtained from *N*,*C*,*N*-pincer bismuthinidene **Bi(I)** (68 mg, 0.15 mmol, 1.0 equiv.) and 2-iodopyrazine (30 µL, 0.30 mmol, 2.0 equiv.) in anhydrous acetonitrile (2.0 mL) after 7 days of red LED irradiation, affording the product as a yellow solid (46 mg, 47%), which was subsequently used for ambiphilic cross-coupling. The spectral data are consistent with previously reported data within experimental error.[47]

^{1}H NMR (600 MHz, CD_3CN) δ 9.86 (s, 2H), 8.74 (d, *J* = 2.5 Hz, 1H), 8.50 (dd, *J* = 2.5, 1.6 Hz, 1H), 8.35 (d, *J* = 7.6 Hz, 2H), 8.08 (d, *J* = 1.6 Hz, 1H), 8.07 (t, *J* = 7.6 Hz, 1H), 1.34 (s, 18H) ppm.
^{13}C NMR (151 MHz, CD_3CN) δ 211.2, 188.5, 169.6, 157.2, 150.1, 149.7, 145.3, 137.7, 131.6, 62.3, 31.0 ppm.
HRMS (ESI) calculated for $C_{20}H_{26}BiN_2$ $[M–I]^+$: 531.1956, found: 531.1959.

General Procedure D **(GP-D)**

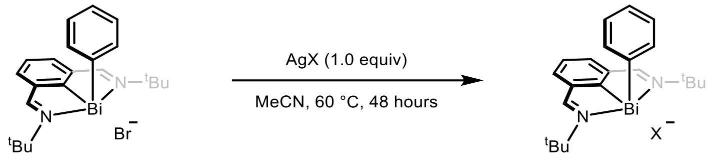

ArBi(Br)-1 **ArBi(X)-1**

A heat-gun dried 100 mL Schlenk flask equipped with a Teflon-coated magnetic stir bar was charged with AgX (1.0 equiv.), and anhydrous acetonitrile (0.050 M) was added under an argon atmosphere in an argon-filled glovebox. After removal from the glovebox, the mixture was stirred at 60 °C for two days. Upon completion, the reaction mixture was filtered through a short pad of Celite and washed with acetonitrile. The combined filtrate was concentrated using a rotary evaporator. The resulting residue was transferred to a 20 mL vial and further dried under high vacuum to afford the desired product as a solid.

[(2,6-(^{t}BuNCH)$_2$C$_6$H$_3$)Bi(phenyl)(BF$_4$)] (*Table S2*, ArBi(BF$_4$)-1)

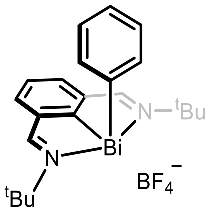

Following *General Procedure D*, the title compound **ArBi(BF$_4$)-1** was obtained from **ArBi(Br)-1** (914 mg, 1.50 mmol, 1.00 equiv.) and silver tetrafluoroborate (AgBF$_4$) (307 mg, 1.05 equiv., 1.00 equiv.) in anhydrous acetonitrile (30 mL) after 48 h at 60 °C, affording the product as an off-white solid (881 mg, 95%).

^{1}H NMR (300 MHz, DMF-*d$_7$*) δ 10.08 (s, 2H), 8.52 (d, *J* = 7.6 Hz, 2H), 8.31 – 8.25 (m, 2H), 8.17 (dd, *J* = 7.8, 7.3 Hz, 1H), 7.65 – 7.55 (m, 2H), 7.48 – 7.40 (m, 1H), 1.37 (s, 18H) ppm.
^{13}C NMR (101 MHz, DMF-*d$_7$*) δ 190.5, 180.7, 168.4, 149.1, 138.2, 137.0, 132.1, 130.7, 129.5, 61.6, 30.5 ppm.
^{19}F{^{1}H} NMR (282 MHz, DMF-*d$_7$*) δ –150.78, –150.83 ppm.
HRMS (ESI) calculated for C$_{22}$H$_{28}$BiN$_2$ [M–BF$_4$]$^+$: 529.2051, found: 529.2056.

[(2,6-(^{t}BuNCH)$_2$C$_6$H$_3$)Bi(phenyl)(OTf)] (*Table S2*, ArBi(OTf)-1)

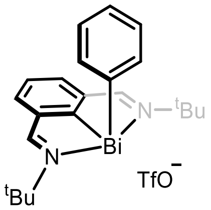

Following *General Procedure D*, the title compound **ArBi(OTf)-1** was obtained from **ArBi(Br)-1** (122 mg, 0.200 mmol, 1.00 equiv.) and silver trifluoromethanesulfonate (AgOTf) (52 mg, 0.20 mmol, 1.0 equiv.) in anhydrous acetonitrile (4.0 mL) after 41 h at 60 °C, affording the product as an beige solid (121 mg, 89%).

^{1}H NMR (300 MHz, DMF-*d$_7$*) δ 10.08 (s, 2H), 8.52 (d, J = 7.6 Hz, 2H), 8.33 – 8.24 (m, 2H), 8.17 (dd, *J* = 7.8, 7.3 Hz, 1H), 7.64 – 7.55 (m, 2H), 7.49 – 7.36 (m, 1H), 1.37 (s, 18H) ppm.
^{13}C NMR (101 MHz, DMF-*d$_7$*) δ 190.5, 180.7, 168.4, 149.1, 138.2, 137.0, 132.1, 130.7, 129.5, 61.6, 30.5 ppm.
^{19}F{^{1}H} NMR (282 MHz, DMF-*d$_7$*) δ –78.66 ppm.
HRMS (ESI) calculated for C$_{22}$H$_{28}$BiN$_2$ [M–OTf]$^+$: 529.2051, found: 529.2054.

***General Procedure E* (GP-E)**

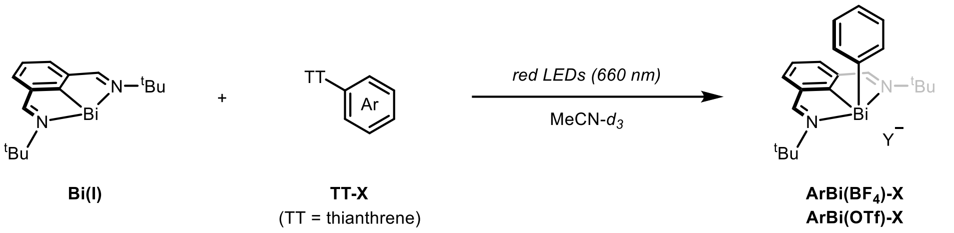

Aryl-bismuth compounds from thianthrenium salts were prepared according to a literature procedure.[32]

An oven-dried 10 mL culture tube equipped with a Teflon-coated magnetic stir bar was charged with *N,C,N*-pincer bismuthinidene **Bi(I)** (1.0 equiv.) and the corresponding aryl thianthrenium salt **TT–X** (1.0 equiv.). Anhydrous and degassed MeCN-*d₃* was then added, and the reaction tube was sealed with a screw cap under an argon atmosphere inside a glovebox. After removal from the glovebox, the reaction mixture was stirred under red-light irradiation for the specified time. The reaction yield was determined by ^{1}H NMR analysis using mesitylene (1.0 equiv.) as an internal standard. After yield determination, the mixture was concentrated under reduced pressure and further dried under high vacuum for 2 h. The crude residue was then directly subjected to the ambiphilic cross-coupling reaction without further purification.

[(2,6-(^{t}BuNCH)$_2$C$_6$H$_3$)Bi(4-trifluoromethylphenyl)(OTf)] (*Table S2*, ArBi(OTf)-2)

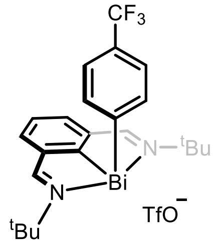

Following *General Procedure E*, the title compound **ArBi(OTf)-2** was obtained from *N*,*C*,*N*-pincer bismuthinidene **Bi(I)** (9.1 mg, 0.020 mmol, 1.0 equiv.) and the corresponding thianthrenium salt **TT-1** (11.2 mg, 0.0220 mmol, 1.10 equiv.) in anhydrous MeCN-*d₃* (1.0 mL) after 10 min of red LED irradiation (660 nm) (95% NMR yield) and used for characterization. Alternatively, the title compound was obtained from *N*,*C*,*N*-pincer bismuthinidene **Bi(I)** (45 mg, 0.10 mmol, 1.0 equiv.) and the corresponding thianthrenium salt **TT-1** (51 mg, 0.10 mmol, 1.0 equiv.) in anhydrous MeCN-*d₃* (2.0 mL) after 1 h of red LED irradiation. The NMR yield was determined using mesitylene (10.0 µL, 0.0719 mmol, 0.719 equiv.) as an internal standard (90% NMR yield). After evaporation of the solvent, the title compound was dried under high vacuum for 1 h and directly used in the ambiphilic cross-coupling without further purification.

^{1}H NMR (600 MHz, CD_3CN) δ 9.75 (s, 2H), 8.34 (d, *J* = 7.6 Hz, 2H), 8.27 – 8.22 (m, 2H), 8.11 – 8.05 (m, 1H), 7.77 – 7.75 (m, 2H) 1.29 (s, 18H) ppm.
^{13}C NMR (151 MHz, CD_3CN) δ 188.2, 183.3, 168.9, 149.5, 139.5, 137.7, 131.9, 131.5 (q, *J* = 32.3 Hz), 129.2 (q, *J* = 3.8 Hz), 125.3 (q, *J* = 271.7 Hz), 122.2 (q, *J* = 321.1 Hz), 118.3, 62.4, 31.0, 1.3 ppm.
^{19}F{^{1}H} NMR (565 MHz, CD_3CN) δ –63.54, –79.31 ppm.
HRMS (ESI) calculated for $C_{23}H_{27}BiF_3N_2$ [M–OTf]$^+$: 597.1925, found: 597.1926.

[(2,6-(^{t}BuNCH)$_2$C$_6$H$_3$)Bi(4-*tert*-butylphenyl)(OTf)] (*Table S2*, ArBi(OTf)-3)

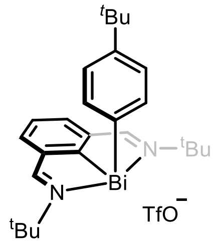

Following *General Procedure E*, the title compound **ArBi(OTf)-3** was obtained from *N*,*C*,*N*-pincer bismuthinidene **Bi(I)** (9.1 mg, 0.020 mmol, 1.0 equiv.) and the corresponding thianthrenium salt **TT-2** (11 mg, 0.022 mmol, 1.1 equiv.) in anhydrous MeCN-d_3 (1.0 mL) after 10 min of red LED irradiation (660 nm) (90% NMR yield) and used for characterization. Alternatively, the title compound was obtained from *N*,*C*,*N*-pincer bismuthinidene **Bi(I)** (45 mg, 0.10 mmol, 1.0 equiv.) and the corresponding thianthrenium salt **TT-2** (50 mg, 0.10 mmol, 1.0 equiv.) in anhydrous MeCN-d_3 (2.0 mL) after 2 h of red LED irradiation. The NMR yield was determined using mesitylene (10.0 µL, 0.0719 mmol, 0.719 equiv.) as an internal standard (83% NMR yield). After evaporation of the solvent, the title compound was dried under high vacuum for 1 h and directly used in the ambiphilic cross-coupling without further purification.

^{1}H NMR (600 MHz, CD$_3$CN) δ 9.69 (s, 2H), 8.30 (d, *J* = 7.6 Hz, 2H), 8.06 (app. dd, *J* = 7.4 Hz, 1H), 7.99 – 7.96 (m, 2H), 7.57 – 7.52 (m, 2H), 1.29 (s, 18H), 1.20 (s, 9H) ppm.

^{13}C NMR (151 MHz, CD$_3$CN) δ 187.4, 174.9, 168.5, 138.5, 137.5, 131.6, 122.2 (q, J = 321.1 Hz), 62.2, 35.6, 31.3, 31.0 ppm.

^{19}F{^{1}H} NMR (565 MHz, CD$_3$CN) δ –79.30 ppm.

HRMS (ESI) calculated for $C_{26}H_{36}BiN_2$ [M–OTf]$^+$: 585.2677, found: 585.2678.

[(2,6-(^{t}BuNCH)$_2$C$_6$H$_3$)Bi(3,5-dibromophenyl)(OTf)] (*Table S2*, ArBi(OTf)-4)

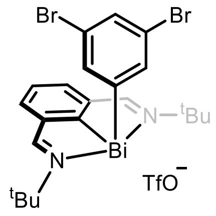

Following *General Procedure E*, the title compound **ArBi(OTf)-4** was obtained from *N*,*C*,*N*-pincer bismuthinidene **Bi(I)** (9.1 mg, 0.020 mmol, 1.0 equiv.) and the corresponding thianthrenium salt **TT-3** (13.2 mg, 0.022 mmol, 1.1 equiv.) in anhydrous MeCN-d_3 (1.0 mL) after 1 h of red LED irradiation (660 nm) (83% NMR yield) and used for characterization. Alternatively, the title compound was obtained from *N*,*C*,*N*-pincer bismuthinidene **Bi(I)** (45 mg, 0.10 mmol, 1.0 equiv.) and the corresponding thianthrenium salt **TT-3** (60 mg, 0.10 mmol, 1.0 equiv.) in anhydrous MeCN-d_3 (2.0 mL) after 2 h of red LED irradiation. The NMR yield was determined using mesitylene (10.0 µL, 0.0719 mmol, 0.719 equiv.) as an internal standard (82% NMR yield). After evaporation of the solvent, the title compound was dried under high vacuum for 1 h and directly used in the ambiphilic cross-coupling without further purification.

^{1}H NMR (600 MHz, CD$_3$CN) δ 9.76 (s, 2H), 8.36 (d, *J* = 7.6 Hz, 2H), 8.16 (d, *J* = 1.8 Hz, 2H), 8.12 – 8.07 (m, 1H), 7.70 – 7.69 (m, 1H), 1.31 (s, 18H) ppm.

^{13}C NMR (151 MHz, CD$_3$CN) δ 188.7, 182.1, 169.0, 149.5, 139.5, 137.8, 135.6, 132.1, 128.8, 122.1 (q, *J* = 320.8 Hz), 62.5, 30.9 ppm.

^{19}F{^{1}H} NMR (565 MHz, CD$_3$CN) δ –79.30 ppm.

HRMS (ESI) calculated for $C_{22}H_{26}BiBr_2N_2$ [M–OTf]$^+$: 685.0261, found: 685.0268.

[(2,6-(^{t}BuNCH)$_2$C$_6$H$_3$)Bi(2-benzo[b]thiophenyl)(OTf)] (*Table S2*, ArBi(OTf)-5)

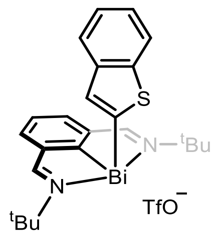

Following *General Procedure E*, the title compound **ArBi(OTf)-5** was obtained from *N*,*C*,*N*-pincer bismuthinidene **Bi(I)** (9.1 mg, 0.020 mmol, 1.0 equiv.) and the corresponding thianthrenium salt **TT-4** (11 mg, 0.022 mmol, 1.1 equiv.) in anhydrous MeCN-d_3 (1.0 mL) after 1 h of red LED irradiation (660 nm) (70% NMR yield, with other minor product) and used for characterization. Alternatively, the title compound was obtained from *N*,*C*,*N*-pincer bismuthinidene **Bi(I)** (45 mg, 0.10 mmol, 1.0 equiv.) and the corresponding thianthrenium salt **TT-4** (50 mg, 0.10 mmol, 1.0 equiv.) in anhydrous MeCN-d_3 (2.0 mL) after 4 h of red LED irradiation. The NMR yield was determined using mesitylene (10.0 µL, 0.0719 mmol, 0.719 equiv.) as an internal standard (75% NMR yield). After evaporation of the solvent, the title compound was dried under high vacuum for 1 h and directly used in the ambiphilic cross-coupling without further purification.

^{1}H NMR (600 MHz, CD$_3$CN) δ 9.75 (s, 2H), 8.35 (d, *J* = 7.6 Hz, 2H), 8.18 (d, *J* = 0.8 Hz, 1H), 8.10 (dd, *J* = 7.8, 7.4 Hz, 1H), 7.85 – 7.82 (m, 1H), 7.76 – 7.72 (m, 1H), 7.38 – 7.30 (m, 2H), 1.33 (s, 18H) ppm.

^{13}C NMR (151 MHz, CD$_3$CN) δ 185.2, 176.1, 168.9, 150.2, 147.4, 142.9, 138.4, 137.3, 126.6, 125.7, 124.6, 123.7, 123.1, 122.1 (q, *J* = 321.2 Hz), 118.3, 62.5, 30.9, 1.3 ppm.

^{19}F NMR (565 MHz, CD$_3$CN) δ –79.31 ppm.

HRMS (ESI) calculated for $C_{24}H_{28}BiN_2S$ [M–OTf]$^+$: 585.1772, found: 585.1777.

[(2,6-(^{t}BuNCH)$_2$C$_6$H$_3$)Bi(6-quinolinyl)(OTf)] (*Table S2*, ArBi(OTf)-6)

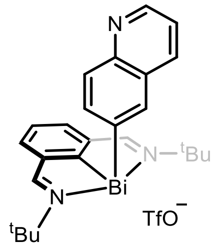

Following *General Procedure E*, the title compound **ArBi(OTf)-6** was obtained from *N*,*C*,*N*-pincer bismuthinidene **Bi(I)** (9.1 mg, 0.020 mmol, 1.0 equiv.) and the corresponding thianthrenium salt **TT-5** (10.9 mg, 0.022 mmol, 1.1 equiv.) in anhydrous MeCN-d_3 (1.0 mL) after 1h of red LED irradiation (660 nm) (94% NMR yield) and used for characterization. Alternatively, the title compound was obtained from *N*,*C*,*N*-pincer bismuthinidene **Bi(I)** (22.6 mg, 0.0500 mmol, 1.00 equiv.) and the corresponding thianthrenium salt **TT-5** (24.7 mg, 0.05 mmol, 1.0 equiv.) in anhydrous MeCN-d_3 (1.0 mL) after 4 h of red LED irradiation. The NMR yield was determined using mesitylene (5.00 µL, 0.0359 mmol, 0.719 equiv.) as an internal standard (88% NMR yield). After evaporation of the solvent, the title compound was dried under high vacuum for 1 h and directly used in the ambiphilic cross-coupling without further purification.

^{1}H NMR (600 MHz, CD$_3$CN) δ 9.74 (s, 2H), 8.85 (dd, *J* = 4.2, 1.7 Hz, 1H), 8.78 (d, *J* = 1.4 Hz, 1H), 8.36 (d, *J* = 7.6 Hz, 2H), 8.19 (dd, *J* = 8.6, 1.4 Hz, 1H), 8.15 – 8.07 (m, 3H), 7.49 (dd, *J* = 8.4, 4.2 Hz, 1H), 1.28 (s, 18H) ppm.

^{13}C NMR (151 MHz, CD$_3$CN) δ 187.9, 176.5, 168.8, 152.7, 149.5, 148.9, 139.7, 138.5, 137.7, 137.0, 133.6, 132.1, 131.8, 123.1, 122.2 (q, *J* = 321.8 Hz), 62.4, 31.0 ppm.

^{19}F{^{1}H} NMR (565 MHz, CD_3CN) δ –79.29 ppm.
HRMS (ESI) calculated for $C_{25}H_{29}BiN_3$ [M–OTf]$^{+}$: 580.2160, found: 580.2166.

[(2,6-(^{t}BuNCH)$_2$C$_6$H$_3$)Bi(8-butoxyquinolin-5-yl)(BF$_4$)] (*Table S2*, ArBi(BF$_4$)-2)

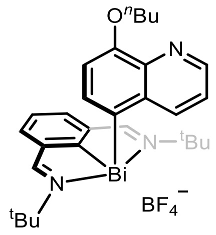

Following *General Procedure E*, the title compound **ArBi(BF$_4$)-2** was obtained from *N*,*C*,*N*-pincer bismuthinidene **Bi(I)** (9.1 mg, 0.020 mmol, 1.0 equiv.) and the corresponding thianthrenium salt **TT-6** (11 mg, 0.022 mmol, 1.1 equiv.) in anhydrous MeCN-*d$_3$* (1.0 mL) after 10 min of red LED irradiation (660 nm) (77% NMR yield, with ca. 25% of other minor product) and used for characterization. Alternatively, the title compound was obtained from *N*,*C*,*N*-pincer bismuthinidene **Bi(I)** (22.6 mg, 0.0500 mmol, 1.00 equiv.) and the corresponding thianthrenium salt **TT-6** (25.2 mg, 0.0500 mmol, 1.00 equiv.) in anhydrous MeCN-*d$_3$* (1.0 mL) after 4 h of red LED irradiation. The NMR yield was determined using mesitylene (5.00 µL, 0.0359 mmol, 0.719 equiv.) as an internal standard (75% NMR yield). After evaporation of the solvent, the title compound was dried under high vacuum for 1 h and directly used in the ambiphilic cross-coupling without further purification.

^{1}H NMR (600 MHz, CD_3CN) δ 9.67 (s, 2H), 8.96 (dd, *J* = 4.2, 1.5 Hz, 1H), 8.78 (d, *J* = 8.4 Hz, 1H), 8.35 (d, *J* = 7.6 Hz, 2H), 8.13 (dd, *J* = 7.8, 7.4 Hz, 1H), 8.05 (d, *J* = 8.0 Hz, 1H), 7.70 (dd, *J* = 8.4, 4.2 Hz, 1H), 7.01 (d, *J* = 8.0 Hz, 1H), 4.06 (t, *J* = 6.6 Hz, 2H), 1.85 – 1.78 (m, 2H), 1.53 – 1.44 (m, 3H), 1.15 (s, 18H), 0.96 (t, *J* = 7.4 Hz, 3H) ppm.
^{13}C NMR (151 MHz, CD_3CN) δ 185.3, 170.3, 168.0, 157.8, 150.2, 149.3, 140.1, 138.8, 137.7, 135.7, 132.1, 123.6, 114.5, 69.5, 62.0, 31.9, 30.9, 20.0, 14.1 ppm.
^{19}F{^{1}H} NMR (565 MHz, CD_3CN) δ –151.70 ppm.
^{11}B NMR (193 MHz, CD_3CN) δ –1.20 ppm.
HRMS (ESI) calculated for $C_{25}H_{29}BiN_3$ [M–BF$_4$]$^{+}$: 580.2160, found: 580.2166.

[(2,6-(^{t}BuNCH)$_2$C$_6$H$_3$)Bi(*atomoxetine*)(BF$_4$)] (*Table S2*, ArBi(BF$_4$)-3)

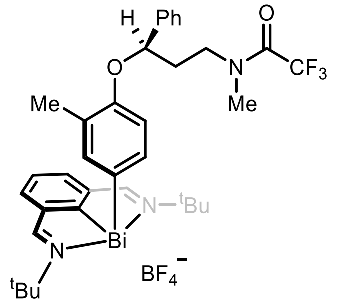

Following *General Procedure E*, the title compound **ArBi(BF$_4$)-3** was obtained from *N*,*C*,*N*-pincer bismuthinidene **Bi(I)** (9.1 mg, 0.020 mmol, 1.0 equiv.) and the corresponding thianthrenium salt **TT-7** (14.4 mg, 0.022 mmol, 1.1 equiv.) in anhydrous MeCN-*d$_3$* (1.0 mL) after 10 min of red LED irradiation (660 nm) (80% NMR yield) and used for characterization. Alternatively, the title compound was obtained from *N*,*C*,*N*-pincer bismuthinidene **Bi(I)** (22.6 mg, 0.0500 mmol, 1.00 equiv.) and the corresponding thianthrenium salt **TT-7** (32.7 mg, 0.100 mmol, 1.00 equiv.) in anhydrous MeCN-*d$_3$* (1.0 mL) after 4 h of red LED irradiation. The NMR yield

was determined using mesitylene (5.00 µL, 0.0359 mmol, 0.719 equiv.) as an internal standard (71% NMR yield). After evaporation of the solvent, the title compound was dried under high vacuum for 1 h and directly used in the ambiphilic cross-coupling without further purification. The spectral data are consistent with previously reported data within experimental error.[32]

^{1}H NMR (600 MHz, CD_3CN, *mixture of rotamers*) δ 9.63 (s, 1H), 9.61 (s, 1H), 8.27 (dd, *J* = 7.6, 1.0 Hz, 1H), 8.25 (dd, *J* = 7.6, 1.0 Hz, 1H), 8.03 (t, *J* = 7.6 Hz, 1H), 7.79 (dd, *J* = 1.7, 0.8 Hz, 0.5H), 7.78 (dd, *J* = 1.7, 0.8 Hz, 0.5H), 7.60 – 7.58 (m, 1H), 7.29 – 7.27 (m, 4H), 7.25 – 7.21 (m, 1H), 6.76 (d, *J* = 8.4 Hz, 0.64H), 6.73 (d, *J* = 8.4 Hz, 0.46H), 5.28 (dd, *J* = 8.8, 3.7 Hz, 0.47H), 5.24 (dd, *J* = 8.7, 4.1 Hz, 0.61H), 3.68 – 3.48 (m, 2H), 3.04 (q, *J* = 1.6 Hz, 1.78H), 2.94 – 2.93 (m, 1.2H), 2.23 – 2.18 (m, 1H), 2.18 (s, 2H), 2.17 (s, 1H), 2.15 – 2.05 (m, 1H), 1.24 (s, 9H), 1.23 – 1.22 (m, 9H) ppm.

^{13}C NMR (151 MHz, CD_3CN, *mixture of rotamers*) δ 187.0, 169.0, 169.0, 168.3, 168.3, 168.3, 157.5, 157.3, 157.2 (q, J = 35.0 Hz), 157.1 (q, J = 35.1 Hz), 149.2, 141.9, 141.7, 141.1, 141.0, 137.7, 137.4, 132.1, 132.0, 131.6, 129.6, 129.6, 128.9, 128.8, 126.9, 126.8, 117.7 (q, *J* = 287.2 Hz), 117.6 (q, *J* = 287.5 Hz), 117.6, 117.4, 78.0, 77.5, 62.2, 47.2, 37.4, 35.7, 35.5 (q, *J* = 3.9 Hz), 34.9 (q, *J* = 0.8 Hz), 16.7, 16.6 ppm.

^{19}F{^{1}H} NMR (565 MHz, CD_3CN, *mixture of rotamers*) δ –69.56, –70.52, –151.60, –151.65 ppm.

^{11}B NMR (193 MHz, CD_3CN) δ –1.20 ppm.

HRMS (ESI) calculated for $C_{35}H_{42}BiF_3N_3O_2$ $[M–BF_4]^+$: 802.3028, found: 802.3031.

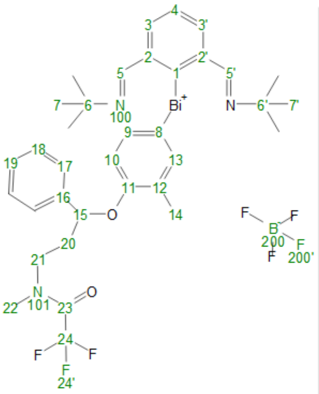

Atom	δ (ppm)	Predicted Shift	J	HSQC	COSY	HMBC	NOESY
1 C	187.021	142.68				3, 5	
2 C	149.201	139.02				3, 4, 5	
2' C	149.201	139.04				3', 4, 5'	
3 C	137.418	131.47		3		3', 4, 5	
H	8.269	7.62	7.60(4), 1.00(3')	3	4	1, 2, 3', 4, 5	4, 5
3' C	137.418	131.49		3'		3, 4, 5'	
H	8.253	7.62	1.00(3), 7.60(4)	3'	4	2', 3, 5'	
4 C	131.571	127.69		4		3	
H	8.027	7.33	7.60(3), 7.60(3')	4	3, 3'	2, 2', 3, 3'	3
5 C	168.279	156.33		5		3	
H	9.634	8.29		5		1, 2, 3, 6, 7, 100	3, 7
5' C	168.268, 168.274	156.37		5'		3'	
H	9.608	8.29		5'		2', 3', 6', 7'	
6 C	62.164	60.00				5, 7	
6' C	62.164	60.04				5', 7'	
7 C	30.945	29.25		7		5, 7	
H3	1.239, 1.239	1.22		7		6, 7, 100	5, 9
7' C	30.945	29.28		7'		5'	
H3	1.231, 1.233	1.22		7'		6'	13
8 C	168.959, 168.999	133.55				10	
9 C	137.660	131.17		9		13	
H	7.590	7.17	8.00(10)	9	10	11, 13	7, 10
10 C	117.431, 117.576	115.00		10			
H	6.733, 6.755	6.69	8.00(9)	10	9	8, 11, 12	9, 15
11 C	157.294, 157.456	155.44				9, 10, 13, 14, 15	
12 C	131.965, 132.120	131.69				10, 14	
13 C	141.036, 141.070	134.64		13		9, 14	
H	7.779, 7.786	7.21		13		9, 11, 14	7', 14

Atom	δ (ppm)	Predicted Shift	J	HSQC	COSY	HMBC	NOESY
14 C	16.617, 16.667	16.21		14		13	
H3	2.170, 2.182	2.22		14		11, 12, 13	13, 15, 17, 22
15 C	77.462, 77.957	79.61		15		17, 20', 21', 21''	
H	5.242, 5.281	5.53		15	20', 20''	11, 16, 17, 20, 21	10, 14, 17, 21', 21''
16 C	141.694, 141.941	139.30				15, 18	
17 C	126.780, 126.903	126.97		17		15, 18, 19	
H	7.280	7.38		17	18	15, 19	14, 15
18 C	129.571, 129.623	128.51		18			
H	7.281	7.38		18	17, 19	16, 17	
19 C	128.834, 128.873	129.19		19		17	
H	7.233	7.33		19	18	17	
20 C	35.733, 37.413	32.09		20', 20''		15, 21', 21''	
H'	2.195, 2.215	2.17, 2.36		20	15, 21', 21''	15, 21	
H''	2.078, 2.132	2.17, 2.36		20	15, 21', 21''	21	22
21 C	47.160	44.90		21', 21''		15, 20', 20'', 22	
H'	3.588, 3.648	3.37, 3.47		21	20', 20''	15, 20, 22, 23	15
H''	3.523, 3.549	3.37, 3.47		21	20', 20''	15, 20, 22, 23	15
22 C	34.890, 35.479	32.69	3.90(24')	22		21', 21''	
H3	2.939, 3.039	2.95		22		21, 23, 101	14, 20''
23 C	157.124, 157.226	158.75	35.10(24')			21', 21'', 22	
24 C	117.626, 117.718	115.87	287.20(24')				
24' F	-70.523, -69.558		35.10(23), 287.20(24), 3.90(22)				
100 N	-70.259					5, 7	
101 N	-271.860, -270.252					22	
200 B	-1.195						
200' F	-151.653, -151.599						

Note: The stereogenic center at C15 renders the ligand backbone unsymmetric, giving rise to two distinct sets of signals. In addition, hindered rotation around the N101–C23 bond results in two interconverting rotameric species in solution (ratio ~2:3). The observed signals are listed at their respective positions; in cases where two signals were detected, the chemical shifts of both rotameric forms are reported.

[(2,6-(^{t}BuNCH)$_2$C$_6$H$_3$)Bi(*pyriproxifen*)(BF$_4$)] (*Table S2*, ArBi(BF$_4$)-4)

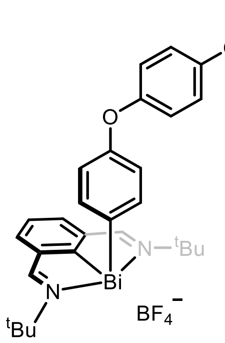

Following *General Procedure E*, the title compound **ArBi(BF$_4$)-4** was obtained from *N*,*C*,*N*-pincer bismuthinidene **Bi(I)** (9.1 mg, 0.020 mmol, 1.0 equiv.) and the corresponding thianthrenium salt **TT-8** (13.7 mg, 0.022 mmol, 1.1 equiv.) in anhydrous MeCN-*d$_3$* (1.0 mL) after 1 h of red LED irradiation (660 nm) (86% NMR yield) and used for characterization. Alternatively, the title compound was obtained from *N*,*C*,*N*-pincer bismuthinidene **Bi(I)** (22.6 mg, 0.0500 mmol, 1.00 equiv.) and the corresponding thianthrenium salt **TT-8** (31.2 mg, 0.0500 mmol, 1.00 equiv.) in anhydrous MeCN-*d$_3$* (1.0 mL) after 4 h of red LED irradiation. The NMR yield was determined using mesitylene (5.00 µL, 0.0359 mmol, 0.719 equiv.) as an internal standard (71% NMR yield). After evaporation of the solvent, the title compound was dried under high vacuum for 1 h and directly used in the ambiphilic cross-coupling without further purification. The spectral data are consistent with previously reported data within experimental error.[32]

^{1}H NMR (600 MHz, CD$_3$CN) δ 9.67 (s, 2H), 8.29 (d, *J* = 7.6 Hz, 2H), 8.12 (ddd, *J* = 5.1, 2.0, 0.9 Hz, 1H), 8.07 – 8.03 (m, 1H), 7.98 – 7.93 (m, 2H), 7.63 (ddd, *J* = 8.3, 7.1, 2.0 Hz, 1H), 6.99 – 6.96 (m, 2H), 6.92 – 6.89 (m, 3H), 6.89 – 6.85 (m, 2H), 6.70 (dt, *J* = 8.3, 0.9 Hz, 1H), 5.54 – 5.00 (m, 1H), 4.14 (dd, *J* = 10.3, 6.0 Hz, 1H), 4.08 (dd, *J* = 10.3, 4.1 Hz, 1H), 1.39 (d, *J* = 6.4 Hz, 2H), 1.30 (s, 18H) ppm.

^{13}C NMR (151 MHz, CD$_3$CN) δ 187.3, 171.6, 168.4, 164.1, 160.5, 156.6, 150.2, 149.3, 147.8, 140.9, 140.1, 137.5, 131.7, 122.1, 121.7, 117.9, 116.8, 112.2, 71.9, 70.2, 62.3, 31.0, 17.0 ppm.

^{19}F{^{1}H} NMR (565 MHz, CD$_3$CN) δ –151.65 ppm.

HRMS (ESI) calculated for C$_{36}$H$_{41}$BiN$_3$O$_3$ [M–BF$_4$]$^+$: 772.2946, found: 772.2952.

3. Optimization of Reaction Conditions.

Table S3. Ambiphilic cross-coupling of aryl-bismuth with aryl iodide

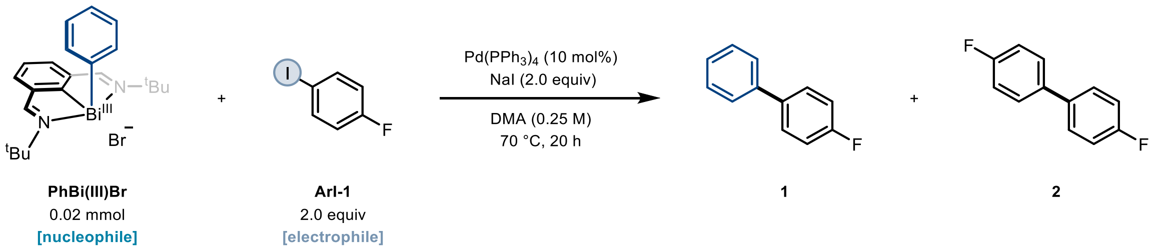

entry	cat. (mol%)	solvent (M)	additive (equiv)	temp. (°C)	time (h)	note	1 (%)[a]	2 (%)[a]
1	$Pd(PPh_3)_4$ (10)	DMA (0.05)	NaI (2.0)	70	20	-	72	24
2	Pd_2dba_3/PPh_3 (10/20)	DMA (0.05)	NaI (2.0)	70	20	-	72	21
3	$Pd(PPh_3)_4$ (10)	NMP (0.05)	NaI (2.0)	70	20	-	54	12
4	$Pd(PPh_3)_4$ (10)	DMF (0.05)	NaI (2.0)	70	20	-	71	18
5	$Pd(PPh_3)_4$ (10)	DME (0.05)	NaI (2.0)	70	20	-	41	12
6	$Pd(PPh_3)_4$ (10)	2-Me-THF (0.5)	NaI (2.0)	70	20	-	38	46
7	$Pd(PPh_3)_4$ (10)	DMA (0.05)	-	70	20	-	33	4
8	$Pd(PPh_3)_4$ (10)	DMA (0.05)	NMe_4I (2.0)	70	20	-	57	13
9	$Pd(PPh_3)_4$ (10)	DMA (0.05)	Na_2CO_3 (2.0)	70	20	-	43	10
10	$Pd(PPh_3)_4$ (10)	DMA (0.05)	K_2CO_3 (2.0)	70	20	-	51	62
11	$Pd(PPh_3)_4$ (10)	DMA (0.05)	Cs_2CO_3 (2.0)	70	20	-	67	22
12	$Pd(PPh_3)_4$ (10)	DMA (0.05)	K_3PO_4 (2.0)	70	20	-	61	15
13	$Pd(PPh_3)_4$ (10)	DMA (0.05)	NaI (2.0)	60	20	-	30	4
14	$Pd(PPh_3)_4$ (10)	DMA (0.05)	NaI (2.0)	80	20	-	72	24
15	$Pd(PPh_3)_4$ (10)	DMA (0.05)	NaI (2.0)	90	20	-	57	9
16	$Pd(PPh_3)_4$ (2.5)	DMA (0.05)	NaI (2.0)	70	20	0.05 mmol scale	38	5
17	$Pd(PPh_3)_4$ (5.0)	DMA (0.05)	NaI (2.0)	70	20	0.05 mmol scale	65	20
18	$Pd(PPh_3)_4$ (7.5)	DMA (0.05)	NaI (2.0)	70	20	0.05 mmol scale	66	21
19	$Pd(PPh_3)_4$ (10)	DMA (0.05)	NaI (2.0)	70	20	0.05 mmol scale	67	21
20	$Pd(PPh_3)_4$ (12.5)	DMA (0.05)	NaI (2.0)	70	20	0.05 mmol scale	69	22
21	$Pd(PPh_3)_4$ (15)	DMA (0.05)	NaI (2.0)	70	20	0.05 mmol scale	70	22
22	$Pd(PPh_3)_4$ (10)	DMA (0.25)	NaI (2.0)	70	20	0.05 mmol scale	76	24
23	$Pd(PPh_3)_4$ (10)	DMA (0.25)	NaI (2.0)	70	20	0.10 mmol scale	79	26
24	$Pd(PPh_3)_4$ (10)	DMA (0.125)	NaI (2.0)	70	20	0.05 mmol scale	75	24
25	$Pd(PPh_3)_4$ (10)	DMA (0.083)	NaI (2.0)	70	20	0.05 mmol scale	72	24
26	$Pd(PPh_3)_4$ (10)	DMA (0.0625)	NaI (2.0)	70	20	0.05 mmol scale	68	22
27	$Pd(PPh_3)_4$ (10)	DMA (0.0416)	NaI (2.0)	70	20	0.05 mmol scale	64	20
28	$Pd(PPh_3)_4$ (10)	DMA (0.033)	NaI (2.0)	70	20	0.05 mmol scale	64	20

[a]Yields were determined by ^{19}F NMR analysis using trifluorotoluene as an internal standard.

*Note: Under typical reaction conditions, the predominant species observed after the reaction are the cross-coupling product **1**, the homocoupling product **2**, and the residual starting material **ArI-1**.*

Figure S1. Evaluation of other electrophilic partners

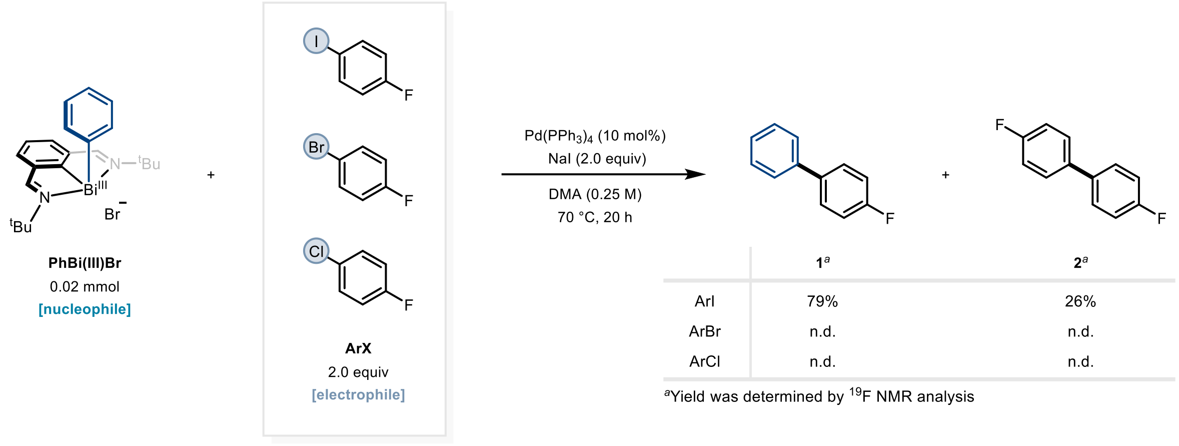

	1[a]	2[a]
ArI	79%	26%
ArBr	n.d.	n.d.
ArCl	n.d.	n.d.

[a]Yield was determined by ^{19}F NMR analysis

Note: Yields were determined by ^{19}F NMR analysis using trifluorotoluene as an internal standard. Reactions with other aryl electrophiles (aryl bromides, aryl chlorides, and aryl triflates), rather than aryl iodides, did not afford any products, highlighting the chemoselectivity observed with dihaloarenes and 4-iodophenyl trifluoromethanesulfonate (see Figure 3, compounds 11–13).

Figure S2. Evaluation of the counteranion in ambiphiles acting as nucleophiles

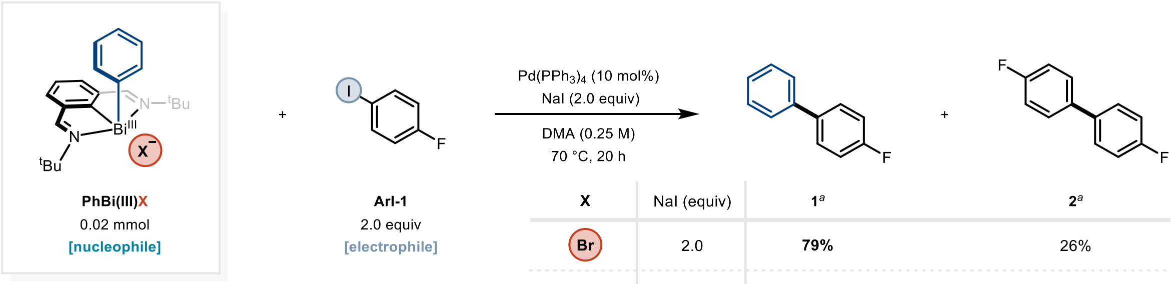

X	NaI (equiv)	1[a]	2[a]
Br	2.0	**79%**	26%
BF_4	2.0	42%	13%
	5.0	58%	17%
	10	**63%**	19%
	20	64%	19%

[a]Yield was determined by ^{19}F NMR analysis

Note: When aryl-bismuth species bearing weakly coordinating anions such as BF_4 and OTf were used as nucleophilic partners, the reactivity was diminished compared to aryl-bismuth species bearing bromide as the counteranion. Further optimization showed that the addition of 10 equivalents of NaI restored the reactivity to a reasonable level. Accordingly, when aryl-bismuth (BF_4 or OTf) was employed as the nucleophile, 10 equivalents of NaI were added (see Figure 3C, compounds 36–41).

Figure S3. Control experiments on the origin of aryl iodide-derived homocoupling products

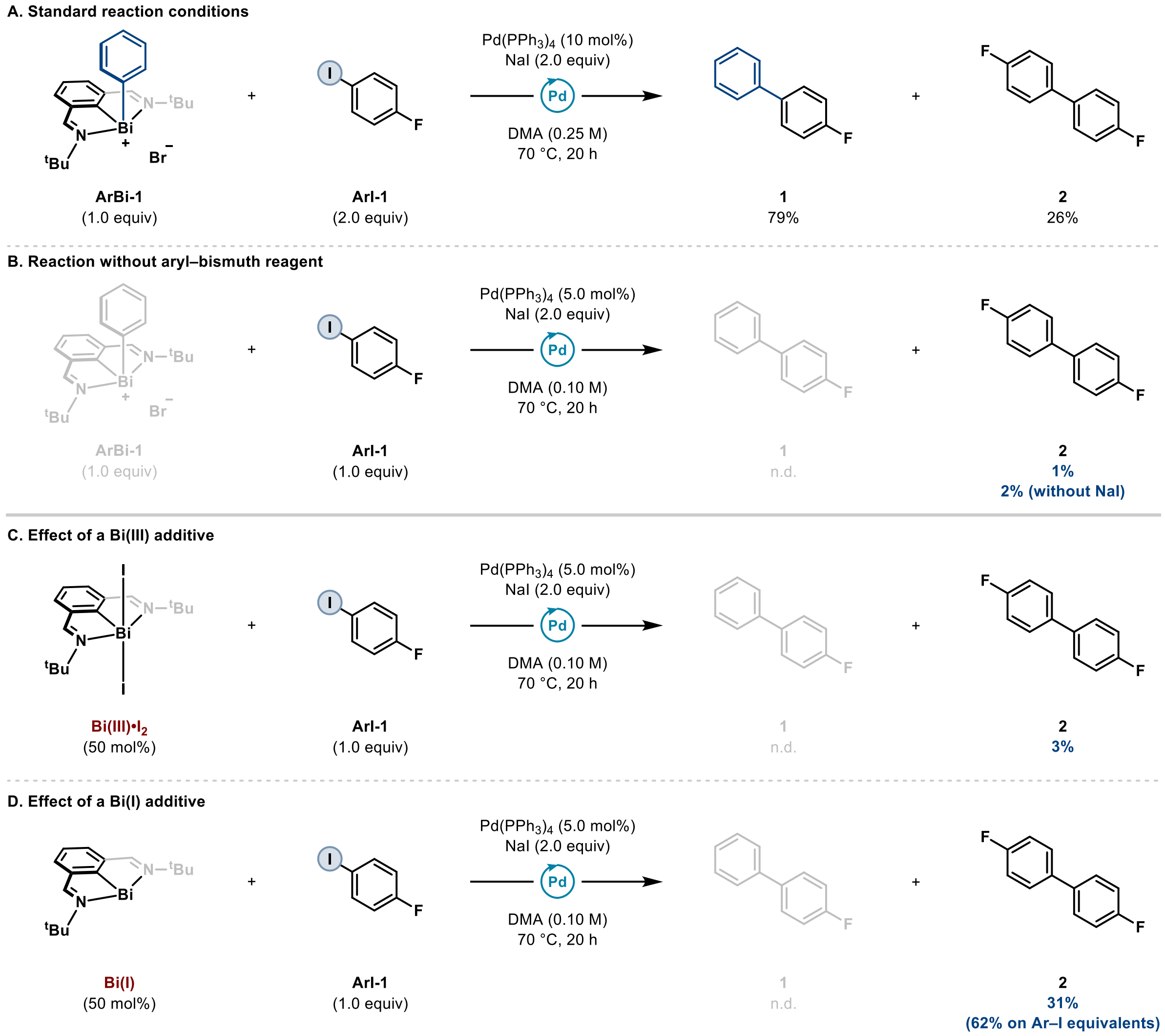

Note: The results of the control experiments suggest that Bi(I) can act as a reductant, thereby promoting palladium-catalyzed reductive homocoupling of the aryl iodide.

Figure S4. Additive effect

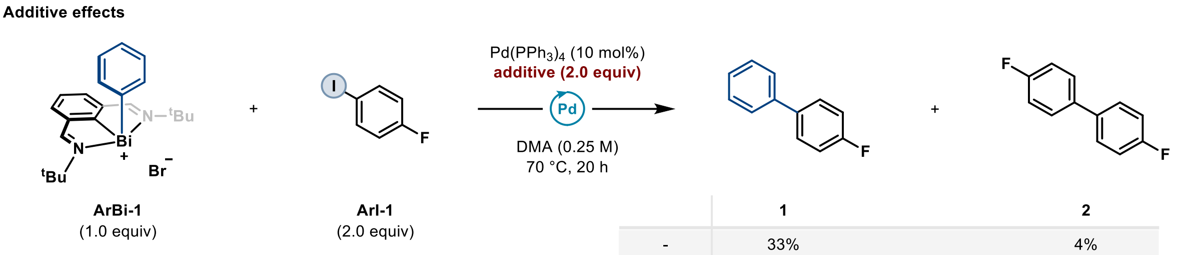

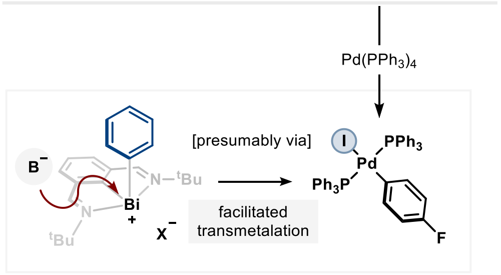

	1	2
-	33%	4%
Na_2CO_3	43%	10%
K_2CO_3	51%	62%
NMe_4I	57%	13%
K_3PO_4	61%	15%
Cs_2CO_3	67%	22%
NaI	72%	24%

Note: Although the precise influence of individual additives on cross-/homocoupling selectivity remains unclear, their overall effect is proposed to arise from activation of the aryl-bismuth reagent, analogous to base-assisted activation of organoboron species in Suzuki coupling.

Figure S5. Observation of aryl bismuth-derived coupling products

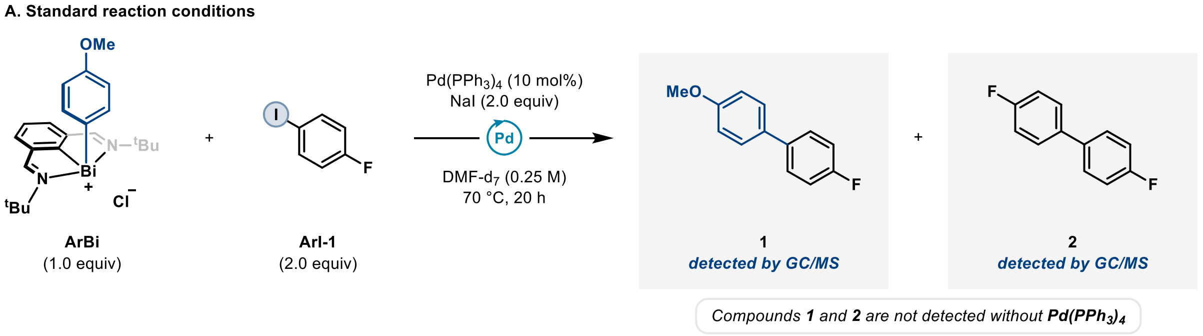

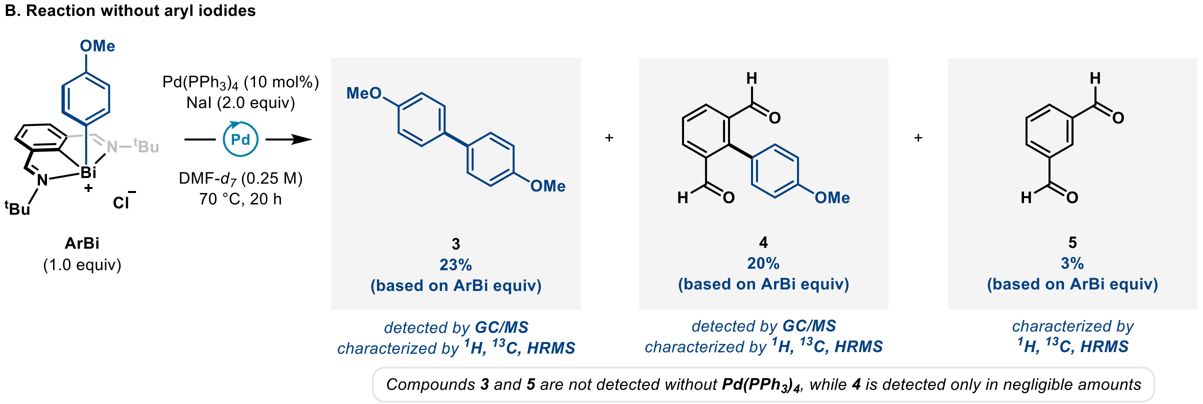

Note: *These observations indicate that aryl-bismuth species can engage in ambiphilic coupling behavior, acting as both a nucleophilic aryl donor and an electrophilic coupling partner. Consistent with this interpretation, aryl bismuth-derived coupling products (**3** and **4**) are observed in the absence of aryl iodide, supporting the possibility of off-cycle reactivity (see Figure S15).*

4,4'-dimethoxy-1,1'-biphenyl (Figure S4)

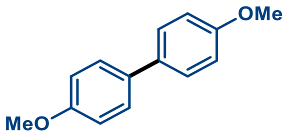

Following *General Procedure F* in the absence of aryl iodide on a 0.100 mmol scale, the crude product was purified by pTLC (10% EtOAc in pentane) to afford the title compound as a white solid (2.5 mg, 23% on ArBi equivalents). The spectral data are consistent with previously reported data within experimental error.[48]

^{1}H NMR (600 MHz, $CDCl_3$) δ 7.50 – 7.46 (m, 4H), 6.98 – 6.94 (m, 4H), 3.84 (s, 6H) ppm.
^{13}C NMR (151 MHz, $CDCl_3$) δ 158.84, 133.64, 127.89, 114.31, 55.50 ppm.
HRMS (EI) calculated for $C_{14}H_{14}O_2$ $[M]^+$: 214.0991, found: 214.0988.

4'-methoxy-[1,1'-biphenyl]-2,6-dicarbaldehyde (Figure S4)

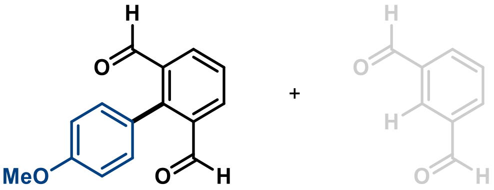

Following *General Procedure F* in the absence of aryl iodide on a 0.100 mmol scale, the crude product was purified by pTLC (10% EtOAc in pentane) to afford the title compound as an orange solid in mixture with the debismuthation side product (2.85 mg, 23% combined yield based on ArBi).

^{1}H NMR (600 MHz, $CDCl_3$) δ 9.85 (d, J = 0.8 Hz, 2H), 8.23 (d, J = 7.7 Hz, 2H), 7.64 (tt, J = 7.7, 0.9 Hz, 1H), 7.30 – 7.27 (m, 2H), 7.06 – 7.03 (m, 2H), 3.90 (s, 3H) ppm.
^{13}C NMR (151 MHz, $CDCl_3$, *mixture with debismuthation side product*) δ 191.41, 191.17, 160.38, 148.14, 137.18, 135.23, 134.78, 132.77, 132.36, 131.18, 130.08, 128.30, 124.39, 114.16, 55.61 ppm.
HRMS (EI) calculated for $C_{15}H_{12}O_3$ $[M]^+$: 240.0781, found: 240.0783.

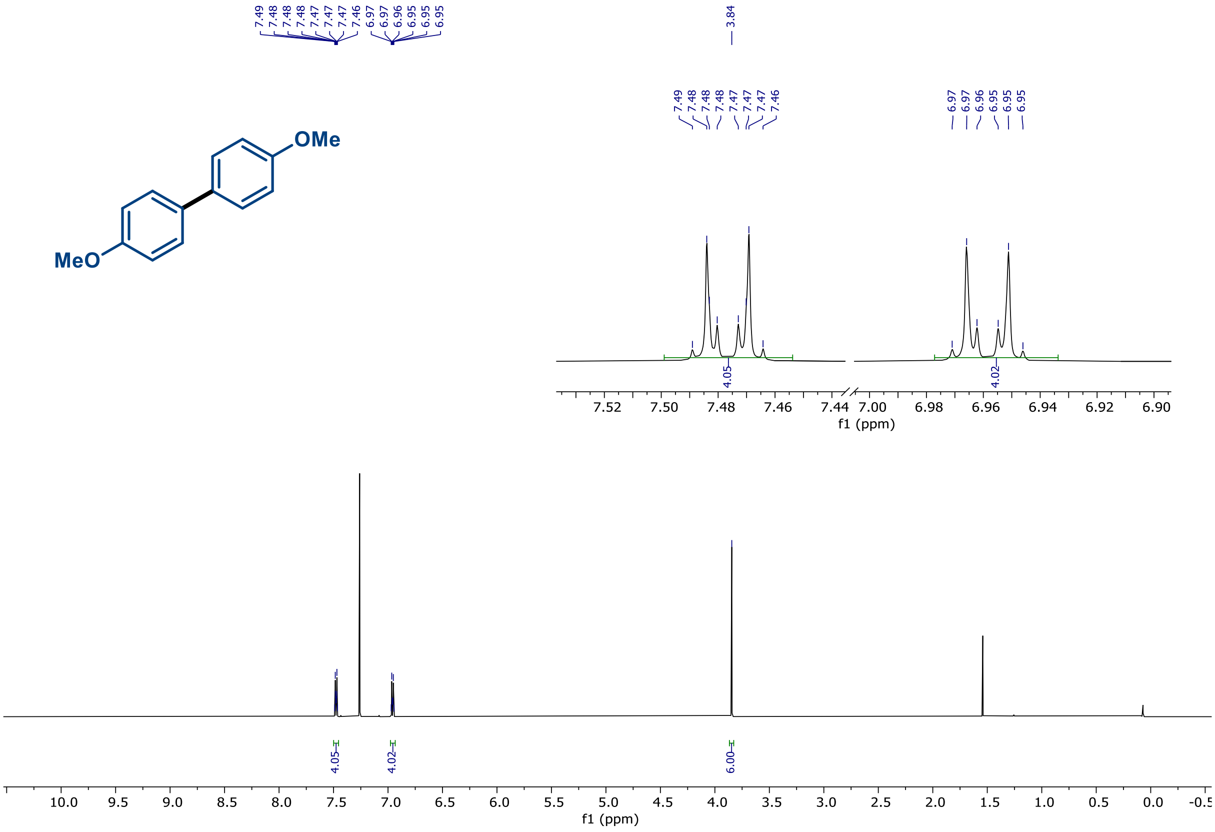
7.49
7.48
7.48
7.48
7.47
7.47
7.47
7.46
6.97
6.97
6.96
6.95
6.95
6.95
3.84
OMe
MeO
4.05
4.02
6.00
f1 (ppm)

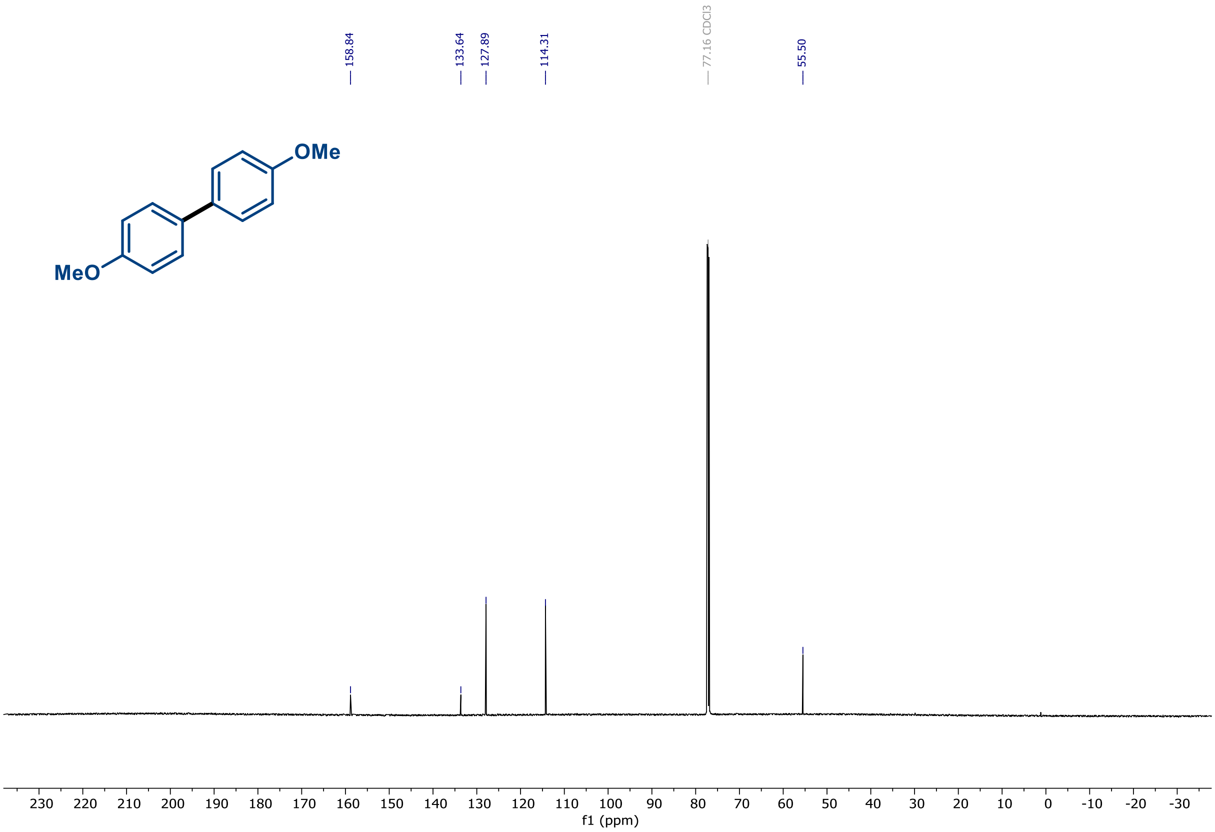
158.84
133.64
127.89
114.31
77.16 CDCl3
55.50
OMe
MeO
f1 (ppm)

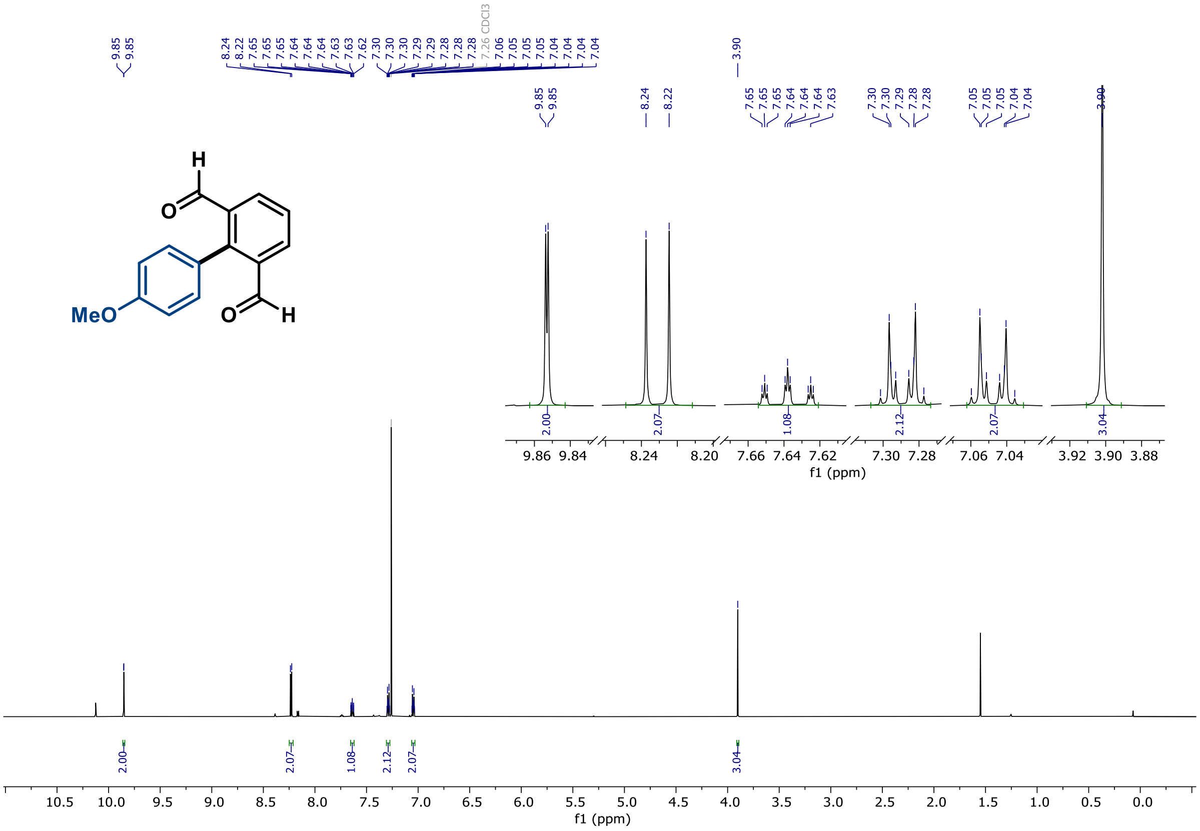
9.85
9.85
8.24
8.22
7.65
7.65
7.65
7.64
7.64
7.64
7.63
7.63
7.62
7.30
7.30
7.30
7.29
7.29
7.28
7.28
7.28
7.26 CDCl3
7.06
7.05
7.05
7.05
7.04
7.04
7.04
7.04
3.90
H
O
MeO
O
H
f1 (ppm)

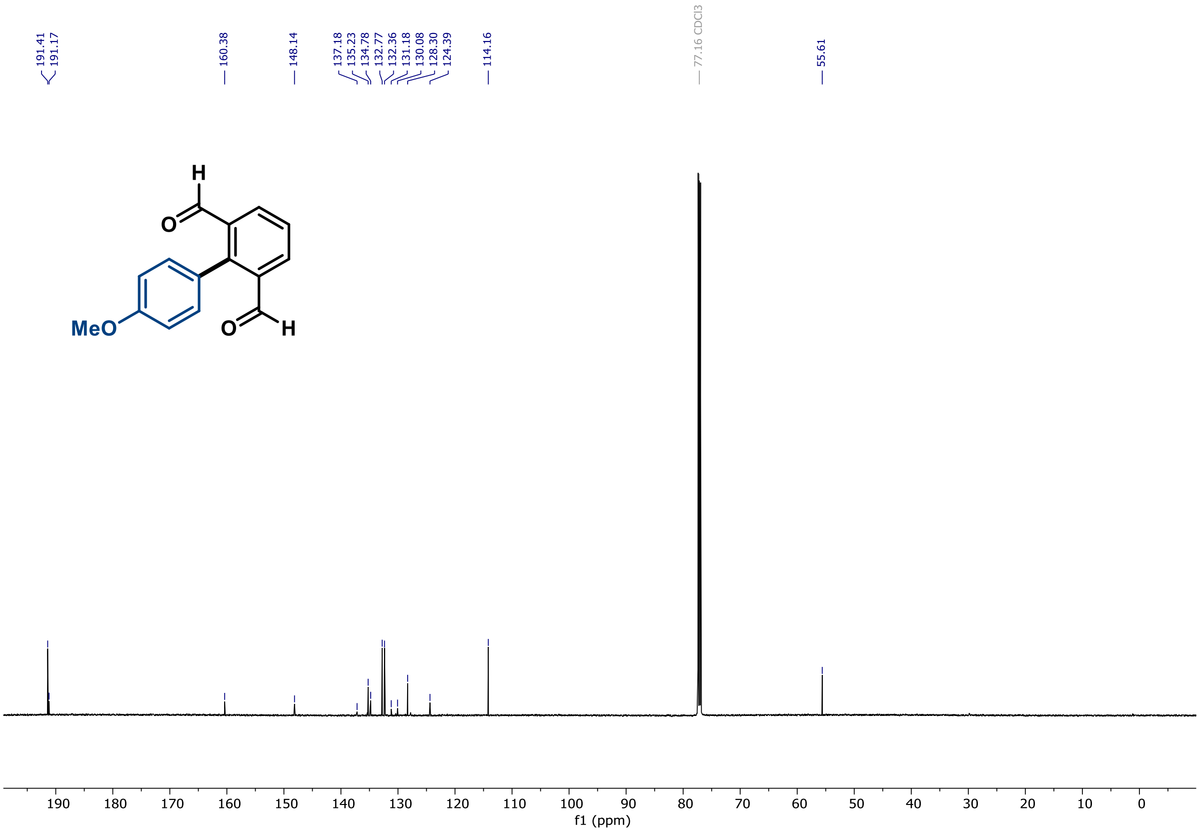
191.41
191.17
160.38
148.14
137.18
135.23
134.78
132.77
132.36
131.18
130.08
128.30
124.39
114.16
77.16 CDCl3
55.61
H
O
MeO
O
H
f1 (ppm)

Figure S6. Systematic analysis of side product profiles across representative substrates

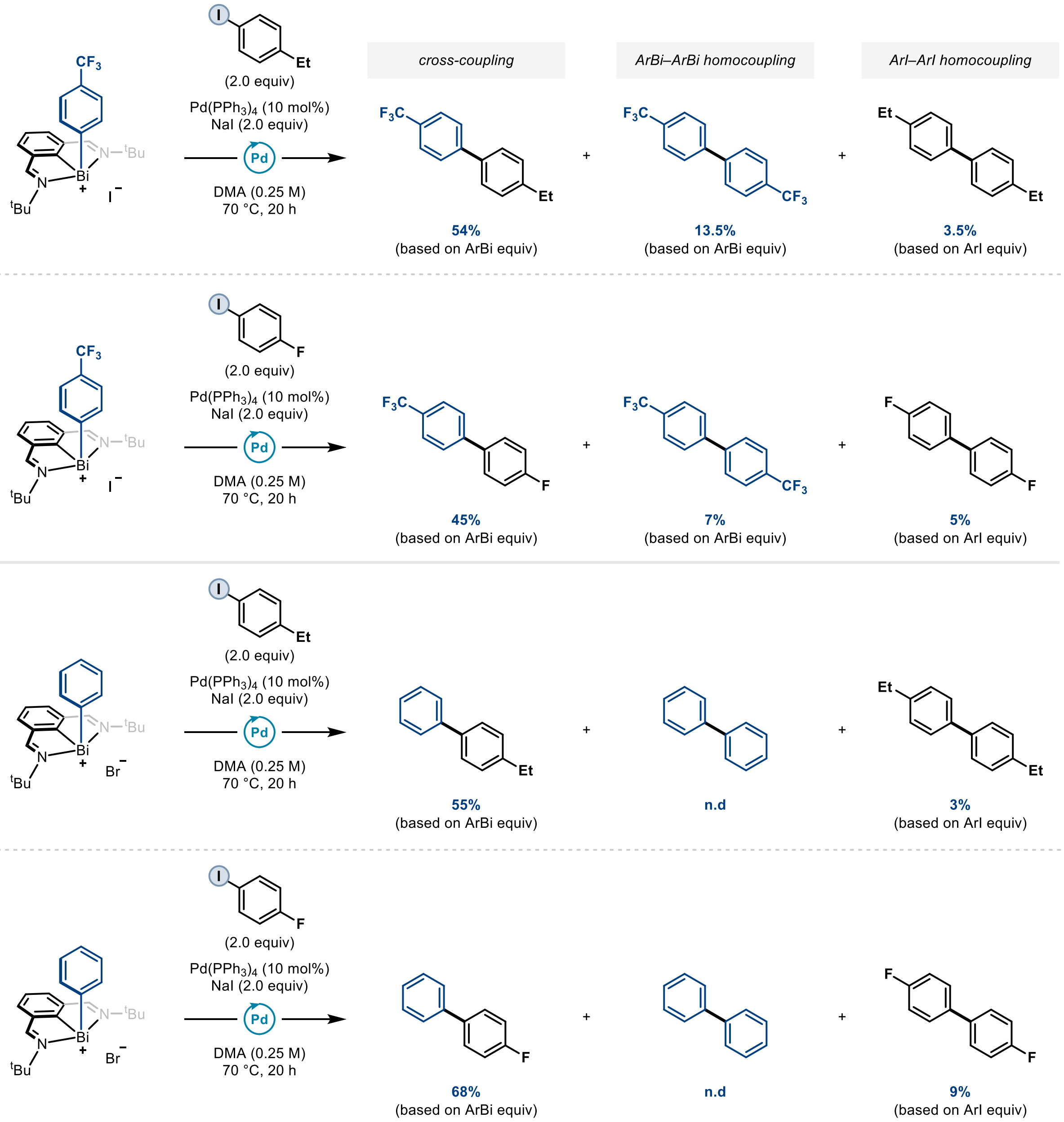

Note: In cross-couplings of aryl-bismuth reagents with aryl electrophiles, electrophile-derived homocoupling was occasionally observed (up to ~15%). In cases where the homocoupled side product exhibits similar polarity to the desired product, chromatographic separation can be challenging.

Table S4. Ambiphilic cross-coupling of aryl-bismuth with aryl-boron derivative

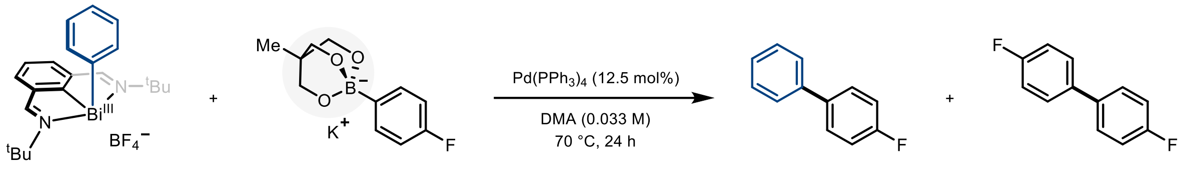

PhBi(III)BF$_4$ 0.02 mmol [electrophile] + **ArB-1** 2.0 equiv [nucleophile] → **1** + **2**

entry	cat. (mol%)	ligand (mol%)	solvent (M)	light	temp. (°C)	time (h)	note	**1** (%)[a]	**2** (%)[a]
1	Pd(PPh$_3$)$_4$ (10)	BrettPhos (20)	DMA (0.05)	456 nm	35~40	24	-	59	<1
2	Pd(PPh$_3$)$_4$ (10)	BrettPhos (20)	DMA (0.05)	-	25	24	-	35	<1
3	Pd(PPh$_3$)$_4$ (10)	BrettPhos (20)	DMA (0.05)	550 nm	35~40	24	-	46	<1
4	Pd(PPh$_3$)$_4$ (10)	BrettPhos (20)	DMA (0.05)	456 nm	35~40	24	**ArB-1** (1.1 equiv)	54	<1
5	Pd(PPh$_3$)$_4$ (10)	BrettPhos (20)	DMA (0.05)	456 nm	35~40	24	**ArB-1** (1.5 equiv)	58	<1
6	Pd(PPh$_3$)$_4$ (10)	-	DMA (0.05)	456 nm	35~40	24	-	60	<1
7	Pd(PPh$_3$)$_4$ (10)	-	DMA (**0.02**)	456 nm	35~40	24	-	62	<1
8	Pd(PPh$_3$)$_4$ (10)	-	DMA (**0.04**)	456 nm	35~40	24	-	45	<1
9	Pd(PPh$_3$)$_4$ (**10**)	-	DMA (0.05)	456 nm	35~40	24	0.05 mmol scale	67	<1
10	Pd(PPh$_3$)$_4$ (**12.5**)	-	DMA (0.05)	456 nm	35~40	24	0.05 mmol scale	74	<1
11	Pd(PPh$_3$)$_4$ (**15**)	-	DMA (0.05)	456 nm	35~40	24	0.05 mmol scale	75	<1
12	Pd(PPh$_3$)$_4$ (10)	-	DMA (**0.042**)	456 nm	35~40	24	0.05 mmol scale	72	<1
13	Pd(PPh$_3$)$_4$ (10)	-	DMA (**0.033**)	456 nm	35~40	24	0.05 mmol scale	75	<1
14	Pd(PPh$_3$)$_4$ (10)	-	DMA (**0.025**)	456 nm	35~40	24	0.05 mmol scale	74	<1
15	Pd(PPh$_3$)$_4$ (12.5)	-	DMA (**0.033**)	456 nm	35~40	24	0.05 mmol scale	75	<1
16	Pd(PPh$_3$)$_4$ (12.5)	-	DMA (**0.033**)	456 nm	35~40	24	0.10 mmol scale	78	<1
17	Pd(PPh$_3$)$_4$ (12.5)	-	DMA (0.033)	-	70	24	ArB(OH)$_2$ *instead of* **ArB-1**	<1	<1
18	Pd(PPh$_3$)$_4$ (12.5)	-	DMA (0.033)	-	70	24	ArBpin *instead of* **ArB-1**	<1	<1
19	Pd(PPh$_3$)$_4$ (12.5)	-	DMA (0.033)	-	70	24	ArB(neo) *instead of* **ArB-1**	<1	<1
20	Pd(PPh$_3$)$_4$ (12.5)	-	DMA (0.033)	-	70	24	Boroxine *instead of* **ArB-1**	<1	<1
21	Pd(PPh$_3$)$_4$ (12.5)	-	DMA (0.033)	-	**25**	24	-	49	<1
22	Pd(PPh$_3$)$_4$ (12.5)	-	DMA (0.033)	-	**50**	24	-	69	<1
23	-	-	DMA (0.033)	-	**70**	24	-	<1	<1
24	Pd(PPh$_3$)$_4$ (12.5)	-	DMA (0.033)	-	**70**	24	-	87	<1
25	Pd(PPh$_3$)$_4$ (12.5)	-	DMA (0.033)	-	**90**	24	-	86	<1
26	Pd(PPh$_3$)$_4$ (12.5)	-	DMA (0.033)	-	**110**	24	-	79	<1

[a]Yields were determined by ^{19}F NMR analysis using trifluorotoluene as an internal standard.

*Note: Under typical reaction conditions, the predominant species observed after the reaction is the cross-coupling product **1**, while in contrast to the previous case (cross-coupling with aryl iodides), no homocoupling product is observed. Furthermore, light irradiation, which was employed during early optimization studies, was found to exert only a thermal (heating) effect rather than promoting a distinct photochemical pathway.*

Figure S7. Evaluation of the counteranion in ambiphiles via anion exchange

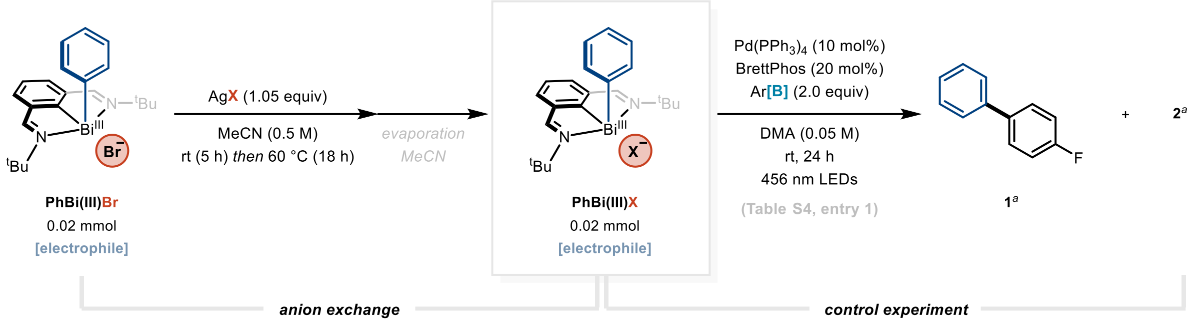

		1[a]	2[a]
**PhBi(III)Br** + *additive* (insitu)	-	22%	<1%
	w/ $NaBF_4$	19%	<1%
	w/ $AgBF_4$	25%	8%
*with an **anion exchange** step*	w/ $AgBF_4$	50%	4%
	w/ $AgPF_4$	42%	1%
	w/ $AgSbF_4$	42%	2%
	w/ AgOTf	45%	4%

[a]Yield was determined by ^{19}F NMR analysis

Note: Compared to the cross-coupling of aryl-bismuth (as a nucleophile) with aryl iodides, the reactions with aryl boron derivatives exhibited higher efficiency when aryl-bismuth species bearing weakly coordinating anions such as BF_4^- and OTf^- were employed. Indeed, anion-exchange of aryl-bismuth bromide with the corresponding silver salts improved the reactivity, whereas the direct addition of silver salts as additives did not have a beneficial effect. Thus, for ambiphilic coupling of aryl-bismuth as an electrophile, aryl-bismuth (BF_4^- or OTf^-) can also be prepared from the corresponding aryl-bismuth (Br) via anion-exchange (see General Procedure D).

Figure S8. Evaluation of the counteranion in ambiphiles acting as electrophiles

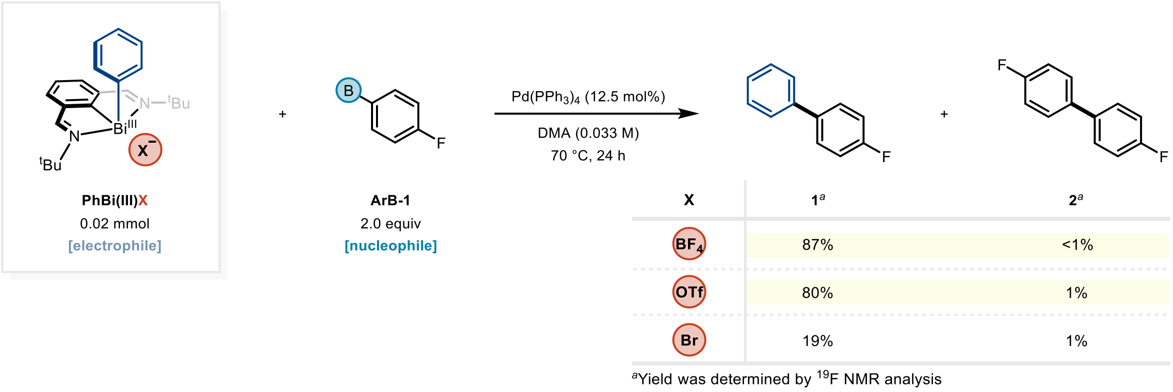

X	1[a]	2[a]
BF_4	87%	<1%
OTf	80%	1%
Br	19%	1%

[a]Yield was determined by ^{19}F NMR analysis

Note: As mentioned in Figure S3 above, aryl-bismuth complexes (BF_4^- or OTf^-) were more competent electrophilic partners for cross-coupling with arylboron derivatives.

Figure S9. Preliminary ambiphilic reactivity of other aryl-bismuth compounds

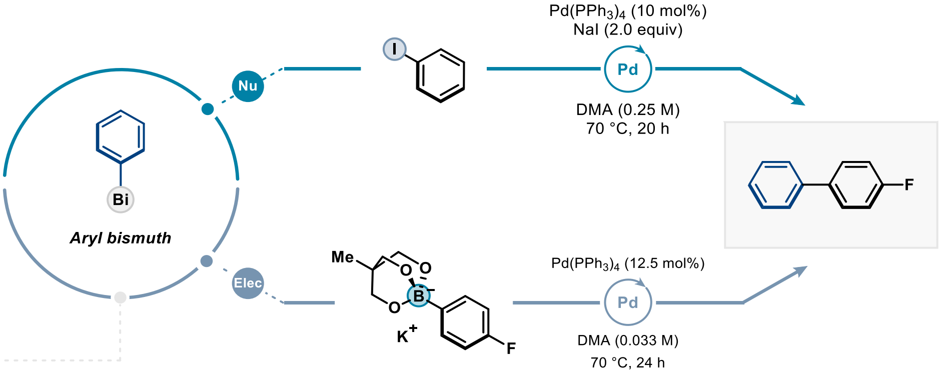

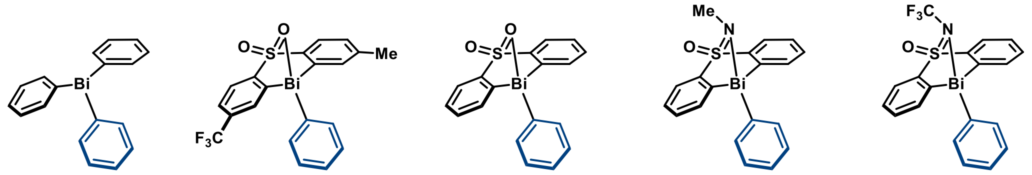

	$BiPh_3$	ArBi-A	ArBi-B	ArBi-C	ArBi-D
Nu	60	23	27	61	32
Elec	<1	<1	<1	<1	<1

[a]Yield was determined by ^{19}F NMR analysis

Note: Cross-coupling reactions of other aryl-bismuth compounds employed as nucleophiles, including triarylbismuth and aryl-bismuth species bearing a sulfonyl backbone, with aryl iodides furnished the corresponding cross-coupled products. In contrast, when aryl-bismuth compounds were used as electrophilic partners with aryl boron derivatives, no cross-coupled products were obtained.

Table S5. Current limitations in the substrate scope of ambiphilic cross-coupling

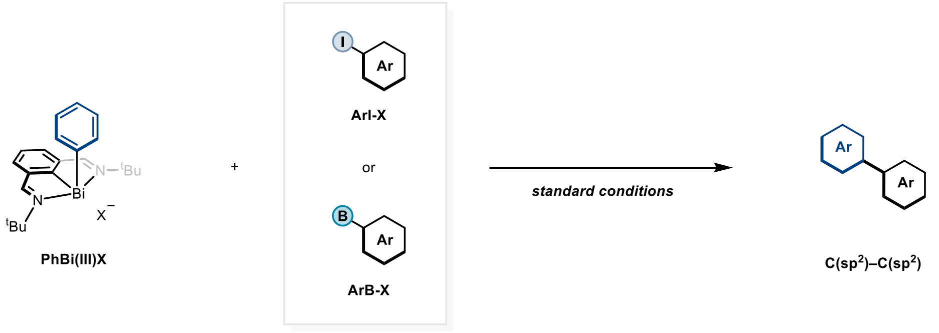

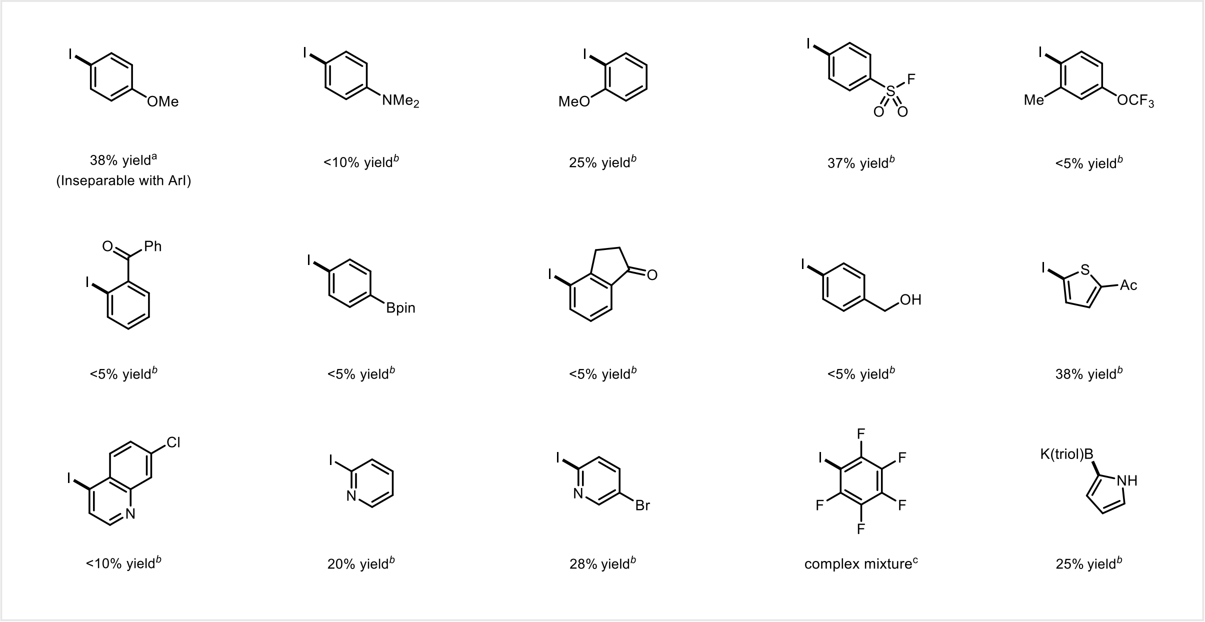

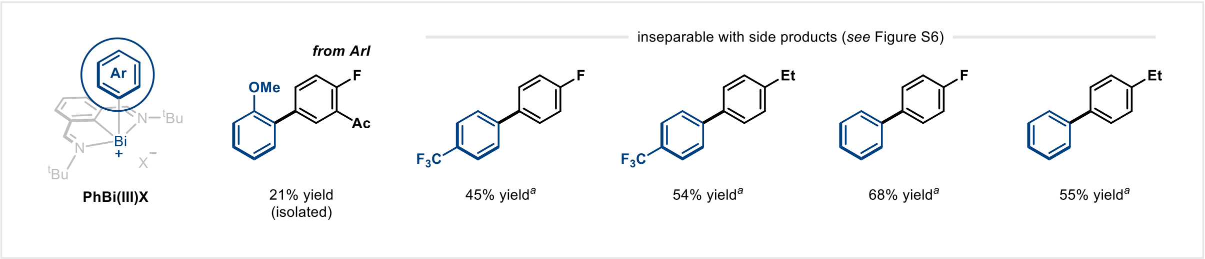

[a]*Isolated yield of the mixture.*
[b]*Yields determined by* ^{1}*H NMR analysis using dibromomethane as an internal standard*
[c]*Yields determined by* ^{19}*F NMR analysis using trifluorotoluene as an internal standard.*

Note: In the crude reaction mixture obtained after cross-coupling with aryl iodides, the major components were the cross-coupling products, the homocoupling products derived from aryl iodides, and the residual aryl iodide starting material. In contrast, the crude mixture from cross-coupling with aryl boron reagents predominantly afforded the desired cross-coupling product. In both cases, a characteristic bismuth-ligand coupling side product—arising from C–C coupling between the 1,3-di-imine aryl ligand and the coupling partner, followed by hydrolysis to the corresponding 1,3-formyl derivative—was occasionally observed (see Figure S5 and S6).

Practical summary for reaction use

A. When aryl-bismuth reagents act as nucleophiles

a) Halide counteranions (I, Br, Cl) provide higher reactivity than non-coordinating anions.
b) For non-coordinating anions (BF_4^-, OTf^-), the addition of NaI (10 equiv) improved efficiency.
c) Homocoupling products derived from aryl iodides are commonly observed.
d) Homocoupling products derived from aryl-bismuth reagents are occasionally observed.
e) Electron-rich aryl-bismuth reagents (e.g., *p*-OMe) show higher reactivity.
f) Electron-deficient aryl–bismuth reagents lead to increased homocoupling and reduced overall efficiency.
g) *Ortho*-substitution on either aryl-bismuth or aryl-iodide decreases reactivity.
h) Electron-rich electrophiles (e.g., *p*-OMe, *p*-NMe_2) show low reactivity.
i) Electron-neutral aryl bromides, chlorides, and triflates are unreactive.
j) Electron-deficient heteroaryl bromides and triflates give low yields (~5–10%) under standard conditions.
k) Electron-deficient heteroaryl bromides, triflates, and thianthrenium salts can afford moderate yields with bulky, electron-rich ligands (e.g. $PAd_2{}^n Bu$).

B. When aryl-bismuth reagents act as electrophiles

a) Non-coordinating anions (BF_4^-, OTf^-) provide higher reactivity.
b) Homocoupling products derived from aryl-boron reagents are generally not observed.
c) Homocoupling products derived from aryl-bismuth reagents are occasionally observed.
d) Electron-deficient aryl-bismuth reagents show higher reactivity.
e) Electron-rich aryl-boron reagents show higher reactivity.
f) Commercial aryl boronic acids and pinacol esters require the addition of K_3PO_4 or Cs_2CO_3 (2.0 equiv) to obtain moderate yields of cross-coupling.

4. General Procedures for Ambiphilic Cross-Coupling

4.1. Ambiphilic cross-coupling of aryl-bismuths with aryl iodides (General Procedure F)

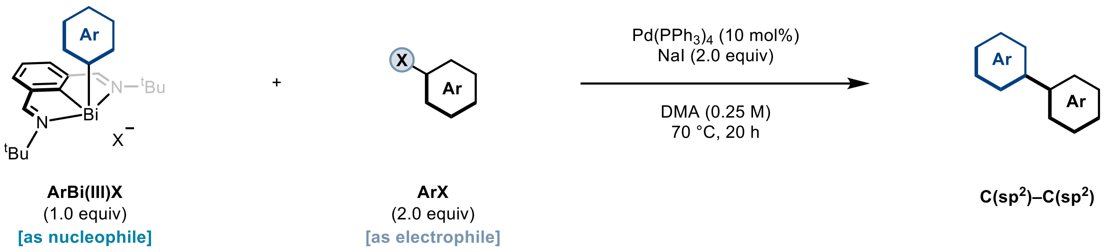

In an argon-filled glovebox, an oven-dried (80 °C, overnight) 10 mL thick-walled culture tube equipped with a Teflon-coated magnetic stir bar was charged with aryl-bismuth reagent **ArBi(III)X** (0.10 mmol), aryl electrophile (0.20 mmol, 2.0 equiv.), $Pd(PPh_3)_4$ (0.0100 mmol, 10.0 mol%, 11.5 mg), and NaI (0.20 mmol, 2.0 equiv., 30 mg). Anhydrous dimethylacetamide (DMA, 0.40 mL) was then added. If the aryl iodide was liquid, it was introduced after the addition of the solvent. The reaction tube was sealed with a screw cap and removed from the glovebox. The reaction mixture was stirred at 70 °C in a preheated heating block for 20 h. Upon completion, the reaction was quenched with brine and extracted with ethyl acetate (three times). The combined organic extracts were dried over Na_2SO_4, filtered, and concentrated under reduced pressure. The crude residue was purified by preparative thin-layer chromatography (pTLC) on silica gel plates (0.5–1.0 mm) using the appropriate eluent system to afford the desired products (compounds **3**–**18**, **36**–**41**, **48**–**50**, **59**–**61**).

*Note: Compounds **3–18**, **37**, **38**, and **59–61** were prepared using aryl-bismuth (Br); compounds **36** and **39** using aryl-bismuth (I); and compounds **40**, **41**, **48–50** using aryl-bismuth (BF_4) with NaI (10 equiv.).*

4.2. Ambiphilic cross-coupling of aryl-bismuths with aryl-boron derivatives (General Procedure G)

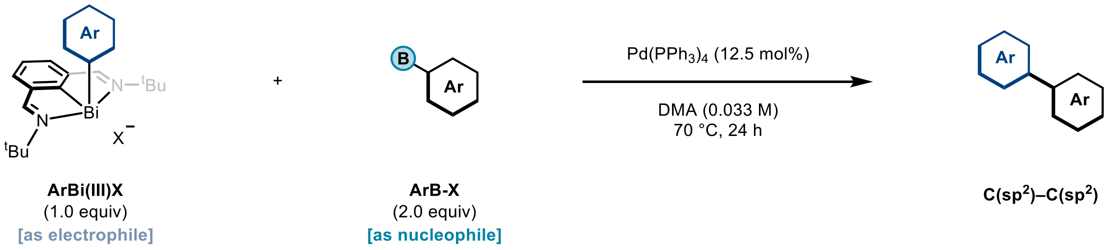

In an argon-filled glovebox, an oven-dried (80 °C, overnight) 10 mL thick-walled culture tube equipped with a Teflon-coated magnetic stir bar was charged with aryl-bismuth reagent **ArBi(III)X** (0.10 mmol), aryl boron reagent (0.20 mmol, 2.0 equiv.), and $Pd(PPh_3)_4$ (0.0125 mmol, 12.5 mol%, 14.4 mg). Anhydrous dimethylacetamide (DMA, 3.0 mL) was then added. The reaction tube was sealed with a screw cap and removed from the glovebox. The reaction mixture was stirred at 70 °C in a preheated heating block for 24 h. Upon completion, the reaction was quenched with brine and extracted with ethyl acetate (three times). The combined organic extracts were dried over Na_2SO_4, filtered, and concentrated under reduced pressure. The crude residue was purified by preparative thin-layer chromatography (pTLC) on silica gel plates (0.5–1.0 mm) using the appropriate eluent system to afford the desired products (compounds **19**–**35**, **42**–**47**, **53**–**58**).

*Note: Compounds **19–35** and **47**, **53–58** were prepared using aryl-bismuth (BF_4), and compounds **42–46** using aryl-bismuth (OTf).*

5. Synthesis and Characterization of the Products

5.1. Ambiphilic cross-coupling of aryl-bismuths with aryl iodides

4-Fluoro-4'-methoxy-1,1'-biphenyl (Figure 3, compound 3)

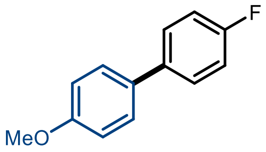

Following *General Procedure F* on a 0.100 mmol scale, the crude product was purified by pTLC (10% EtOAc in cyclohexane) to afford the title compound **3** as a white solid (17.6 mg, 87%). The spectral data are consistent with previously reported data within experimental error.[49]

^{1}H NMR (400 MHz, $CDCl_3$) δ 7.55 – 7.43 (m, 4H), 7.16 – 7.05 (m, 2H), 7.02 – 6.94 (m, 2H), 3.86 (s, 3H) ppm.
^{13}C NMR (101 MHz, $CDCl_3$) 162.2 (d, *J* = 245.5 Hz), 159.3, 137.1, 137.1, 133.0, 128.4 (d, *J* = 7.8 Hz), 128.2, 115.7 (d, *J* = 21.6 Hz), 114.4, 55.5 ppm.
^{19}F{^{1}H} NMR (282 MHz, $CDCl_3$) δ –116.79 ppm.
HRMS (EI) calculated for $C_{13}H_{11}OF$ $[M]^+$: 202.0788, found: 202.0791.

4-Nitro-1,1'-biphenyl (Figure 3, compound 4)

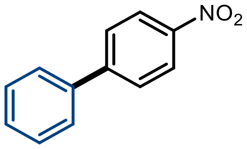

Following *General Procedure F* on a 0.100 mmol scale, the crude product was purified by pTLC (5% EtOAc in cyclohexane) to afford the title compound **4** (16.6 mg, 83%) as a pale yellow solid. The spectral data are consistent with previously reported data within experimental error.[50]

^{1}H NMR (400 MHz, $CDCl_3$) δ 8.34 – 8.27 (m, 2H), 7.77 – 7.70 (m, 2H), 7.65 – 7.60 (m, 2H), 7.53 – 7.47 (m, 2H), 7.47 – 7.42 (m, 1H) ppm.
^{13}C NMR (101 MHz, $CDCl_3$) δ 147.8, 147.3, 138.9, 129.3, 129.1, 128.0, 127.5, 124.3 ppm.
HRMS (EI) calculated for $C_{12}H_9NO_2$ $[M]^+$: 199.0628, found: 199.0630.

4-(Methylsulfonyl)-1,1'-biphenyl (Figure 3, compound 5)

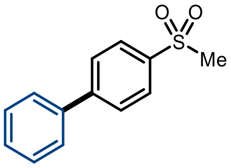

Following *General Procedure F* on a 0.100 mmol scale, the crude product was purified by pTLC (25% EtOAc in pentane) to afford the title compound **5** as a white solid (11.7 mg, 50%).The spectral data are consistent with previously reported data within experimental error.[51]

^{1}H NMR (600 MHz, $CDCl_3$) δ 8.03 – 8.00 (m, 2H), 7.79 – 7.76 (m, 2H), 7.63 – 7.60 (m, 2H), 7.51 – 7.47 (m, 2H), 7.46 – 7.42 (m, 1H), 3.10 (s, 3H) ppm.
^{13}C NMR (151 MHz, $CDCl_3$) δ 146.9, 139.3, 139.2, 129.3, 128.8, 128.2, 128.1, 127.6, 44.8 ppm.
HRMS (EI) calculated for $C_{13}H_{12}O_2S$ $[M]^+$: 232.0553, found: 232.0556.

[1,1'-Biphenyl]-3-carbonitrile (Figure 3, compound 6)

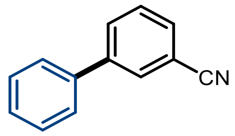

Following *General Procedure F* on a 0.100 mmol scale, the crude product was purified by pTLC (5% EtOAc in cyclohexane) to afford the title compound **6** as a colorless oil (15.8 mg, 88%). The spectral data are consistent with previously reported data within experimental error.[52]

^{1}H NMR (400 MHz, $CDCl_3$) δ 7.88 – 7.86 (m, 1H), 7.82 (ddd, *J* = 7.8, 1.9, 1.2 Hz, 1H), 7.63 (dt, *J* = 7.7, 1.4 Hz, 1H), 7.58 – 7.52 (m, 3H), 7.51 – 7.46 (m, 2H), 7.45 – 7.39 (m, 1H) ppm.
^{13}C NMR (101 MHz, $CDCl_3$) δ 142.6, 139.0, 131.6, 130.9, 130.8, 129.7, 129.3, 128.5, 127.2, 119.0, 113.1 ppm.
HRMS (EI) calculated for $C_{13}H_9N$ $[M]^+$: 179.0730, found: 179.0730.

4'-Methoxy-[1,1'-biphenyl]-2-carbaldehyde (Figure 3, compound 7)

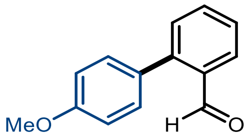

Following *General Procedure F* on a 0.100 mmol scale, the crude product was purified by pTLC (10% EtOAc in pentane) to afford the title compound **7** as a colorless oil (15.3 mg, 72%). The spectral data are consistent with previously reported data within experimental error.[53]

^{1}H NMR (400 MHz, $CDCl_3$) δ 10.00 (d, *J* = 0.9 Hz, 1H), 8.01 (ddd, *J* = 7.8, 1.5, 0.6 Hz, 1H), 7.62 (td, *J* = 7.5, 1.5 Hz, 1H), 7.51 – 7.39 (m, 2H), 7.36 – 7.26 (m, 2H), 7.05 – 6.95 (m, 2H), 3.88 (s, 3H) ppm.
^{13}C NMR (101 MHz, $CDCl_3$) δ 192.8, 159.8, 145.8, 133.9, 133.7, 131.4, 130.9, 130.2, 127.8, 127.5, 114.1, 55.5 ppm.
HRMS (EI) calculated for $C_{14}H_{12}O_2$ $[M]^+$: 212.0832, found: 212.0835.

4-Ethyl-4'-methoxy-1,1'-biphenyl (Figure 3, compound 8)

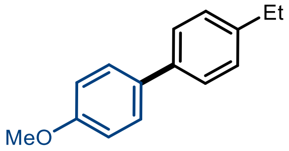

Following *General Procedure F* on a 0.100 mmol scale, the crude product was purified by pTLC (10% EtOAc in pentane) to afford the title compound **8** as a white solid (17.1 mg, 80%). The spectral data are consistent with previously reported data within experimental error.[54]

^{1}H NMR (400 MHz, $CDCl_3$) δ 7.55 – 7.45 (m, 4H), 7.28 – 7.22 (m, 2H), 7.01 – 6.95 (m, 2H), 3.85 (s, 3H), 2.69 (q, *J* = 7.6 Hz, 2H), 1.28 (t, *J* = 7.6 Hz, 3H) ppm.
^{13}C NMR (101 MHz, $CDCl_3$) δ 159.1, 142.9, 138.4, 133.9, 128.4, 128.1, 126.8, 114.3, 55.5, 28.6, 15.8 ppm.
HRMS (EI) calculated for $C_{15}H_{16}O$ $[M]^+$: 212.1196, found: 212.1199.

2-Phenyl-9H-fluorene (Figure 3, compound 9)

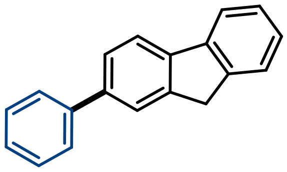

Following *General Procedure F* on a 0.100 mmol scale, the crude product was purified by pTLC (all pentane) to afford the title compound **9** as a white solid (12.5 mg, 52%). The spectral data are consistent with previously reported data within experimental error.[55]

^{1}H NMR (400 MHz, $CDCl_3$) δ 7.86 (d, *J* = 7.9 Hz, 1H), 7.82 (d, *J* = 7.6 Hz, 1H), 7.79 (d, *J* = 0.9 Hz, 1H), 7.70 – 7.65 (m, 2H), 7.65 – 7.62 (m, 1H), 7.58 (d, *J* = 7.4 Hz, 1H), 7.47 (t, *J* = 7.6 Hz, 2H), 7.43 – 7.30 (m, 3H), 3.98 (s, 2H) ppm.

^{13}C NMR (101 MHz, $CDCl_3$) δ 144.0, 143.6, 141.6, 141.5, 141.1, 140.0, 128.9, 127.3, 127.3, 127.0, 126.9, 126.2, 125.2, 124.0, 120.3, 120.1, 37.2 ppm.

HRMS (EI) calculated for $C_{19}H_{14}$ $[M]^+$: 242.1090, found: 242.1091.

1-(4-Methoxyphenyl)naphthalene (Figure 3, compound 10)

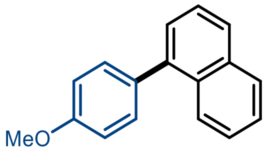

Following *General Procedure F* on a 0.100 mmol scale, the crude product was purified by pTLC (10% EtOAc in pentane) to afford the title compound **10** as a white solid (11.5 mg, 49%). The spectral data are consistent with previously reported data within experimental error.[56]

^{1}H NMR (400 MHz, $CDCl_3$) δ δ 7.95 – 7.89 (m, 2H), 7.84 (d, *J* = 8.2 Hz, 1H), 7.56 – 7.47 (m, 2H), 7.45 – 7.37 (m, 4H), 7.08 – 7.00 (m, 2H), 3.90 (s, 3H).

^{13}C NMR (101 MHz, $CDCl_3$) δ 159.1, 140.1, 134.0, 133.3, 132.0, 131.3, 128.4, 127.5, 127.1, 126.2, 126.1, 125.8, 125.6, 113.9, 55.5 ppm.

HRMS (EI) calculated for $C_{17}H_{14}O$ $[M]^+$: 234.1039, found: 234.1041.

4-Bromo-4'-methoxy-1,1'-biphenyl (Figure 3, compound 11)

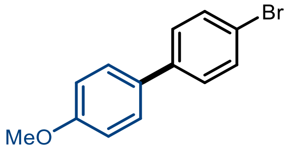

Following *General Procedure F* on a 0.100 mmol scale, the crude product was purified by pTLC (5% EtOAc in pentane) to afford the title compound **11** as a white solid (17.8 mg, 68%). The spectral data are consistent with previously reported data within experimental error.[57]

^{1}H NMR (400 MHz, $CDCl_3$) δ 7.55 – 7.51 (m, 2H), 7.51 – 7.46 (m, 2H), 7.43 – 7.39 (m, 2H), 7.01 – 6.95 (m, 2H), 3.85 (s, 3H) ppm.

^{13}C NMR (101 MHz, $CDCl_3$) δ 159.6, 139.9, 132.6, 131.9, 128.5, 128.1, 120.9, 114.5, 55.5 ppm.

HRMS (EI) calculated for $C_{13}H_{11}OBr$ $[M]^+$: 261.9988, found: 261.9988.

4-Chloro-4'-methoxy-2-methyl-1,1'-biphenyl (Figure 3, compound 12)

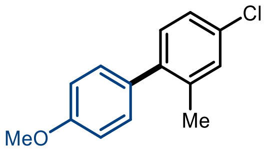

Following *General Procedure F* on a 0.100 mmol scale, the crude product was purified by pTLC (10% EtOAc in pentane) to afford the title compound **12** as a colorless oil (9.6 mg, 41%). The spectral data are consistent with previously reported data within experimental error.[58]

^{1}H NMR (400 MHz, $CDCl_3$) δ 7.25 (d, J = 2.3 Hz, 1H), 7.23 – 7.17 (m, 3H), 7.13 (d, J = 8.2 Hz, 1H), 6.99 – 6.92 (m, 2H), 3.85 (s, 3H), 2.25 (s, 3H) ppm.
^{13}C NMR (101 MHz, $CDCl_3$) δ 158.9, 140.2, 137.5, 133.3, 132.7, 131.2, 130.3, 130.2, 126.0, 113.8, 55.5, 20.6 ppm.
HRMS (EI) calculated for $C_{14}H_{13}ClO$ $[M]^+$: 232.0649, found: 232.0653.

[1,1'-Biphenyl]-4-yl trifluoromethanesulfonate (Figure 3, compound 13)

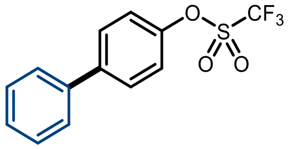

Following *General Procedure F* on a 0.100 mmol scale, the crude product was purified by pTLC (10% EtOAc in pentane) to afford the title compound **13** as a white solid (13.2 mg, 44%). The spectral data are consistent with previously reported data within experimental error.[59]

^{1}H NMR (400 MHz, $CDCl_3$) δ 7.67 – 7.62 (m, 2H), 7.57 – 7.53 (m, 2H), 7.50 – 7.44 (m, 2H), 7.42 – 7.37 (m, 1H), 7.37 – 7.32 (m, 2H) ppm.
^{13}C NMR (101 MHz, $CDCl_3$) δ 149.1, 141.9, 139.5, 129.1, 129.0, 128.2, 127.3, 121.8, 118.9 (q, J = 320.7 Hz) ppm.
^{19}F{^{1}H} NMR (282 MHz, $CDCl_3$) δ –72.82 ppm.
HRMS (EI) calculated for $C_{13}H_9O_3SF_3$ $[M]^+$: 302.0219, found: 302.0224.

2-Phenyldibenzo[b,d]thiophene (Figure 3, compound 14)

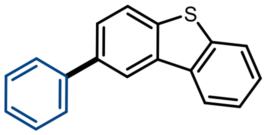

Following *General Procedure F* on a 0.100 mmol scale, the crude product was purified by pTLC (5% EtOAc in pentane) to afford the title compound **14** as a colorless oil (17.2 mg, 66%). The spectral data are consistent with previously reported data within experimental error.[60]

^{1}H NMR (300 MHz, $CDCl_3$) δ 8.36 (d, J = 1.8 Hz, 1H), 8.29 – 8.17 (m, 1H), 7.96 – 7.82 (m, 2H), 7.71 (ddd, J = 8.1, 4.3, 1.7 Hz, 3H), 7.56 – 7.44 (m, 4H), 7.40 (t, J = 7.4 Hz, 1H) ppm.
^{13}C NMR (101 MHz, $CDCl_3$) δ 141.4, 140.1, 138.7, 138.1, 136.3, 135.7, 129.1, 127.6, 127.4, 127.0, 126.3, 124.6, 123.2, 123.1, 121.8, 120.2 ppm.

HRMS (EI) calculated for $C_{18}H_{12}S$ $[M]^+$: 260.0654, found: 260.0654.

2-Phenyldibenzo[b,d]furan (Figure 3, compound 15)

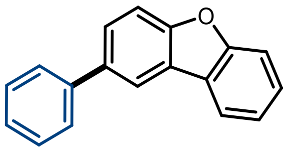

Following *General Procedure F* on a 0.100 mmol scale, the crude product was purified by pTLC (5% EtOAc in pentane) to afford the title compound **15** as a white solid (17.4 mg, 71%). The spectral data are consistent with previously reported data within experimental error.[61]

^{1}H NMR (300 MHz, $CDCl_3$) δ 8.15 (d, *J* = 1.8 Hz, 1H), 8.01 (d, *J* = 6.9 Hz, 1H), 7.73 – 7.57 (m, 5H), 7.49 (t, *J* = 7.6 Hz, 3H), 7.41 – 7.33 (m, 2H) ppm.

^{13}C NMR (101 MHz, $CDCl_3$) δ 156.8, 155.9, 141.5, 136.6, 129.0, 127.6, 127.5, 127.2, 126.8, 124.9, 124.4, 123.0, 120.9, 119.3, 111.9 ppm (one carbon not observed due to overlapping).

HRMS (EI) calculated for $C_{18}H_{12}O$ $[M]^+$: 244.0883, found: 244.0887.

2-(4-Methoxyphenyl)thiophene (Figure 3, compound 16)

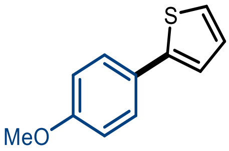

Following *General Procedure F* on a 0.100 mmol scale, the crude product was purified by pTLC (10% EtOAc in pentane) to afford the title compound **16** as a white solid (14.5 mg, 76%). The spectral data are consistent with previously reported data within experimental error.[62]

^{1}H NMR (400 MHz, $CDCl_3$) δ 7.60 – 7.50 (m, 2H), 7.23 – 7.18 (m, 2H), 7.06 (dd, *J* = 5.1, 3.6 Hz, 1H), 6.98 – 6.88 (m, 2H), 3.84 (s, 3H) ppm.

^{13}C NMR (101 MHz, $CDCl_3$) δ 159.3, 144.5, 128.1, 127.5, 127.4, 124.0, 122.2, 114.4, 55.5 ppm.

HRMS (EI) calculated for $C_{11}H_{10}OS$ $[M]^+$: 190.0447, found: 190.0449.

4-(4-Methoxyphenyl)pyridine (Figure 3, compound 17)

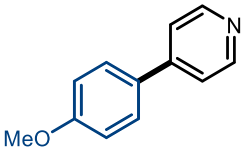

Following *General Procedure F* on a 0.100 mmol scale, the crude product was purified by pTLC (10% EtOAc in hexanes) to afford the title compound **17** as a pale yellow solid (13.2 mg, 71%). The spectral data are consistent with previously reported data within experimental error.[63]

^{1}H NMR (400 MHz, $CDCl_3$) δ 8.62 (dd, *J* = 4.8, 1.6 Hz, 2H), 7.64 – 7.56 (m, 2H), 7.50 – 7.44 (m, 2H), 7.05 – 6.97 (m, 2H), 3.87 (s, 3H) ppm.

^{13}C NMR (101 MHz, $CDCl_3$) δ 160.7, 150.4, 147.9, 130.5, 128.3, 121.2, 114.7, 55.5 ppm.

HRMS (EI) calculated for $C_{12}H_{11}NO$ $[M]^+$: 185.0835, found: 185.0838.

***N*-(3-chloro-4-((3-fluorobenzyl)oxy)phenyl)-6-(4-methoxyphenyl)quinazolin-4-amine (Figure 3, compound 18)**

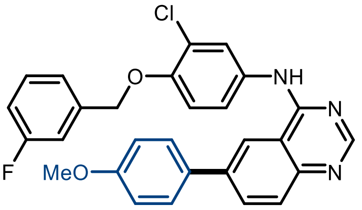

Following *General Procedure F* on a 0.100 mmol scale, the crude product was purified by pTLC (30% EtOAc in CH_2Cl_2) to afford the title compound **18** as a pale yellow solid (35.8 mg, 73%) with small amounts of inseparable impurities.

^{1}H NMR (400 MHz, DMSO-d_6) δ 9.88 (s, 1H), 8.73 (d, *J* = 2.0 Hz, 1H), 8.57 (s, 1H), 8.15 (dd, *J* = 8.7, 1.9 Hz, 1H), 8.03 (d, *J* = 2.6 Hz, 1H), 7.86 – 7.79 (m, 3H), 7.76 (dd, *J* = 8.9, 2.6 Hz, 1H), 7.51 – 7.42 (m, 1H), 7.35 – 7.26 (m, 3H), 7.18 (td, *J* = 8.7, 2.7 Hz, 1H), 7.13 – 7.08 (m, 2H), 5.26 (s, 2H), 3.83 (s, 3H) ppm.
^{13}C NMR (101 MHz, DMSO-d_6) δ 162.2 (d, *J* = 243.7 Hz), 159.3, 157.6, 154.2, 149.7, 148.6, 139.7 (d, *J* = 7.5 Hz), 137.8, 133.2, 131.5, 131.4, 130.6 (d, *J* = 8.2 Hz), 128.3, 128.3, 124.2, 123.3 (d, *J* = 2.7 Hz), 122.4, 121.1, 119.3, 115.3, 114.7 (d, *J* = 21.0 Hz), 114.5, 114.3, 114.0 (d, *J* = 21.9 Hz), 69.4, 55.3 ppm.
^{19}F{^{1}H} NMR (282 MHz, DMSO-d_6) –113.08 ppm.
HRMS (ESI) calculated for $C_{28}H_{22}ClFN_3O_2$ $[M+H]^+$: 486.1379, found: 486.1383.

5.2. Ambiphilic cross-coupling of aryl-bismuths with aryl boron derivatives

4-Fluoro-1,1'-biphenyl (Figure 3, compound 19)

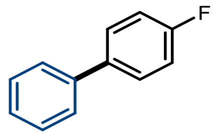

Following *General Procedure G* on a 0.100 mmol scale, the crude product was purified by pTLC (all pentane) to afford the title compound **19** as a colorless oil (14.3 mg, 83%). The spectral data are consistent with previously reported data within experimental error.[64]

^{1}H NMR (400 MHz, $CDCl_3$) δ 7.58 – 7.51 (m, 4H), 7.47 – 7.40 (m, 2H), 7.37 – 7.32 (m, 1H), 7.17 – 7.08 (m, 2H) ppm.

^{13}C NMR (151 MHz, $CDCl_3$) δ 162.6 (d, *J* = 246.3 Hz), 137.5 (d, *J* = 3.2 Hz), 129.0, 128.8 (d, *J* = 8.2 Hz), 127.4, 127.2, 115.8 (d, *J* = 21.4 Hz) ppm.

^{19}F{^{1}H} NMR (282 MHz, $CDCl_3$) δ –115.93 ppm.

HRMS (EI) calculated for $C_{12}H_9F$ $[M]^+$: 172.0683, found: 172.0684.

Methyl [1,1'-biphenyl]-4-carboxylate (Figure 3, compound 20)

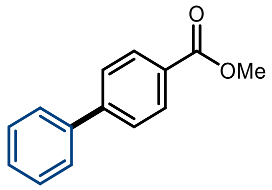

Following *General Procedure G* on a 0.100 mmol scale, the crude product was purified by pTLC (20% EtOAc in cyclohexane) to afford the title compound **20** as a white solid (17.9 mg, 84%). The spectral data are consistent with previously reported data within experimental error.[65]

^{1}H NMR (400 MHz, $CDCl_3$) δ 8.15 – 8.07 (m, 2H), 7.69 – 7.60 (m, 4H), 7.50 – 7.44 (m, 2H), 7.43 – 7.36 (m, 1H) ppm.

^{13}C NMR (101 MHz, $CDCl_3$) δ 167.1, 145.8, 140.2, 130.2, 129.1, 129.0, 128.3, 127.4, 127.2, 52.3 ppm.

HRMS (EI) calculated for $C_{14}H_{12}O_2$ $[M]^+$: 212.0832, found: 212.0835.

3,5-Dimethoxy-1,1'-biphenyl (Figure 3, compound 21)

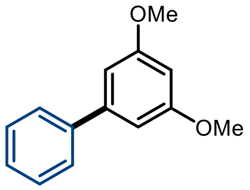

Following *General Procedure G* on a 0.100 mmol scale, the crude product was purified by pTLC (10% EtOAc in pentane) to afford the title compound **21** as a colorless oil (19.2 mg, 90%). The spectral data are consistent with previously reported data within experimental error.[66]

^{1}H NMR (400 MHz, $CDCl_3$) δ 7.61 – 7.56 (m, 2H), 7.47 – 7.40 (m, 2H), 7.38 – 7.33 (m, 1H), 6.74 (d, *J* = 2.3 Hz, 2H), 6.48 (t, *J* = 2.3 Hz, 1H), 3.86 (s, 6H) ppm.

^{13}C NMR (101 MHz, $CDCl_3$) δ 161.2, 143.6, 141.4, 128.8, 127.7, 127.3, 105.6, 99.4, 55.6 ppm.

HRMS (EI) calculated for $C_{14}H_{14}O_2$ $[M]^+$: 214.0988, found: 214.0992.

4-(Trifluoromethoxy)-1,1'-biphenyl (Figure 3, compound 22)

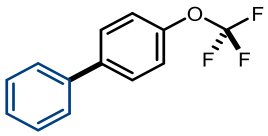

Following *General Procedure G* on a 0.100 mmol scale, the crude product was purified by pTLC (5% CH_2Cl_2 in pentane) to afford the title compound **22** as a white solid (17.4 mg, 73%). The spectral data are consistent with previously reported data within experimental error.[67]

^{1}H NMR (400 MHz, $CDCl_3$) δ 7.64 – 7.58 (m, 2H), 7.58 – 7.54 (m, 2H), 7.49 – 7.43 (m, 2H), 7.42 – 7.34 (m, 1H), 7.33 – 7.26 (m, 2H) ppm.

^{13}C NMR (101 MHz, $CDCl_3$) δ 148.8 (q, J = 1.84 Hz), 140.1, 140.0, 129.1, 128.6, 127.8, 127.3, 121.4, 120.7 (q, J = 257.0 Hz) ppm.

^{19}F{^{1}H} NMR (282 MHz, $CDCl_3$) δ –57.85 ppm.

HRMS (EI) calculated for $C_{13}H_9OF_3$ [M]$^+$: 238.0600, found: 238.0602.

4-Bromo-1,1'-biphenyl (Figure 3, compound 23)

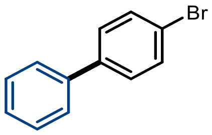

Following *General Procedure G* on a 0.100 mmol scale, the crude product was purified by pTLC (all pentane) to afford the title compound **23** as a white solid (16.5 mg, 70%). The spectral data are consistent with previously reported data within experimental error.[68]

^{1}H NMR (400 MHz, $CDCl_3$) δ 7.61 – 7.52 (m, 4H), 7.48 – 7.41 (m, 4H), 7.39 – 7.33 (m, 1H) ppm.

^{13}C NMR (101 MHz, $CDCl_3$) δ 140.3, 140.2, 132.0, 129.1, 128.9, 127.8, 127.1, 121.7 ppm.

HRMS (EI) calculated for $C_{12}H_9Br$ [M]$^+$: 231.9882, found: 231.9885.

2-Phenylnaphthalene (Figure 3, compound 24)

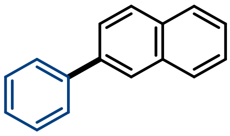

Following *General Procedure G* on a 0.100 mmol scale, the crude product was purified by pTLC (all pentane) to afford the title compound **24** as a white solid (18.8 mg, 92%). The spectral data are consistent with previously reported data within experimental error.[69]

^{1}H NMR (400 MHz, $CDCl_3$) δ 8.10 – 8.02 (m, 1H), 7.96 – 7.85 (m, 3H), 7.79 – 7.72 (m, 3H), 7.55 – 7.46 (m, 4H), 7.42 – 7.37 (m, 1H) ppm.

^{13}C NMR (101 MHz, $CDCl_3$) δ 141.3, 138.7, 133.8, 132.8, 129.0, 128.6, 128.3, 127.8, 127.6, 127.5, 126.4, 126.1, 126.0, 125.7 ppm.

HRMS (EI) calculated for $C_{16}H_{12}$ [M]$^+$: 204.0934, found: 204.0933.

4-((4-Methoxyphenyl)ethynyl)-1,1'-biphenyl (Figure 3, compound 25)

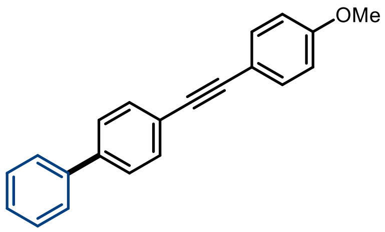

Following *General Procedure G* on a 0.100 mmol scale, the crude product was purified by pTLC (25% CH_2Cl_2 in cyclohexane) to afford the title compound **25** as a white solid (18.6 mg, 65%). The spectral data are consistent with previously reported data within experimental error.[70]

^{1}H NMR (400 MHz, $CDCl_3$) δ 7.63 – 7.60 (m, 2H), 7.59 – 7.57 (m, 4H), 7.52 – 7.48 (m, 2H), 7.47 – 7.43 (m, 2H), 7.39 – 7.34 (m, 1H), 6.92 – 6.87 (m, 2H), 3.84 (s, 3H) ppm.
^{13}C NMR (101 MHz, $CDCl_3$) δ 159.8, 140.8, 140.6, 133.2, 132.0, 129.0, 127.7, 127.2, 127.1, 122.7, 115.6, 114.2, 90.2, 88.2, 55.5 ppm.
HRMS (EI) calculated for $C_{21}H_{16}O$ $[M]^+$: 284.1196, found: 284.1200.

1-([1,1'-Biphenyl]-4-yl)-4-methylpiperazine (Figure 3, compound 26)

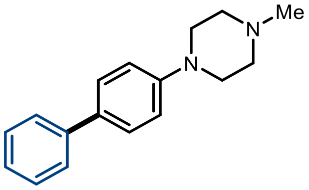

Following *General Procedure G* on a 0.100 mmol scale, the crude product was purified by pTLC (20% MeOH in EtOAc) to afford the title compound **26** as a pale yellow solid (14.2 mg, 56%). The spectral data are consistent with previously reported data within experimental error.[71]

^{1}H NMR (400 MHz, $CDCl_3$) δ 7.58 – 7.54 (m, 2H), 7.54 – 7.50 (m, 2H), 7.45 – 7.36 (m, 2H), 7.31 – 7.26 (m, 1H), 7.04 – 6.96 (m, 2H), 3.30 – 3.23 (m, 4H), 2.62 – 2.58 (m, 4H), 2.37 (s, 3H) ppm.
^{13}C NMR (101 MHz, $CDCl_3$) δ 150.7, 141.1, 132.4, 128.8, 127.9, 126.7, 126.6, 116.2, 55.3, 49.1, 46.3 ppm.
HRMS (EI) calculated for $C_{17}H_{20}N_2$ $[M]^+$: 252.1621, found: 252.1624.

2,2-Difluoro-5-phenylbenzo[d][1,3]dioxole (Figure 3, compound 27)

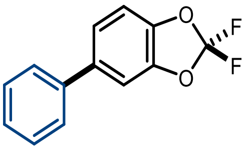

Following *General Procedure G* on a 0.100 mmol scale, the crude product was purified by pTLC (5% EtOAc in pentane) to afford the title compound **27** as a white solid (17.4 mg, 74%).

^{1}H NMR (400 MHz, $CDCl_3$) δ 7.54 – 7.49 (m, 2H), 7.47 – 7.41 (m, 2H), 7.40 – 7.34 (m, 1H), 7.30 – 7.27 (m, 2H), 7.14 – 7.09 (m, 1H) ppm.
^{13}C NMR (101 MHz, $CDCl_3$) δ 144.4, 143.3, 140.2, 138.0, 131.9 (t, J = 255.1 Hz), 129.1, 127.8, 127.3, 122.6, 109.7, 108.7 ppm.
^{19}F{^{1}H} NMR (282 MHz, $CDCl_3$) δ –50.06 ppm.
HRMS (EI) calculated for $C_{13}H_8O_2F_2$ $[M]^+$: 234.0487, found: 234.0491.

3-Phenylthiophene (Figure 3, compound 28)

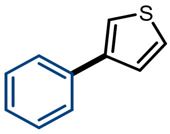

Following *General Procedure G* on a 0.100 mmol scale, the crude product was purified by pTLC (10% CH_2Cl_2 in cyclohexane) to afford the title compound **28** as a white solid (14.5 mg, 90%). The spectral data are consistent with previously reported data within experimental error.[72]

^{1}H NMR (400 MHz, $CDCl_3$) δ 7.64 – 7.58 (m, 2H), 7.46 (dd, *J* = 2.6, 1.8 Hz, 1H), 7.43 – 7.36 (m, 4H), 7.32 – 7.27 (m, 1H) ppm.
^{13}C NMR (101 MHz, $CDCl_3$) δ 142.5, 136.0, 129.0, 127.3, 126.6, 126.5, 126.3, 120.4 ppm.
HRMS (EI) calculated for $C_{10}H_8S$ $[M]^+$: 160.0341, found: 160.0343.

2-Phenylthiophene (Figure 3, compound 29)

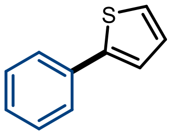

Following *General Procedure G* on a 0.100 mmol scale, the crude product was purified by pTLC (all pentane) to afford the title compound **29** as a white solid (13.2 mg, 82%). The spectral data are consistent with previously reported data within experimental error.[73]

^{1}H NMR (400 MHz, $CDCl_3$) δ 7.66 – 7.58 (m, 2H), 7.42 – 7.36 (m, 2H), 7.34 – 7.26 (m, 3H), 7.09 (dd, *J* = 5.1, 3.6 Hz, 1H) ppm.
^{13}C NMR (101 MHz, $CDCl_3$) δ 144.6, 134.6, 129.0, 128.1, 127.6, 126.1, 124.9, 123.2 ppm.
HRMS (EI) calculated for $C_{10}H_8S$ $[M]^+$: 160.0341, found: 160.0344.

3-Phenylbenzo[b]thiophene (Figure 3, compound 30)

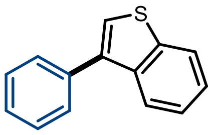

Following *General Procedure G* on a 0.100 mmol scale, the crude product was purified by pTLC (5% EtOAc in pentane) to afford the title compound **30** as a colorless oil (18.6 mg, 88%). The spectral data are consistent with previously reported data within experimental error.[55]

^{1}H NMR (400 MHz, $CDCl_3$) δ 7.97 – 7.89 (m, 2H), 7.64 – 7.57 (m, 2H), 7.54 – 7.46 (m, 2H), 7.45 – 7.36 (m, 4H) ppm.
^{13}C NMR (101 MHz, $CDCl_3$) δ 140.8, 138.3, 138.1, 136.2, 128.9, 127.7, 124.6, 124.5, 123.6, 123.1 ppm (two carbon not observed due to overlapping).
HRMS (EI) calculated for $C_{14}H_{10}S$ $[M]^+$: 210.0498, found: 210.0500.

6-Phenylbenzo[d]thiazole (Figure 3, compound 31)

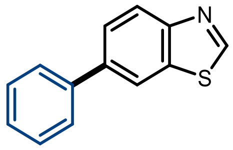

Following *General Procedure G* on a 0.100 mmol scale, the crude product was purified by pTLC (10% EtOAc in pentane) to afford the title compound **31** as an off-white solid (17.9 mg, 85%). The spectral data are consistent with previously reported data within experimental error.[74]

^{1}H NMR (400 MHz, $CDCl_3$) δ 9.00 (s, 1H), 8.20 (dd, J = 8.5, 0.6 Hz, 1H), 8.16 (dd, J = 1.9, 0.6 Hz, 1H), 7.76 (dd, J = 8.5, 1.8 Hz, 1H), 7.68 – 7.62 (m, 2H), 7.51 – 7.45 (m, 2H), 7.43 – 7.36 (m, 1H) ppm.
^{13}C NMR (101 MHz, $CDCl_3$) δ 154.2, 152.7, 140.7, 139.3, 134.7, 129.1, 127.8, 127.6, 126.1, 123.8, 120.3 ppm.
HRMS (EI) calculated for $C_{13}H_9NS$ $[M]^+$: 211.0450, found: 211.0449.

6-Phenylquinoline (Figure 3, compound 32)

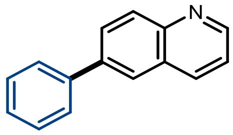

Following *General Procedure G* on a 0.100 mmol scale, the crude product was purified by pTLC (25% EtOAc in pentane) to afford the title compound **32** as an off-white solid (17.5 mg, 85%). The spectral data are consistent with previously reported data within experimental error.[75]

^{1}H NMR (400 MHz, $CDCl_3$) δ 8.93 (dd, J = 4.2, 1.8 Hz, 1H), 8.24 (ddd, J = 8.6, 1.8, 0.9 Hz, 1H), 8.13 (dt, J = 8.5, 1.1 Hz, 1H), 7.76 (dd, J = 8.5, 7.1 Hz, 1H), 7.54 – 7.42 (m, 6H), 7.35 (dd, J = 8.6, 4.2 Hz, 1H) ppm.
^{13}C NMR (101 MHz, $CDCl_3$) δ 150.4, 148.7, 140.6, 139.5, 134.5, 130.2, 129.1, 129.1, 128.6, 127.8, 127.4, 126.9, 121.2 ppm.
HRMS (EI) calculated for $C_{15}H_{11}N$ $[M]^+$: 205.0886, found: 205.0885.

4-([1,1'-Biphenyl]-4-yl)pyridine (Figure 3, compound 33)

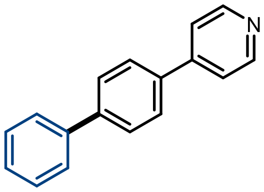

Following *General Procedure G* on a 0.100 mmol scale, the crude product was purified by pTLC (25% EtOAc in pentane) to afford the title compound **33** as an off-white solid (15.5 mg, 67%). The spectral data are consistent with previously reported data within experimental error.[76]

^{1}H NMR (400 MHz, $CDCl_3$) δ 8.70 – 8.66 (m, 2H), 7.75 – 7.70 (m, 4H), 7.67 – 7.61 (m, 2H), 7.58 – 7.53 (m, 2H), 7.51 – 7.45 (m, 2H), 7.43 – 7.36 (m, 1H) ppm.
^{13}C NMR (101 MHz, $CDCl_3$) δ 150.5, 148.0, 142.1, 140.4, 137.1, 129.1, 128.0, 127.9, 127.5, 127.2, 121.6 ppm.
HRMS (EI) calculated for $C_{17}H_{13}N$ $[M]^+$: 231.1042, found: 231.1042.

4-Phenyl-1H-pyrazole (Figure 3, compound 34)

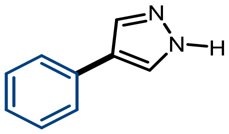

Following *General Procedure G* on a 0.100 mmol scale, the crude product was purified by pTLC (EtOAc 50% in pentane) to afford the title compound **34** as a white solid (10.1 mg, 70%). The spectral data are consistent with previously reported data within experimental error.[77]

^{1}H NMR (400 MHz, DMSO-*d₆*) δ 12.92 (s, 1H), 8.18 (s, 1H), 7.91 (s, 1H), 7.63 – 7.55 (m, 2H), 7.39 – 7.30 (m, 2H), 7.20 – 7.14 (m, 1H) ppm.

^{13}C NMR (101 MHz, DMSO-d_6) δ 136.2, 132.9, 128.7, 125.8, 125.4, 125.1, 121.1 ppm.

HRMS (EI) calculated for $C_9H_8N_2$ $[M]^+$: 144.0682, found: 144.0684.

5-Phenyl-1H-indole (Figure 3, compound 35)

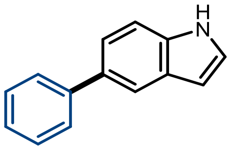

Following *General Procedure G* on a 0.100 mmol scale, the crude product was purified by pTLC (EtOAc 25% in pentane) to afford the title compound **35** as pale orange solid (17.0 mg, 88%). The spectral data are consistent with previously reported data within experimental error.[78]

^{1}H NMR (400 MHz, $CDCl_3$) δ 8.15 (brs, 1H), 7.91 – 7.84 (m, 1H), 7.71 – 7.62 (m, 2H), 7.52 – 7.40 (m, 4H), 7.37 – 7.29 (m, 1H), 7.26 – 7.22 (m, 1H), 6.64 – 6.60 (m, 1H) ppm.

^{13}C NMR (101 MHz, $CDCl_3$) δ 142.7, 135.4, 133.6, 128.8, 128.5, 127.5, 126.5, 125.0, 122.1, 119.4, 111.4, 103.2 ppm.

HRMS (EI) calculated for $C_{14}H_{11}N$ $[M]^+$: 193.0886, found: 193.0888.

5.3. Ambiphilic cross-coupling with structurally diverse aryl-bismuth reagents

1-(4-Fluoro-4'-(trifluoromethyl)-[1,1'-biphenyl]-3-yl)ethan-1-one (Figure 3, compound 36)

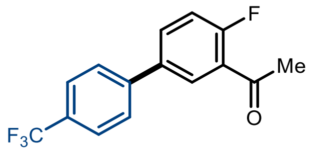

Following *General Procedure F* on a 0.050 mmol scale, the crude product was purified by pTLC (5% EtOAc in pentane) to afford the title compound **36** as an off-white solid (5.6 mg, 40%).

^{1}H NMR (600 MHz, $CDCl_3$) δ 8.11 (dd, *J* = 6.9, 2.6 Hz, 1H), 7.75 (ddd, *J* = 8.5, 4.6, 2.6 Hz, 1H), 7.72 – 7.66 (m, 4H), 7.28 – 7.24 (m, 1H), 2.70 (d, *J* = 5.0 Hz, 3H) ppm.
^{13}C NMR (151 MHz, $CDCl_3$) δ 195.7 (d, *J* = 3.5 Hz), 162.4 (d, *J* = 256.5 Hz), 142.8 (q, *J* = 1.3 Hz), 136.4 (d, *J* = 3.5 Hz), 133.4 (d, *J* = 9.3 Hz), 130.1 (q, *J* = 32.7) 129.5 (d, *J* = 2.9 Hz), 126.1 (d, *J* = 13.6 Hz), 126.1 (q, *J* = 3.8 Hz), 125.2, 123.4, 117.6 (d, *J* = 24.5 Hz), 31.7 (d, *J* = 7.6 Hz) ppm.
^{19}F{^{1}H} NMR (565 MHz, $CDCl_3$) δ –62.55, –110.62 ppm.
HRMS (EI) calculated for $C_{15}H_{10}OF_4$ $[M]^+$: 282.0662, found: 282.0666.

1-(4-Fluoro-[1,1'-biphenyl]-3-yl)ethan-1-one (Figure 3, compound 37)

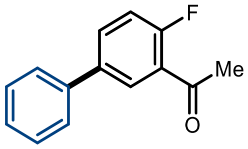

Following *General Procedure F* on a 0.100 mmol scale, the crude product was purified by pTLC (5% EtOAc in pentane) to afford the title compound **37** as a colorless oil (15.6 mg, 73%). The spectral data are consistent with previously reported data within experimental error.[79]

^{1}H NMR (300 MHz, $CDCl_3$) δ 8.10 (dd, *J* = 7.0, 2.5 Hz, 1H), 7.73 (ddd, *J* = 8.6, 4.7, 2.5 Hz, 1H), 7.62 – 7.53 (m, 2H), 7.49 – 7.41 (m, 2H), 7.40 – 7.33 (m, 1H), 7.21 (dd, *J* = 10.7, 8.6 Hz, 1H), 2.69 (d, *J* = 5.0 Hz, 3H) ppm.
^{13}C NMR (101 MHz, $CDCl_3$) δ 196.0 (d, *J* = 3.6 Hz), 161.9 (d, *J* = 255.3 Hz), 139.3, 137.9 (d, *J* = 3.3 Hz), 133.3 (d, *J* = 9.1 Hz), 129.2 (d, *J* = 2.6 Hz), 129.1, 127.9, 127.2, 125.9 (d, *J* = 13.1 Hz), 117.3 (d, *J* = 24.4 Hz), 31.7 (d, *J* = 7.5 Hz) ppm.
^{19}F{^{1}H} NMR (282 MHz, $CDCl_3$) δ –112.28 ppm.
HRMS (EI) calculated for $C_{14}H_{11}OF$ $[M]^+$: 214.0788, found: 214.0791.

1-(4-Fluoro-4'-methoxy-[1,1'-biphenyl]-3-yl)ethan-1-one (Figure 3, compound 38)

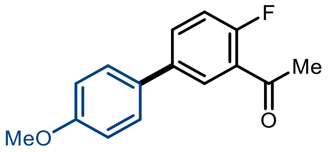

Following *General Procedure F* on a 0.100 mmol scale, the crude product was purified by pTLC (10% EtOAc in pentane) to afford the title compound **38** as a white solid (18.5 mg, 76%).

^{1}H NMR (300 MHz, $CDCl_3$) δ 8.04 (dd, *J* = 7.0, 2.6 Hz, 1H), 7.68 (ddd, *J* = 8.6, 4.7, 2.6 Hz, 1H), 7.53 – 7.46 (m, 2H), 7.18 (dd, *J* = 10.7, 8.5 Hz, 1H), 7.02 – 6.94 (m, 2H), 3.85 (s, 3H), 2.68 (d, *J* = 4.9 Hz, 3H) ppm.

^{13}C NMR (101 MHz, $CDCl_3$) δ 196.1 (d, *J* = 3.4 Hz), 161.6 (d, *J* = 254.3 Hz), 159.6, 137.5 (d, *J* = 3.4 Hz), 132.8 (d, *J* = 8.8 Hz), 131.8, 128.6 (d, *J* = 2.6 Hz), 128.2, 125.8 (d, *J* = 13.3 Hz), 117.2 (d, *J* = 24.1 Hz), 114.5, 55.5, 31.7 (d, *J* = 7.5 Hz) ppm.

^{19}F{^{1}H} NMR (282 MHz, $CDCl_3$) δ –113.13 ppm.

HRMS (EI) calculated for $C_{15}H_{13}O_2F$ $[M]^+$: 244.0894, found: 244.0897.

1-(2-Fluoro-5-(pyrazin-2-yl)phenyl)ethan-1-one (Figure 3, compound 39)

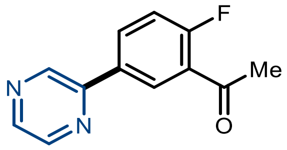

Following *General Procedure F* on a 0.050 mmol scale, the crude product was purified by pTLC (15% EtOAc in pentane) to afford the title compound **39** as an off-white solid (3.8 mg, 35%).

^{1}H NMR (600 MHz, $CDCl_3$) δ 9.06 (d, *J* = 1.6 Hz, 1H), 8.64 (dd, *J* = 2.5, 1.6 Hz, 1H), 8.55 (d, *J* = 2.5 Hz, 1H), 8.52 (dd, *J* = 6.8, 2.4 Hz, 1H), 8.25 (ddd, *J* = 8.6, 4.7, 2.5 Hz, 1H), 7.31 (dd, *J* = 10.7, 8.8 Hz, 1H), 2.71 (d, *J* = 4.8 Hz, 3H) ppm.

^{13}C NMR (151 MHz, $CDCl_3$) δ 195.5 (d, *J* = 3.6 Hz), 163.4 (d, *J* = 258.5 Hz), 151.0, 144.4, 143.6, 142.1, 133.3 (d, *J* = 9.6 Hz), 133.1 (d, *J* = 3.4 Hz), 129.3 (d, *J* = 3.2 Hz), 126.2 (d, *J* = 13.9 Hz), 117.9 (d, *J* = 24.7 Hz), 31.6 (d, *J* = 7.3 Hz) ppm.

^{19}F{^{1}H} NMR (565 MHz, $CDCl_3$) δ –108.06 ppm.

HRMS (EI) calculated for $C_{12}H_9NOF$ $[M]^+$: 216.0693, found: 216.0696.

1-(5-(8-Butoxyquinolin-5-yl)-2-fluorophenyl)ethan-1-one (Figure 3, compound 40)

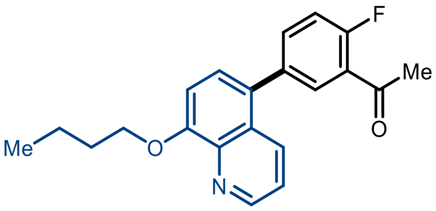

Following *General Procedure F* on a 0.050 mmol scale, the crude product was purified by pTLC (50% EtOAc in pentane) to afford the title compound **40** as a pale yellow oil (7.7 mg, 61%).

^{1}H NMR (300 MHz, $CDCl_3$) δ 8.98 (dd, *J* = 4.2, 1.7 Hz, 1H), 8.08 (dd, *J* = 8.7, 1.8 Hz, 1H), 7.94 (dd, *J* = 7.1, 2.4 Hz, 1H), 7.58 (ddd, *J* = 7.6, 4.7, 2.4 Hz, 1H), 7.42 – 7.35 (m, 2H), 7.31 – 7.22 (m, 1H), 7.11 (d, *J* = 8.1 Hz, 1H), 4.30 (t, *J* = 6.9 Hz, 2H), 2.71 (d, *J* = 4.9 Hz, 3H), 2.11 – 1.95 (m, 2H), 1.66 – 1.57 (m, 2H), 1.03 (t, *J* = 7.4 Hz, 3H) ppm.

^{13}C NMR (101 MHz, $CDCl_3$) δ 196.0 (d, *J* = 3.8 Hz), 161.7 (d, *J* = 255.1 Hz), 154.9, 149.4, 140.6, 136.3 (d, *J* = 9.1 Hz), 136.2 (d, *J* = 3.7 Hz), 133.7, 132.1 (d, *J* = 2.7 Hz), 129.8, 127.8, 127.7, 121.9, 119.5, 117.0 (d, *J* = 24.3 Hz), 108.2, 69.0, 31.7 (d, *J* = 7.2 Hz), 31.1, 19.5, 14.1 ppm.

^{19}F{^{1}H} NMR (282 MHz, $CDCl_3$) δ –111.78 ppm.

HRMS (EI) calculated for $C_{21}H_{20}NO_2F$ $[M]^+$: 337.1473, found: 337.1473.

2-((1-(4-(4-(Pyridin-4-yl)phenoxy)phenoxy)propan-2-yl)oxy)pyridine (Figure 3, compound 41)

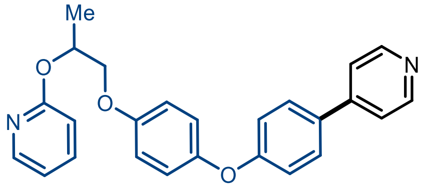

Following *General Procedure F* on a 0.036 mmol scale, the crude product was purified by pTLC (15% EtOAc in pentane) to afford the title compound **41** as a pale yellow oil (8.9 mg, 62%).

^{1}H NMR (400 MHz, $CDCl_3$) δ 8.66 – 8.60 (m, 2H), 8.15 (ddd, *J* = 5.1, 2.0, 0.8 Hz, 1H), 7.60 – 7.54 (m, 3H), 7.48 – 7.44 (m, 2H), 7.06 – 6.92 (m, 6H), 6.87 (ddd, *J* = 7.1, 5.0, 0.9 Hz, 1H), 6.75 (dt, *J* = 8.4, 0.9 Hz, 1H), 5.65 – 5.54 (m, 1H), 4.21 (dd, *J* = 9.9, 5.3 Hz, 1H), 4.09 (dd, *J* = 9.9, 4.9 Hz, 1H), 1.49 (d, *J* = 6.4 Hz, 3H) ppm.

^{13}C NMR (101 MHz, $CDCl_3$) δ 163.3, 159.9, 155.8, 150.4, 149.7, 147.8, 146.9, 138.9, 132.2, 128.4, 121.4, 121.3, 117.9, 116.9, 116.1, 111.8, 71.2, 69.4, 17.2 ppm.

HRMS (ESI) calculated for $C_{25}H_{23}N_2O_3$ $[M+H]^+$: 399.1703, found: 399.1700.

4-Methoxy-4'-(trifluoromethyl)-1,1'-biphenyl (Figure 3, compound 42)

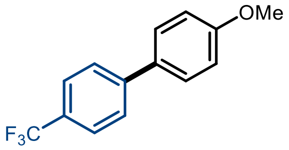

Following *General Procedure G* on a 0.090 mmol scale, the crude product was purified by pTLC (5% EtOAc in pentane) to afford the title compound **42** as a white solid (20.3 mg, 89%). The spectral data are consistent with previously reported data within experimental error.[64]

^{1}H NMR (400 MHz, $CDCl_3$) δ 7.70 – 7.62 (m, 4H), 7.59 – 7.51 (m, 2H), 7.04 – 6.97 (m, 2H), 3.87 (s, 3H) ppm.
^{13}C NMR (101 MHz, $CDCl_3$) δ 160.0, 144.4, 132.3, 128.8 (q, *J* = 32.5 Hz), 128.5, 127.0, 125.8 (q, *J* = 3.7 Hz), 123.2, 114.6, 55.5 ppm.
^{19}F{^{1}H} NMR (282 MHz, $CDCl_3$) δ –62.37 ppm.
HRMS (EI) calculated for $C_{14}H_{11}OF_3$ $[M]^+$: 252.0757, found: 252.0759.

4-(*tert*-Butyl)-4'-methoxy-1,1'-biphenyl (Figure 3, compound 43)

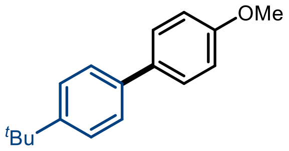

Following *General Procedure G* on a 0.083 mmol scale, the crude product was purified by pTLC (5% EtOAc in pentane) to afford the title compound **43** as a white solid (14.5 mg, 73%). The spectral data are consistent with previously reported data within experimental error.[80]

^{1}H NMR (400 MHz, $CDCl_3$) δ 7.56 – 7.48 (m, 4H), 7.48 – 7.43 (m, 2H), 7.02 – 6.93 (m, 2H), 3.85 (s, 3H), 1.37 (s, 9H) ppm.
^{13}C NMR (101 MHz, $CDCl_3$) δ 159.1, 149.8, 138.1, 133.8, 128.2, 126.5, 125.8, 114.3, 55.5, 34.6, 31.5 ppm.
HRMS (EI) calculated for $C_{17}H_{20}O$ $[M]^+$: 240.1509, found: 240.1512.

3,5-Dibromo-4'-methoxy-1,1'-biphenyl (Figure 3, compound 44)

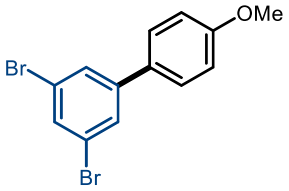

Following *General Procedure G* on a 0.082 mmol scale, the crude product was purified by pTLC (5% EtOAc in pentane) to afford the title compound **44** as a white solid (11.3 mg, 40%). The spectral data are consistent with previously reported data within experimental error.[81]

^{1}H NMR (400 MHz, $CDCl_3$) δ 7.61 (d, *J* = 1.8 Hz, 2H), 7.58 (t, *J* = 1.7 Hz, 1H), 7.49 – 7.43 (m, 2H), 7.01 – 6.93 (m, 2H), 3.86 (s, 3H) ppm.
^{13}C NMR (101 MHz, $CDCl_3$) δ 160.2, 144.6, 132.0, 130.9, 128.6, 128.4, 123.4, 114.6, 55.5 ppm.
HRMS (EI) calculated for $C_{13}H_{10}OBr_2$ $[M]^+$: 339.9093, found: 339.9098.

2-(4-Methoxyphenyl)benzo[b]thiophene (Figure 3, compound 45)

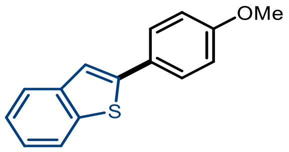

Following *General Procedure G* on a 0.075 mmol scale, the crude product was purified by pTLC (5% EtOAc in pentane) to afford the title compound **45** as a pale yellow solid (11.1 mg, 62%). The spectral data are consistent with previously reported data within experimental error.[82]

^{1}H NMR (400 MHz, $CDCl_3$) δ 7.81 (d, *J* = 8.4 Hz, 1H), 7.75 (d, *J* = 8.0 Hz, 1H), 7.69 – 7.61 (m, 2H), 7.43 (d, *J* = 0.8 Hz, 1H), 7.37 – 7.27 (m, 1H), 7.00 – 6.92 (m, 2H), 3.86 (s, 3H) ppm.
^{13}C NMR (101 MHz, $CDCl_3$) δ 160.0, 144.3, 141.0, 139.3, 127.9, 127.2, 124.6, 124.1, 123.4, 122.3, 118.4, 114.5, 55.5 ppm.
HRMS (EI) calculated for $C_{15}H_{12}OS$ $[M]^+$: 240.0603, found: 240.0607.

6-(4-Methoxyphenyl)quinoline (Figure 3, compound 46)

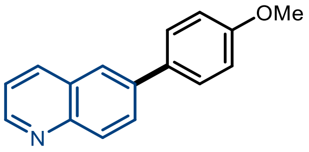

Following *General Procedure G* on a 0.044 mmol scale, the crude product was purified by pTLC (25% EtOAc in pentane) to afford the title compound **46** as an off-white solid (8.8 mg, 85%). The spectral data are consistent with previously reported data within experimental error.[83]

^{1}H NMR (400 MHz, $CDCl_3$) δ 8.90 (dd, *J* = 4.2, 1.7 Hz, 1H), 8.22 – 8.11 (m, 2H), 8.00 – 7.93 (m, 2H), 7.69 – 7.64 (m, 2H), 7.42 (dd, *J* = 8.3, 4.2 Hz, 1H), 7.08 – 7.01 (m, 2H), 3.88 (s, 3H) ppm.

^{13}C NMR (101 MHz, $CDCl_3$) δ 159.7, 150.3, 147.6, 139.1, 136.3, 133.0, 130.0, 129.2, 128.7, 128.7, 124.8, 121.6, 114.6, 55.6 ppm.

HRMS (EI) calculated for $C_{16}H_{13}NO$ $[M]^+$: 235.0992, found: 235.0995.

5-(4-(4-(2-(Pyridin-2-yloxy)propoxy)phenoxy)phenyl)-1H-indole (Figure 3, compound 47)

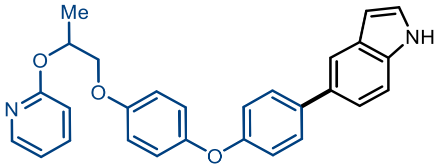

Following *General Procedure G* on a 0.036 mmol scale, the crude product was purified by pTLC (EtOAc 25% in pentane) to afford the title compound **47** as an off-white solid (11.5 mg, 74%).

^{1}H NMR (600 MHz, $CDCl_3$) δ 8.22 (brs, 1H), 8.16 (ddd, *J* = 5.1, 2.0, 0.8 Hz, 1H), 7.83 – 7.80 (m, 1H), 7.59 – 7.55 (m, 3H), 7.44 (dt, *J* = 8.4, 0.8 Hz, 1H), 7.41 (dd, *J* = 8.4, 1.7 Hz, 1H), 7.24 (dd, *J* = 3.2, 2.4 Hz, 1H), 7.03 – 7.00 (m, 4H), 6.97 – 6.93 (m, 2H), 6.87 (ddd, *J* = 7.1, 5.1, 1.0 Hz, 1H), 6.76 (dt, *J* = 8.4, 0.9 Hz, 1H), 6.60 (ddd, *J* = 3.0, 2.0, 0.9 Hz, 1H), 5.63 – 5.57 (m, 1H), 4.20 (dd, *J* = 9.9, 5.3 Hz, 1H), 4.09 (dd, *J* = 9.9, 4.8 Hz, 1H), 1.50 (d, *J* = 6.4 Hz, 3H).

^{13}C NMR (101 MHz, $CDCl_3$) δ 163.3, 157.4, 155.3, 150.7, 146.9, 138.8, 137.2, 135.3, 133.0, 128.6, 128.5, 125.0, 121.9, 120.8, 119.1, 118.0, 116.9, 116.0, 111.8, 111.3, 103.1, 71.2, 69.4, 17.2 ppm.

HRMS (ESI) calculated for $C_{28}H_{25}N_2O_3$ $[M+H]^+$: 437.1860, found: 437.1859.

(*S*)-*N*-(3-(4-(Dibenzo[b,d]furan-2-yl)-2-methylphenoxy)-3-phenylpropyl)-2,2,2-trifluoro-*N*-methylacetamide (Figure 4, compound 48)

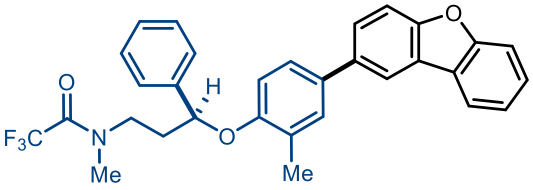

Following *General Procedure F* on a 0.036 mmol scale, the crude product was purified by pTLC (EtOAc 10% in pentane) to afford the title compound **48** as a pale yellow oil (13.1 mg, 71%).

^{1}H NMR (400 MHz, $CDCl_3$, *mixture of rotamers*) δ 8.04 – 8.00 (m, 1H), 7.96 (ddd, *J* = 7.7, 1.4, 0.7 Hz, 1H), 7.59 – 7.53 (m, 3H), 7.49 – 7.43 (m, 2H), 7.41 – 7.23 (m, 7H), 6.67 (dd, *J* = 15.9, 8.5 Hz, 1H), 5.26 (dd, *J* = 8.4, 4.0 Hz, 1H), 3.84 – 3.59 (m, 2H), 3.18 – 3.01 (m, 3H), 2.45 (d, *J* = 3.2 Hz, 3H), 2.38 – 2.17 (m, 2H) ppm.

^{13}C NMR (151 MHz, $CDCl_3$, *mixture of rotamers*) δ 157.1 (q, *J* = 35.9 Hz), 157.0 (q, *J* = 35.9 Hz), 156.7, 155.5, 155.5, 155.1, 155.0, 141.1, 140.7, 136.2, 136.2, 134.0, 133.9, 130.1, 130.0, 129.1, 129.0, 128.2, 128.1, 127.4, 127.4, 127.4, 127.3, 126.4, 125.8, 125.7, 125.6, 125.6, 124.7, 124.7, 124.4, 124.4, 122.9, 122.8, 120.8, 118.8, 116.7 (q, *J* = 287.4 Hz), 116.6 (q, *J* = 287.9 Hz), 113.1, 112.9, 111.9, 111.9, 111.8, 111.8, 77.5, 77.4, 77.2, 77.1, 77.0, 46.8 (q, *J* = 3.3 Hz), 47.3, 37.7, 35.8, 35.5 (q, *J* = 3.9 Hz), 34.9, 16.9, 16.8 ppm.

^{19}F{^{1}H} NMR (565 MHz, $CDCl_3$, *mixture of rotamers*) δ –68.94, –69.85 ppm.

HRMS (ESI) calculated for $C_{31}H_{26}F_3NO_3Na$ $[M+Na]^+$: 540.1757, found: 540.1760.

(*S*)-2,2,2-trifluoro-*N*-methyl-*N*-(3-(2-methyl-4-(6-(trifluoromethyl)pyridin-3-yl)phenoxy)-3-phenylpropyl)acetamide (Figure 4, compound 49)

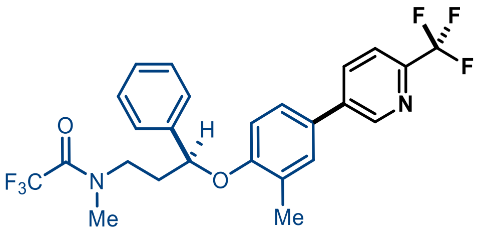

Following *General Procedure F* on a 0.036 mmol scale, the crude product was purified by pTLC (EtOAc 30% in pentane) to afford the title compound **49** as a pale yellow oil (12.1 mg, 69%). *Note: Aryl bromide was used instead of aryl iodide.*

**Variation under standard conditions: ArBi (1.0 equiv), NaI (10.0 equiv), Pd(TFA)$_2$ (12.5 mol%), PAd$_2$(n-Bu) (15.0 mol%), DMA (0.25 M), 70 °C, 20 hours.*

^{1}H NMR (600 MHz, $CDCl_3$, *mixture of rotamers*) δ 8.85 – 8.80 (m, 1H), 7.94 – 7.88 (m, 1H), 7.70 – 7.64 (m, 1H), 7.41 – 7.27 (m, 6H), 7.23 – 7.18 (m, 1H), 6.69 (dd, *J* = 22.3, 8.5 Hz, 1H), 5.26 (dd, *J* = 8.7, 3.9 Hz, 1H), 3.80 – 3.58 (m, 2H), 3.16 – 3.02 (m, 2H), 2.46 – 2.39 (m, 3H), 2.37 – 2.19 (m, 2H) ppm.

^{13}C NMR (151 MHz, $CDCl_3$, *mixture of rotamers*) δ 157.1 (q, *J* = 35.8 Hz), 157.1 (q, *J* = 36.0 Hz), 156.6, 156.4, 148.2, 146.2 (q, *J* = 34.6 Hz), 146.2 (q, *J* = 34.7 Hz), 140.7, 140.2, 139.3, 139.2, 134.9, 134.9, 129.9, 129.8, 129.2, 129.1, 128.8, 128.7, 128.4, 128.3, 128.2, 128.2, 125.9, 125.9, 125.7, 125.6, 121.9 (q, *J* = 273.5 Hz), 121.9 (q, *J* = 273.8 Hz), 116.7 (q, *J* = 287.5 Hz), 116.5 (q, *J* = 287.9 Hz), 113.4, 113.2, 60.5, 47.1, 46.7 (q, *J* = 3.3 Hz), 37.6, 35.8, 35.4 (q, *J* = 3.9 Hz), 34.9, 29.9, 21.2, 16.8, 16.7, 14.3 ppm.

^{19}F{^{1}H} NMR (565 MHz, $CDCl_3$, *mixture of rotamers*) δ –67.66, –67.67, –68.94, –69.88 ppm.

HRMS (ESI) calculated for $C_{25}H_{22}F_6N_2O_2Na$ $[M+Na]^+$: 519.1475, found: 519.1475.

(*S*)-*N*-(3-(4-(2-bromopyridin-4-yl)-2-methylphenoxy)-3-phenylpropyl)-2,2,2-trifluoro-*N*-methylacetamide (Figure 4, compound 50)

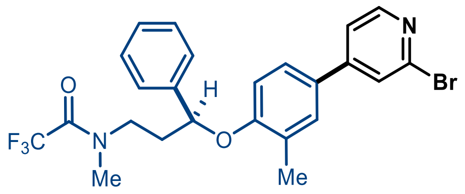

Following *General Procedure F* on a 0.036 mmol scale, the crude product was purified by pTLC (EtOAc 30% in pentane) to afford the title compound **50** as a pale yellow oil (12.2 mg, 68%). *Note: Aryl thianthrenium salt was used instead of aryl iodide.*

^{1}H NMR (600 MHz, $CDCl_3$, *mixture of rotamers*) δ 8.31 (ddd, *J* = 5.2, 2.5, 0.6 Hz, 1H), 7.60 – 7.58 (m, 1H), 7.42 – 7.27 (m, 7H), 7.24 – 7.20 (m, 1H), 6.65 (dd, *J* = 22.9, 8.6 Hz, 1H), 5.26 (dd, *J* = 8.6, 3.9 Hz, 1H), 3.80 – 3.57 (m, 2H), 3.22 – 2.96 (m, 3H), 2.41 (d, *J* = 5.3 Hz, 3H), 2.36 – 2.26 (m, 1H), 2.26 – 2.17 (m, 1H) ppm.

^{13}C NMR (151 MHz, $CDCl_3$, *mixture of rotamers*) δ 157.1 (q, *J* = 35.9 Hz), 157.1 (q, *J* = 36.0 Hz), 157.1, 156.9, 152.1, 151.2, 151.0, 150.9, 150.4, 150.3, 143.0, 143.0, 140.6, 140.1, 129.6, 129.5, 129.2, 129.1, 129.0, 128.8, 128.4, 128.3, 128.1, 128.1, 125.7, 125.7, 125.5, 125.3, 125.2, 120.7, 120.3, 118.4, 116.7 (q, *J* = 287.7 Hz), 116.5 (q, *J* = 287.9 Hz), 113.7, 113.2, 113.1, 60.5, 47.1, 46.7 (q, *J* = 3.3 Hz), 37.6, 35.7, 35.4 (q, *J* = 3.8 Hz), 34.9, 29.8, 21.2, 16.8, 16.7, 14.3 ppm.

^{19}F{^{1}H} NMR (565 MHz, $CDCl_3$, *mixture of rotamers*) δ –68.94, –69.88 ppm.

HRMS (ESI) calculated for $C_{24}H_{23}BrF_3N_2O_2$ $[M+H]^+$: 507.0890, found: 507.0890.

(*S*)-2,2,2-trifluoro-*N*-methyl-*N*-(3-((3-methyl-4'-(trifluoromethyl)-[1,1'-biphenyl]-4-yl)oxy)-3-phenylpropyl)acetamide (Figure 4, compound 51)

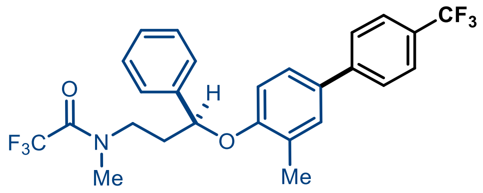

Following *General Procedure F* on a 0.036 mmol scale, the crude product was purified by pTLC (EtOAc 20% in pentane) to afford the title compound **51** as a pale yellow oil (6.2 mg, 35%). *Note: Aryl triflate was used instead of aryl iodide.*

****Variation under standard conditions**: ArBi (1.0 equiv), NaI (10.0 equiv), Pd(TFA)$_2$ (12.5 mol%), PAd$_2$(n-Bu) (15.0 mol%), DMA (0.25 M), 70 °C, 20 hours.*

^{1}H NMR (600 MHz, $CDCl_3$, *mixture of rotamers*) δ 7.65 – 7.56 (m, 4H), 7.42 – 7.32 (m, 5H), 7.32 – 7.27 (m, 1H), 7.23 – 7.17 (m, 1H), 6.65 (dd, *J* = 22.9, 8.5 Hz, 1H), 5.25 (dd, *J* = 8.6, 3.9 Hz, 1H), 3.80 – 3.59 (m, 2H), 3.18 – 2.95 (m, 3H), 2.42 (d, *J* = 5.1 Hz, 3H), 2.37 – 2.26 (m, 1H), 2.26 – 2.19 (m, 1H) ppm.

^{13}C NMR (151 MHz, $CDCl_3$, *mixture of rotamers*) δ 157.07 (q, *J* = 35.9 Hz), 157.06 (q, *J* = 35.9 Hz), 155.97, 155.80, 144.46, 144.39, 140.87, 140.45, 132.29, 132.16, 129.88, 129.80, 129.16, 129.03, 128.89, 128.83, 128.68, 128.61, 128.31, 128.17, 127.69, 127.65, 126.98, 126.96, 125.78, 125.75, 125.73, 125.71, 125.69, 125.64, 125.58, 125.42, 125.40, 123.61, 116.69 (q, *J* = 287.4 Hz), 116.56 (q, *J* = 287.8 Hz), 113.10, 112.91, 60.55, 47.20, 46.78 (q, *J* = 3.3 Hz), 37.70, 35.80, 35.47 (q, *J* = 3.9 Hz), 34.91, 16.82, 16.72 ppm.

^{19}F{^{1}H} NMR (565 MHz, $CDCl_3$, *mixture of rotamers*) δ –62.35, –62.36, –69.0, –69.9 ppm.

HRMS (ESI) calculated for $C_{26}H_{23}F_6NNaO_2$ $[M+Na]^+$: 518.1525, found: 518.1529.

4-methoxy-4'-(trifluoromethyl)-1,1'-biphenyl

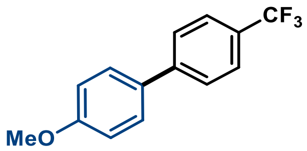

Following *General Procedure F* on a 0.050 mmol scale, the crude product was purified by pTLC (EtOAc 5% in pentane) to afford the title compound as a white solid (6.9 mg, 55%). The spectral data are consistent with previously reported data within experimental error.[64] *Note: Aryl triflate was used instead of aryl iodide.*

***Variation under standard conditions**: *ArBi (1.0 equiv), NaI (10.0 equiv), Pd(TFA)$_2$ (12.5 mol%), PAd$_2$(n-Bu) (15.0 mol%), DMA (0.25 M), 70 °C, 20 hours.*

^{1}H NMR (400 MHz, CDCl$_3$) δ 7.70 – 7.60 (m, 4H), 7.58 – 7.51 (m, 2H), 7.03 – 6.95 (m, 2H), 3.87 (s, 3H) ppm.
^{13}C NMR (101 MHz, CDCl$_3$) δ 160.00, 144.45, 132.34, 128.84 (q, *J* = 32.5 Hz), 128.51, 127.02, 125.82 (q, *J* = 3.9 Hz), 114.58, 55.54 ppm.
^{19}F{^{1}H} NMR (376 MHz, CDCl$_3$) δ –62.33 ppm.

(*S*)-2,2,2-trifluoro-*N*-methyl-*N*-(3-(2-methyl-4-(1-tosyl-1,2,3,6-tetrahydropyridin-4-yl)phenoxy)-3-phenylpropyl)acetamide (Figure 4, compound 52)

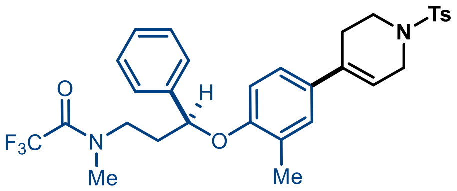

Following *General Procedure F* on a 0.036 mmol scale, the crude product was purified by pTLC (EtOAc 30% in pentane) to afford the title compound **52** as a pale yellow oil (7.4 mg, 36%). *Note: vinyl triflate was used instead of aryl iodide.*

^{1}H NMR (600 MHz, CDCl$_3$, *mixture of rotamers*) δ 7.71 – 7.66 (m, 2H), 7.36 – 7.23 (m, 7H), 7.08 (ddd, *J* = 8.0, 2.4, 0.9 Hz, 1H), 6.91 – 6.86 (m, 1H), 6.49 (dd, *J* = 22.8, 8.6 Hz, 1H), 5.82 – 5.75 (m, 1H), 5.17 (dd, *J* = 8.6, 4.0 Hz, 1H), 3.75 – 3.55 (m, 4H), 3.13 – 2.99 (m, 2H), 2.54 – 2.47 (m, 3H), 2.42 (s, 3H), 2.32 (d, *J* = 5.1 Hz, 3H), 2.29 – 2.14 (m, 2H) ppm.
^{13}C NMR (151 MHz, CDCl$_3$, *mixture of rotamers*) δ 157.2, 157.1, 156.9, 156.9, 155.3, 155.1, 143.7, 143.7, 141.0, 140.6, 134.9, 134.8, 133.3, 133.3, 132.8, 132.6, 129.8, 129.1, 129.0, 128.2, 128.1, 127.9, 127.7, 127.6, 126.9, 126.9, 125.7, 125.6, 123.4, 123.3, 117.5, 117.4, 112.5, 112.3, 60.5, 47.2, 46.8 (q, *J* = 3.2 Hz), 45.4, 43.2, 37.7, 35.8, 35.5 (q, *J* = 3.7 Hz), 34.9, 27.7, 27.7, 21.7, 21.2, 16.8, 16.7, 14.4 ppm.
^{19}F{^{1}H} NMR (565 MHz, CDCl$_3$, *mixture of rotamers*) δ –69.00, –69.90 ppm.
HRMS (ESI) calculated for $C_{31}H_{33}F_3N_2NaO_4S$ $[M+Na]^+$: 609.2005, found: 609.2003.

1-Tosyl-1,2,3,6-tetrahydropyridin-4-yl trifluoromethanesulfonate

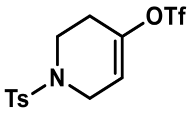

The LDA solution was prepared as follows: n-BuLi (0.37 mL, 2.35 M in hexanes, 0.87 mmol, 1.1 equiv.) was added to a solution of diidopropylamine (0.12 mL, 0.87 mmol, 1.1 equiv.) in anhydrous THF (5.0 mL, 0.17 M) at –78 °C and stirred at this temperature for 15 min to give the LDA solution.

Next, a solution of 1-tosylpiperidin-4-one (200 mg, 0.79 mmol, 1.0 equiv.) in anhydrous THF (2.5 mL, 0.32 M) was added to the LDA solution at –78 °C and the mixture was stirred at this temperature for 30 min. A solution of *N*-phenyltrifluoromethanesulfonimide (282 mg, 0.79 mmol, 1.0 equiv.) in THF (4 mL, 0.20 M) was added dropwise and the reaction mixture was stirred at –78 °C for a further 1 h, after which the reaction mixture was quenched with water and extracted three times with EtOAc. The combined organic layers were dried over anhydrous $MgSO_4$, filtered and concentrated under reduced pressure. Purification by flash column chromatography afforded the product (191 mg, 63% yield) as a waxy white solid. The spectral data are consistent with previously reported data within experimental error.[84]

^{1}H NMR (400 MHz, $CDCl_3$) δ 7.71 – 7.63 (m, 2H), 7.36 – 7.31 (m, 2H), 5.76 – 5.69 (m, 1H), 3.78 (q, *J* = 3.0 Hz, 2H), 3.35 (t, *J* = 5.7 Hz, 2H), 2.51 – 2.45 (m, 2H), 2.44 (s, 3H) ppm.

^{13}C NMR (101 MHz, $CDCl_3$) δ 146.6, 144.4, 133.4, 130.1, 127.6, 118.5 (q, *J* = 320.5 Hz), 114.6, 43.5, 42.8, 28.0, 21.7 ppm.

^{19}F NMR (376 MHz, $CDCl_3$) δ –73.71(s).

HRMS (ESI) calculated for $C_{13}H_{14}NO_5S_2F_3Na$ $[M+Na]^+$: 408.0158, found: 408.0159.

(*S*)-*N*-(3-(4-(Benzo[b]thiophen-3-yl)-2-methylphenoxy)-3-phenylpropyl)-2,2,2-trifluoro-*N*-methylacetamide (Figure 4, compound 53)

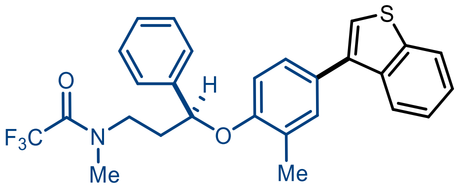

Following *General Procedure G* on a 0.036 mmol scale, the crude product was purified by pTLC (EtOAc 10% in pentane) to afford the title compound **53** as a pale yellow oil (8.3 mg, 51%).

^{1}H NMR (400 MHz, $CDCl_3$, *mixture of rotamers*) δ 7.91 – 7.81 (m, 2H), 7.42 – 7.27 (m, 8H), 7.18 (dt, *J* = 8.4, 2.1 Hz, 1H), 6.67 (dd, *J* = 14.9, 8.4 Hz, 1H), 5.26 (dd, *J* = 8.5, 4.0 Hz, 1H), 3.85 – 3.58 (m, 2H), 3.14 (q, *J* = 1.5 Hz, 2H), 3.05 (s, 1H), 2.42 (d, *J* = 3.4 Hz, 3H), 2.37 – 2.17 (m, 2H) ppm.

^{13}C NMR (151 MHz, $CDCl_3$, *mixture of rotamers*) δ 157.1 (q, *J* = 35.8 Hz), 157.0 (q, *J* = 35.8 Hz), 155.3, 155.2, 141.1, 140.7, 140.6, 138.2, 138.1, 137.9, 137.8, 131.3, 131.2, 129.1, 129.0, 128.6, 128.5, 128.3, 128.1, 127.4, 127.3, 127.1, 127.0, 125.8, 125.6, 124.4, 124.4, 124.3, 124.3, 123.1, 123.1, 123.0, 123.0, 122.6, 122.6, 116.7 (q, *J* = 287.7 Hz), 116.6 (q, *J* = 287.7 Hz), 112.9, 112.7, 77.5, 77.4, 77.2, 77.0, 47.3, 46.9 (q, *J* = 3.2 Hz), 37.8, 35.9, 35.5 (q, *J* = 3.8 Hz), 34.9, 16.8, 16.7 ppm.

^{19}F{^{1}H} NMR (565 MHz, $CDCl_3$, *mixture of rotamers*) δ –68.95, –69.85 ppm.

HRMS (ESI) calculated for $C_{27}H_{24}F_3NO_2SNa$ $[M+Na]^+$: 506.1372, found: 506.1373.

(*S*)-*N*-(3-((3'-cyano-3-methyl-[1,1'-biphenyl]-4-yl)oxy)-3-phenylpropyl)-2,2,2-trifluoro-*N*-methylacetamide (Figure 4, compound 54)

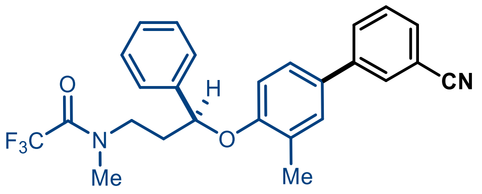

Following *General Procedure F* on a 0.036 mmol scale, the crude product was purified by pTLC (EtOAc 20% in pentane) to afford the title compound **54** as a pale yellow oil (7.2 mg, 45%) with small amounts of inseparable impurities. *Note: Aryl boronic acid pinacol ester was used instead of potassium cyclic triol borate with potassium phosphate (2.0 equiv.) as an additive.*

^{1}H NMR (600 MHz, $CDCl_3$, *mixture of rotamers*) δ 7.77 – 7.73 (m, 1H), 7.71 – 7.66 (m, 1H), 7.56 – 7.52 (m, 1H), 7.50 – 7.44 (m, 1H), 7.40 – 7.31 (m, 5H), 7.31 – 7.27 (m, 1H), 7.17 – 7.12 (m, 1H), 6.65 (dd, *J* = 22.4, 8.5 Hz, 1H), 5.25 (dd, *J* = 8.6, 3.9 Hz, 1H), 3.82 – 3.57 (m, 2H), 3.17 – 3.01 (m, 3H), 2.44 – 2.37 (m, 3H), 2.36 – 2.17 (m, 2H) ppm.

^{13}C NMR (151 MHz, $CDCl_3$, *mixture of rotamers*) δ 157.1 (q, *J* = 35.9 Hz), 156.3, 156.1, 155.9, 142.2, 142.1, 140.8, 140.3, 135.7, 131.4, 131.3, 131.1, 130.7, 130.3, 130.3, 130.2, 130.1, 129.7, 129.6, 129.6, 129.2, 129.0, 128.9, 128.3, 128.2, 127.9, 127.9, 127.8, 125.7, 125.6, 125.5, 125.5, 124.6, 120.6, 119.1, 119.1, 118.8, 116.7 (d, *J* = 287.5 Hz), 116.5 (q, *J* = 287.7 Hz), 113.6, 113.4, 113.2, 113.0, 112.9, 112.9, 60.6, 47.2, 46.8 (q, *J* = 3.4 Hz), 37.6, 35.8, 35.5 (q, *J* = 3.8 Hz), 34.9, 25.0, 25.0, 21.2, 16.8, 16.7, 14.3 ppm.

^{19}F{^{1}H} NMR (565 MHz, $CDCl_3$, *mixture of rotamers*) δ –68.95, –69.87 ppm.

HRMS (ESI) calculated for $C_{26}H_{23}F_3N_2O_2Na$ $[M+Na]^+$: 475.1604, found: 475.1602.

(*S*)-*N*-(3-((3',5'-dimethoxy-3-methyl-[1,1'-biphenyl]-4-yl)oxy)-3-phenylpropyl)-2,2,2-trifluoro-*N*-methylacetamide (Figure 4, compound 55)

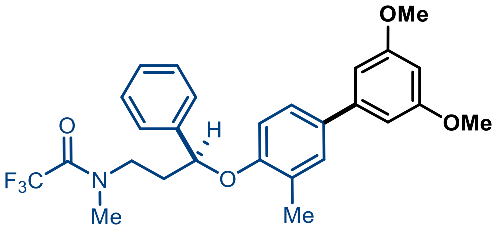

Following *General Procedure F* on a 0.036 mmol scale, the crude product was purified by pTLC (EtOAc 30% in pentane) to afford the title compound **55** as a pale yellow oil (7.4 mg, 43%) with small amounts of inseparable impurities. *Note: Aryl boronic acid was used instead of potassium cyclic triol borate with potassium phosphate (2.0 equiv.) as an additive.*

^{1}H NMR (600 MHz, $CDCl_3$, *mixture of rotamers*) δ 7.39 – 7.32 (m, 5H), 7.31 – 7.26 (m, 1H), 7.19 – 7.14 (m, 1H), 6.64 – 6.57 (m, 3H), 6.40 (q, *J* = 2.2 Hz, 1H), 5.23 (dd, *J* = 8.7, 3.9 Hz, 1H), 3.81 (s, 6H), 3.77 – 3.59 (m, 2H), 3.14 – 3.03 (m, 3H), 2.42 – 2.38 (m, 3H), 2.33 – 2.16 (m, 2H) ppm.

^{13}C NMR (151 MHz, $CDCl_3$, *mixture of rotamers*) δ 161.1, 161.1, 157.1 (q, *J* = 35.8 Hz), 157.0 (q, *J* = 35.8 Hz), 155.5, 155.3, 143.2, 143.2, 141.0, 140.6, 133.8, 133.7, 129.8, 129.8, 129.1, 129.0, 128.2, 128.1, 127.3, 127.2, 125.7, 125.6, 125.5, 125.4, 116.7 (q, *J* = 287.4 Hz), 116.5 (q, *J* = 287.9 Hz), 112.9, 112.7, 105.1, 105.1, 98.8, 60.6, 55.5, 47.3, 46.8 (q, *J* = 3.3 Hz), 37.7, 35.8, 35.5 (q, *J* = 3.8 Hz), 34.9, 16.8, 16.7, 14.4 ppm.

^{19}F{^{1}H} NMR (565 MHz, $CDCl_3$, *mixture of rotamers*) δ –68.97, –69.87 ppm.

HRMS (ESI) calculated for $C_{27}H_{28}NO_4F_3Na$ $[M+Na]^+$: 510.1863, found: 510.1866.

5.4. Practical synthetic usage of ambiphilic coupling: case studies

	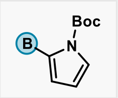	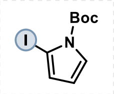	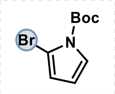	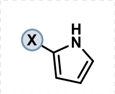	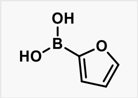	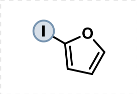	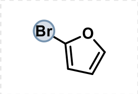
**CAS**	135884-31-0 ($B(OH)_2$)<br>1072944-98-9 (Bpin)	117657-39-3	117657-37-1	67655-27-0 (I)<br>38480-28-3 (Br)	13331-23-2	54829-48-0	584-12-3
**Merck**	1 g/115 €<br>1g/ 10 € (Bpin)<br>5g/ 22 € (Bpin)	-	1 g/725 €<br>5 g/1455 €	-	1 g/96 €<br>10 g/658 €	500 mg/614 €	100 mg/44 €<br>5 g/150 €
**TCI**	1 g/54 €<br>5 g/170 €	-	-	-	1 g/22 €<br>5 g/68 €	-	1g/ 39 €
**Thermo Fisher**	1 g/73 € (Bpin)<br>5 g/281 € (Bpin)	-	-	-	1 g/55 €<br>5 g/169 €<br>25 g/640 €	-	-
**BLD**	1 g/10 €<br>1 g/10 € (Bpin)<br>25 g/33 €<br>100g/ 349 € (Bpin)<br>500 g/654 €	-	-	1 g/976 € (I)<br>- (Br)	25 g/42 €<br>100 g/144 €<br>500 g/715 €	100 mg/278 €	-

*Prices are based on values as of February 10, 2026

Figure S10. Price comparison of aryl boron derivatives and electron-rich 2-haloheteroarenes from commercial vendors

tert-Butyl 2-phenyl-1H-pyrrole-1-carboxylate (Figure 5, compound 56)

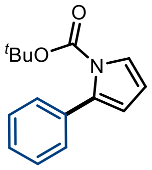

Following *General Procedure G* on a 0.100 mmol scale, the crude product was purified by pTLC (5% EtOAc in pentane) to afford the title compound **56** as a colorless oil (12.4 mg, 51%). The spectral data are consistent with previously reported data within experimental error.[85]

^{1}H NMR (600 MHz, $CDCl_3$) δ 7.36 – 7.33 (m, 5H), 7.32 – 7.28 (m, 1H), 6.23 (t, *J* = 3.3 Hz, 1H), 6.19 (dd, *J* = 3.3, 1.8 Hz, 1H), 1.35 (s, 9H) ppm.
^{13}C NMR (151 MHz, $CDCl_3$) δ 149.5, 135.2, 134.6, 129.3, 127.7, 127.3, 122.6, 114.5, 110.7, 83.7, 27.7 ppm.
HRMS (EI) calculated for $C_{15}H_{17}NO_2$ $[M]^+$: 243.1254, found: 243.1252.
Note: Aryl boronic acid pinacol ester was used with potassium phosphate (2.0 equiv.) as an additive.

2-Phenylfuran (Figure 5, compound 57)

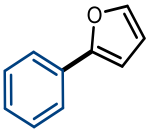

Following *General Procedure G* on a 0.100 mmol scale, the crude product was purified by pTLC (all pentane) to afford the title compound **57** (46% ^{1}H NMR yield). Because the title compound is volatile and readily evaporates under high vacuum, only the NMR yield is reported here. The spectral data are consistent with previously reported data within experimental error.[64]

^{1}H NMR (600 MHz, $CDCl_3$) δ 7.69 – 7.65 (m, 2H), 7.47 (dd, *J* = 1.8, 0.8 Hz, 1H), 7.40 – 7.36 (m, 2H), 7.28 – 7.24 (m, 1H), 6.65 (dd, *J* = 3.3, 0.8 Hz, 1H), 6.47 (dd, *J* = 3.4, 1.8 Hz, 1H) ppm.
^{13}C NMR (151 MHz, $CDCl_3$) δ 154.1, 142.2, 131.0, 128.8, 127.5, 123.9, 111.8, 105.1 ppm.
HRMS (EI) calculated for $C_{10}H_8O$ $[M]^+$: 144.0570, found: 144.0570.
Note: Aryl boronic acid was used with potassium phosphate (2.0 equiv.) as an additive.

4-Methoxy-1,1'-biphenyl (Figure 5, compound 58)

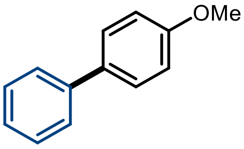

Following *General Procedure G* on a 0.100 mmol scale, the crude product was purified by pTLC (10% CH_2Cl_2 in cyclohexane) to afford the title compound **58** as a white solid (15.7 mg, 85%). The spectral data are consistent with previously reported data within experimental error.[50]

^{1}H NMR (400 MHz, $CDCl_3$) δ 7.60 – 7.50 (m, 4H), 7.47 – 7.38 (m, 2H), 7.34 – 7.28 (m, 1H), 7.02 – 6.96 (m, 2H), 3.86 (s, 3H) ppm.
^{13}C NMR (101 MHz, $CDCl_3$) δ 159.3, 141.0, 133.9, 128.9, 128.3, 126.9, 126.8, 114.3, 55.5 ppm.
HRMS (EI) calculated for $C_{13}H_{12}O$ $[M]^+$: 184.0883, found: 184.0886.

4'-Methoxy-3,5-bis(trifluoromethyl)-1,1'-biphenyl (Figure 5, compound 59)

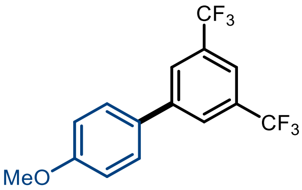

Following *General Procedure F* on a 0.100 mmol scale, the crude product was purified by pTLC (5% EtOAc in cyclohexane) to afford the title compound **59** as a pale yellow oil (21.7 mg, 68%). The spectral data are consistent with previously reported data within experimental error.[86]

^{1}H NMR (400 MHz, $CDCl_3$) δ 7.97 (s, 2H), 7.80 (s, 1H), 7.60 – 7.51 (m, 2H), 7.07 – 6.99 (m, 2H), 3.88 (s, 3H).
^{13}C NMR (101 MHz, $CDCl_3$) δ 160.5, 143.0, 132.2 (q, *J* = 33.1 Hz), 130.8, 128.5, 126.8 (m), 123.6 (q, *J* = 272.6 Hz), 120.4 (p, *J* = 3.9 Hz), 114.9, 55.6 ppm.
^{19}F{^{1}H} NMR (282 MHz, $CDCl_3$) δ –62.91 ppm.
HRMS (EI) calculated for $C_{15}H_{10}OF_6$ $[M]^+$: 320.0630, found: 320.0636.

2-Phenylquinoxaline (Figure 5, compound 60)

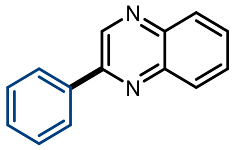

Following *General Procedure F* on a 0.100 mmol scale, the crude product was purified by pTLC (15% EtOAc in pentane) to afford the title compound **60** as pale yellow solid (15.7 mg, 76%). The spectral data are consistent with previously reported data within experimental error.[87]

^{1}H NMR (400 MHz, $CDCl_3$) δ 9.34 (s, 1H), 8.24 – 8.10 (m, 4H), 7.83 – 7.73 (m, 2H), 7.61 – 7.51 (m, 3H).
^{13}C NMR (101 MHz, $CDCl_3$) δ 152.0, 143.6, 142.5, 141.8, 137.0, 130.5, 130.4, 129.8, 129.7, 129.3, 129.3, 127.7 ppm.
HRMS (EI) calculated for $C_{14}H_{10}N_2$ $[M]^+$: 206.0838, found: 206.0840.

2-Phenylpyrazine (Figure 5, compound 61)

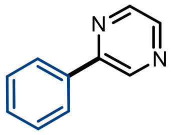

Following *General Procedure F* on a 0.100 mmol scale, the crude product was purified by pTLC (25% EtOAc in pentane) to afford the title compound **61** as a white solid (10.3 mg, 65%). The spectral data are consistent with previously reported data within experimental error.[88]

^{1}H NMR (400 MHz, $CDCl_3$) δ 9.04 (d, *J* = 1.6 Hz, 1H), 8.64 (dd, *J* = 2.5, 1.6 Hz, 1H), 8.51 (d, *J* = 2.5 Hz, 1H), 8.05 – 7.98 (m, 2H), 7.55 – 7.43 (m, 3H) ppm.
^{13}C NMR (101 MHz, $CDCl_3$) δ 153.0, 144.3, 143.1, 142.4, 136.5, 130.1, 129.2, 127.1 ppm.
HRMS (EI) calculated for $C_{10}H_8O_2$ $[M]^+$: 156.0682, found: 156.0684.

6. Mechanistic Investigations

$(PPh_3)_2Pd(II)(4\text{-}F\text{-}Ph)(I)$ (Figure 2, OAC-1)

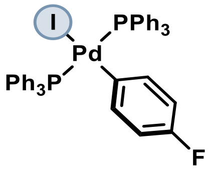

The title compound was prepared according to a modified literature procedure as follows[89]:

In an argon-filled glovebox, a heat-gun-dried Schlenk flask equipped with a Teflon-coated magnetic stir bar was charged with $Pd(PPh_3)_4$ (1.155 g, 1.000 mmol, 1.000 equiv.). The reaction flask was sealed and removed from the glovebox. Anhydrous toluene (20 mL) and 4-fluoroiodobenzene (0.23 mL, 2.0 mmol, 2.0 equiv.) were then sequentially added to the Schlenk flask. The reaction mixture was stirred at room temperature for 20 h in the dark under aluminum foil. After completion, the reaction mixture was filtered, and the solid residue was washed with MeOH (20 mL) and diethyl ether (20 mL) to afford the title compound as an off-white solid (560 mg, 66%).

^{1}H NMR (300 MHz, $CDCl_3$) δ 7.59 – 7.43 (m, 12H), 7.39 – 7.30 (m, 6H), 7.30 – 7.17 (m, 12H), 6.55 – 6.42 (m, 2H), 6.03 (t, J = 9.0 Hz, 2H) ppm.
^{13}C NMR (151 MHz, $CDCl_3$) δ 160.3 (dt, J = 239.3, 1.6 Hz), 151.8 (q, J = 2.7 Hz), 135.9 (q, J = 5.5 Hz), 135.0 (t, J = 6.2 Hz), 132.1 (t, J = 23.3 Hz), 130.0, 128.0 (t, J = 5.1 Hz), 114.6 (d, J = 19.2 Hz) ppm.
^{31}P NMR (122 MHz, $CDCl_3$) δ 22.70 (d, J = 3.3 Hz) ppm.
^{19}F{^{1}H} NMR (282 MHz, $CDCl_3$) δ –125.11 (t, J = 3.5 Hz) ppm.
HRMS (ESI) calculated for $C_{42}H_{34}FP_2Pd$ $[M–I]^+$: 725.1149, found: 725.1146.

Figure S11. Control experiment using **OAC-1** as the precatalyst

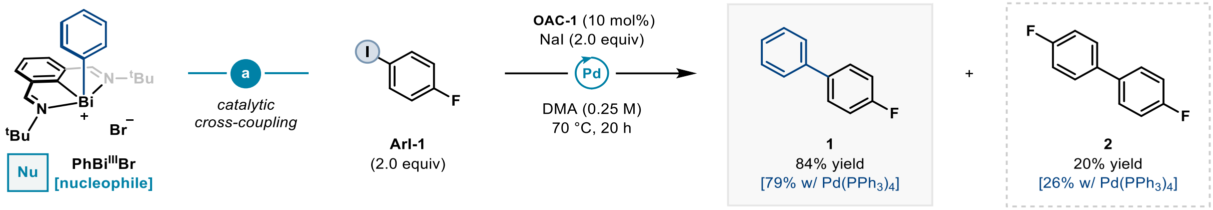

In an argon-filled glovebox, an oven-dried (80 °C, overnight) 10 mL thick-walled culture tube equipped with a Teflon-coated magnetic stir bar was charged with **ArBi(Br)-1** (61 mg, 0.10 mmol, 1.0 equiv.), **OAC-1** (8.53 mg, 0.0100 mmol, 10.0 mol%), and NaI (30 mg, 0.20 mmol, 2.0 equiv.). Anhydrous dimethylacetamide (DMA, 0.40 mL) and **ArI-1** (23 µL, 0.20 mmol, 2.0 equiv.) were then sequentially added. The reaction tube was sealed with a screw cap and removed from the glovebox. The mixture was stirred at 70 °C in a preheated heating block for 20 h. Upon completion, the reaction yield was determined by ^{19}F NMR analysis using trifluorotoluene (10 µL, 0.0821 mmol, 0.821 equiv.) as an internal standard.

Figure S12. Control experiment employing OAC-1 as a stoichiometric electrophile

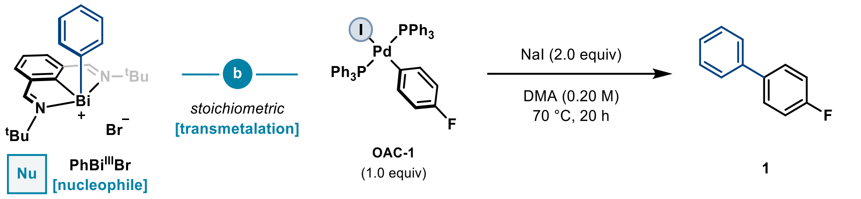

	variation	1^b	2^b
1	none	33%	6%
2	none^a	23%	6%
3	w/o NaI	25%	5%
4	PPh_3 (2.0)	21%	13%
5	$AgBF_4$ (2.0)	n.d.	n.d.
6	DMA (0.083 M)	29%	5%
7	ArI (1.0)	80%	27%
8	ArI (2.0)^a	78%	45%

^{a}0.05 mmol scale. ^{b}determined by ^{19}F NMR

In an argon-filled glovebox, an oven-dried (80 °C, overnight) 10 mL thick-walled culture tube equipped with a Teflon-coated magnetic stir bar was charged with **ArBi(Br)-1** (12.2 mg, 0.0200 mmol, 1.00 equiv.), **OAC-1** (17.1 mg, 0.0200 mmol, 1.00 equiv.), and NaI (6.0 mg, 0.040 mmol, 2.0 equiv.). Anhydrous dimethylacetamide (DMA, 0.10 mL) was then added. The reaction tube was sealed with a screw cap and removed from the glovebox. The mixture was stirred at 70 °C in a preheated heating block for 20 h. Upon completion, the reaction yield was determined by ^{19}F NMR analysis using trifluorotoluene (10.0 μL, 0.0821 mmol, 4.10 equiv.) as an internal standard.

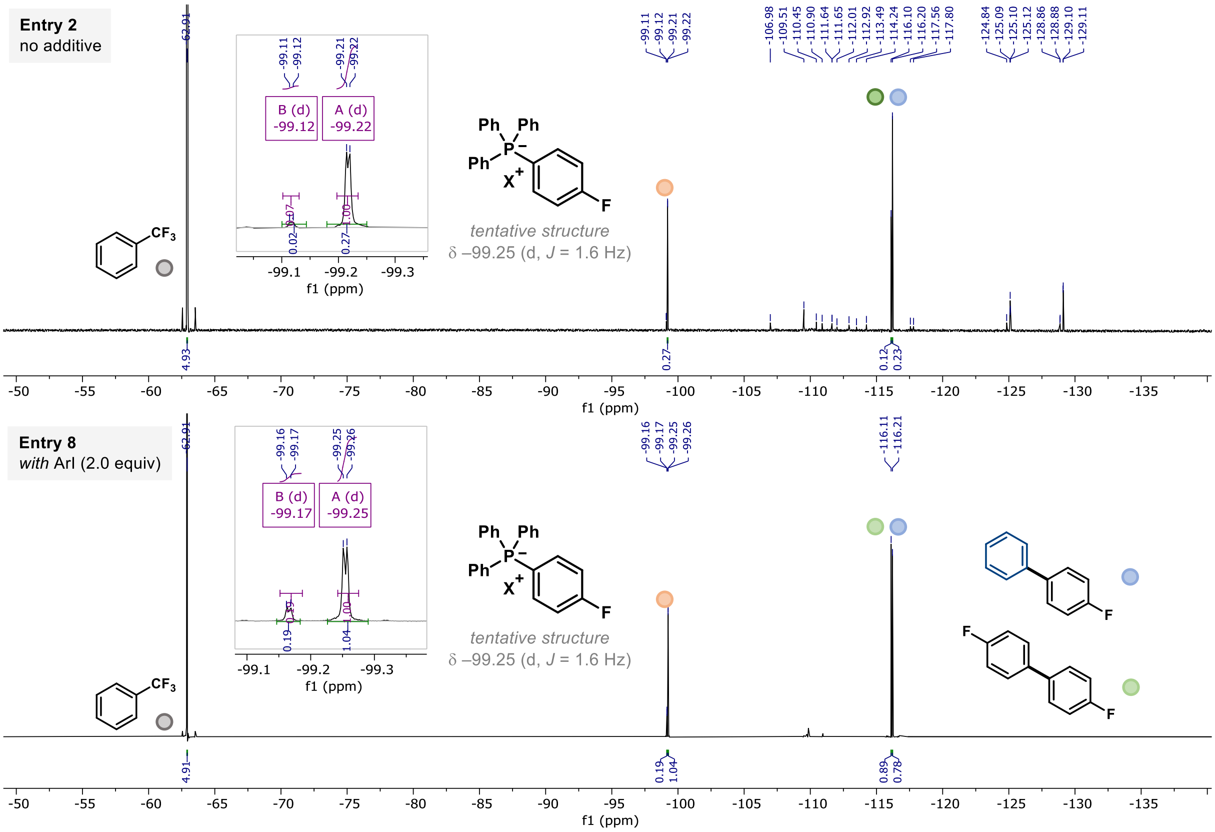

Figure S13. ^{19}F NMR spectra of reaction mixtures for entries 2 and 7

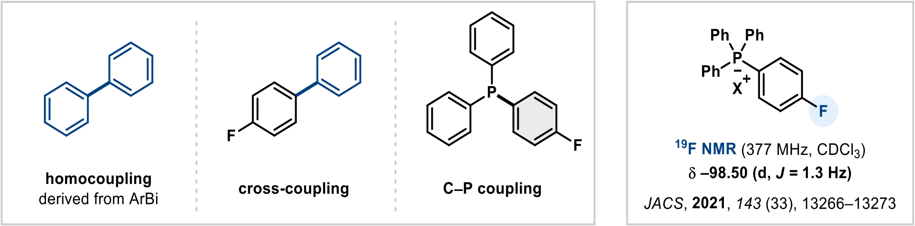

Figure S14. Structures of products observed by GC/MS and reported phosphonium salts for comparison[90]

Note: GC/MS analysis revealed a common species present in both the cross-coupled product, the Ph–Ph homocoupling product derived from the aryl-bismuth reagents, and an unexpected C–P coupling product.[91] Consistently, ^{19}F NMR analysis showed a signal near –99 ppm. Comparison with reported ^{19}F NMR data (377 MHz, $CDCl_3$, δ –98.50 (d, J = 1.3 Hz) for Ph_3P(4-F-Ph)Cl, reference 90) supports tentative assignment of this species as a phosphonium salt, likely formed via C–P cross-coupling between an aryl fragment and PPh_3 derived from Pd(PPh_3)$_4$. Related observations were also made in subsequent experiments examining oxidative addition between aryl-bismuth reagents and Pd(PPh_3)$_4$ (see Figure 16).

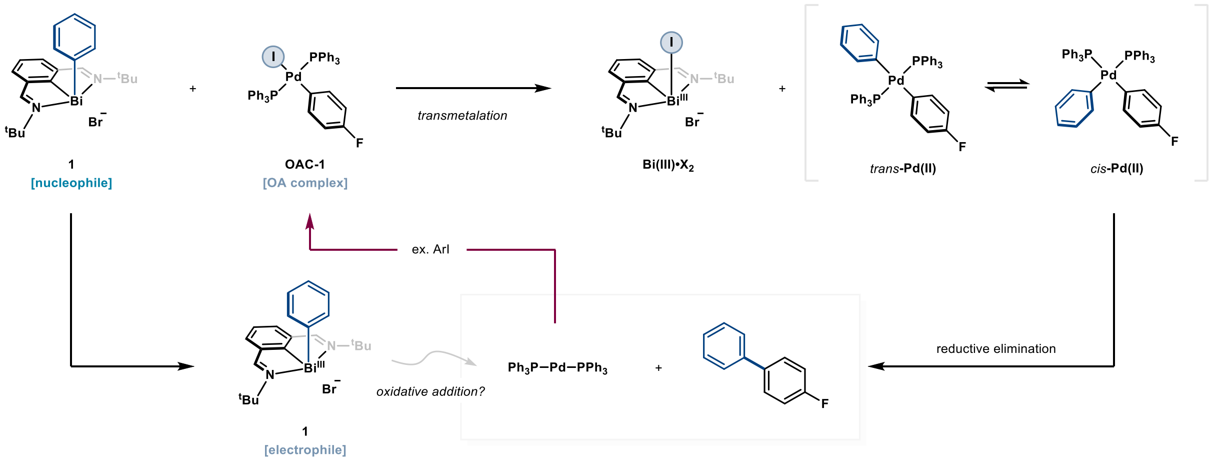

Figure S15. Plausible mechanism in stoichiometric experiment of **OAC-1** with aryl-bismuth

*Note: In stoichiometric reactions between aryl–bismuth and the oxidative addition complex **OAC-1**, the reactivity was markedly diminished compared to the catalytic process. The addition of 2.0 equiv. of aryl iodide, however, restored the reactivity, leading to product formation in yields comparable to the catalytic system (80%). These findings suggest competitive side reactions. Specifically, after transmetalation of aryl-bismuth **1** to **OAC-1**, the cross-coupled product is generated, with concomitant formation of Pd(PPh_3)$_2$. If aryl-bismuth also undergoes oxidative addition with Pd(PPh_3)$_2$ acting as the electrophile, the overall efficiency may be reduced. Based on this hypothesis, aryl-bismuth could act as an electrophile and undergo oxidative addition with palladium (see Figure S5). We subsequently carried out additional experiments to examine this possibility.*

Figure S16. Stoichiometric studies on the oxidative addition of ambiphilic aryl-bismuth

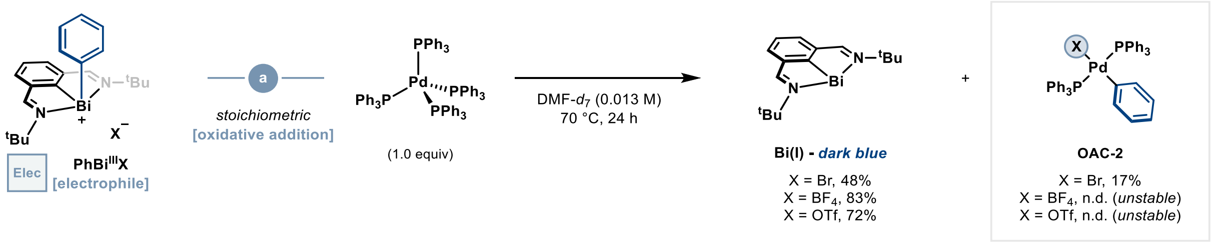

In an argon-filled glovebox, an oven-dried (80 °C, overnight) 10 mL thick-walled culture tube equipped with a Teflon-coated magnetic stir bar was charged with **ArBi(X)-1** (0.0200 mmol, 1.00 equiv.; **X** = **Br**, 12.2 mg; **X** = **BF_4**, 12.3 mg; **X** = **OTf**, 13.6 mg) and $Pd(PPh_3)_4$ (23.1 mg, 0.0200 mmol, 1.00 equiv.). Anhydrous DMF-d_7 (1.50 mL) was then added. The tube was sealed with a screw cap and removed from the glovebox. The mixture was stirred at 70 °C in a preheated heating block for 24 h. Upon completion, the yield was determined by ^1H NMR analysis using mesitylene (10.0 μL, 0.0719 mmol, 3.60 equiv.) as an internal standard.

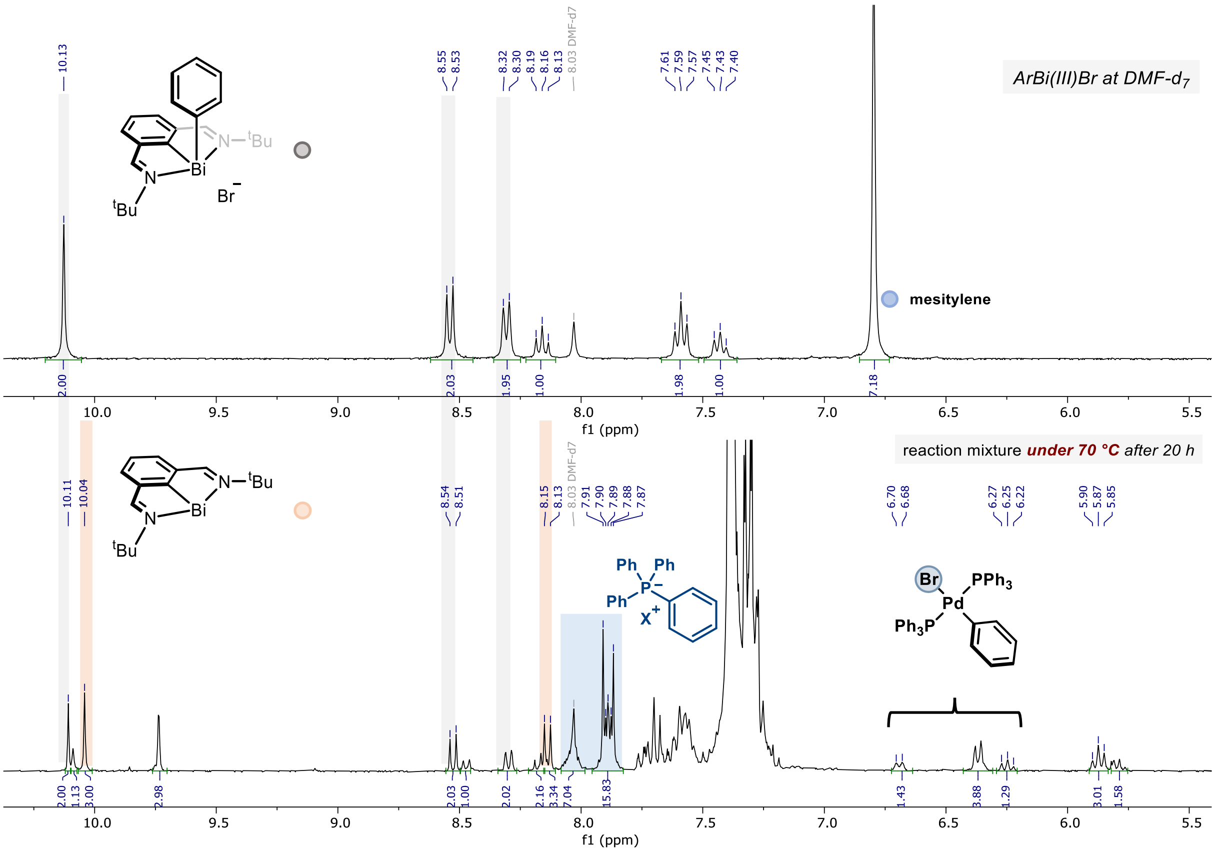

Figure S17. Oxidative addition complexes from the reaction of **ArBi(Br)-1** with $Pd(PPh_3)_4$

Note: See Figure S24 to S30 for details on the characterization of phosphonium salts.

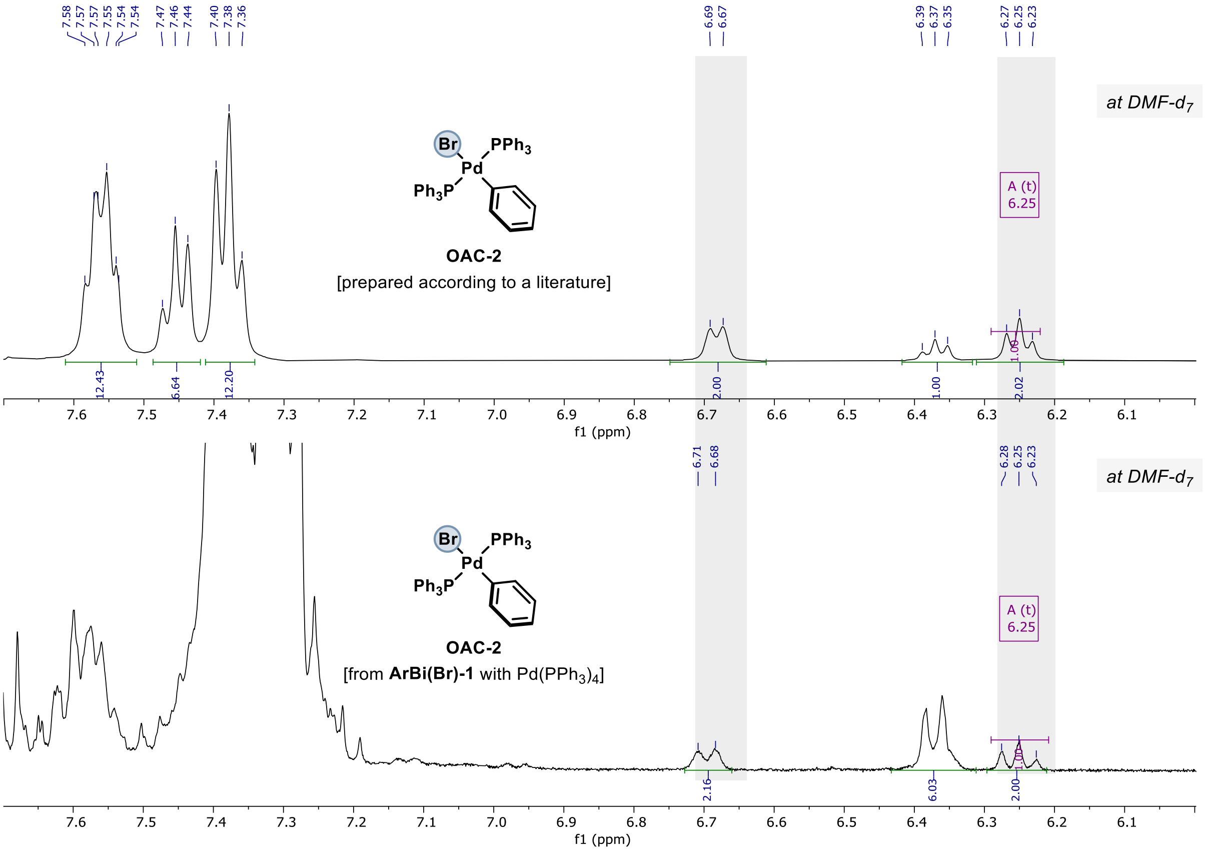

Figure S18. Independent synthesis of oxidative addition complex **OAC-2** and comparison of its ^{1}H NMR spectrum with that observed in the reaction of **ArBi(Br)-1** with $Pd(PPh_3)_4$

Note: **OAC-2** was synthesized separately according to the reported procedure[92], and the spectra was measured in DMF-d_7.

^{1}H NMR (400 MHz, DMF-d_7) δ 7.61 – 7.50 (m, 12H), 7.46 (t, J = 7.3 Hz, 3H), 7.41 – 7.32 (m, 12H), 6.73 – 6.62 (m, 2H), 6.37 (t, J = 7.2 Hz, 1H), 6.25 (t, J = 7.4 Hz, 2H) ppm.
^{13}C NMR (101 MHz, DMF-d_7) δ 159.9, 136.5, 135.0 (t, J = 6.3 Hz), 132.1 (t, J = 22.6 Hz), 130.3, 128.3 (t, J = 5.1 Hz), 127.9, 122.1 ppm.
^{31}P NMR (162 MHz, DMF-d_7) δ 23.93 ppm.

These spectra were then compared with that of the reaction mixture from the stoichiometric experiment of **ArBi(Br)-1** with $Pd(PPh_3)_4$.

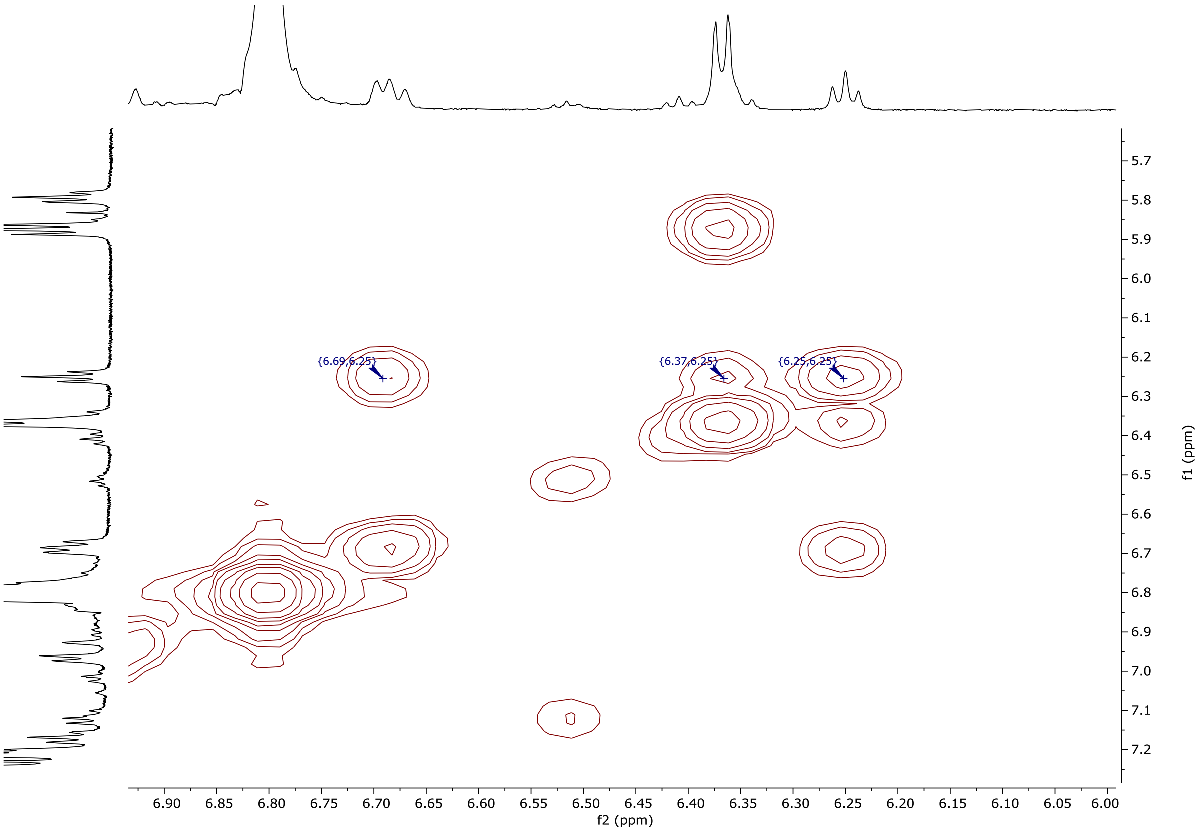

Figure S19. COSY spectrum of the reaction of **ArBi(Br)-1** with $Pd(PPh_3)_4$

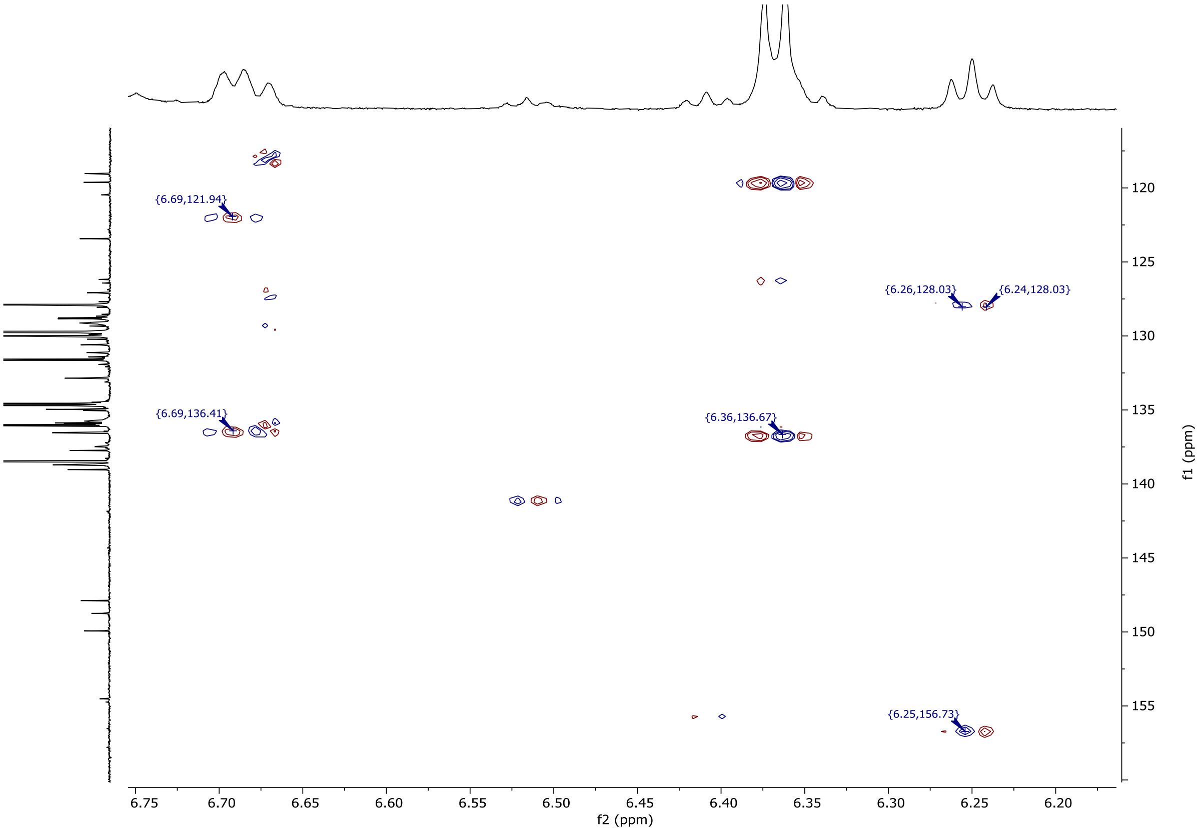

Figure S20. HMBC (^1H–^{13}C) spectrum of the reaction of **ArBi(Br)-1** with $Pd(PPh_3)_4$

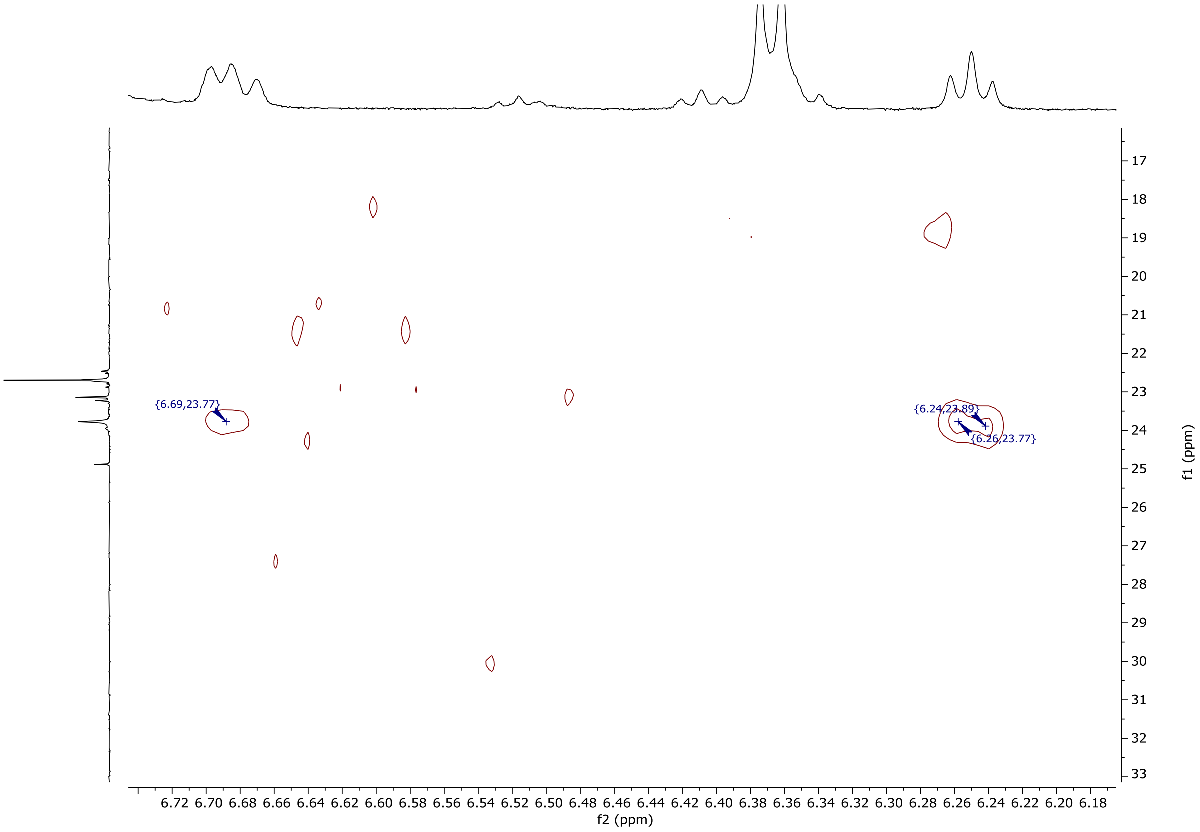

Figure S21. HMBC (^{1}H–^{31}P) spectrum of the reaction of **ArBi(Br)-1** with $Pd(PPh_3)_4$

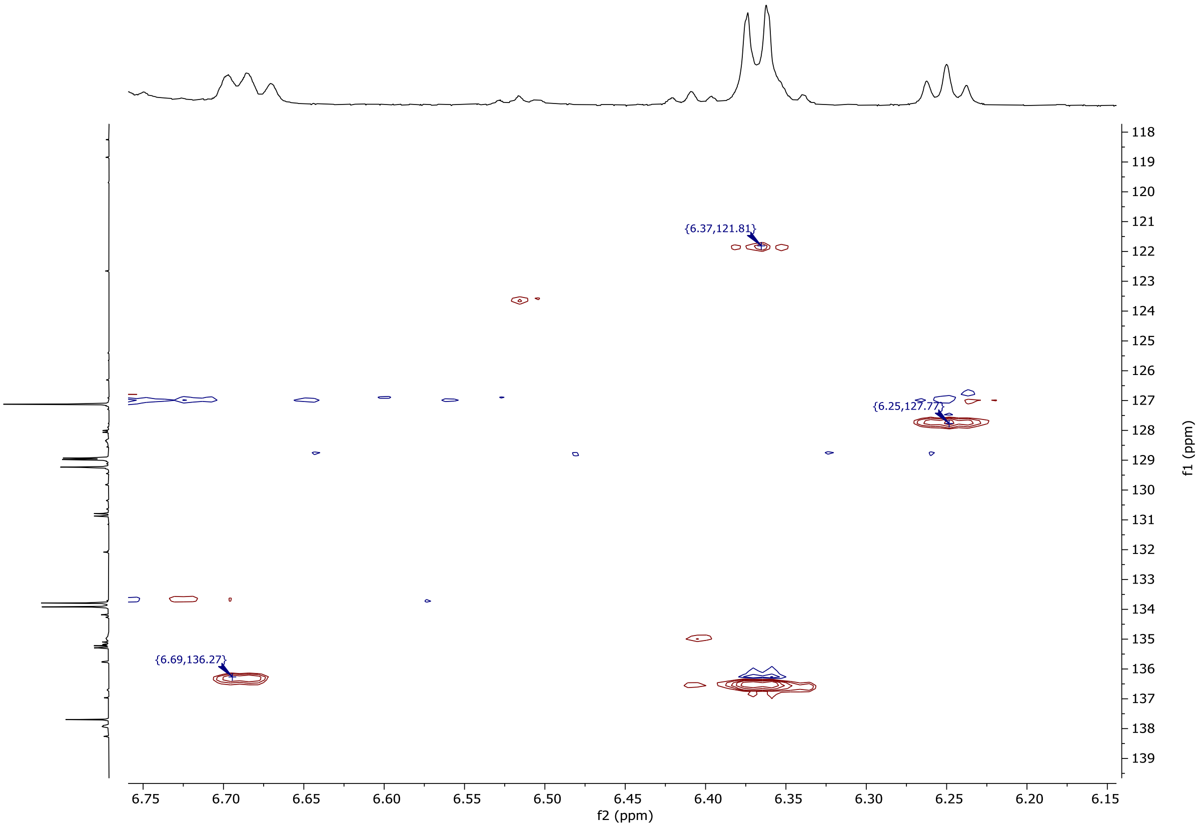

Figure S22. HSQC (^{1}H–^{13}C) spectrum of the reaction of **ArBi(Br)-1** with $Pd(PPh_3)_4$

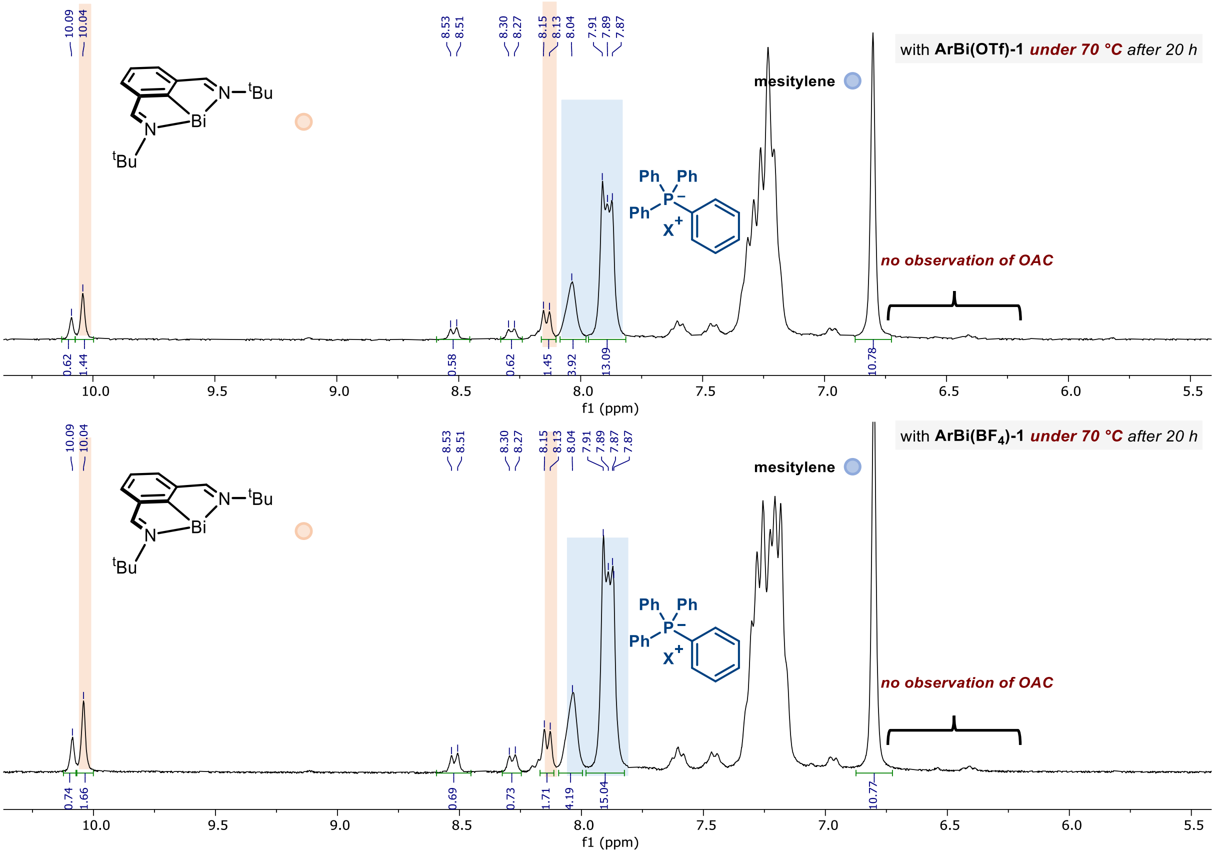

Figure S23. No oxidative addition complexes were observed from **ArBi(OTf)-1** or **ArBi(BF₄)-1** with Pd(PPh₃)₄

*Note: In contrast to the reaction with **ArBi(Br)-1**, no oxidative addition complexes were observed in the ^{1}H NMR spectra when **ArBi(OTf)-1** or **ArBi(BF₄)-1** were treated with Pd(PPh₃)₄, but N,C,N-pincer* bismuthinidene ***Bi(I)*** *species was detected as a side product. This outcome is likely due to the intrinsic instability of the cationic palladium species. To investigate this further, we performed additional experiments. As shown in Scheme S18, upon addition of extra equivalents of aryl borate, the desired cross-coupled product was obtained in high yield, indicating that the cationic OACs were indeed generated as intermediates and, owing to their high reactivity, rapidly underwent reaction with the aryl borate.*

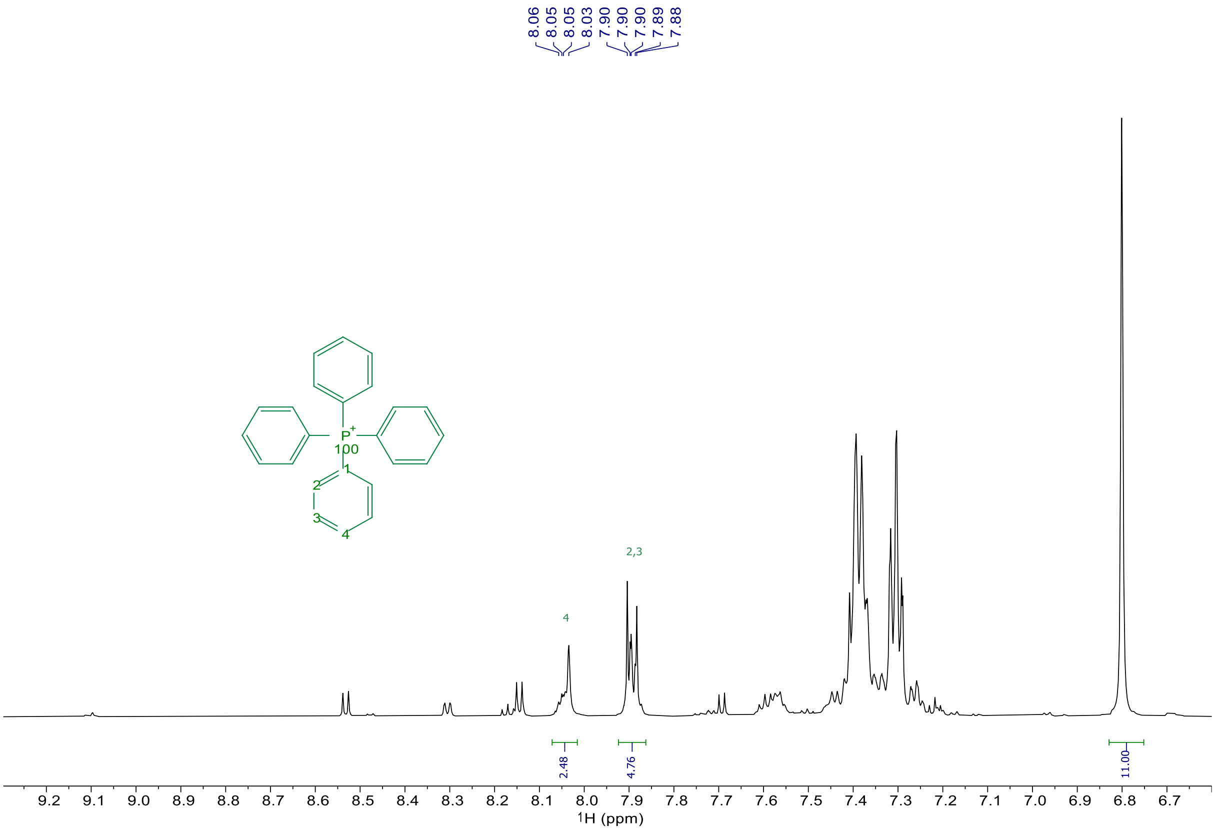

Figure S24. ^{1}H NMR spectrum of the phosphonium salt side product formed via C–P coupling

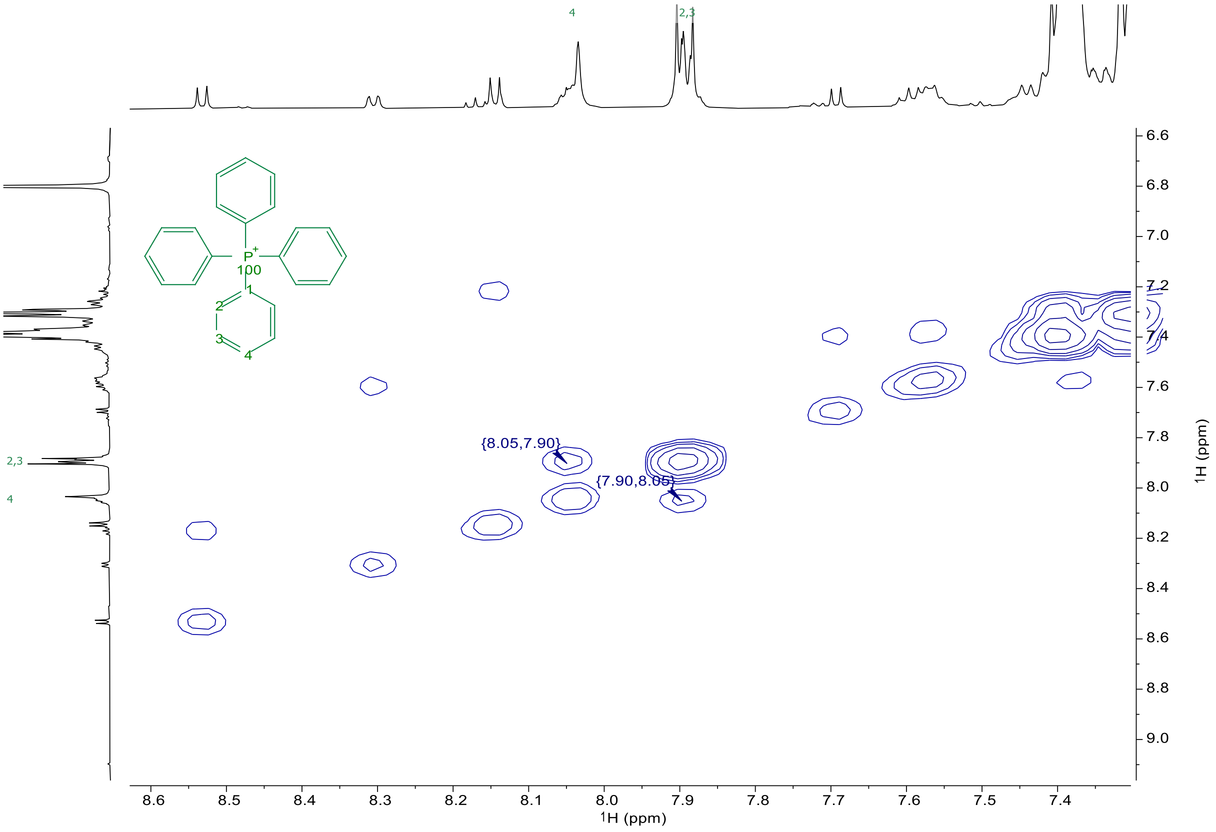

Figure S25. COSY spectrum of the phosphonium salt side product formed via C–P coupling

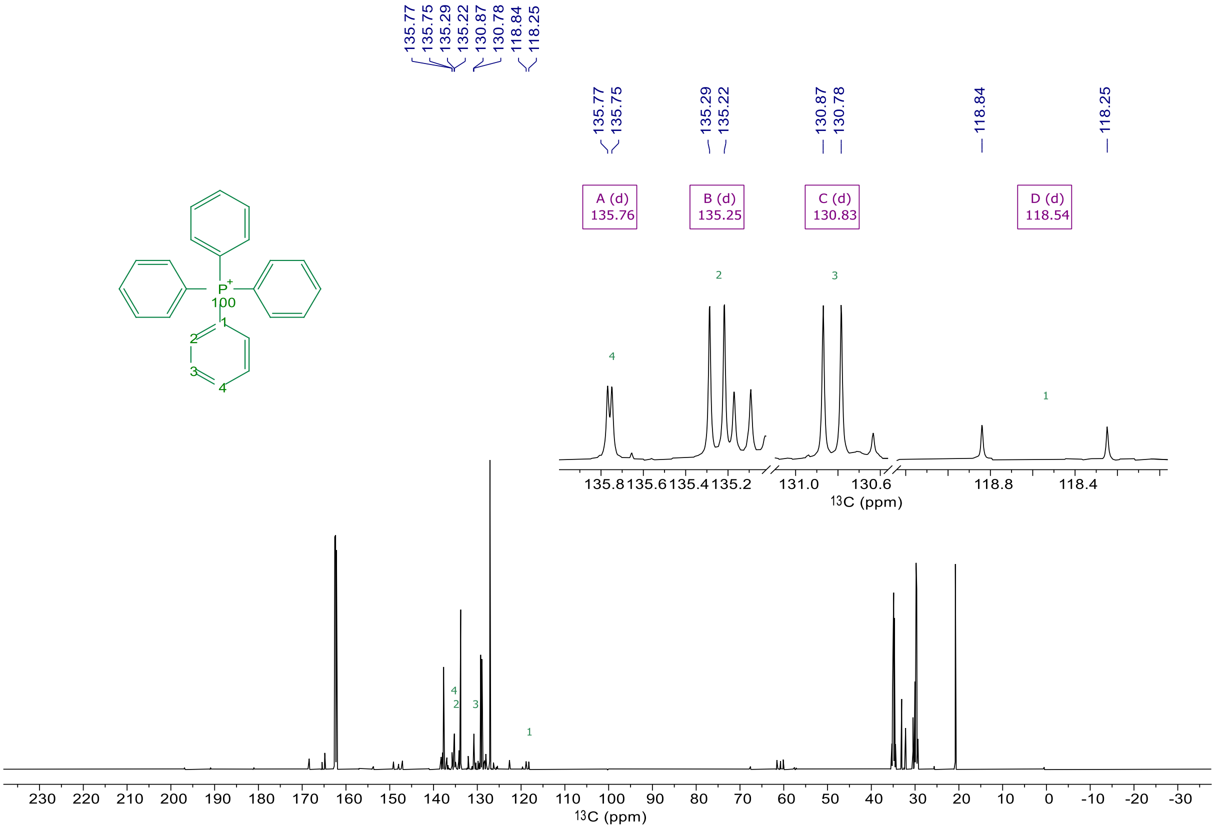

Figure S26. ^{13}C NMR spectrum of the phosphonium salt side product formed via C–P coupling

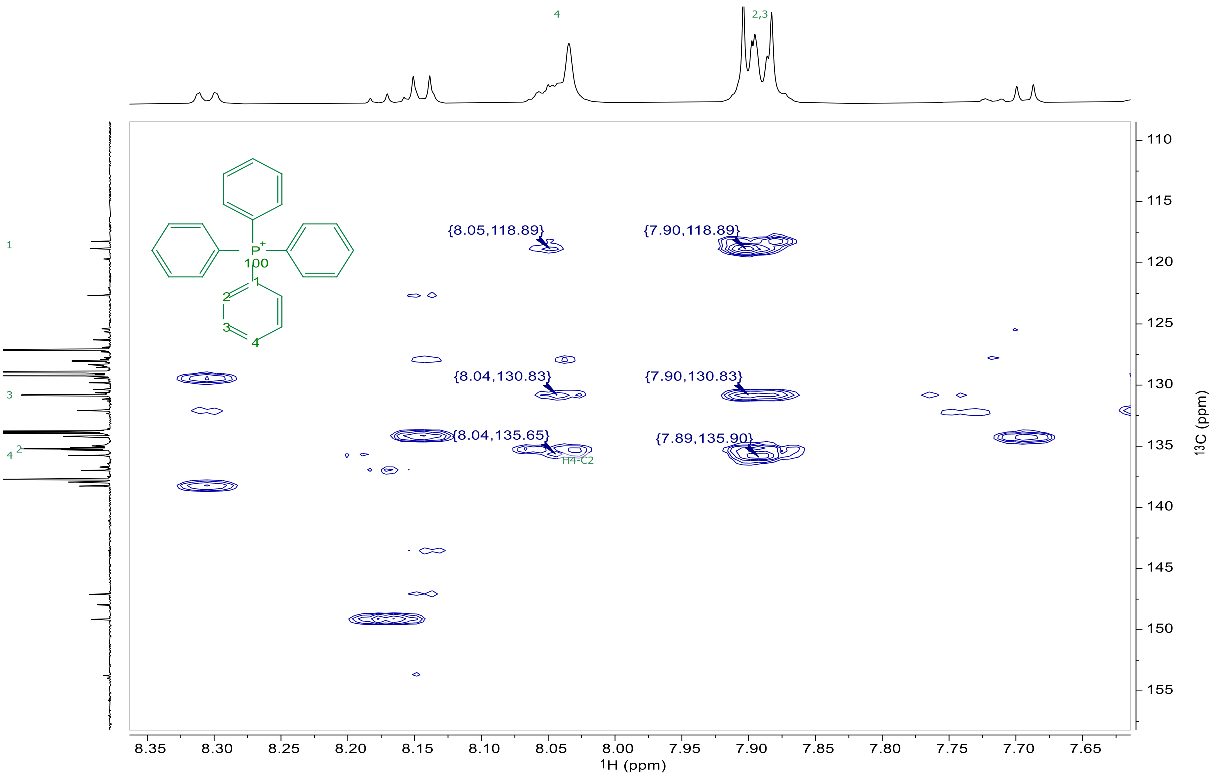

Figure S27. HMBC (^{1}H–^{13}C) spectrum of the phosphonium salt side product formed via C–P coupling

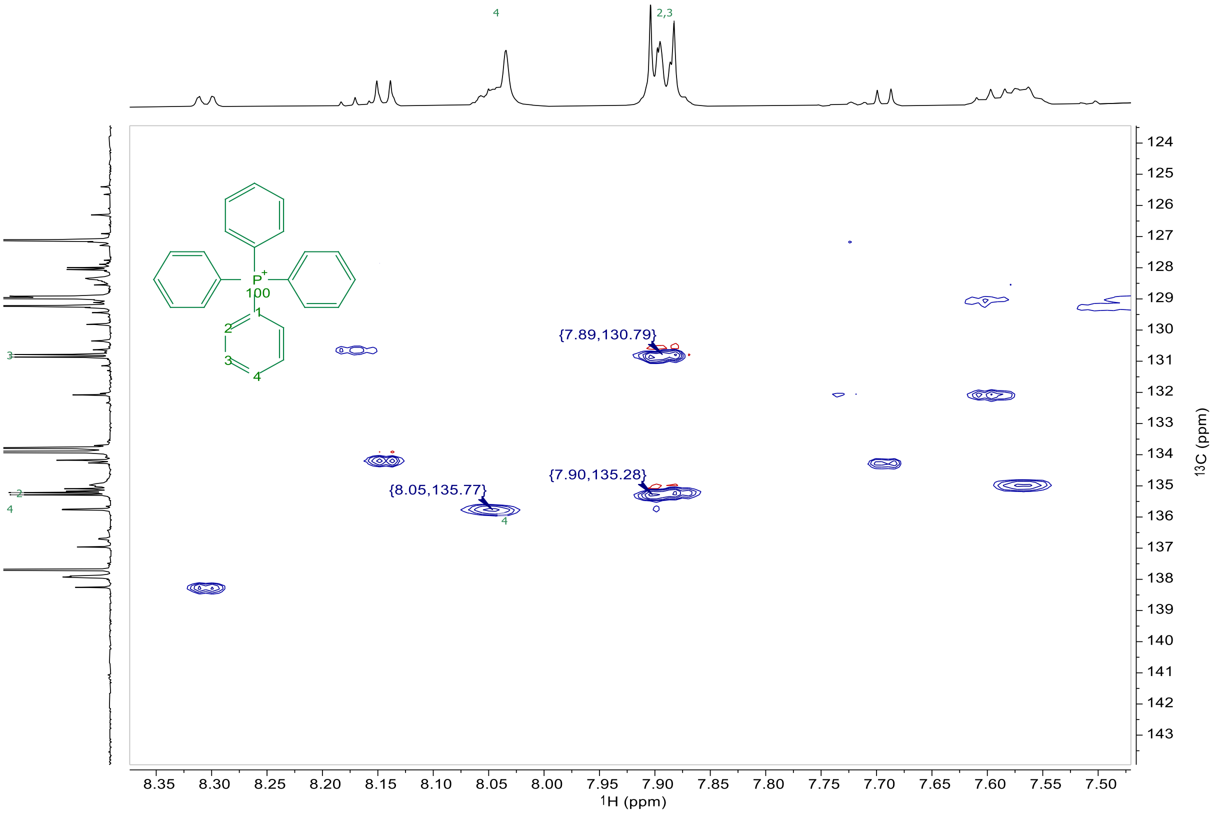

Figure S28. HSQC (^{1}H–^{13}C) spectrum of the phosphonium salt side product formed via C–P coupling

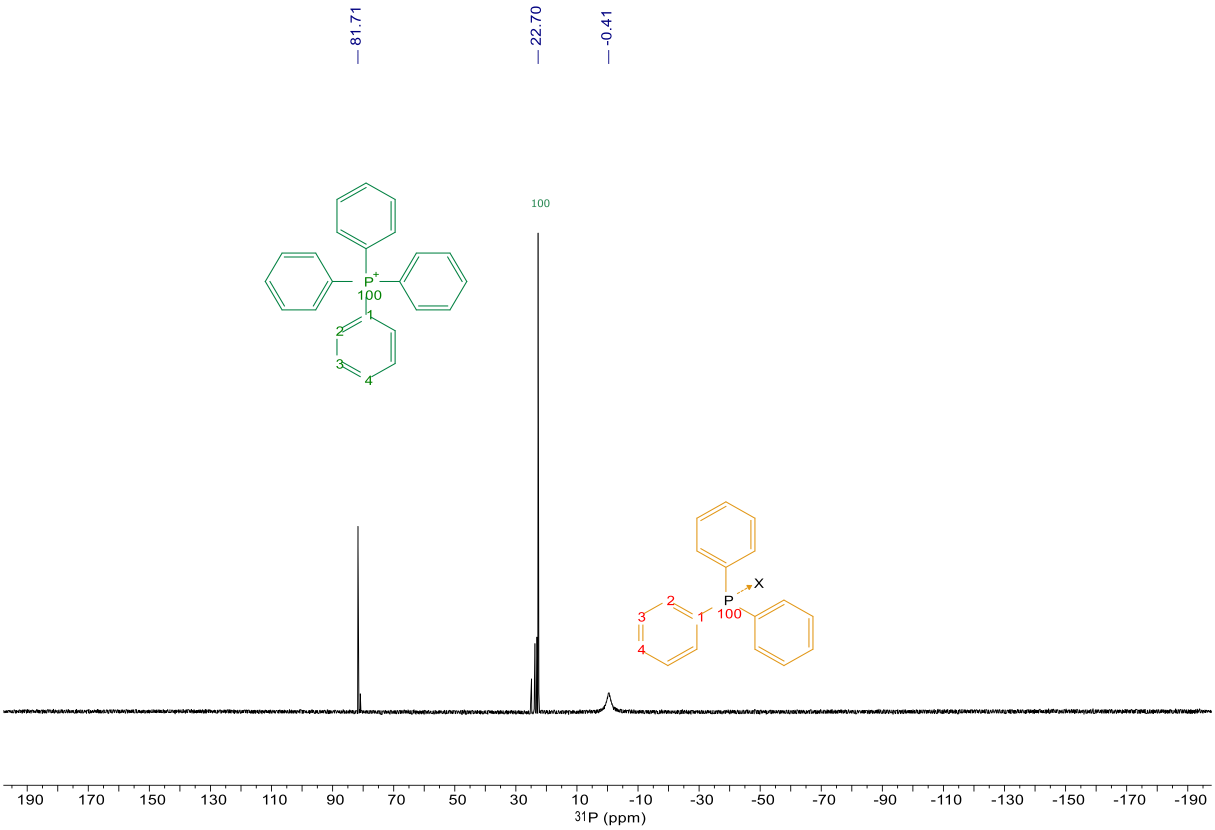

Figure S29. ^{31}P NMR spectrum of the phosphonium salt side product formed via C–P coupling

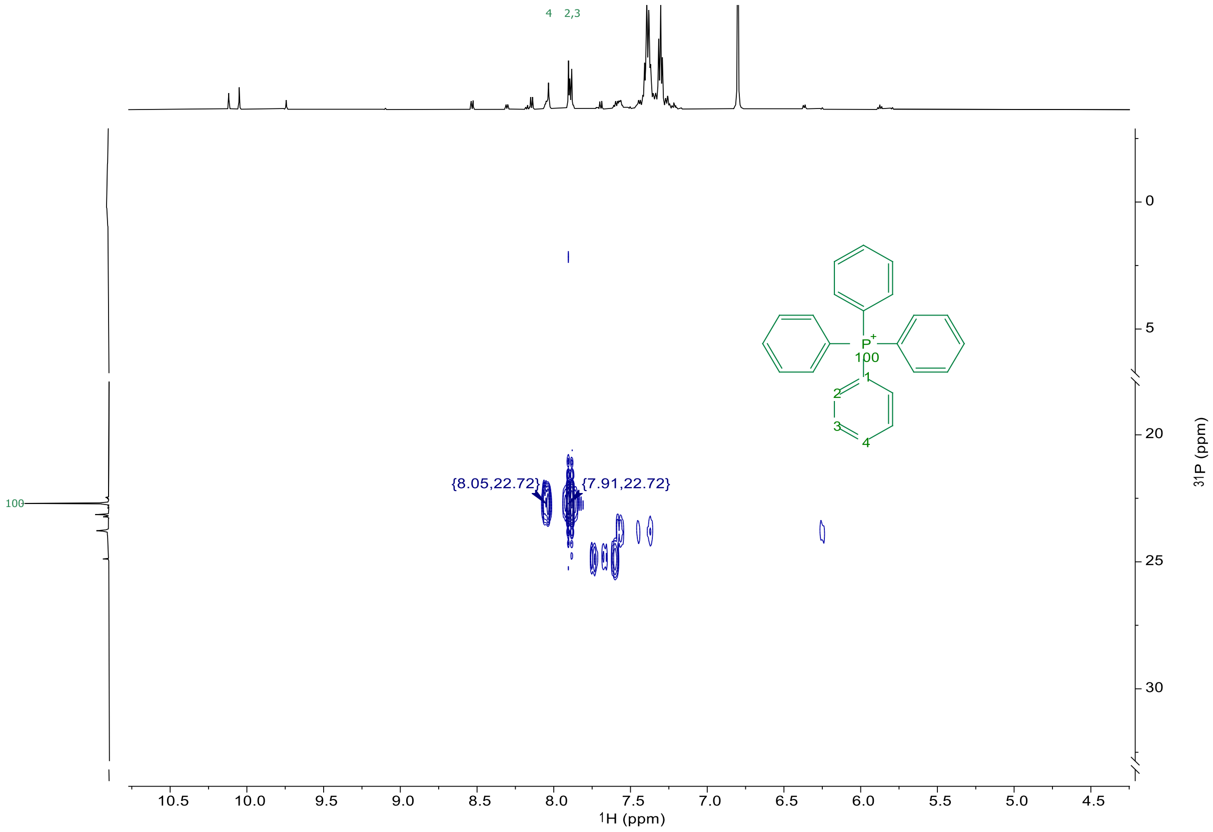

Figure S30. HMBC (^{1}H–^{31}P) spectrum of the phosphonium salt side product formed via C–P coupling

Figure S31. Stoichiometric experiment using **ArBi^{III}X** as the electrophile with stoichiometric Pd(PPh_3)$_4$

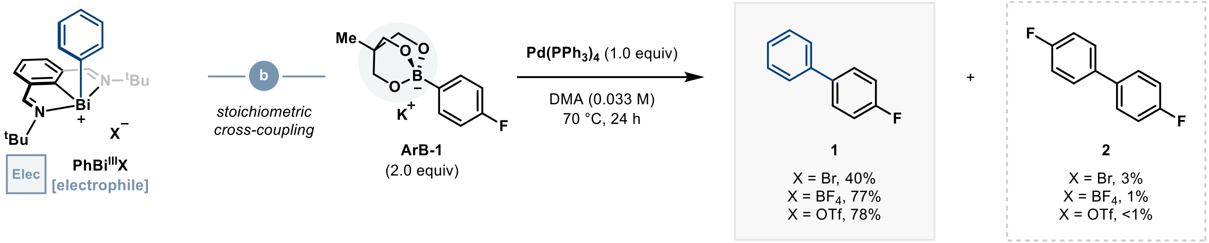

In an argon-filled glovebox, an oven-dried (80 °C, overnight) 10 mL thick-walled culture tube equipped with a Teflon-coated magnetic stir bar was charged with **ArBi(X)-1** (0.02 mmol, 1.0 equiv.; **X** = **Br**, 12.2 mg; **X** = **BF$_4$**, 12.3 mg; **X** = **OTf**, 13.6 mg), **ArB-1** (10.5 mg, 0.04 mmol, 2.0 equiv.) and Pd(PPh_3)$_4$ (23.1 mg, 0.02 mmol, 1.0 equiv.). Anhydrous DMF-d_7 (0.6 mL) was then added. The tube was sealed with a screw cap and removed from the glovebox. The mixture was stirred at 70 °C in a preheated heating block for 24 h. Upon completion, the yield was determined by ^{1}H NMR analysis using mesitylene (10.0 µL, 0.0719 mmol, 3.60 equiv.) as an internal standard.

7. Convergent Synthesis of Ambiphilic *N,C,N*-Pincer Aryl-Bismuth Compounds

Figure S32. Overview of the synthesis of ambiphiles

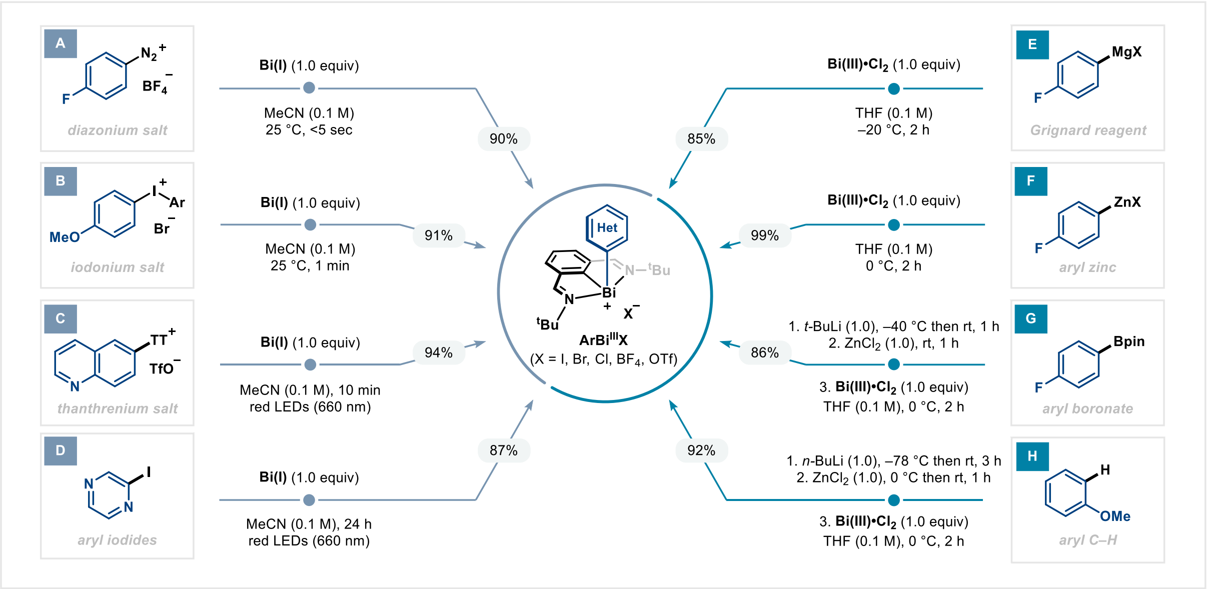

*Note: Aryl-bismuth complexes derived from the corresponding diazonium and iodonium salts were prepared with 1.0 equiv. of N,C,N-pincer bismuthinidene **Bi(I)** at room temperature, following the reported procedure (Path A and B).*[32] *Aryl-bismuth complexes derived from the corresponding thianthrenium salts and aryl iodides were prepared with 1.0 equiv. of N,C,N-pincer bismuthinidene **Bi(I)** under red LED irradiation (Path C and D); see GP-C and GP-E for details.*[32]

Path A: Synthesis of ayl-bismuth from the corresponding diazonium salt (Figure 5A, left)

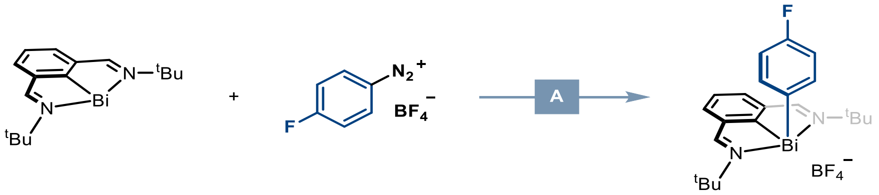

According to a reported literature procedure[32], the corresponding aryl-bismuth complex was obtained from *N,C,N*-pincer bismuthinidene **Bi(I)** (9.1 mg, 0.020 mmol, 1.0 equiv.) and 4-fluorophenyl diazonium tetrafluoroborate (4.6 mg, 0.022 mmol, 1.1 equiv.) in anhydrous MeCN-d_3 (0.6 mL), affording the product in 90% yield as determined by ^{1}H NMR. ^{1}H NMR (300 MHz, CD_3CN) δ 9.73 (s, 2H), 8.35 (d, J = 7.6 Hz, 2H), 8.15 – 8.03 (m, 3H), 7.26 – 7.17 (m, 2H), 1.32 (s, 18H) ppm. The characterization data were consistent with those previously reported (*26*).

Path B: Synthesis of aryl-bismuth from the corresponding iodonium salt (Figure 5A, left)

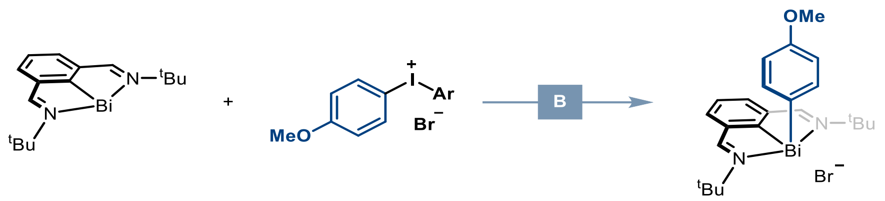

According to a reported procedure[32], the corresponding aryl-bismuth complex was obtained from *N*,*C*,*N*-pincer bismuthinidene **Bi(I)** (9.1 mg, 0.020 mmol, 1.0 equiv.) and bis(4-methoxyphenyl)iodonium bromide (12.6 mg, 0.0300 mmol, 1.50 equiv.) in anhydrous MeCN-d_3 (2.0 mL), affording the product in 91% yield as determined by ^{1}H NMR. The characterization data were consistent with those of **ArBi(Br)-2**.

Path C: Synthesis of aryl-bismuth from the corresponding thianthrenium salt (Figure 5A, left)

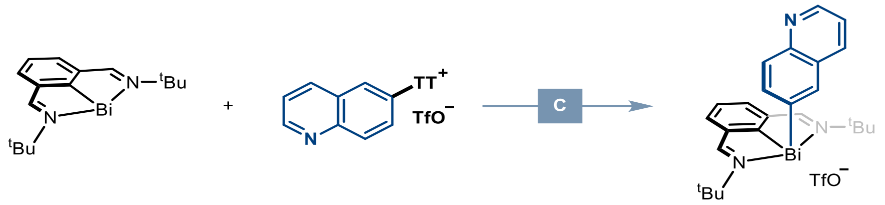

Following *General Procedure E*, the product was obtained from *N*,*C*,*N*-pincer bismuthinidene **Bi(I)** (1.0 equiv.) and thianthrenium salt **TT-5** (1.1 equiv.). For details, see the synthesis of **ArBi(OTf)-6**.

Path D: Synthesis of aryl-bismuth from the corresponding aryl iodide (Figure 5A, left)

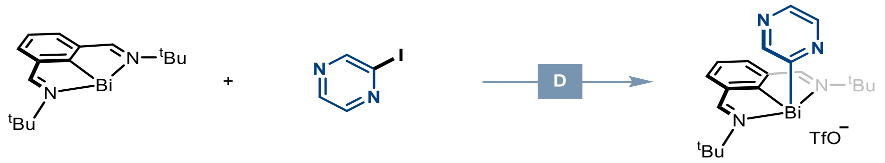

Following *General Procedure C*, the product was obtained from *N*,*C*,*N*-pincer bismuthinidene **Bi(I)** (1.0 equiv.) and aryl iodide (2.0 equiv.). For details, see the synthesis of **ArBi(I)-2.**

Path E, F and G: Synthesis of aryl-bismuth from the corresponding aryl Grignard reagent or aryl zinc reagent (Figure 5A, right)

[(2,6-(*t*BuNCH)$_2$C$_6$H$_3$)Bi(4-fluorophenyl)(Cl)], ArBi(Cl)-1

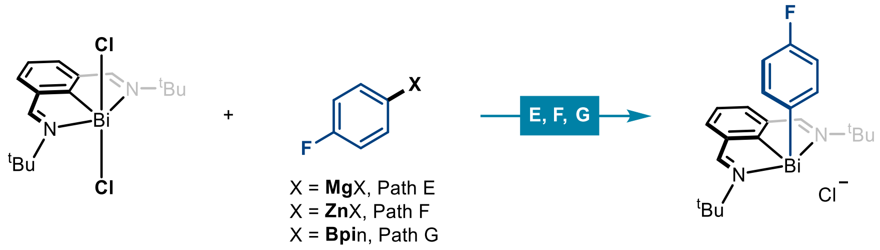

From aryl boronic ester: *The aryl zinc chloride solution was prepared as follows:* *t*BuLi (1.7 M in pentane, 0.5 mmol, 1.0 equiv.) was added to a solution of 2-(4-fluorophenyl)-4,4,5,5-tetramethyl-1,3,2-dioxaborolane (111 mg, 0.5 mmol, 1.0 equiv.) in THF (1.0 ml, 0.5 M) at −40 °C under Ar. The reaction mixture was stirred at this temperature for a further 30 mins after which the reaction mixture was warmed to room temperature and stirred for a further 1 h. A solution of ZnCl$_2$ (0.75 ml, 0.7 M in THF, 0.53 mmol, 1.05 equiv.) was then added and the reaction was stirred at room temperature for 1.5 h. The arylzinc solution was titrated using I$_2$ according to Knochel's method[93] and the concentration was determined to be 0.18 M.

An aliquot of the arylzinc reagent (0.56 mL, 0.18 M, 0.10 mmol, 1.0 equiv.,) was added dropwise to a stirred solution of *N*,*C*,*N*-pincer bismuthinidene **Bi(III)·Cl$_2$** (0.10 mmol, 1.0 equiv., 52.3 mg) in THF (1.0 mL, 0.1 M) at 0 °C under an argon atmosphere and stirred for 2 h. The reaction mixture was then quenched with aq. sat. NH$_4$Cl solution and extracted with CH$_2$Cl$_2$. The combined organic fraction was dried over anhydrous MgSO$_4$, filtered and concentrated under reduced pressure. Trituration of the mixture with Et$_2$O gives the product as a white solid (50.3 mg, 86% yield).

From aryl zinc reagent: *The aryl zinc chloride solution was prepared as follows:* 1-fluoro-4-iodobenzene (0.28 mL, 2.0 mmol, 1.0 equiv.) was added to a stirred solution of *i*PrMgCl·LiCl (1.7 mL, 1.3 M in THF, 2.2 mmol, 1.1 equiv.) at 0 °C under an argon atmosphere and stirred for 1 h. A solution of ZnCl$_2$ (3.1 mL, 0.7 M in THF, 2.2 mmol, 1.1 equiv.) was added, and the reaction was allowed to warm to room temperature and subsequently stirred for a further 1 h. The aryl zinc solution was titrated using I$_2$ according to Knochel's method[93], and the concentration was determined to be 0.38 M.

An aliquot of the aryl zinc reagent (0.79 mL, 0.38 M, 0.30 mmol, 1.0 equiv.) was added dropwise to a stirred solution of *N*,*C*,*N*-pincer bismuthinidene **Bi(III)·Cl$_2$** (157 mg, 0.300 mmol, 1.00 equiv.) in THF (3.0 mL, 0.1 M) at 0 °C under an argon atmosphere, and the mixture was stirred for 2 h. The reaction mixture was then quenched with aq. sat. NH$_4$Cl solution and extracted with CH$_2$Cl$_2$. The combined organic fraction was dried over anhydrous MgSO$_4$, filtered and concentrated under reduced pressure to give the product as a pale yellow solid (173 mg, 99% yield).

From Grignard reagent: *The aryl Grignard solution was prepared as follows:* 1-fluoro-4-iodobenzene (58 µL, 0.50 mmol, 1.0 equiv.) was added to a stirred solution of *i*PrMgCl·LiCl (0.42 mL, 1.3 M in THF, 0.55 mmol, 1.1 equiv.) at 0 °C under an argon atmosphere, and the mixture was stirred for 1 h. This solution was then diluted with THF (0.58 mL) to generate a 0.5 M solution.

An aliquot of the aryl Grignard reagent (0.20 mL, 0.10 mmol, 1.0 equiv.) was added dropwise to a stirred solution of *N*,*C*,*N*-pincer bismuthinidene **Bi(III)·Cl$_2$** (52.3 mg, 0.100 mmol, 1.00 equiv.) in THF (1.0 mL, 0.1 M) at −20 °C under an argon atmosphere, and the mixture was stirred for 2 h. The reaction mixture was then quenched with brine and extracted thrice with CH$_2$Cl$_2$. The combined organic fractions were dried over anhydrous Na$_2$SO$_4$, filtered, and concentrated under reduced pressure. ^{1}H NMR analysis of the reaction mixture revealed an 85% yield.

^{1}H NMR (400 MHz, CD$_3$CN) δ 9.75 (s, 2H), 8.35 (d, *J* = 7.6 Hz, 2H), 8.14 – 8.03 (m, 3H), 7.23 – 7.12 (m, 2H), 1.30 (s, 18H) ppm.

^{13}**C NMR** (101 MHz, CD_3CN) δ 188.5, 174.9, 168.6, 164.0 (d, *J* = 247.4 Hz), 149.4, 141.5 (d, *J* = 7.6 Hz), 137.6, 131.6, 120.1 (d, *J* = 20.5 Hz), 62.3, 31.0 ppm.
^{19}**F{^{1}H} NMR** (282 MHz, $CDCl_3$) δ −111.40 ppm.
HRMS (ESI) calculated for $C_{22}H_{27}BiFN_2$ [M–Cl]$^+$: 547.19568, found: 547.19537.

[(2,6-(^{t}BuNCH)$_2$C$_6$H$_3$)Bi(4-methoxyphenyl)(Cl)], ArBi(Cl)-2

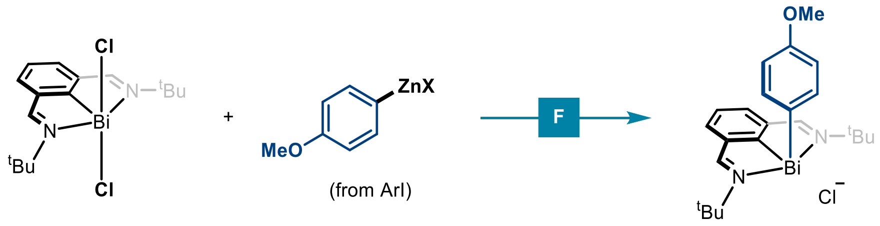

From aryl zinc reagent*: The aryl zinc chloride solution was prepared as follows:* a solution of *i*PrMgCl·LiCl (1.7 mL, 1.3 M in THF, 2.2 mmol, 1.1 equiv.) was added to a solution of 4-iodoanisole (444 mg, 2.0 mmol, 1.0 equiv.) in anhydrous THF (2 mL. 1.0 M) at 0 °C under an argon atmosphere. The reaction was stirred for 1 h at the same temperature, and then a solution of $ZnCl_2$ (4.4 mL, 0.5 M in THF, 2.2 mmol, 1.1 equiv.) was added, and the reaction was allowed to warm to room temperature and subsequently stirred for a further 30 min. The aryl zinc solution was titrated using I_2 according to Knochel's method[93], and the concentration was determined to be 0.15 M.

An aliquot of the aryl zinc reagent (3.3 mL, 0.15 M, 0.50 mmol, 1.0 equiv.) was added dropwise to a stirred solution of *N*,*C*,*N*-pincer bismuthinidene **Bi(III)·Cl$_2$** (261.6 mg, 0.500 mmol, 1.0 equiv.) in THF (5.0 mL, 0.1 M) at 0 °C under an argon atmosphere, and the mixture was stirred at that temperature for 2 h. The reaction mixture was then quenched with sat. aq. NH_4Cl solution and extracted with CH_2Cl_2. The combined organic fractions were dried over anhydrous $MgSO_4$, filtered, and concentrated under reduced pressure to ~10 mL. The product was precipitated out of solution with MTBE and isolated by filtration to give the product as a white solid (290 mg, 98% yield).

^{1}**H NMR** (400 MHz, CD_2Cl_2) δ 9.73 (s, 2H), 8.43 (d, *J* = 7.6 Hz, 2H), 8.09 (t, *J* = 7.6 Hz, 1H), 7.96 – 7.90 (m, 2H), 7.01 – 6.93 (m, 2H), 3.72 (s, 3H), 1.34 (s, 18H) ppm.
^{13}**C NMR** (101 MHz, CD_2Cl_2) δ 185.8, 168.7, 167.5, 160.9, 148.4, 140.0, 137.4, 131.4, 118.3, 61.9, 55.5, 31.3 ppm.
HRMS (ESI) calculated for $C_{23}H_{30}BiN_2O$ [M–Cl]$^+$: 559.2157, found: 559.2165.

[(2,6-(^{t}BuNCH)$_2$C$_6$H$_3$)Bi(4-trifluoromethylphenyl)(I)], ArBi(I)-1

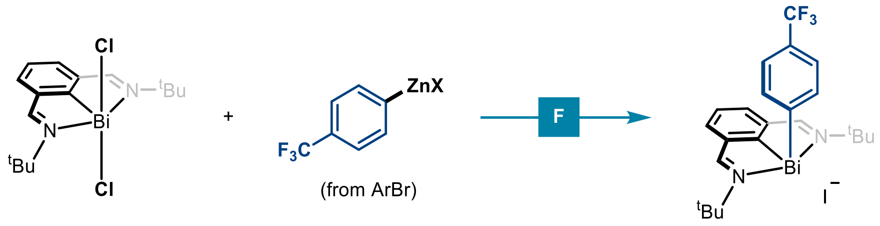

From aryl zinc reagent*: The aryl zinc chloride solution was prepared as follows:* *n*-BuLi (1.37 mL, 2.35 M in hexanes, 2.86 mmol, 1.0 equiv.) was added to a stirred solution of 4-bromobenzotrifluoride (0.40 mL, 2.86

mmol, 1.0 equiv.) in anhydrous THF (2.0 mL, 1.5 M) at −78 °C under an argon atmosphere. The reaction was subsequently stirred for 15 min, after which a solution of $ZnCl_2$ (4.1 mL, 0.7 M in THF, 2.86 mmol, 1.0 equiv.) was added at the same temperature, and the reaction was allowed to warm to ambient temperature (~20 °C) and subsequently stirred for a further 40 min. The aryl zinc solution was titrated using I_2 according to Knochel's method[93], and the concentration was determined to be 0.31 M.

An aliquot of the aryl zinc reagent (1.51 mL, 0.33 M, 0.525 mmol, 1.05 equiv.) was added dropwise to a stirred solution of *N*,*C*,*N*-pincer bismuthinidene **Bi(III)·Cl₂** (261.6 mg, 0.500 mmol, 1.0 equiv.) in THF (3.0 mL, 0.17 M) at room temperature under an argon atmosphere, and the mixture was stirred at that temperature for a further 4 h. The reaction mixture was then quenched with an aq. 2 M NaI solution and extracted with CH_2Cl_2. The combined organic fractions were dried over anhydrous $MgSO_4$, filtered and concentrated under reduced pressure to ~10 mL. The product was precipitated out of solution with MTBE and isolated by filtration to give a yellow solid, which was then dissolved in MeCN (2 mL) and filtered. The filtrate was then concentrated under reduced pressure to give the product as a yellow solid (348 mg, 96% yield).

*Spectral data consistent with that disclosed earlier in this report (**ArBi(I)-1**).*

Path H: Synthesis of aryl-bismuth from anisole (Figure 5A, right)

[(2,6-(^{t}BuNCH)$_2$C$_6$H$_3$)Bi(2-methoxyphenyl)(Cl)], ArBi(Cl)-3

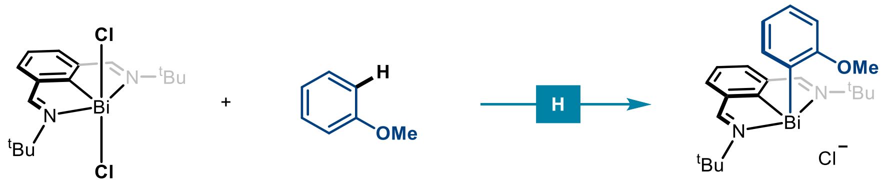

The aryl zinc chloride solution was prepared as follows: A solution of *n*-BuLi (0.5 mL, 2.0 M in hexanes, 1.0 mmol, 1.0 equiv.) was added dropwise to a stirred solution of anisole (0.12 mL, 1.1 mmol, 1.1 equiv.) in THF (0.75 mL, 1.3 M) at –78 °C under an argon atmosphere. The reaction mixture was then warmed to room temperature and stirred for a further 3 h, after which it was cooled to 0 °C and a solution of $ZnCl_2$ (2.0 mL, 0.5 M in THF, 1.0 mmol, 1.0 equiv.) was added. The reaction mixture was then warmed back to room temperature and stirred for a further 1 h. The aryl zinc solution was titrated using I_2 according to Knochel's method[93], and the concentration was determined to be 0.27 M.

An aliquot of the aryl zinc reagent (0.37 mL, 0.10 mmol, 1.0 equiv.) was added dropwise to a stirred solution of *N*,*C*,*N*-pincer bismuthinidene **Bi(III)·Cl₂** (52.3 mg, 0.100 mmol, 1.00 equiv.) in THF (1.0 mL, 0.1 M) at 0 °C under an argon atmosphere, and the mixture was stirred for 2 h. The reaction mixture was then quenched with sat. aq. NH_4Cl and extracted thrice with CH_2Cl_2. The combined organic fractions were dried over anhydrous Na_2SO_4, filtered, and concentrated under reduced pressure. ^{1}H NMR analysis of the reaction mixture revealed a 91% yield. Trituration of the mixture with Et_2O gave the product, together with 2% **Bi(III)·Cl₂** and 8% of ***n*BuBi(III)·Cl**, as a white solid (54.8 mg, 92% yield).

^{1}H NMR (400 MHz, CD_3CN) δ 9.72 (s, 1H), 8.33 (d, *J* = 7.5 Hz, 2H), 8.05 (t, *J* = 7.6 Hz, 1H), 7.63 – 7.55 (m, 1H), 7.45 – 7.38 (m, 2H), 6.92 (ddd, *J* = 8.1, 6.2, 2.3 Hz, 1H), 3.98 (s, 3H), 1.28 (s, 18H) ppm.

^{13}C NMR (101 MHz, CD_3CN) δ 186.8, 168.4, 164.8, 162.6, 149.7, 137.9, 137.4, 132.7, 131.5, 126.6, 112.1, 62.0, 56.5, 30.8 ppm.

HRMS (ESI) calculated for $C_{23}H_{30}BiN_2O$ $[M–Cl]^+$: 559.2157, found: 559.2153.

[(2,6-(^{t}BuNCH)$_2$C$_6$H$_3$)Bi(2-methoxyphenyl)(Cl)], ArBi(Cl)-3

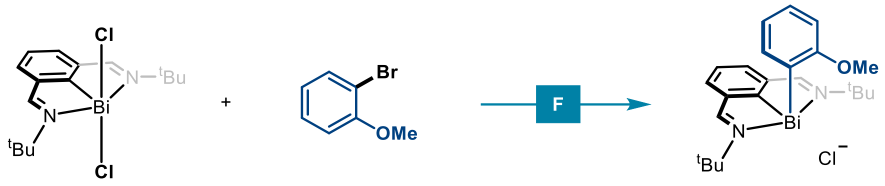

The aryl zinc chloride solution was prepared as follows: n-BuLi (1.37 mL, 2.35 M in hexanes, 3.21 mmol, 1.0 equiv.) was added to a stirred solution of 2-bromoanisole (0.40 mL, 3.21 mmol, 1.0 equiv.) in anhydrous THF (2.0 mL, 1.76 M) at –78 °C under an argon atmosphere. The reaction was subsequently stirred for 15 min, after which a solution of $ZnCl_2$ (4.6 mL, 0.7 M in THF, 3.21 mmol, 1.0 equiv.) was added at the same temperature, and the reaction was allowed to warm to ambient temperature (~20 °C) and subsequently stirred for a further 40 min. The aryl zinc solution was titrated using I_2 according to Knochel's method[48], and the concentration was determined to be 0.33 M.

An aliquot of the aryl zinc reagent (1.51 mL, 0.33 M, 0.50 mmol, 1.0 equiv.) was added dropwise to a stirred solution of *N*,*C*,*N*-pincer bismuthinidene **Bi(III)·Cl$_2$** (261.6 mg, 0.500 mmol, 1.0 equiv.) in THF (5.0 mL, 0.1 M) at 0 °C under an argon atmosphere, and the mixture was stirred at that temperature for 2 h. The reaction mixture was then quenched with sat. aq. NH_4Cl solution and extracted with CH_2Cl_2. The combined organic fractions were dried over anhydrous $MgSO_4$, filtered, and concentrated under reduced pressure to ~10 mL. The product was precipitated out of solution with MTBE and isolated by filtration to give the product as a white solid (282 mg, 95% yield).

^{1}H NMR (400 MHz, CD_2Cl_2) δ 9.79 (d, *J* = 2.0 Hz, 2H), 8.48 (dd, *J* = 7.6, 1.5 Hz, 2H), 8.09 (td, *J* = 7.6, 2.0 Hz, 1H), 7.63 (d, *J* = 7.2 Hz, 1H), 7.43 – 7.30 (m, 2H), 6.91 (t, *J* = 7.2 Hz, 1H), 3.99 (s, 3H), 1.31 (s, 18H) ppm.

^{13}C NMR (101 MHz, CD_2Cl_2) δ 184.9, 167.8, 163.2, 161.7, 148.9, 137.6, 137.5, 132.0, 131.5, 126.3, 111.1, 61.6, 56.1, 30.9 ppm.

HRMS (ESI) calculated for $C_{23}H_{30}BiN_2O$ [M–Cl]$^+$: 559.2157, found: 559.2153.

8. References

1. de Meijere, A., Diederich, F. Eds., *Metal-Catalyzed Cross-Coupling Reactions*. (Wiley-VCH, Weinheim, Germany, 2004).
2. Brown, D.G., Boström, J. *J. Med. Chem.* **59**, 4443–4458 (2016).
3. de Meijere, A., Bräse, S., Oestreich, M. Eds., *Metal-Catalyzed Cross-Coupling Reactions and More* (Wiley-VCH, Weinheim, Germany, 2013).
4. Johansson Seechurn, C. C. C., Kitching, M. O., Colacot, T. J., Snieckus, V. *Angew. Chem. Int. Ed.* **51**, 5062–5085 (2012).
5. Johansson Seechurn, C. C. C., DeAngelis, A., Colacot, T. J. *New Trends in Cross-Coupling: Theory and Applications*, T. J. Colacot, Ed. (RSC, Cambridge, 2014).
6. Fricke, C., Schoenebeck, F. *Acc. Chem. Res.* **53**, 2715–2725 (2020).
7. Denmark, S. E., Ambrosi, A. *Org. Process Res. Dev.* **19**, 982–994 (2015).
8. Yu, D.-G., Li, B.-J., Shi, Z.-J. *Acc. Chem. Res.* **43**, 1486–1495 (2010).
9. Korch, K. M., Watson, D. A. *Chem. Rev.* **119**, 8192–8228 (2019).
10. Berger, F., Plutschack, M. B., Riegger, J., Yu, W., Speicher, S., Ho, M., Frank, N., Ritter, T. *Nature* **567**, 223–228 (2019).
11. Berger, F., Ritter, T. *Synlett* **33**, 339–345 (2022).
12. Meng, H., Liu, M.-S., Shu, W. *Chem. Sci.* **13**, 13690–13707 (2022).
13. Biscoe, M. R., Cornella, J., Kalyani, D., Neufeldt, S. *J. Org. Chem.* **89**, 16065–16069 (2024).
14. Liu, C., Zhang, H., Shi, W., Lei, A. *Chem. Rev.* **111**, 1780–1824 (2011).
15. Shi, W., Liu, C., Lei, A. *Chem. Soc. Rev.* **40**, 2761–2776 (2011).
16. Ehehalt, L. E., Beleh, O. M., Priest, I. C., Mouat, J. M., Olszewski, A. K., Ahern, B. N., Cruz, A. R., Chi, B. K., Castro, A. J., Kang, K., Wang, J., Weix, D. J. *Chem. Rev.* **124**, 13397–13569 (2024).
17. Shimada, S., Rao, M. L. N. "*Transition-Metal Catalyzed C–C Bond Formation Using Organobismuth Compounds*", in *Bismuth-Mediated Organic Reactions*, T. Ollevier, Ed., *Top. Curr. Chem.* **311**, 199–228 (Springer, Berlin, 2012).
18. Barton, D. H. R., Ozbalik, N., Ramesh, M. *Tetrahedron* **44**, 5661–5668 (1988).
19. Suzuki, H., Murafuji, T., Azuma, N. *J. Chem. Soc., Perkin Trans. 1*, 1593–1600 (1992).
20. Rao, M. L. N., Shimada, S., Tanaka, M. *Org. Lett.* **1**, 1271–1273 (1999).
21. Rao, M. L. N., Shimada, S., Yamazaki, O., Tanaka, M. *J. Organomet. Chem.* **659**, 117 (2002).
22. Rao, M. L. N., Venkatesh, V., Jadhav, D. N. *Tetrahedron Lett.* **47**, 6975–6978 (2006).
23. Shimada, S., Yamazaki, O., Tanaka, T., Rao, M. L. N., Suzuki, Y., Tanaka, M. *Angew. Chem. Int. Ed.* **42**, 1845–1848 (2003).
24. Shimada, S., Wang, X.-B., Tanaka, M. *Chem. Commun.* **56**, 15216–15219 (2020).
25. Chen, L., Sanchez, D. R., Zhang, B., Carrow, B. P. *J. Am. Chem. Soc.* **139**, 12418–12421 (2017).
26. Zhao, D., Niu, J., Song, H., Cui, Y., Yan, K., Ritter, T. *Nature* **600**, 444–449 (2021).
27. Zhang, L., Xie, Y., Bai, Z., Ritter, T. *Nat. Synth.* **3**, 1490–1497 (2024).
28. Lipshultz, J. M., Li, G., Radosevich, A. T. *J. Am. Chem. Soc.* **143**, 1699–1721 (2021).
29. Moon, H.-W., Cornella, J. *ACS Catal.* **12**, 1382–1393 (2022).
30. Mato, M., Cornella, J. *Angew. Chem. Int. Ed.* **63**, e202315046 (2024).
31. Pang, Y., Leutzsch, M., Nöthling, N., Katzenburg, F., Cornella, J. *J. Am. Chem. Soc.* **143**, 12487–12493 (2021).
32. Mato, M., Bruzzese, P. C., Takahashi, T., Leutzsch, M., Reijerse, E. J., Schnegg, A., Cornella, J. *J. Am. Chem. Soc.* **145**, 18742–18747 (2023).
33. Mato, M., Stamoulis, A., Cleto-Bruzzese, P. C., Cornella, J. *Angew. Chem. Int. Ed.* **64**, e202418367 (2024).
34. Stamoulis, A., Mato, M., Cleto-Bruzzese, P. C., Leutzsch, M., Cadranel, M., Gil-Sepulcre, M., Neese, M., Cornella, J. *J. Am. Chem. Soc.* **147**, 6037–6048 (2025).

35. Liu, X., Moon, H.-W., Spinnato, D., Leutzsch, M., Cornella, J. *Angew. Chem. Int. Ed.* **64**, e202510360 (2025).
36. Snieckus, V. *Chem. Rev.* **90**, 879–933 (1990).
37. Gilman, H., Mallory, H. E., Wright, G. F. *J. Am. Chem. Soc.* **54**, 733–736 (1932).
38. Cordell, G. A. *J. Org. Chem.* **40**, 3161–3169 (1975).
39. Fauvarque, J.-F., Pflüger, F., Troupel, M. *J. Organomet. Chem.* **208**, 419–427 (1981).
40. Nishikata, T., Yamamoto, Y., Miyaura, N. *Organometallics* **23**, 4317–4324 (2004).
41. Cook, X. A. F., de Gombert, A., McKnight, J., Pantaine, L. R. E., Willis, M. C. *Angew. Chem. Int. Ed.* **60**, 11068–11091 (2021).
42. Li, G.-Q., Kiyomura, S., Yamamoto, Y., Miyaura, N. *Chem. Lett.* **40**, 702–704 (2011).
43. Ingner, F. J. L., Schmitt, A. C., Orthaber, A., Gates, P. J., Pilarski, L. T. *ChemSusChem* **13**, 2032–2037 (2020).
44. Li, G.-Q., Yamamoto, Y., Miyaura, N. *Tetrahedron* **67**, 6804–6811 (2011).
45. Chen, X.-Y., Li, Y.-N., Wu, Y., Bai, J., Guo, Y., Wang, P. *J. Am. Chem. Soc.* **145**, 10431–10440 (2023).
46. Ahmadli, D., Müller, S., Xie, Y., Smejkal, T., Jaeckh, S., Iosub, A. V., Williams, S. R., Ritter, T. *J. Am. Chem. Soc.* **147**, 4268–4283 (2025).
47. Ni, S., Stamoulis, A., Béland, V. A., Cornella, J. *Nat. Catal.* **8**, 1232–1240 (2025).
48. Dzhevakov, P. B., Topchiy, M. A., Zharkova, D. A., Morozov, O. S., Asachenko, A. F., Nechaev, M. S. *Adv. Synth. Catal.* **358**, 977–983 (2016).
49. de Gombert, A., McKay, A. I., Davis, C. J., Wheelhouse, K. M., Willis, M. C. *J. Am. Chem. Soc.* **142**, 3564–3576 (2020).
50. Ahmed, J., Chakraborty, S., Jose, A., Sreejyothi, P., Mandal, S. K. *J. Am. Chem. Soc.* **140**, 8330–8339 (2018).
51. Zhao, J., Niu, S., Jiang, X., Jiang, Y., Zhang, X., Sun, T., Ma, D. *J. Org. Chem.* **83**, 6589–6598 (2018).
52. Takise, R., Itami, K., Yamaguchi, J. *Org. Lett.* **18**, 4428–4431 (2016).
53. Steib, A. K., Kuzmina, O. M., Fernandez, S., Flubacher, D., Knochel, P. *J. Am. Chem. Soc.* **135**, 15346–15349 (2013).
54. Guan, B.-T., Xiang, S.-K., Wang, B.-Q., Sun, Z.-P., Wang, Y., Zhao, K.-Q., Shi, Z.-J. *J. Am. Chem. Soc.* **130**, 3268–3269 (2008).
55. Guo, L., Srimontree, W., Zhu, C., Maity, B., Liu, X., Cavallo, L., Rueping, M. *Nat. Commun.* **10**, 1957 (2019).
56. Mohadjer Beromi, M., Nova, A., Balcells, D., Brasacchio, A. M., Brudvig, G. W., Guard, L. M., Hazari, N., Vinyard, D. J. *J. Am. Chem. Soc.* **139**, 922–936 (2017).
57. Zhang, R., Zhao, Y., Liu, K.-M., Duan, X.-F. *Org. Lett.* **20**, 7942–7946 (2018).
58. Reeves, E. K., Humke, J. N., Neufeldt, S. R. *J. Org. Chem.* **84**, 11799–11812 (2019).
59. Kalvet, I., Magnin, G., Schoenebeck, F. *Angew. Chem. Int. Ed.* **56**, 1581–1585 (2017).
60. Okura, K., Teranishi, T., Yoshida, Y., Shirakawa, E. *Angew. Chem. Int. Ed.* **57**, 7186–7190 (2018).
61. Morofuji, T., Yoshida, T., Tsutsumi, R., Yamanaka, M., Kano, N. *Chem. Commun.* **56**, 13995–13998 (2020).
62. Jong, H., Eey, S. T.-C., Lim, Y. H., Pandey, S., Iqbal, N. A. B., Yong, F. F., Robins, E. G, Johannes, C. W. *Adv. Synth. Catal.* **359**, 616–622 (2017).
63. Che, Y.-Y., Yue, Y., Lin, L.-Z., Pei, B., Deng, X., Feng, C. *Angew. Chem. Int. Ed.* **59**, 16414–16419 (2020).
64. Chen, Q., Wu, S., Yan, S., Li, C., Abduhulam, H., Shi, Y., Dang, Y., Cao, C. *ACS Catal.* **10**, 8168–8176 (2020).
65. Martinez-Solorio, D., Melillo, B., Sanchez, L., Liang, Y., Lam, E., Houk, K. N., Smith, A. B. III. *J. Am. Chem. Soc.* **138**, 1836–1839 (2016).
66. El-Deeb, I. Y., Funakoshi, T., Shimomoto, Y., Matsubara, R., Hayashi, M. *J. Org. Chem.* **82**, 2630–2640 (2017).

67. Zhou, M., Ni, C., Zeng, Y., Hu, J. *J. Am. Chem. Soc.* **140**, 6801–6805 (2018).
68. Altmann, L.-M., Fürst, M. C. D., Gans, E. I., Zantop, V., Pratsch, G., Heinrich, M. R. *Org. Lett.* **22**, 479–482 (2020).
69. Liang, Q., Xing, P., Huang, Z., Dong, J., Sharpless, K. B., Li, X., Jiang, B. *Org. Lett.* **17**, 1942–1945 (2015).
70. Qin, H.-T., Xu, X., Liu, F. *ChemCatChem* **9**, 1409–1412 (2017).
71. Zhu, F., Wang, Z.-X. *Adv. Synth. Catal.* **355**, 3694–3702 (2013).
72. Duczynski, J., Sobolev, A. N., Moggach, S. A., Dorta, R., Stewart, S. G. *Organometallics* **39**, 105–115 (2019).
73. Martinez-Solorio, D., Melillo, B., Sanchez, L., Liang, Y., Lam, E., Houk, K. N., Smith, A. B. III. *J. Am. Chem. Soc.* **138**, 1836–1839 (2016).
74. Guo, Y.-Q., Chen, F., Deng, C.-L., Zhang, X.-G. *Chem. Commun.* **57**, 1923–1926 (2021).
75. So, C. M., Lee, H. W., Lau, C. P., Kwong, F. Y. *Org. Lett.* **11**, 317–320 (2009).
76. Kobayashi, O., Uraguchi, D., Yamakawa, T. *Org. Lett.* **11**, 2679–2682 (2009).
77. Kumar, R., Namboothiri, I. N. N. *Org. Lett.* **13**, 4016–4019 (2011).
78. Shao, C., Shi, G., Zhang, Y., Pan, S., Guan, X. *Org. Lett.* **17**, 2652–2655 (2015).
79. Wu, Q., Mao, Y.-J., Zhou, K., Wang, S., Chen, L., Xu, Z.-Y., Lou, S.-J., Xu, D.-Q. *Chem. Commun.* **57**, 4544–4547 (2021).
80. Nguyen, M. H., Smith, A. B. III. *Org. Lett.* **16**, 2070–2073 (2014).
81. Shen, B., He, Y., Kim, Y., Wang, Y., Lee, M. *Angew. Chem. Int. Ed.* **55**, 2382–2386 (2016).
82. Tamba, S., Okubo, Y., Tanaka, S., Monguchi, D., Mori, A. *J. Org. Chem.* **75**, 6998–7001 (2010).
83. Leowanawat, P., Zhang, N., Resmerita, A.-M., Rosen, B. M., Percec, V. *J. Org. Chem.* **76**, 9946–9955 (2011).
84. Tao, X., Ma, G., Song, Y., Chen, Y., Qian, Q., Sun, D., Gong, H. *Org. Lett.* **23**, 7418–7422 (2021).
85. Zhang, S., Tang, Z., Bao, W., Li, J., Guo, B., Huang, S., Zhang, Y., Rao, Y. *Org. Biomol. Chem.* **17**, 4364–4369 (2019).
86. Gurung, S. K., Thapa, S., Kafle, A., Dickie, D. A., Giri, R. *Org. Lett.* **16**, 1264–1267 (2014).
87. Selvi, T., Srinivasan, K. *J. Org. Chem.* **79**, 3653–3658 (2014).
88. Singh, P. P., Aithagani, S. K., Yadav, M., Singh, V. P., Vishwakarma, R. A. *J. Org. Chem.* **78**, 2639–2648 (2013).
89. Ivančič, A., Košmrlj, J., Gazvoda, M. *Commun. Chem.* **6**, 51 (2023).
90. Xu, J., Cao, J., Wu, X., Wang, H., Yang, X., Tang, X., Toh, R., Zhou, R., Yeow, E. K. L., Wu, J. *J. Am. Chem. Soc.* **143**, 13266–13273 (2021).
91. Lee, Y. H., Morandi, B. *Nat. Chem.* **10**, 1016–1022 (2018).
92. Molloy, J. J., Seath, C. P., West, M. J., McLaughlin, C., Fazakerley, N. J., Kennedy, A. R., Nelson, D. J., Watson, A. J. B. *J. Am. Chem. Soc.* **140**, 126–130 (2018).
93. Krasovskiy, A., Knochel, P. *Synthesis* **2006**, 0890–0891 (2006).

9. NMR spectra for Isolated Products and Substrates

Potassium (4-fluorophenyl)triolborate (ArB-1)

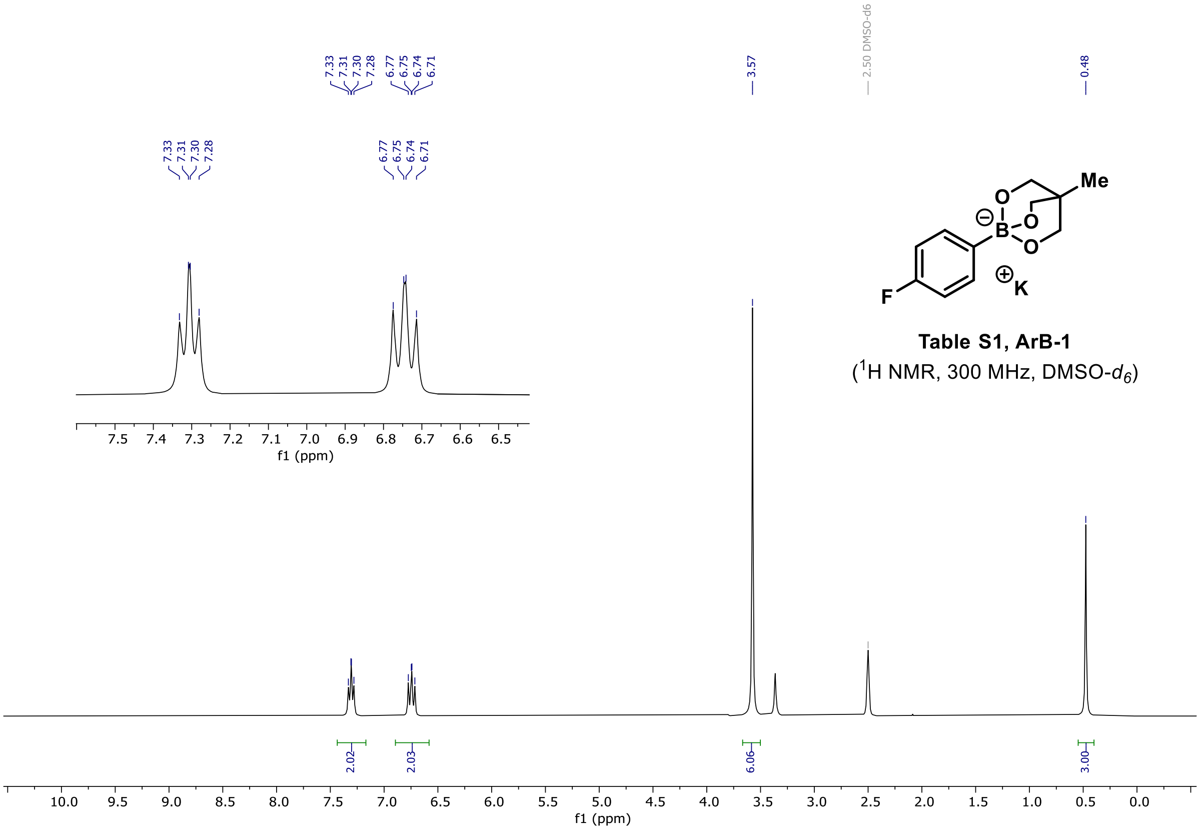

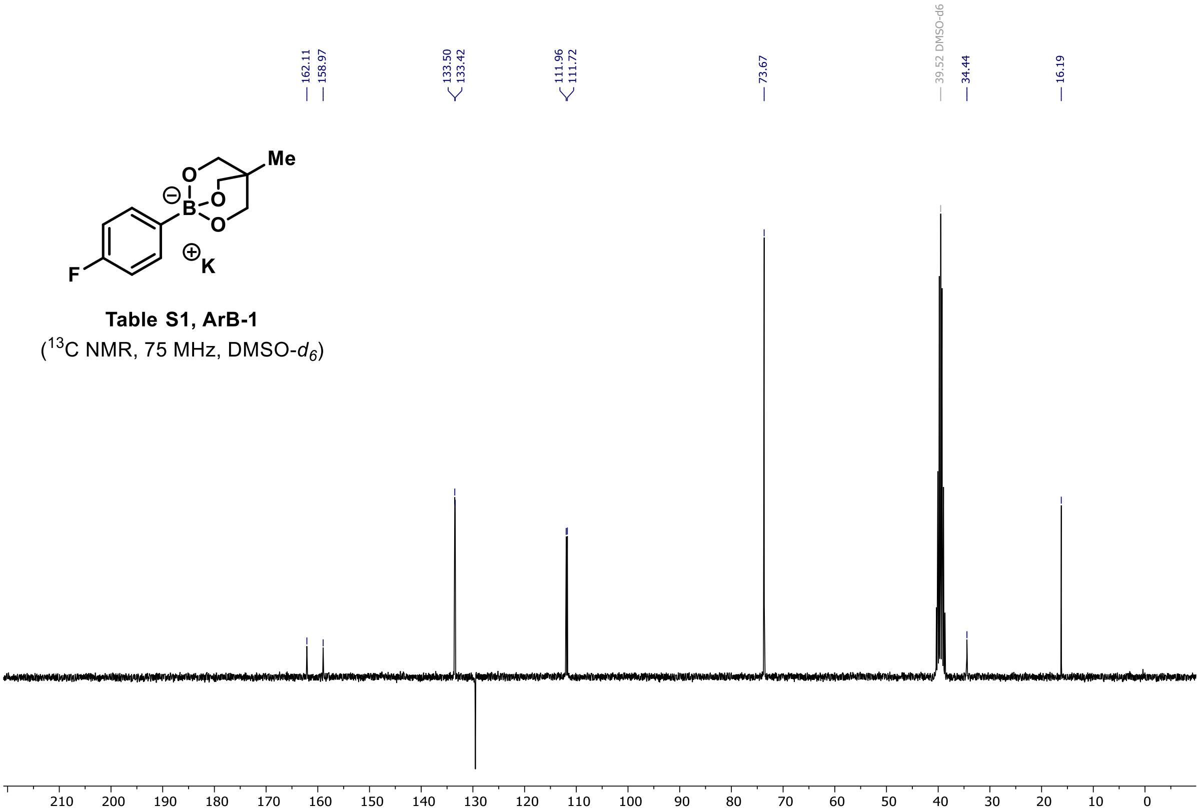

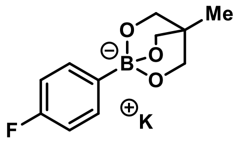

Table S1, ArB-1
(^{19}F NMR, 282 MHz, DMSO-d_6)

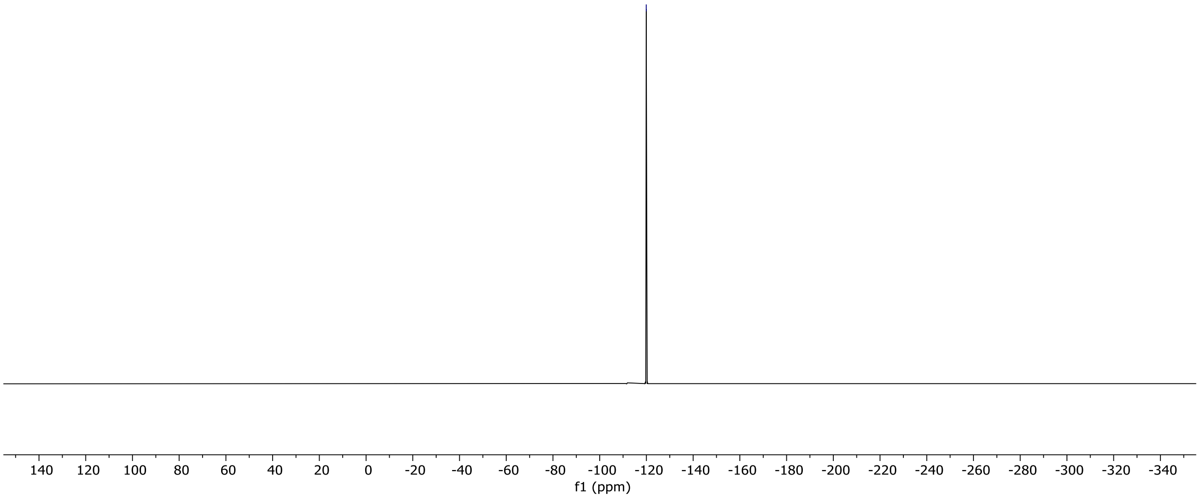

Potassium (4-methoxyphenyl)triolborate (ArB-2)

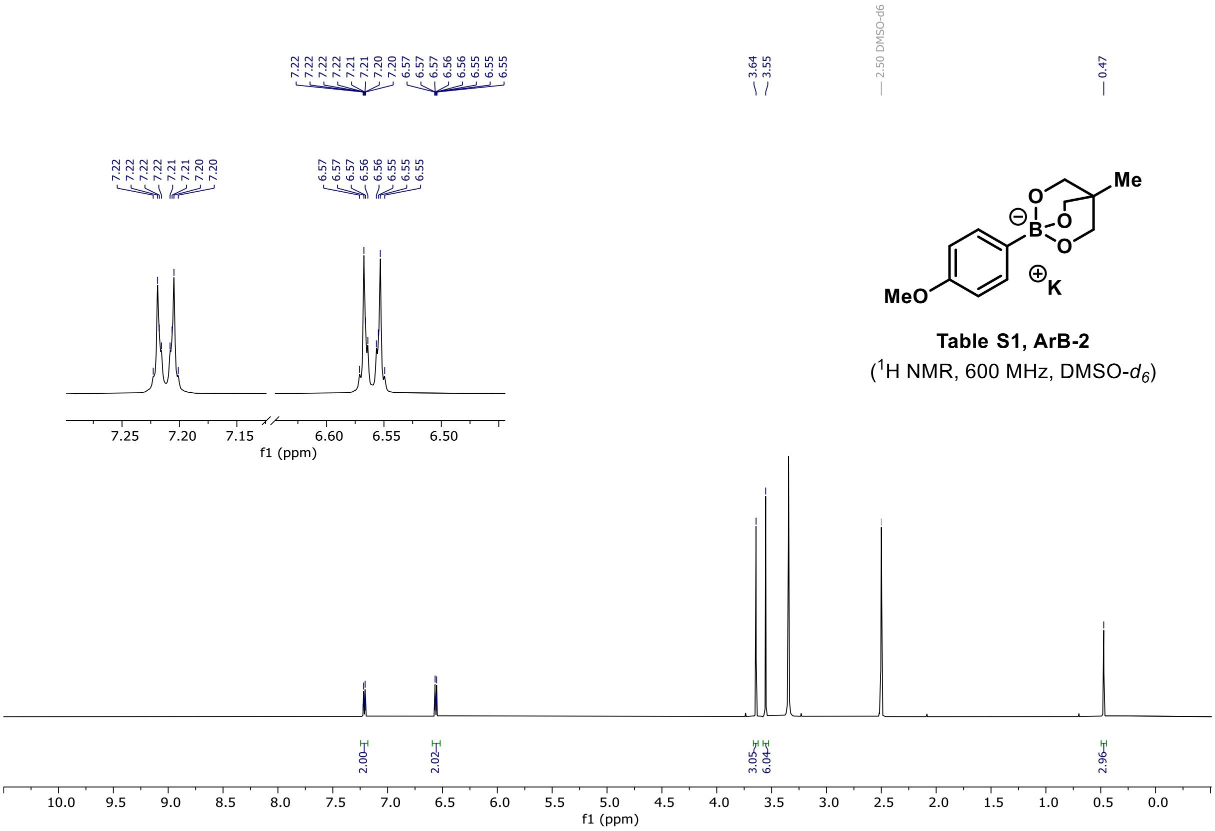

Table S1, ArB-2
(^{1}H NMR, 600 MHz, DMSO-d_6)

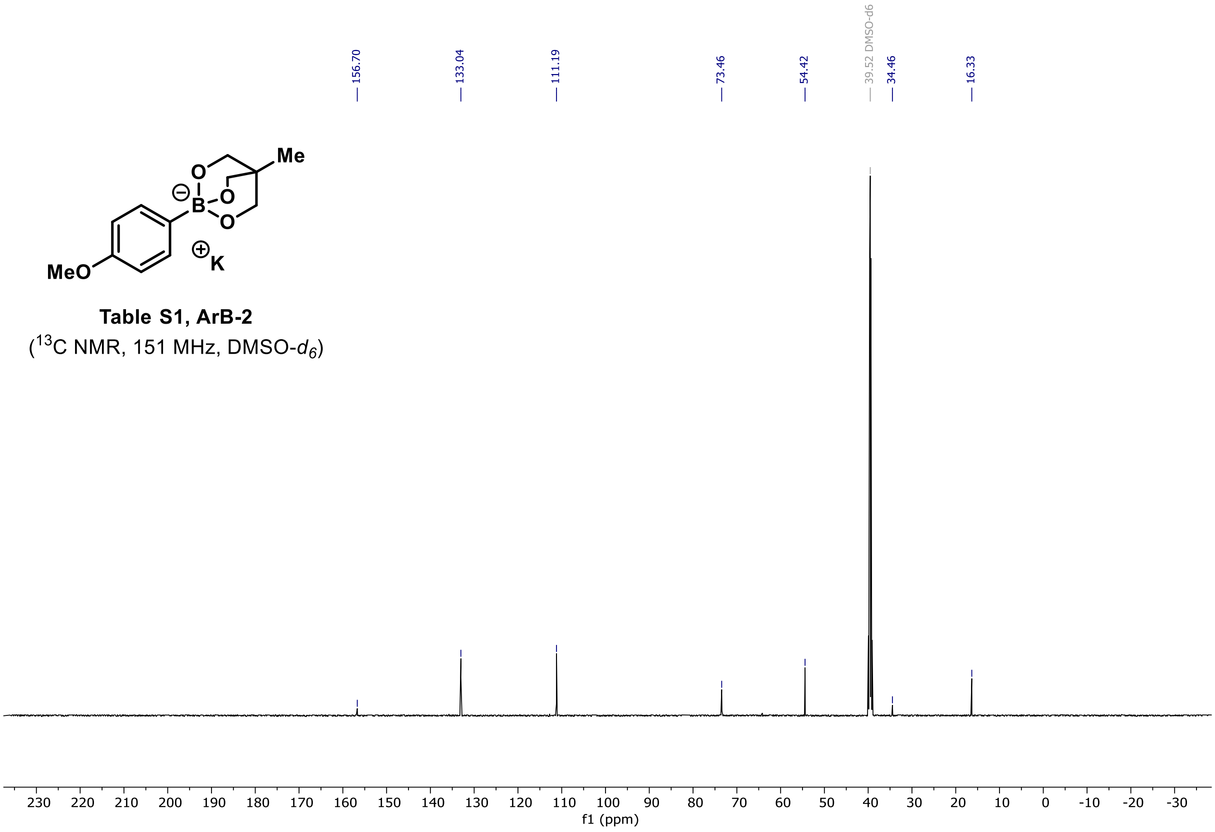

Table S1, ArB-2
(^{13}C NMR, 151 MHz, DMSO-d_6)

Potassium (4-(methoxycarbonyl)phenyl)triolborate (ArB-3)

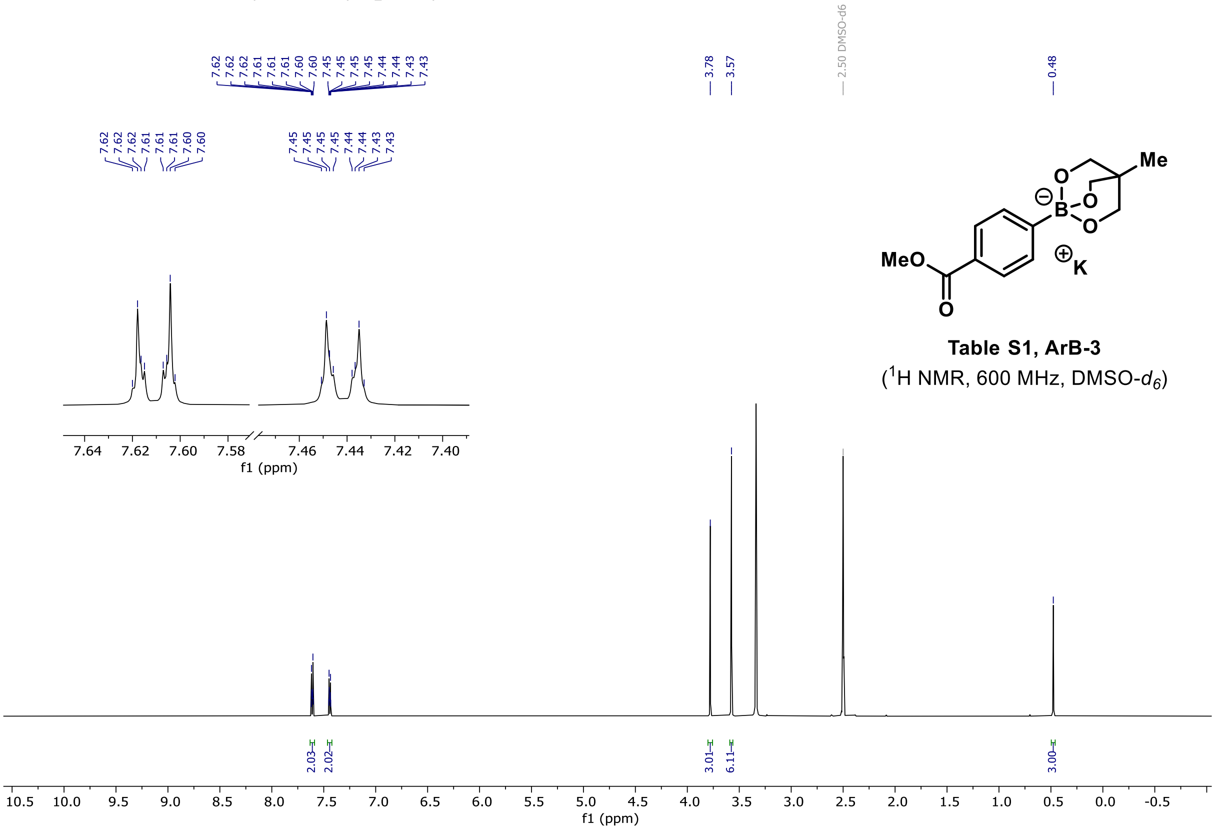

Table S1, ArB-3
(^{1}H NMR, 600 MHz, DMSO-d_6)

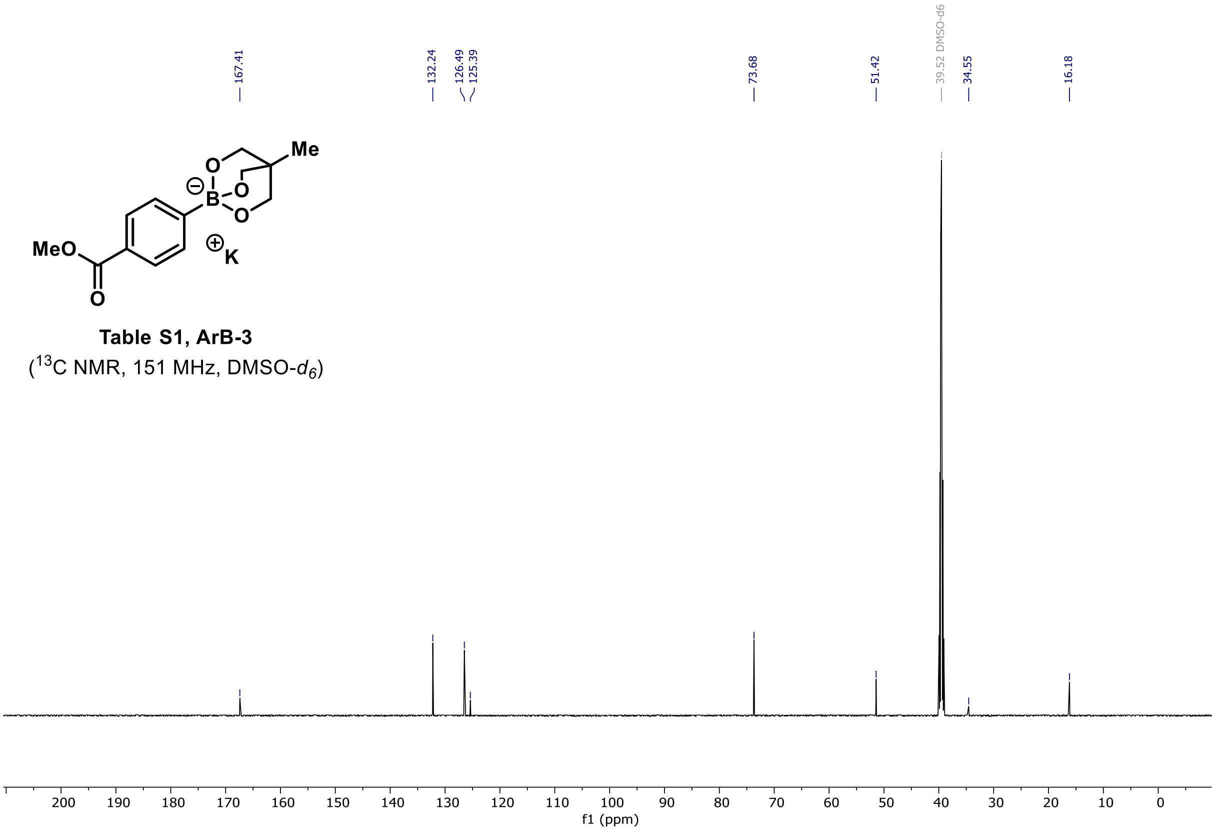

Table S1, ArB-3
(^{13}C NMR, 151 MHz, DMSO-d_6)

Potassium (4-(4-methylpiperazin-1-yl)phenyl)triolborate (ArB-4)

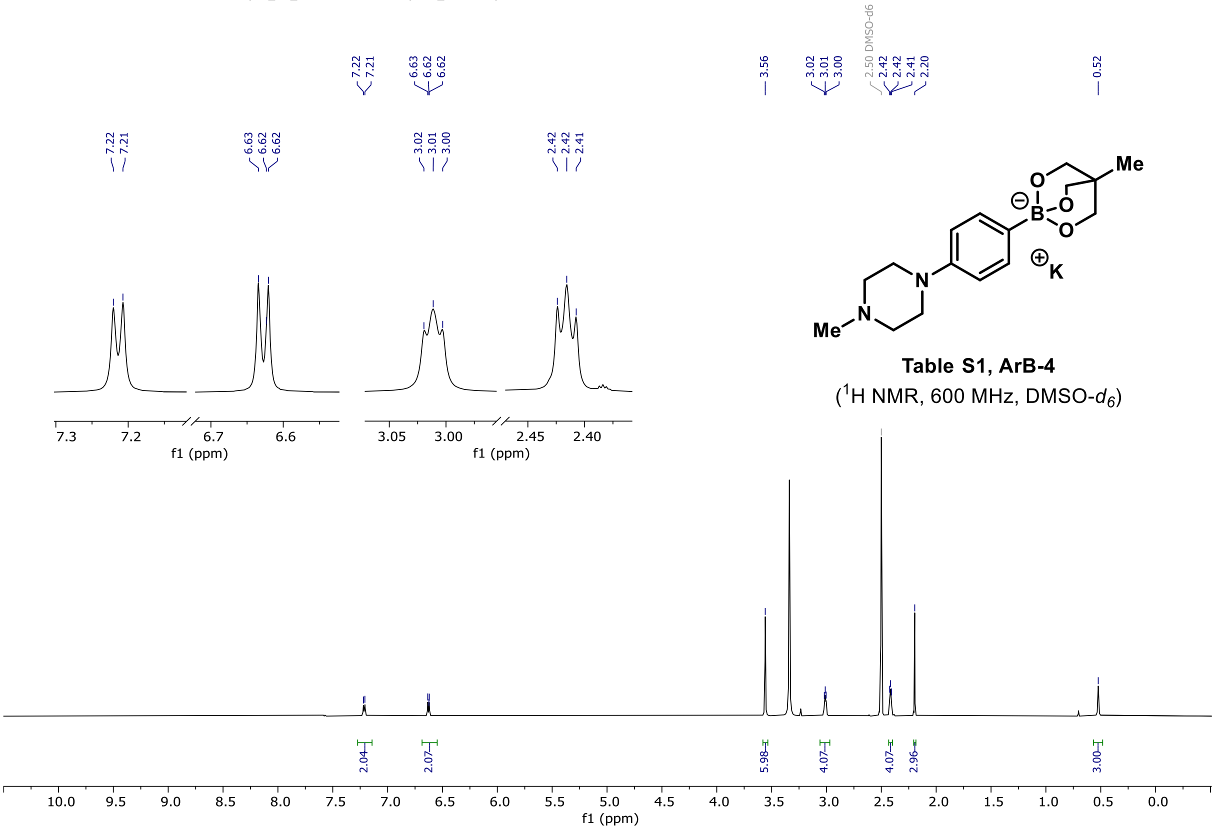

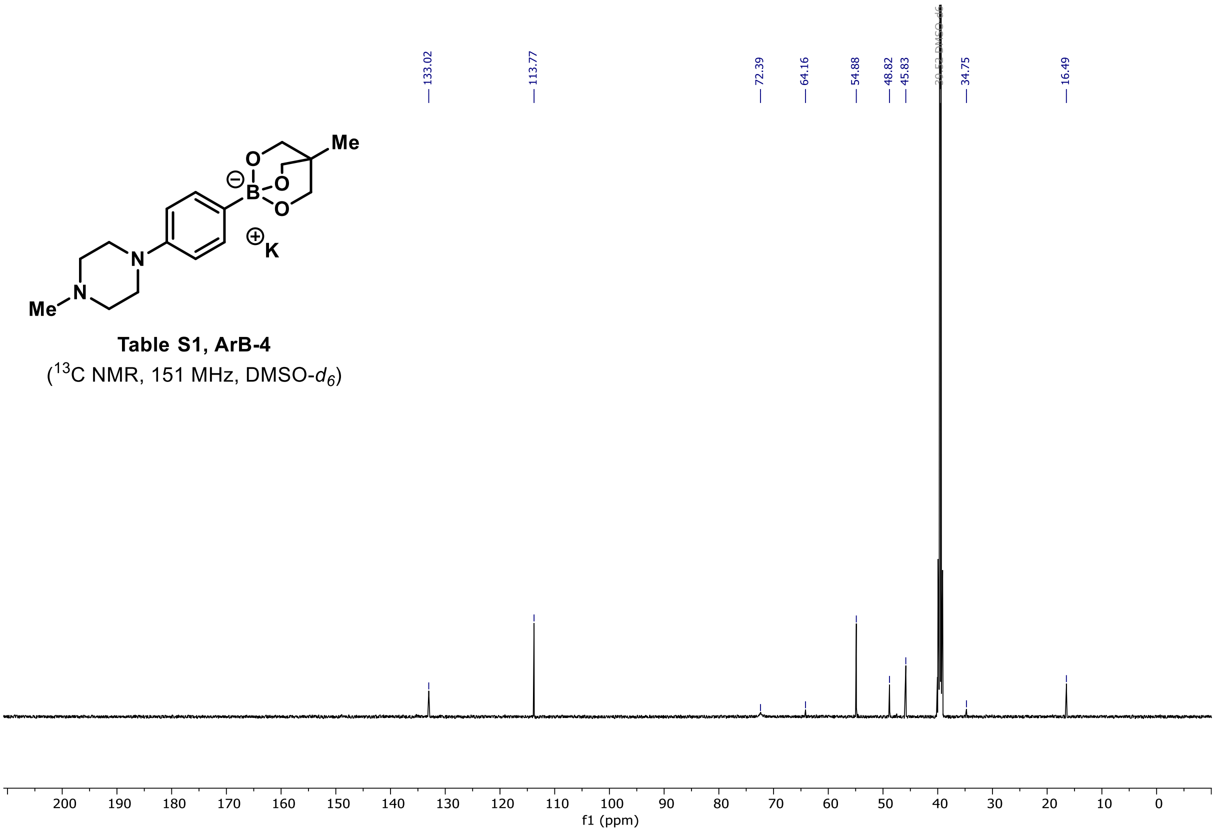

Potassium (4-(trifluoromethoxy)phenyl)triolborate (ArB-5)

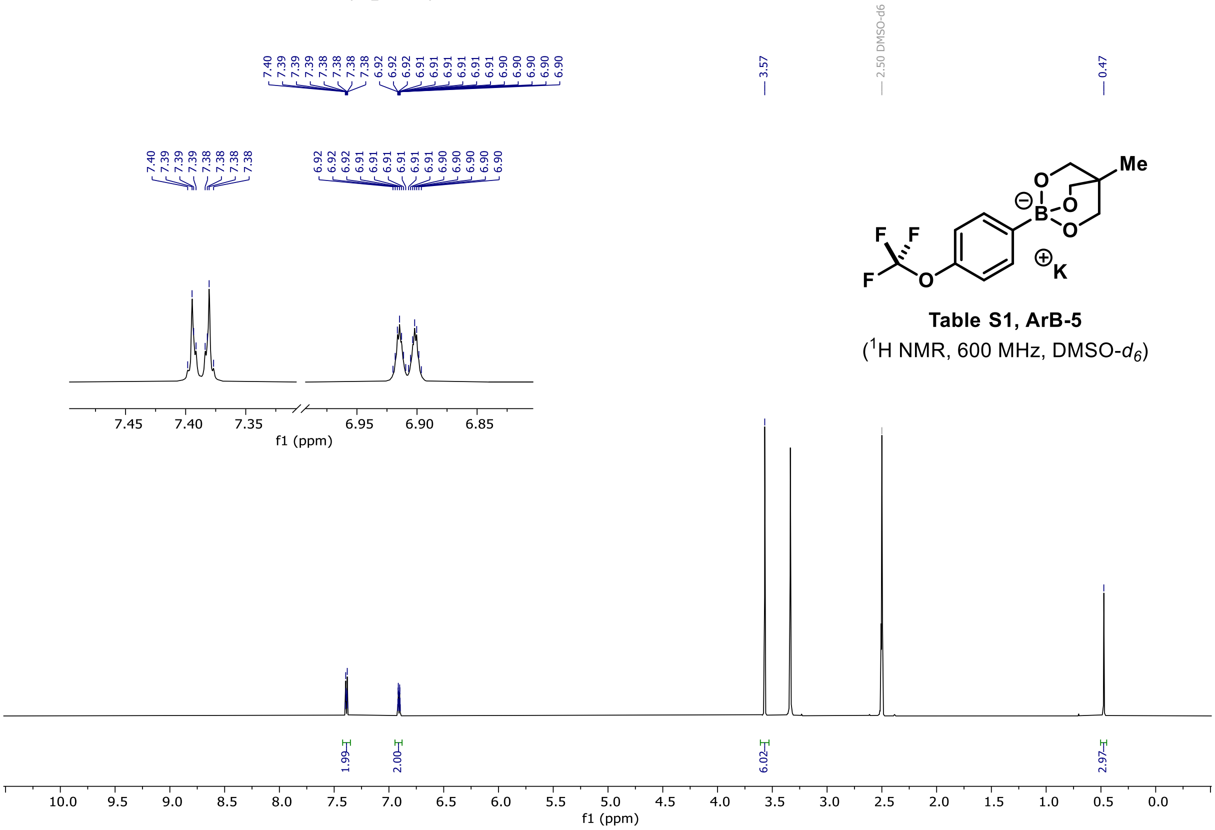

Table S1, ArB-5
(^{1}H NMR, 600 MHz, DMSO-d_6)

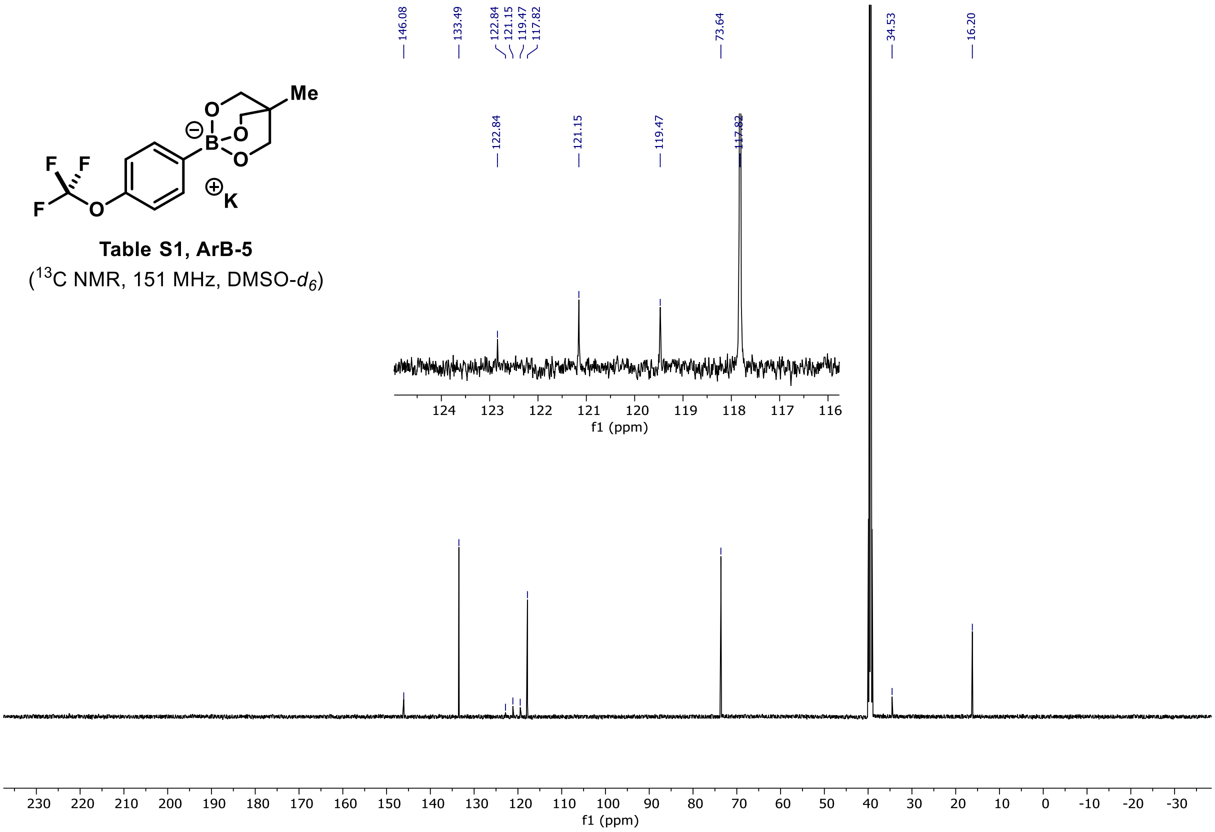

Table S1, ArB-5
(^{13}C NMR, 151 MHz, DMSO-d_6)

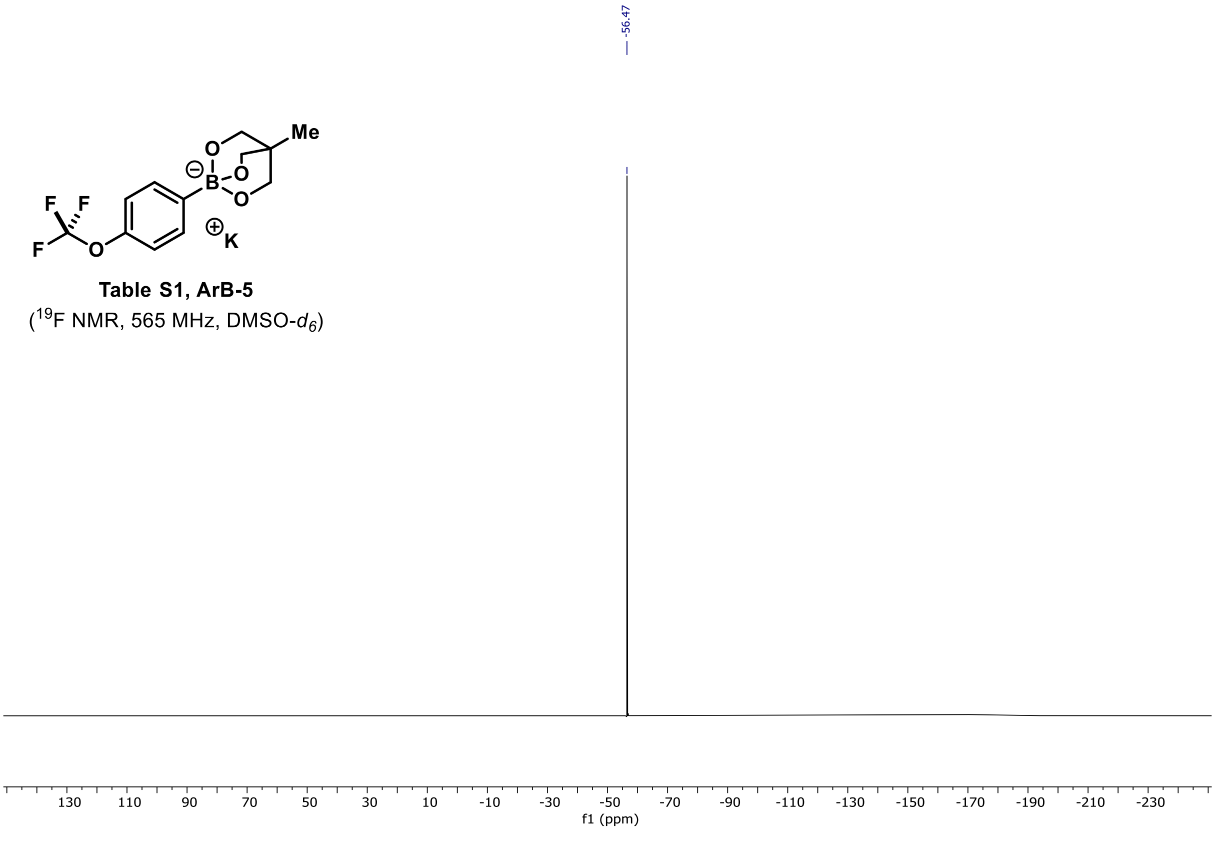

Table S1, ArB-5
(^{19}F NMR, 565 MHz, DMSO-d_6)

Potassium (4-bromophenyl)triolborate (ArB-6)

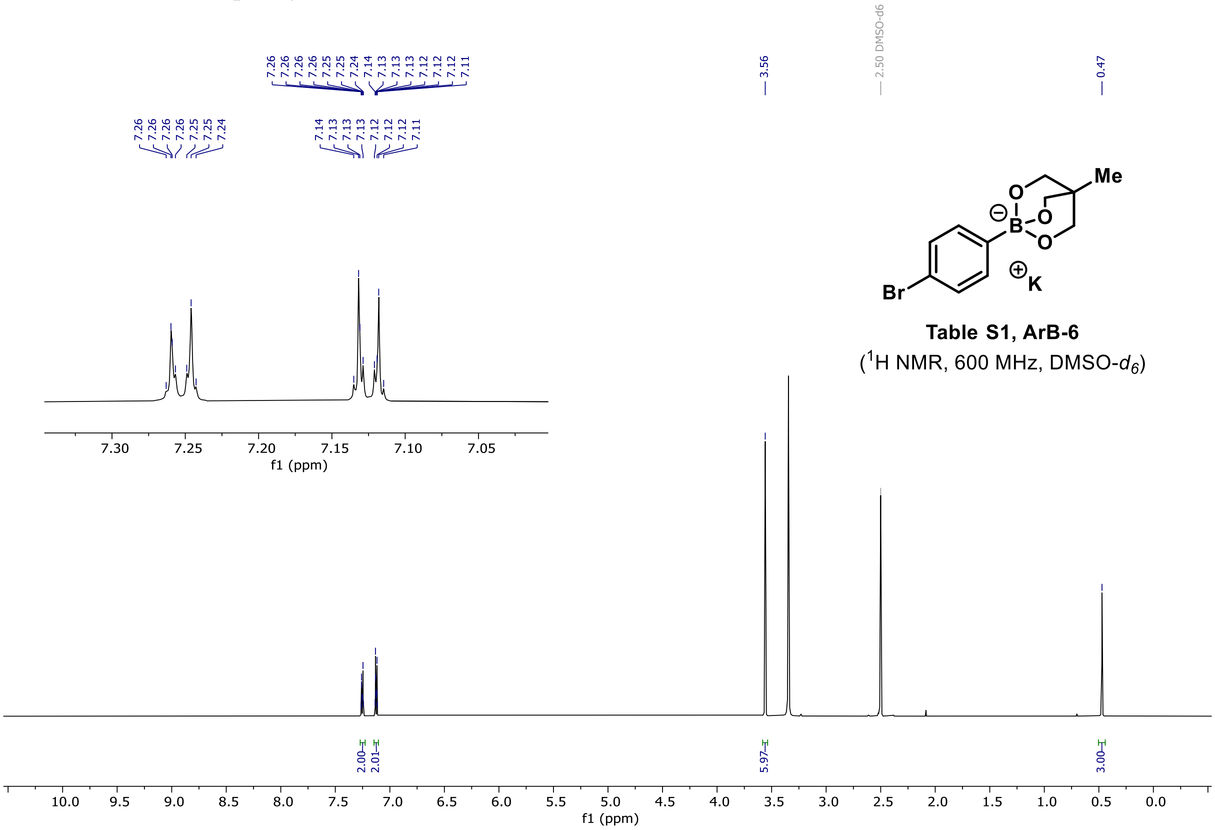

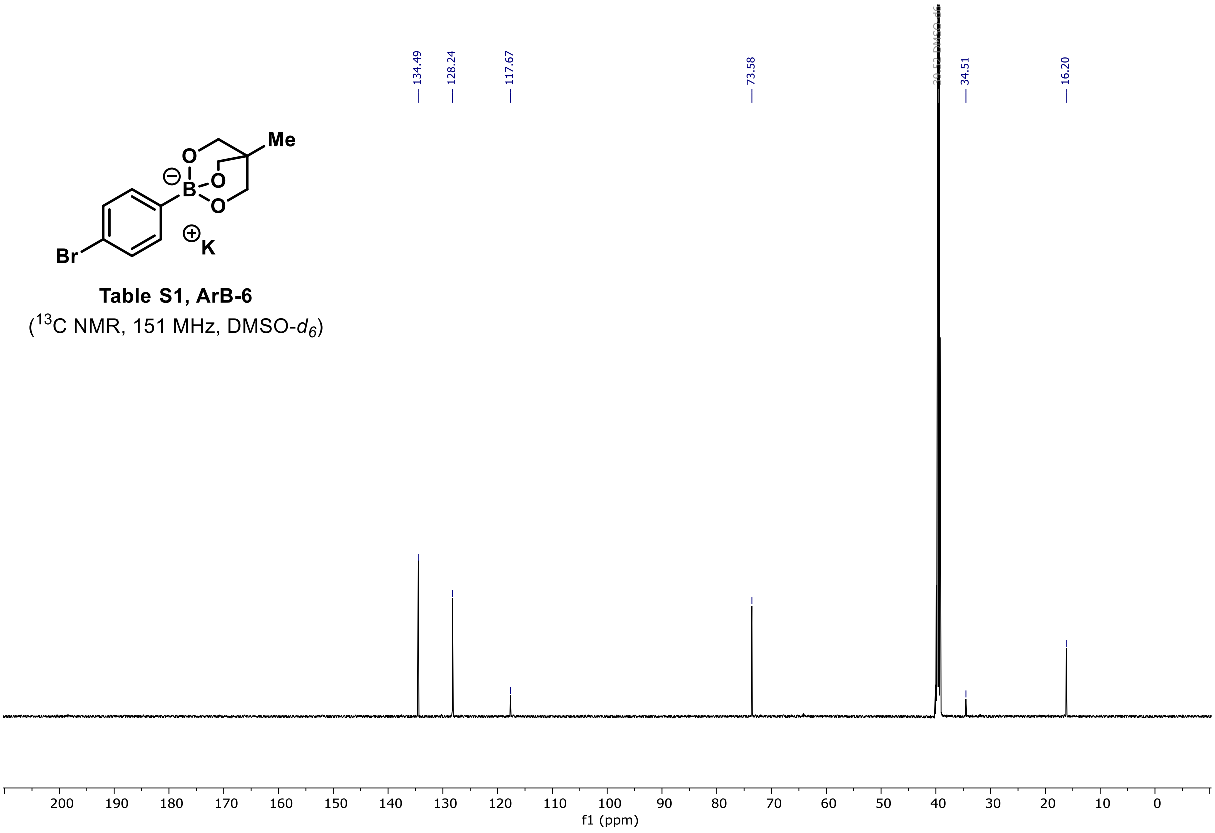

Potassium (2,2-difluorobenzo[d][1,3]dioxol-5-yl)triolborate (ArB-7)

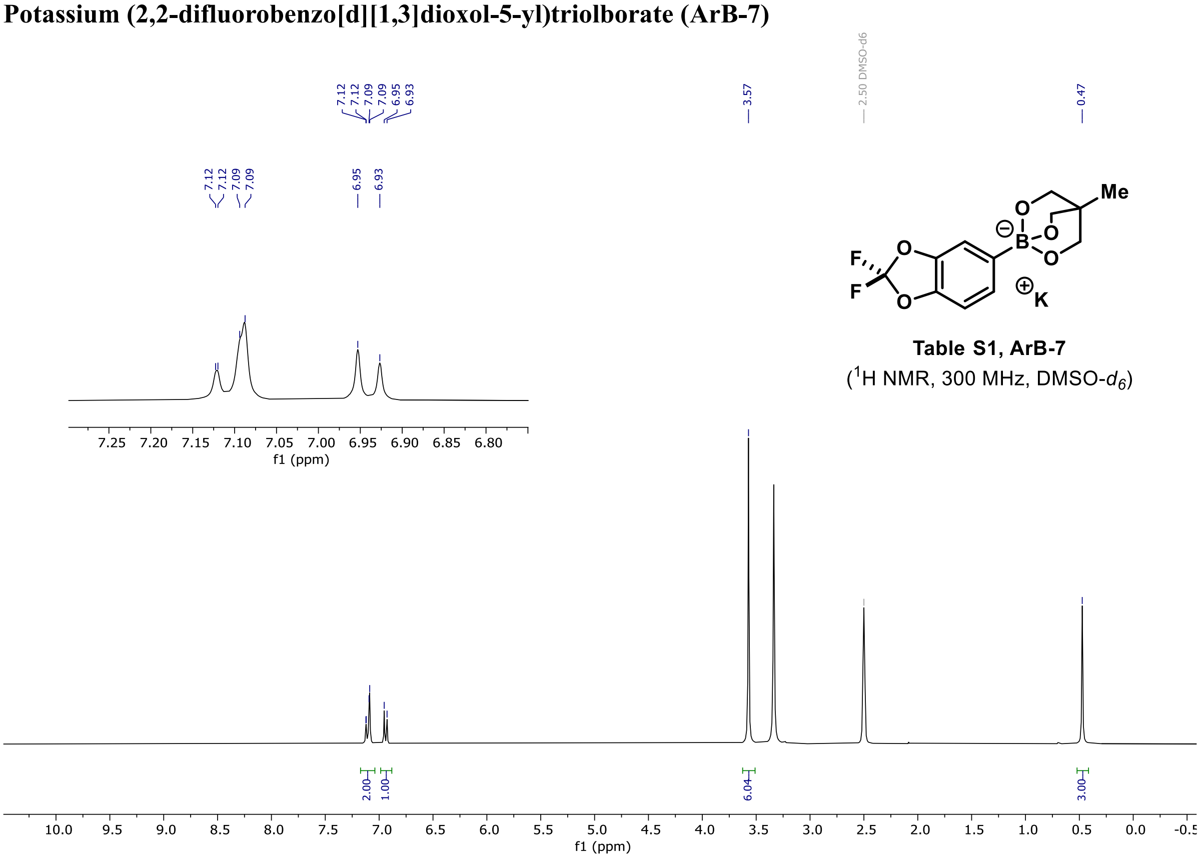

Table S1, ArB-7
(^{1}H NMR, 300 MHz, DMSO-d_6)

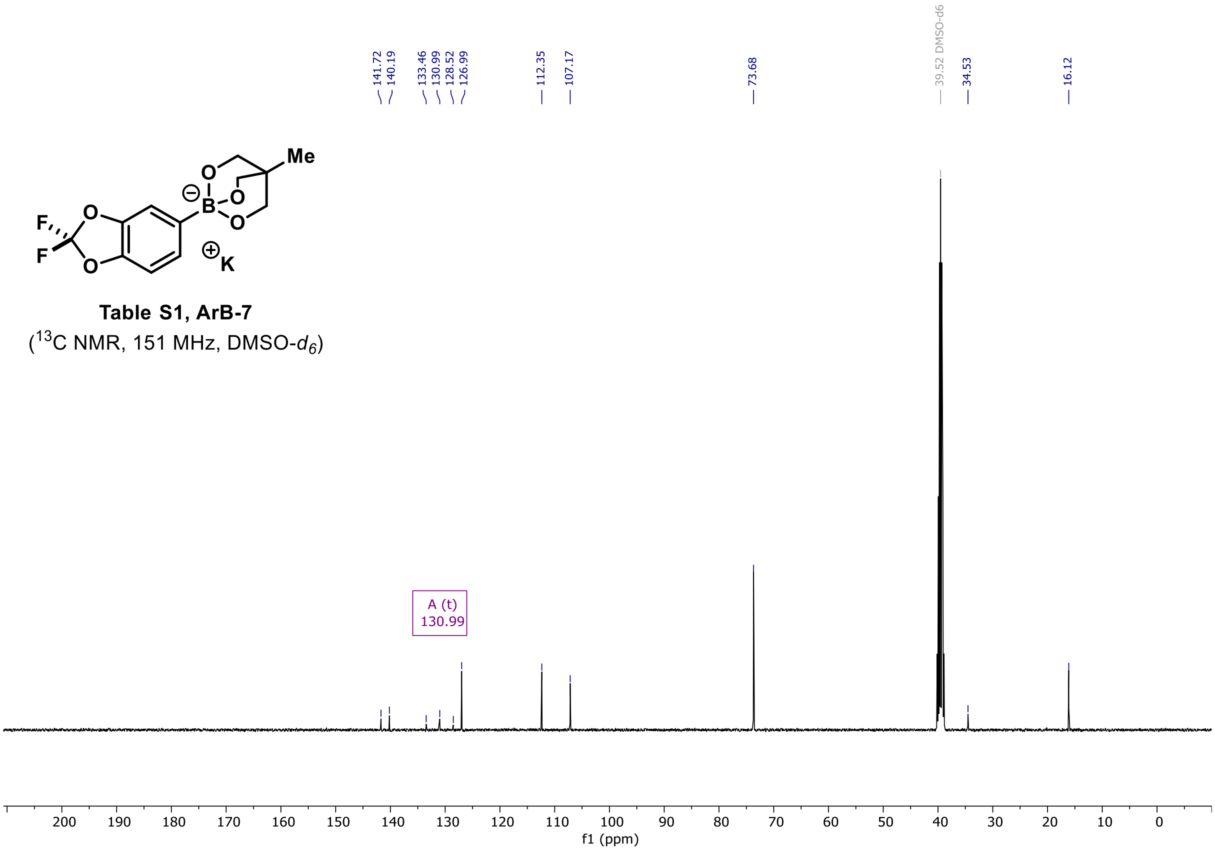

Table S1, ArB-7
(^{13}C NMR, 151 MHz, DMSO-d_6)

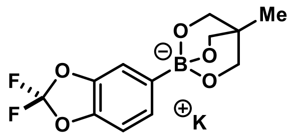

Table S1, ArB-7

(^{19}F NMR, 282 MHz, DMSO-d_6)

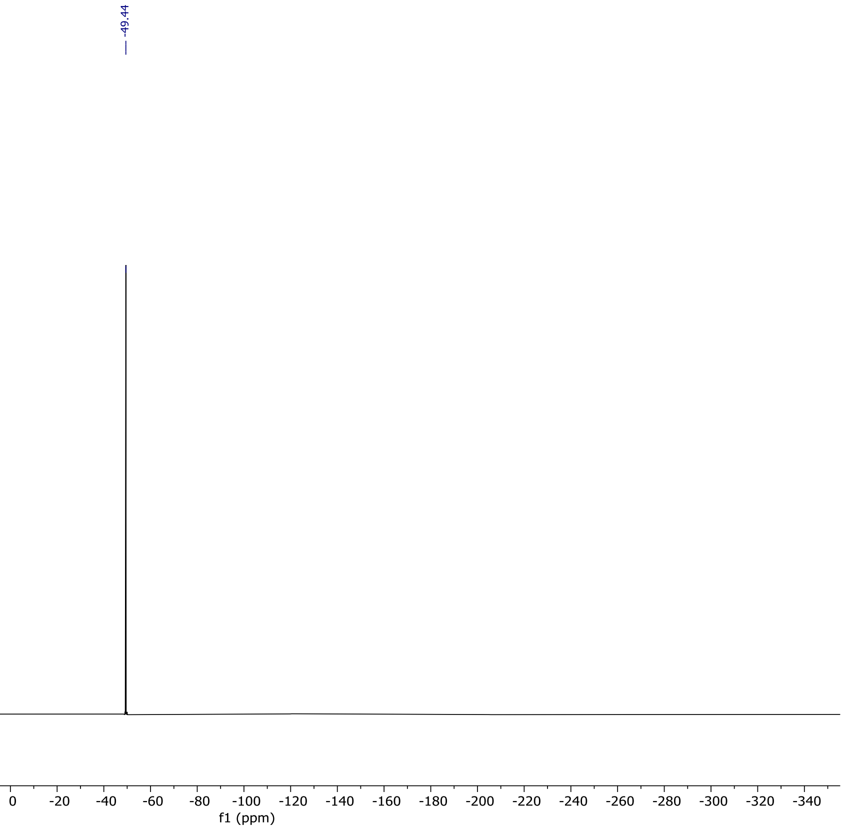

Potassium (4-((4-methoxyphenyl)ethynyl)phenyl)triolborate (ArB-8)

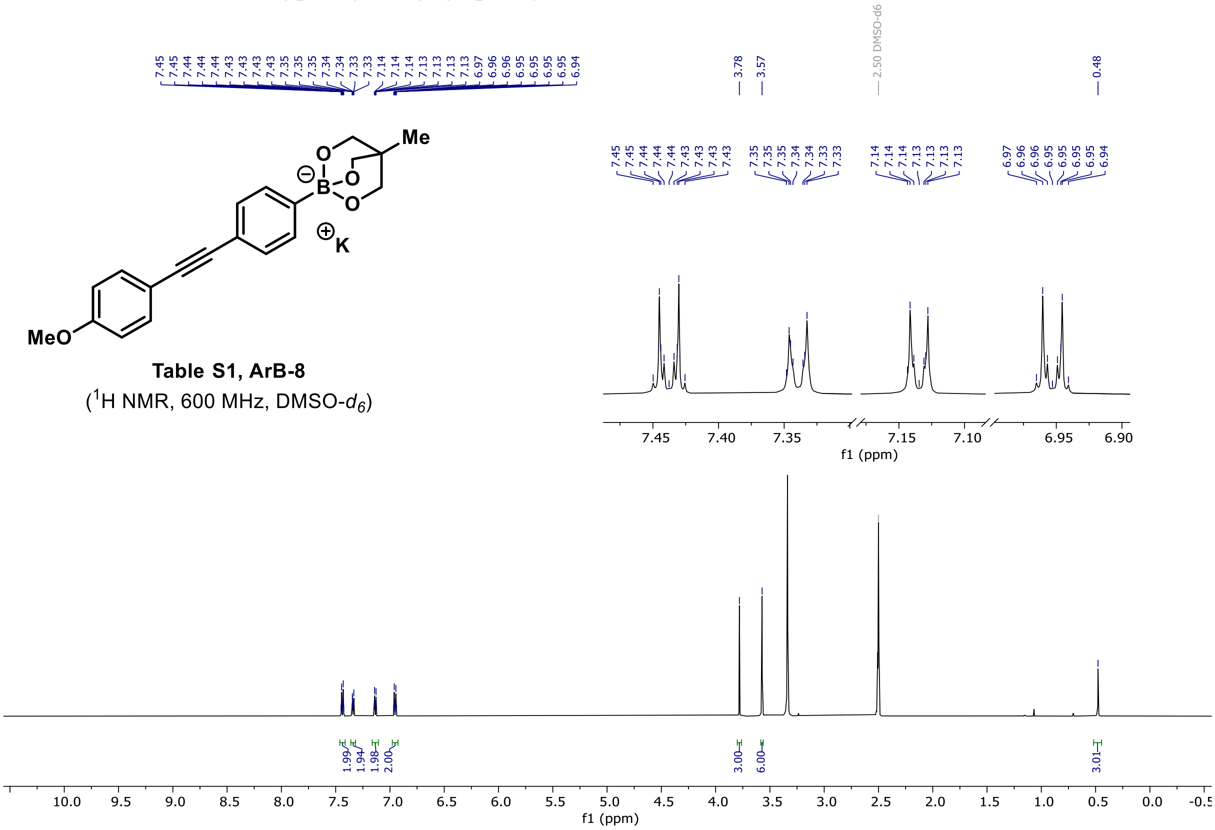

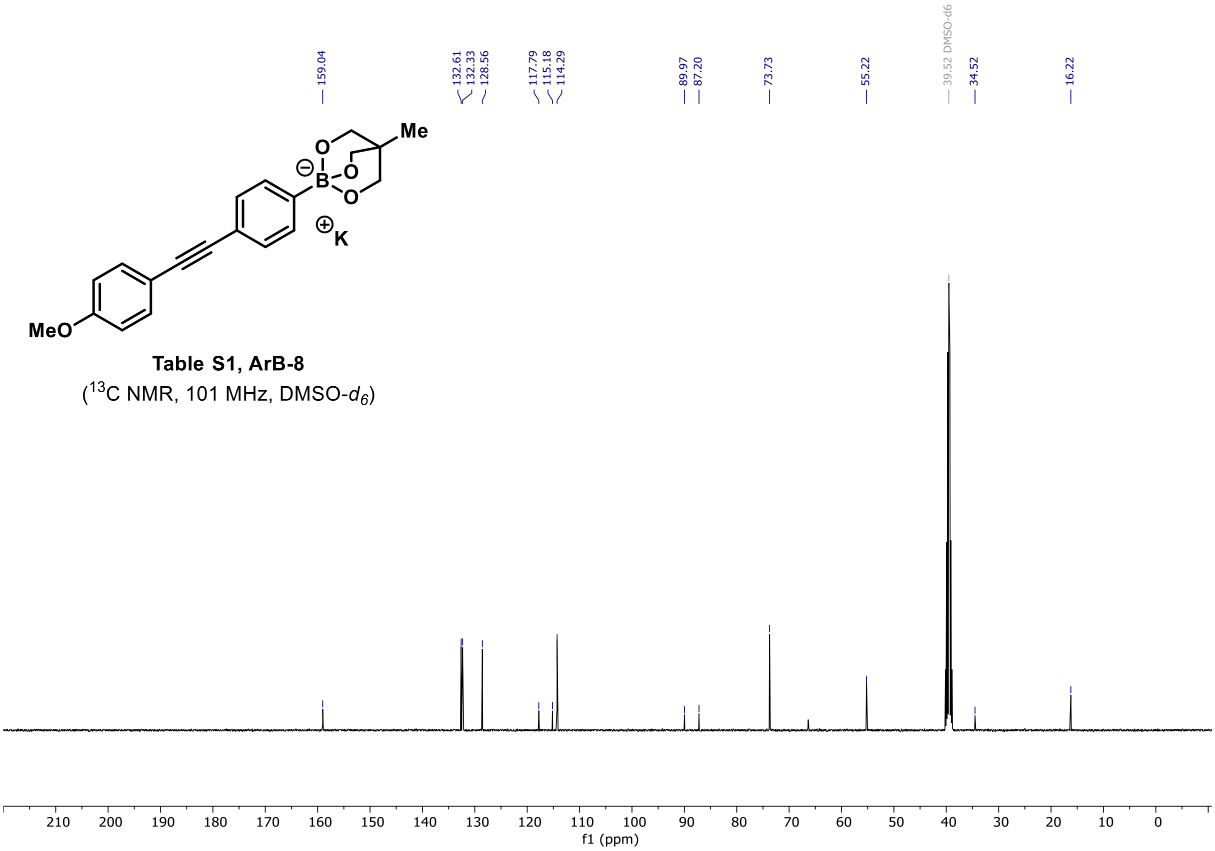

Potassium (2-naphthyl)triolborate (ArB-9)

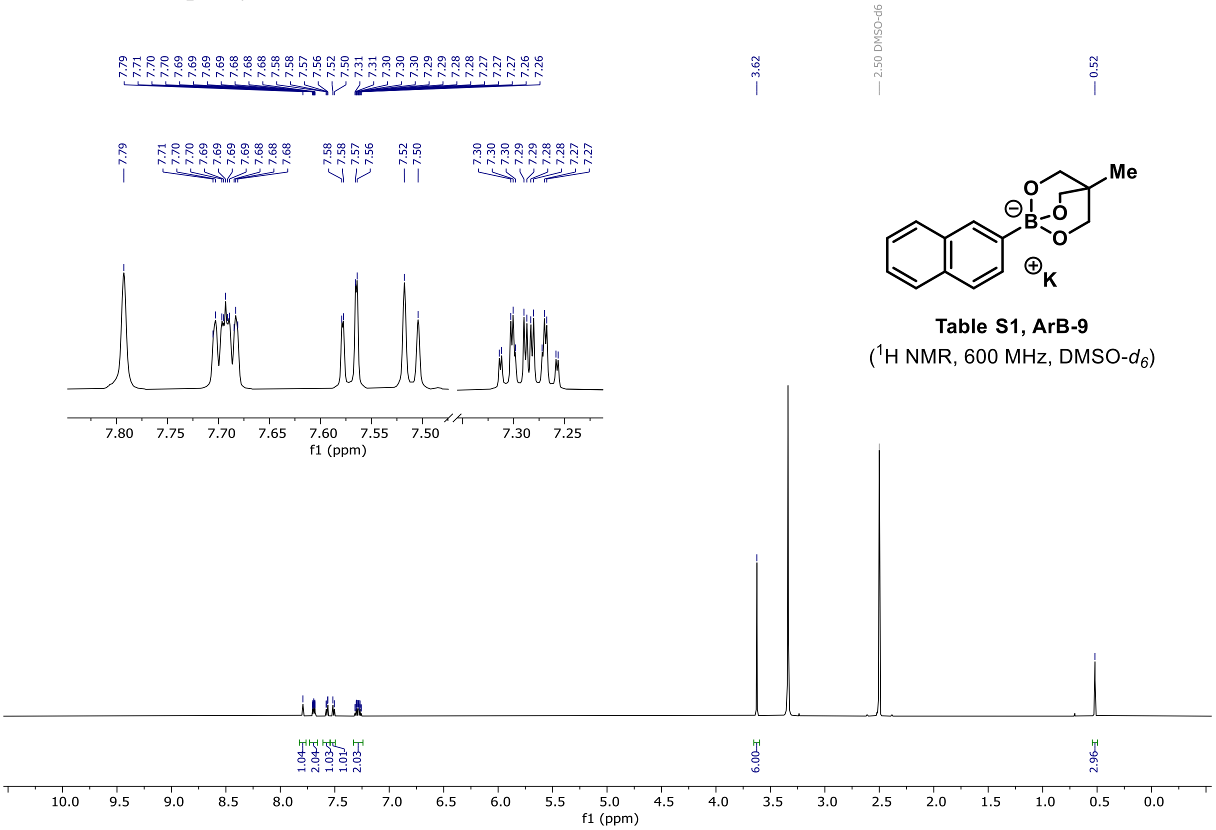

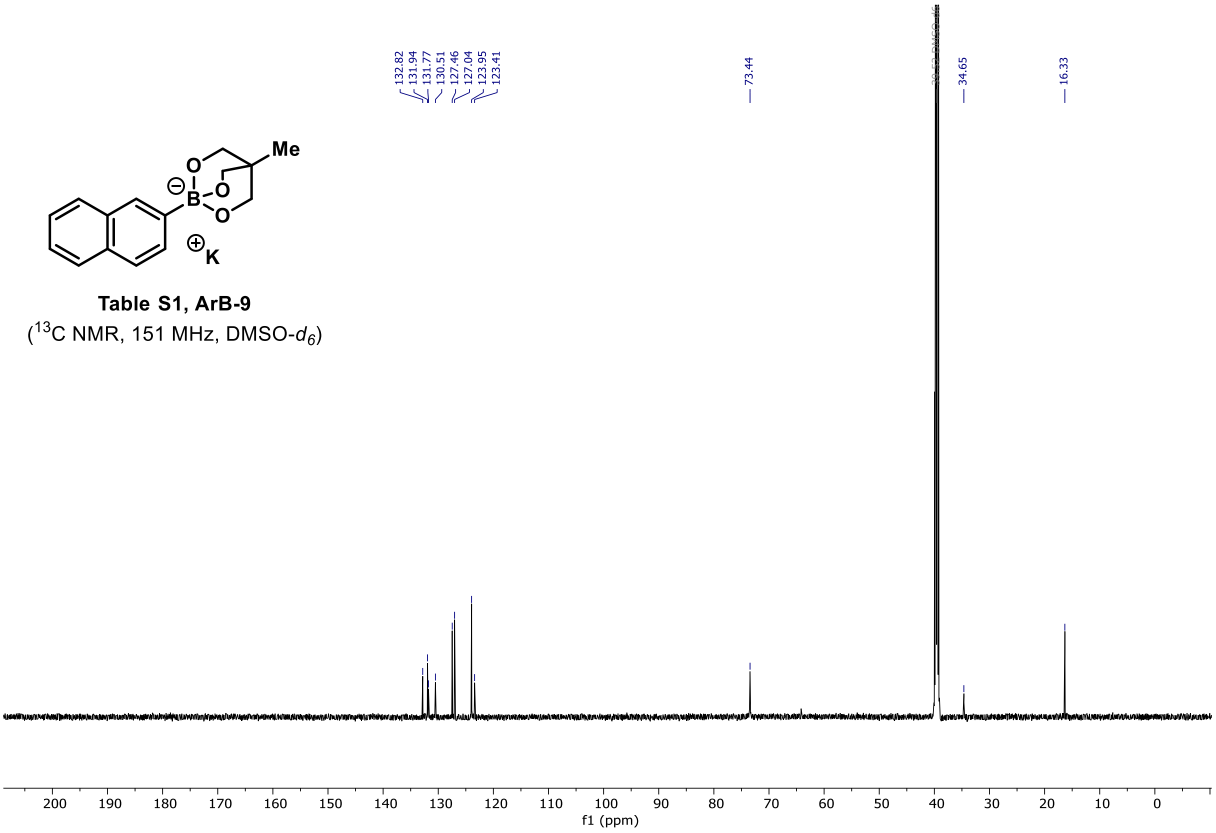

Potassium (thiophene-3-yl)triolborate (ArB-10)

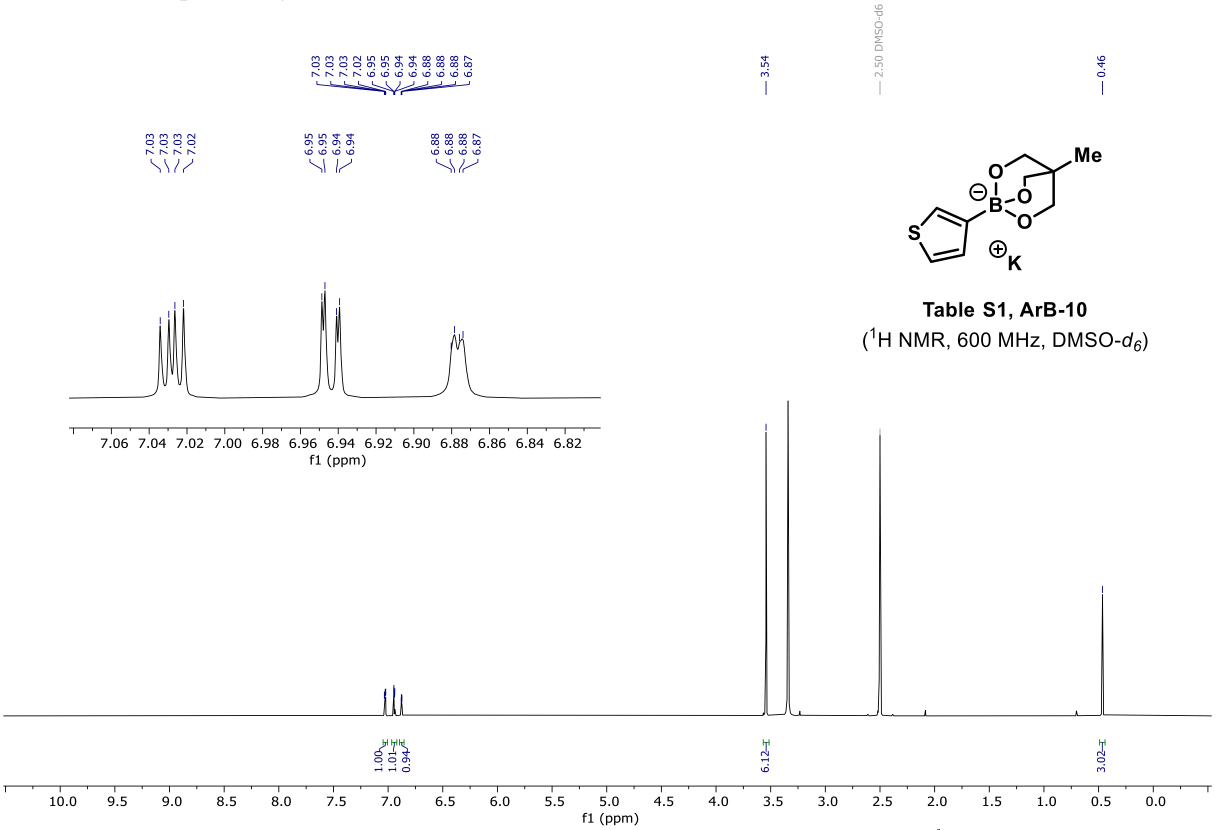

Table S1, ArB-10
(^{1}H NMR, 600 MHz, DMSO-d_6)

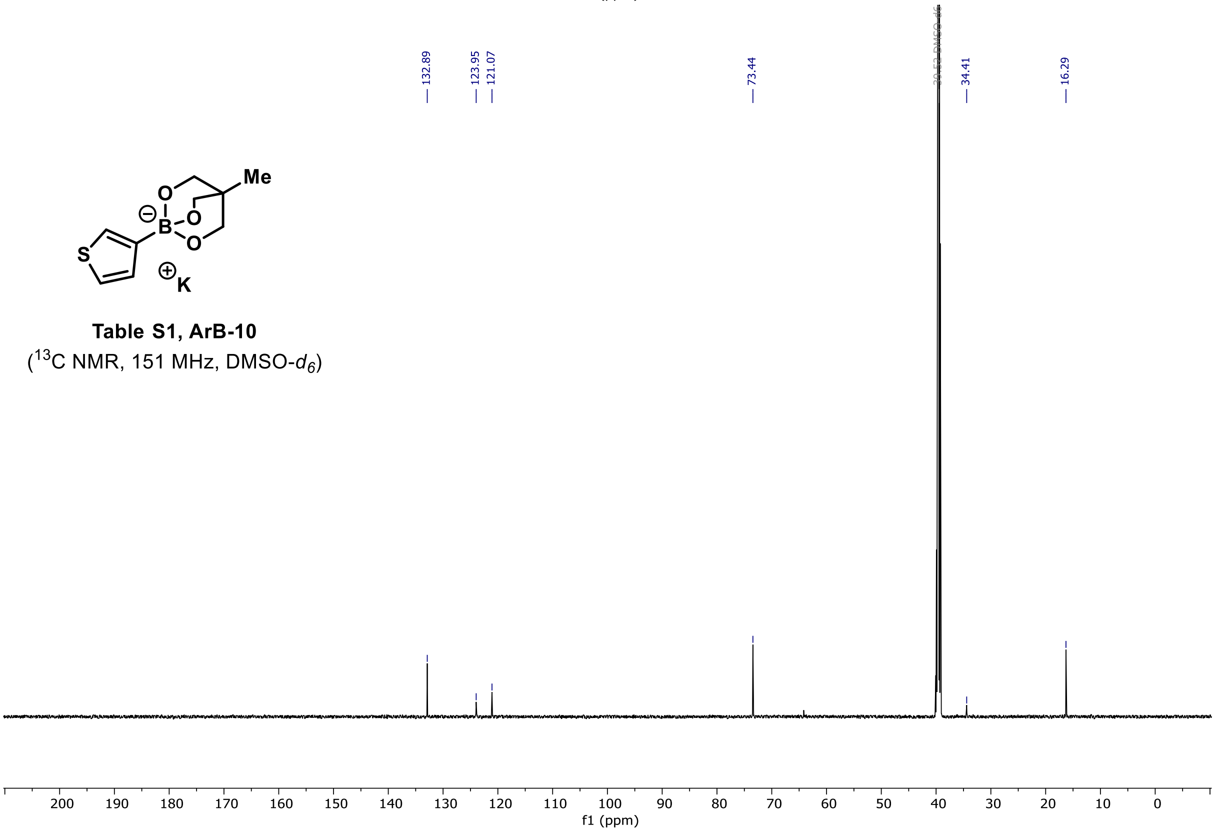

Table S1, ArB-10
(^{13}C NMR, 151 MHz, DMSO-d_6)

Potassium (thiophene-2-yl)triolborate (ArB-11)

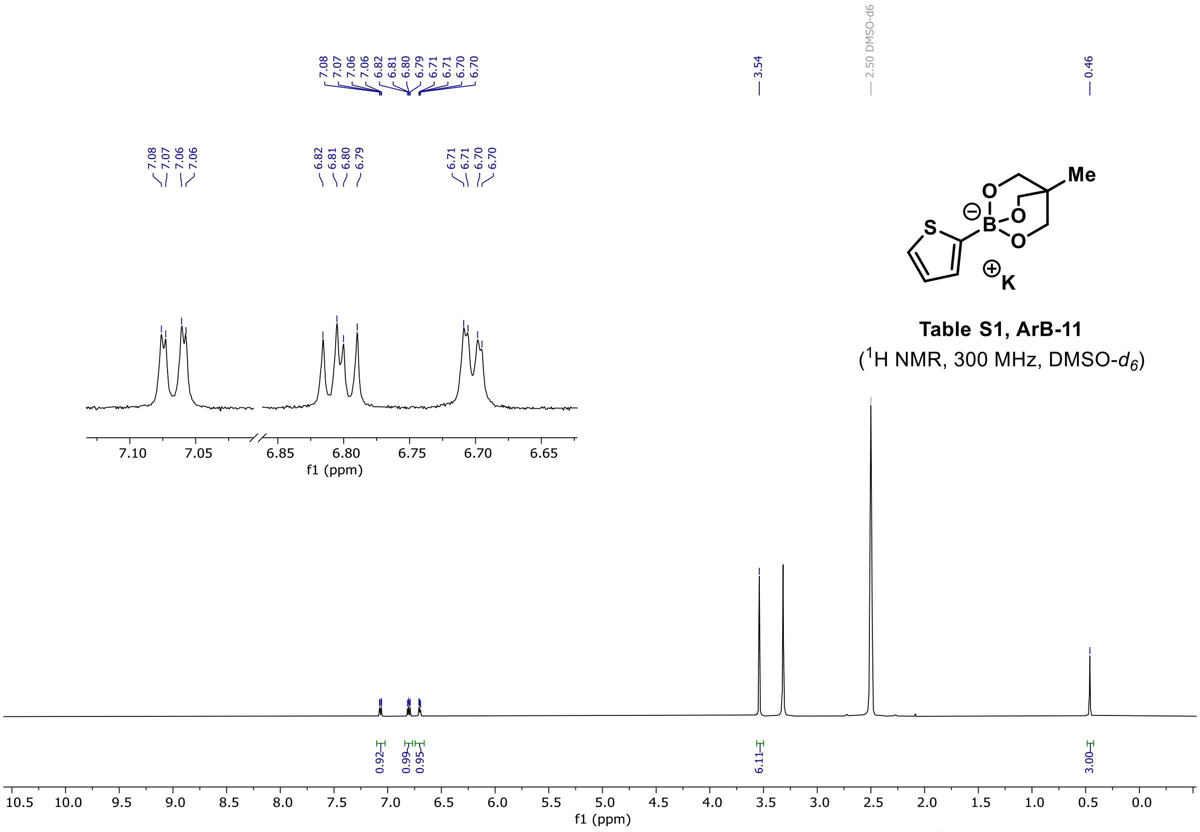

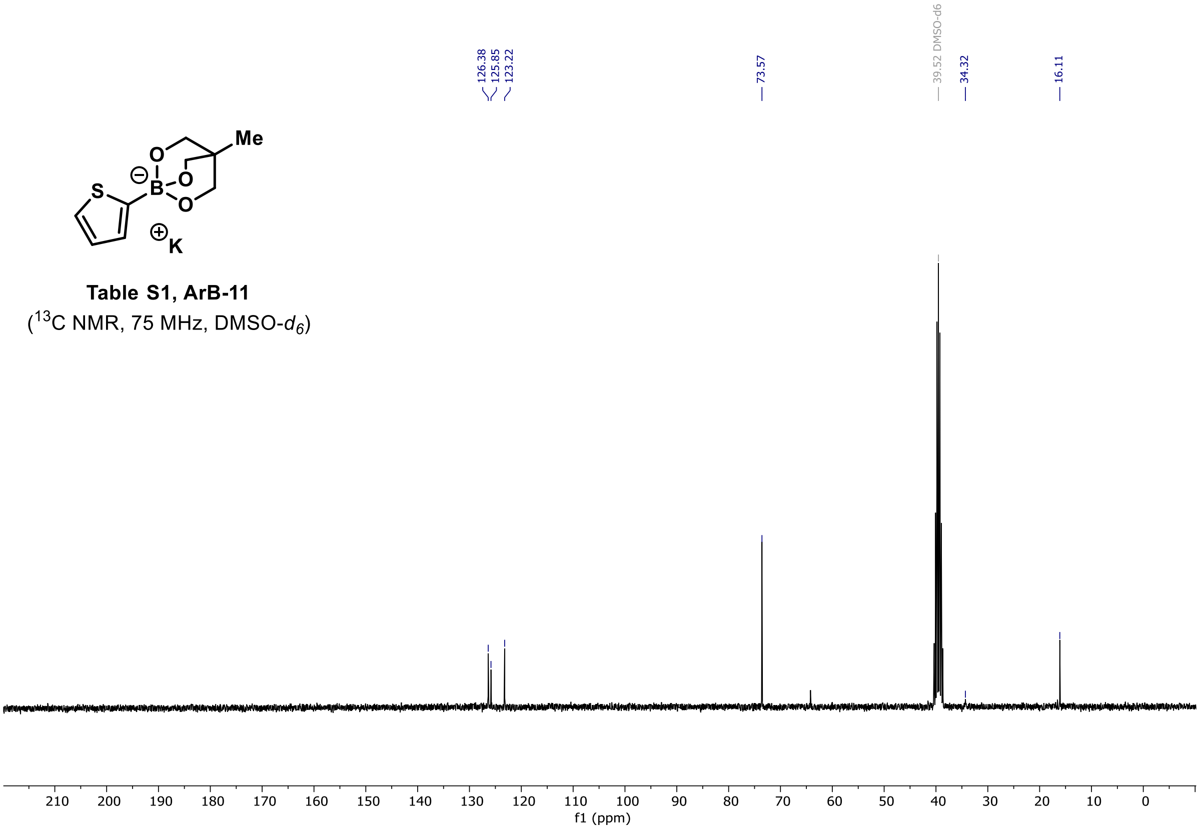

Potassium (benzo[b]thiophen-3-yl)triolborate (ArB-12)

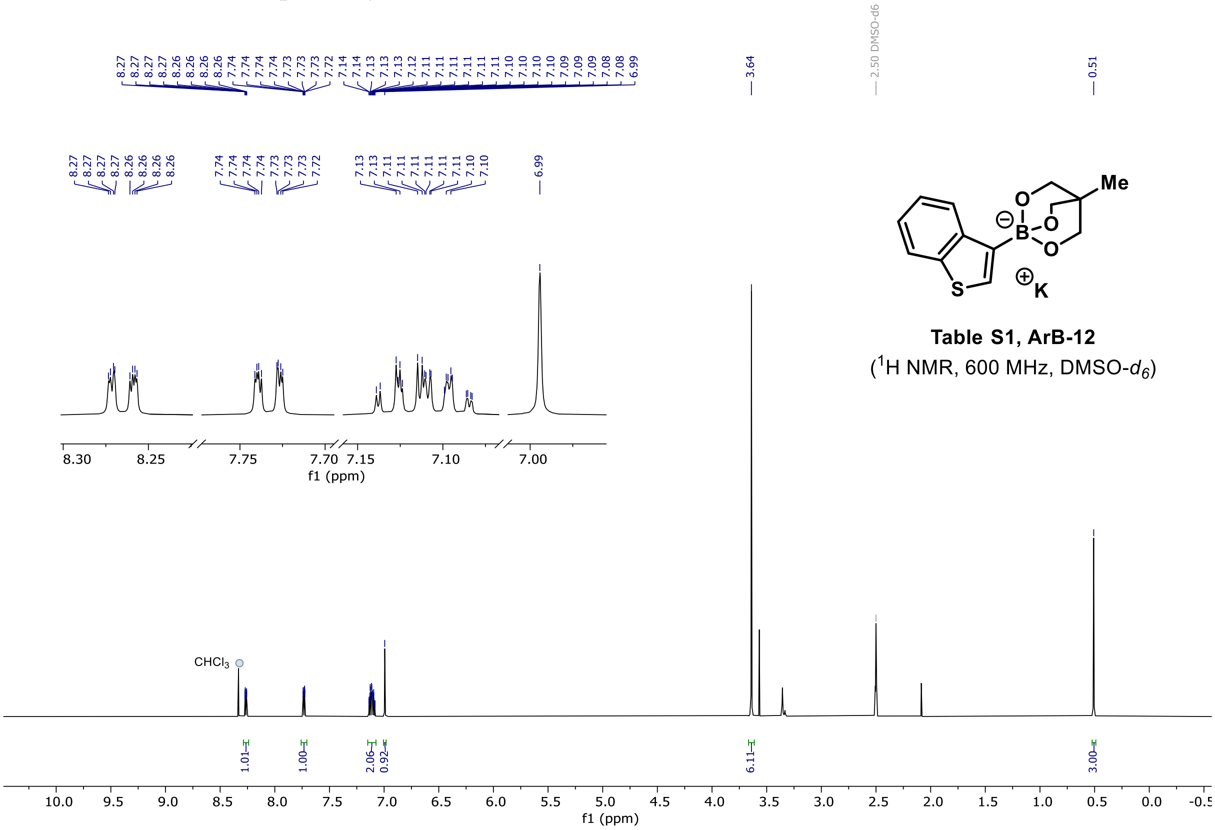

Table S1, ArB-12
(^{1}H NMR, 600 MHz, DMSO-d_6)

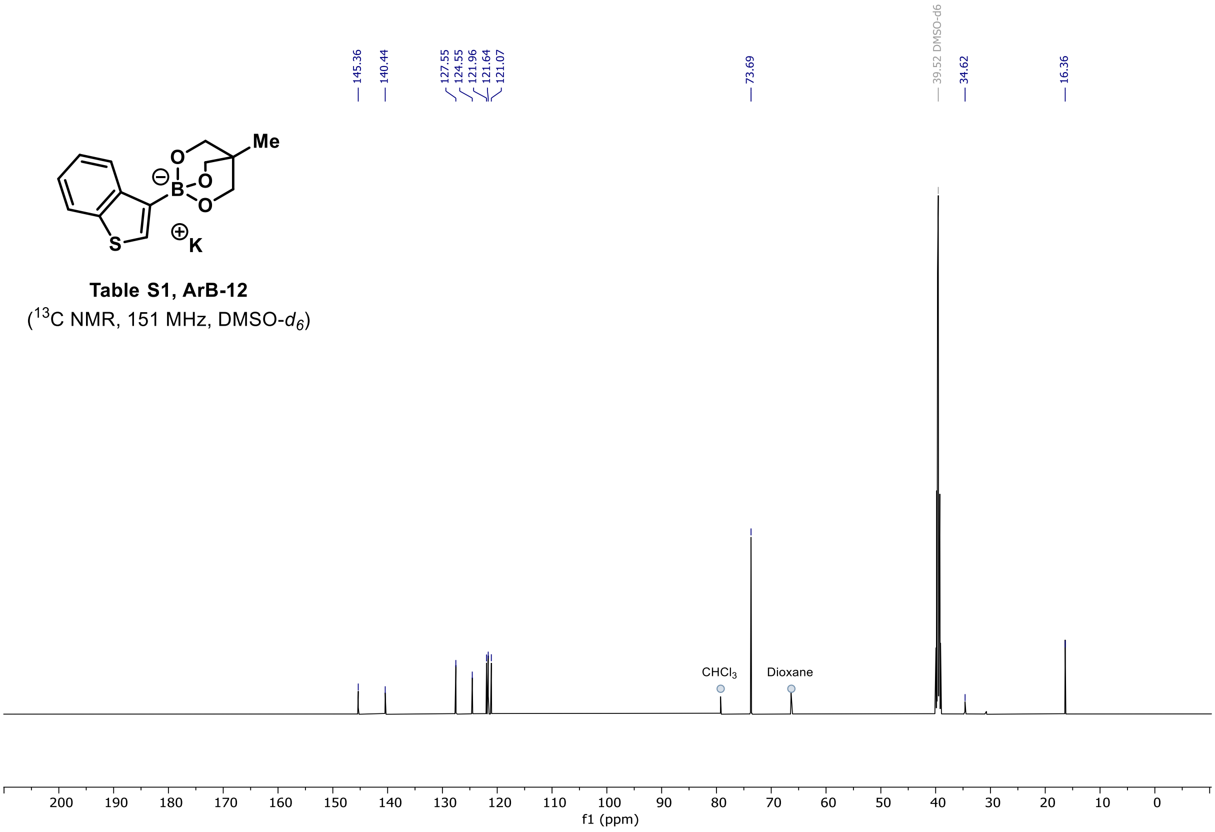

Table S1, ArB-12
(^{13}C NMR, 151 MHz, DMSO-d_6)

Potassium (benzo[d]thiazol-6-yl)triolborate (ArB-13)

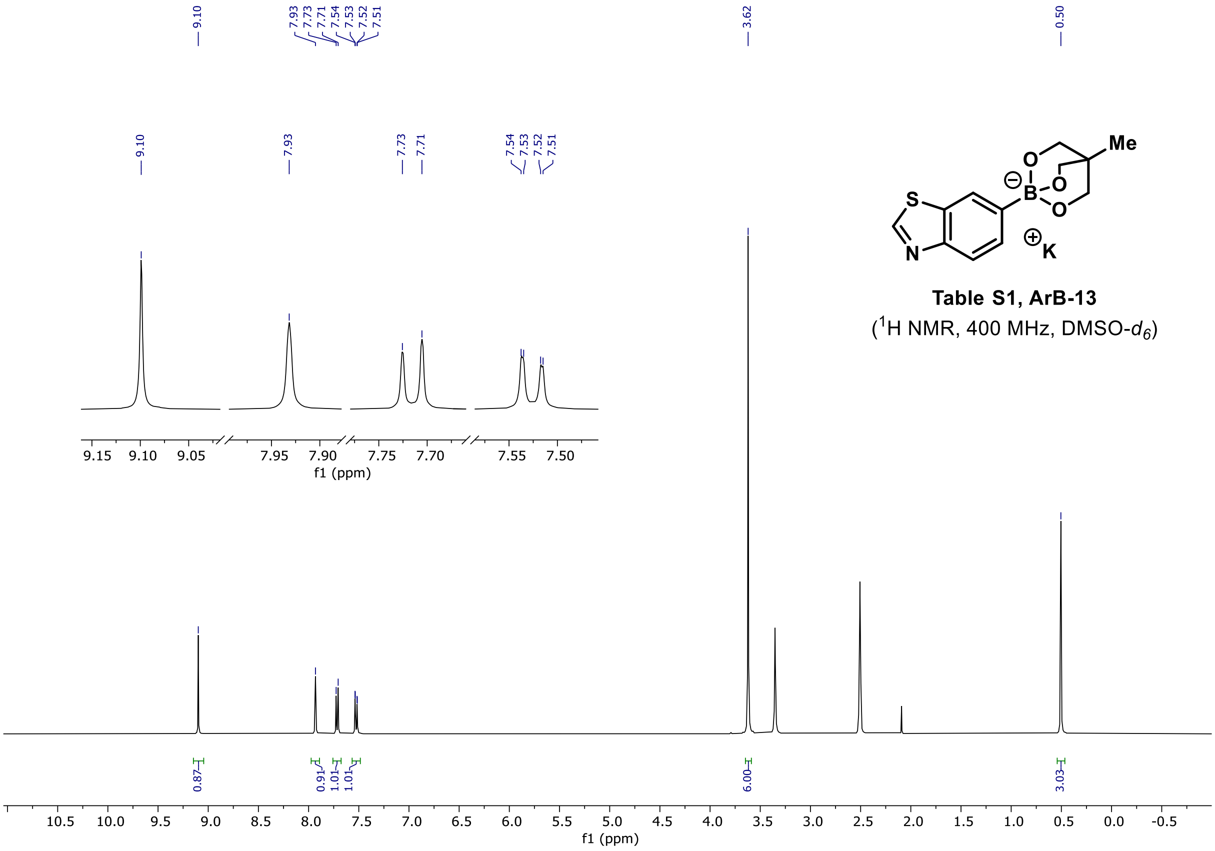

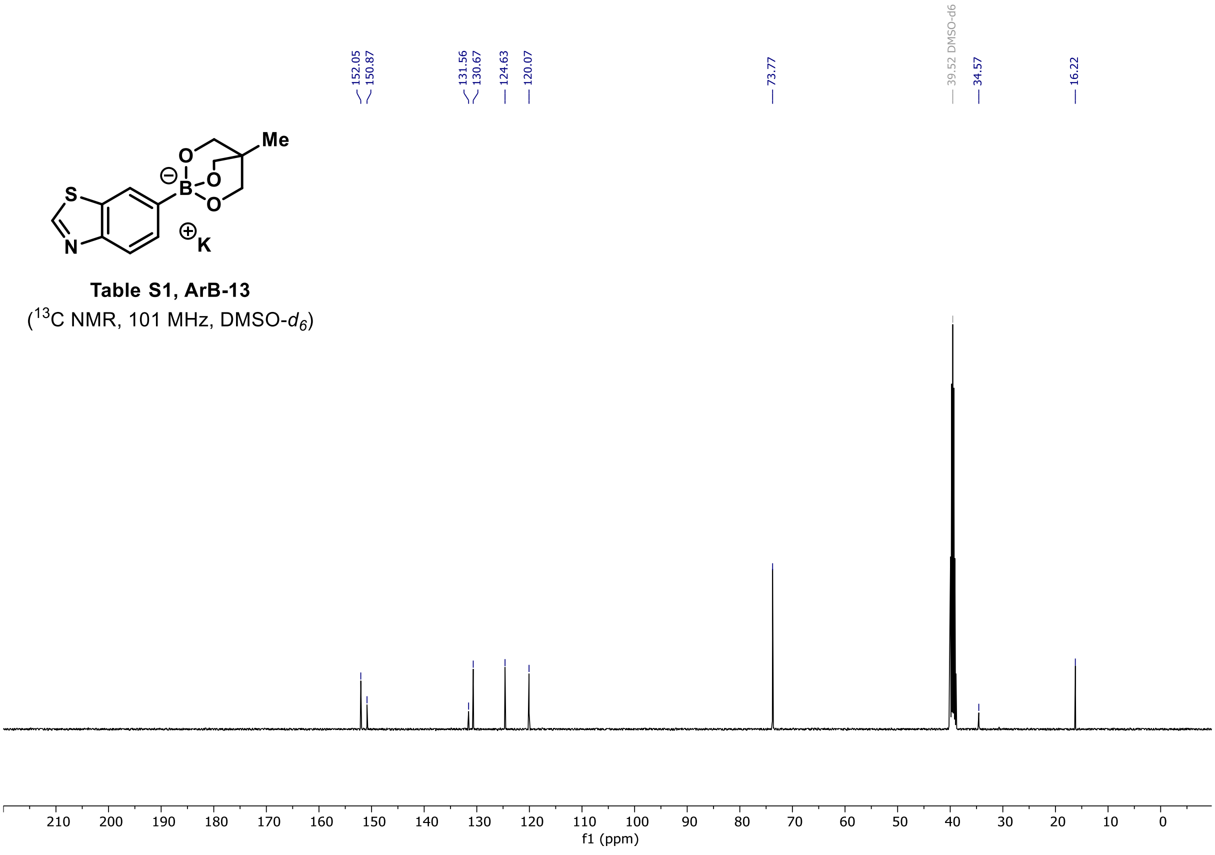

Potassium (quinolin-6-yl)triolborate (ArB-14)

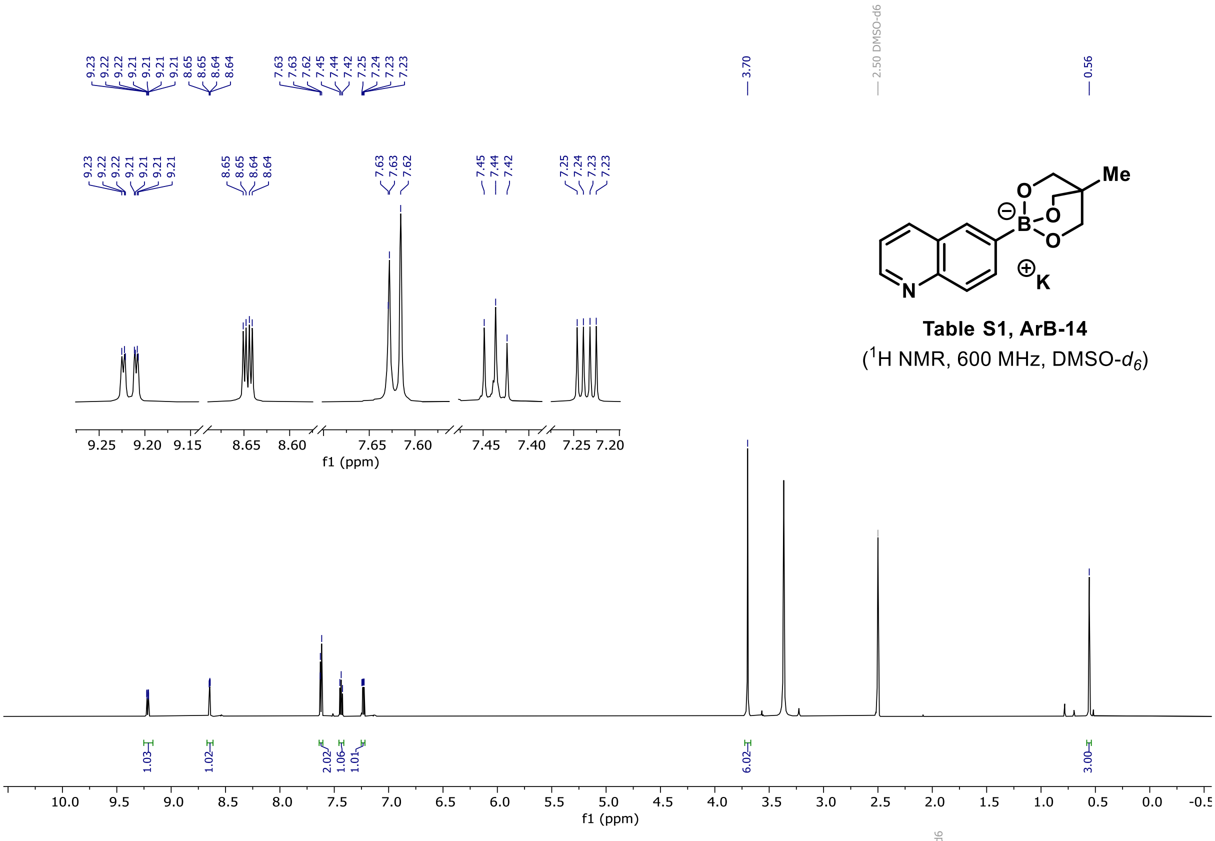

Table S1, ArB-14
(^1H NMR, 600 MHz, DMSO-d_6)

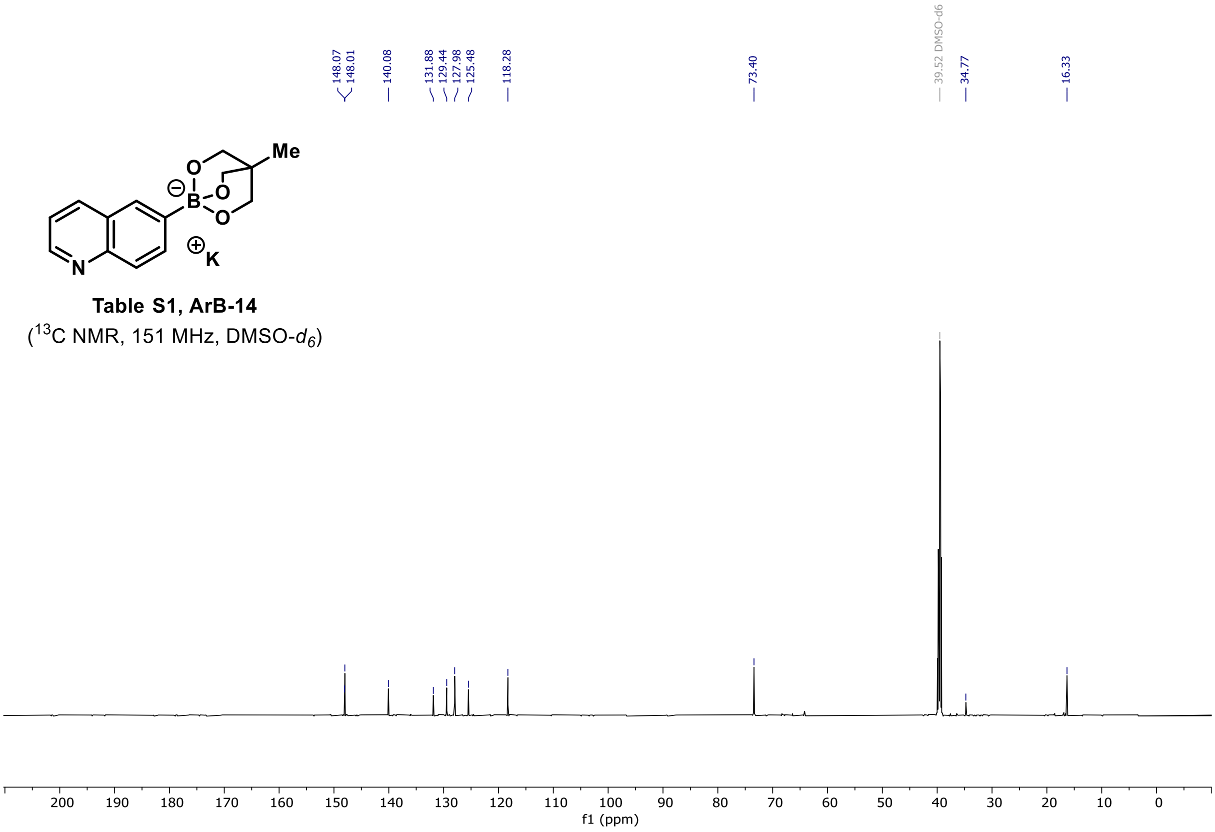

Table S1, ArB-14
(^{13}C NMR, 151 MHz, DMSO-d_6)

Potassium (4-(pyridin-4-yl)phenyl)triolborate (ArB-15)

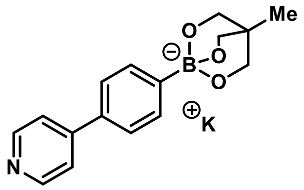

Table S1, ArB-15
(^{1}H NMR, 400 MHz, DMSO-d_6)

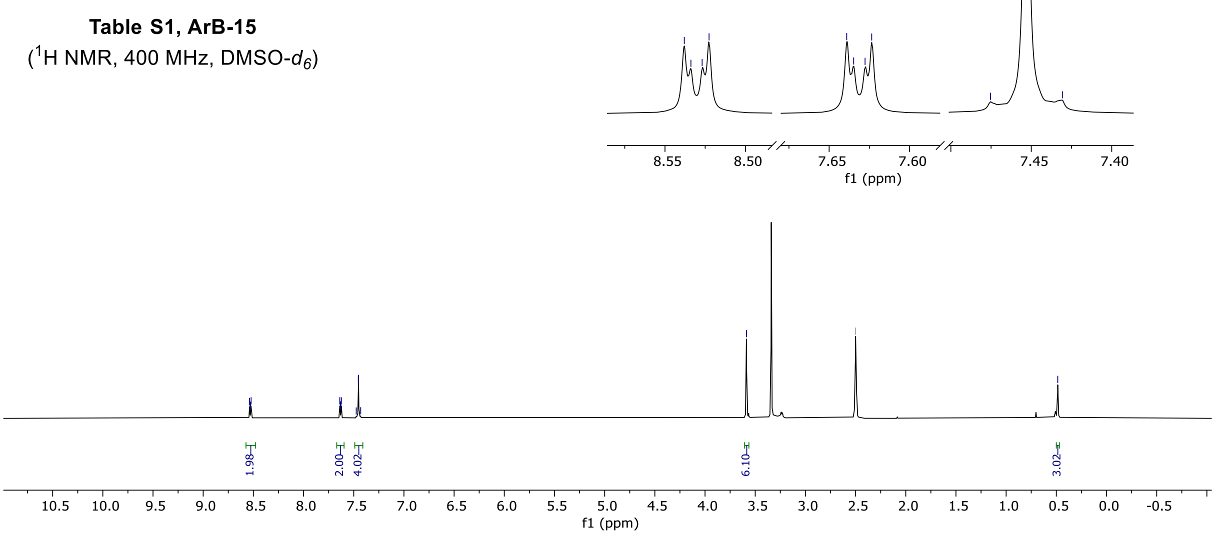

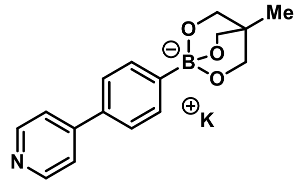

Table S1, ArB-15
(^{13}C NMR, 101 MHz, DMSO-d_6)

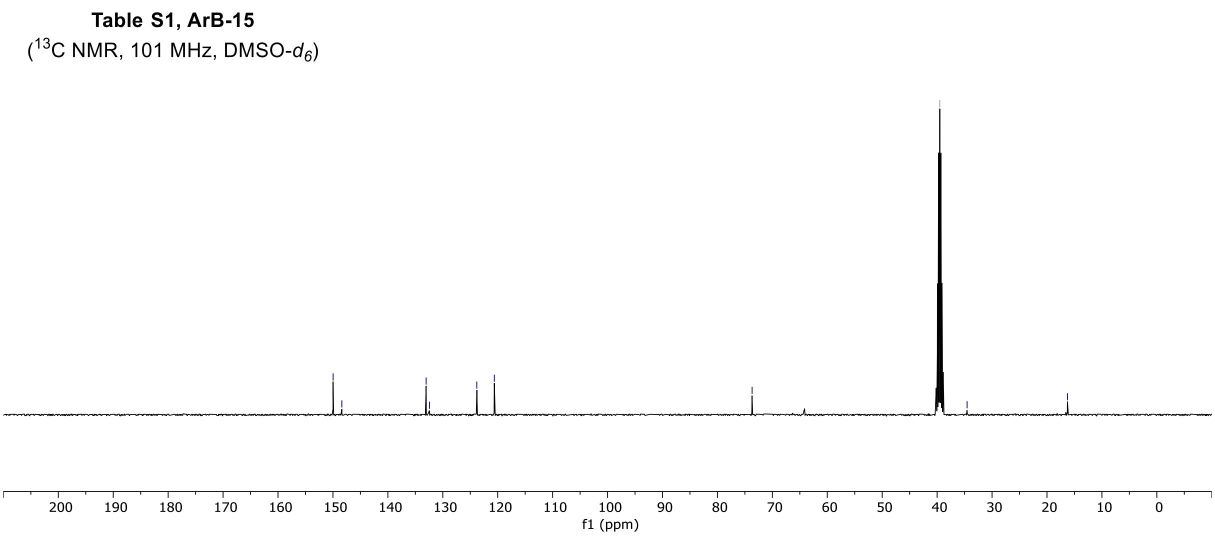

Potassium (1H-pyrazol-4-yl)triolborate (ArB-16)

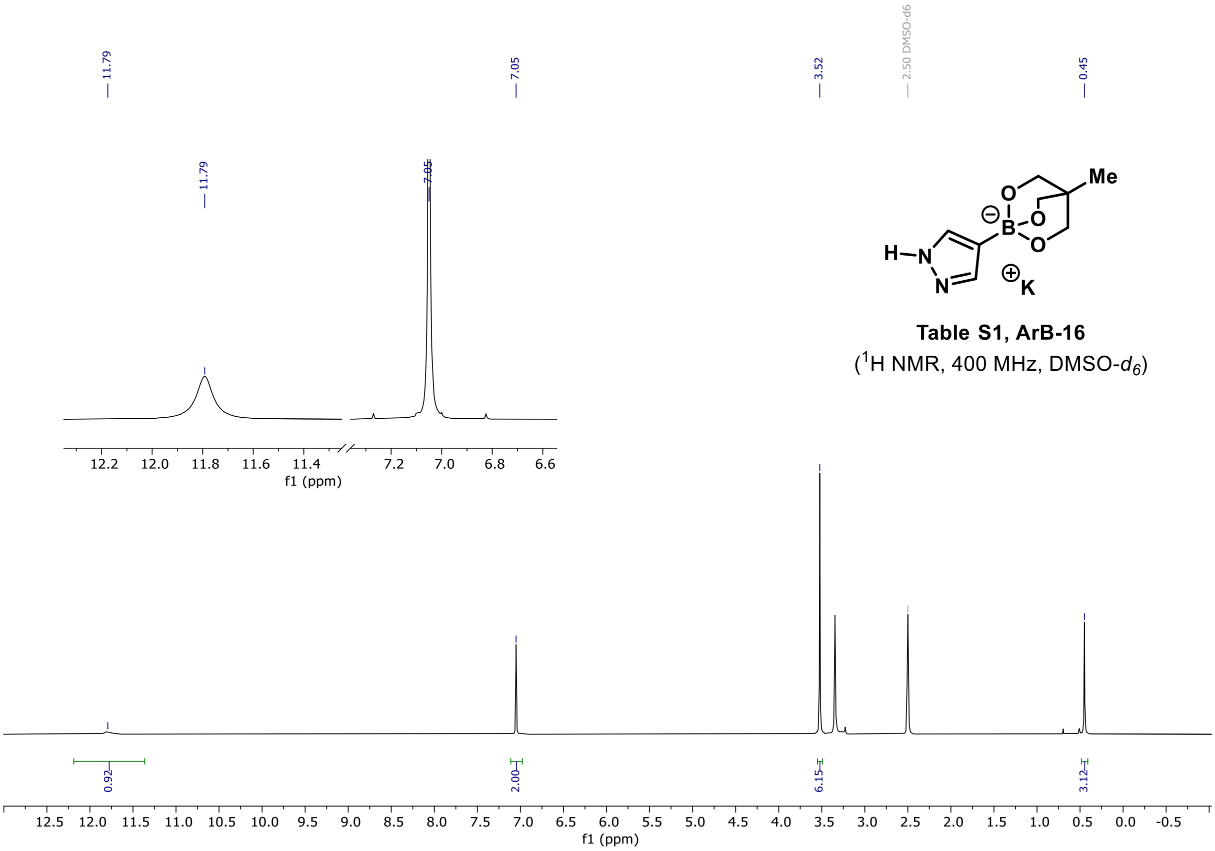

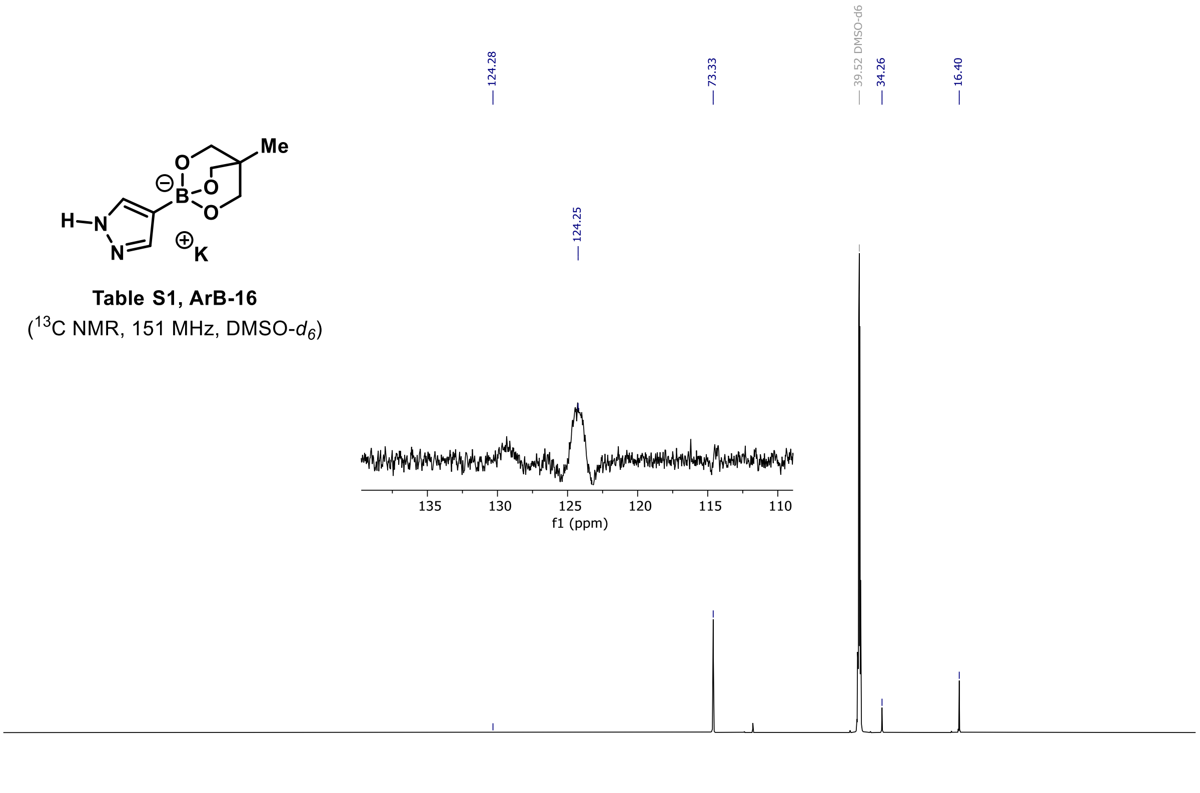

Potassium (1H-indol-5-yl)triolborate (ArB-17)

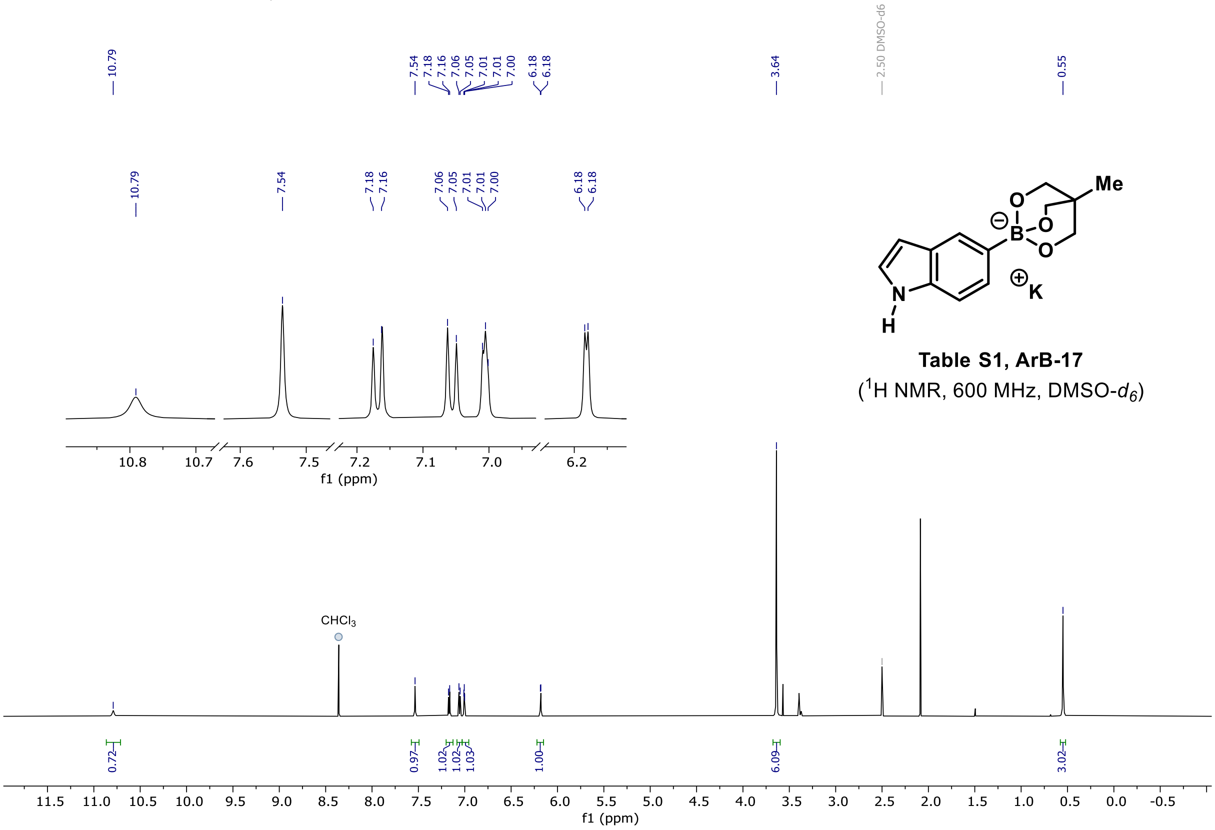

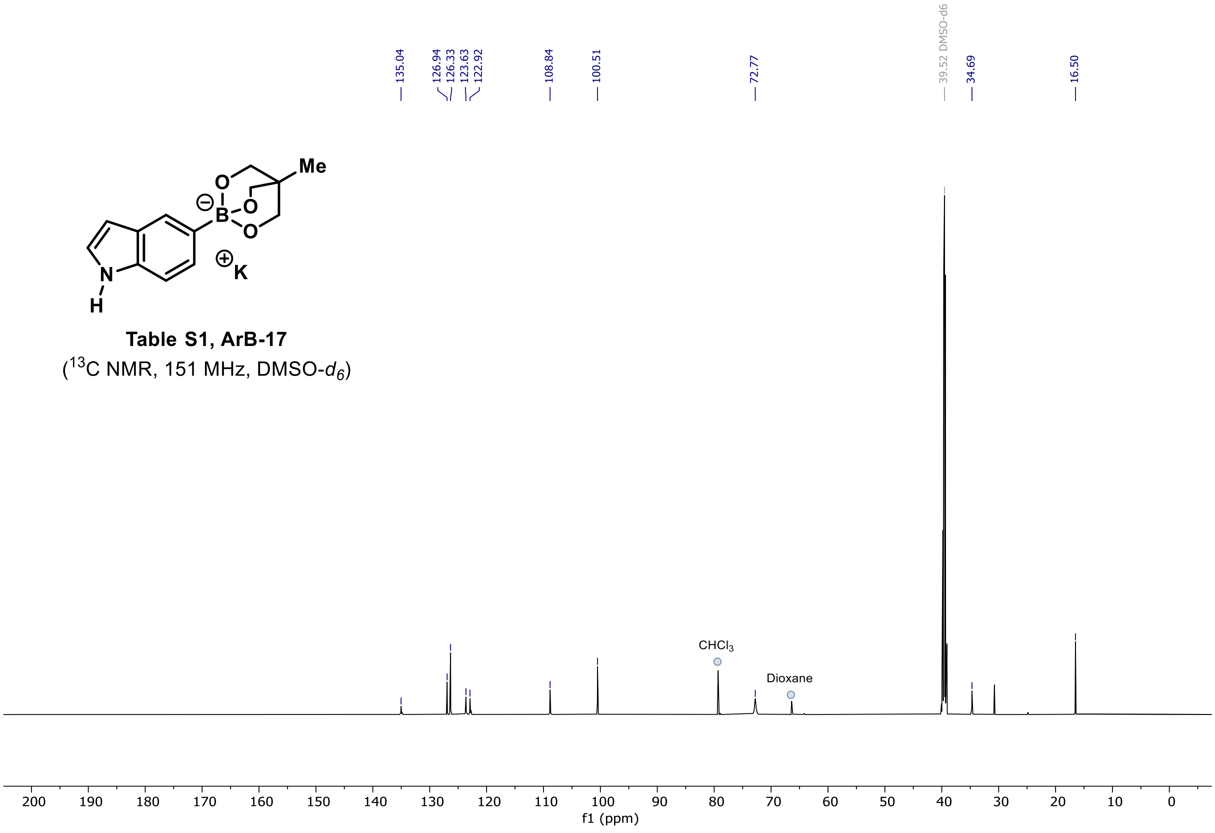

Potassium (3,5-dimethoxyphenyl)triolborate (ArB-20)

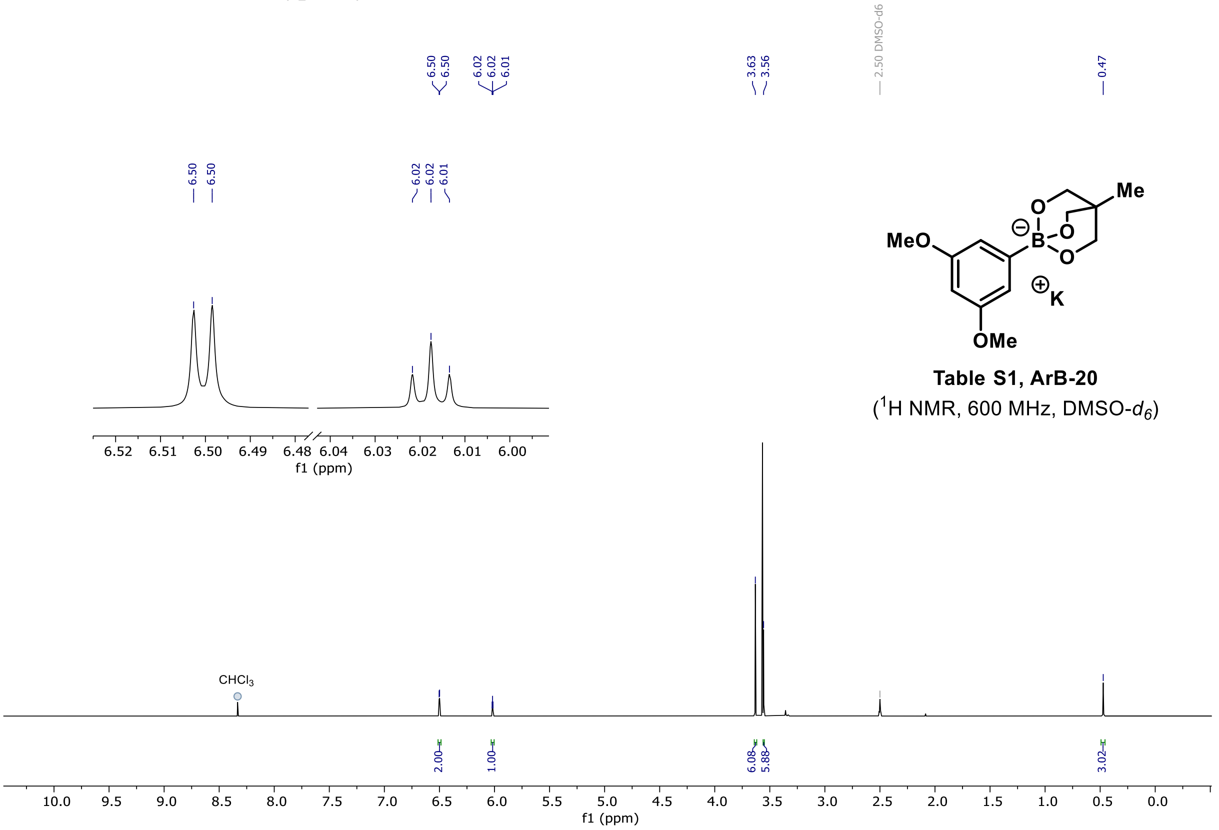

Table S1, ArB-20
(^{1}H NMR, 600 MHz, DMSO-d_6)

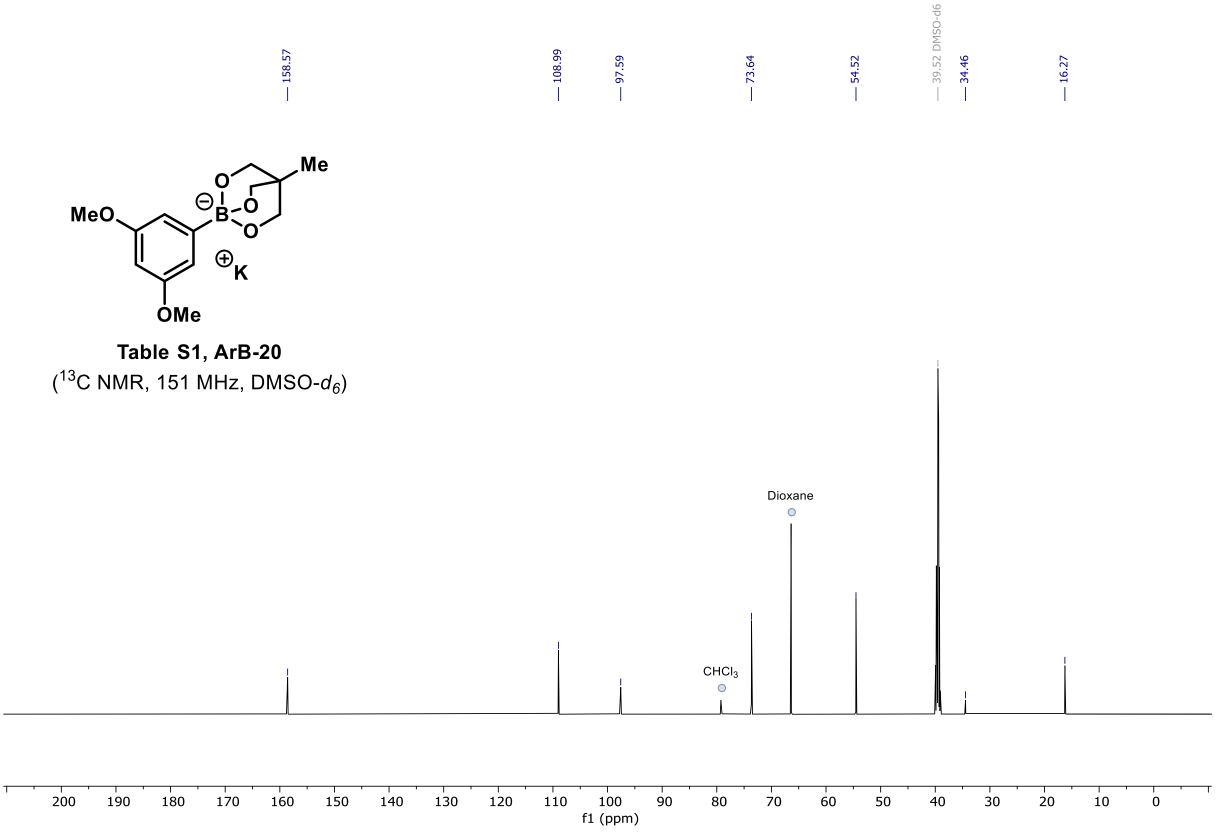

Table S1, ArB-20
(^{13}C NMR, 151 MHz, DMSO-d_6)

[(2,6-(^{t}BuNCH)$_2$C$_6$H$_3$)Bi(phenyl)(bromide)] (ArBi(Br)-1)

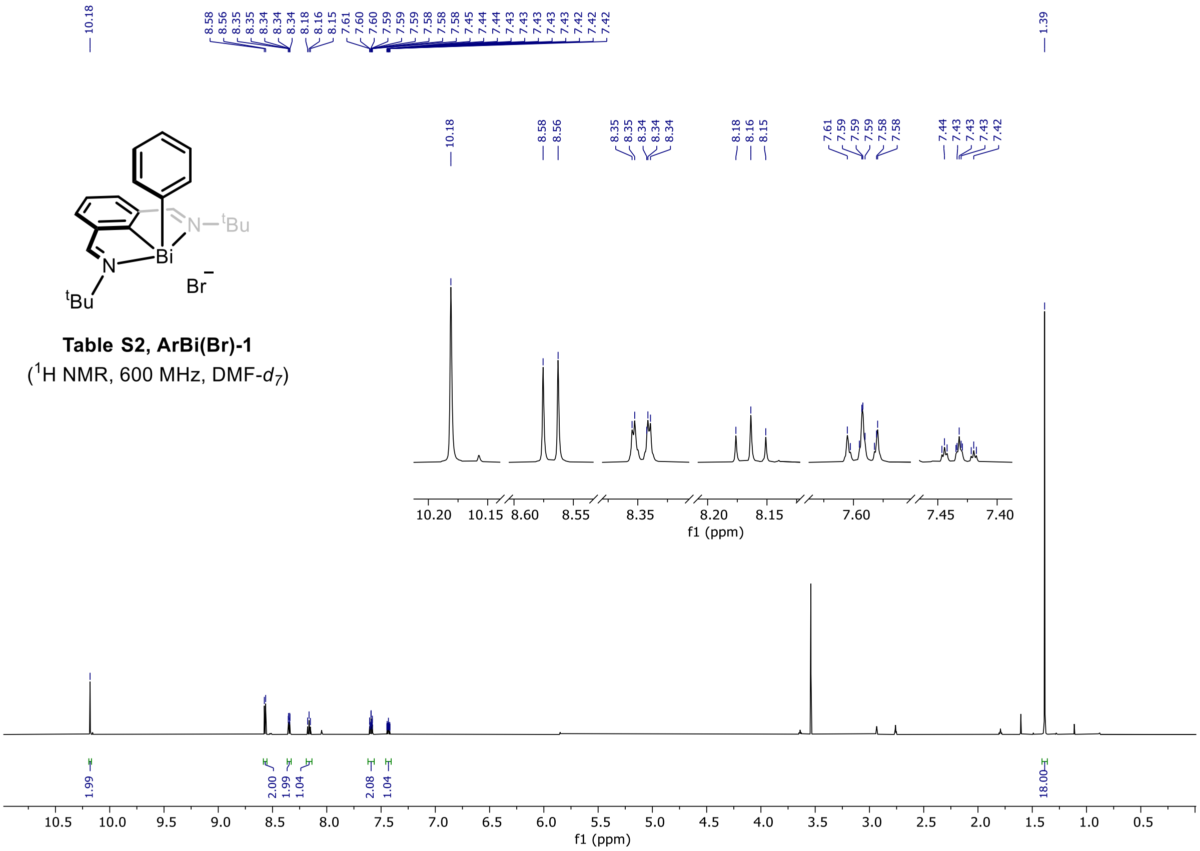

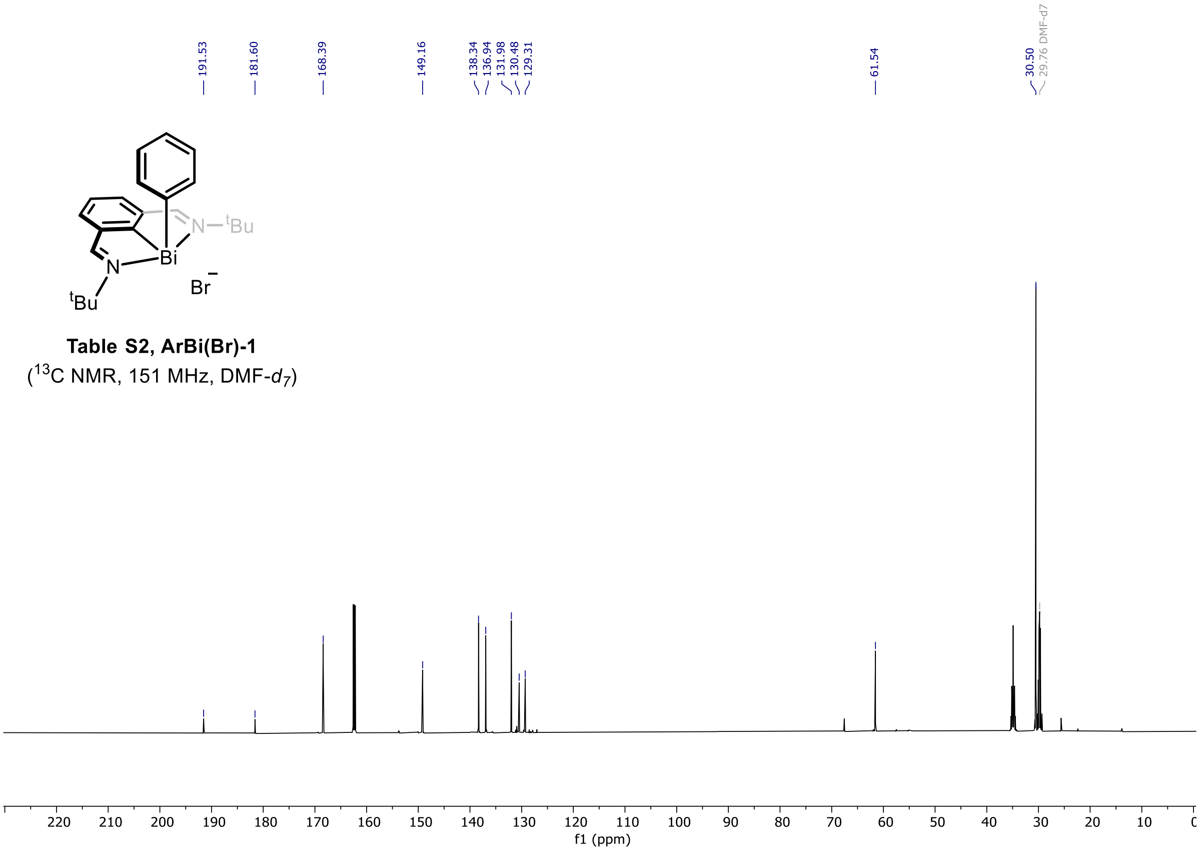

[(2,6-(^{t}BuNCH)$_2$C$_6$H$_3$)Bi(4-methoxyphenyl)(bromide)] (ArBi(Br)-2)

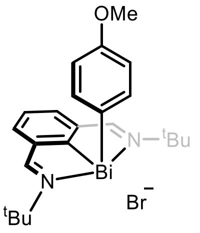

Table S2, ArBi(Br)-2
(^{1}H NMR, 600 MHz, DMF-d_7)

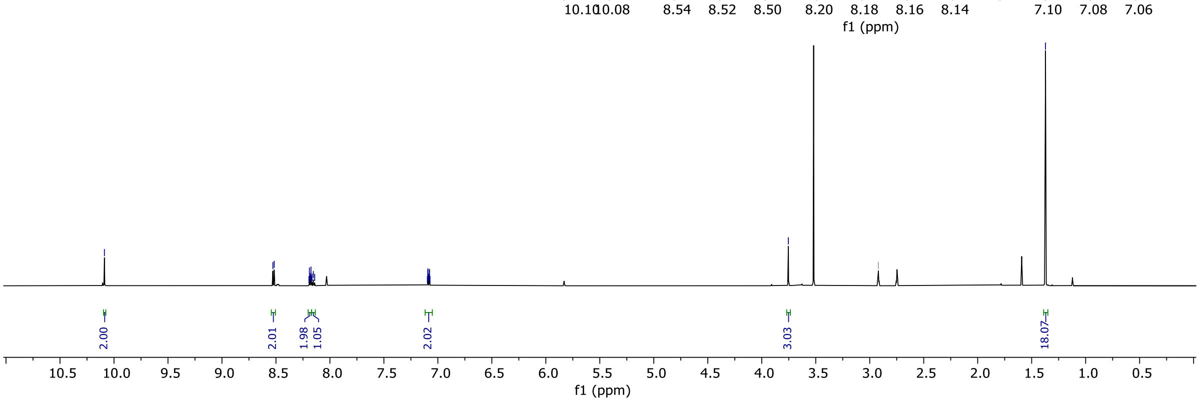

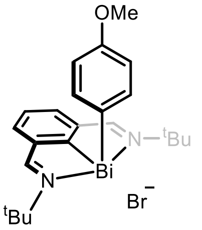

Table S2, ArBi(Br)-2
(^{13}C NMR, 151 MHz, DMF-d_7)

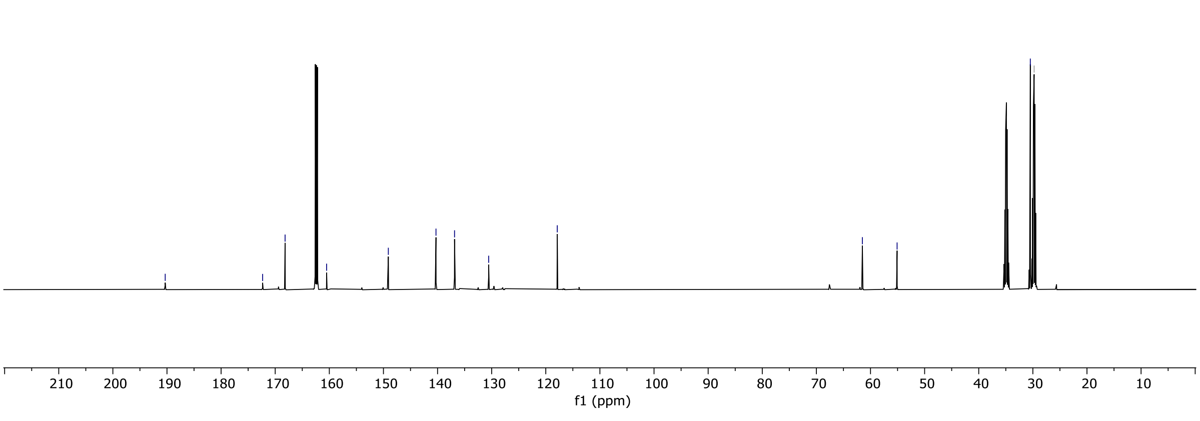

[(2,6-(^{t}BuNCH)$_2$C$_6$H$_3$)Bi(4-trifluoromethylphenyl)(iodide)] (ArBi(I)-1)

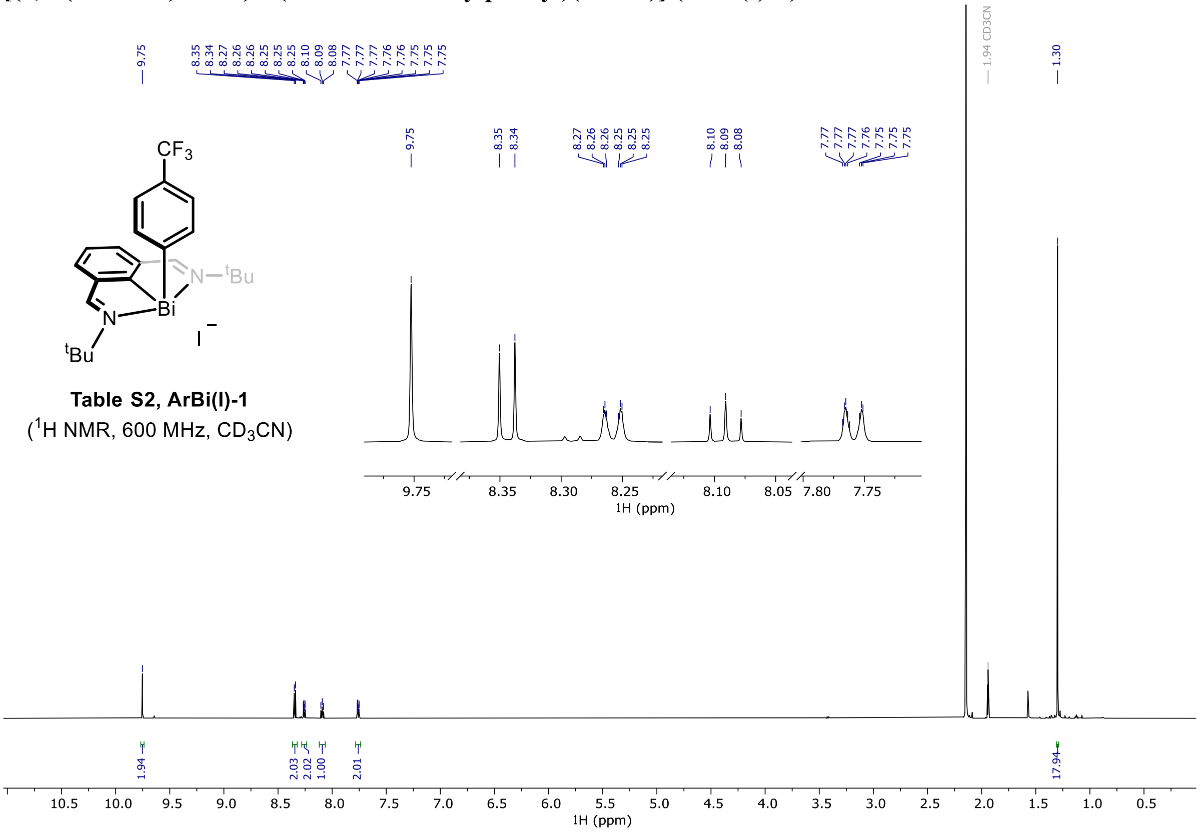

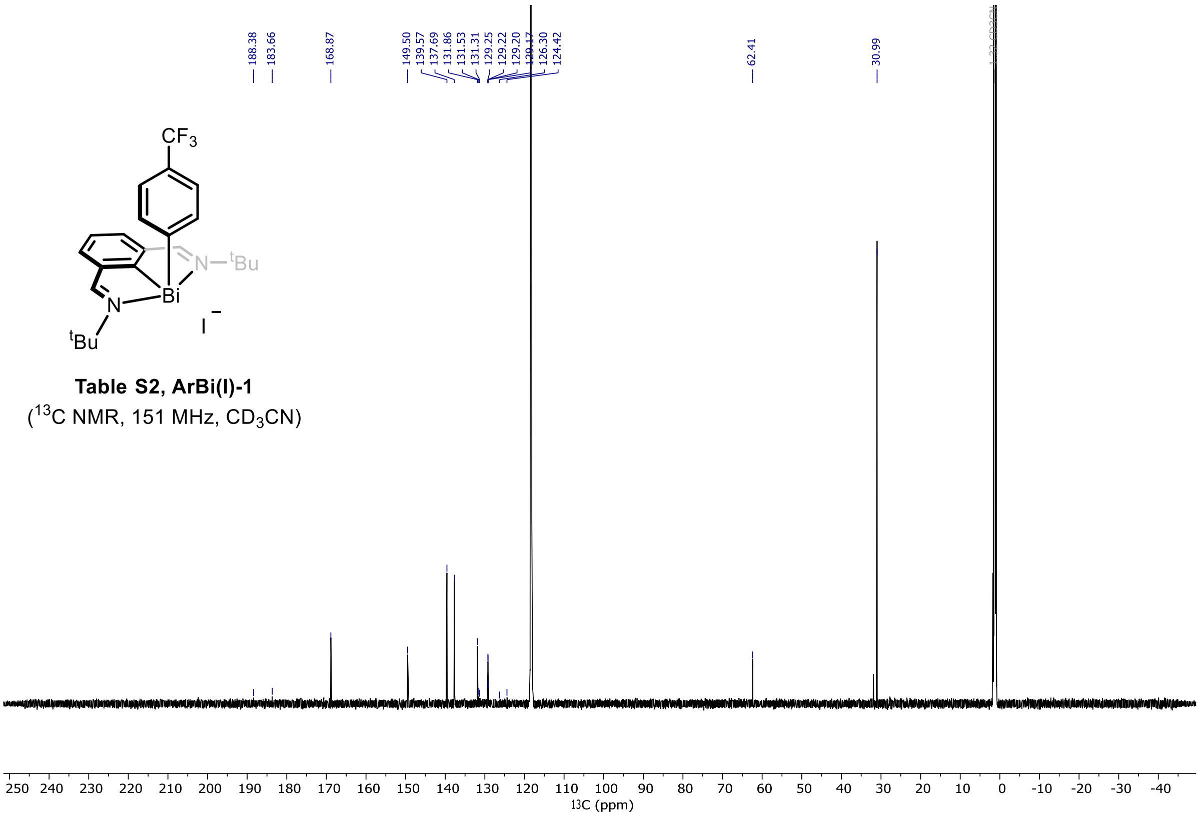

[(2,6-(^{t}BuNCH)$_2$C$_6$H$_3$)Bi(pyrazin-2-yl)(iodide)] (ArBi(I)-2)

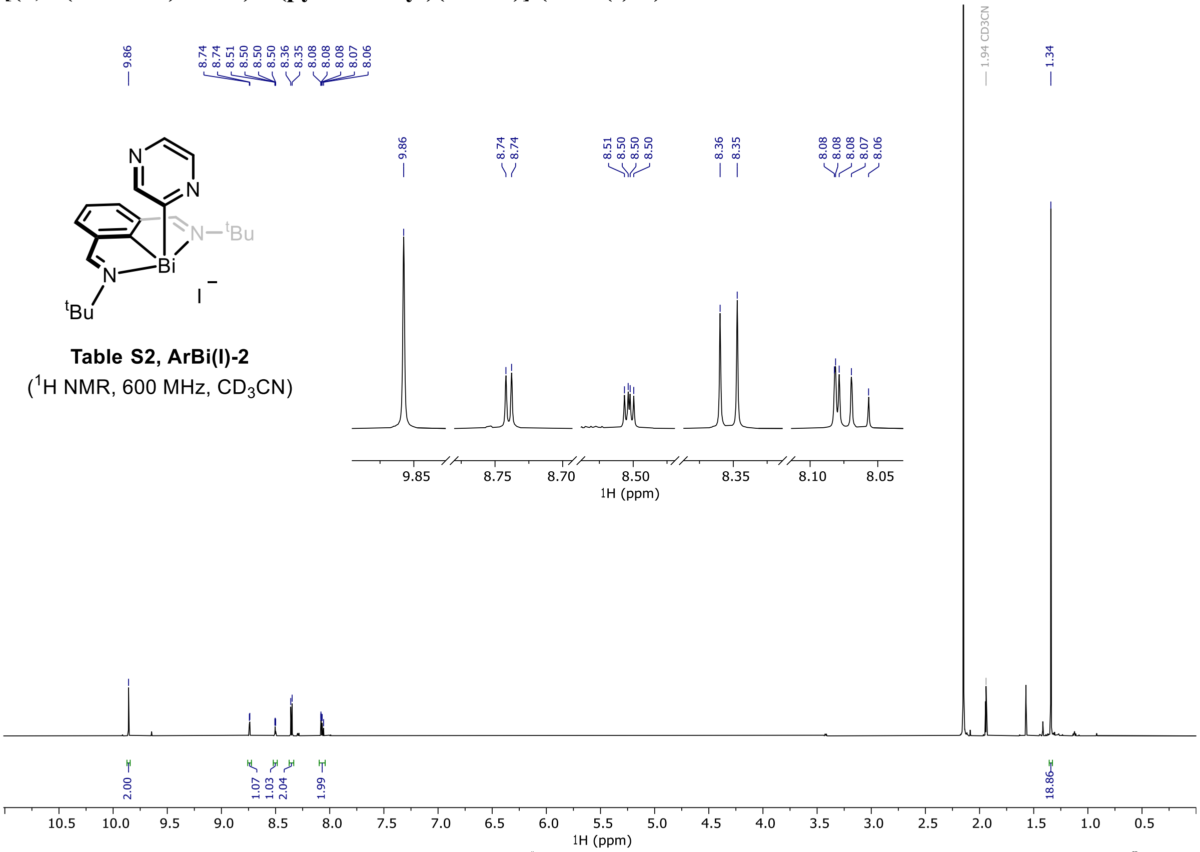

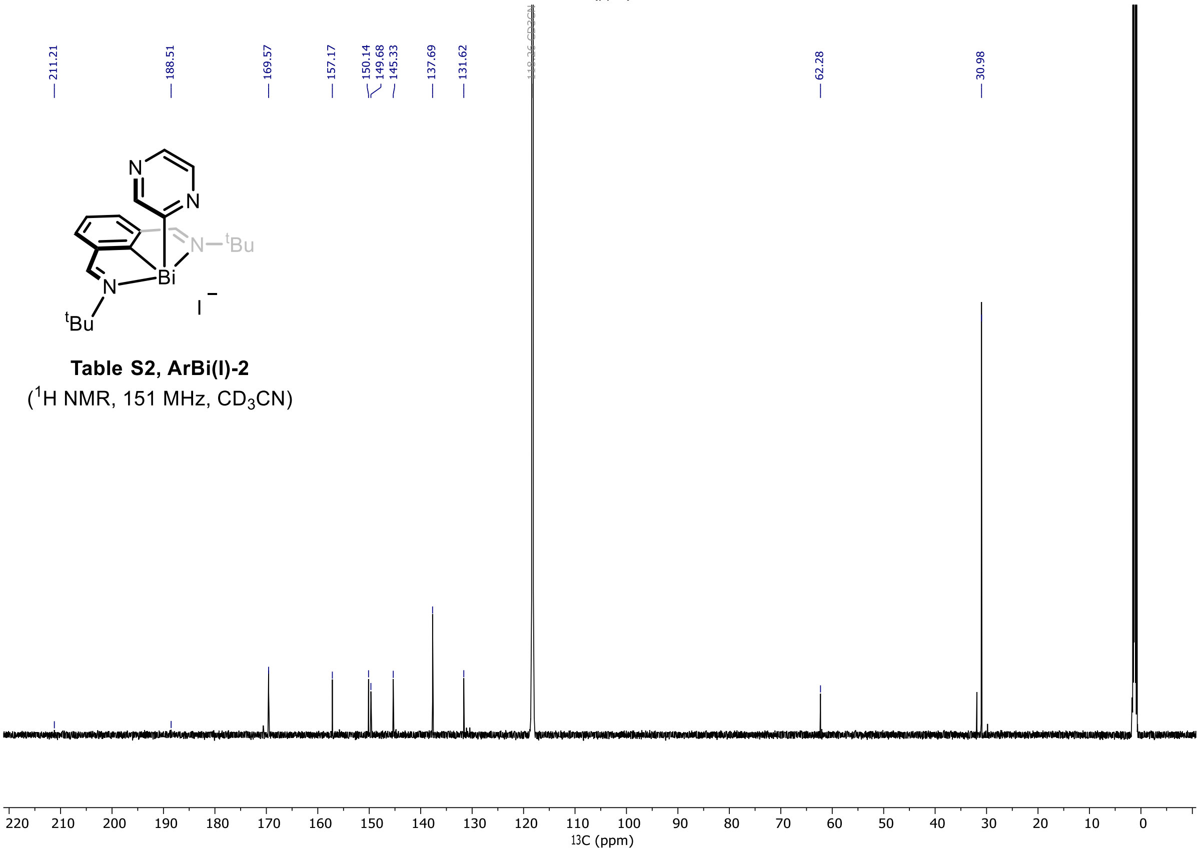

[(2,6-(^{t}BuNCH)$_2$C$_6$H$_3$)Bi(phenyl)(BF$_4$)] (ArBi(BF$_4$)-1)

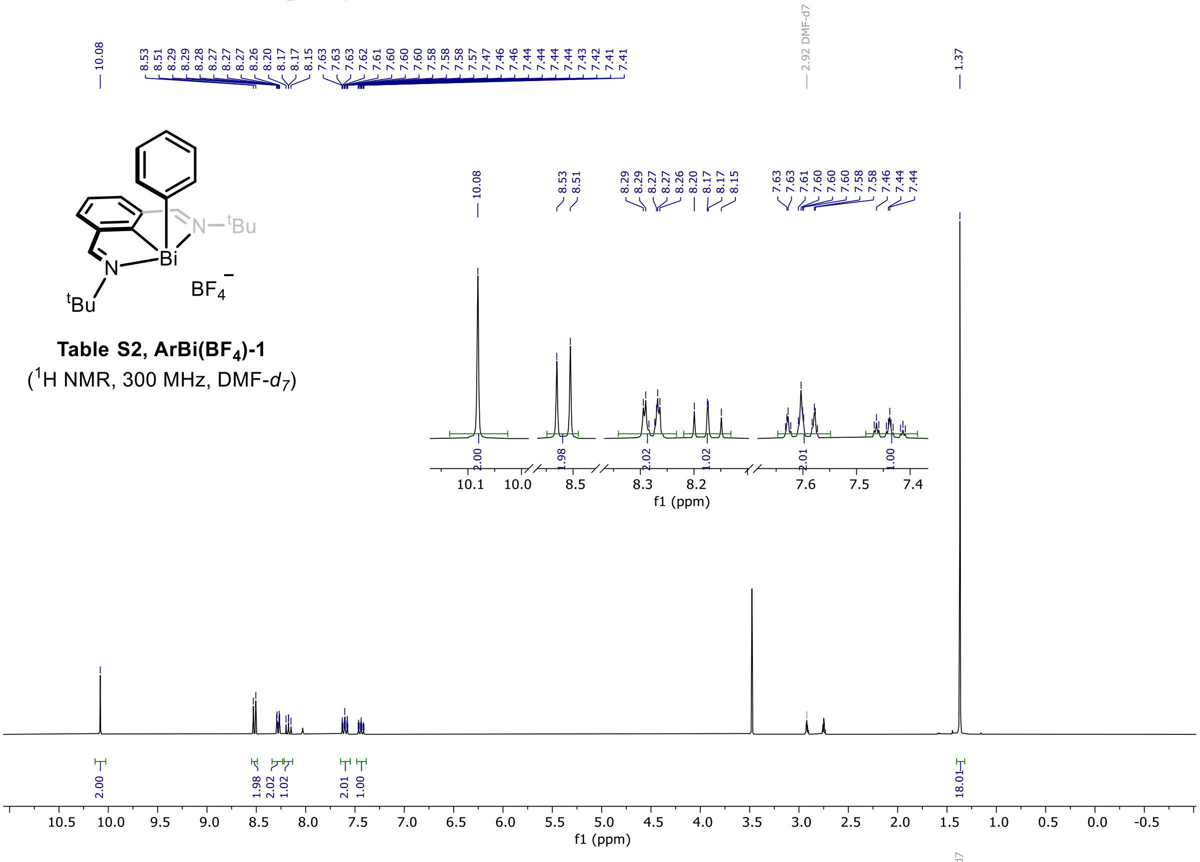

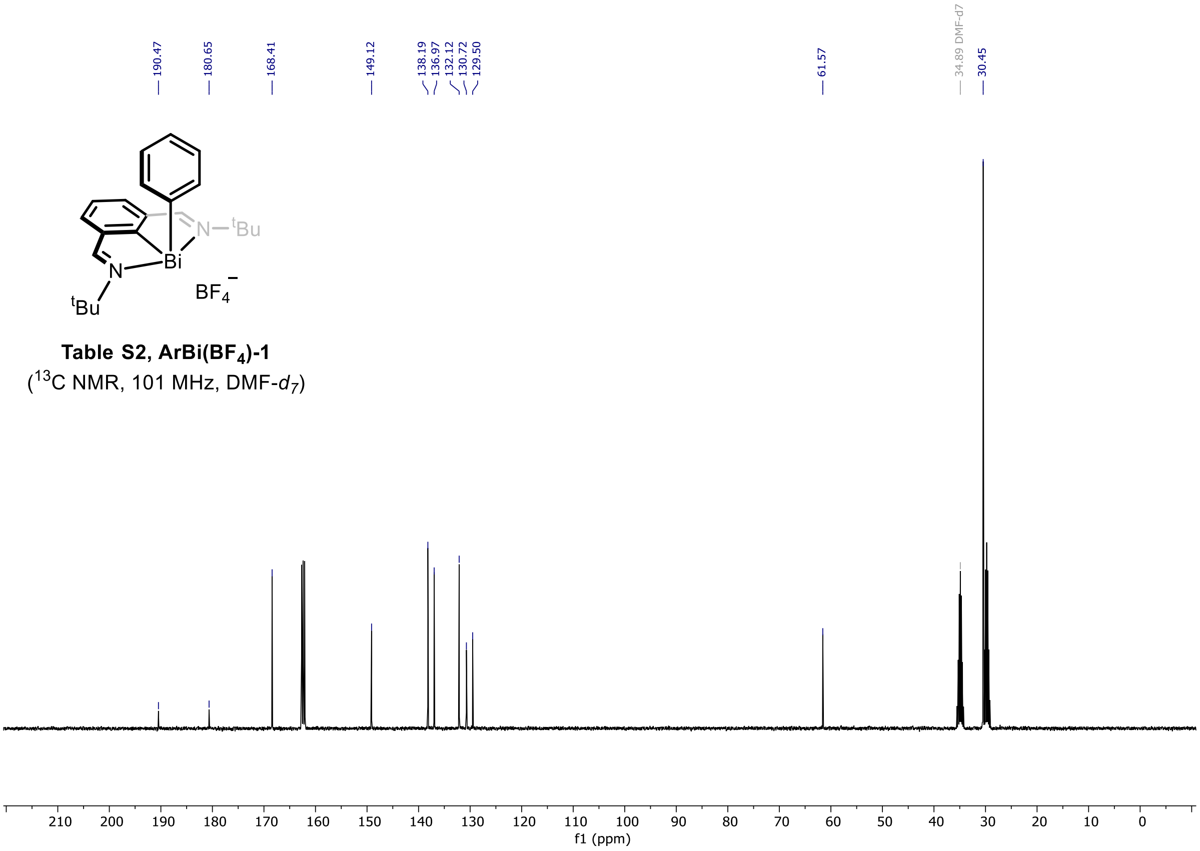

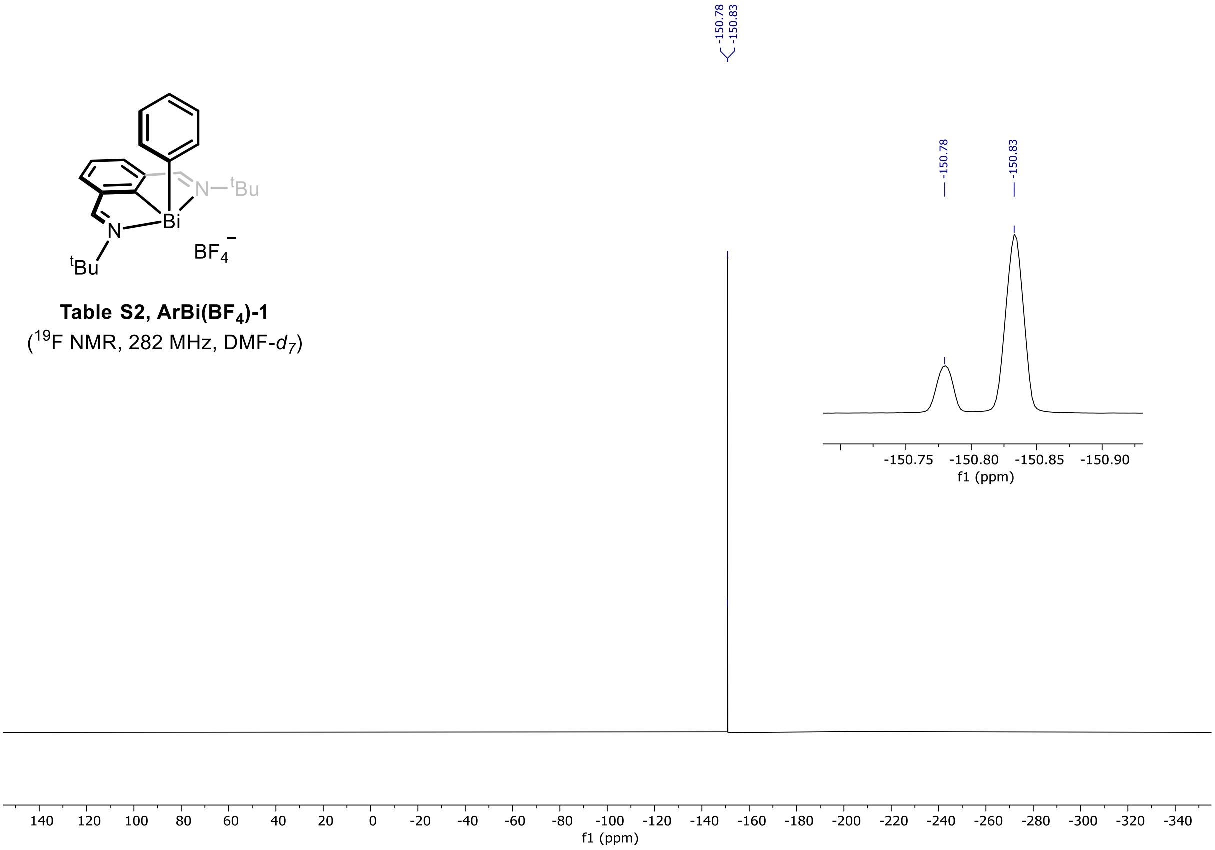

Table S2, ArBi(BF_4)-1
(^{19}F NMR, 282 MHz, DMF-d_7)

[(2,6-(^{t}BuNCH)$_2$C$_6$H$_3$)Bi(phenyl)(OTf)] (ArBi(OTf)-1)

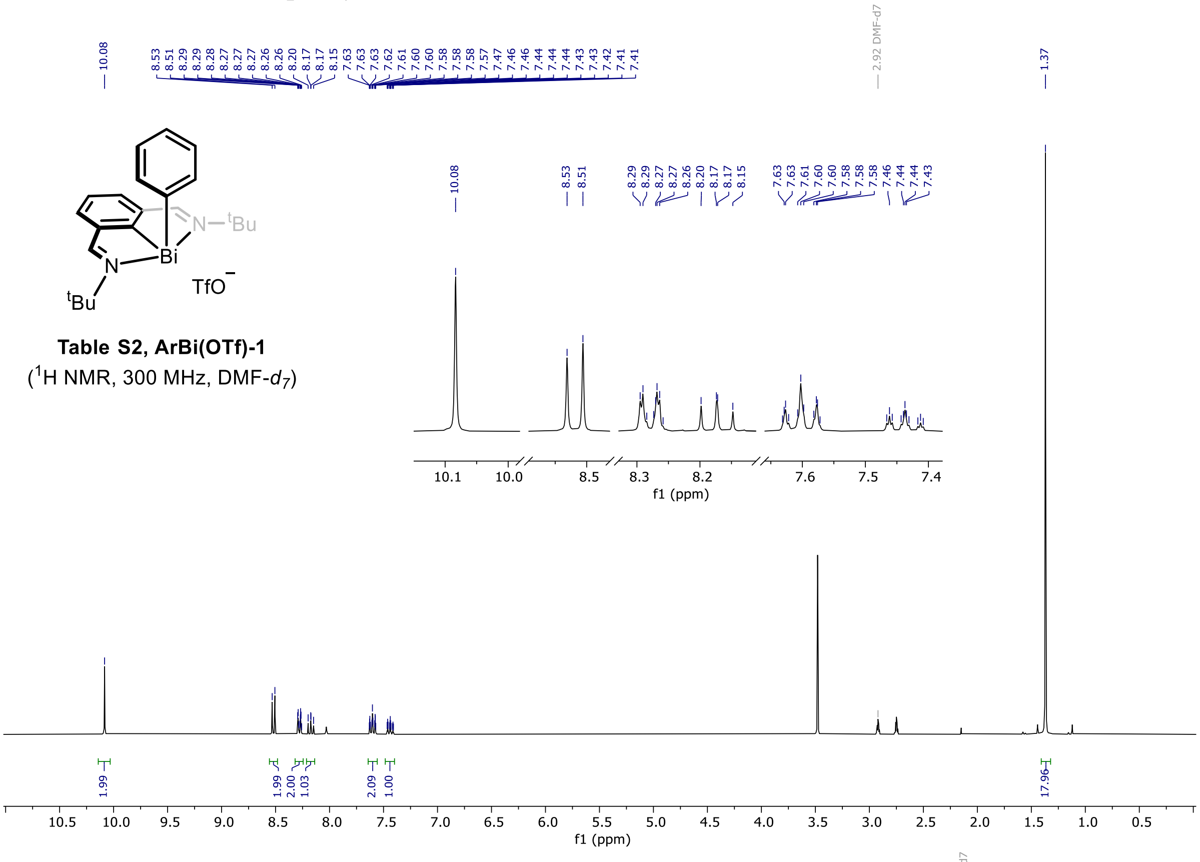

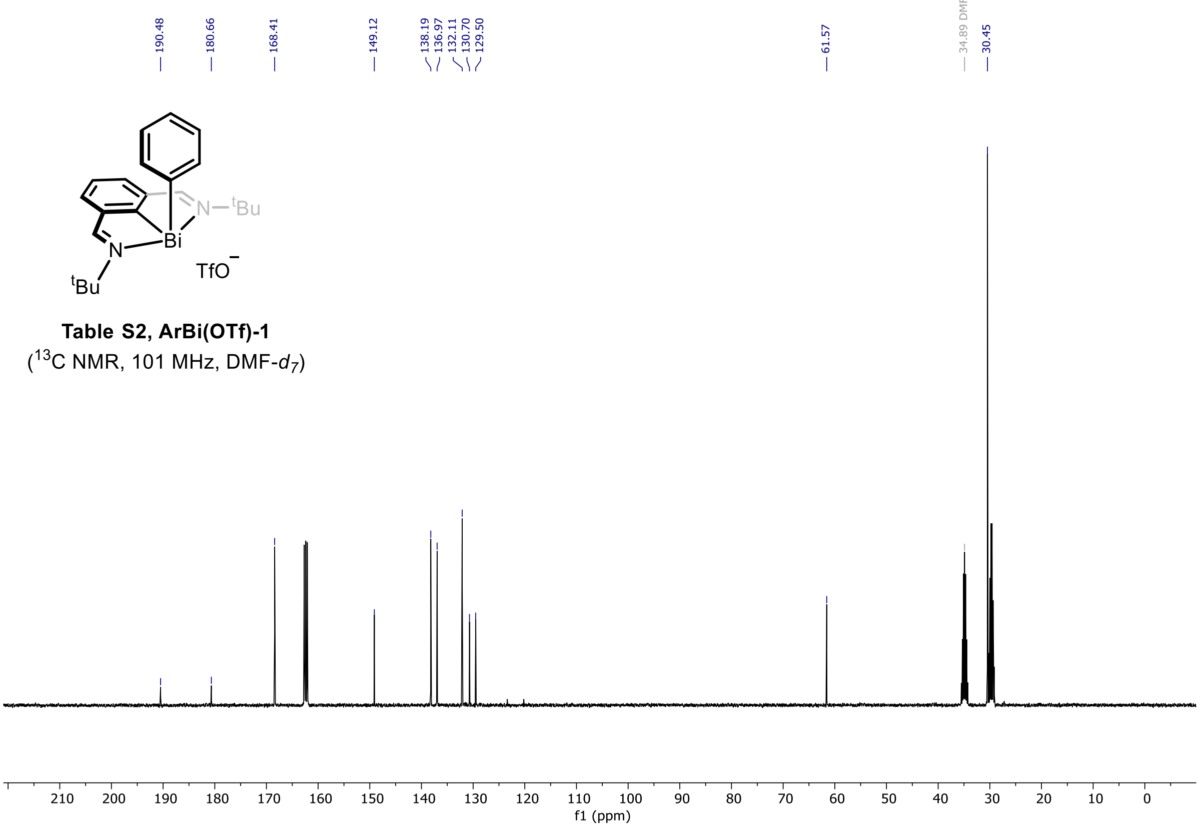

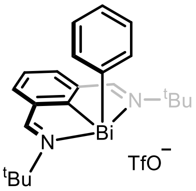

Table S2, ArBi(OTf)-1

(^{19}F NMR, 282 MHz, DMF-d_7)

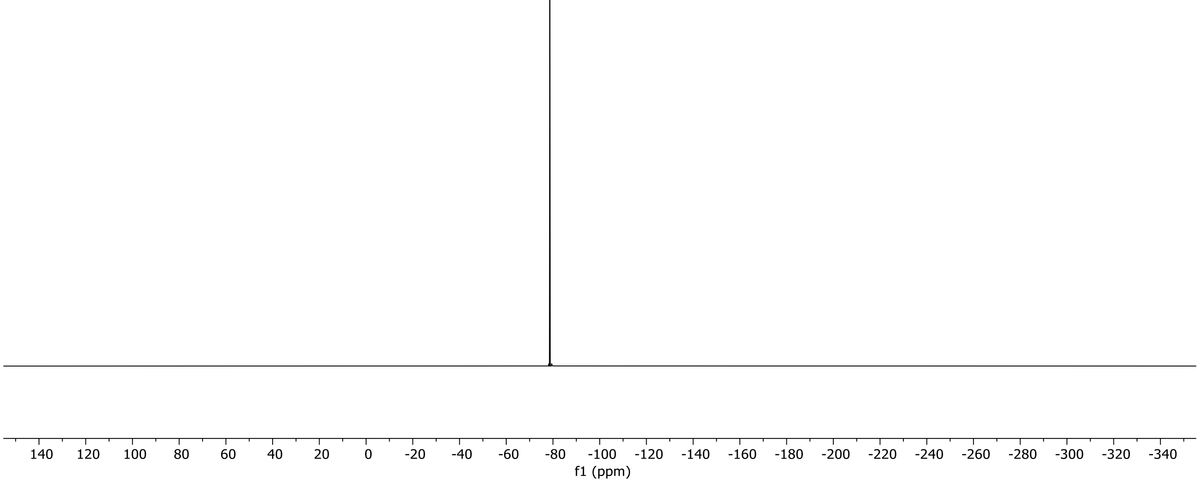

[(2,6-(^{t}BuNCH)$_2$C$_6$H$_3$)Bi(4-trifluoromethylphenyl)(OTf)] (ArBi(OTf)-2) (with 0.12 equiv. of unreacted ArTT salt)

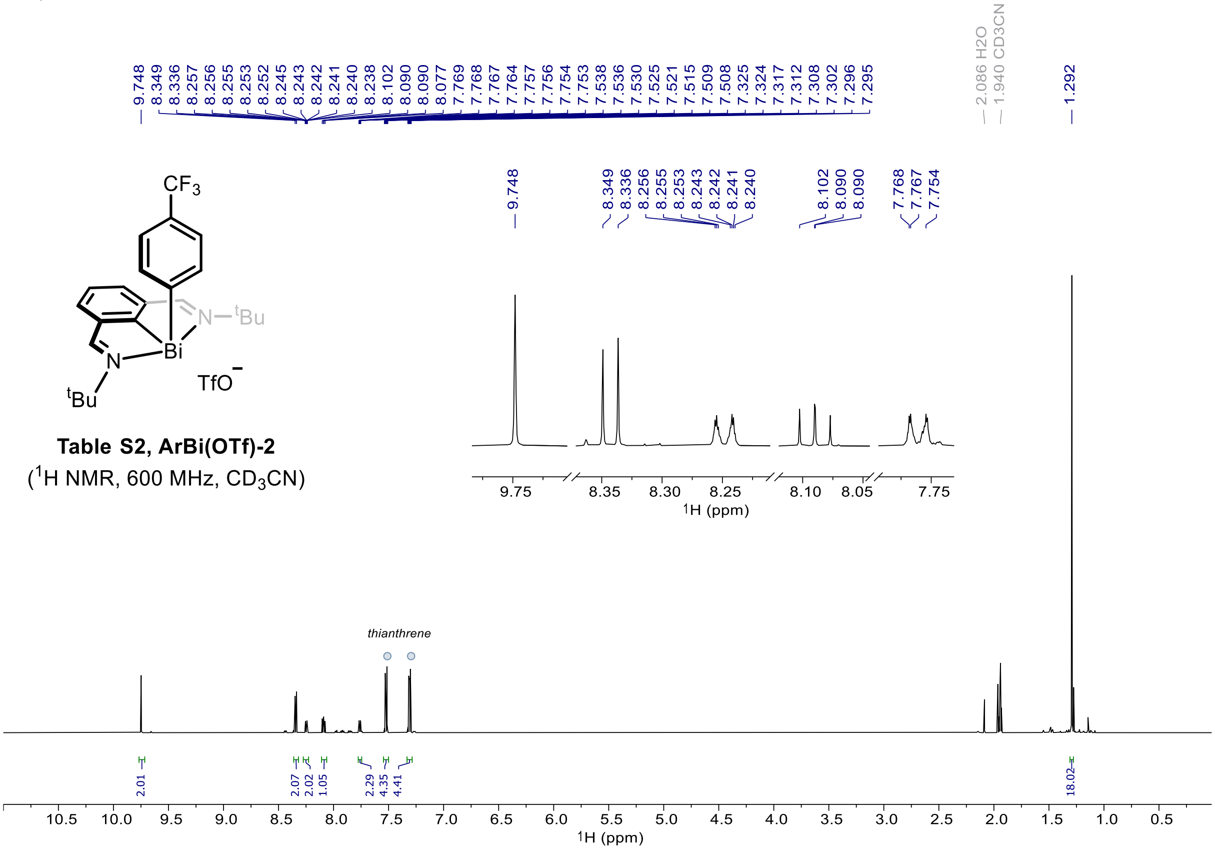

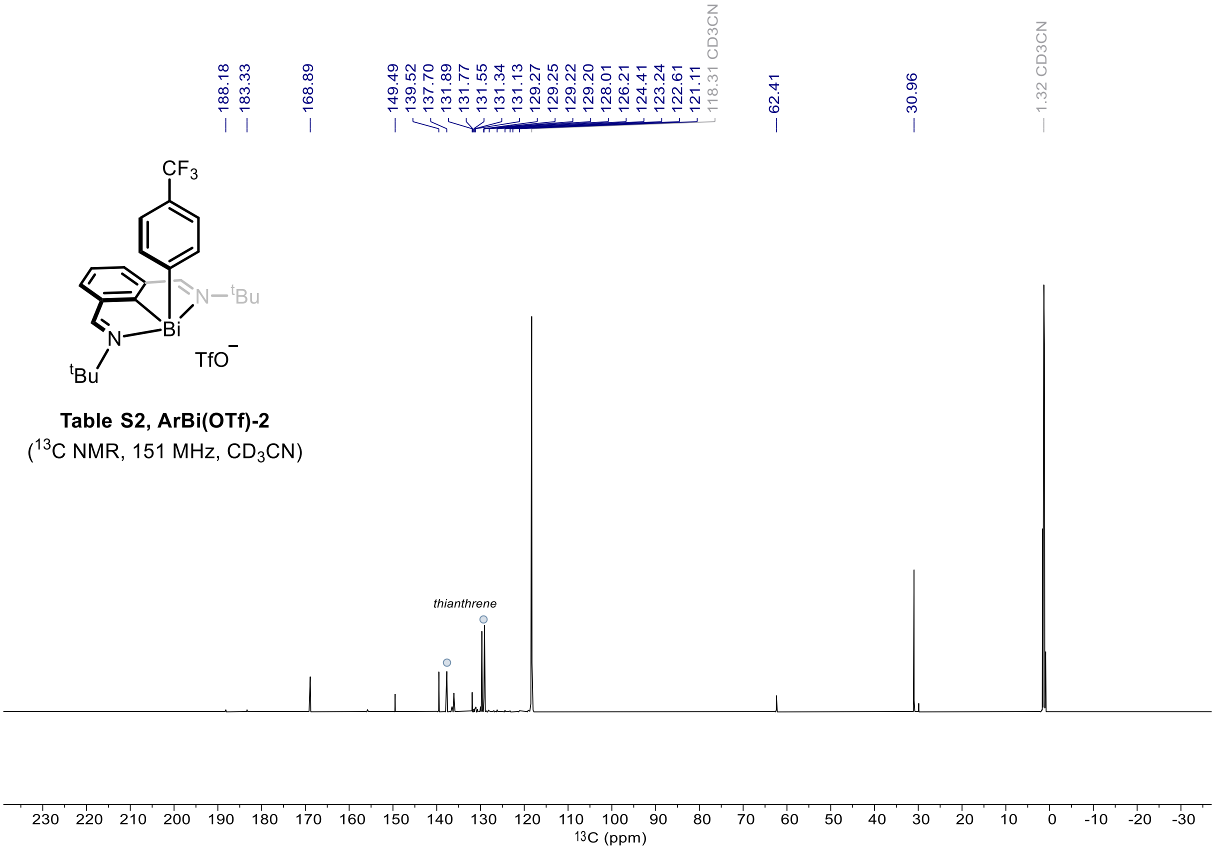

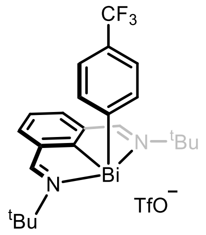

Table S2, ArBi(OTf)-2

(^{19}F NMR, 565 MHz, CD_3CN)

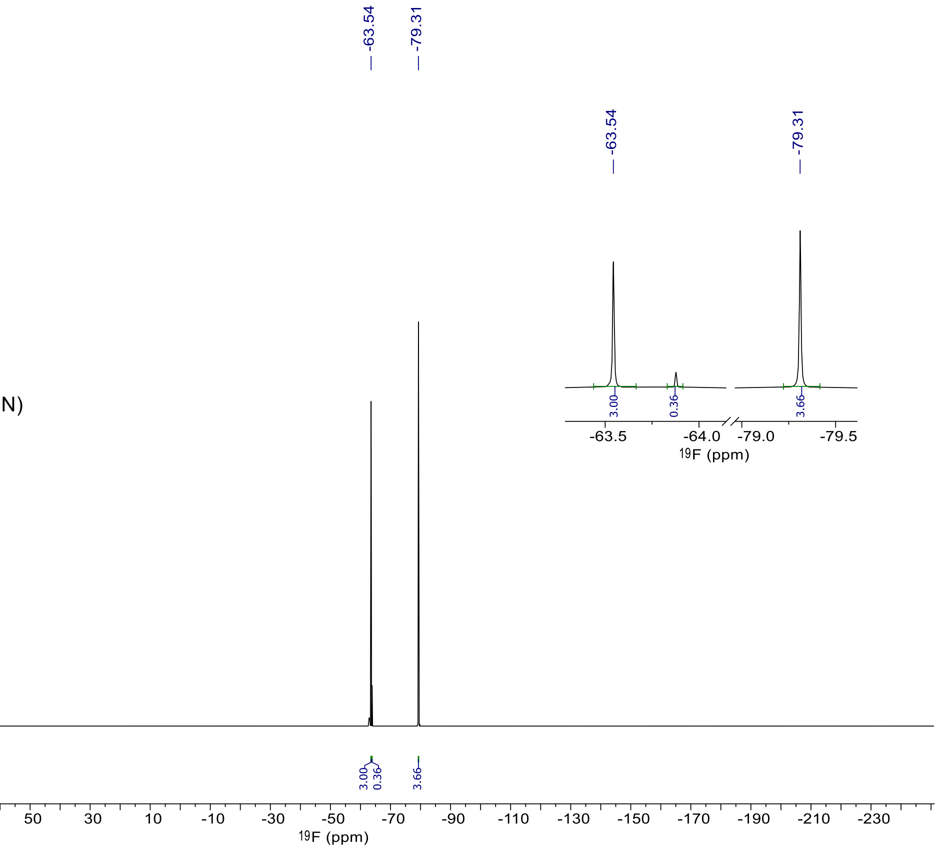

[(2,6-(^{t}BuNCH)$_2$C$_6$H$_3$)Bi(4-*tert*-butylphenyl)(OTf)] (ArBi(OTf)-3) (with 0.15 equiv. of unreacted ArTT salt)

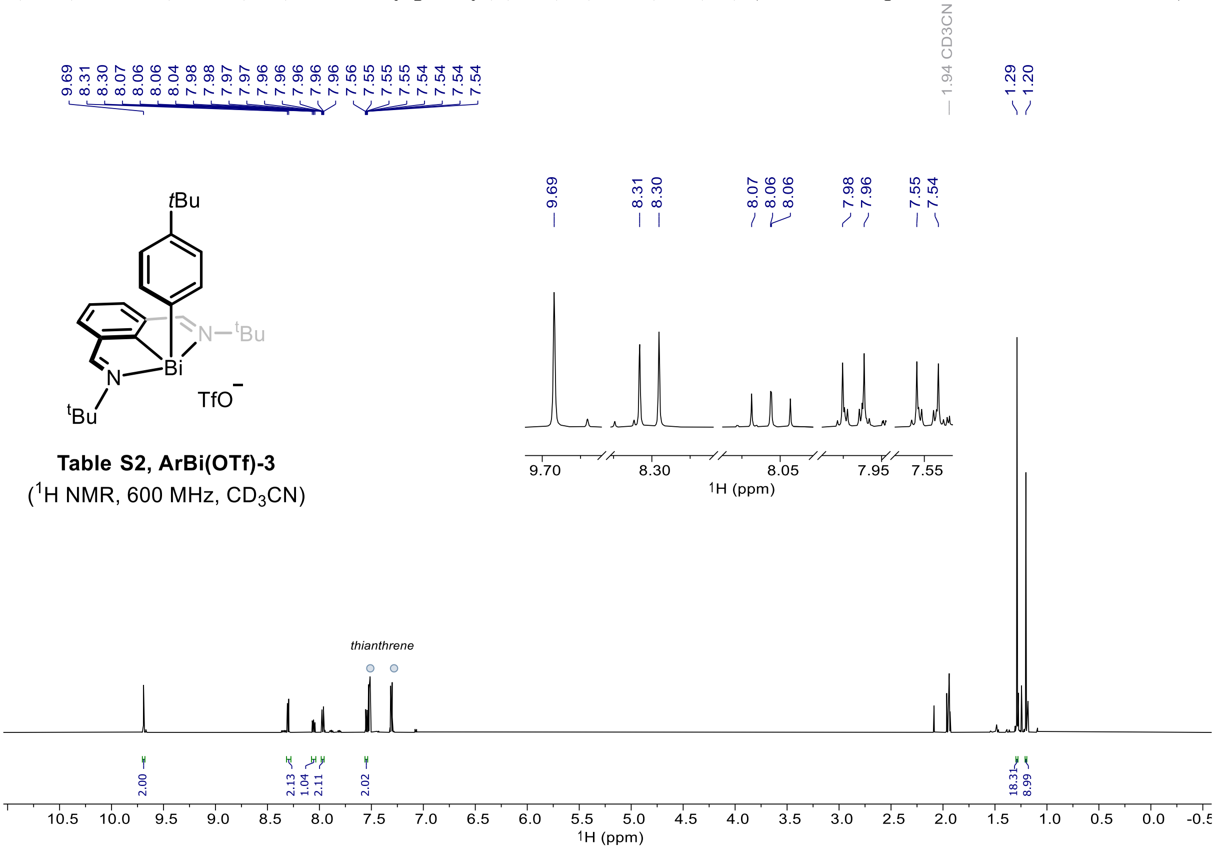

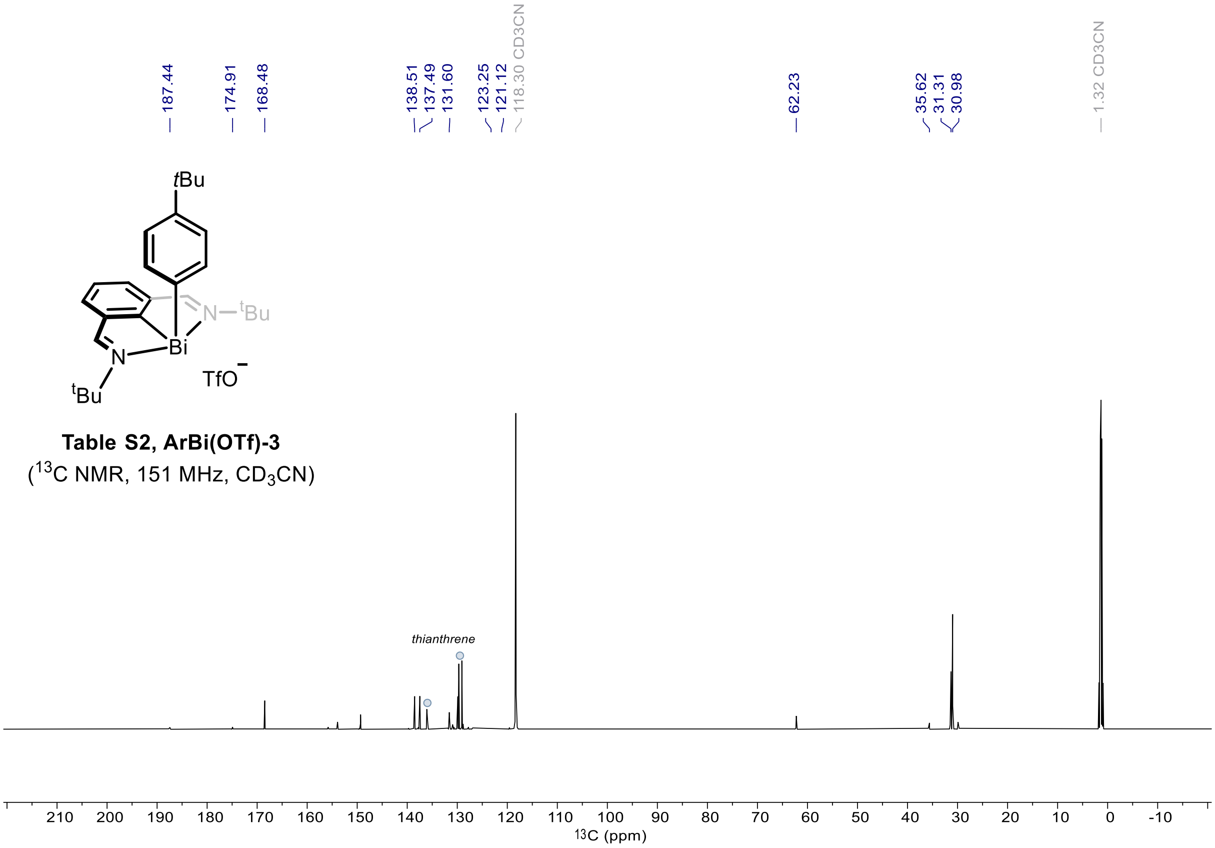

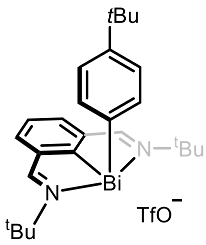

Table S2, ArBi(OTf)-3
(^{19}F NMR, 565 MHz, CD_3CN)

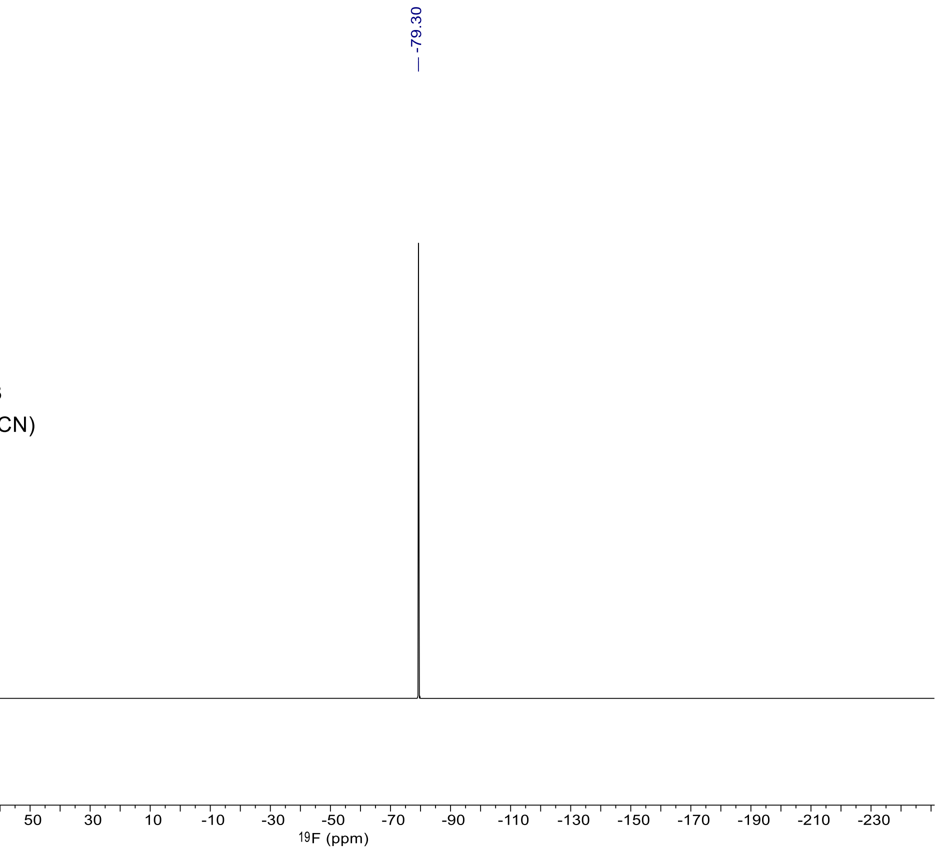

[(2,6-(^{t}BuNCH)$_2$C$_6$H$_3$)Bi(3,5-dibromophenyl)(OTf)] (ArBi(OTf)-4) (with 0.20 equiv. of unreacted ArTT salt)

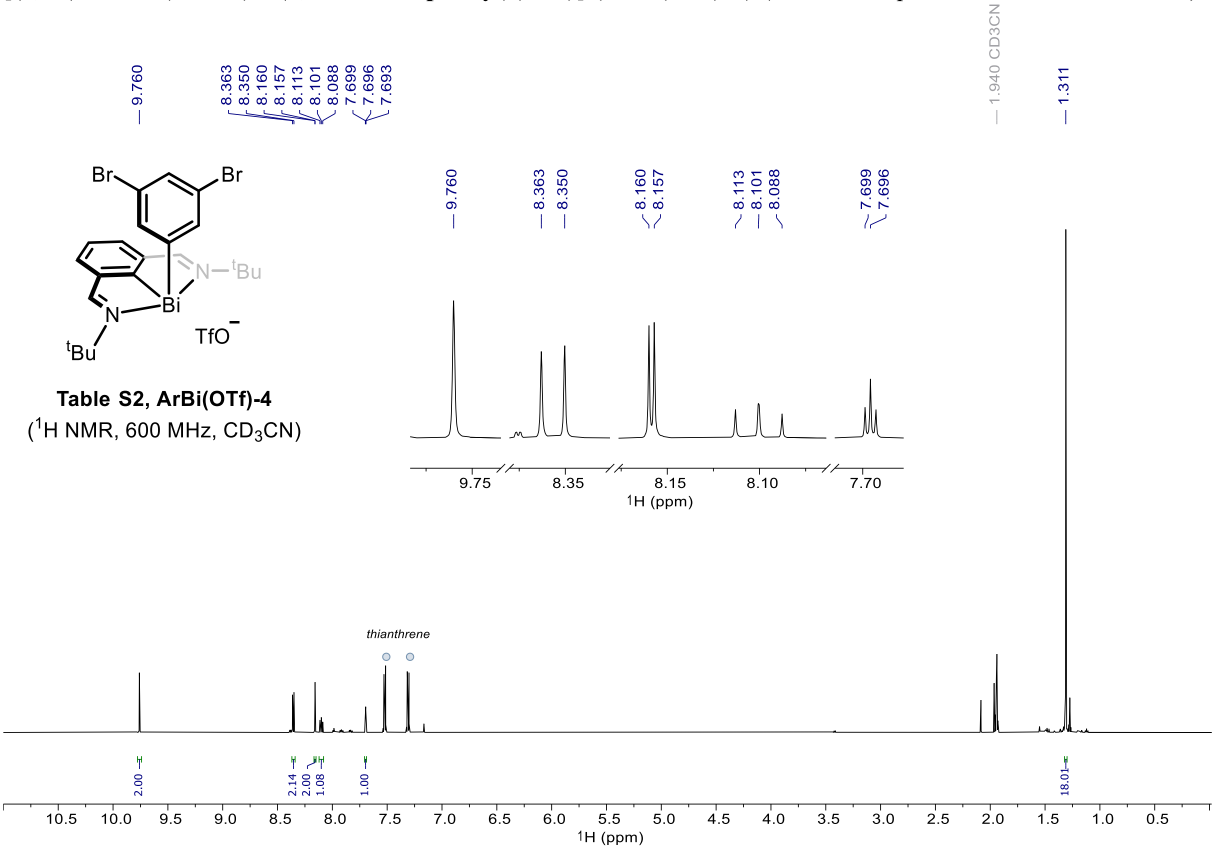

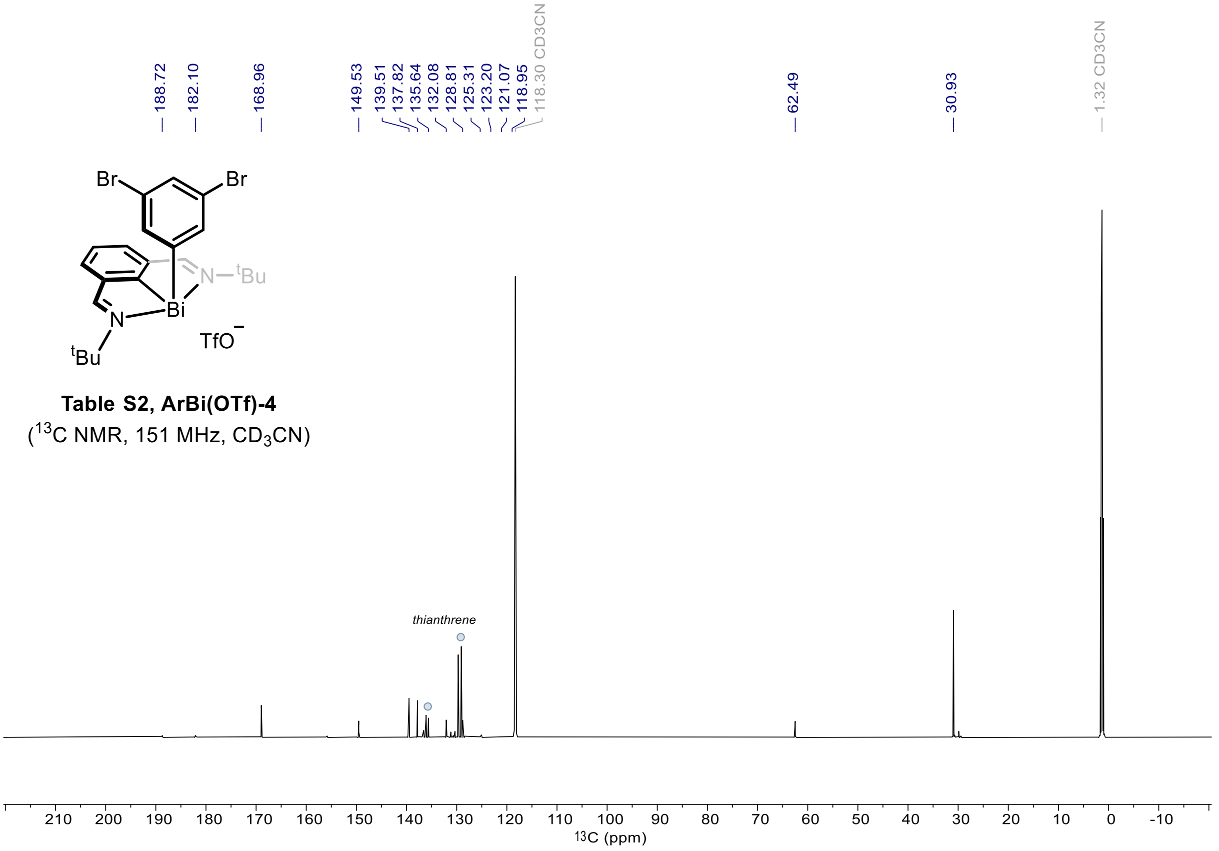

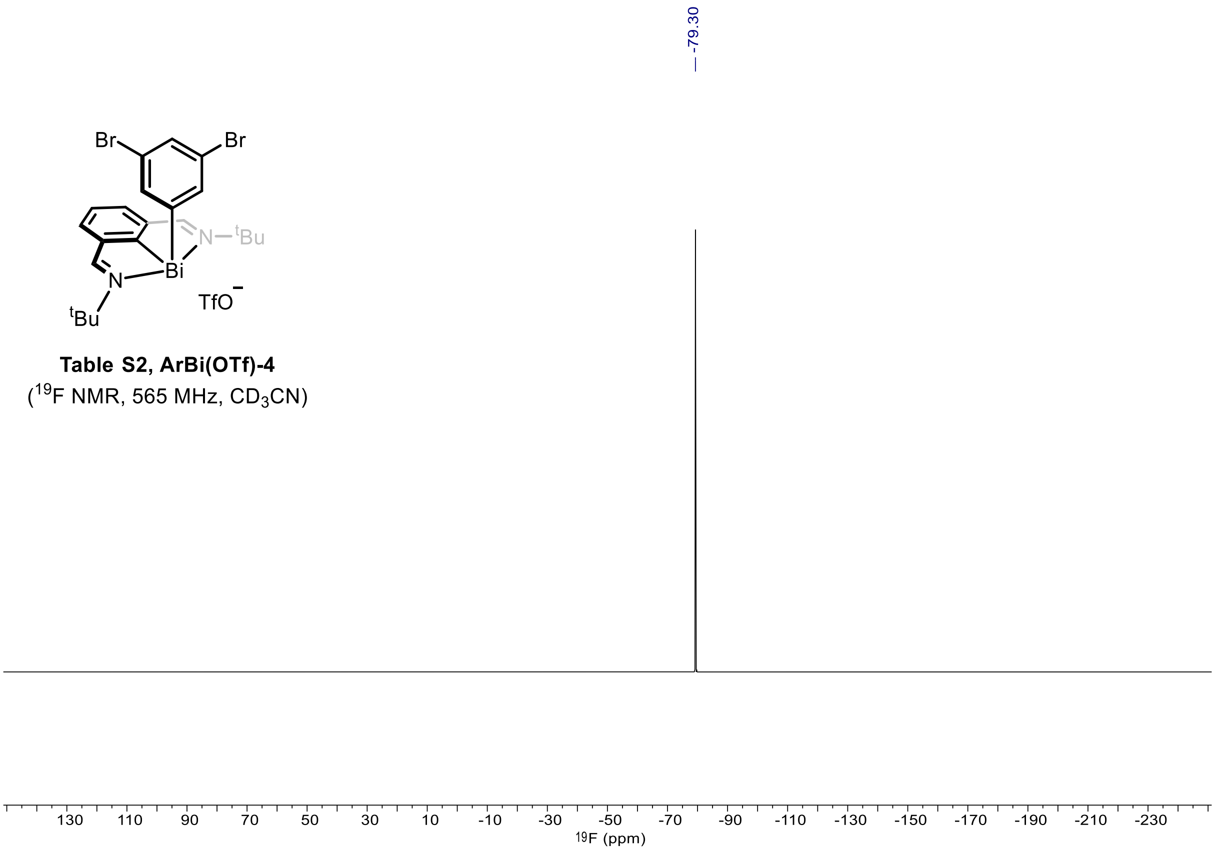

Table S2, ArBi(OTf)-4

(^{19}F NMR, 565 MHz, CD_3CN)

[(2,6-(^{t}BuNCH)$_2$C$_6$H$_3$)Bi(2-benzo[b]thiophenyl)(OTf)] (ArBi(OTf)-5) (with unknown side products)

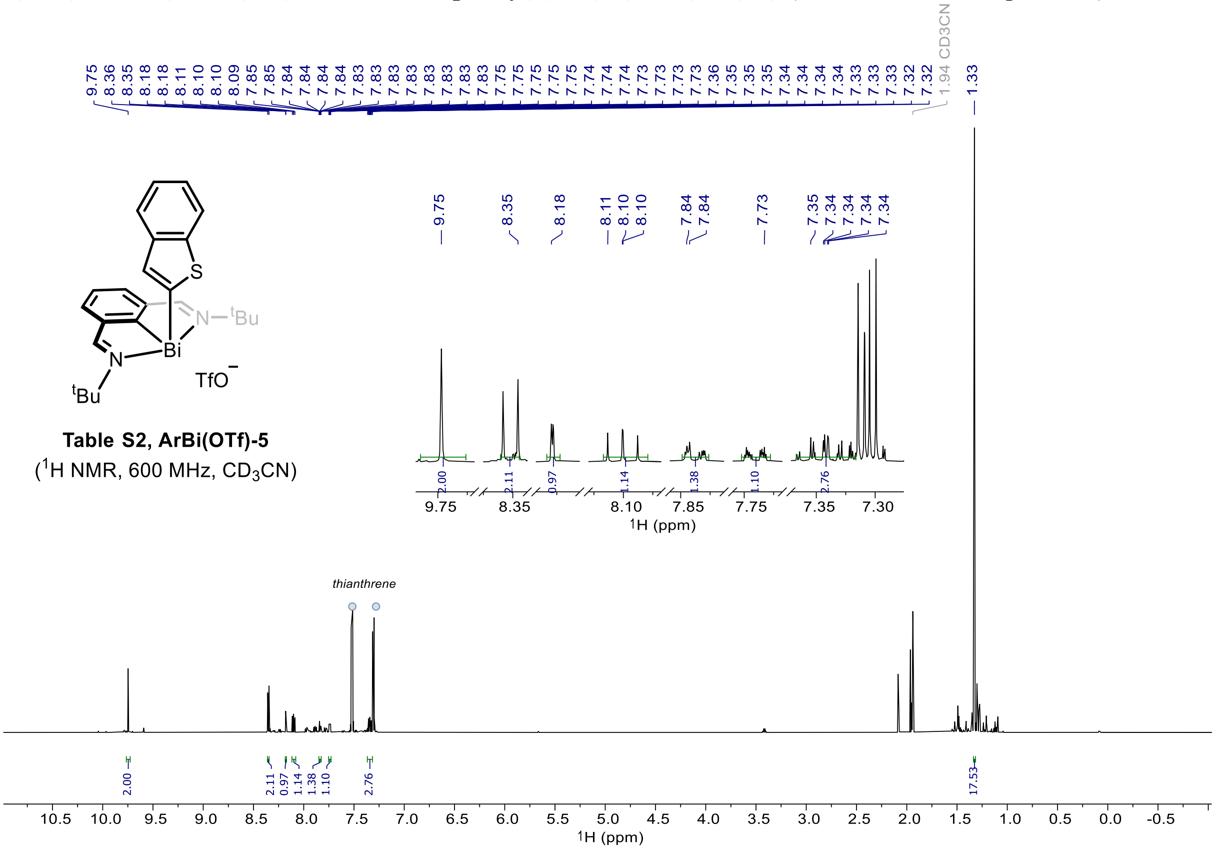

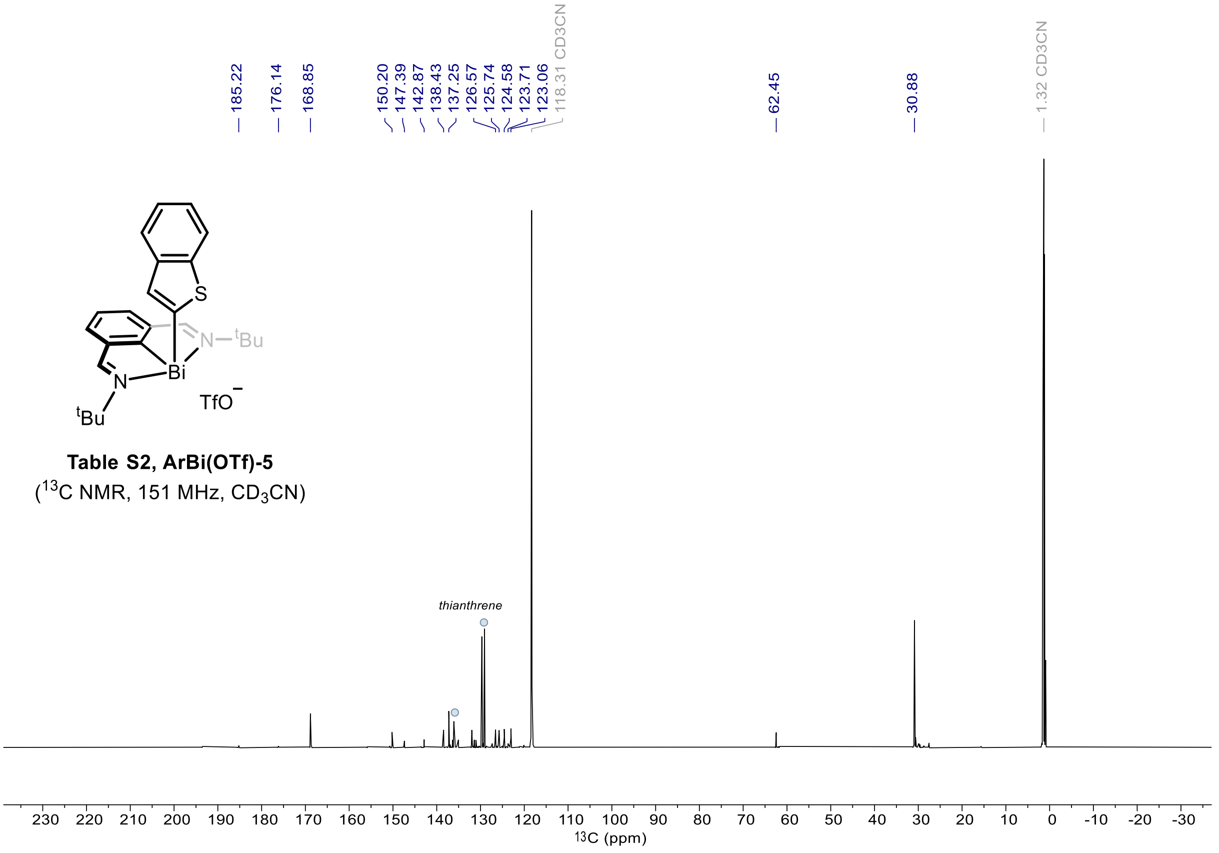

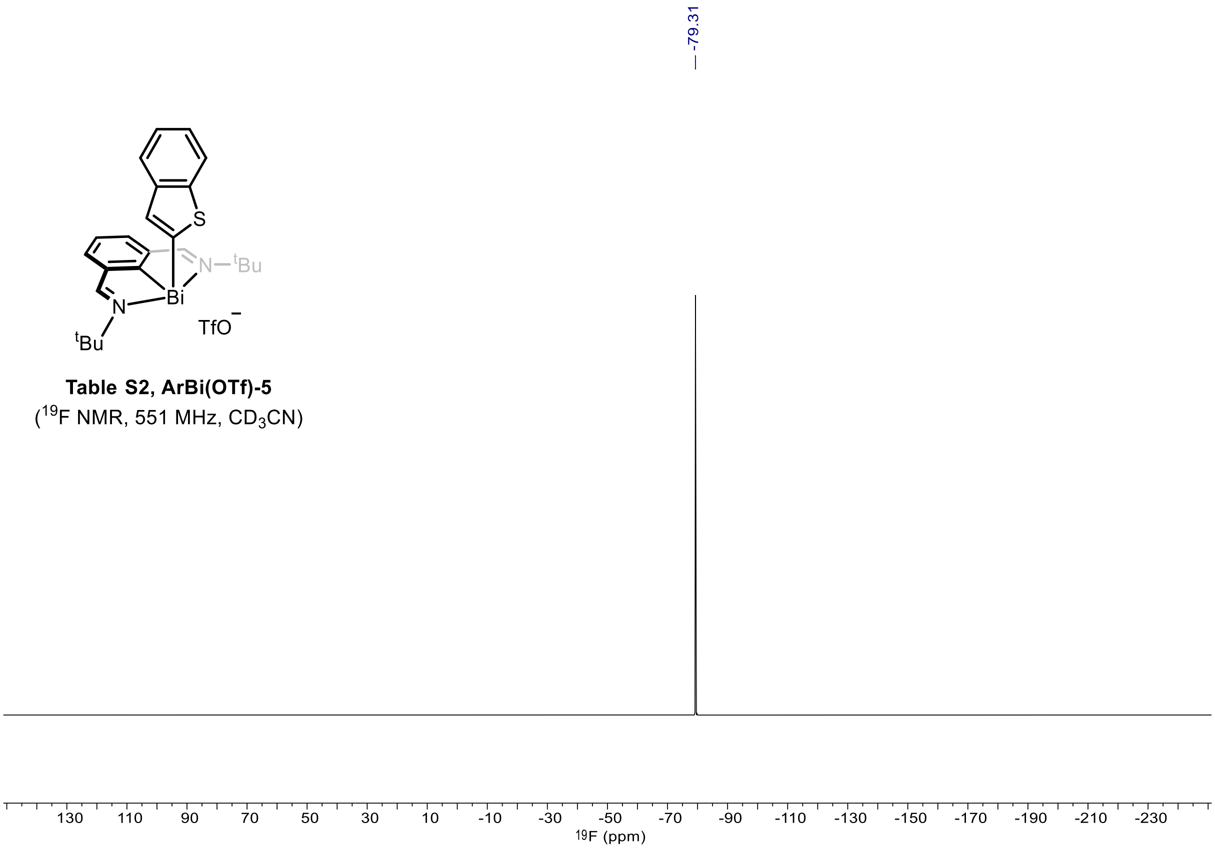

Table S2, ArBi(OTf)-5
(^{19}F NMR, 551 MHz, CD_3CN)

[(2,6-(^{t}BuNCH)$_2$C$_6$H$_3$)Bi(6-quinolinyl)(OTf)] (ArBi(OTf)-6)

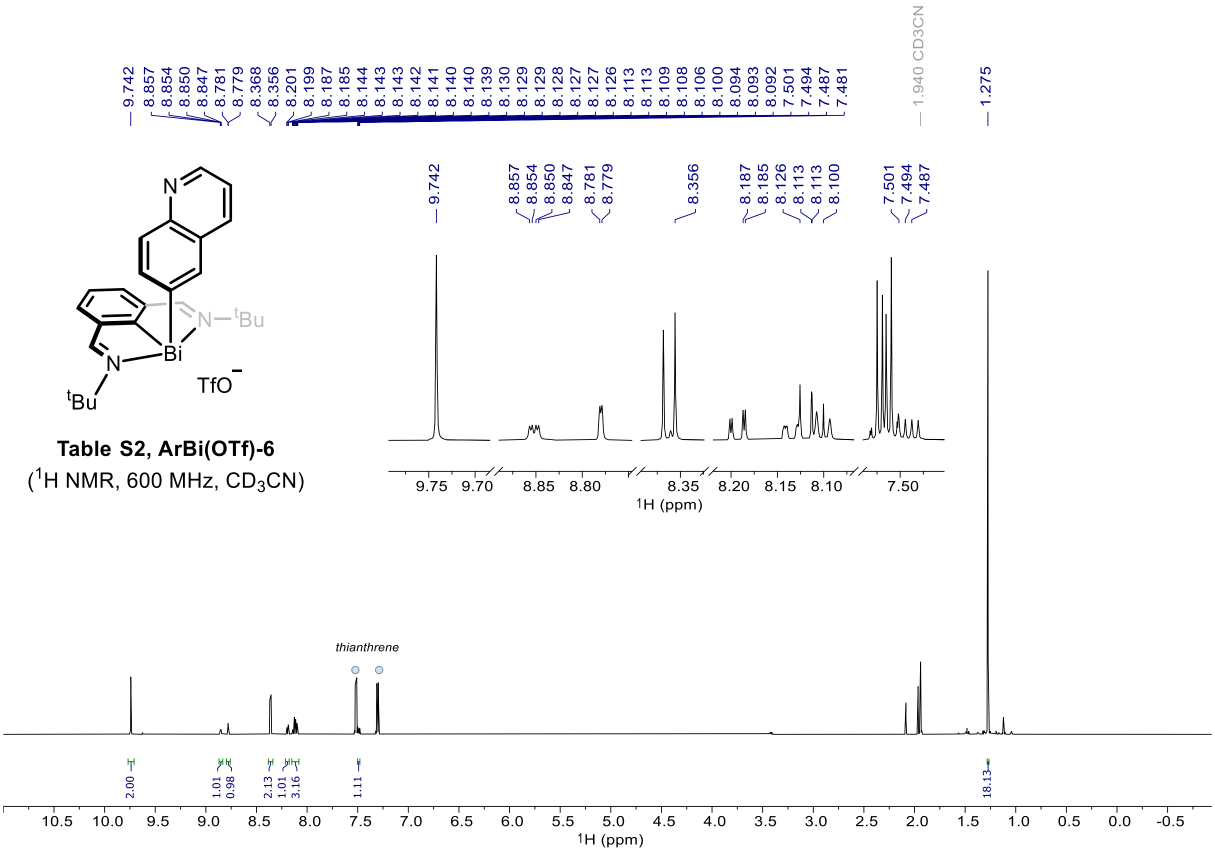

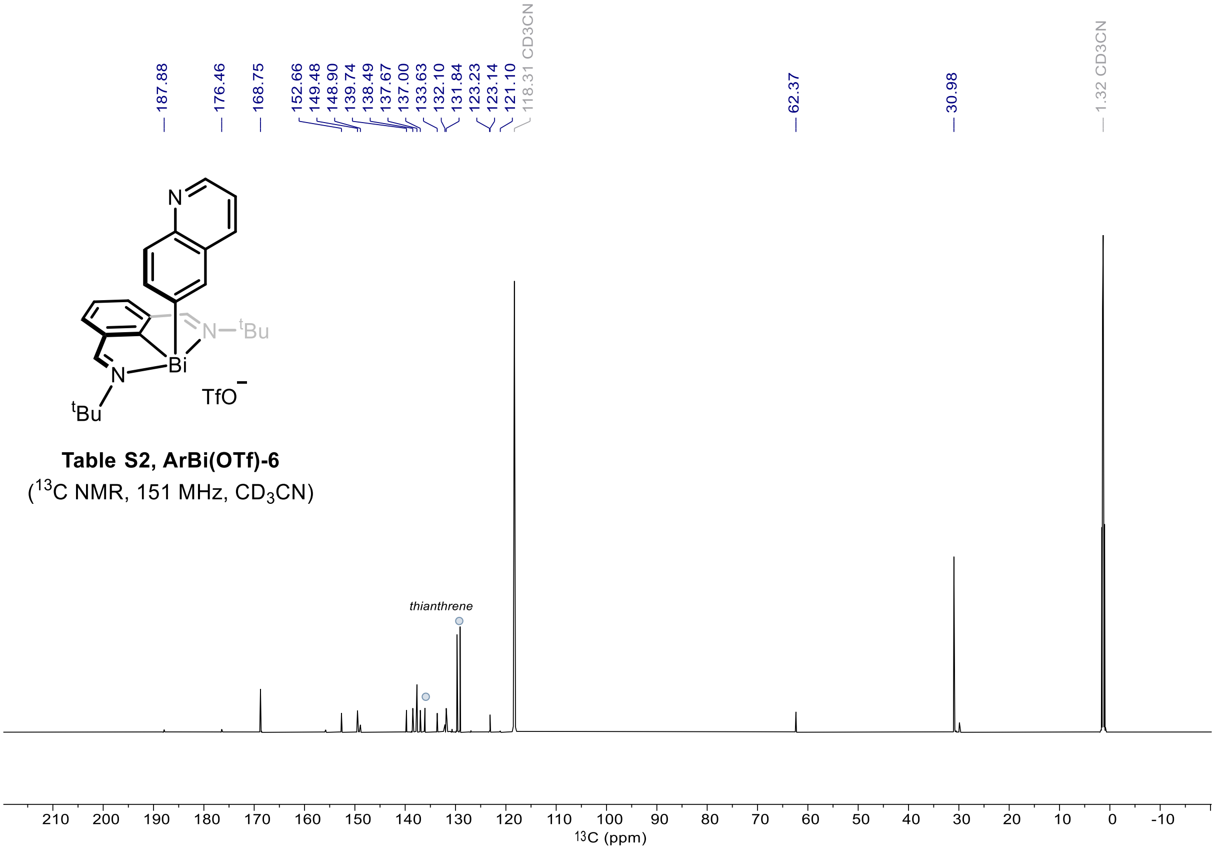

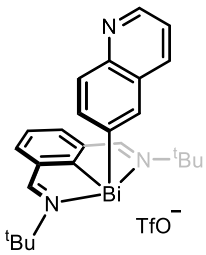

Table S2, ArBi(OTf)-6
(^{19}F NMR, 565 MHz, CD_3CN)

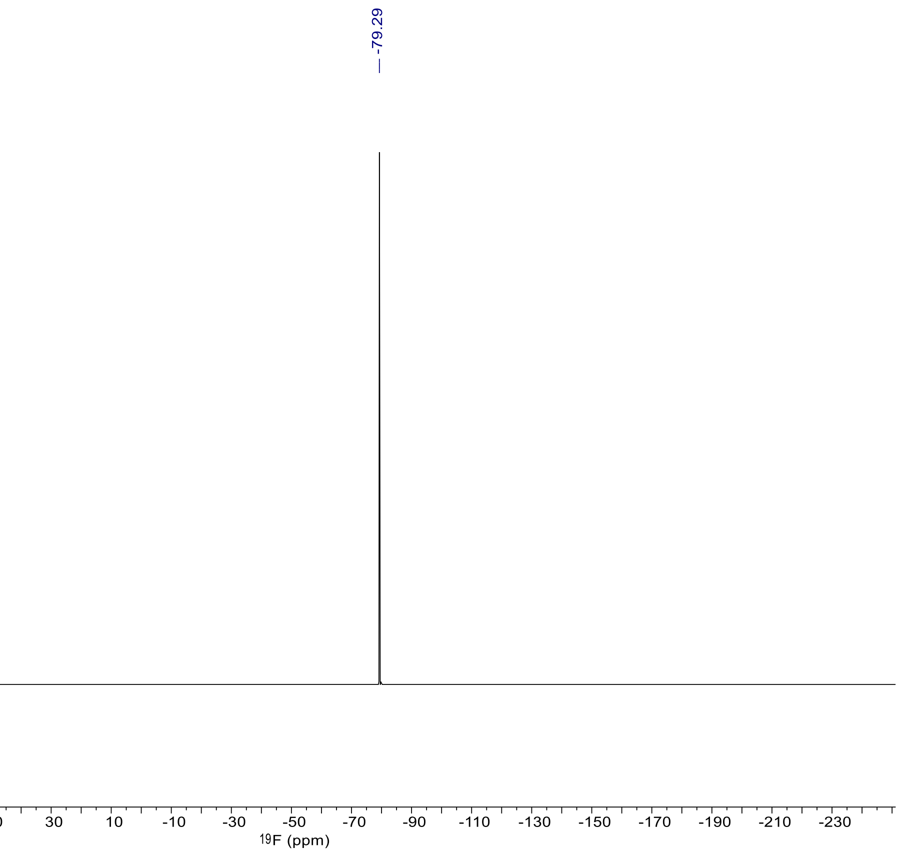

[(2,6-(^{t}BuNCH)$_2$C$_6$H$_3$)Bi(8-butoxyquinolin-5-yl)(BF$_4$)] (ArBi(BF$_4$)-2) (with 25% of unknown side product)

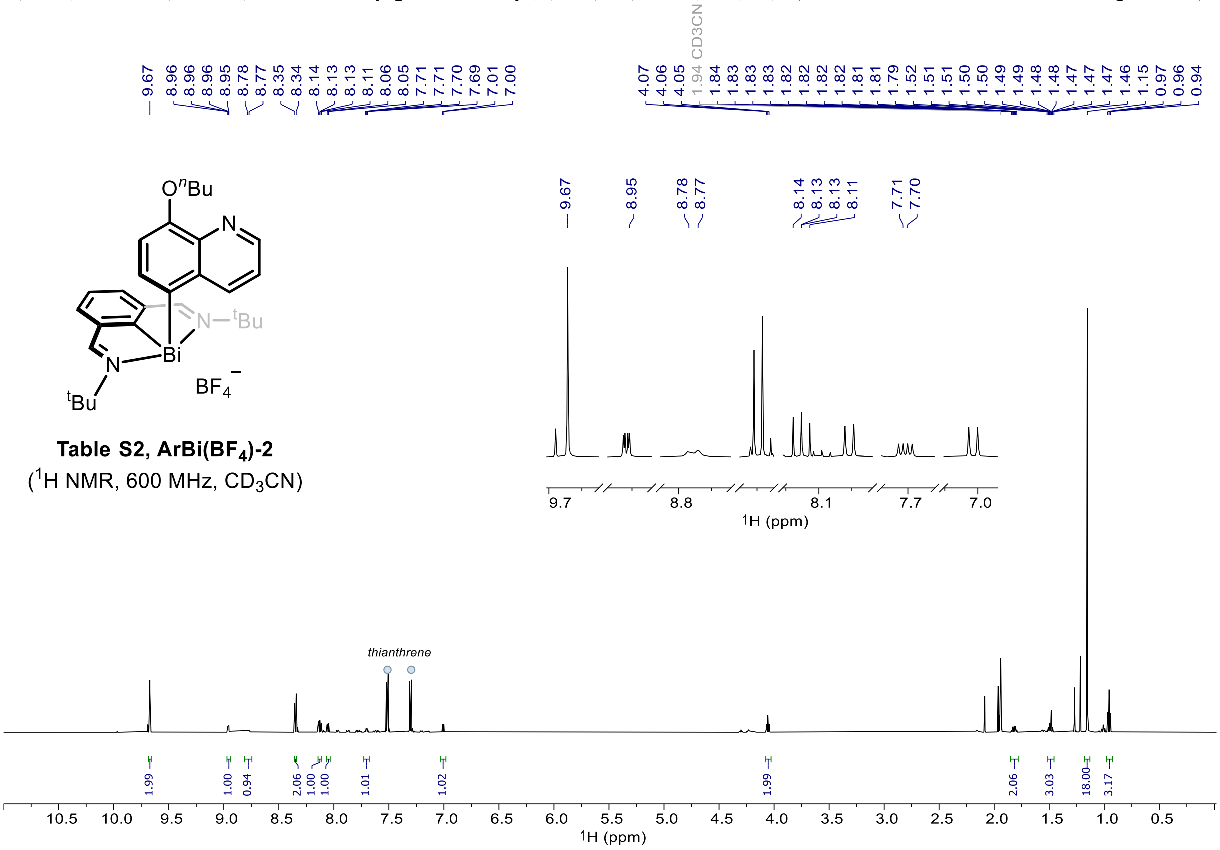

Table S2, ArBi(BF$_4$)-2
(^{1}H NMR, 600 MHz, CD$_3$CN)

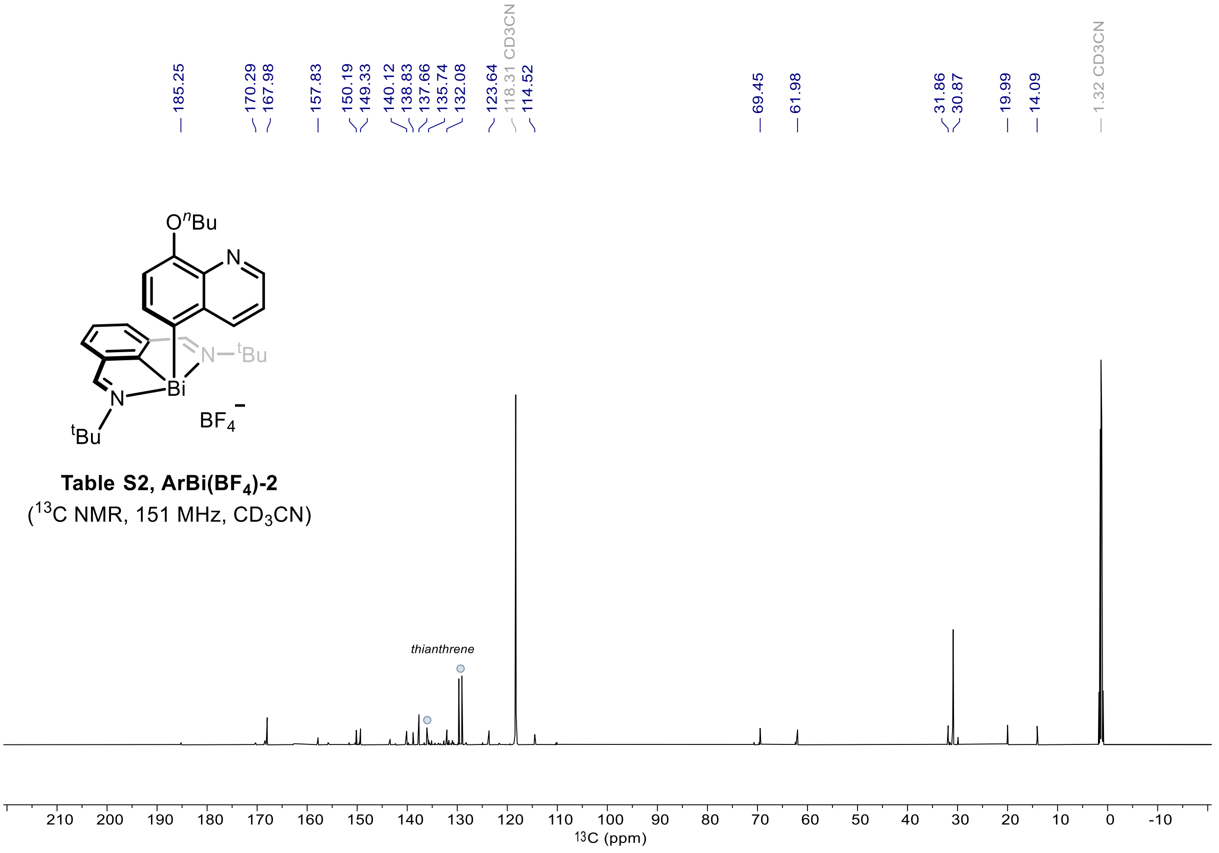

Table S2, ArBi(BF$_4$)-2
(^{13}C NMR, 151 MHz, CD$_3$CN)

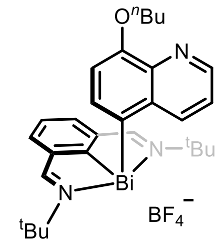

Table S2, ArBi(BF$_4$)-2
(^{19}F NMR, 565 MHz, CD_3CN)

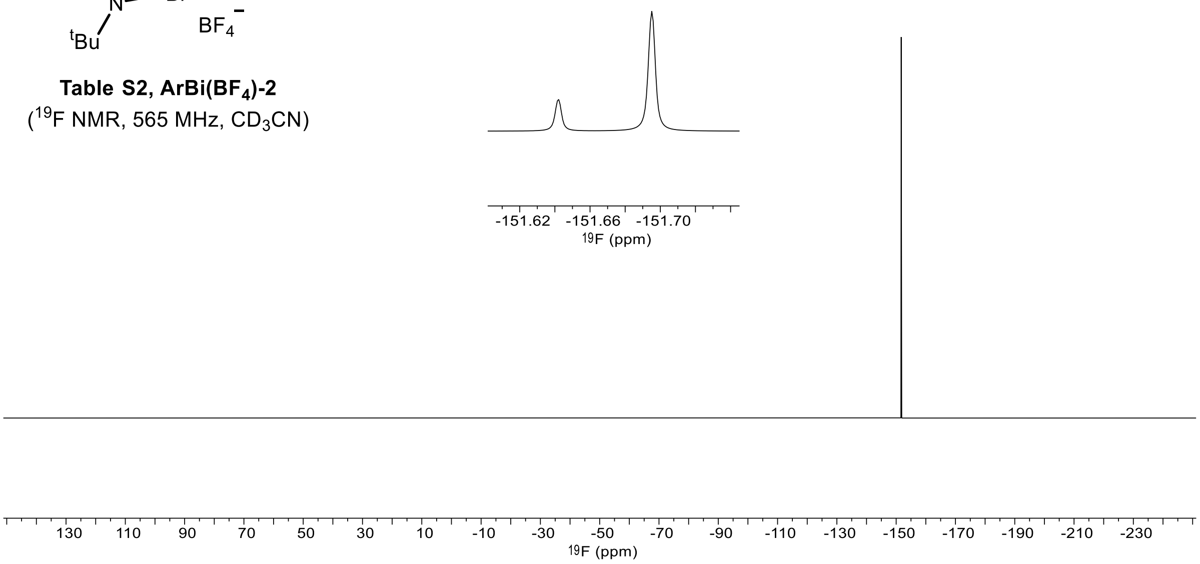

[(2,6-(^{t}BuNCH)$_2$C$_6$H$_3$)Bi(*atomoxetine*)(BF$_4$)] (ArBi(BF$_4$)-3)

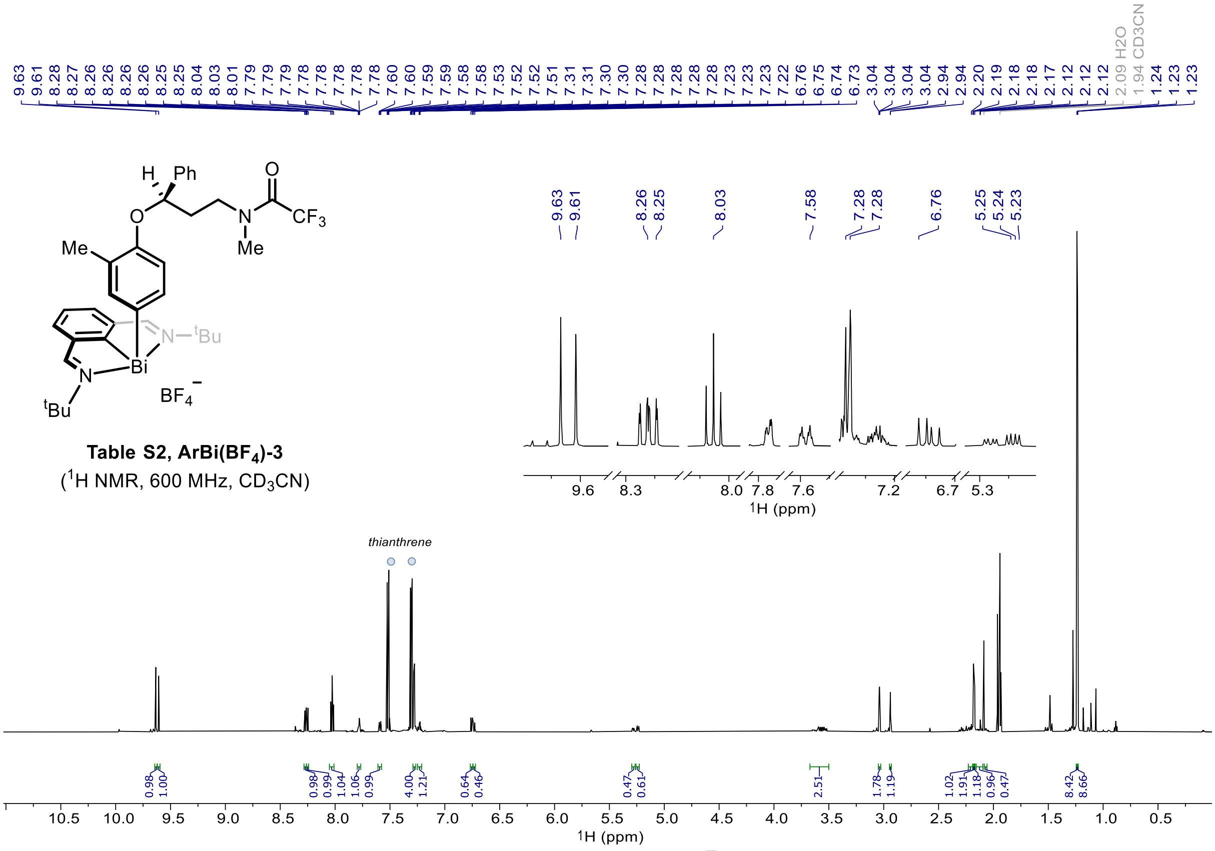

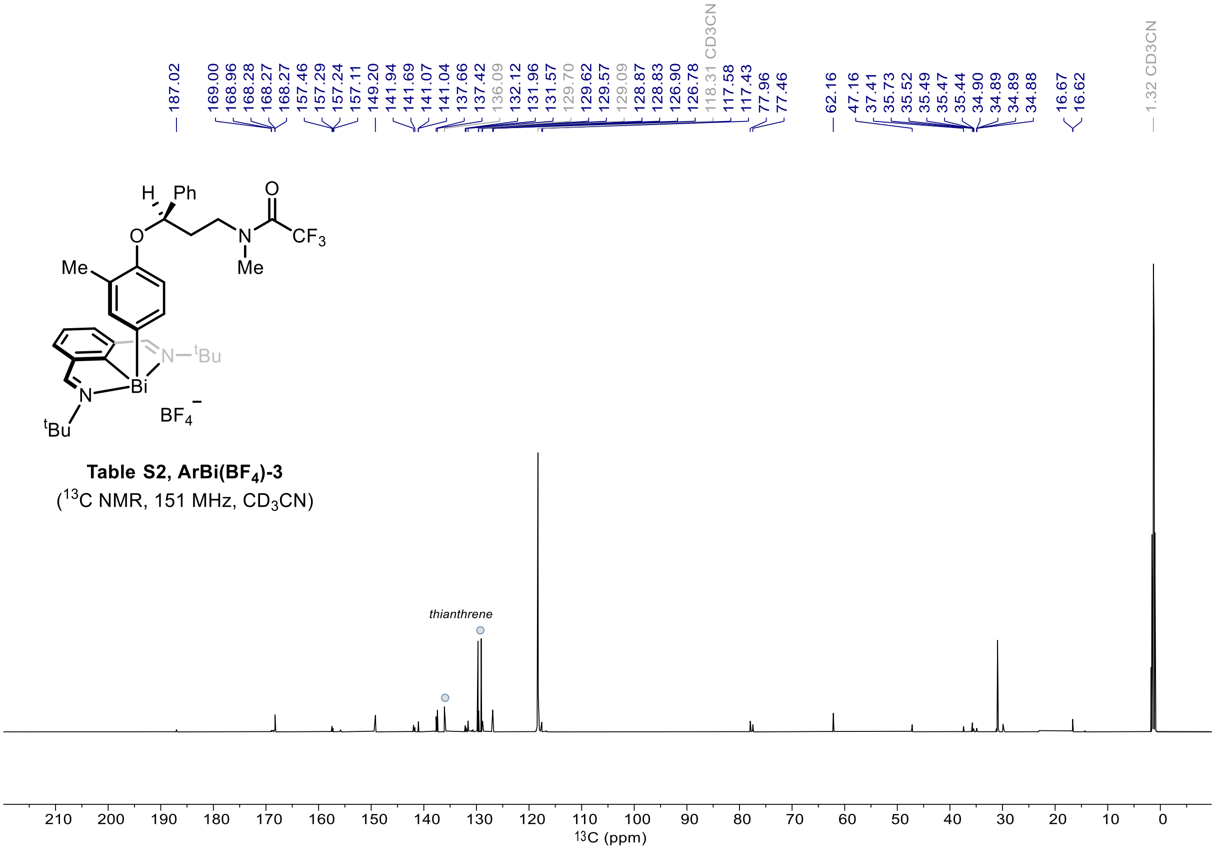

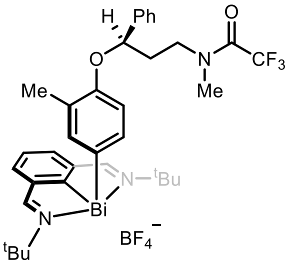

Table S2, ArBi(BF_4)-3
(^{13}F NMR, 565 MHz, CD_3CN)

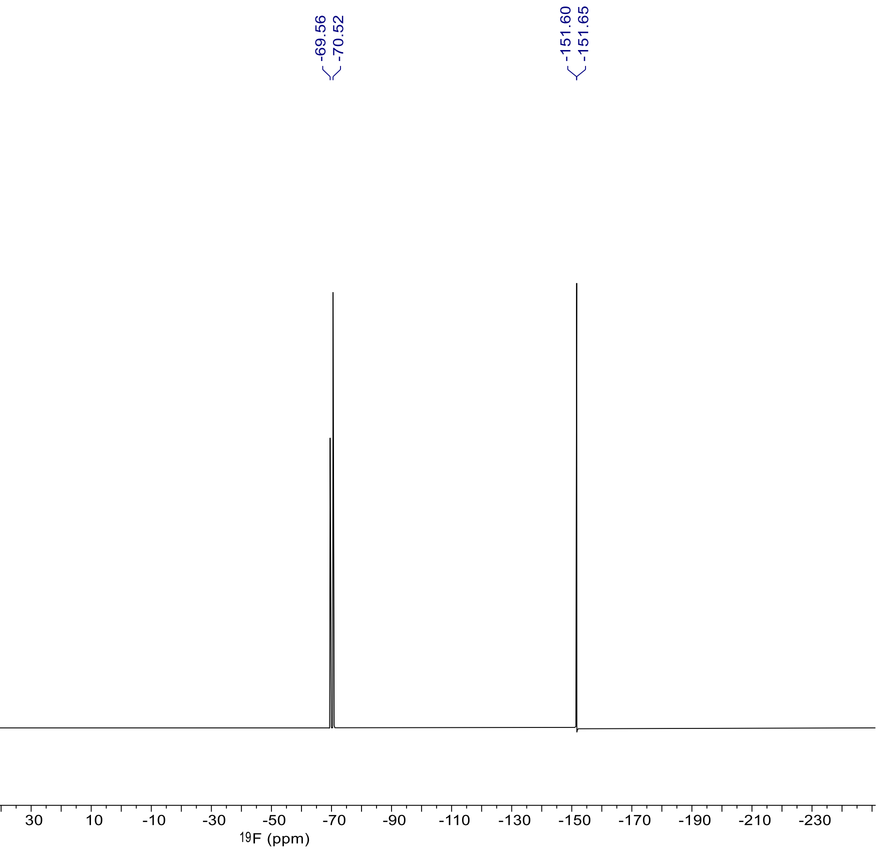

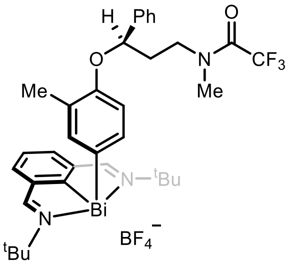

Table S2, ArBi(BF_4)-3
(^{11}B NMR, 193 MHz, CD_3CN)

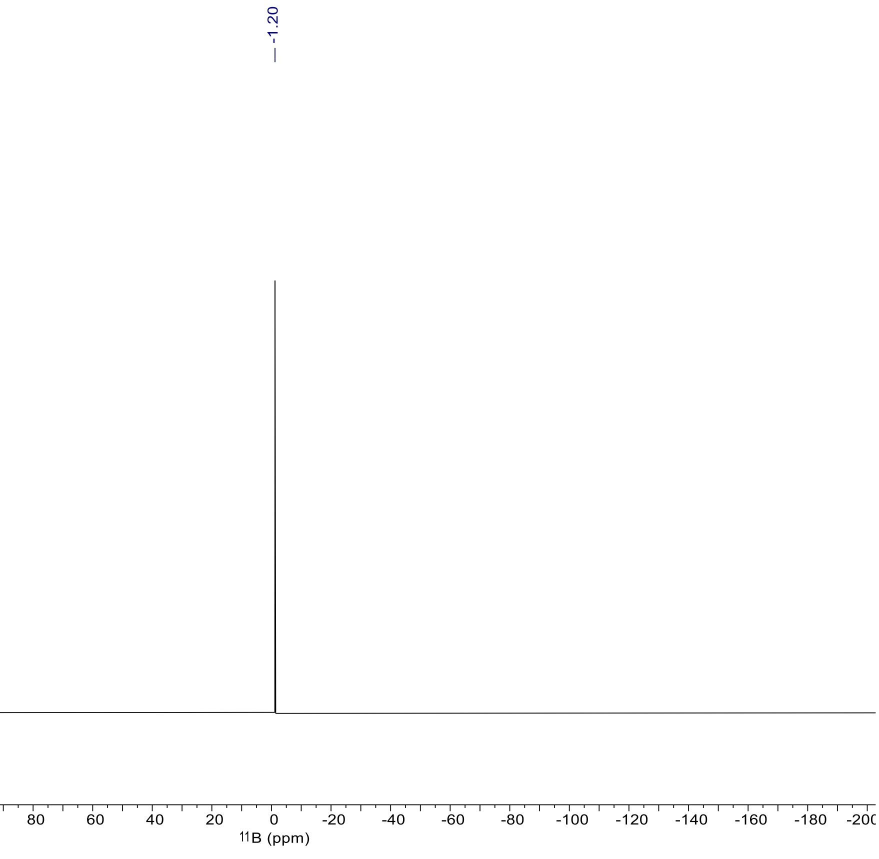

[(2,6-(^{t}BuNCH)$_2$C$_6$H$_3$)Bi(*pyriproxifen*)(BF$_4$)] (ArBi(BF$_4$)-4)

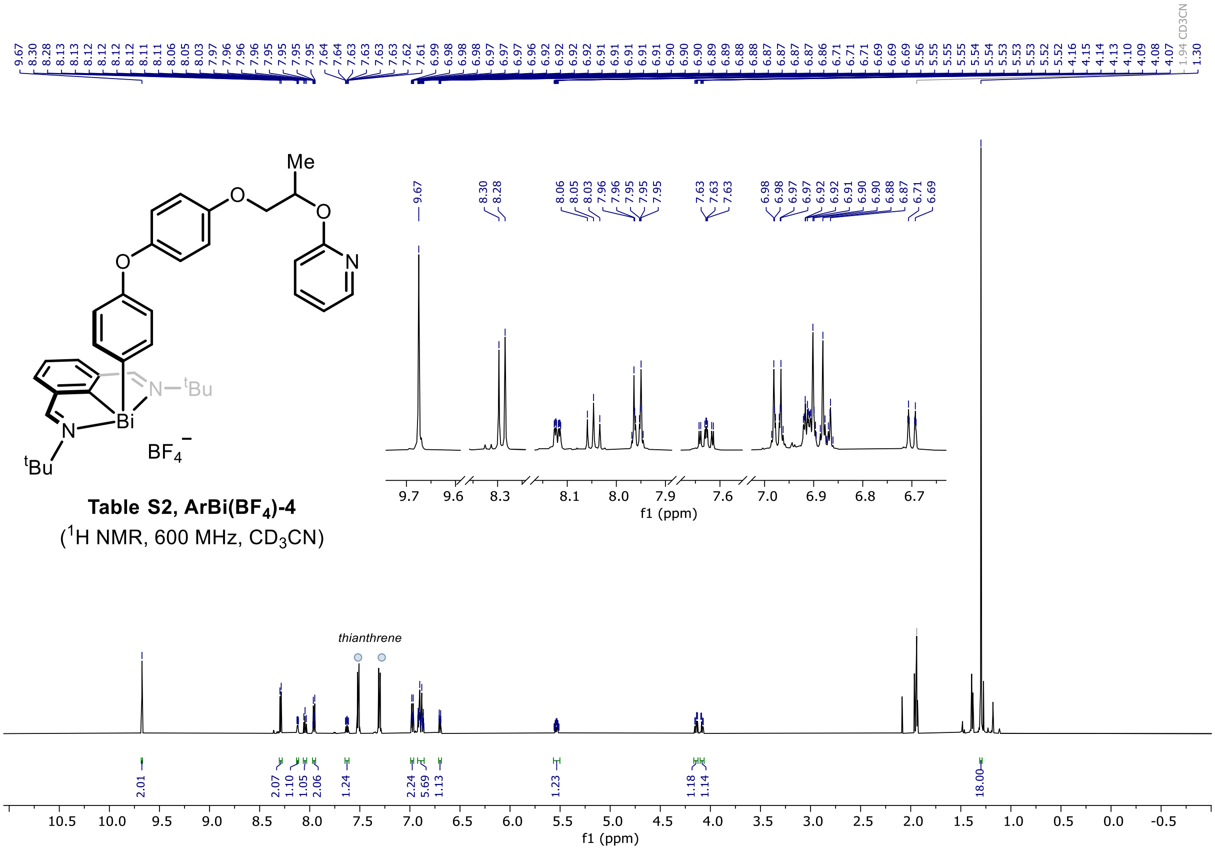

Table S2, ArBi(BF$_4$)-4 (^{1}H NMR, 600 MHz, CD_3CN)

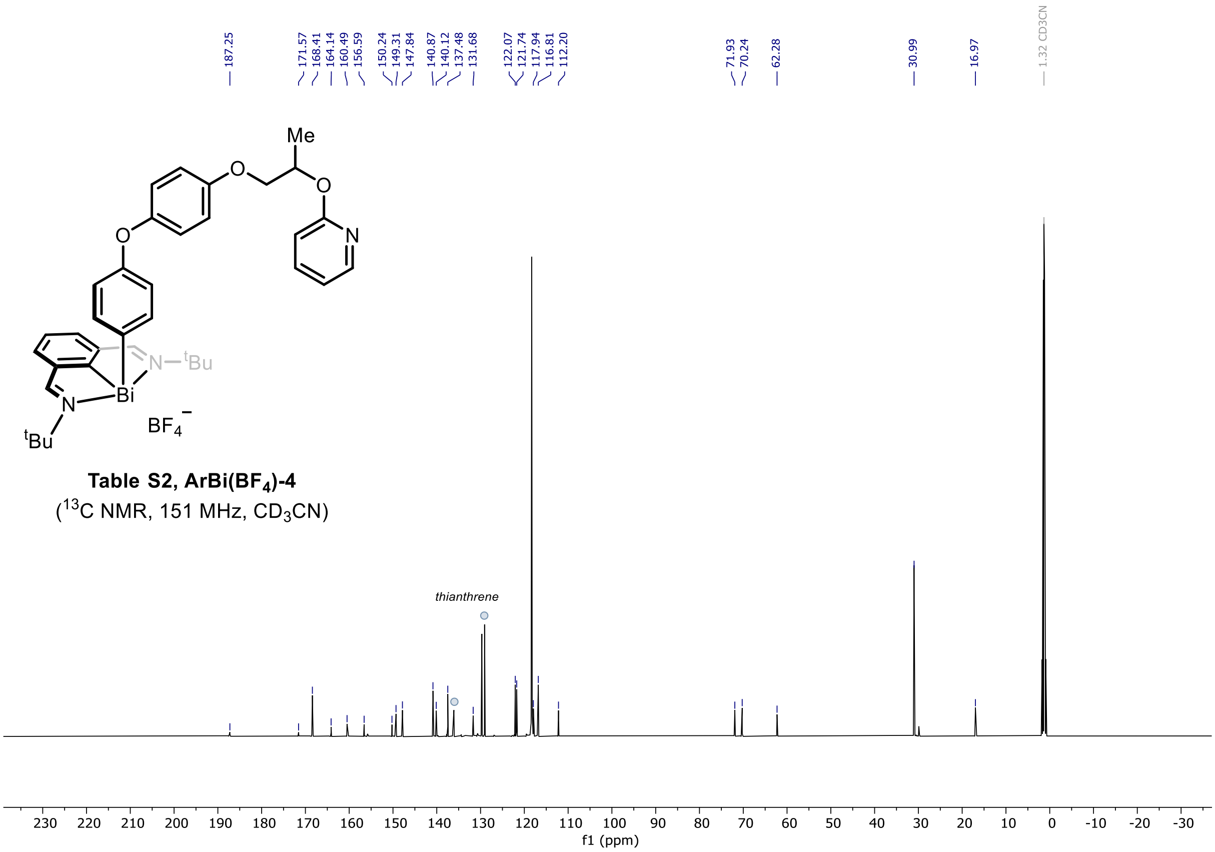

Table S2, ArBi(BF$_4$)-4 (^{13}C NMR, 151 MHz, CD_3CN)

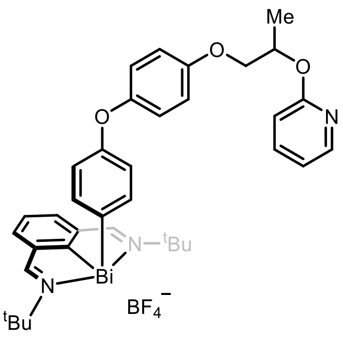

Table S2, ArBi(BF_4)-4
(^{19}F NMR, 565 MHz, CD_3CN)

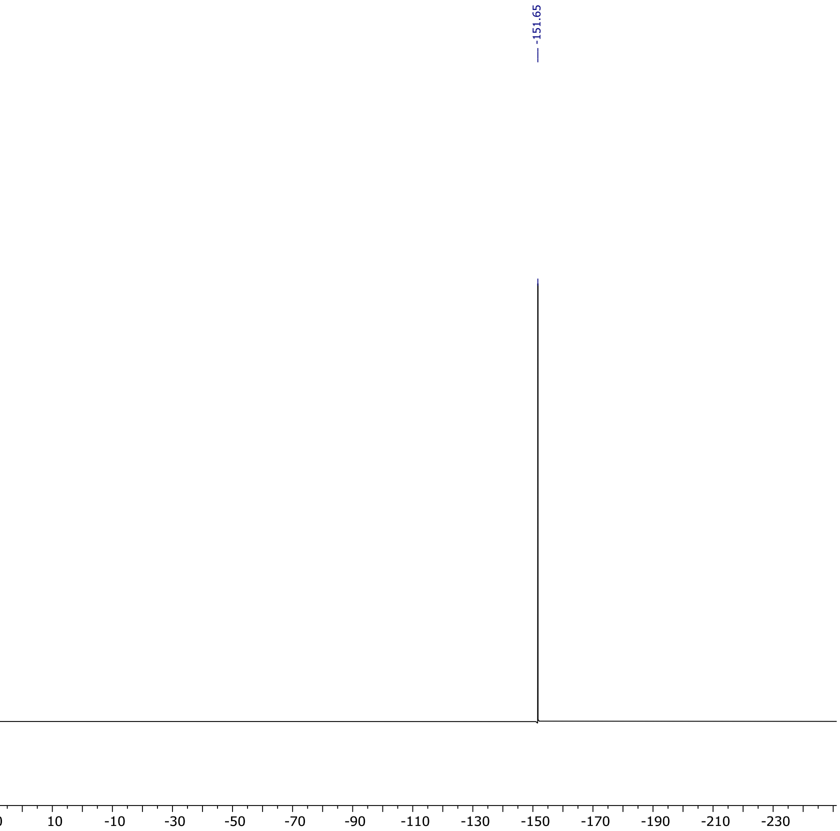

4-Fluoro-4'-methoxy-1,1'-biphenyl (compound 3)

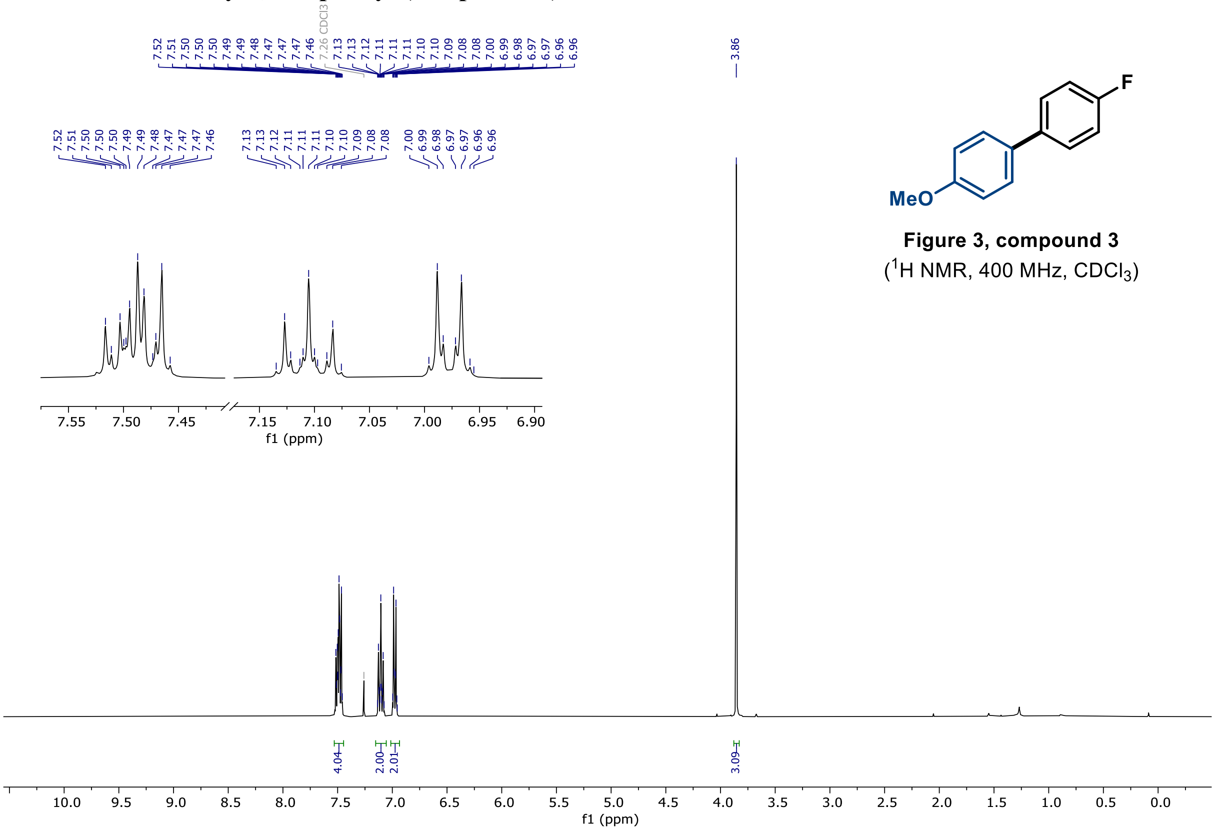

Figure 3, compound 3
(^{1}H NMR, 400 MHz, $CDCl_3$)

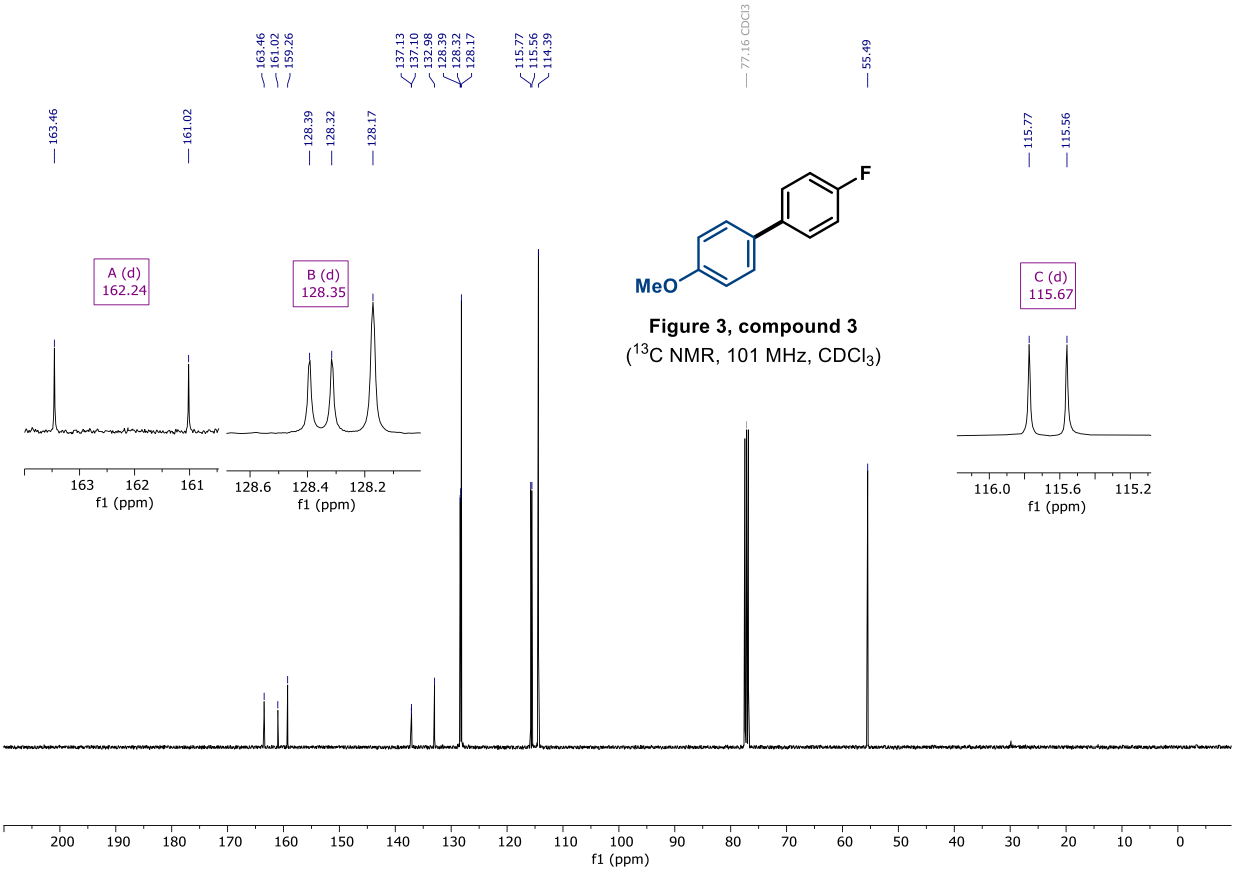

Figure 3, compound 3
(^{13}C NMR, 101 MHz, $CDCl_3$)

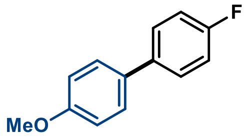

Figure 3, compound 3
(^{19}F NMR, 282 MHz, $CDCl_3$)

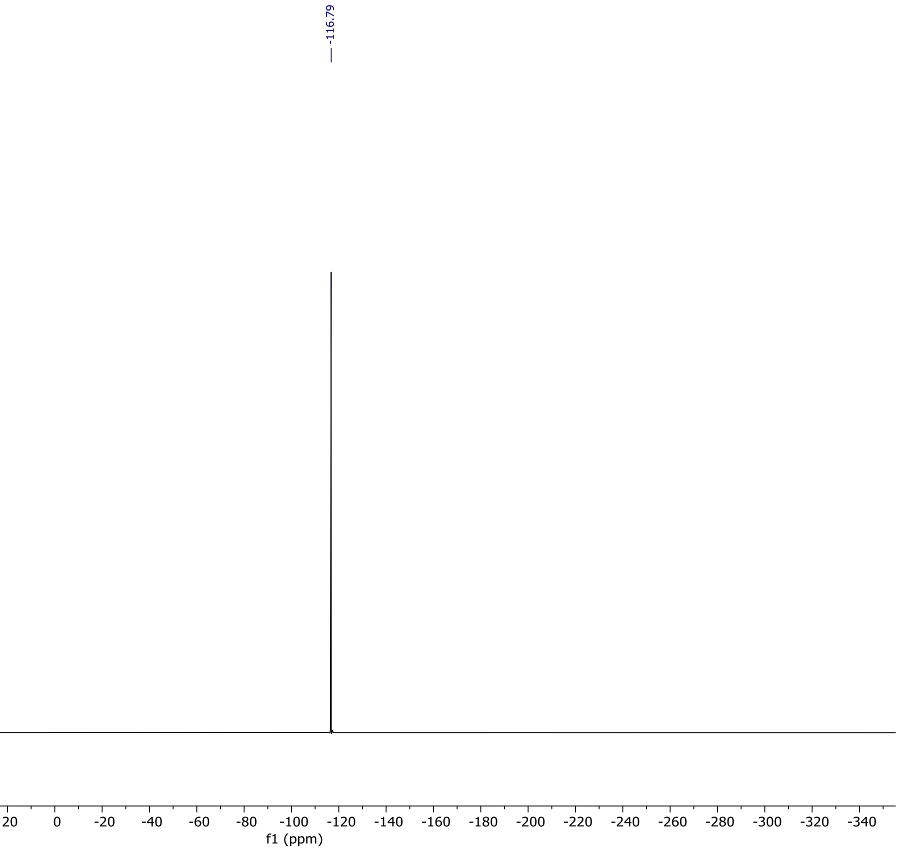

4-Nitro-1,1'-biphenyl (compound 4)

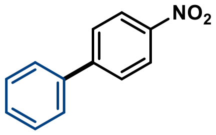

Figure 3, compound 4

(^{1}H NMR, 400 MHz, $CDCl_3$)

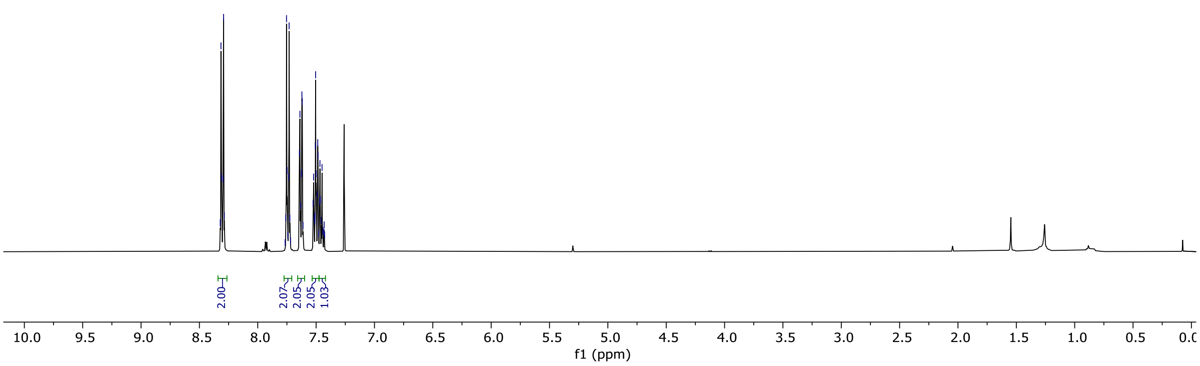

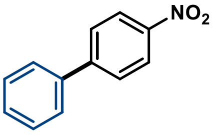

Figure 3, compound 4

(^{13}C NMR, 101 MHz, $CDCl_3$)

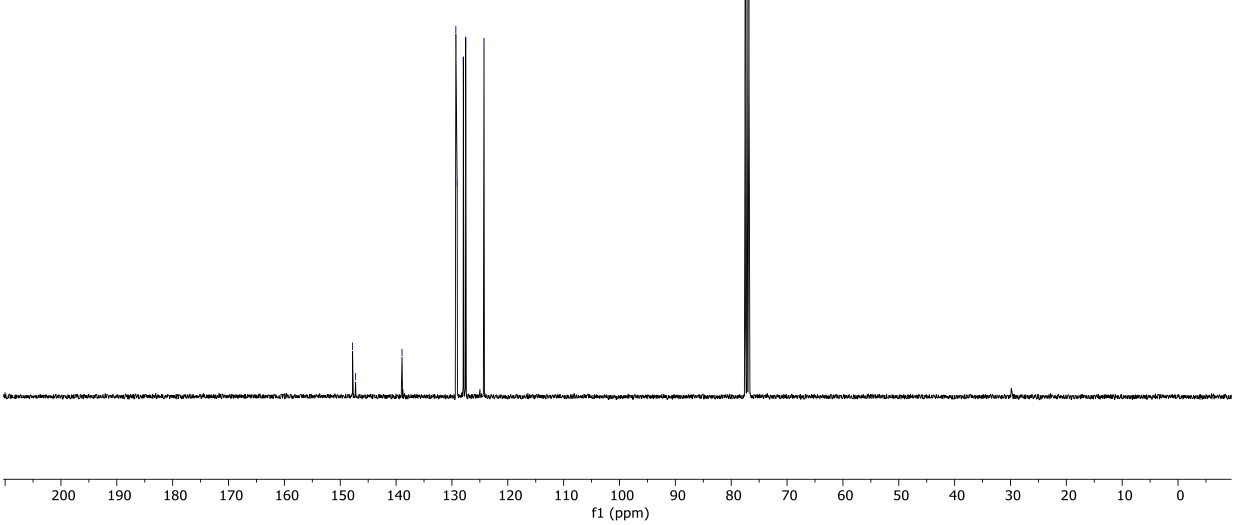

4-(Methylsulfonyl)-1,1'-biphenyl (compound 5)

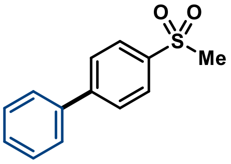

Figure 3, compound 5
(^{1}H NMR, 600 MHz, $CDCl_3$)

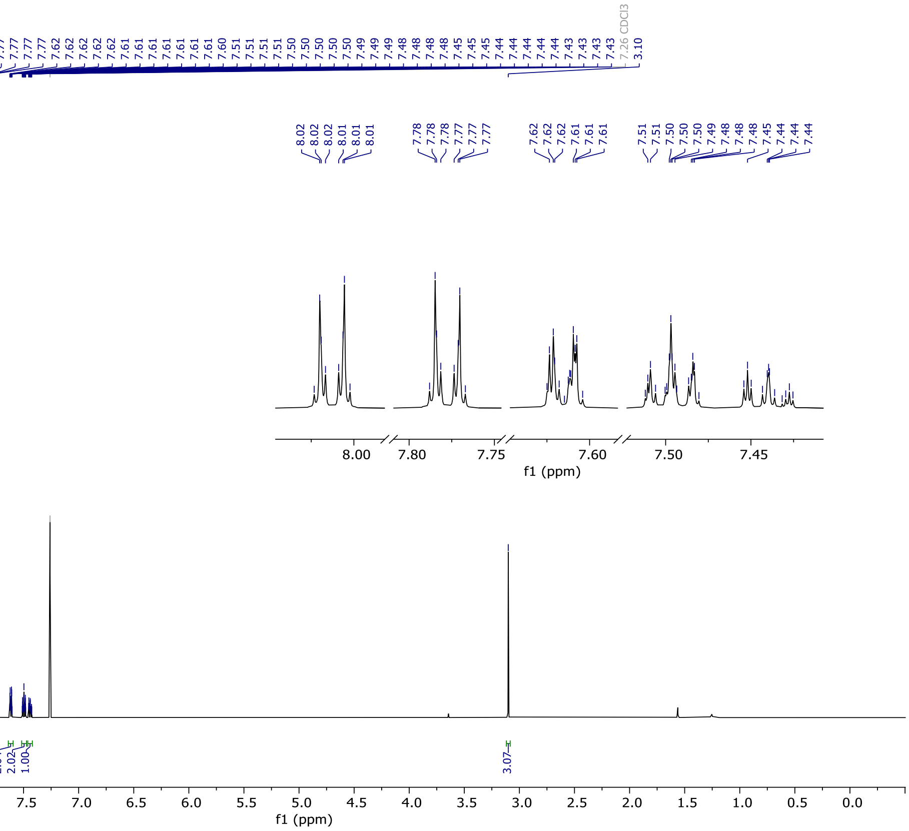

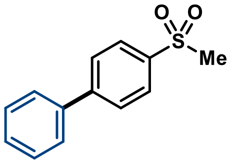

Figure 3, compound 5
(^{13}C NMR, 151 MHz, $CDCl_3$)

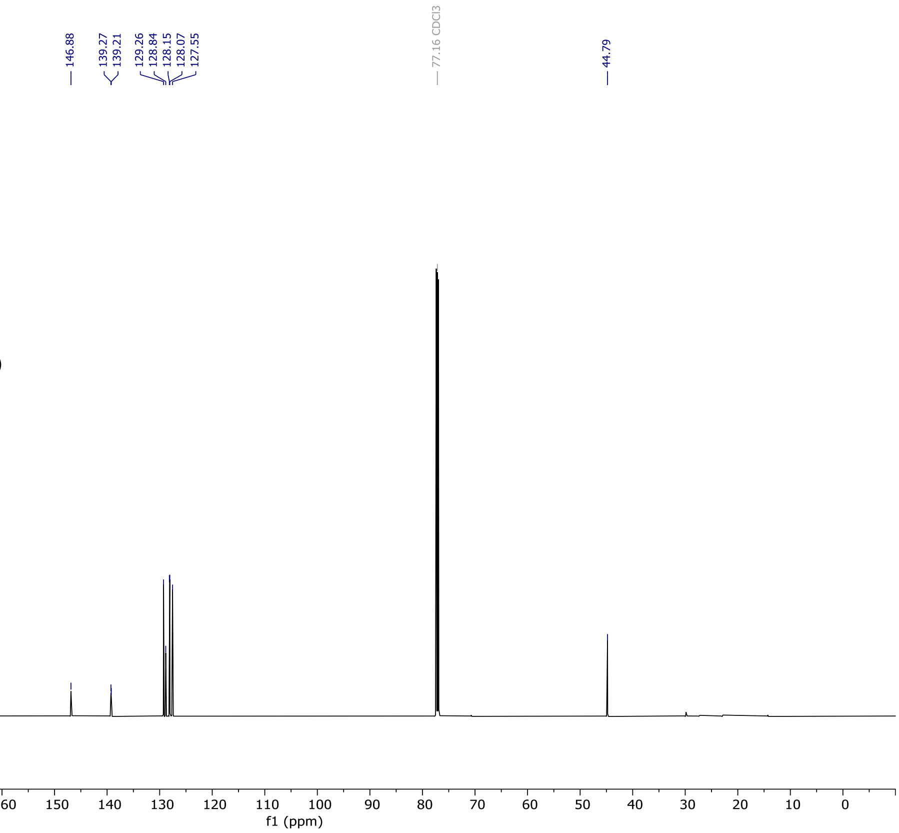

[1,1'-Biphenyl]-3-carbonitrile (compound 6)

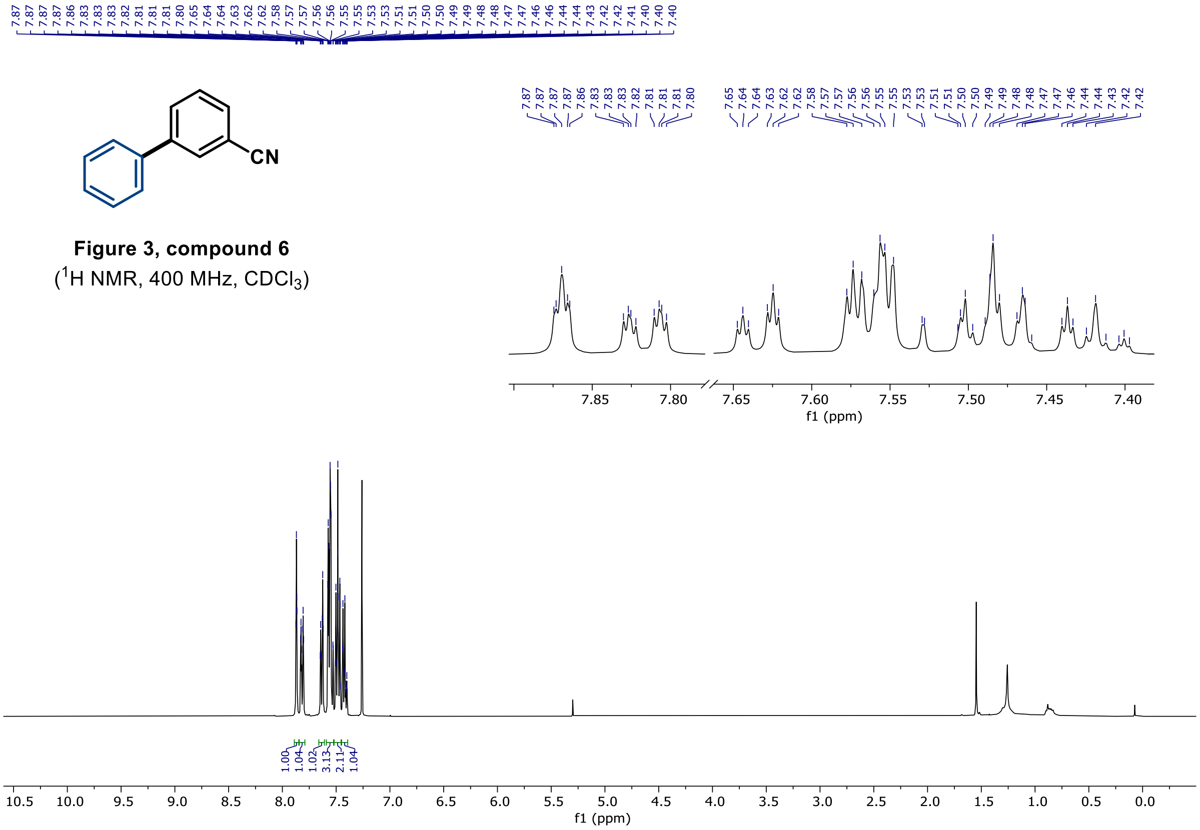

Figure 3, compound 6

(^{1}H NMR, 400 MHz, $CDCl_3$)

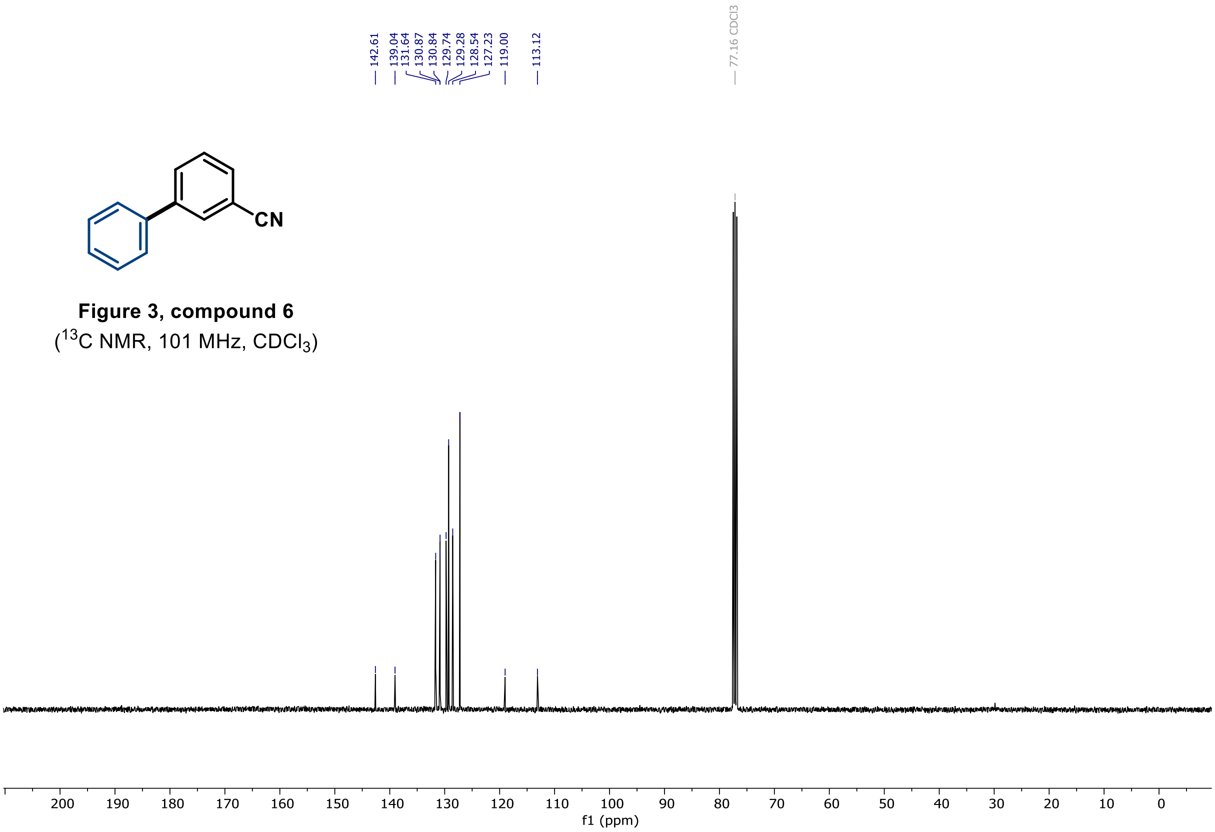

Figure 3, compound 6

(^{13}C NMR, 101 MHz, $CDCl_3$)

4'-Methoxy-[1,1'-biphenyl]-2-carbaldehyde (compound 7)

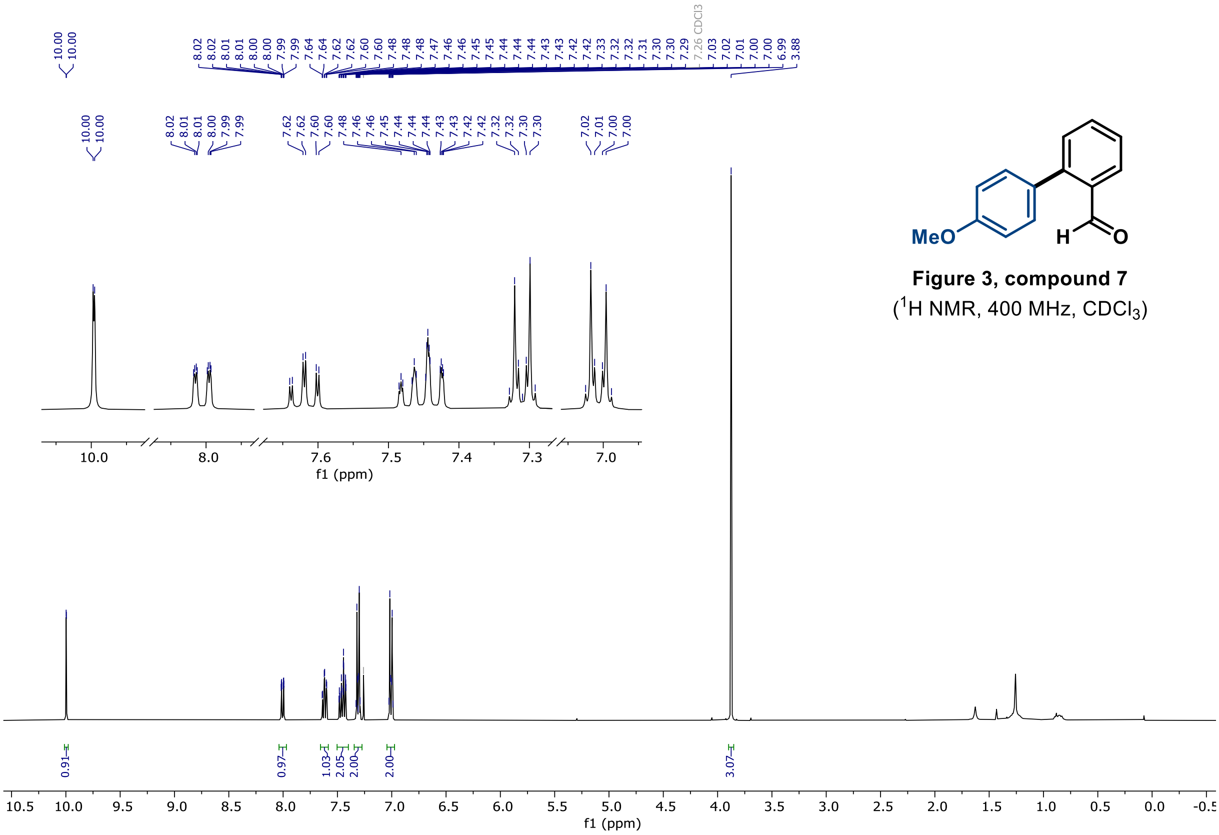

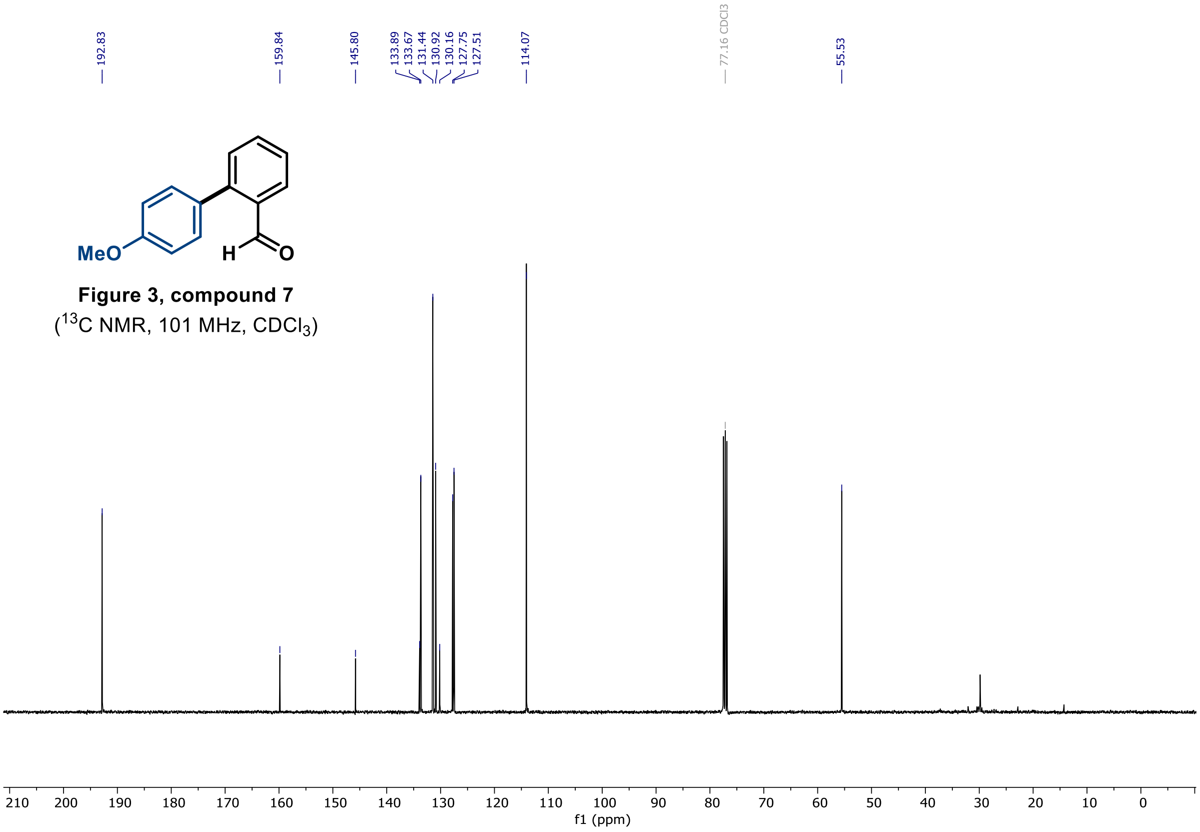

4-Ethyl-4'-methoxy-1,1'-biphenyl (compound 8)

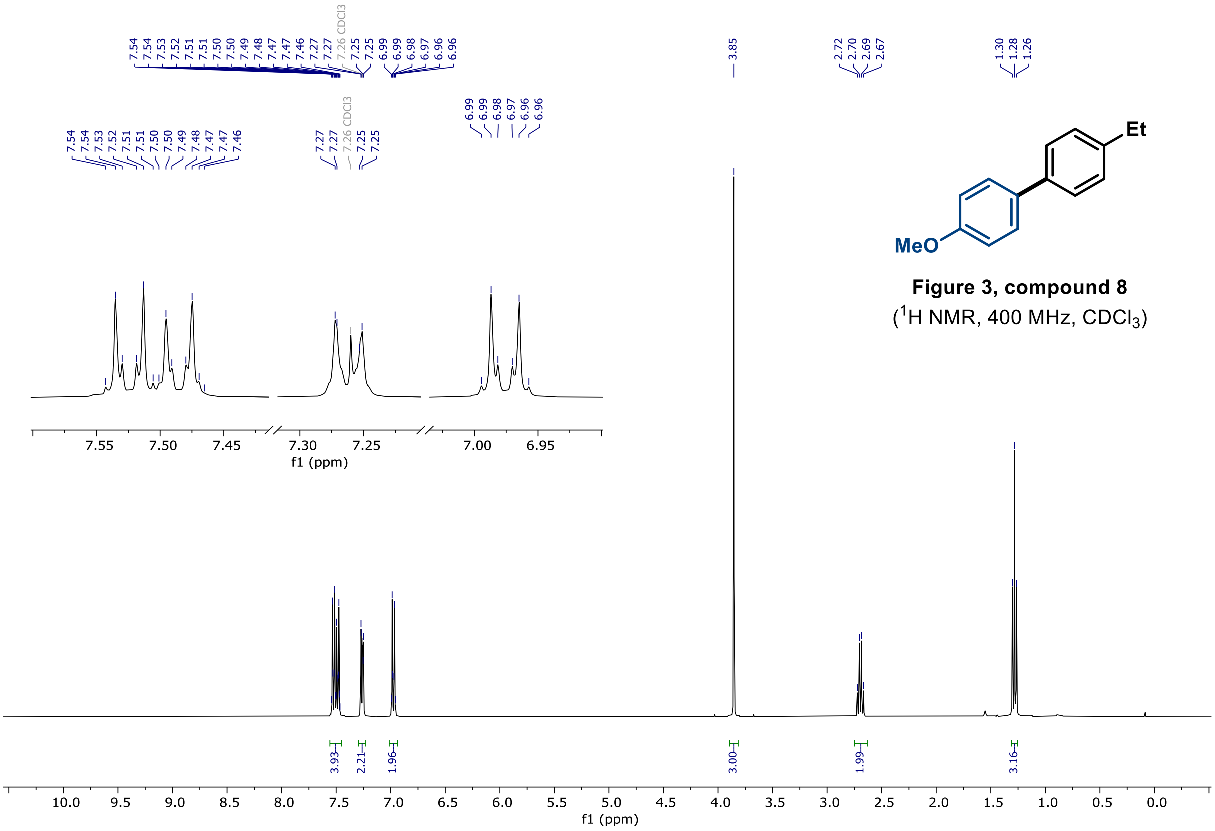

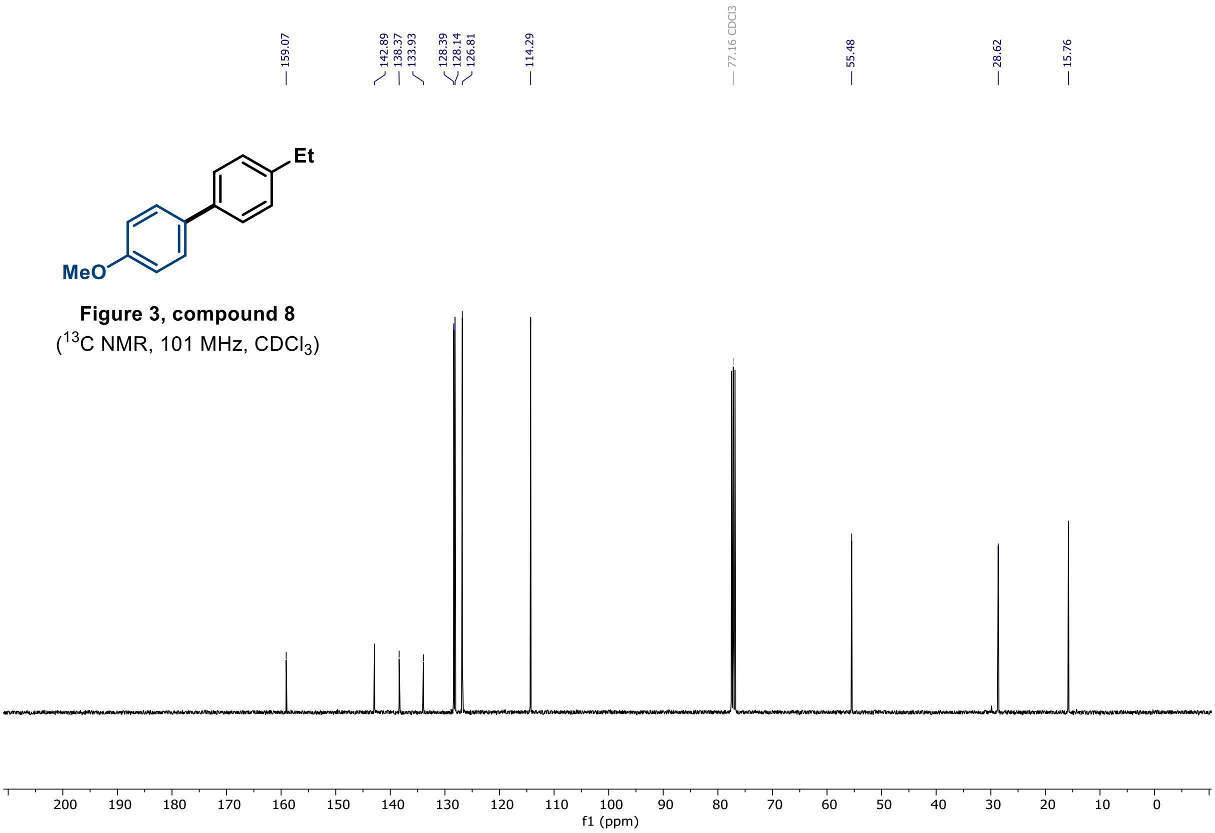

2-Phenyl-9H-fluorene (compound 9)

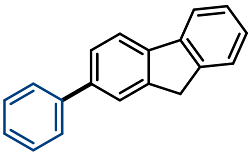

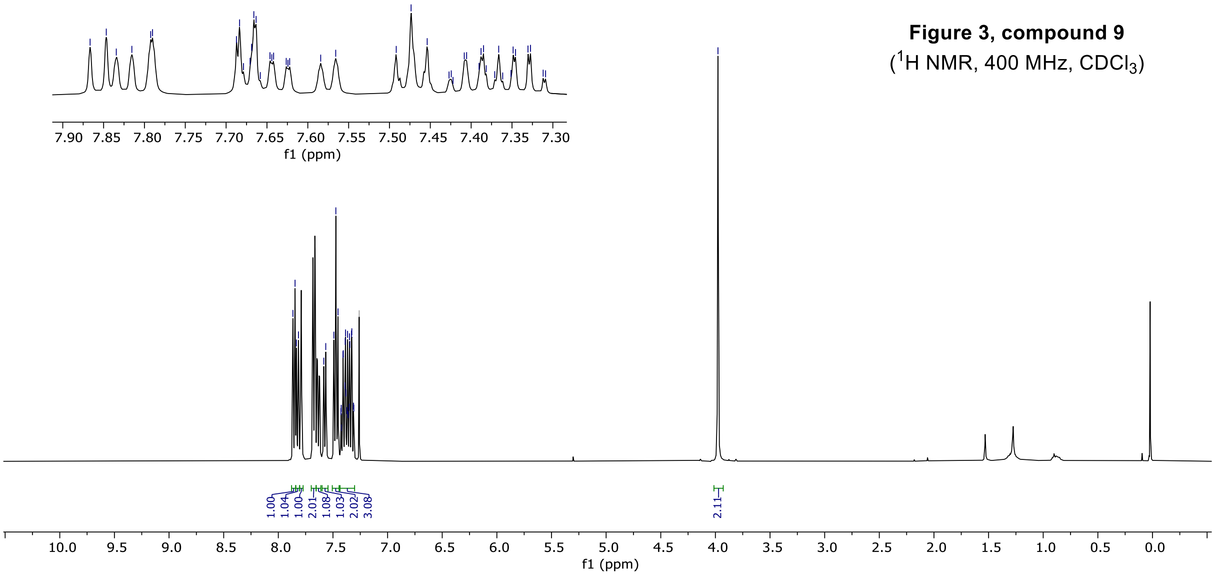

Figure 3, compound 9
(^{1}H NMR, 400 MHz, $CDCl_3$)

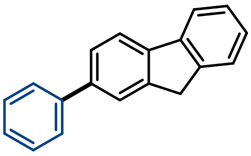

Figure 3, compound 9
(^{13}C NMR, 101 MHz, $CDCl_3$)

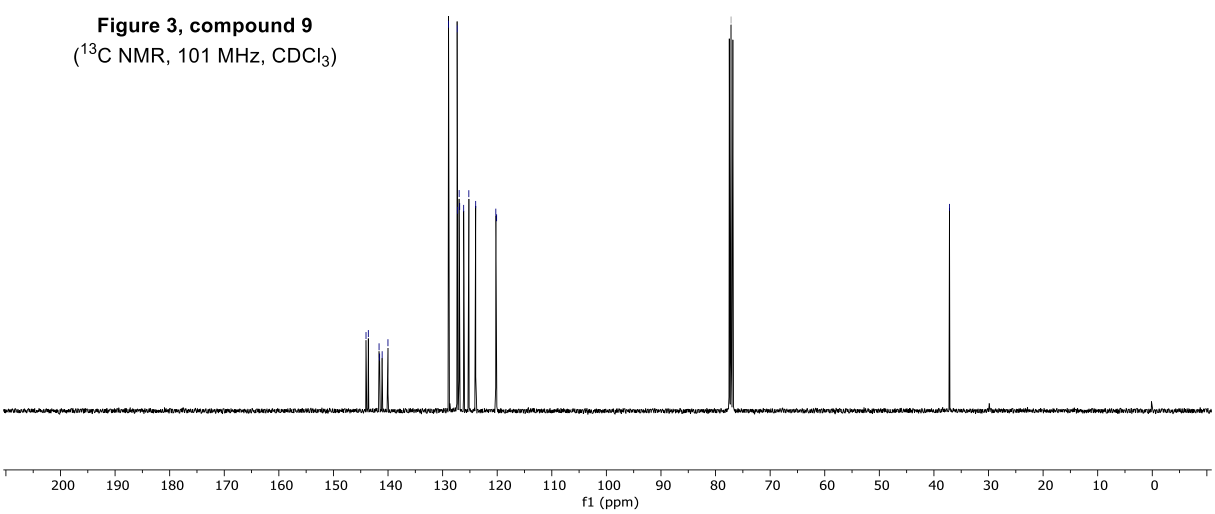

1-(4-Methoxyphenyl)naphthalene (compound 10)

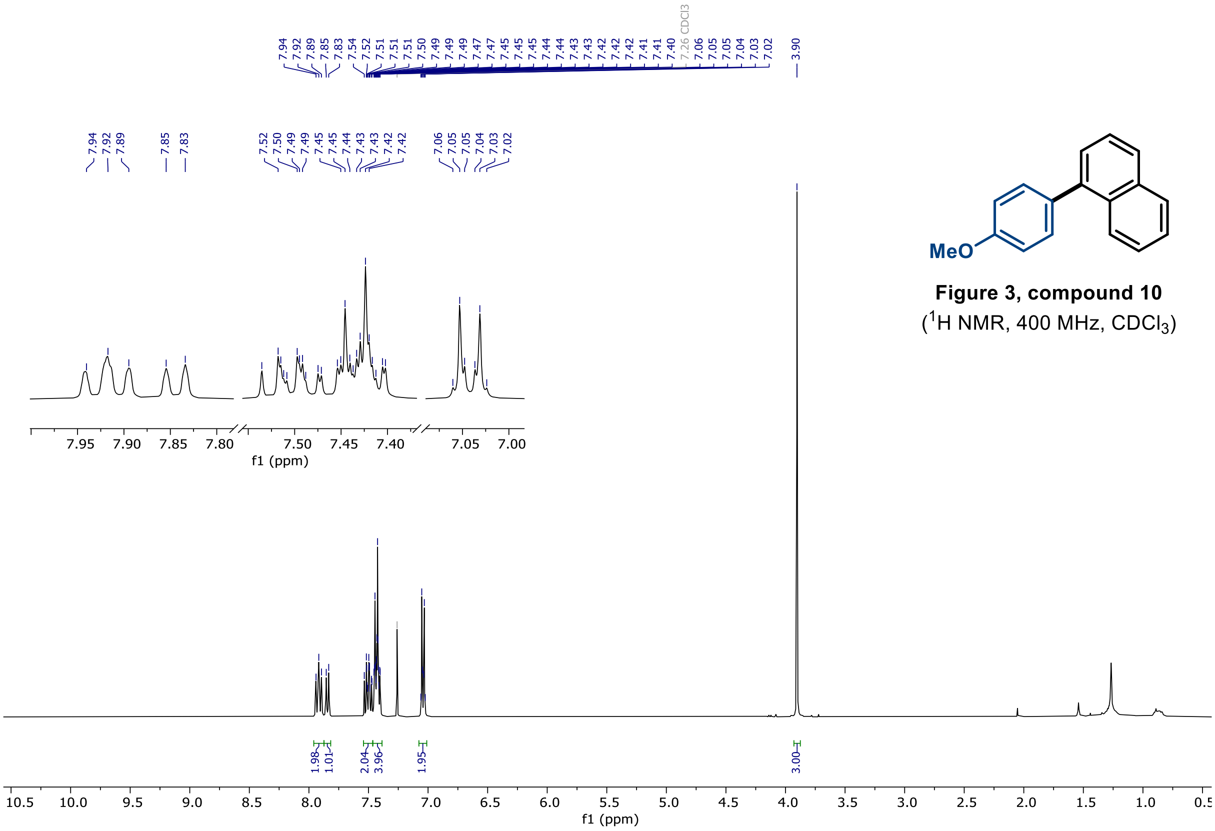

Figure 3, compound 10
(^{1}H NMR, 400 MHz, $CDCl_3$)

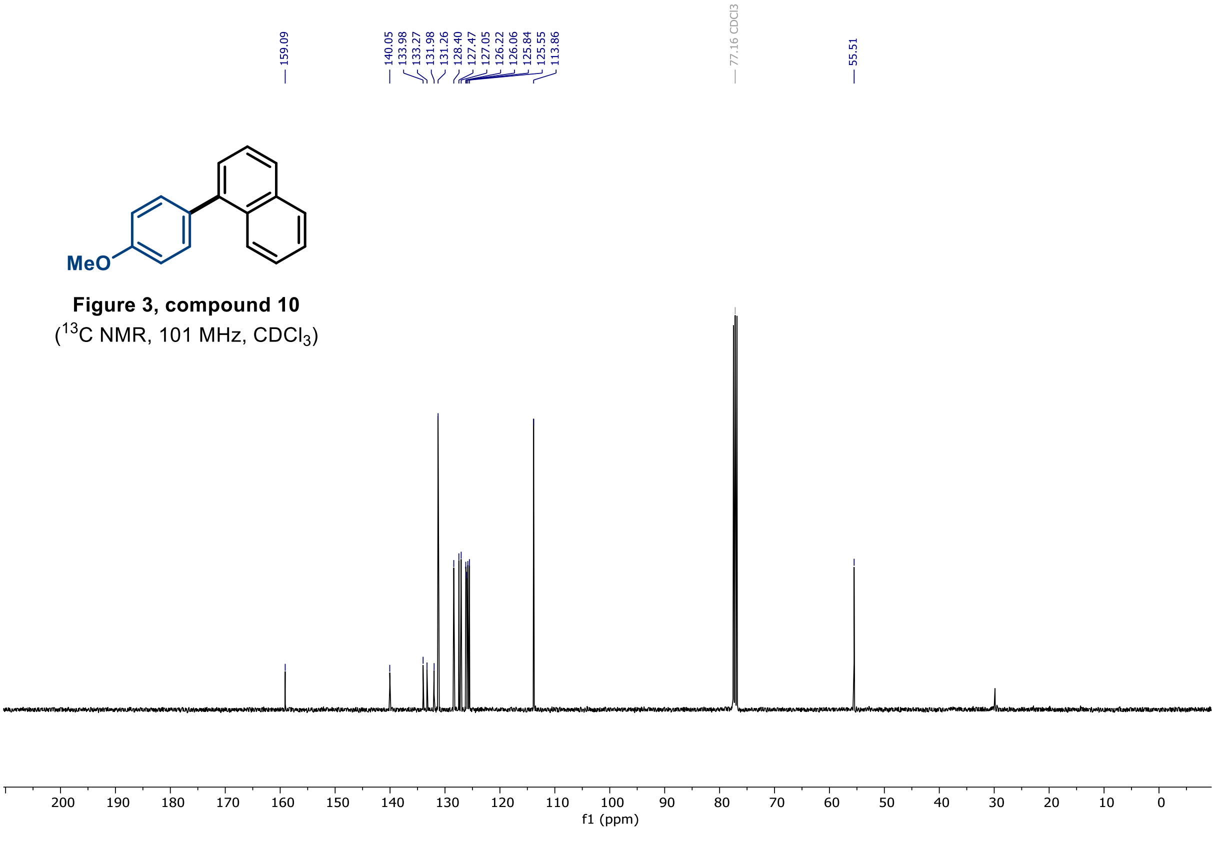

Figure 3, compound 10
(^{13}C NMR, 101 MHz, $CDCl_3$)

4-Bromo-4'-methoxy-1,1'-biphenyl (compound 11)

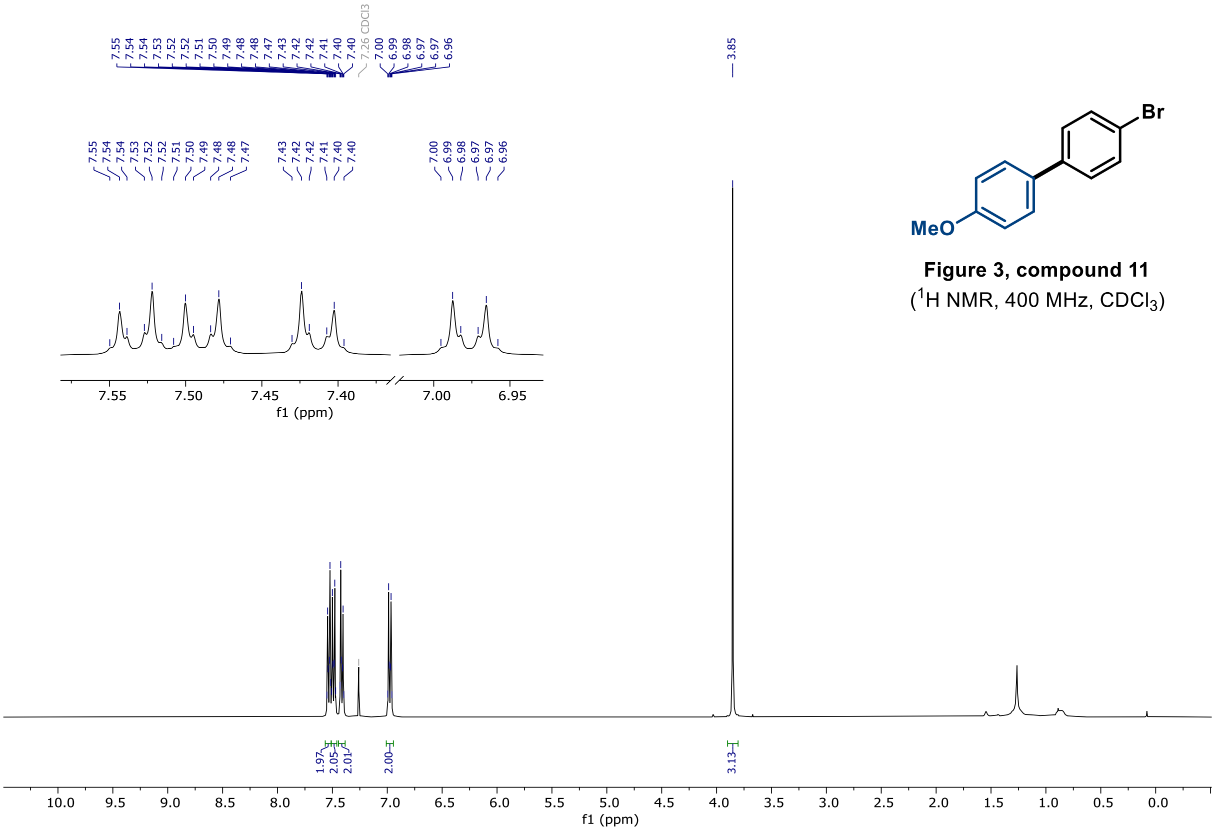

Figure 3, compound 11
(^1H NMR, 400 MHz, $CDCl_3$)

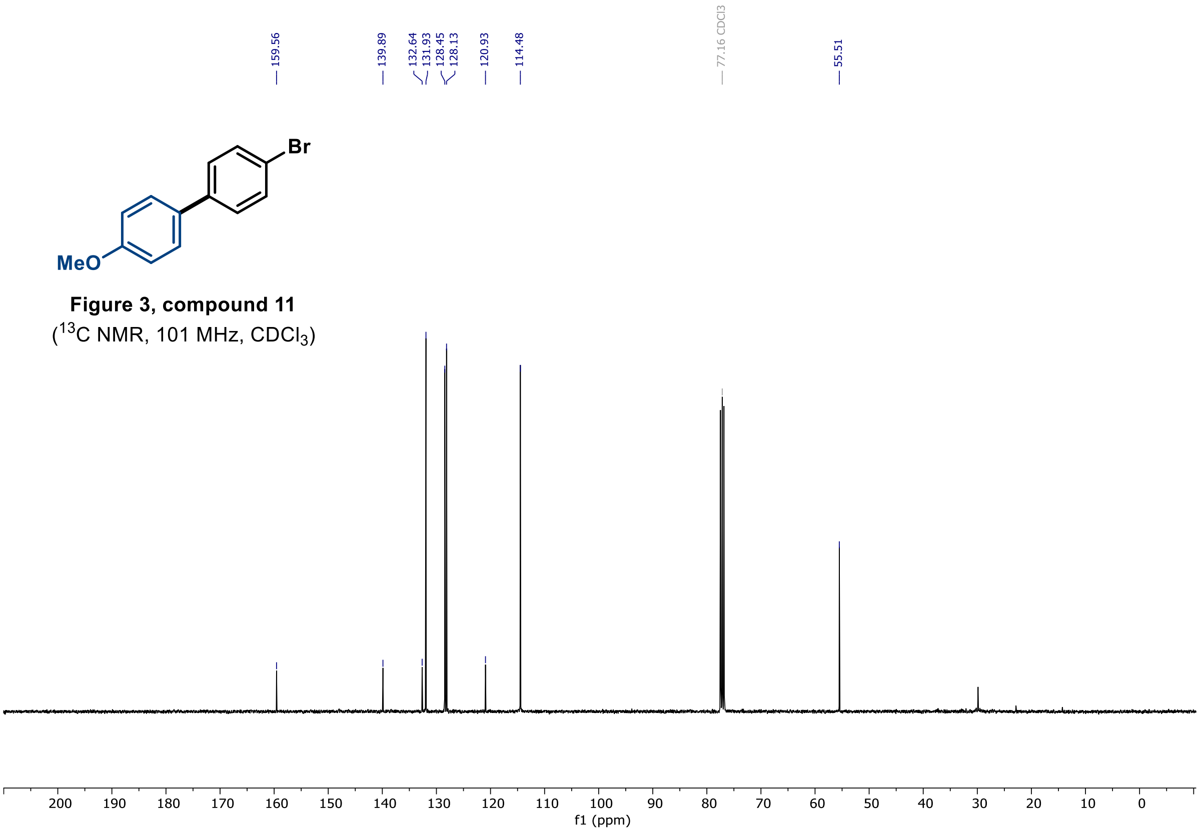

Figure 3, compound 11
(^{13}C NMR, 101 MHz, $CDCl_3$)

4-Chloro-4'-methoxy-2-methyl-1,1'-biphenyl (compound 12)

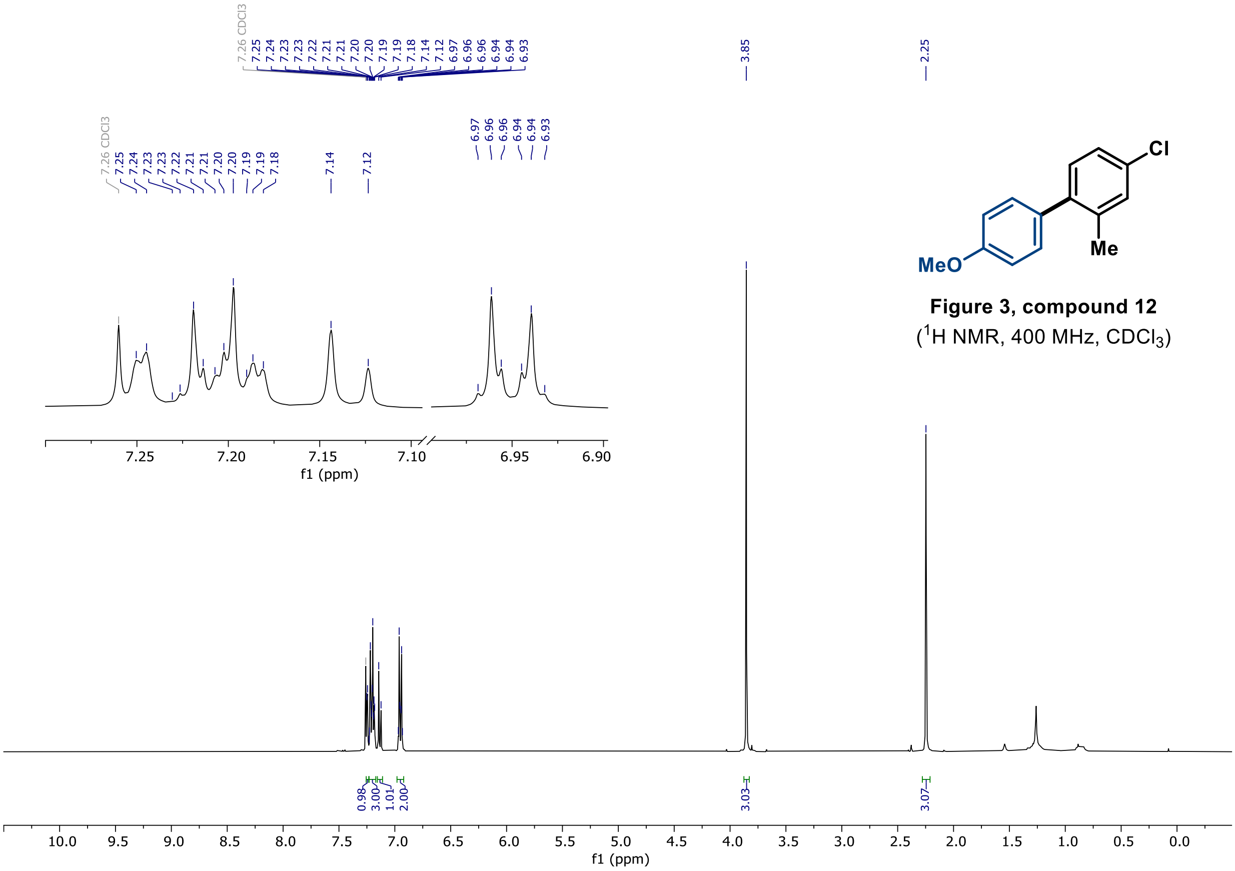

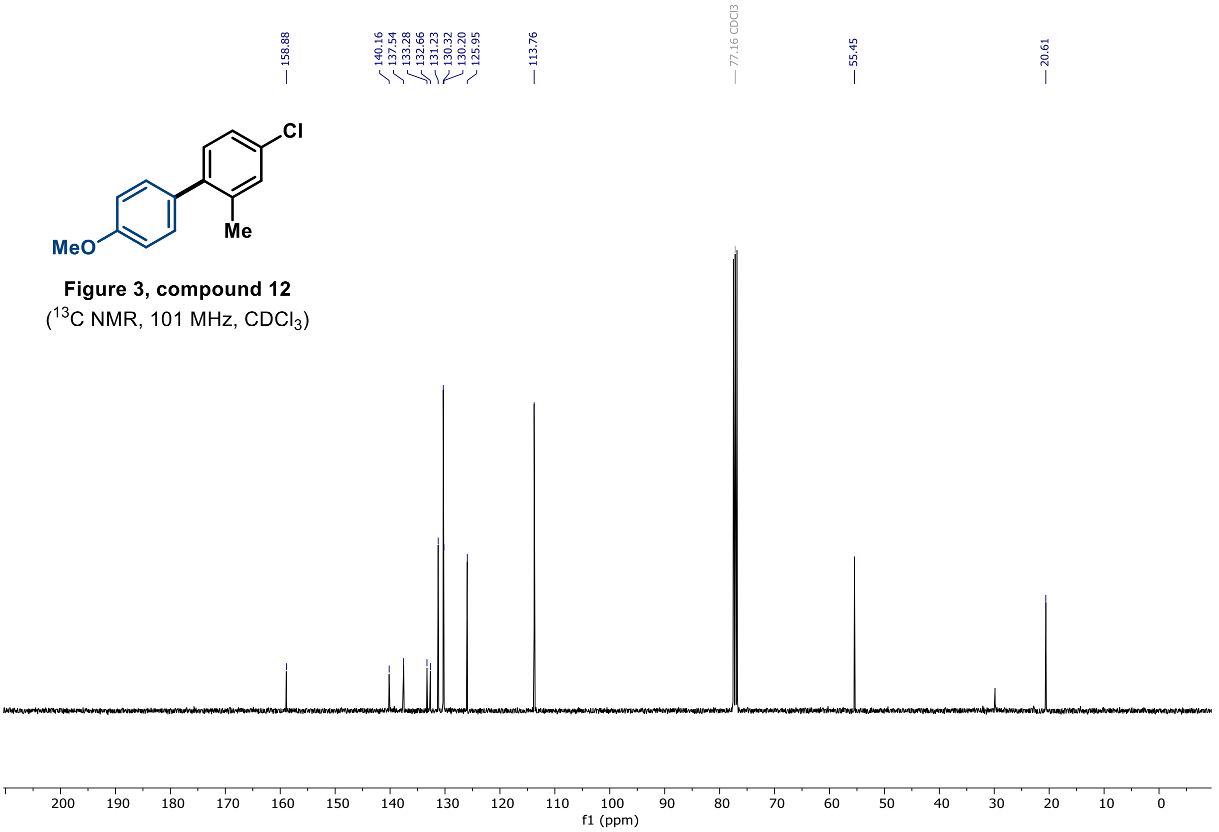

[1,1'-Biphenyl]-4-yl trifluoromethanesulfonate (compound 13)

Figure 3, compound 13
(^{1}H NMR, 400 MHz, $CDCl_3$)

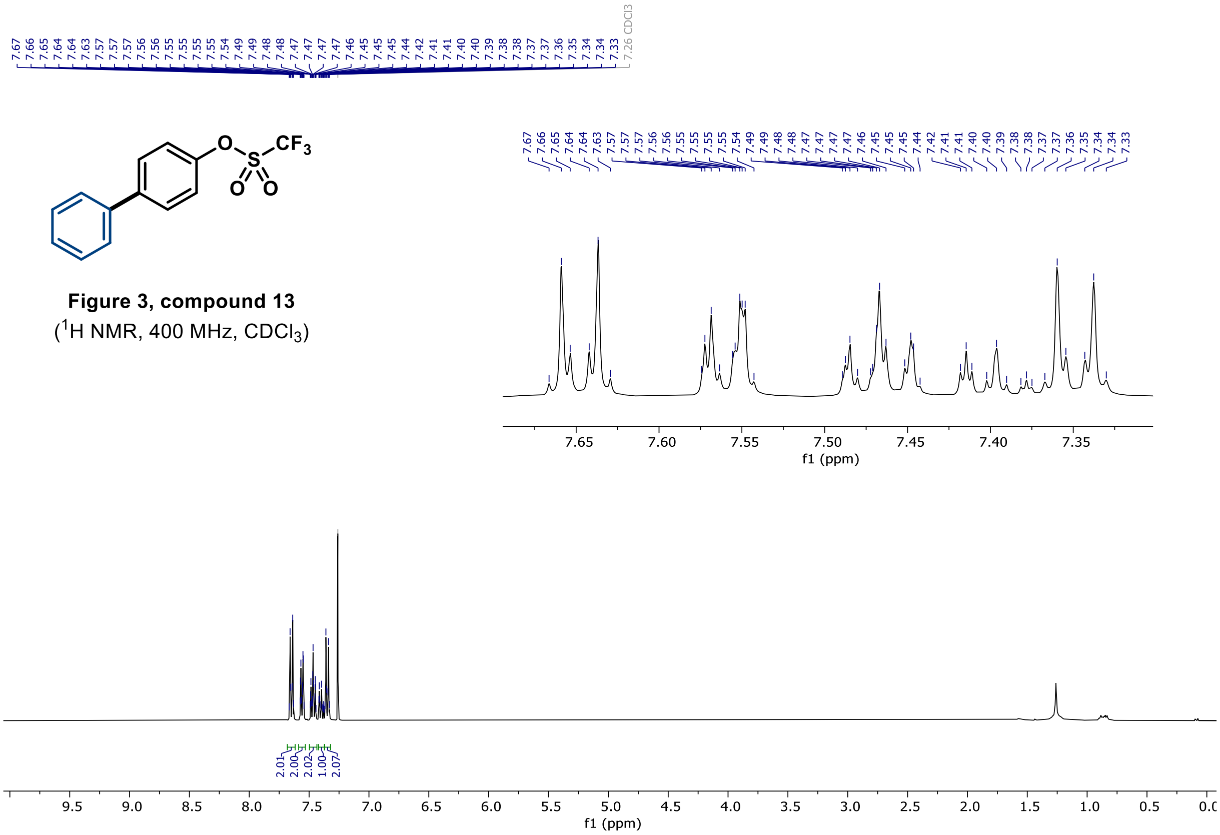

Figure 3, compound 13
(^{13}C NMR, 101 MHz, $CDCl_3$)

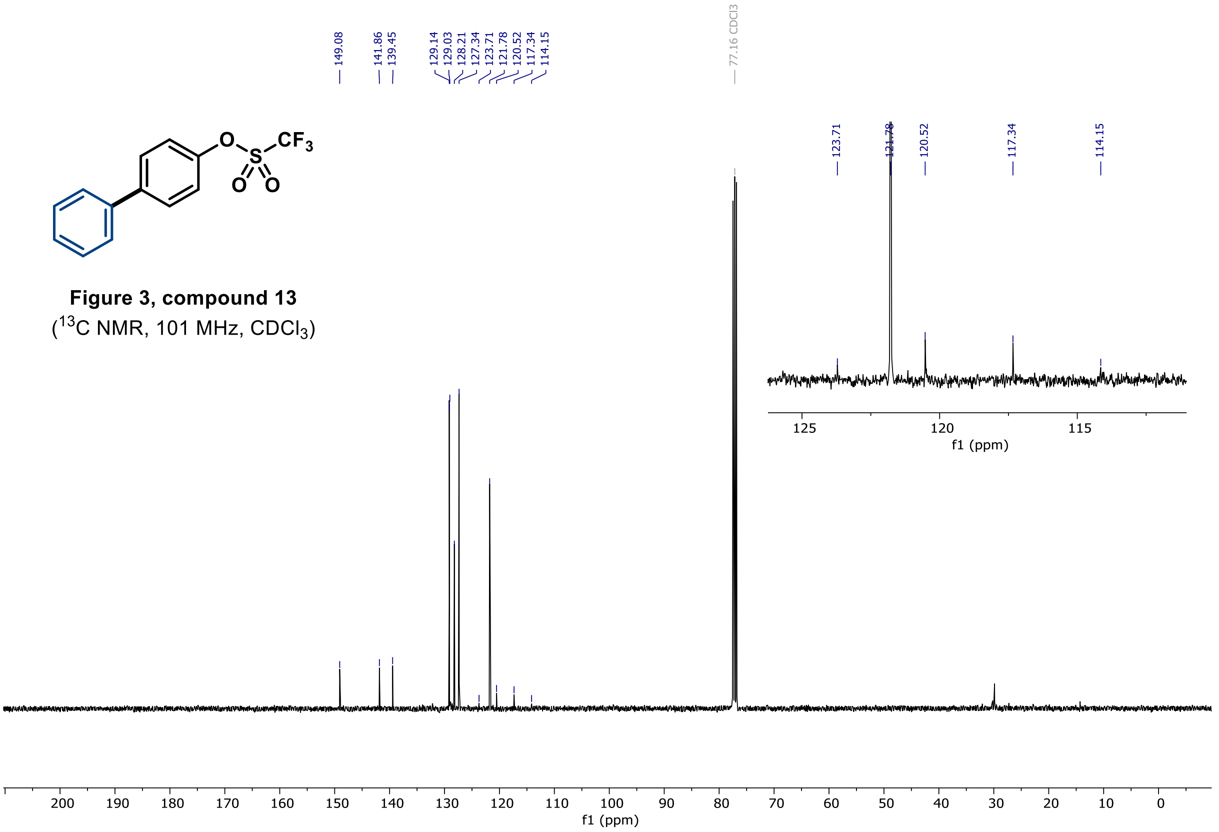

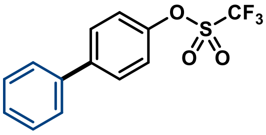

Figure 3, compound 13
(^{13}C NMR, 101 MHz, $CDCl_3$)

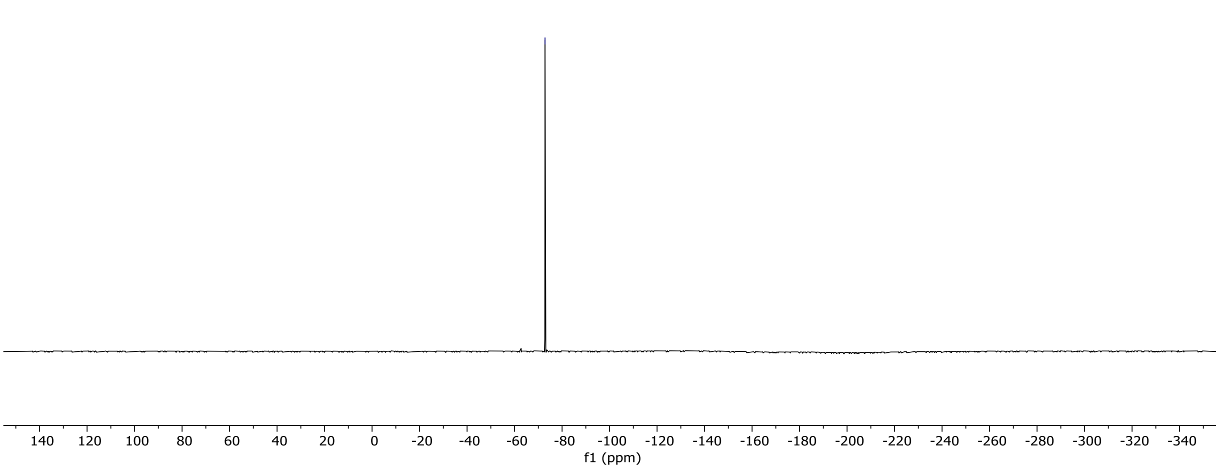

3-Phenyldibenzo[b,d]thiophene (compound 14)

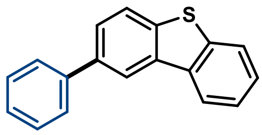

Figure 3, compound 14
(^{1}H NMR, 400 MHz, $CDCl_3$)

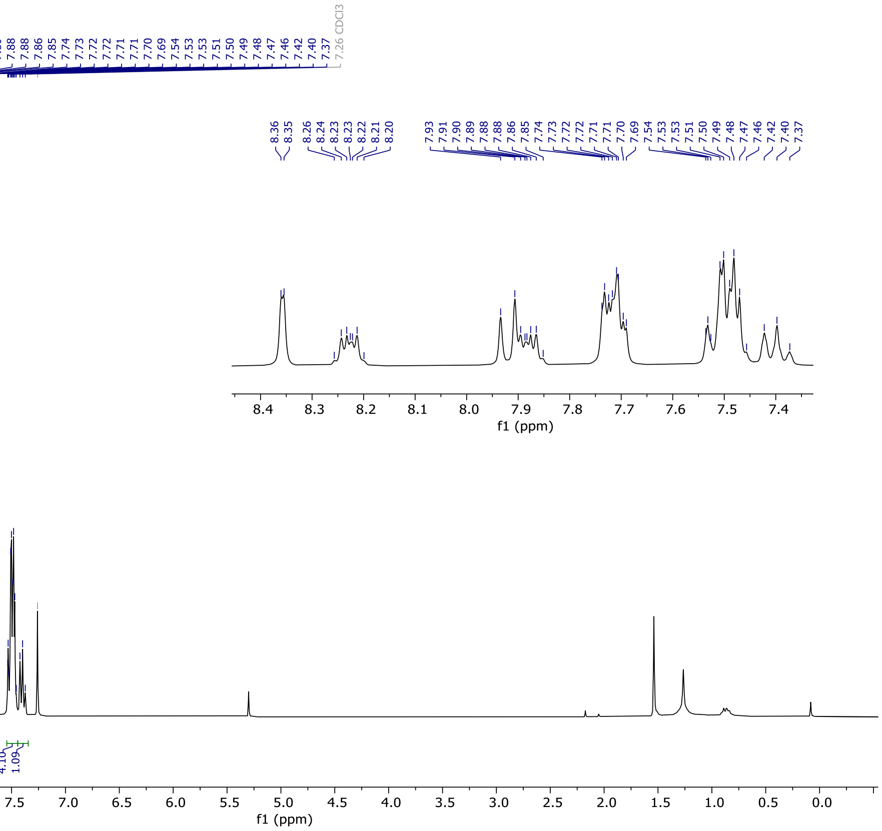

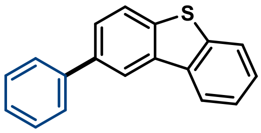

Figure 3, compound 14
(^{13}C NMR, 101 MHz, $CDCl_3$)

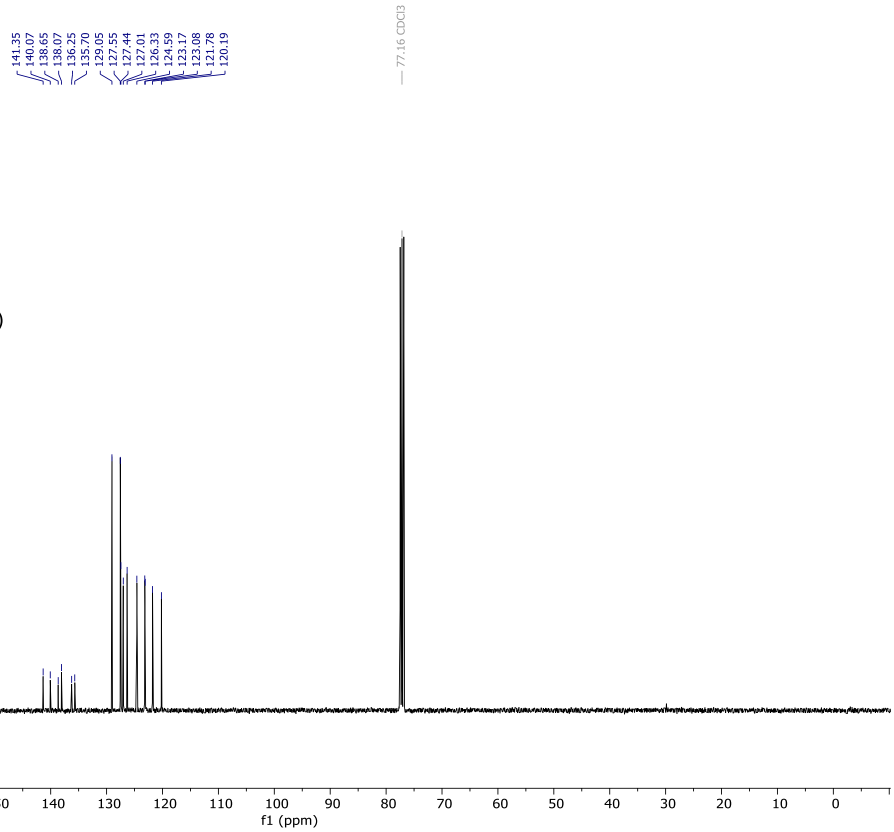

3-Phenyldibenzo[b,d]furan (compound 15)

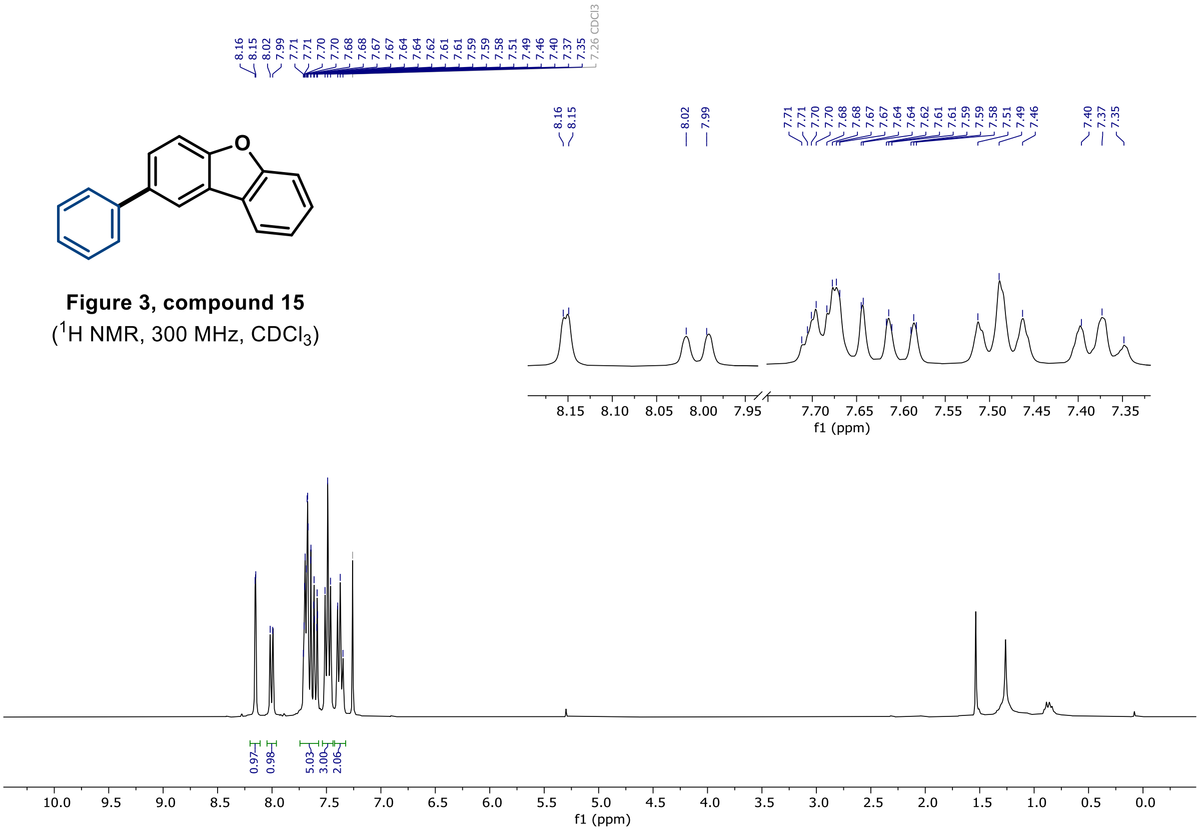

Figure 3, compound 15
(^{1}H NMR, 300 MHz, $CDCl_3$)

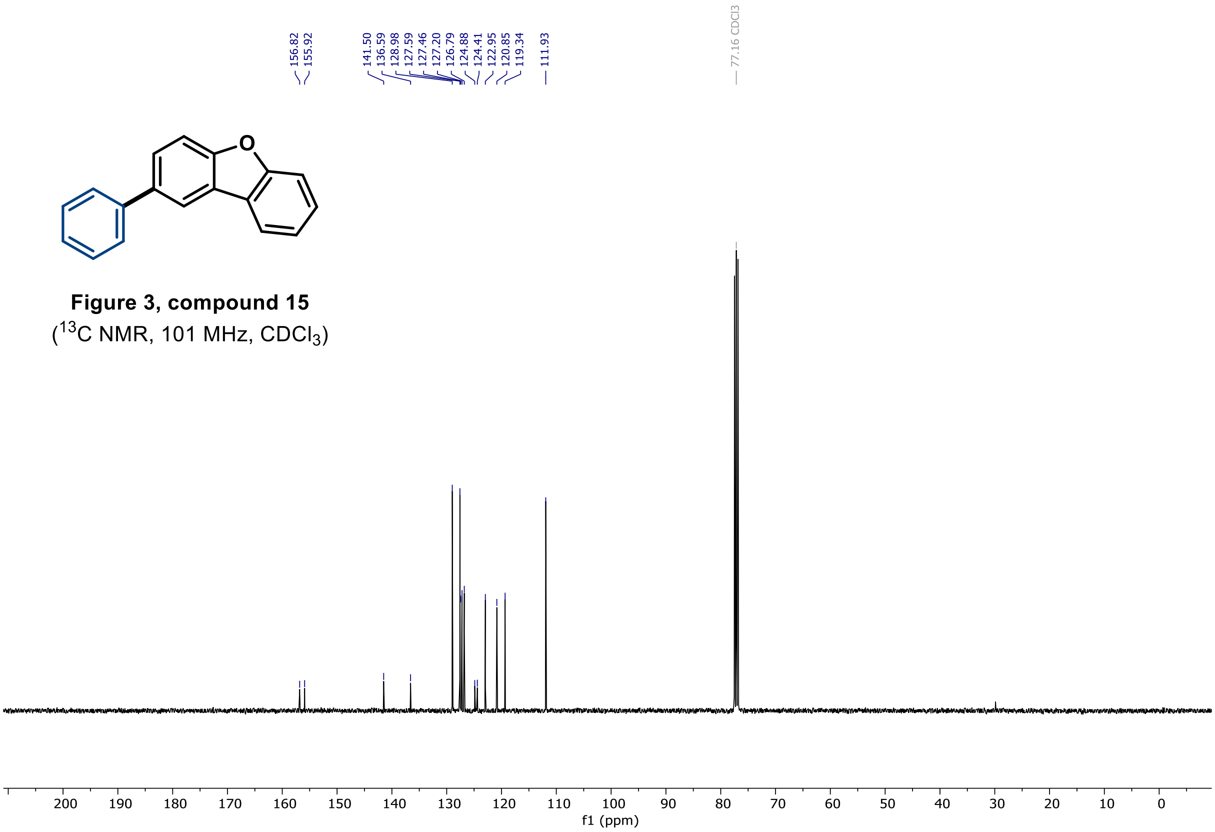

Figure 3, compound 15
(^{13}C NMR, 101 MHz, $CDCl_3$)

2-(4-Methoxyphenyl)thiophene (compound 16)

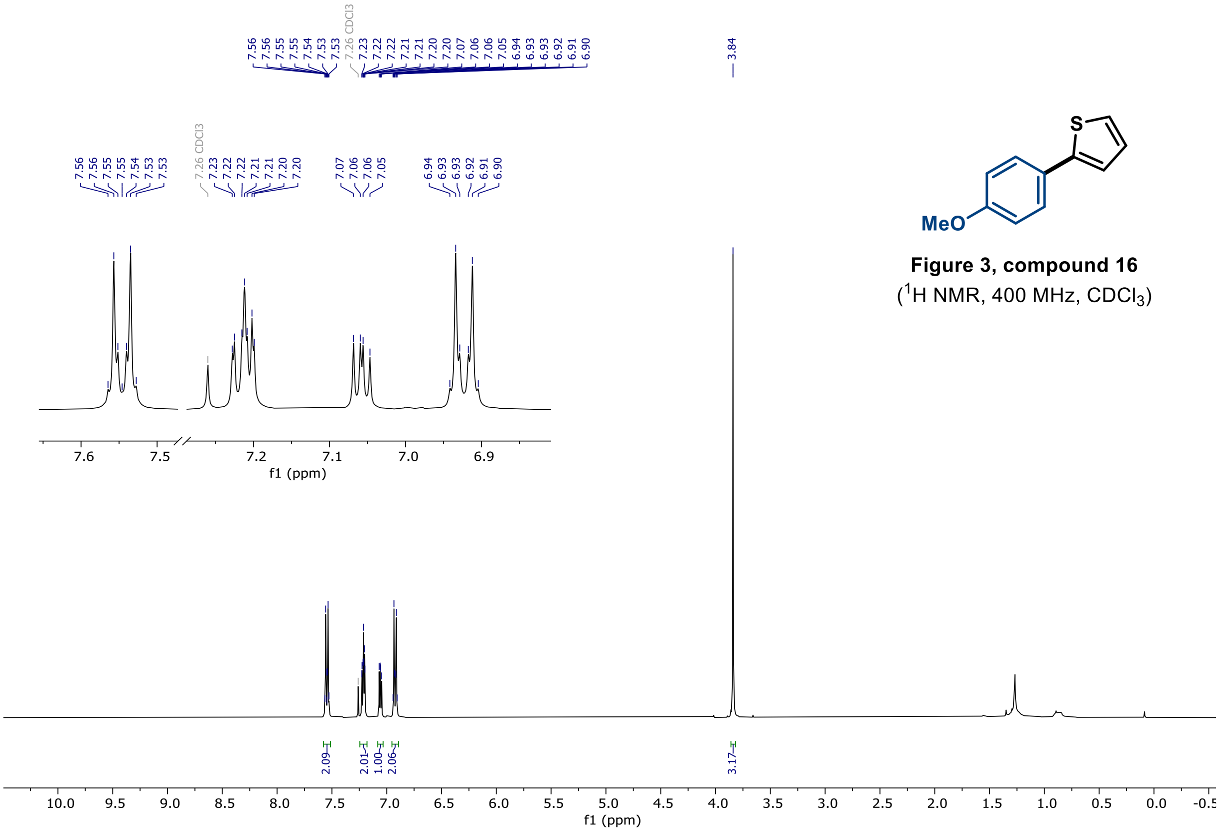

Figure 3, compound 16
(^{1}H NMR, 400 MHz, $CDCl_3$)

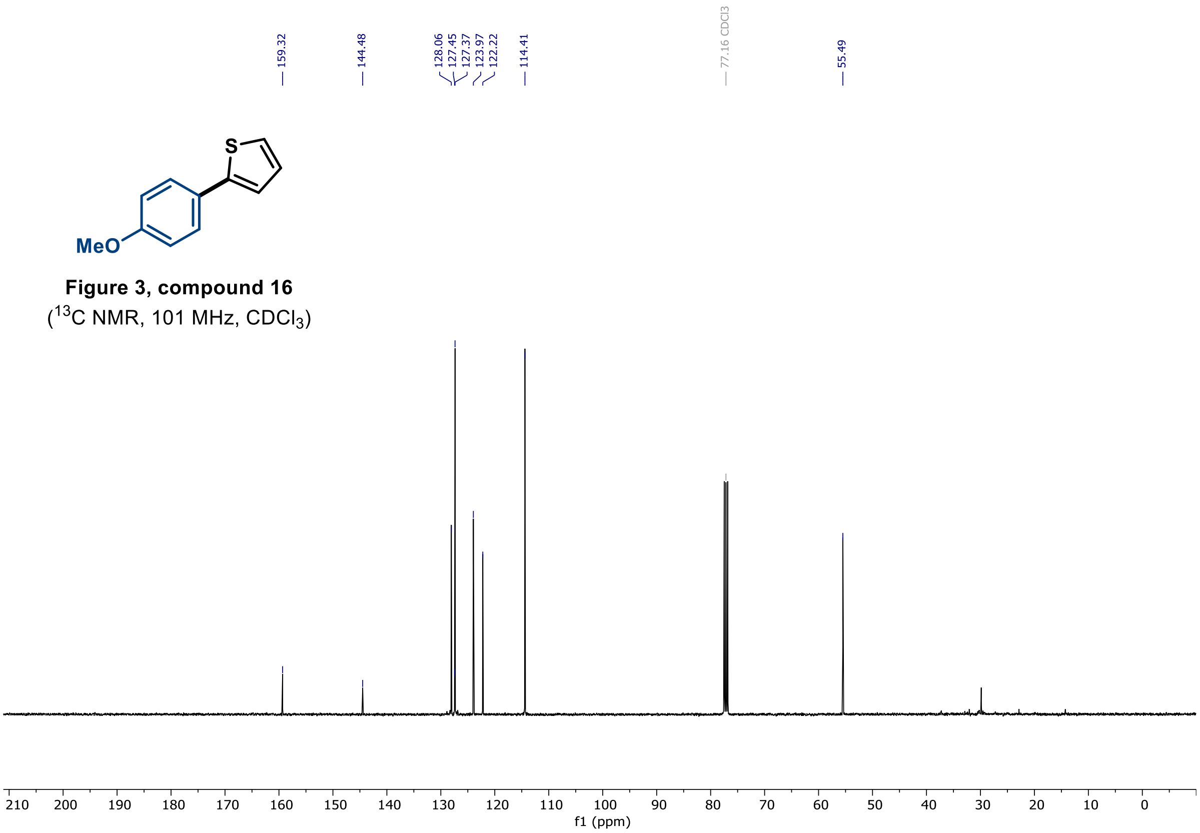

Figure 3, compound 16
(^{13}C NMR, 101 MHz, $CDCl_3$)

4-(4-Methoxyphenyl)pyridine (compound 17)

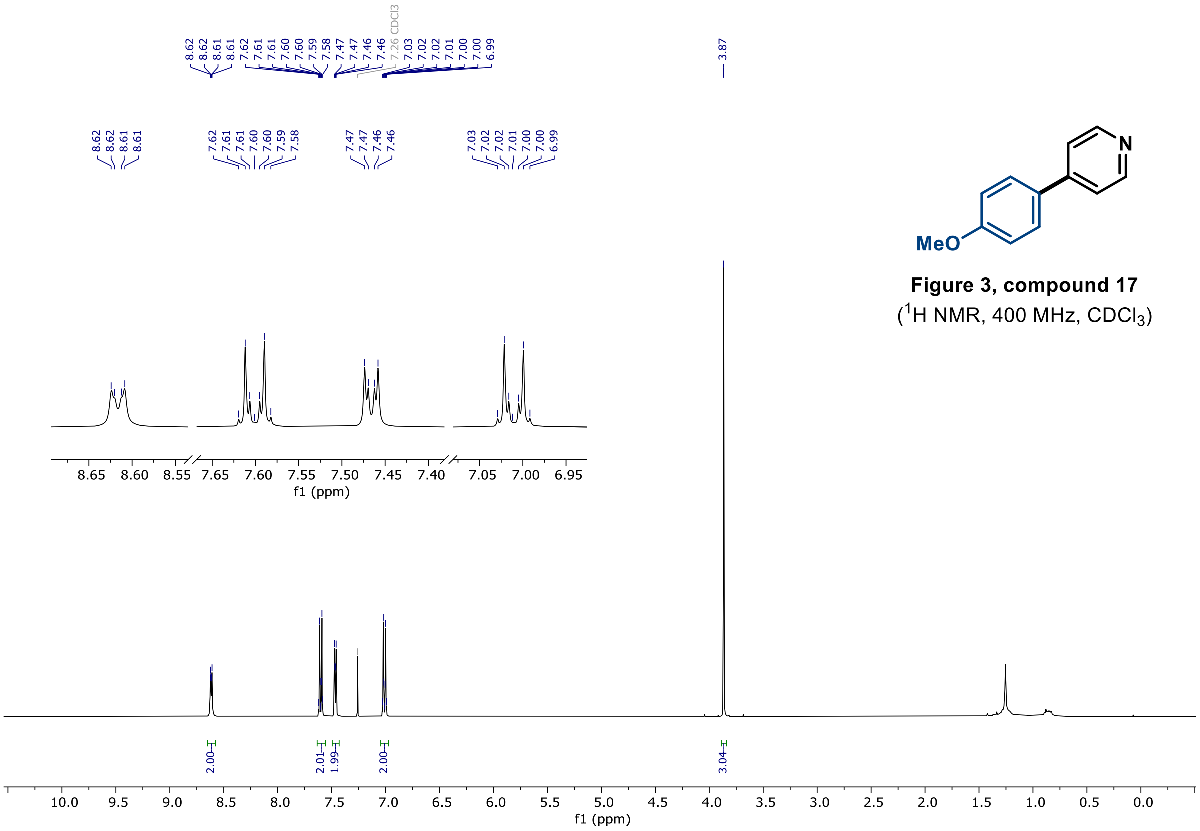

Figure 3, compound 17
(^{1}H NMR, 400 MHz, $CDCl_3$)

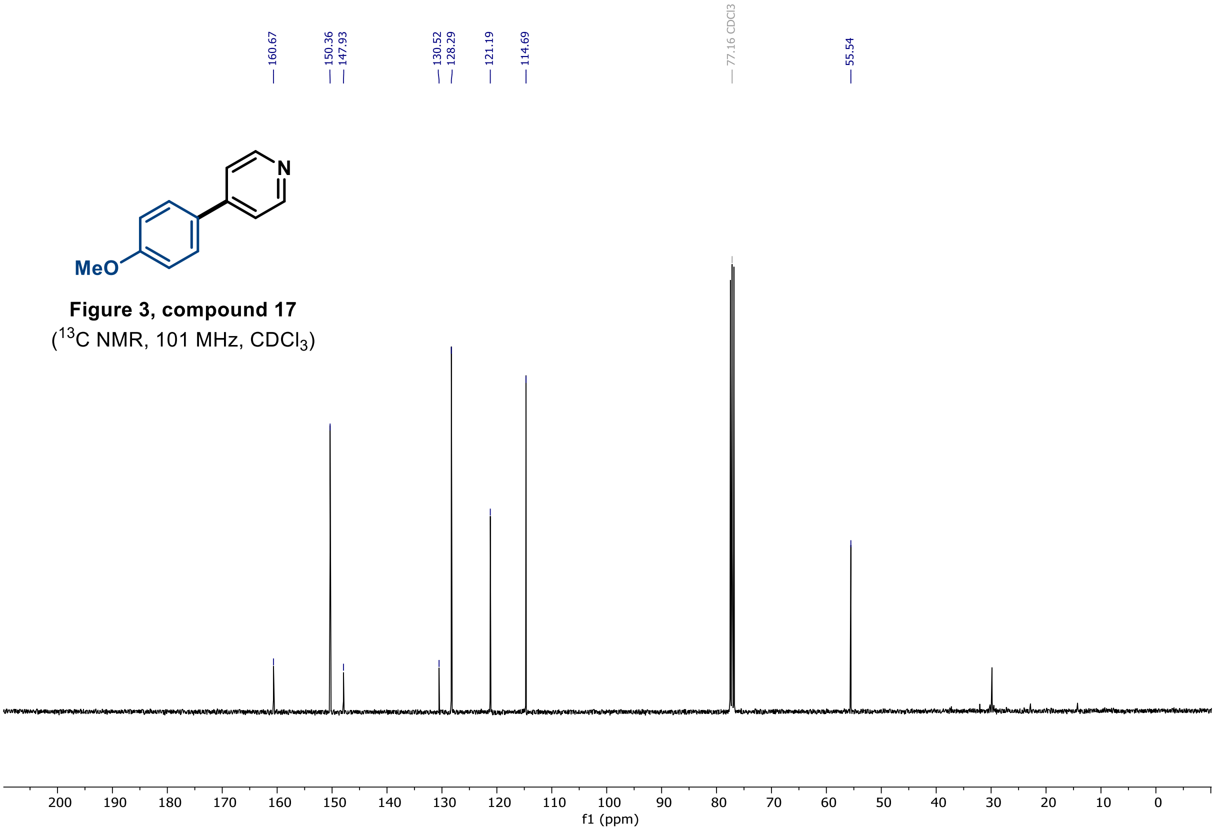

Figure 3, compound 17
(^{13}C NMR, 101 MHz, $CDCl_3$)

N-(3-chloro-4-((3-fluorobenzyl)oxy)phenyl)-6-(4-methoxyphenyl)quinazolin-4-amine (compound 18)

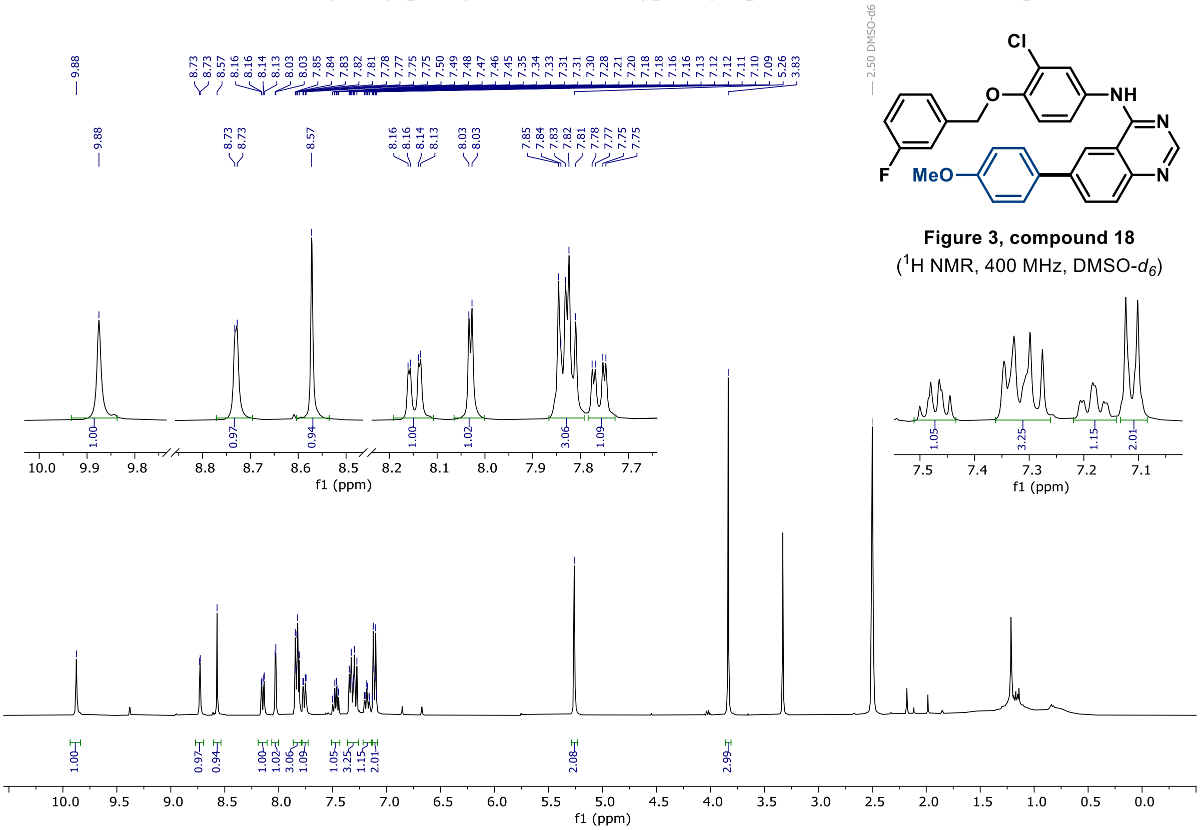

Figure 3, compound 18
(^{1}H NMR, 400 MHz, DMSO-d_6)

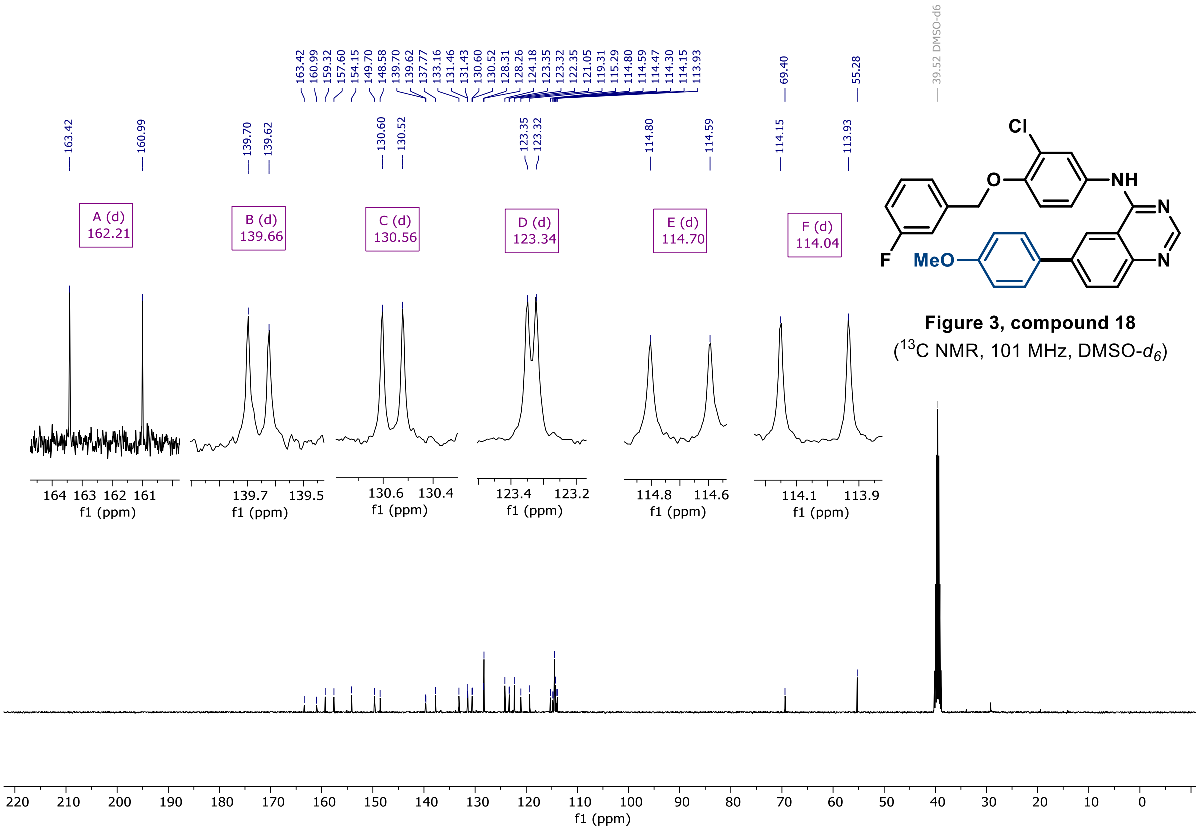

Figure 3, compound 18
(^{13}C NMR, 101 MHz, DMSO-d_6)

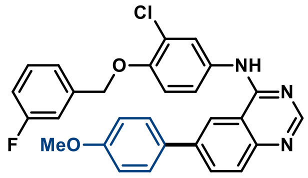

Figure 3, compound 18
(^{19}F NMR, 282 MHz, DMSO-d_6)

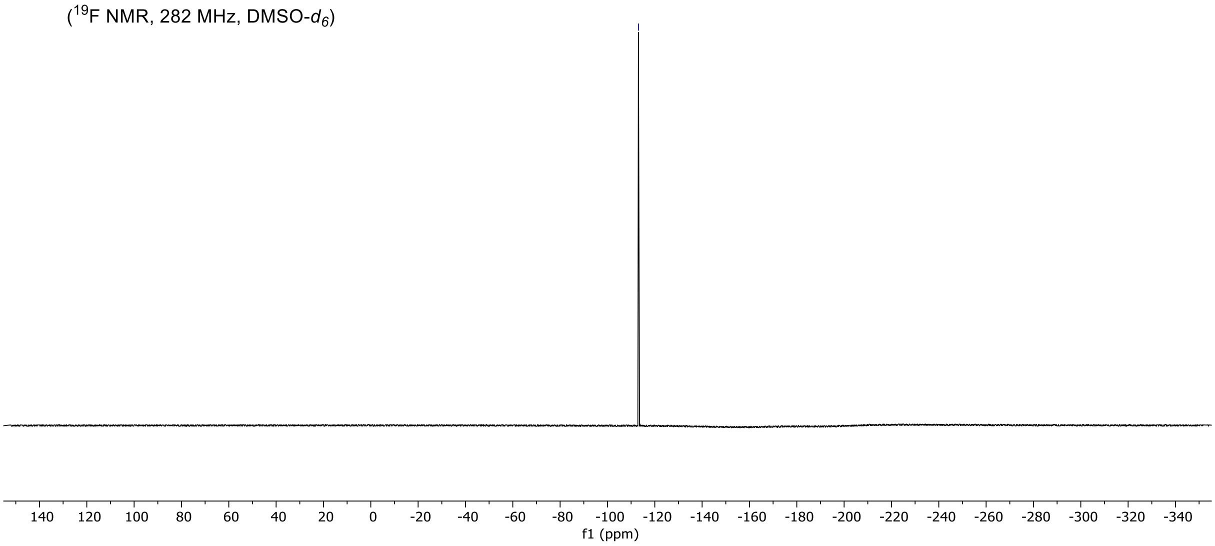

4-Fluoro-1,1'-biphenyl (compound 19)

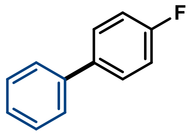

Figure 3, compound 19
(^{1}H NMR, 400 MHz, $CDCl_3$)

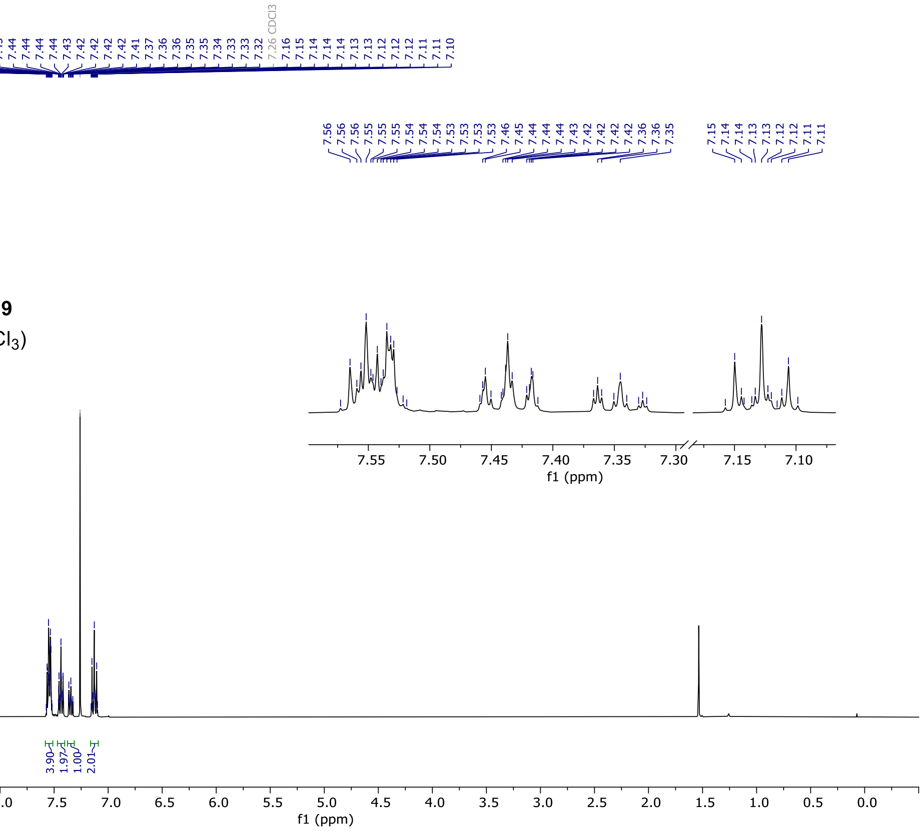

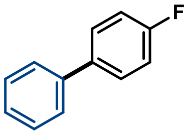

Figure 3, compound 19
(^{13}C NMR, 151 MHz, $CDCl_3$)

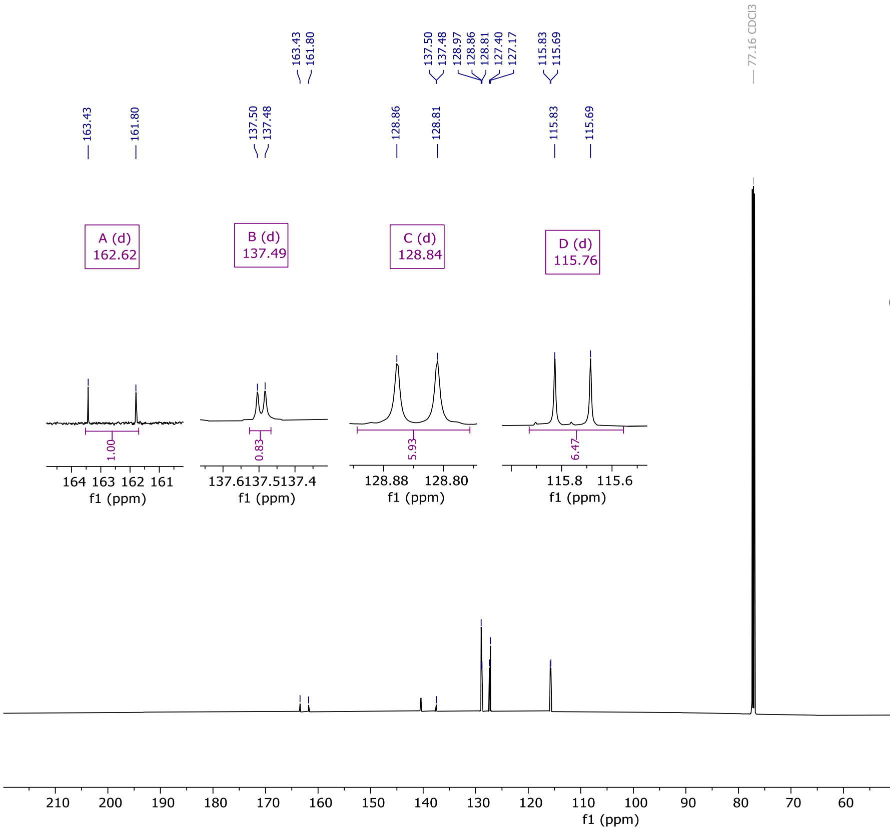

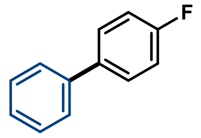

Figure 3, compound 19
(^{19}F NMR, 282 MHz, $CDCl_3$)

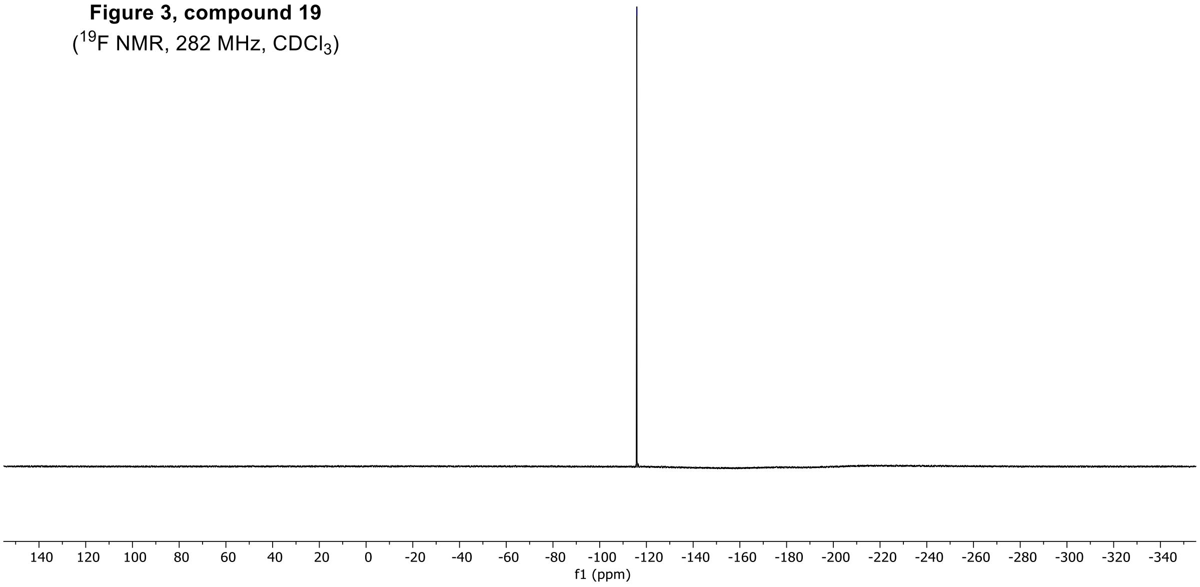

Methyl [1,1'-biphenyl]-4-carboxylate (compound 20)

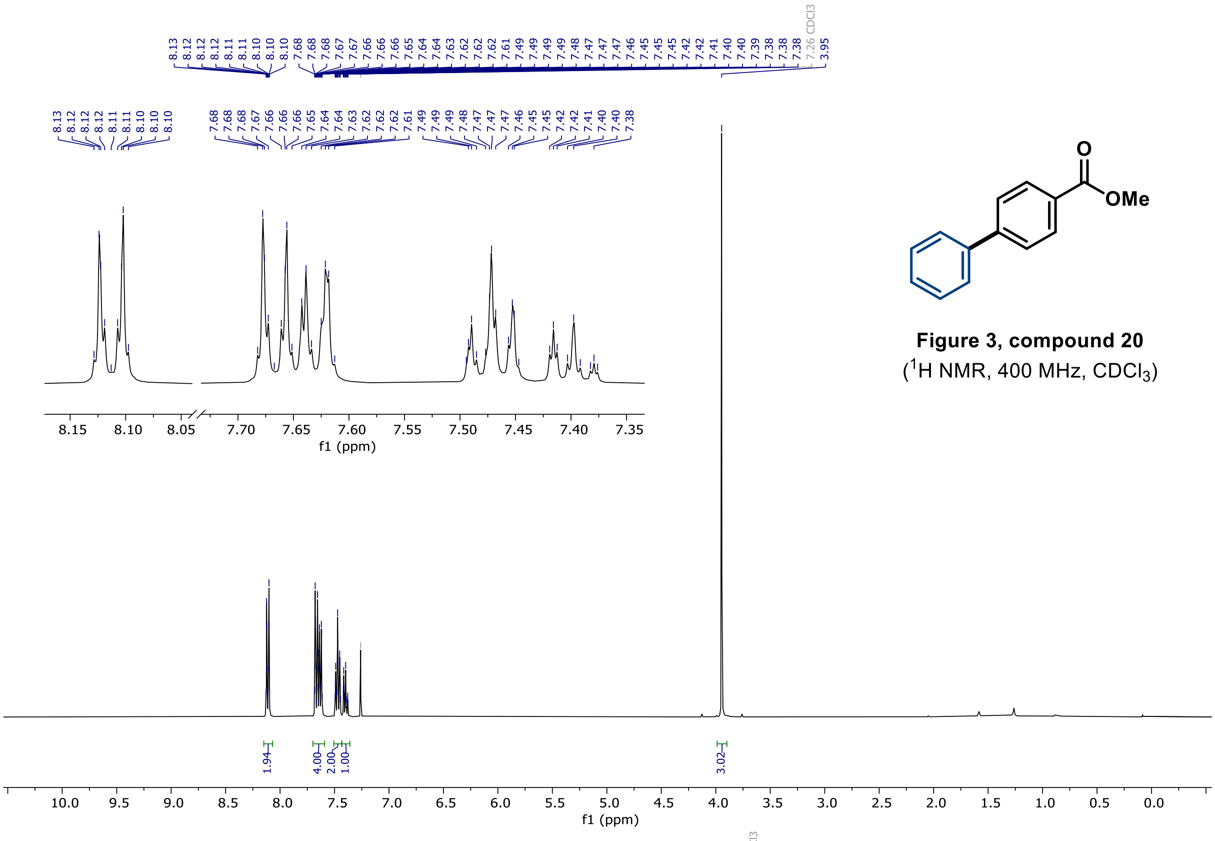

Figure 3, compound 20
(^{1}H NMR, 400 MHz, $CDCl_3$)

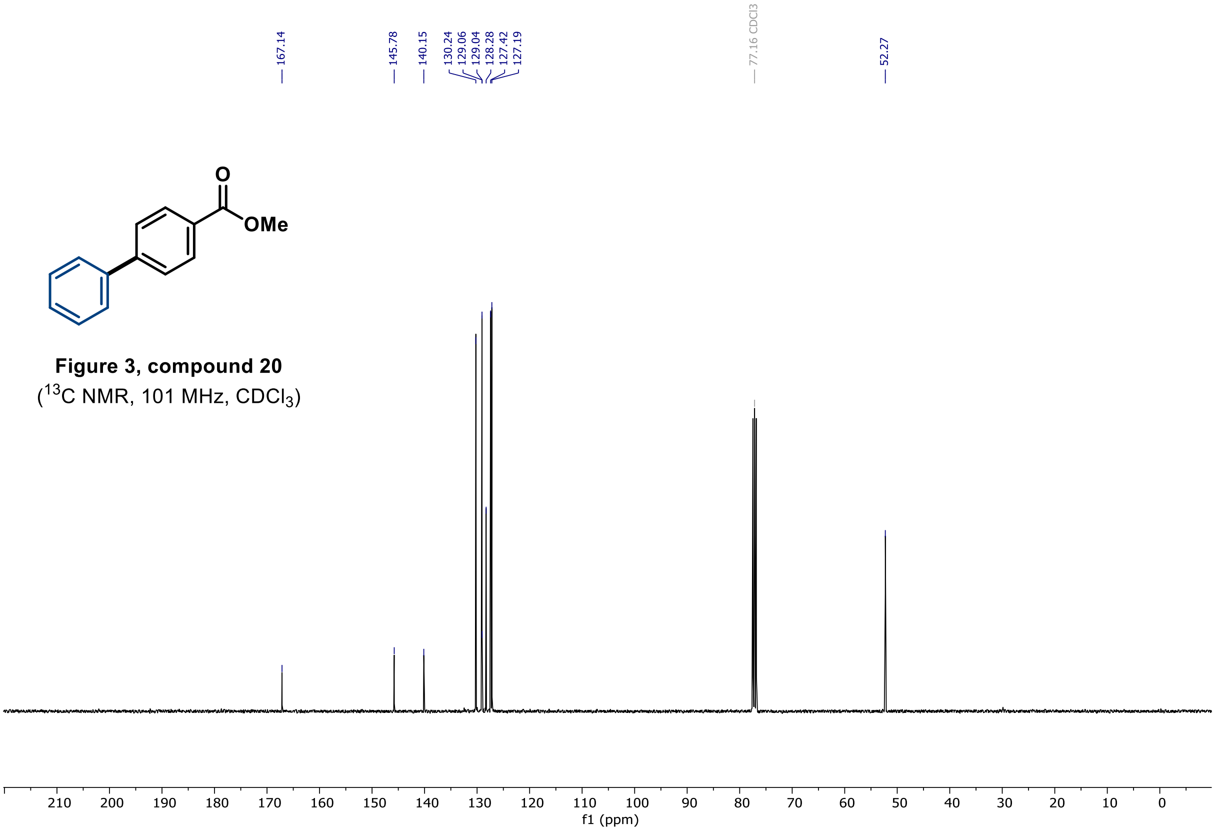

Figure 3, compound 20
(^{13}C NMR, 101 MHz, $CDCl_3$)

3,5-Dimethoxy-1,1'-biphenyl (compound 21)

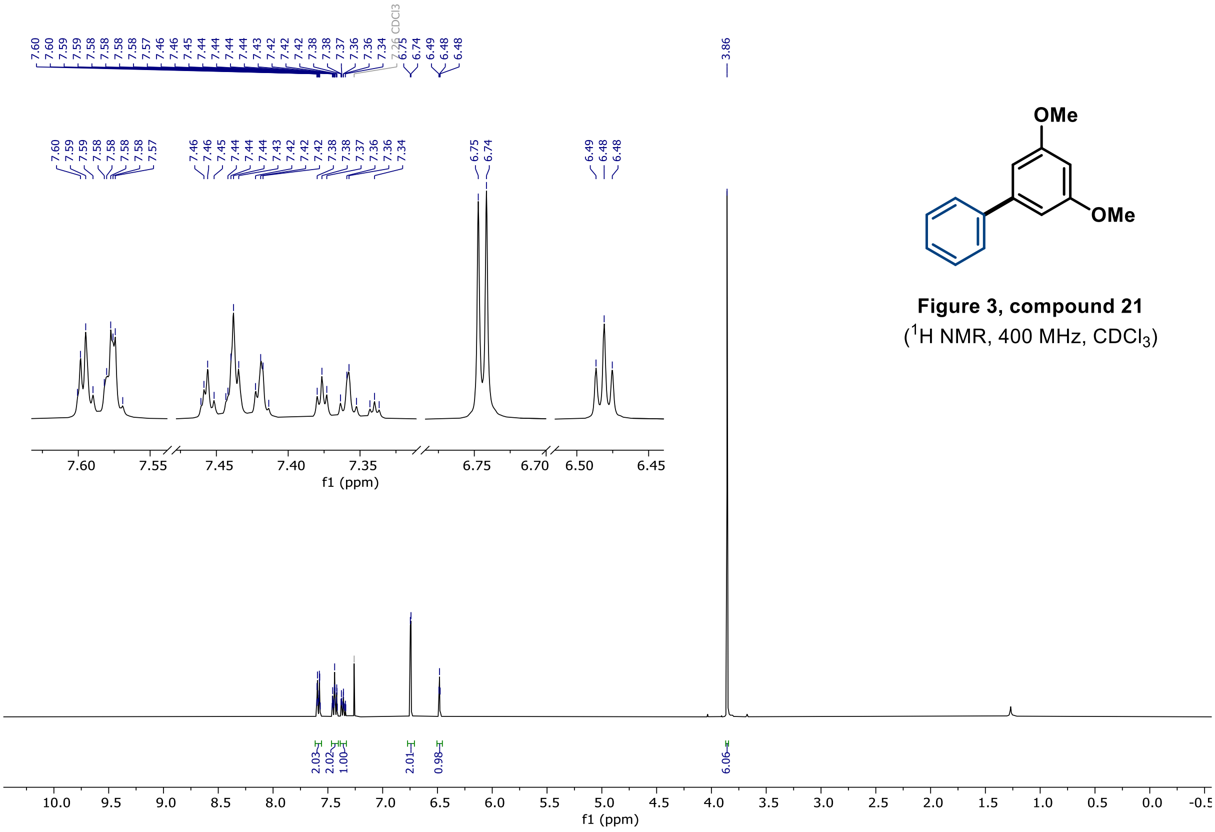

Figure 3, compound 21
(^{1}H NMR, 400 MHz, $CDCl_3$)

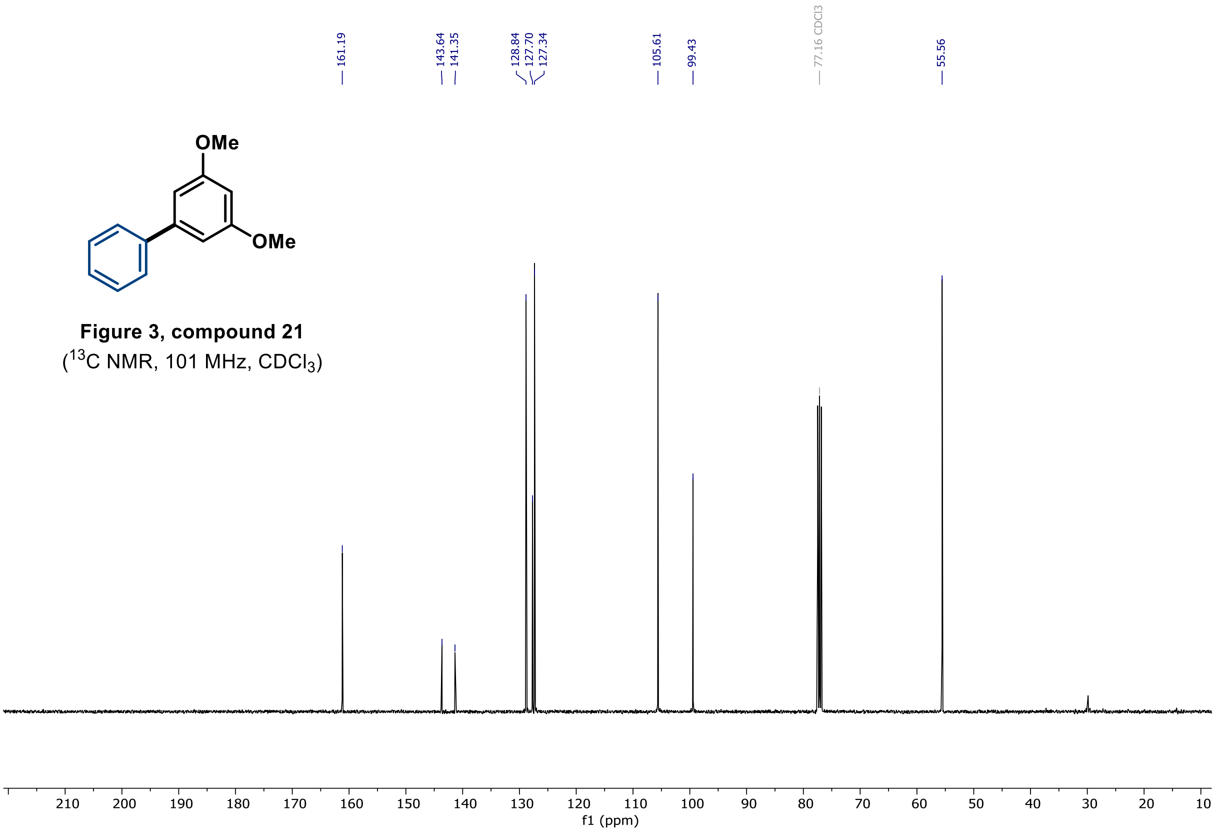

Figure 3, compound 21
(^{13}C NMR, 101 MHz, $CDCl_3$)

4-(Trifluoromethoxy)-1,1'-biphenyl (compound 22)

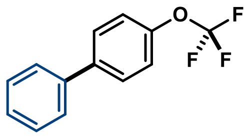

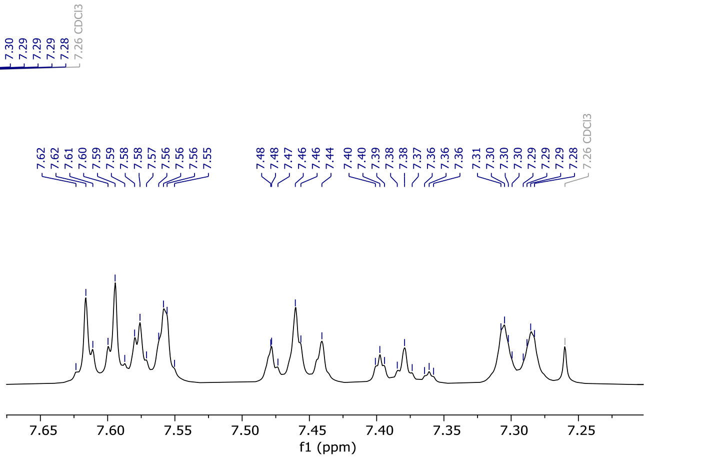

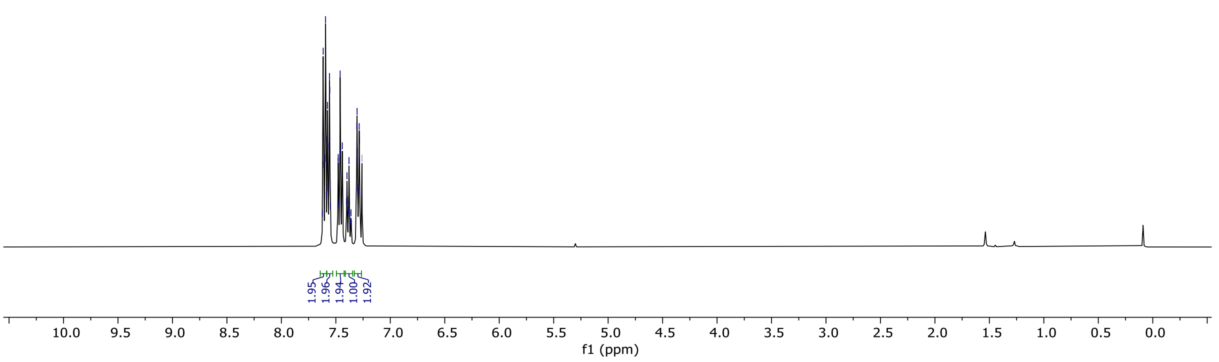

Figure 3, compound 22
(^1H NMR, 400 MHz, $CDCl_3$)

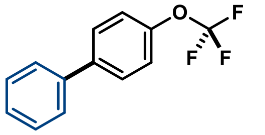

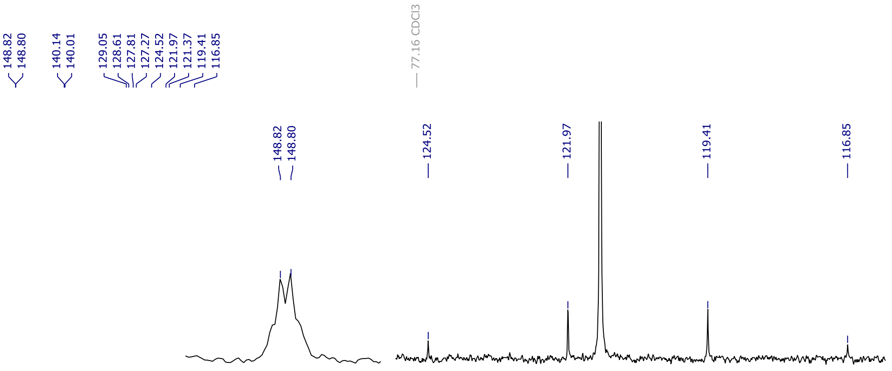

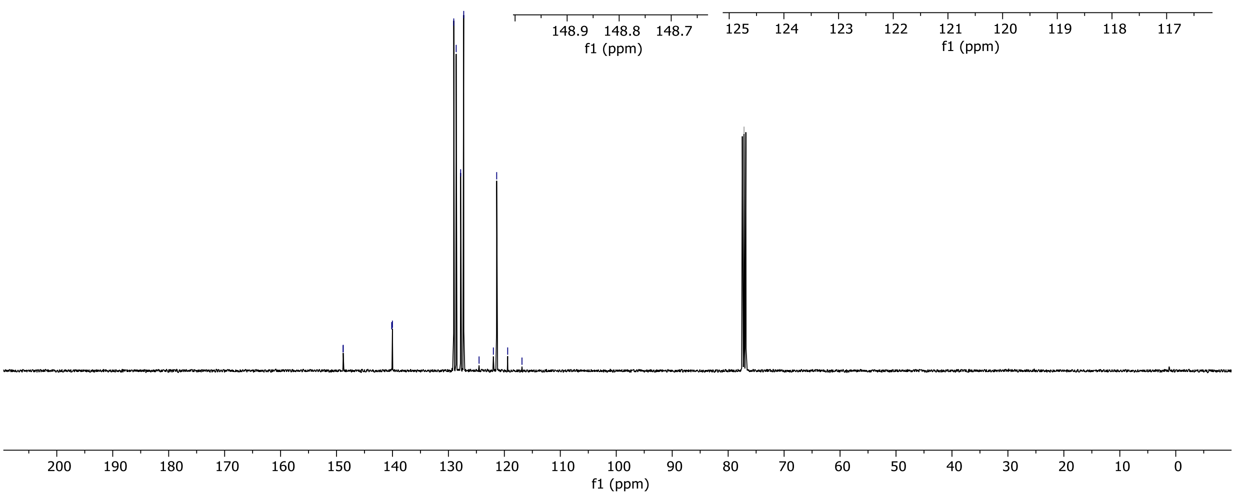

Figure 3, compound 22
(^{13}C NMR, 101 MHz, $CDCl_3$)

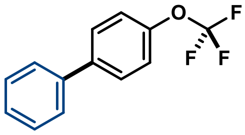

Figure 3, compound 22
(^{19}F NMR, 282 MHz, $CDCl_3$)

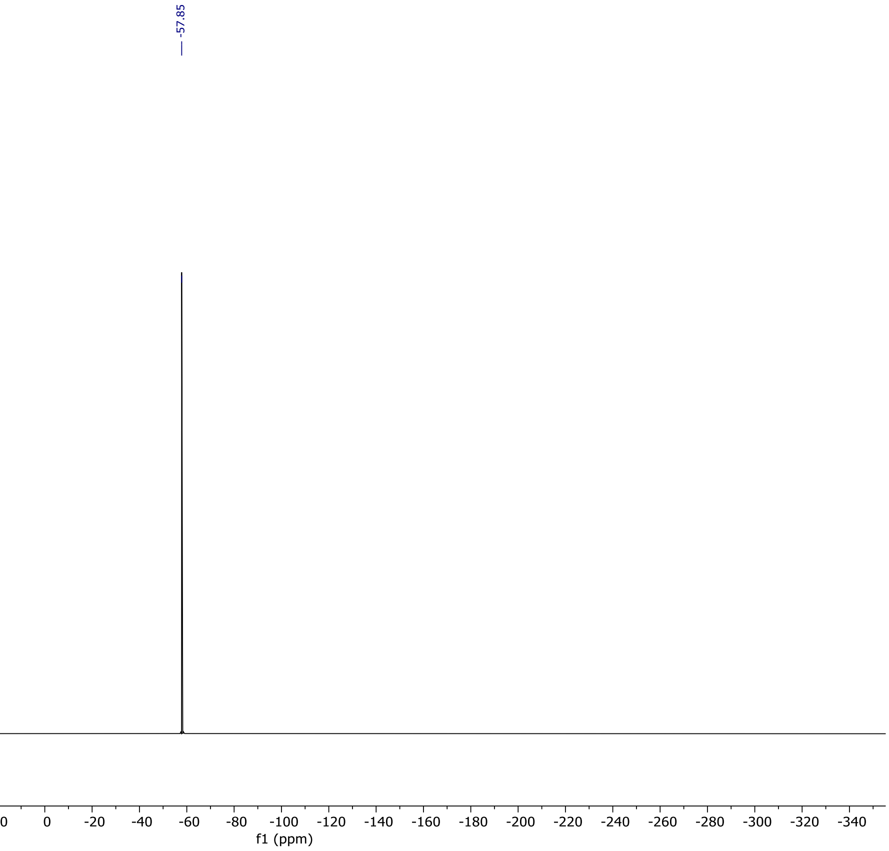

4-Bromo-1,1'-biphenyl (compound 23)

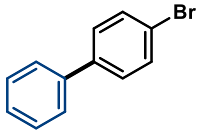

Figure 3, compound 23
(^{1}H NMR, 400 MHz, $CDCl_3$)

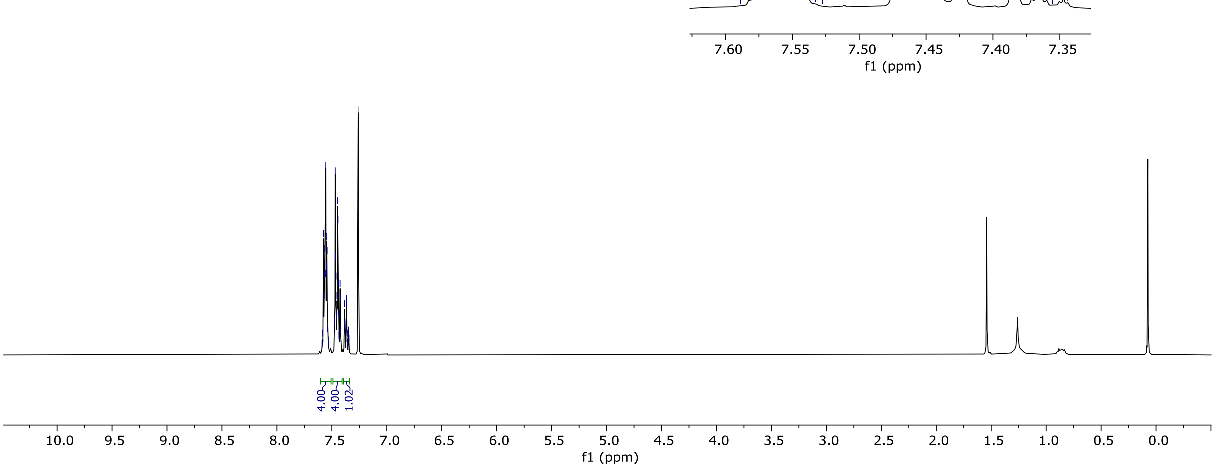

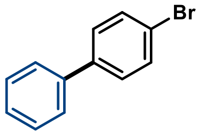

Figure 3, compound 23
(^{13}C NMR, 101 MHz, $CDCl_3$)

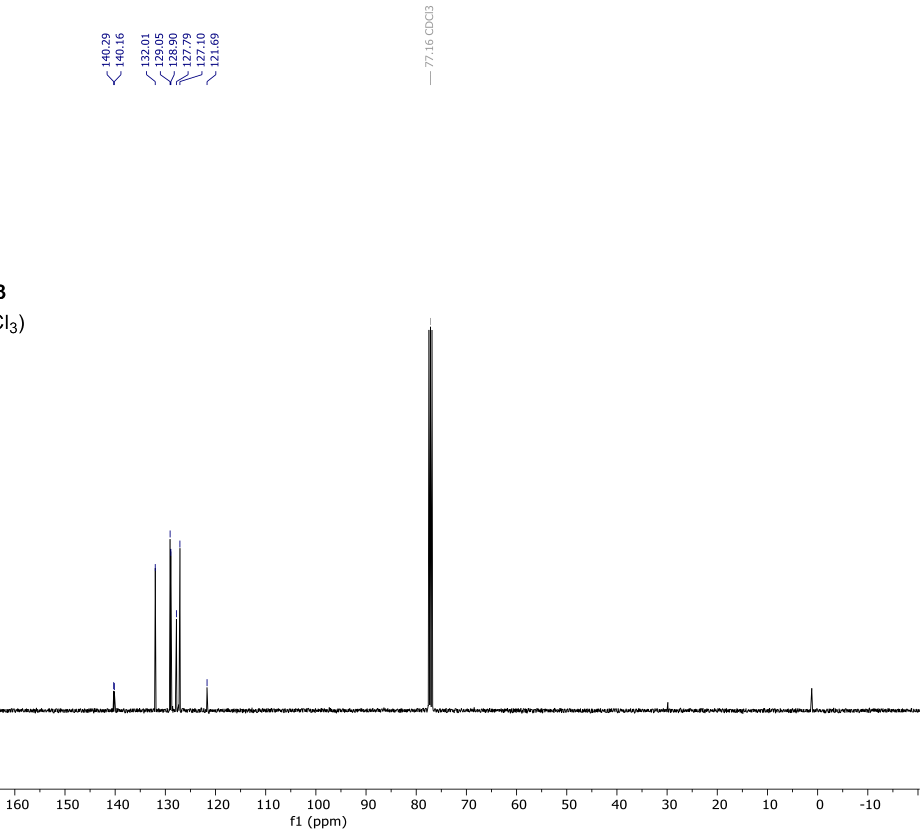

2-Phenylnaphthalene (compound 24)

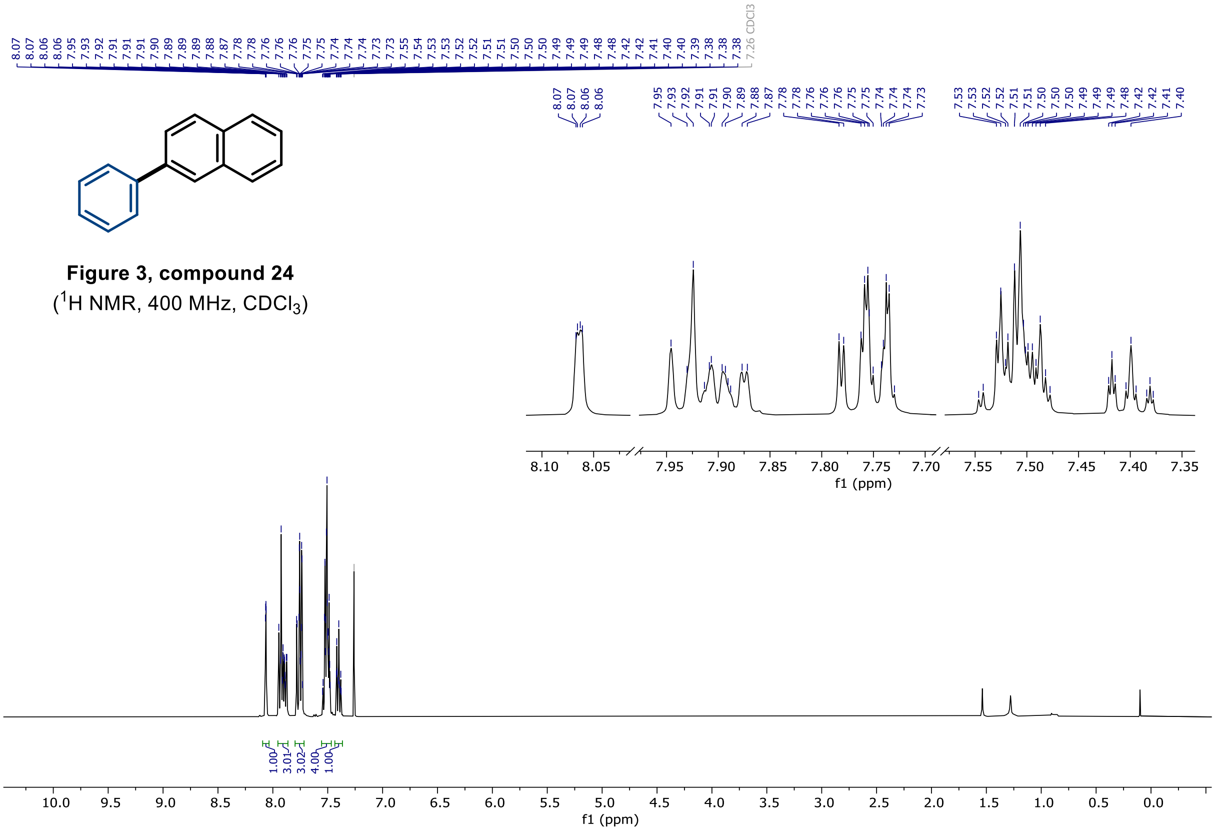

Figure 3, compound 24
(^{1}H NMR, 400 MHz, $CDCl_3$)

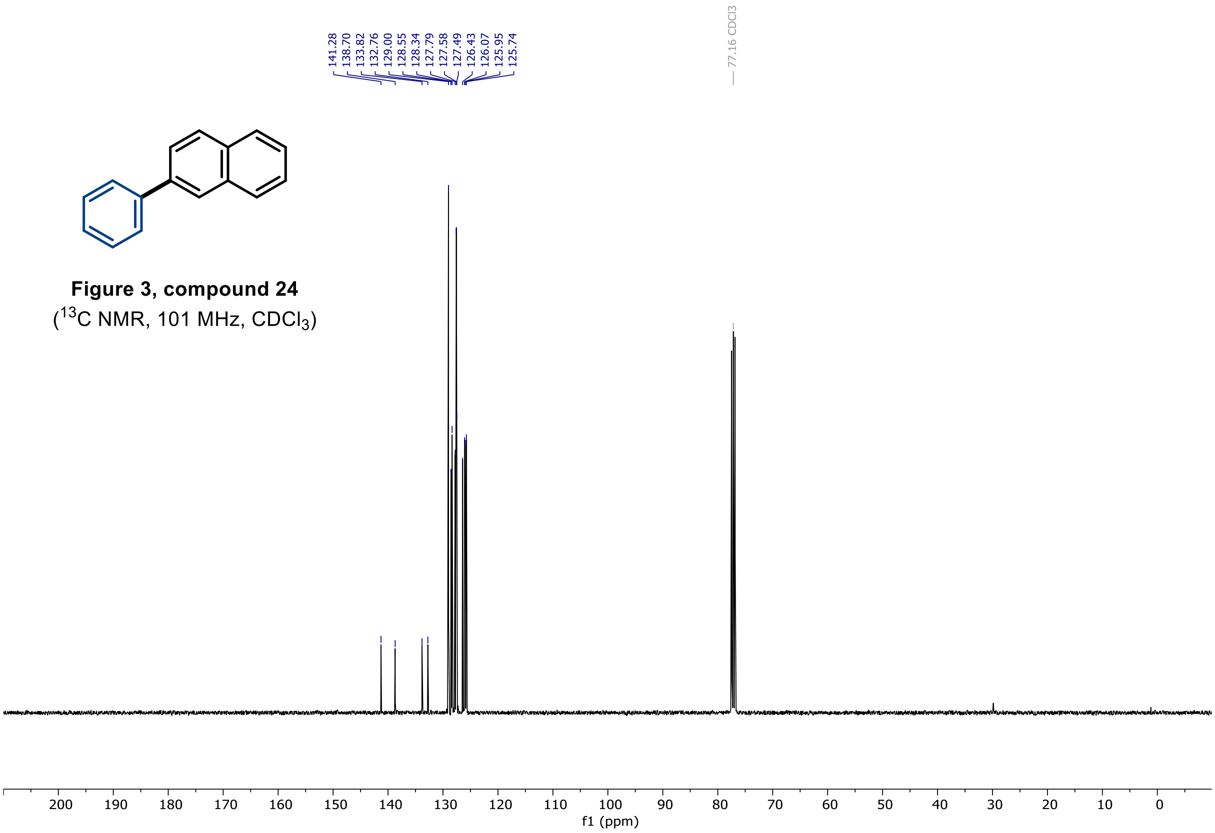

Figure 3, compound 24
(^{13}C NMR, 101 MHz, $CDCl_3$)

4-((4-Methoxyphenyl)ethynyl)-1,1'-biphenyl (compound 25)

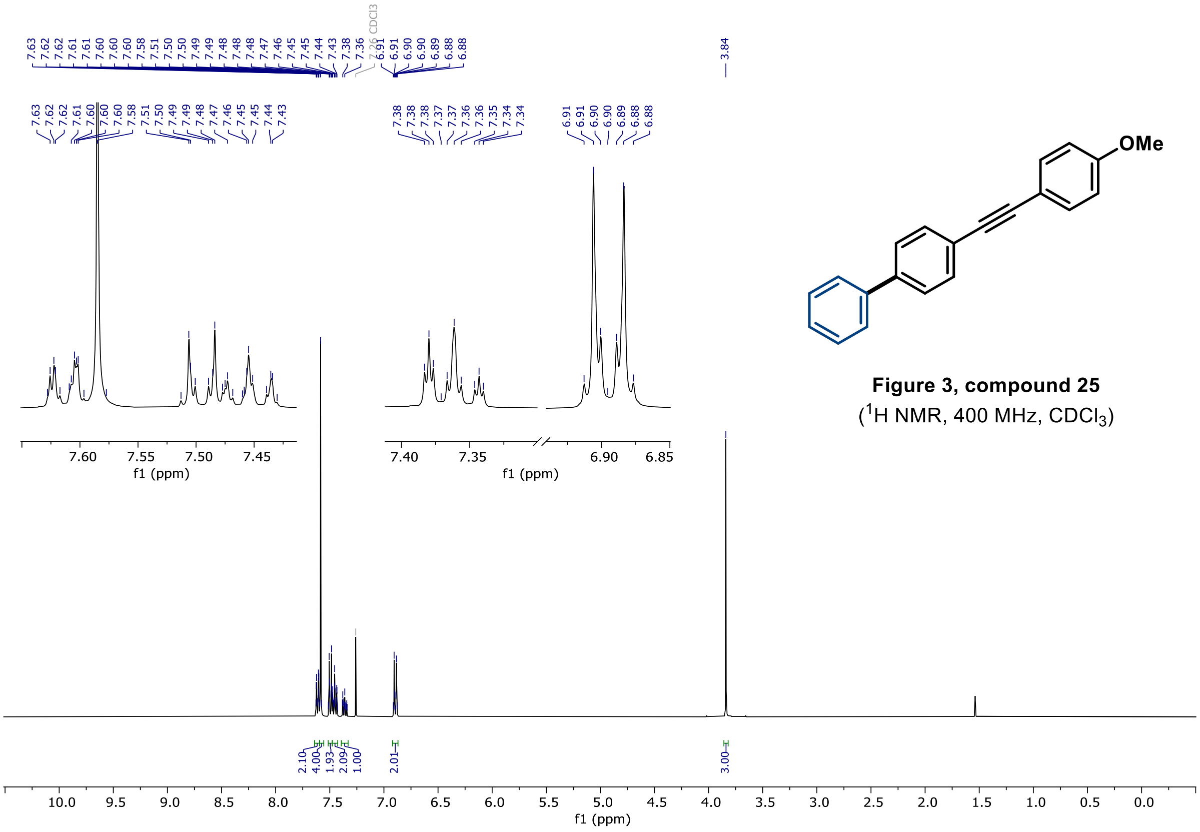

Figure 3, compound 25
(^{1}H NMR, 400 MHz, $CDCl_3$)

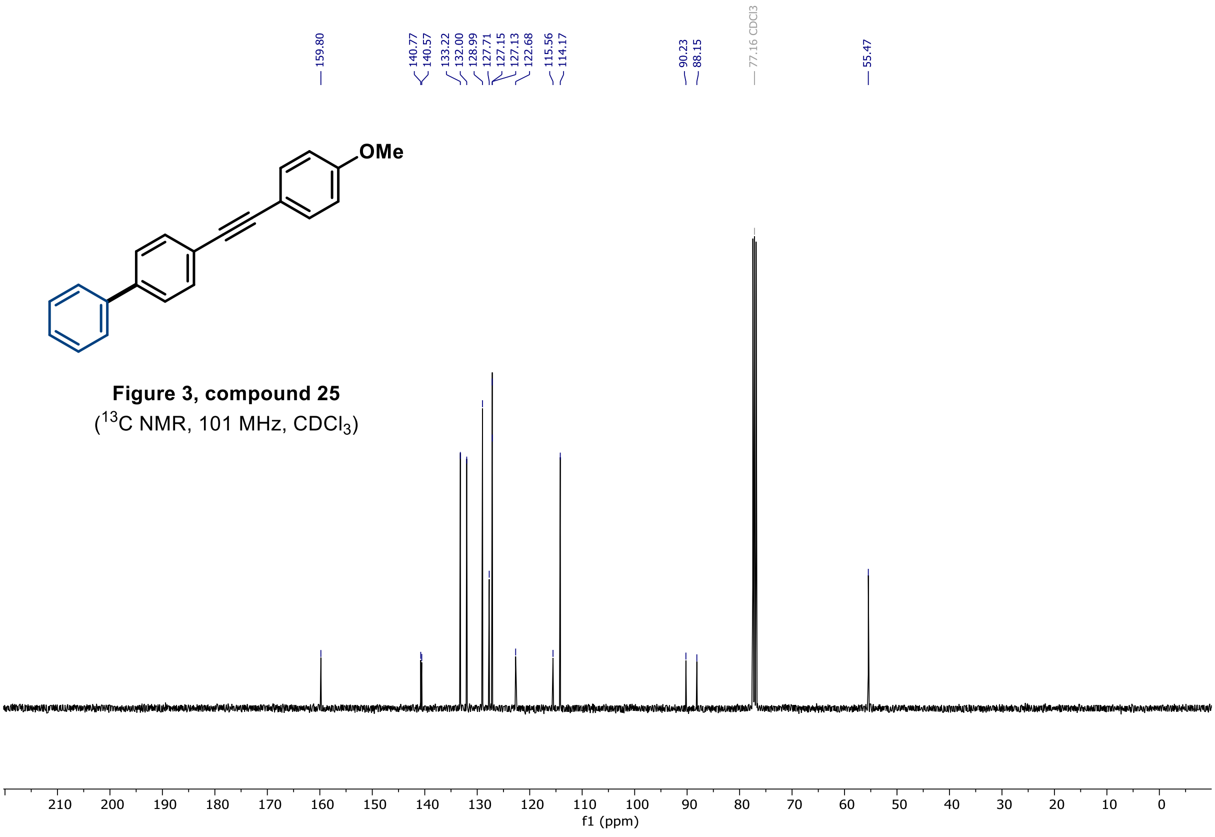

Figure 3, compound 25
(^{13}C NMR, 101 MHz, $CDCl_3$)

1-([1,1'-Biphenyl]-4-yl)-4-methylpiperazine (compound 26)

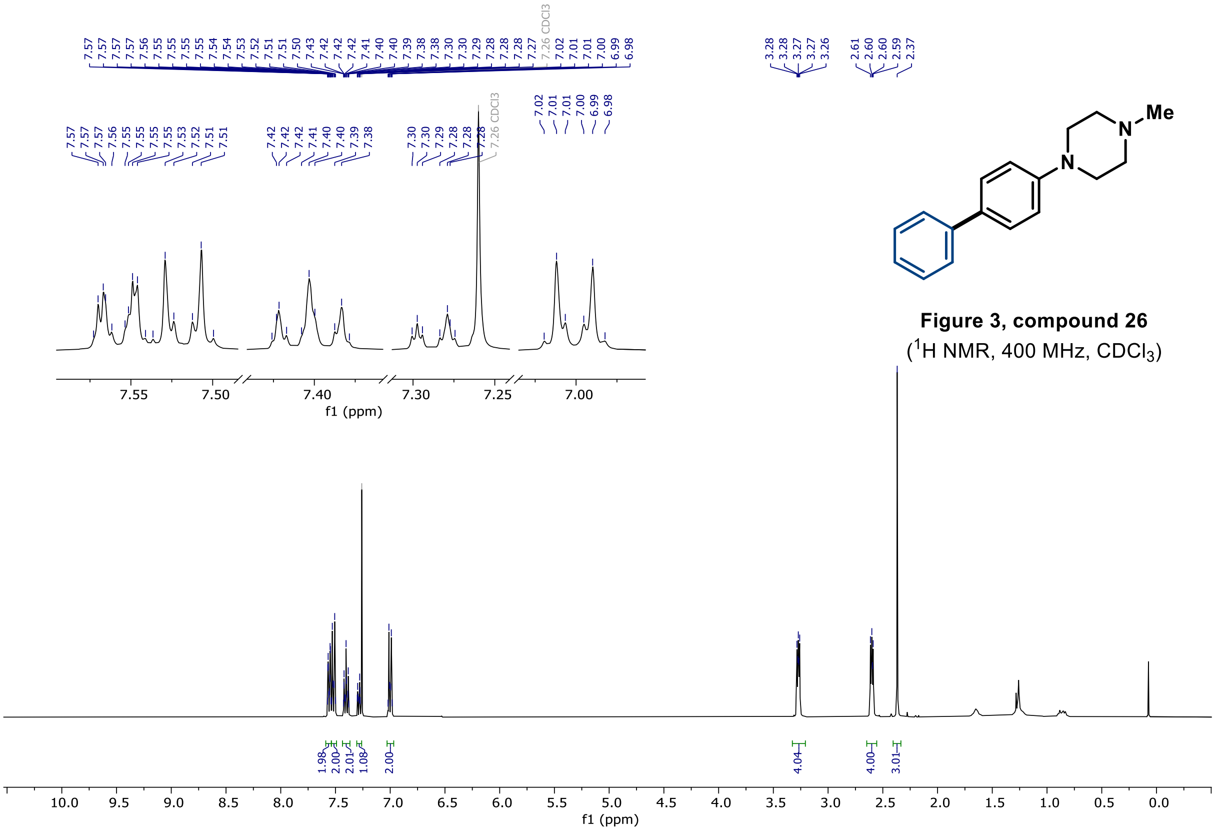

Figure 3, compound 26
(^{1}H NMR, 400 MHz, $CDCl_3$)

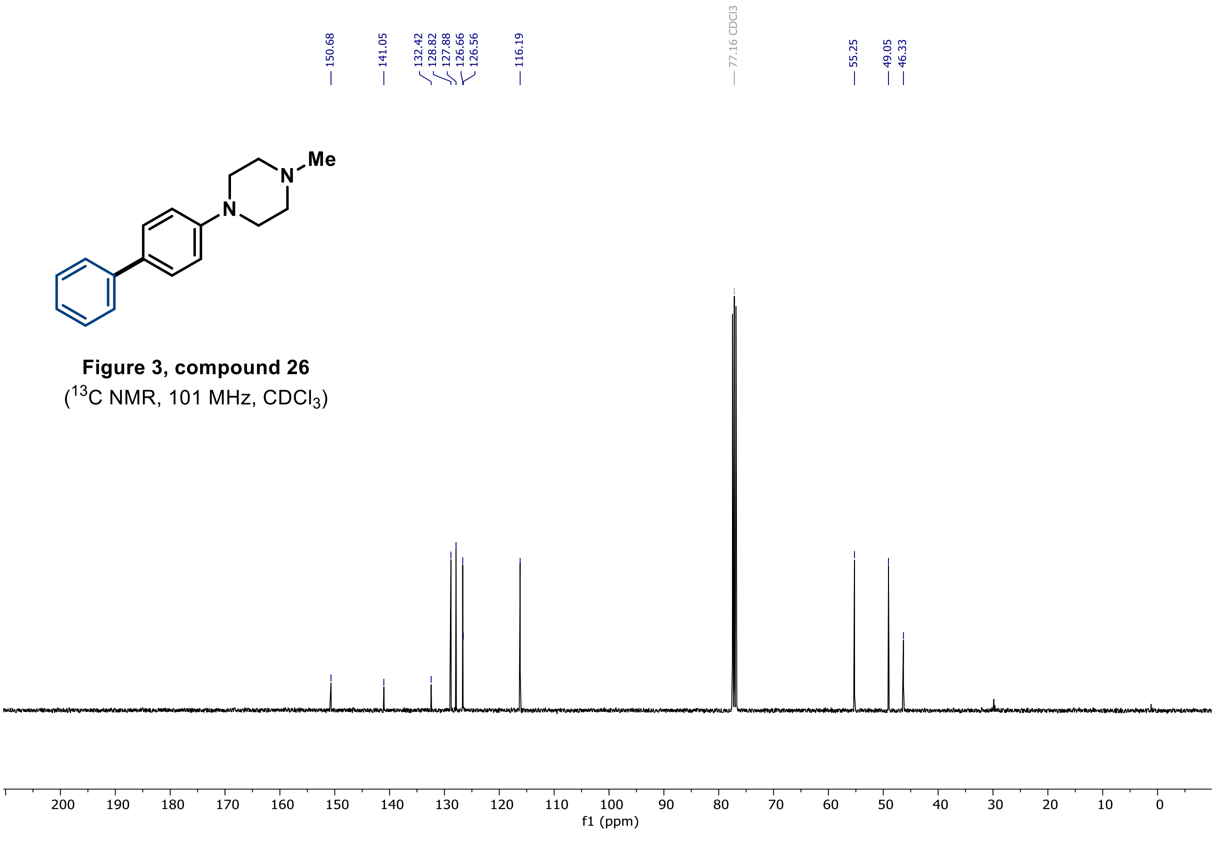

Figure 3, compound 26
(^{13}C NMR, 101 MHz, $CDCl_3$)

2,2-Difluoro-5-phenylbenzo[d][1,3]dioxole (compound 27)

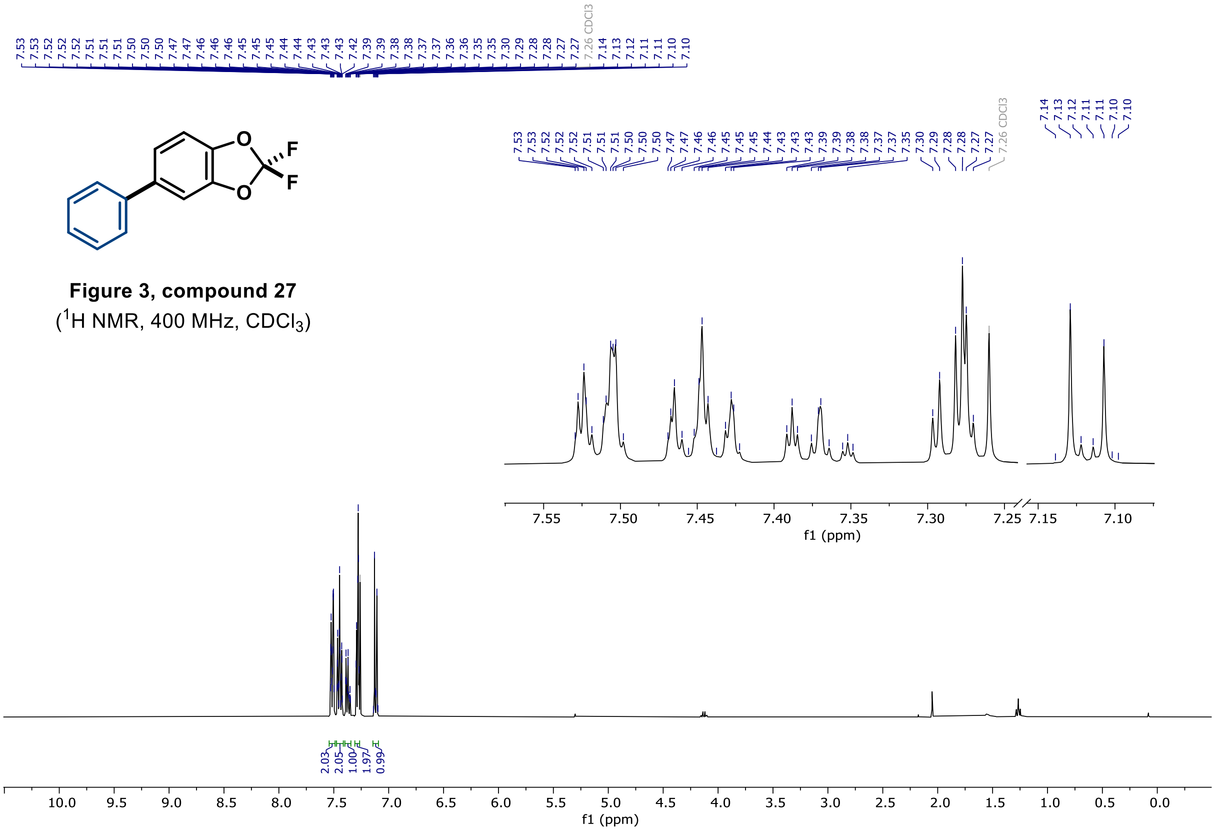

Figure 3, compound 27
(^{1}H NMR, 400 MHz, $CDCl_3$)

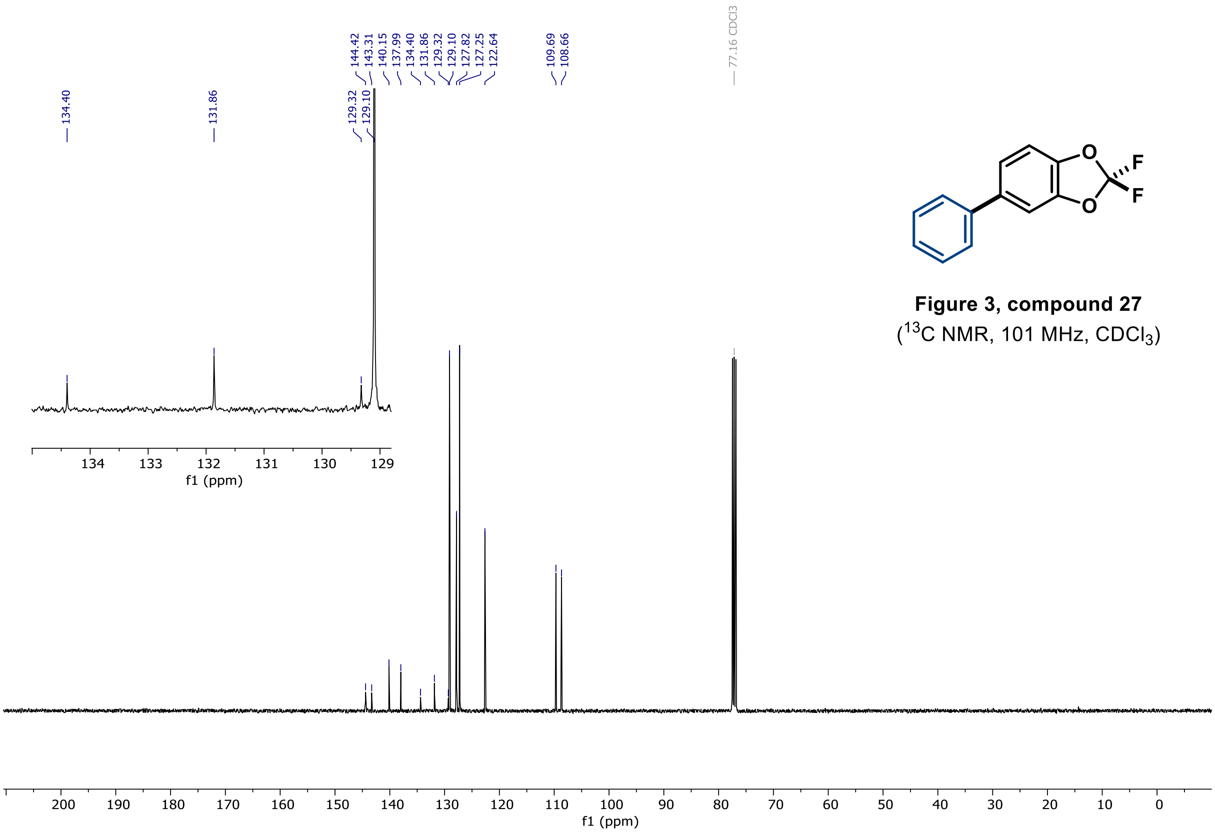

Figure 3, compound 27
(^{13}C NMR, 101 MHz, $CDCl_3$)

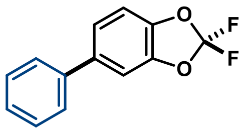

Figure 3, compound 27
(^{19}F NMR, 282 MHz, $CDCl_3$)

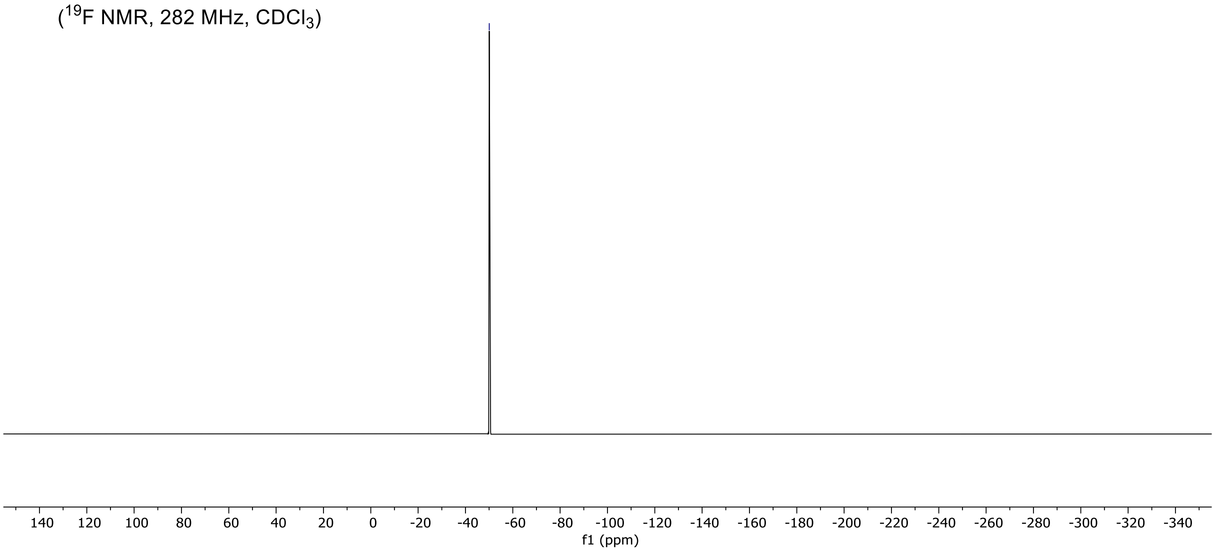

3-Phenylthiophene (compound 28)

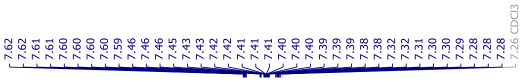

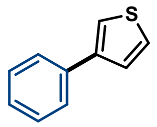

Figure 3, compound 28
(^{1}H NMR, 400 MHz, $CDCl_3$)

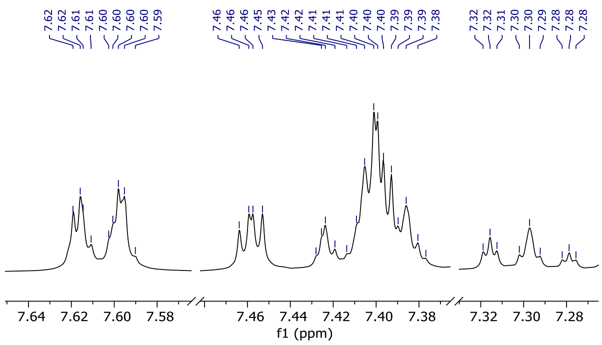

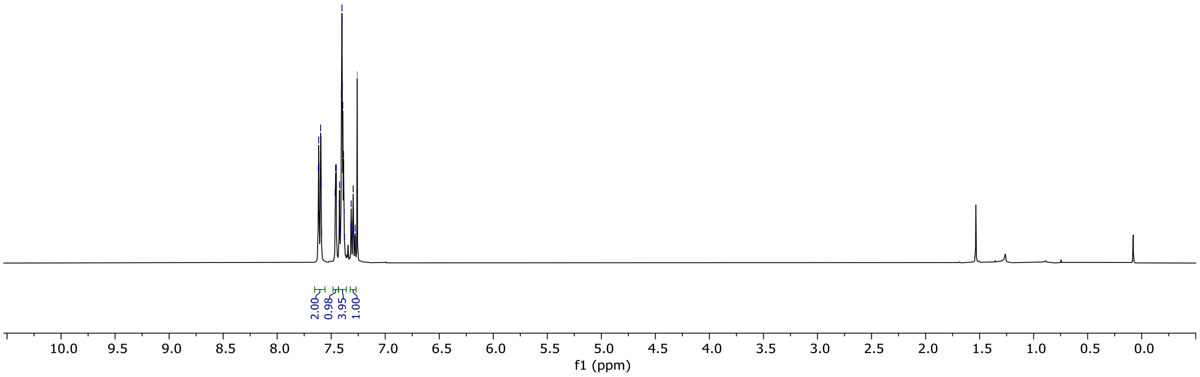

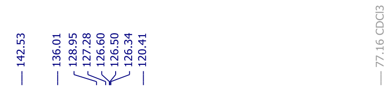

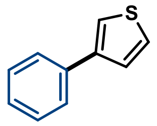

Figure 3, compound 28
(^{13}C NMR, 101 MHz, $CDCl_3$)

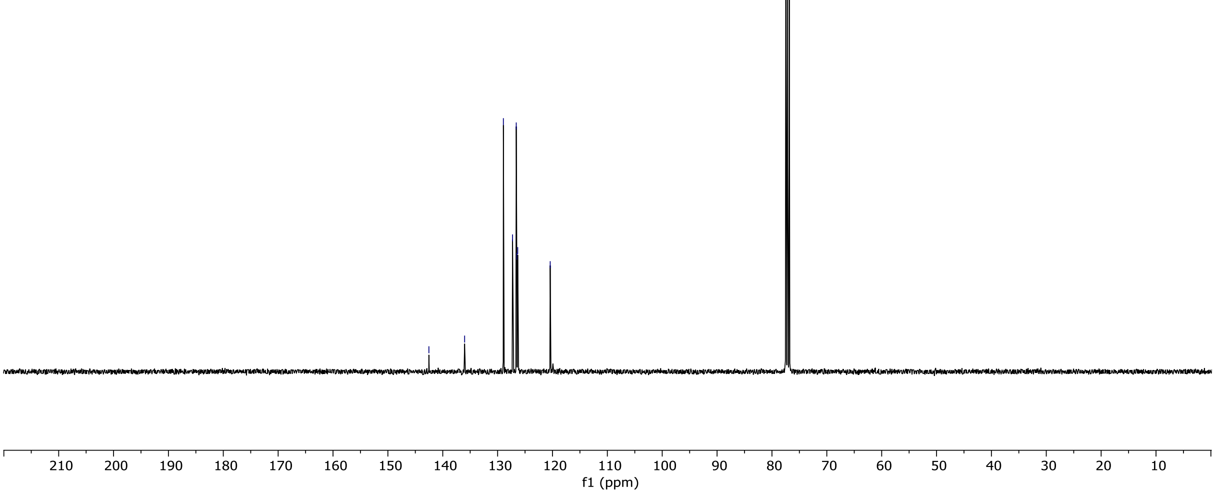

2-Phenylthiophene (compound 29)

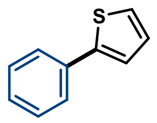

Figure 3, compound 29
(^{1}H NMR, 400 MHz, $CDCl_3$)

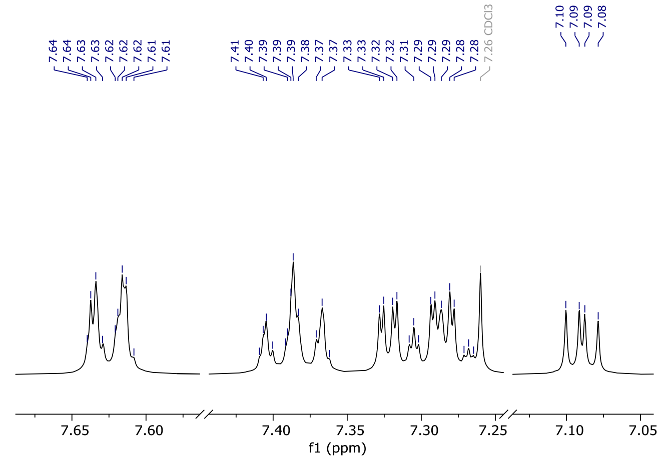

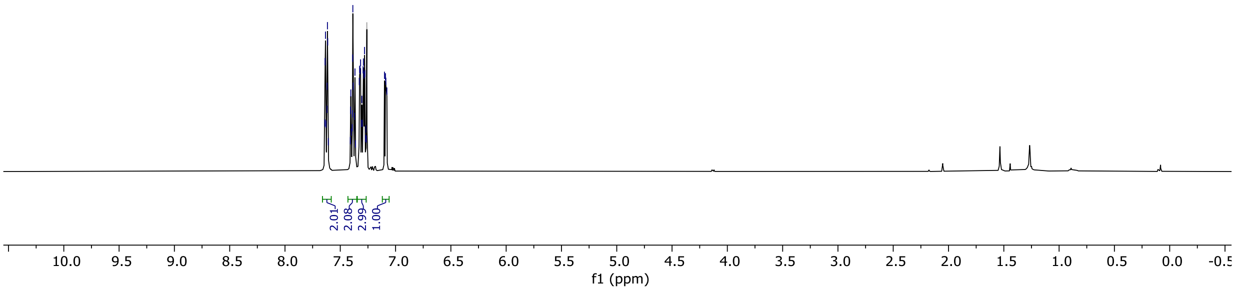

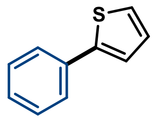

Figure 3, compound 29
(^{13}C NMR, 101 MHz, $CDCl_3$)

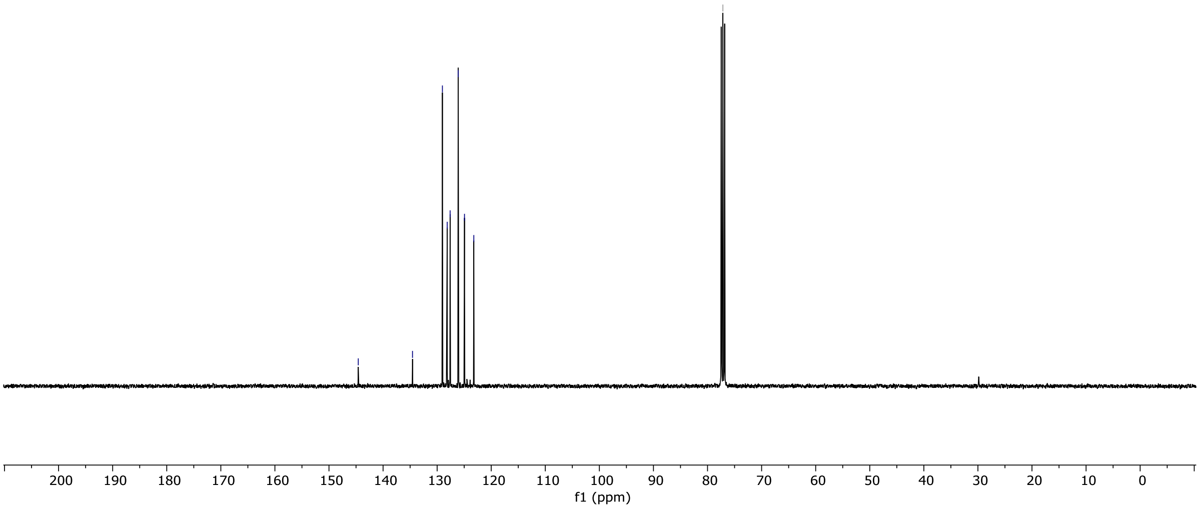

3-Phenylbenzo[b]thiophene (compound 30)

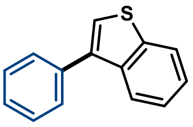

Figure 3, compound 30
(^{1}H NMR, 400 MHz, $CDCl_3$)

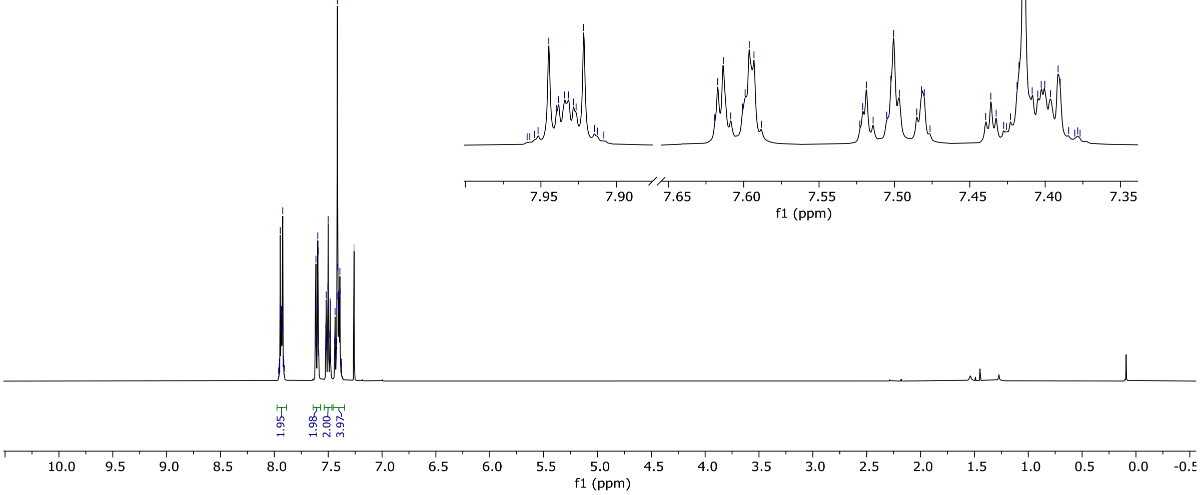

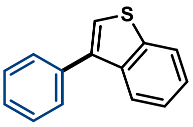

Figure 3, compound 30
(^{13}C NMR, 101 MHz, $CDCl_3$)

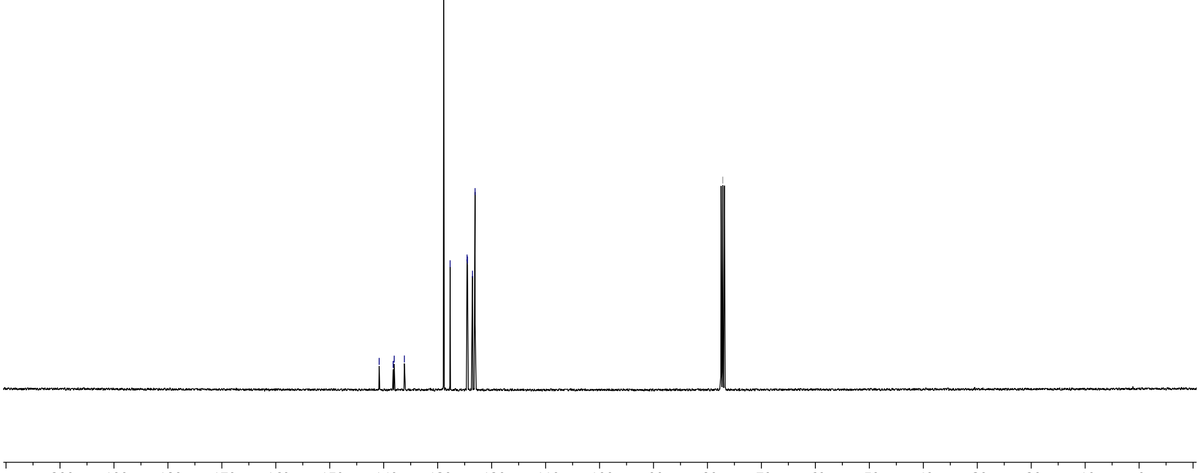

6-Phenylbenzo[d]thiazole (compound 31)

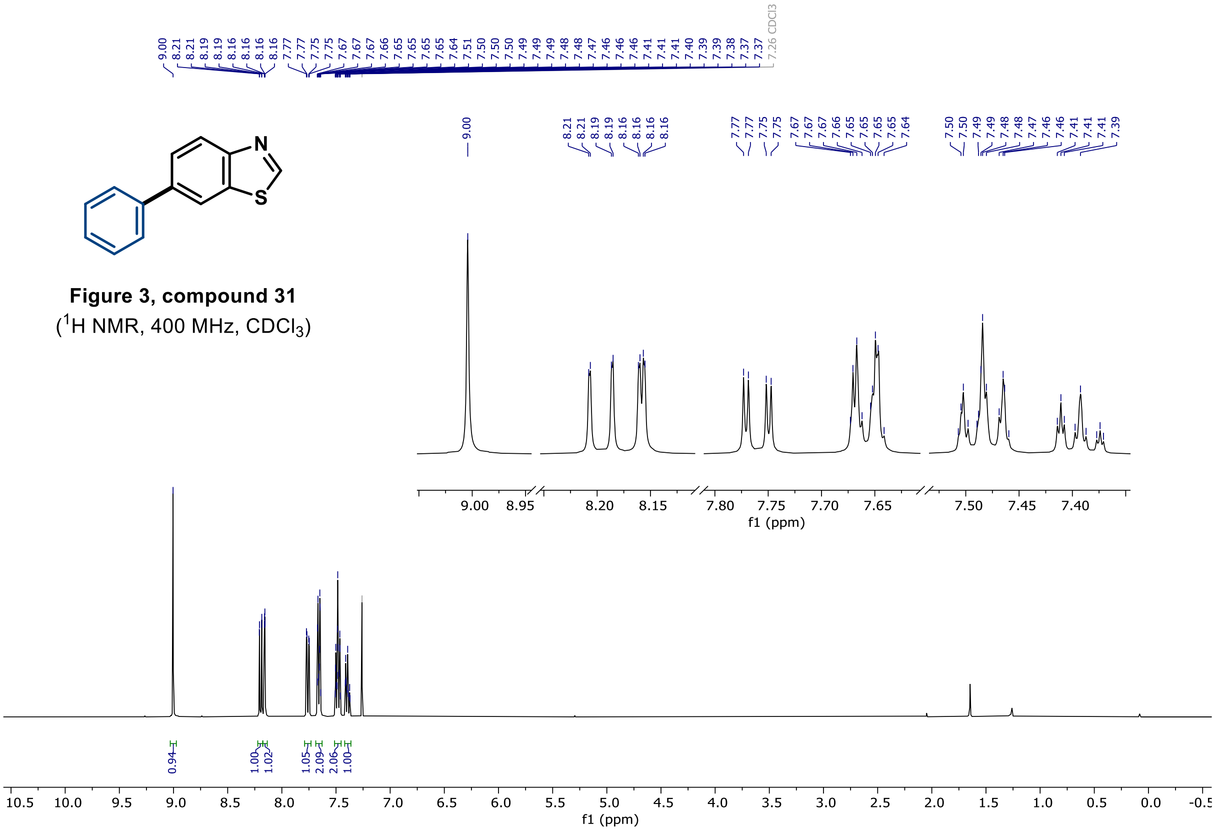

Figure 3, compound 31
(^{1}H NMR, 400 MHz, $CDCl_3$)

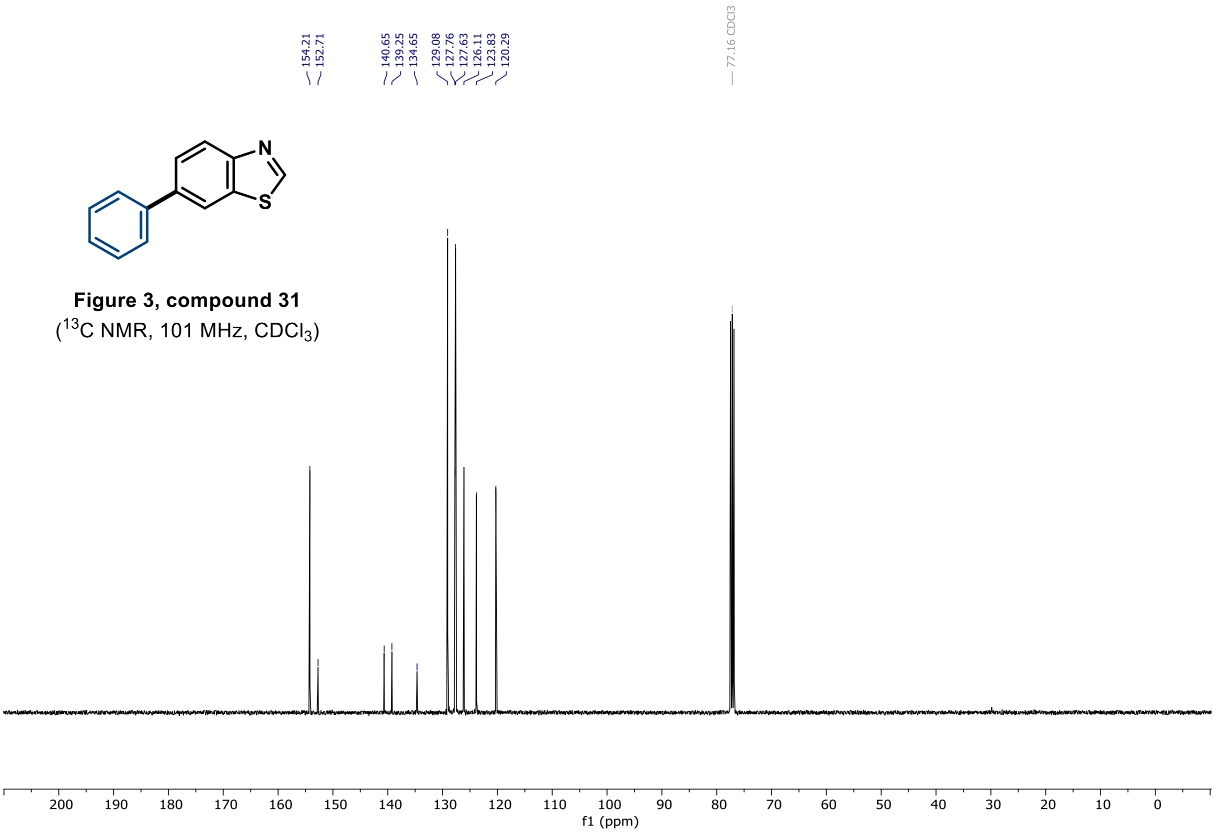

Figure 3, compound 31
(^{13}C NMR, 101 MHz, $CDCl_3$)

6-Phenylquinoline (compound 32)

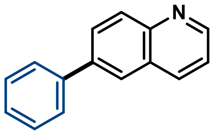

Figure 3, compound 32
(^{1}H NMR, 400 MHz, $CDCl_3$)

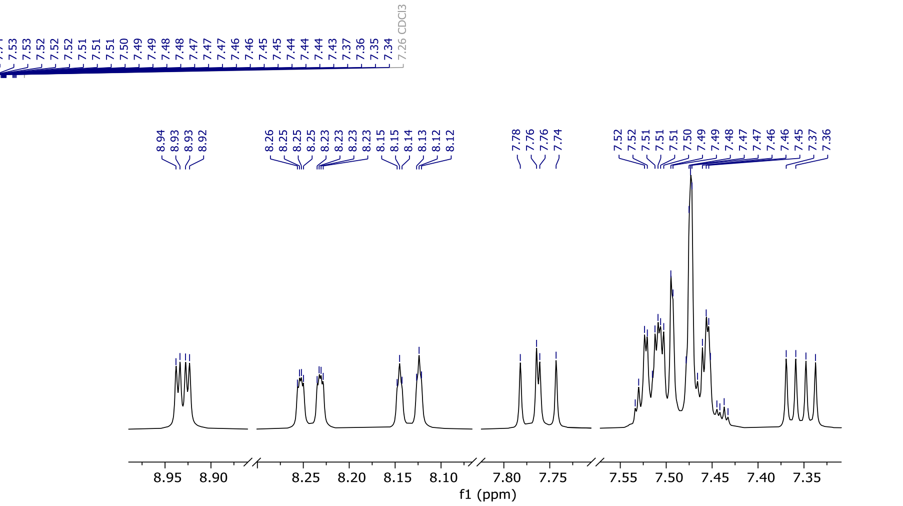

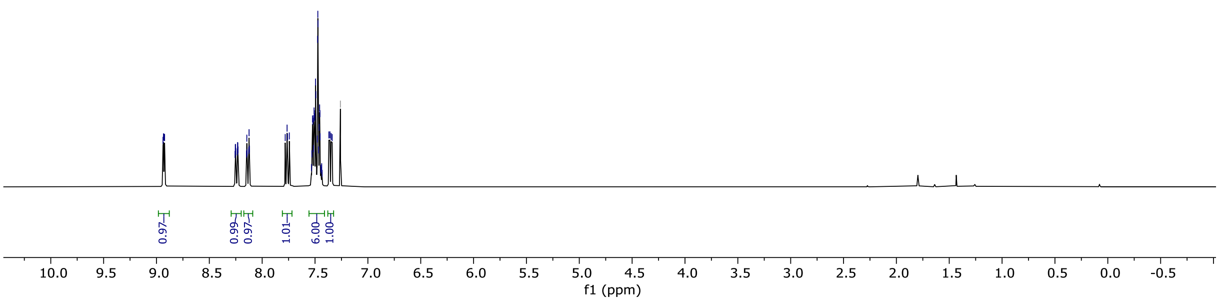

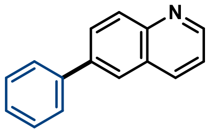

Figure 3, compound 32
(^{13}C NMR, 101 MHz, $CDCl_3$)

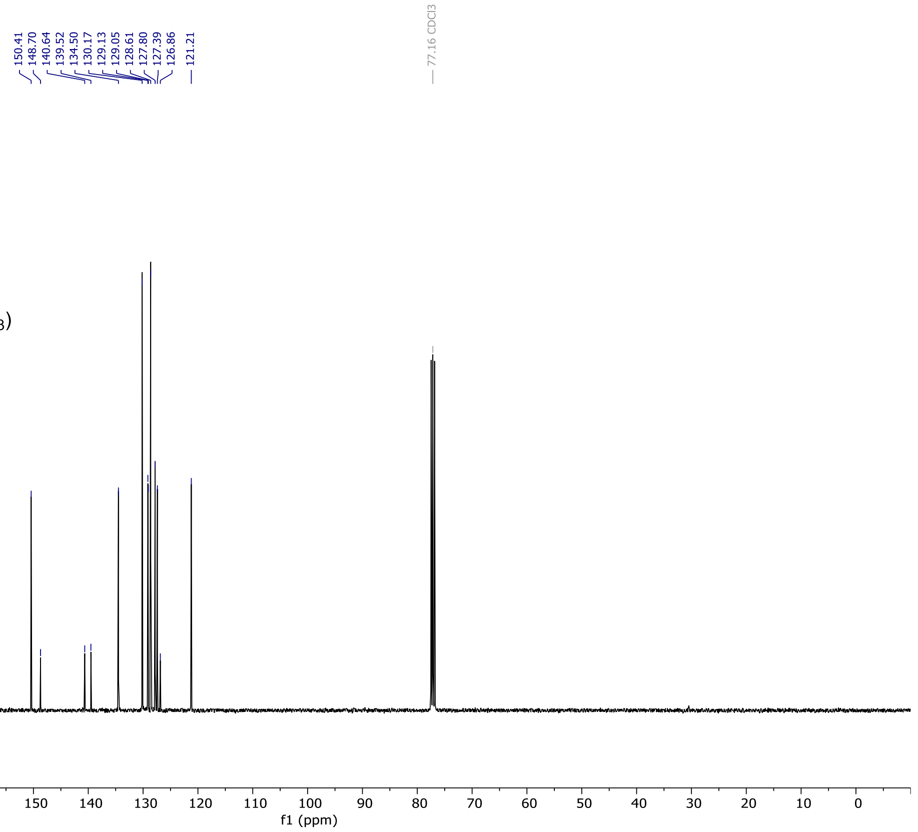

4-([1,1'-Biphenyl]-4-yl)pyridine (compound 33)

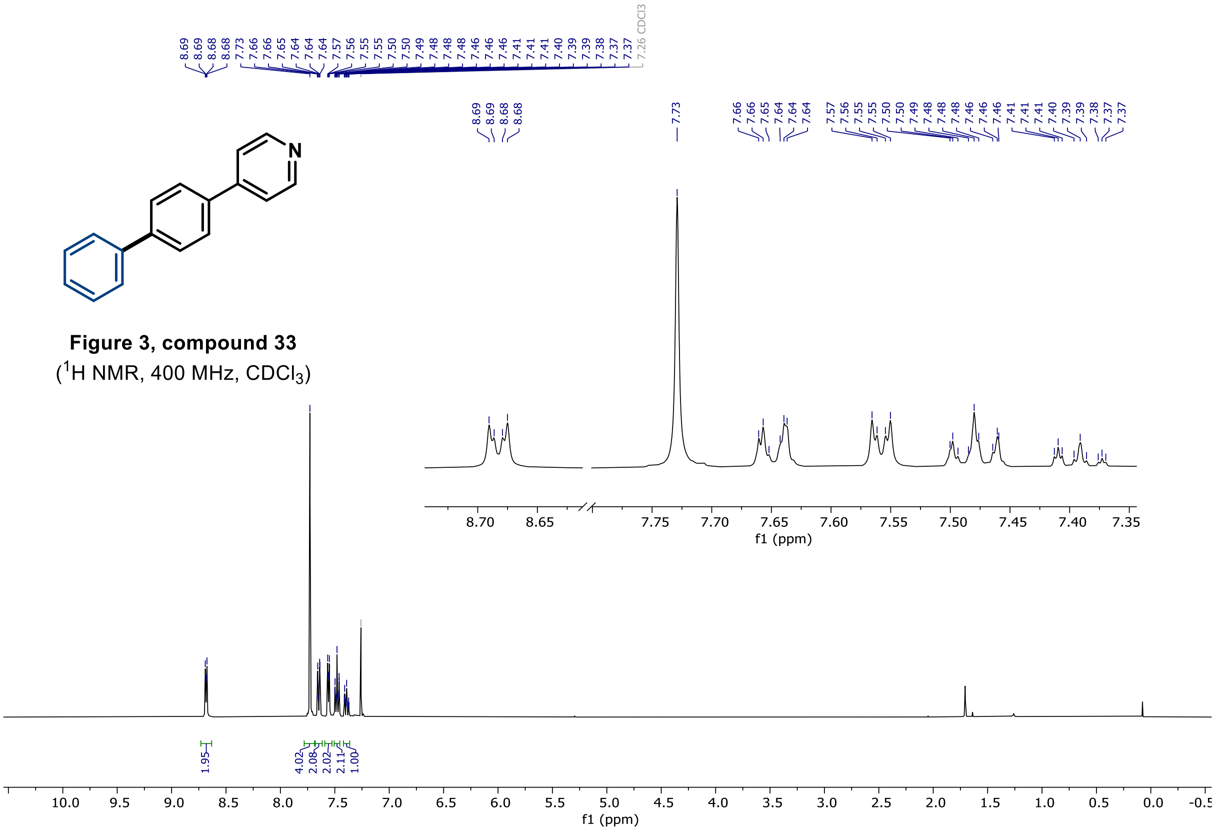

Figure 3, compound 33
(^{1}H NMR, 400 MHz, $CDCl_3$)

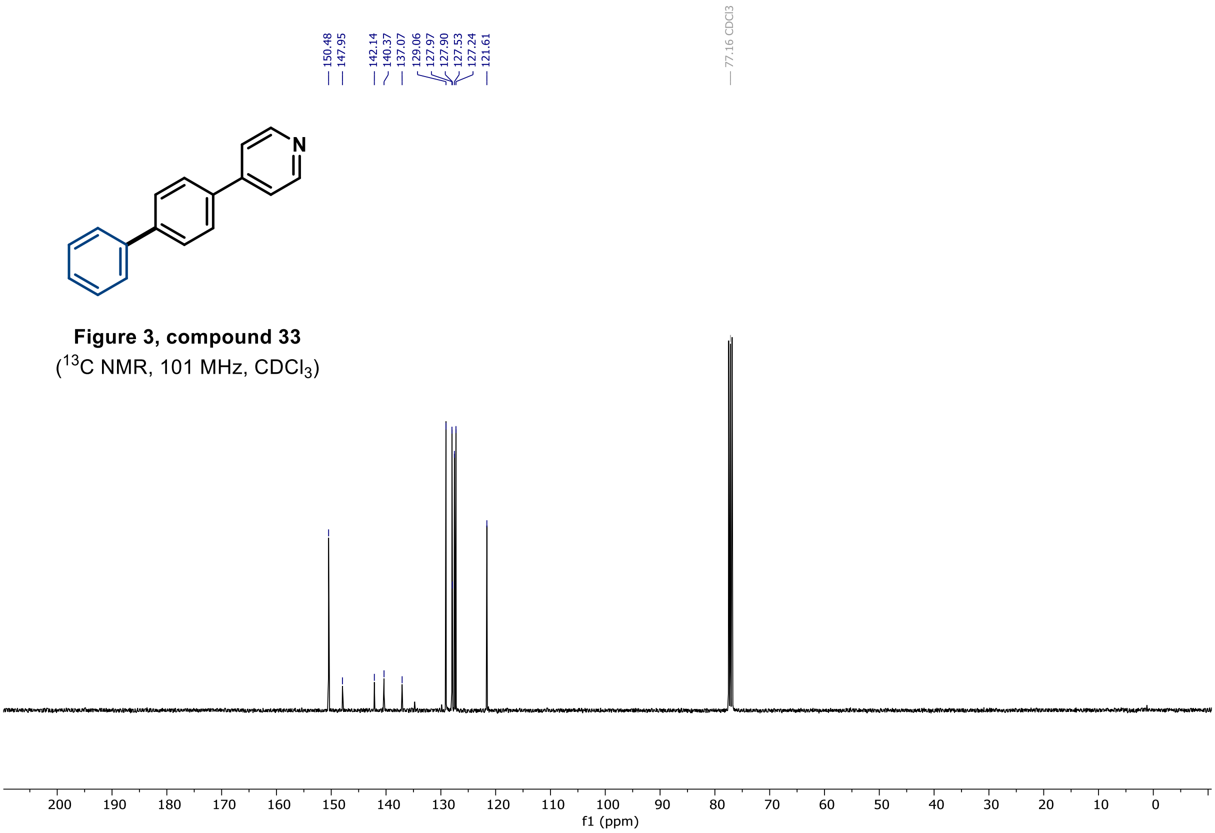

Figure 3, compound 33
(^{13}C NMR, 101 MHz, $CDCl_3$)

4-Phenyl-1H-pyrazole (compound 34)

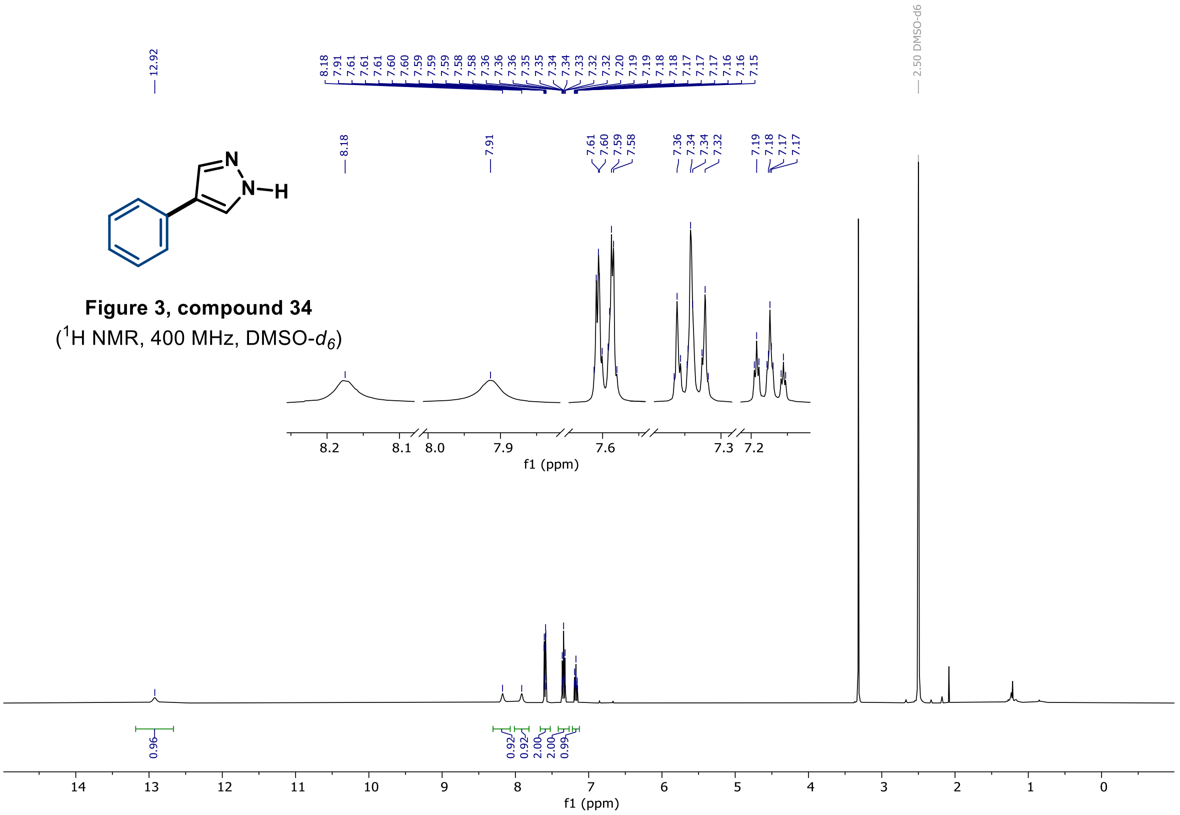

Figure 3, compound 34
(^{1}H NMR, 400 MHz, DMSO-d_6)

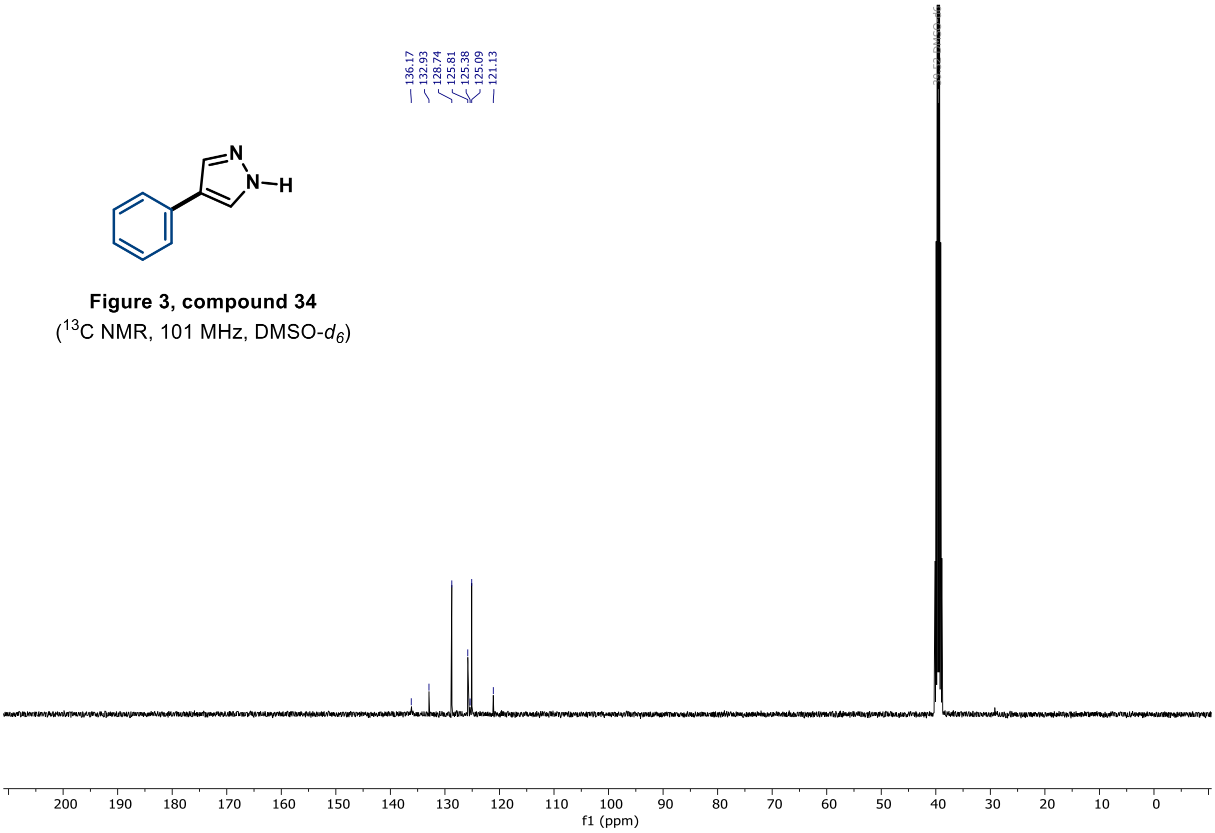

Figure 3, compound 34
(^{13}C NMR, 101 MHz, DMSO-d_6)

5-Phenyl-1H-indole (compound 35)

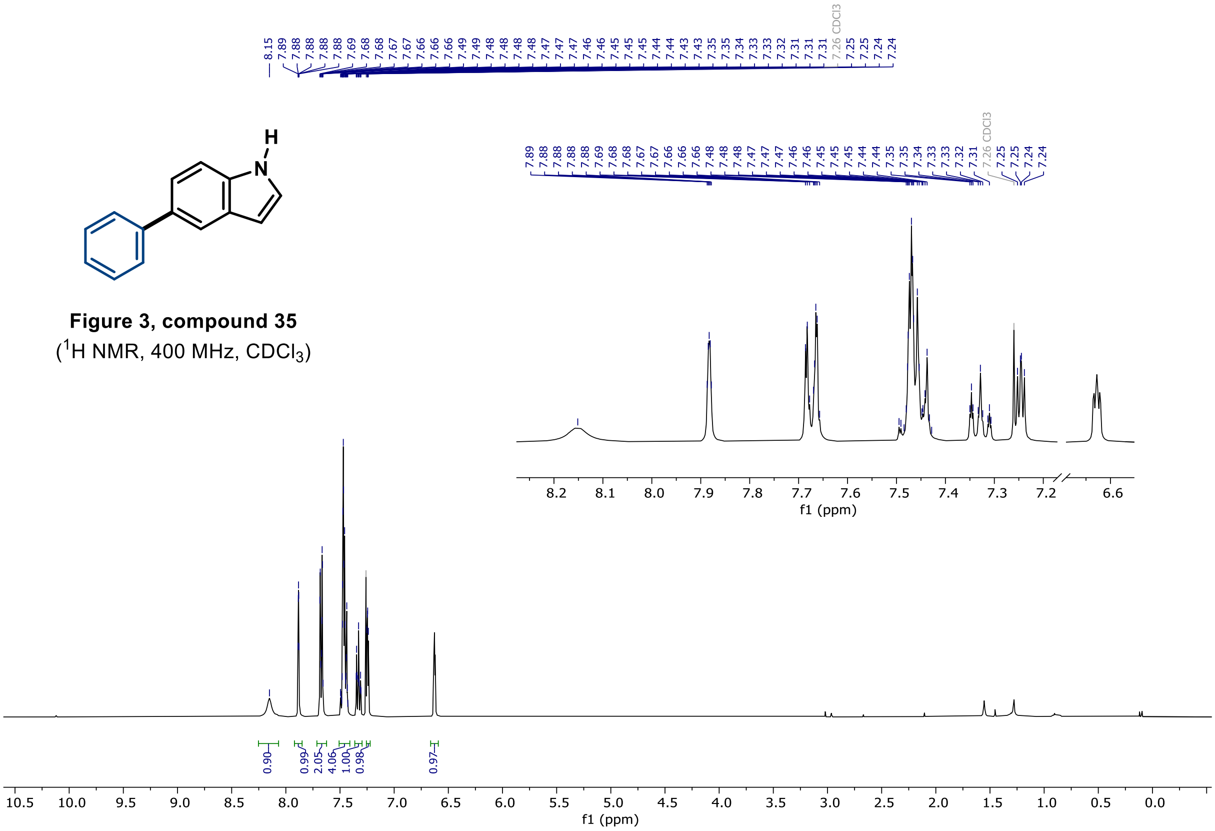

Figure 3, compound 35
(^{1}H NMR, 400 MHz, $CDCl_3$)

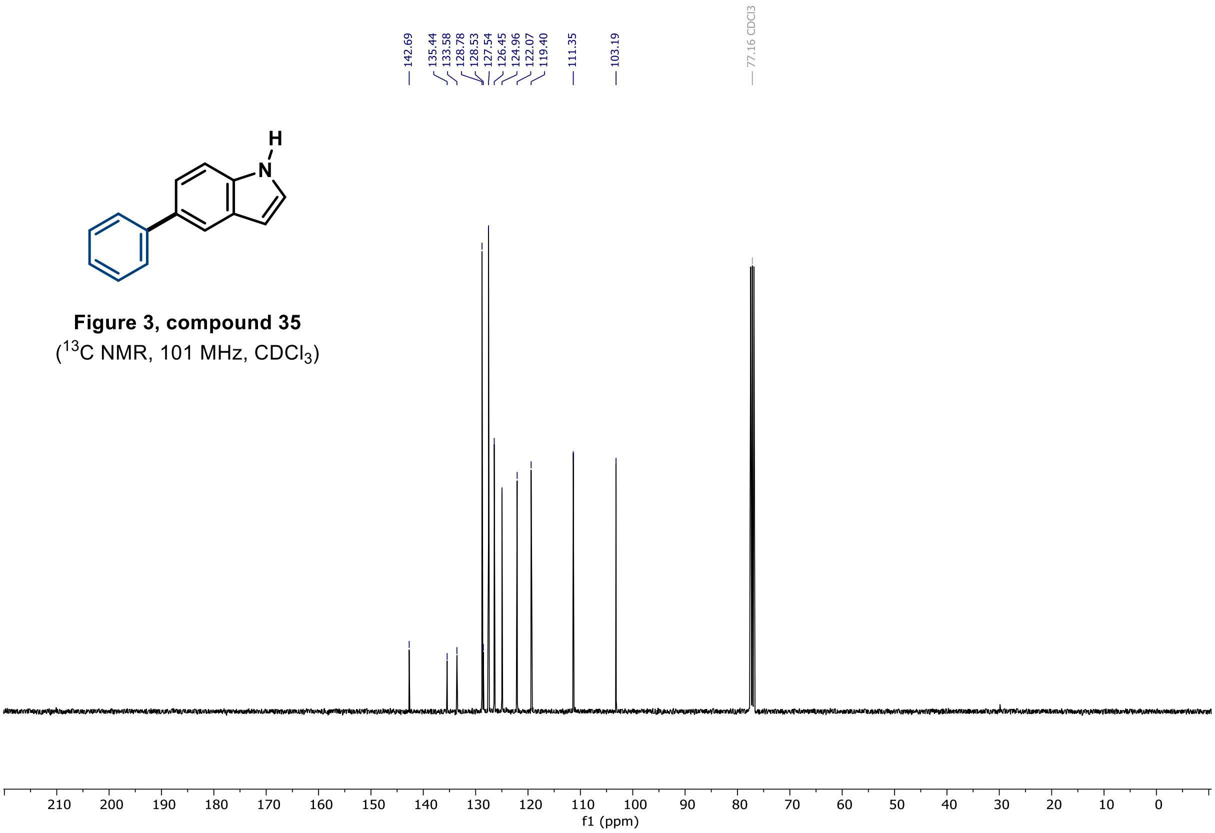

Figure 3, compound 35
(^{13}C NMR, 101 MHz, $CDCl_3$)

1-(4-Fluoro-4'-(trifluoromethyl)-[1,1'-biphenyl]-3-yl)ethan-1-one (compound 36)

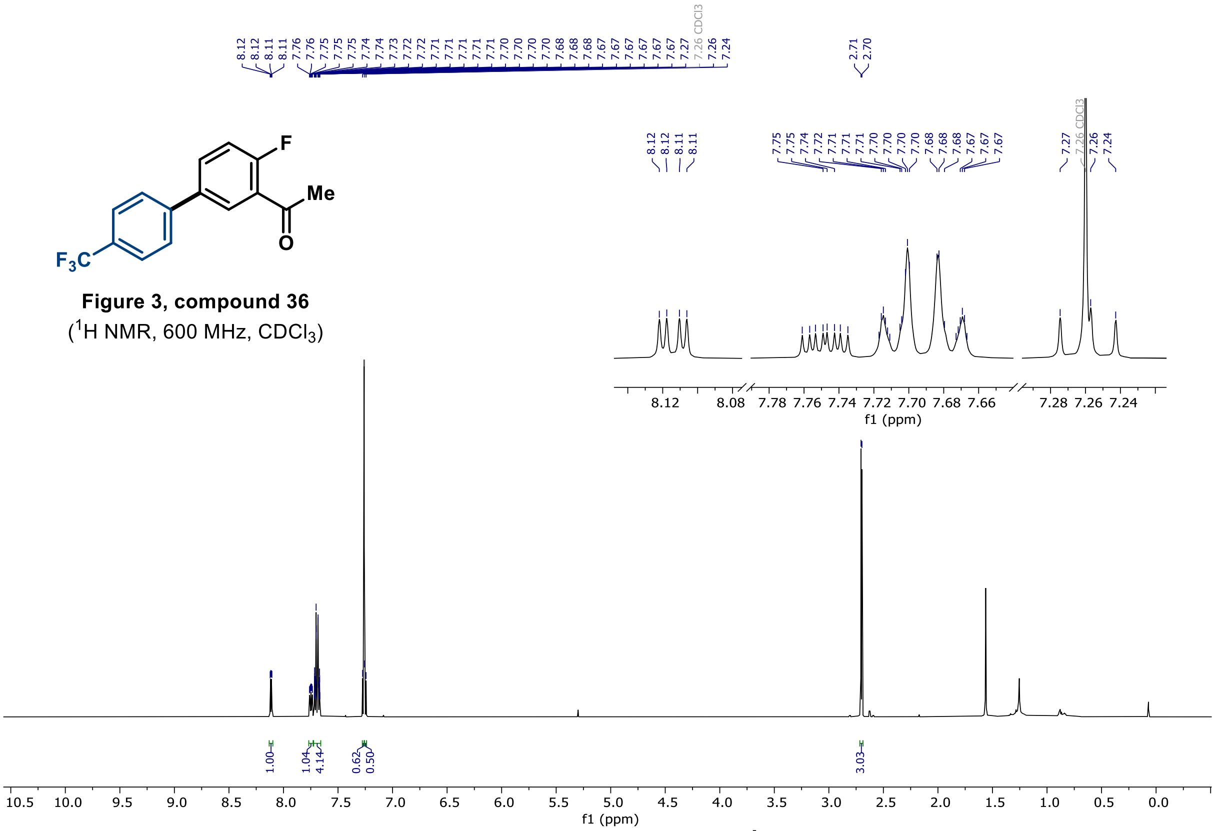

Figure 3, compound 36
(^{1}H NMR, 600 MHz, $CDCl_3$)

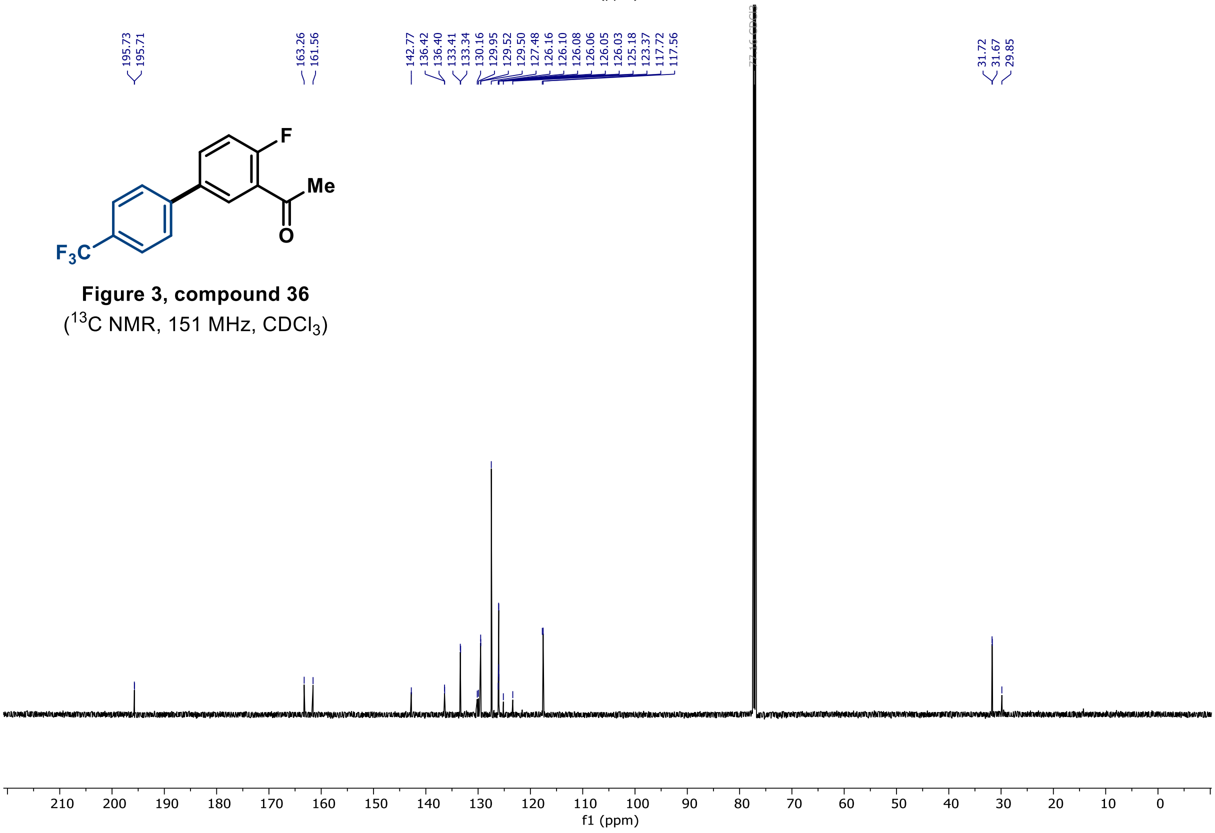

Figure 3, compound 36
(^{13}C NMR, 151 MHz, $CDCl_3$)

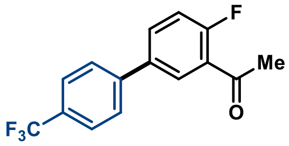

Figure 3, compound 36
(^{19}F NMR, 565 MHz, $CDCl_3$)

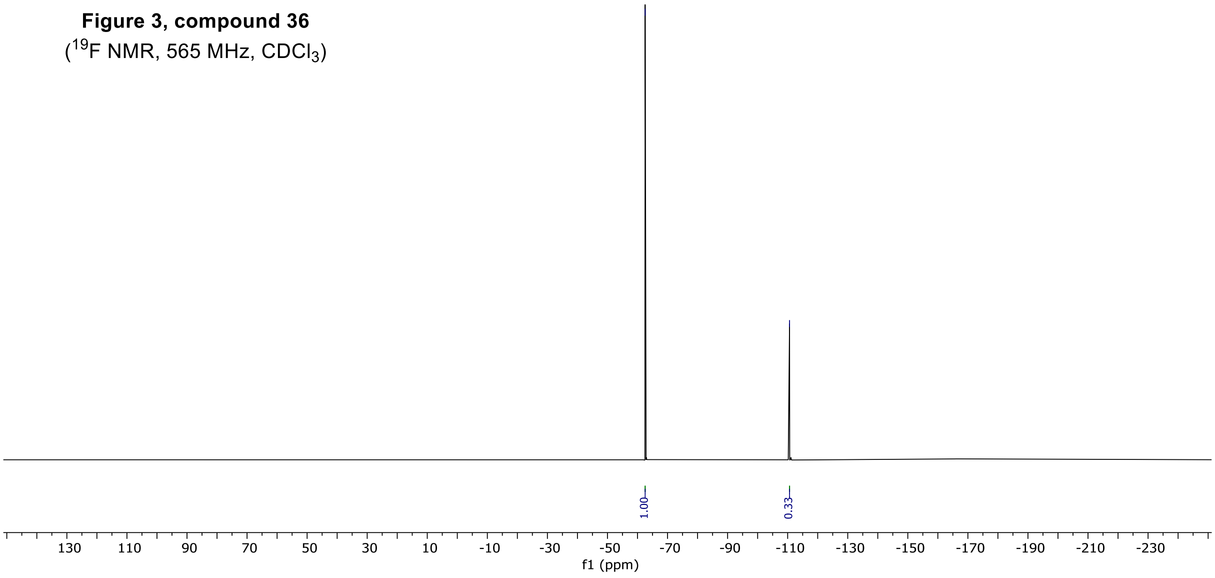

1-(4-Fluoro-[1,1'-biphenyl]-3-yl)ethan-1-one (compound 37)

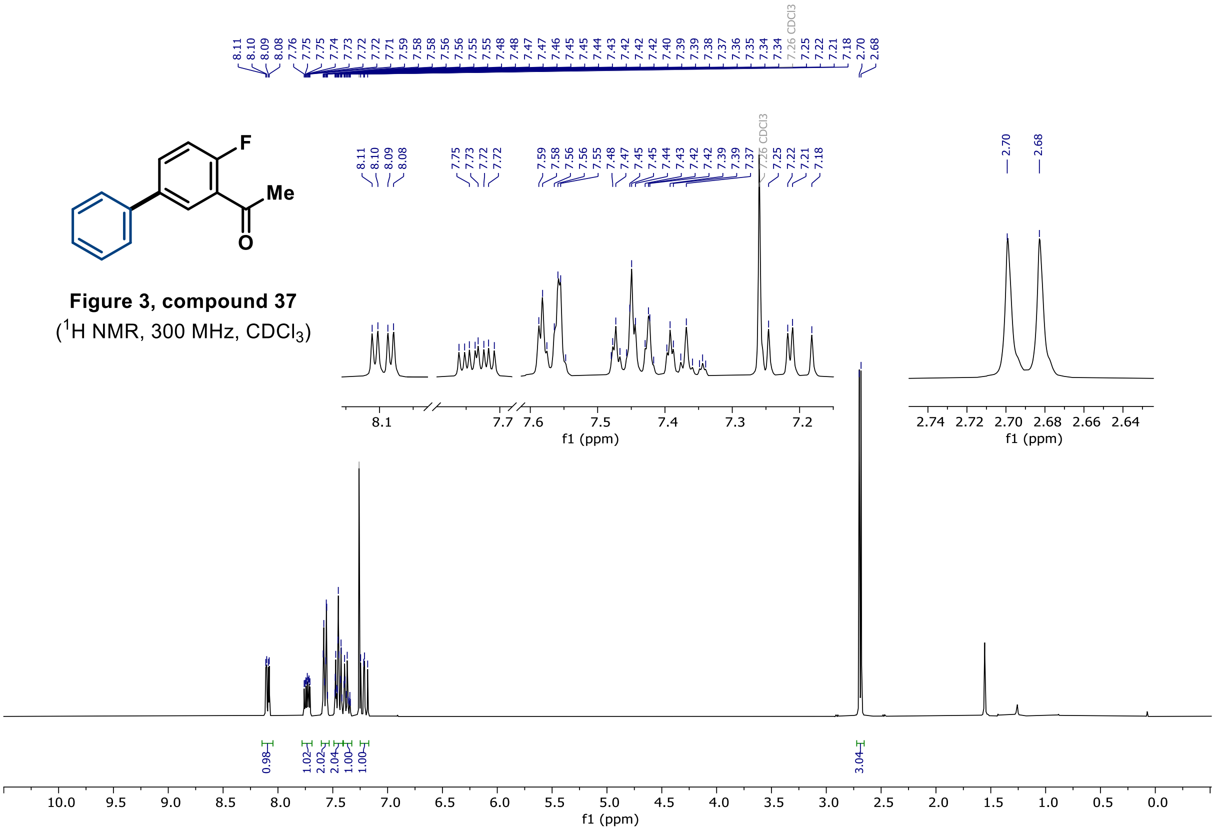

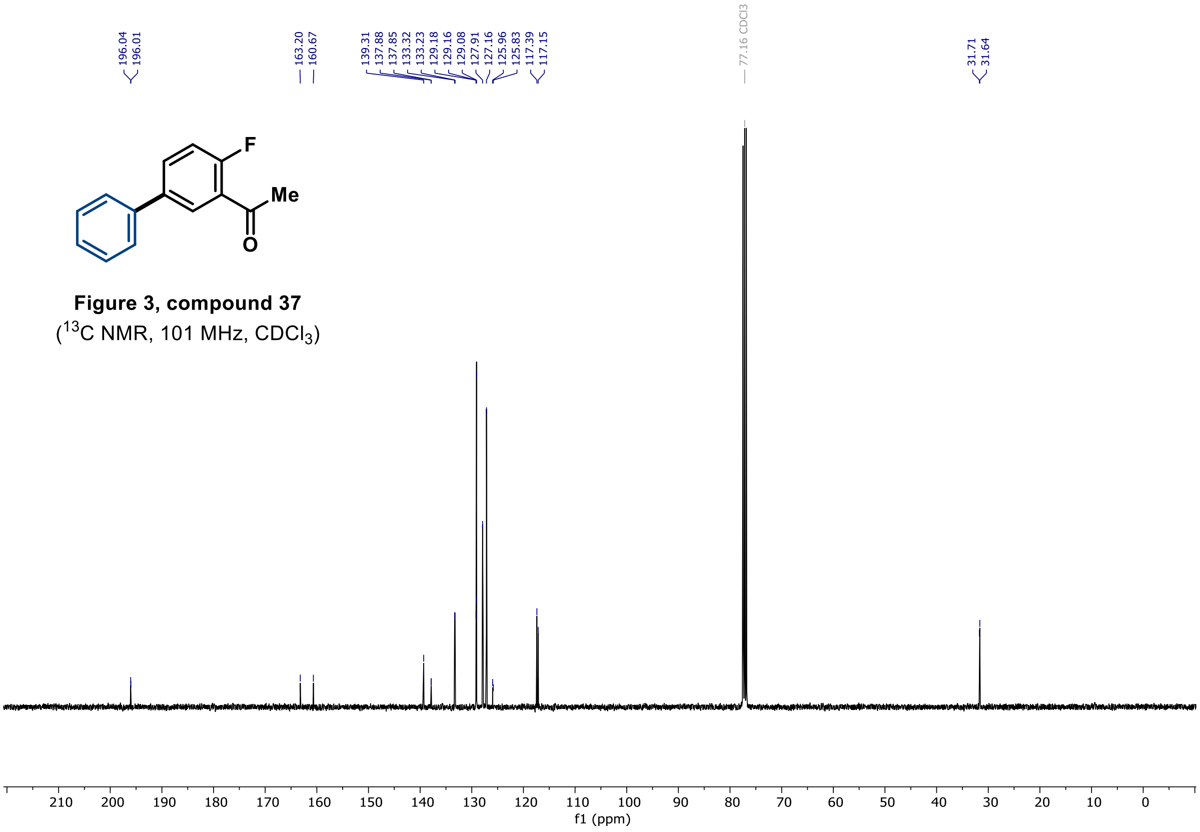

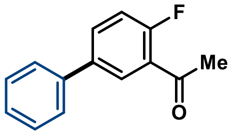

Figure 3, compound 37
(^{19}F NMR, 282 MHz, $CDCl_3$)

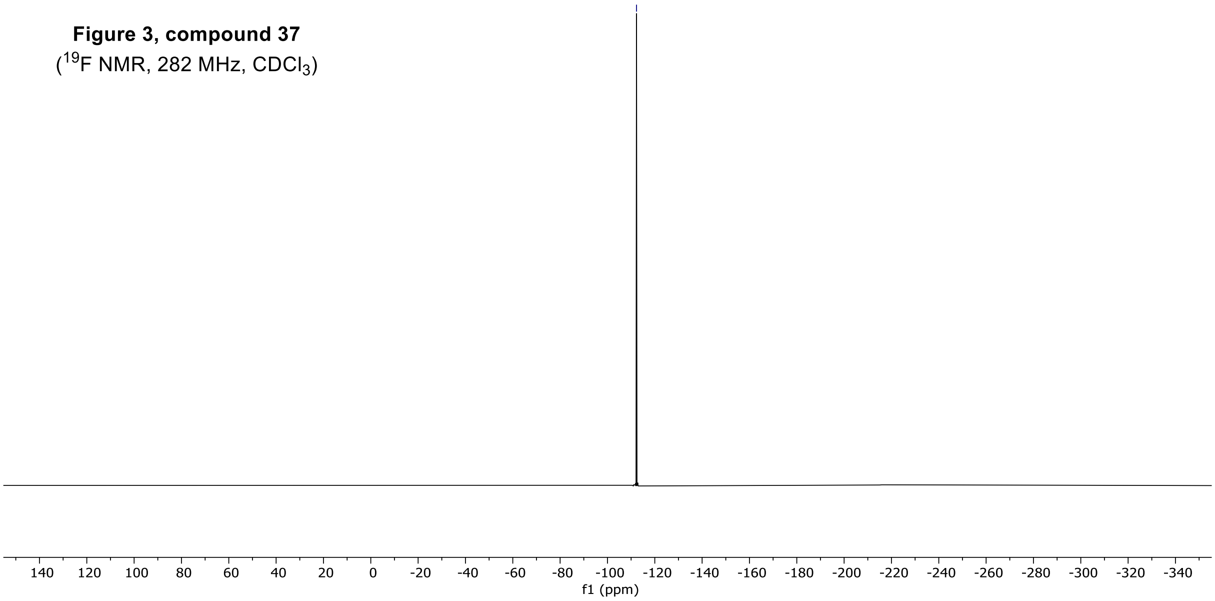

1-(4-Fluoro-4'-methoxy-[1,1'-biphenyl]-3-yl)ethan-1-one (compound 38)

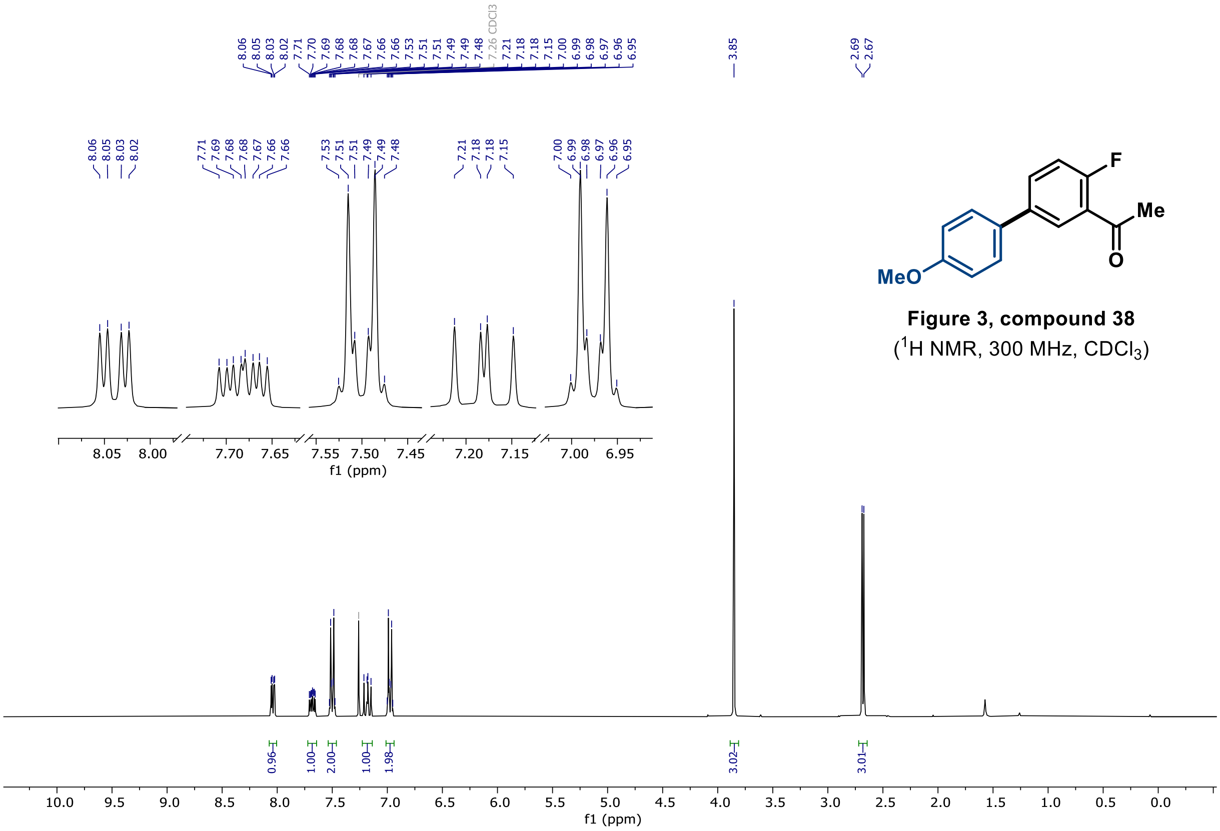

Figure 3, compound 38
(^{1}H NMR, 300 MHz, $CDCl_3$)

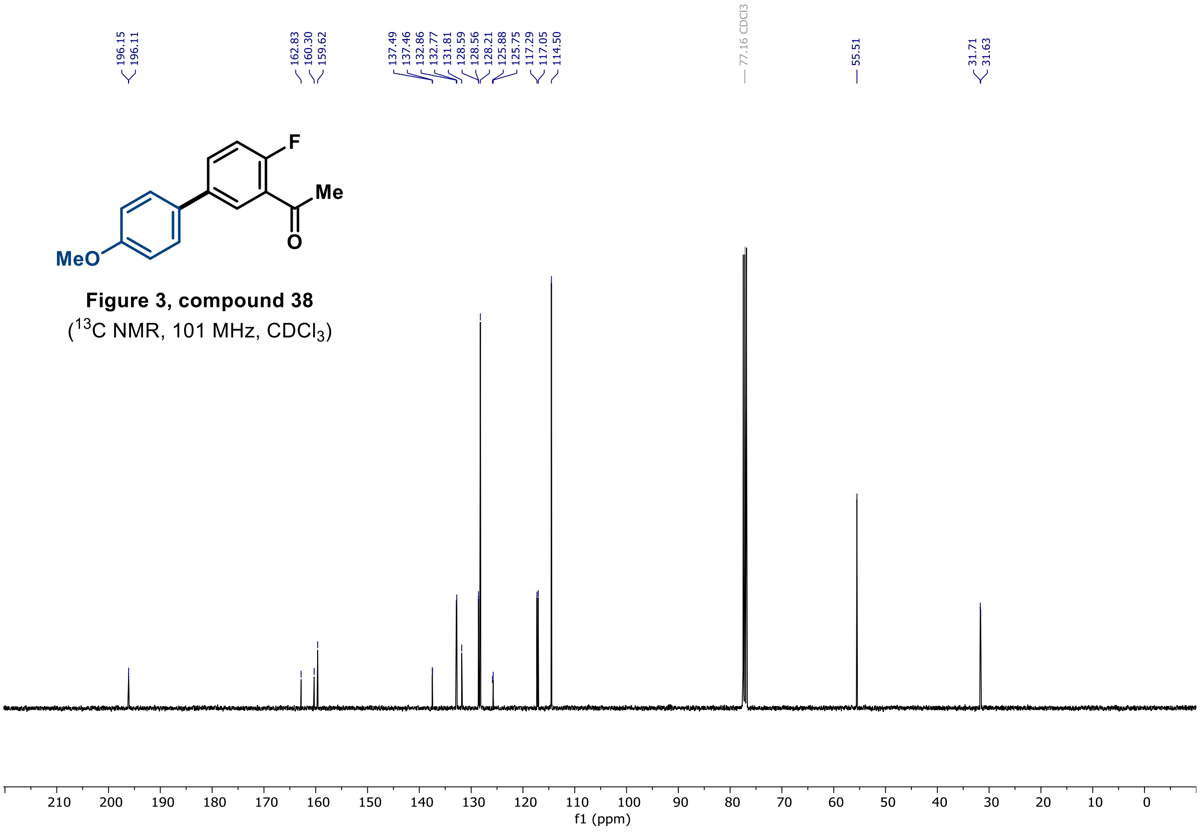

Figure 3, compound 38
(^{13}C NMR, 101 MHz, $CDCl_3$)

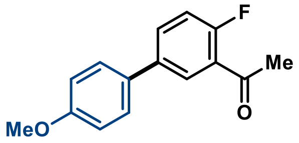

Figure 3, compound 38
(^{19}F NMR, 282 MHz, $CDCl_3$)

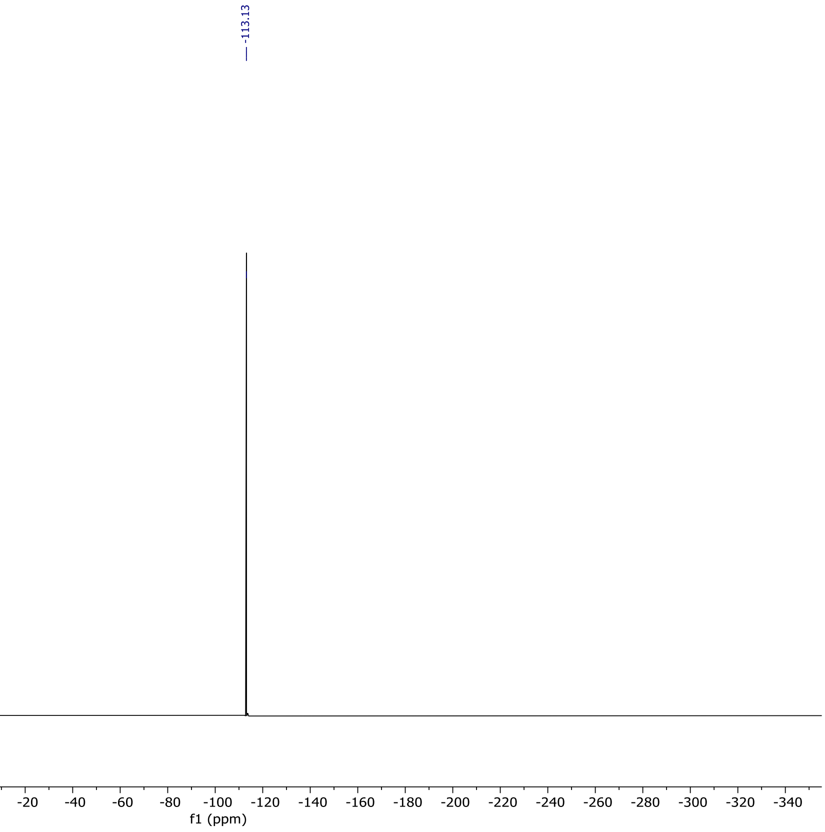

1-(2-Fluoro-5-(pyrazin-2-yl)phenyl)ethan-1-one (compound 39)

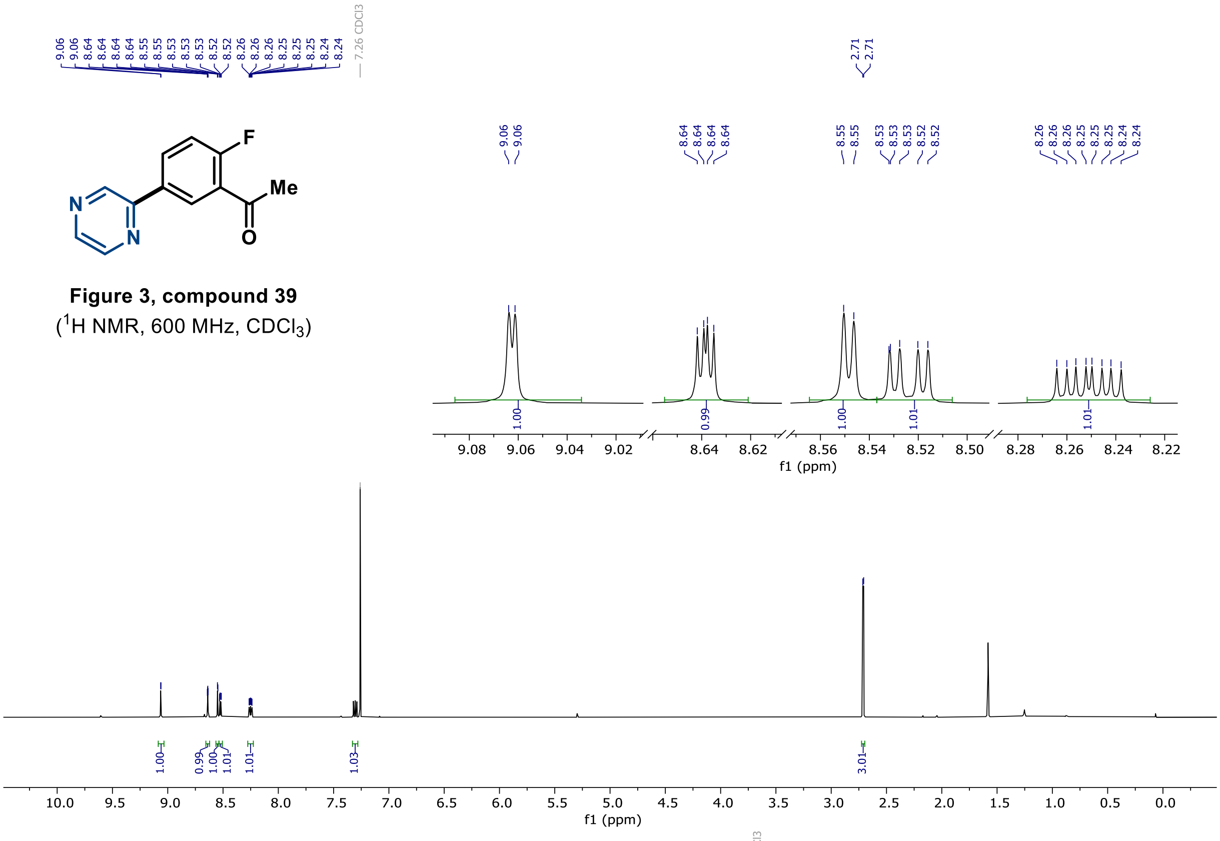

Figure 3, compound 39
(^{1}H NMR, 600 MHz, $CDCl_3$)

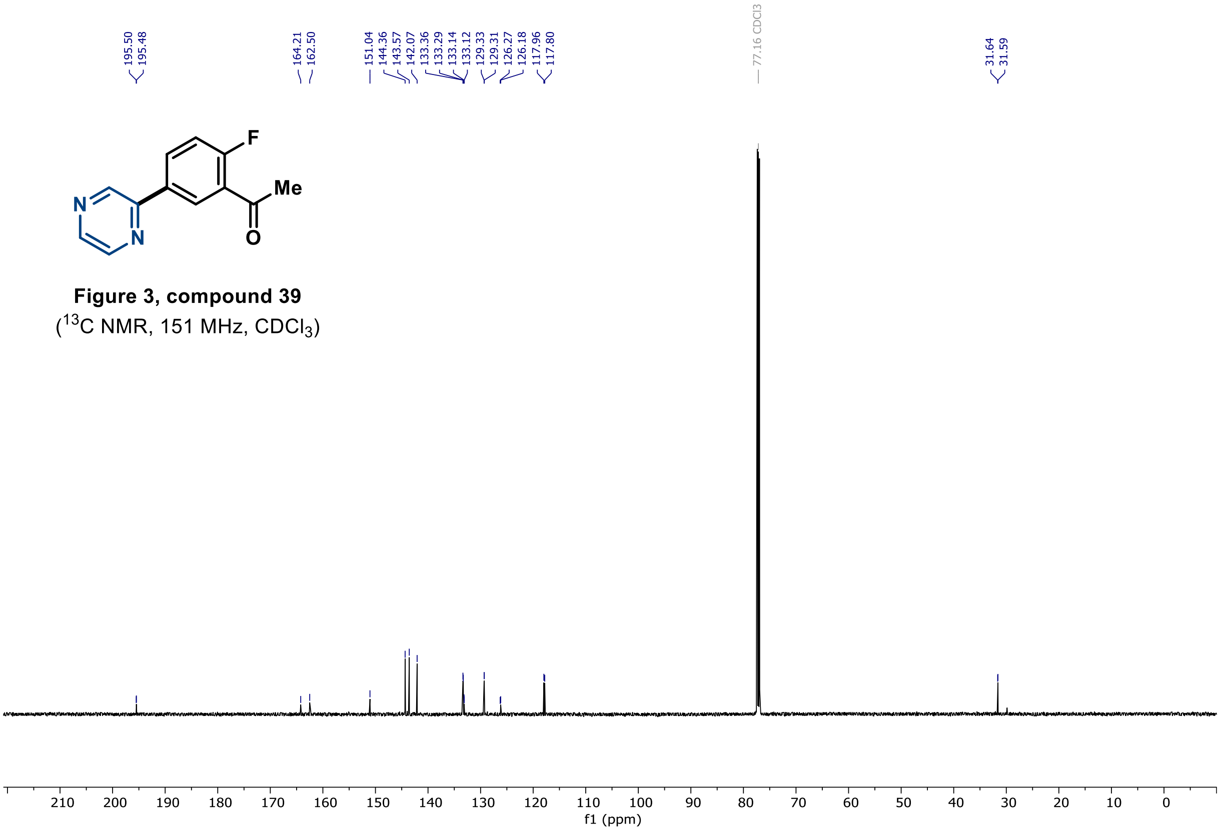

Figure 3, compound 39
(^{13}C NMR, 151 MHz, $CDCl_3$)

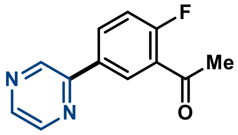

Figure 3, compound 39
(^{19}F NMR, 565 MHz, $CDCl_3$)

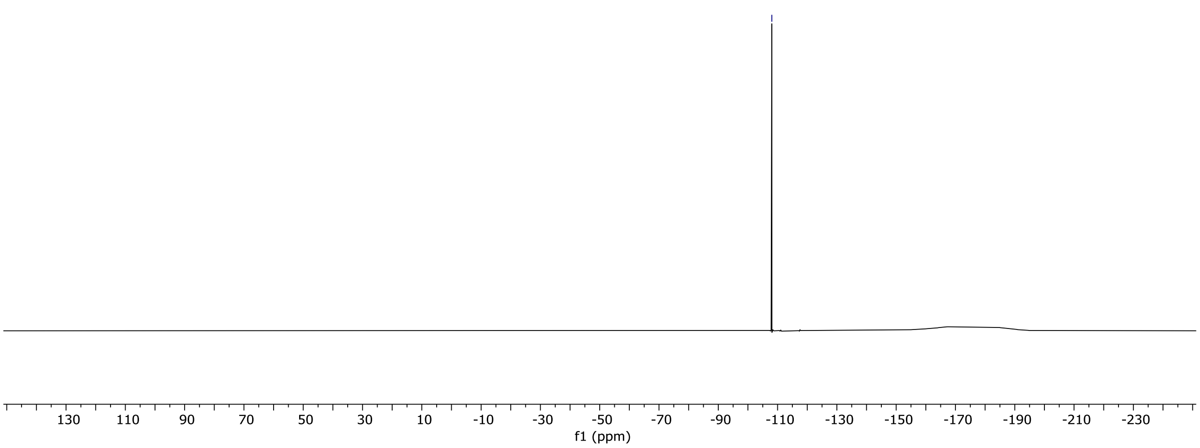

1-(5-(8-Butoxyquinolin-5-yl)-2-fluorophenyl)ethan-1-one (compound 40)

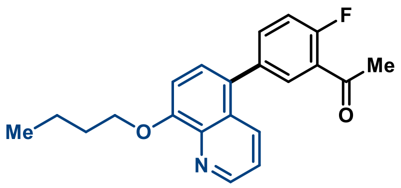

Figure 3, compound 40
(^{1}H NMR, 300 MHz, $CDCl_3$)

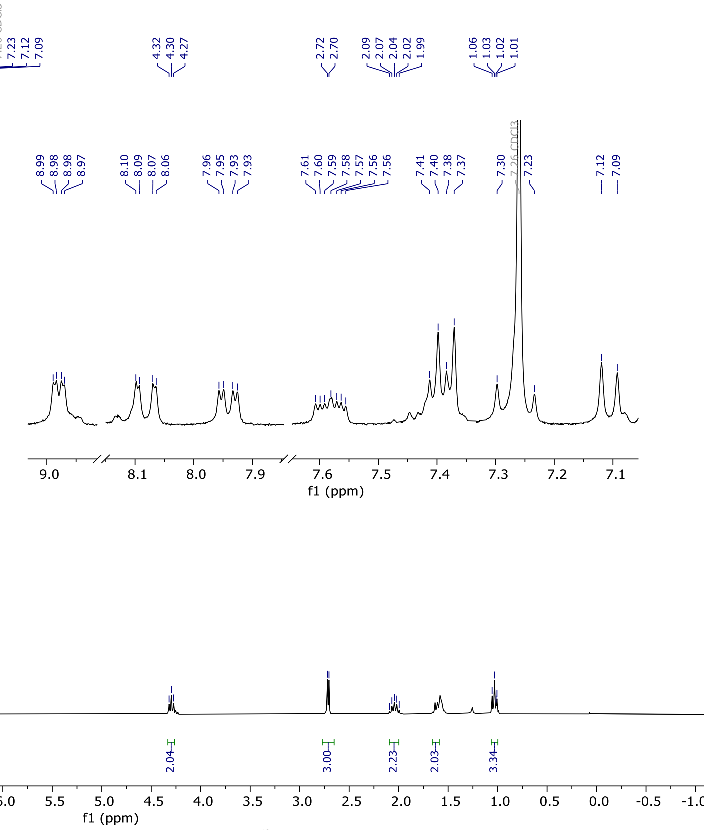

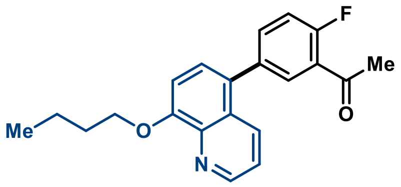

Figure 3, compound 40
(^{13}C NMR, 101 MHz, $CDCl_3$)

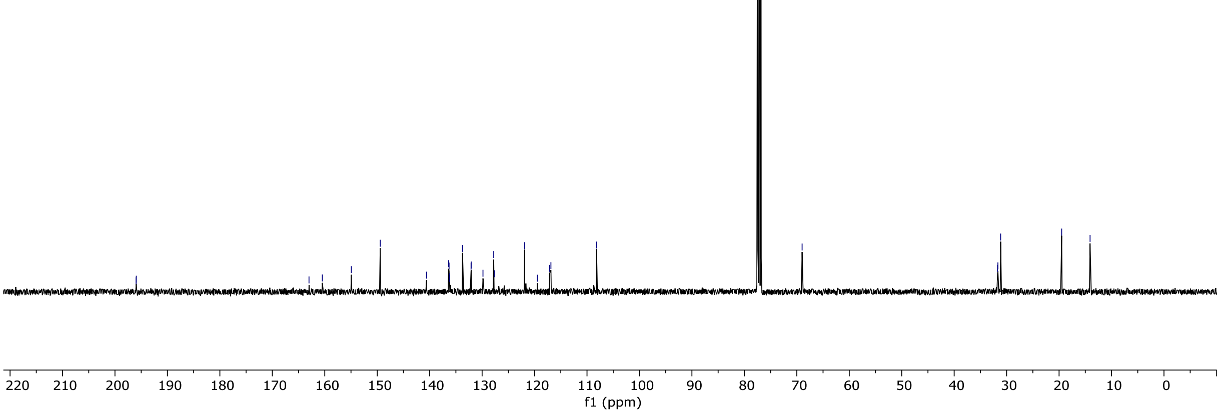

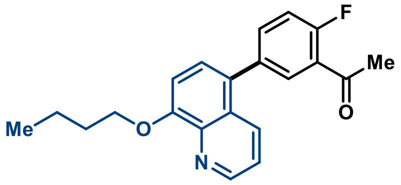

Figure 3, compound 40
(^{19}F NMR, 282 MHz, $CDCl_3$)

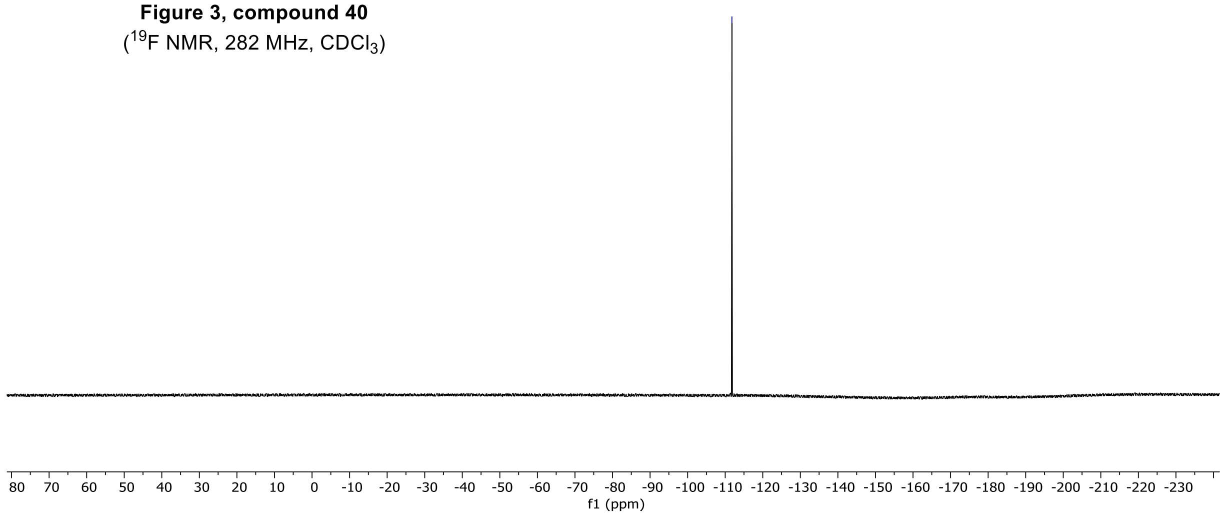

2-((1-(4-(4-(Pyridin-4-yl)phenoxy)phenoxy)propan-2-yl)oxy)pyridine (compound 41)

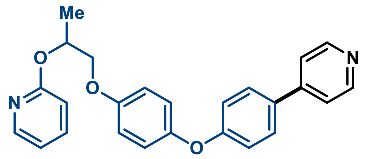

Figure 3, compound 41
(^{1}H NMR, 400 MHz, $CDCl_3$)

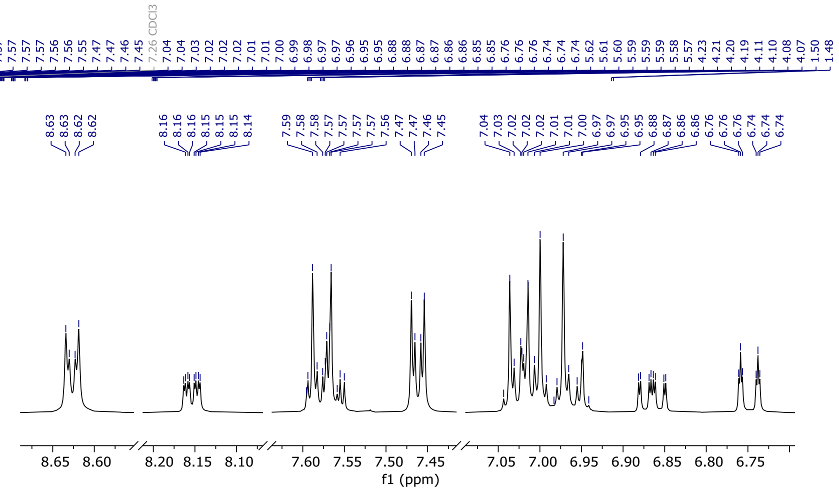

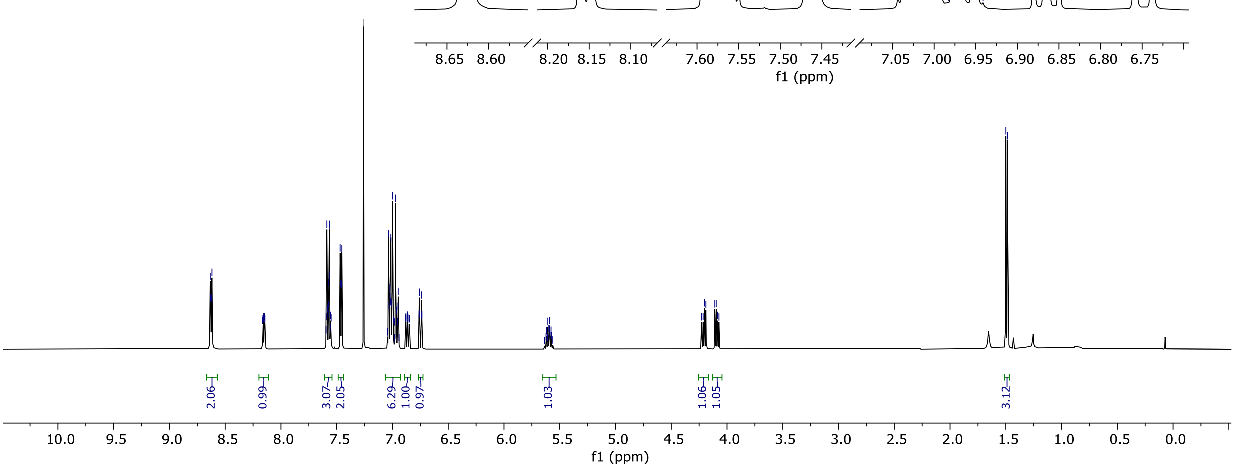

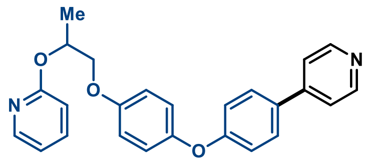

Figure 3, compound 41
(^{13}C NMR, 101 MHz, $CDCl_3$)

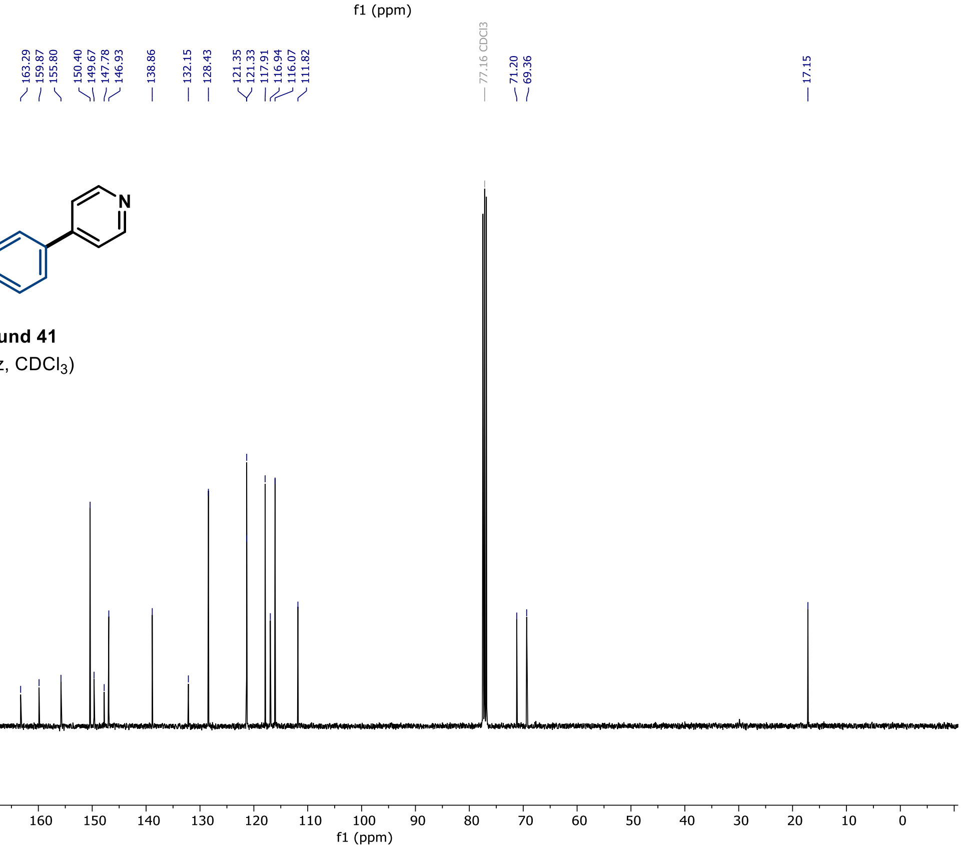

4-Methoxy-4'-(trifluoromethyl)-1,1'-biphenyl (compound 42)

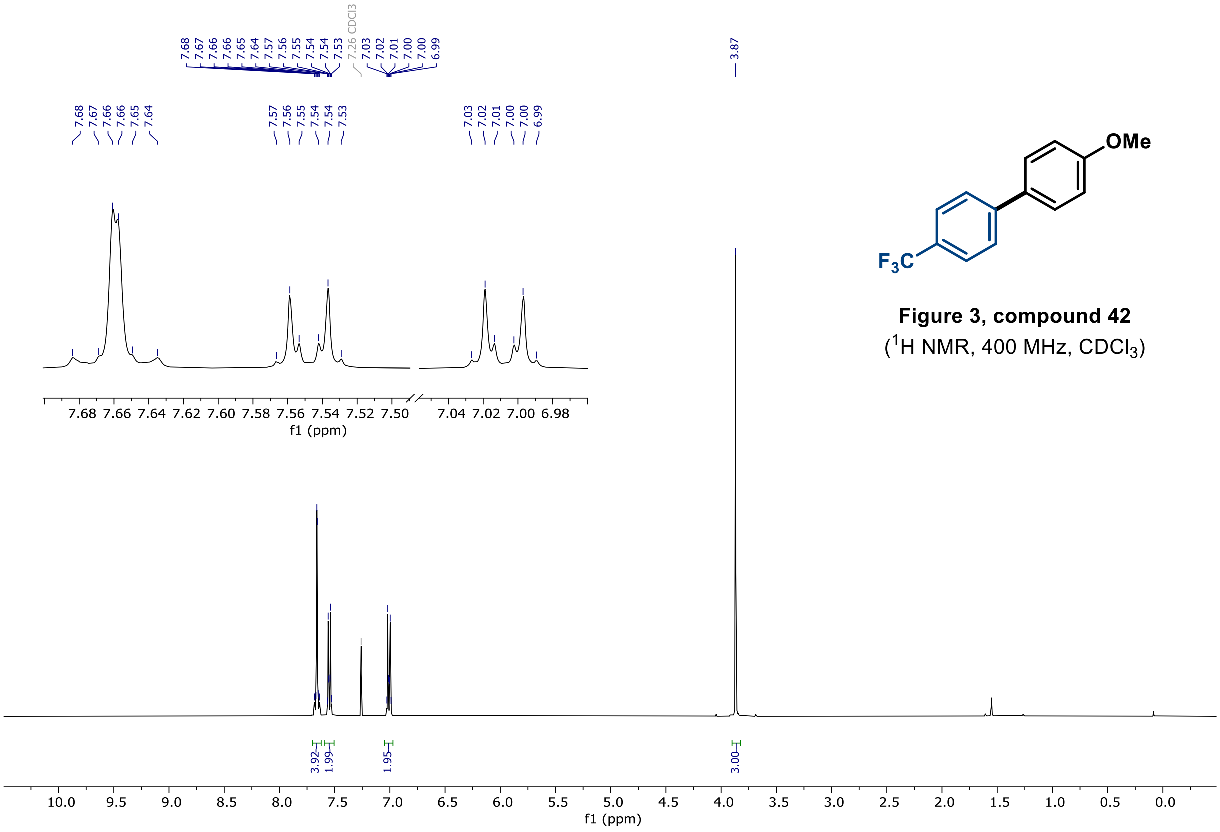

Figure 3, compound 42
(^{1}H NMR, 400 MHz, $CDCl_3$)

.

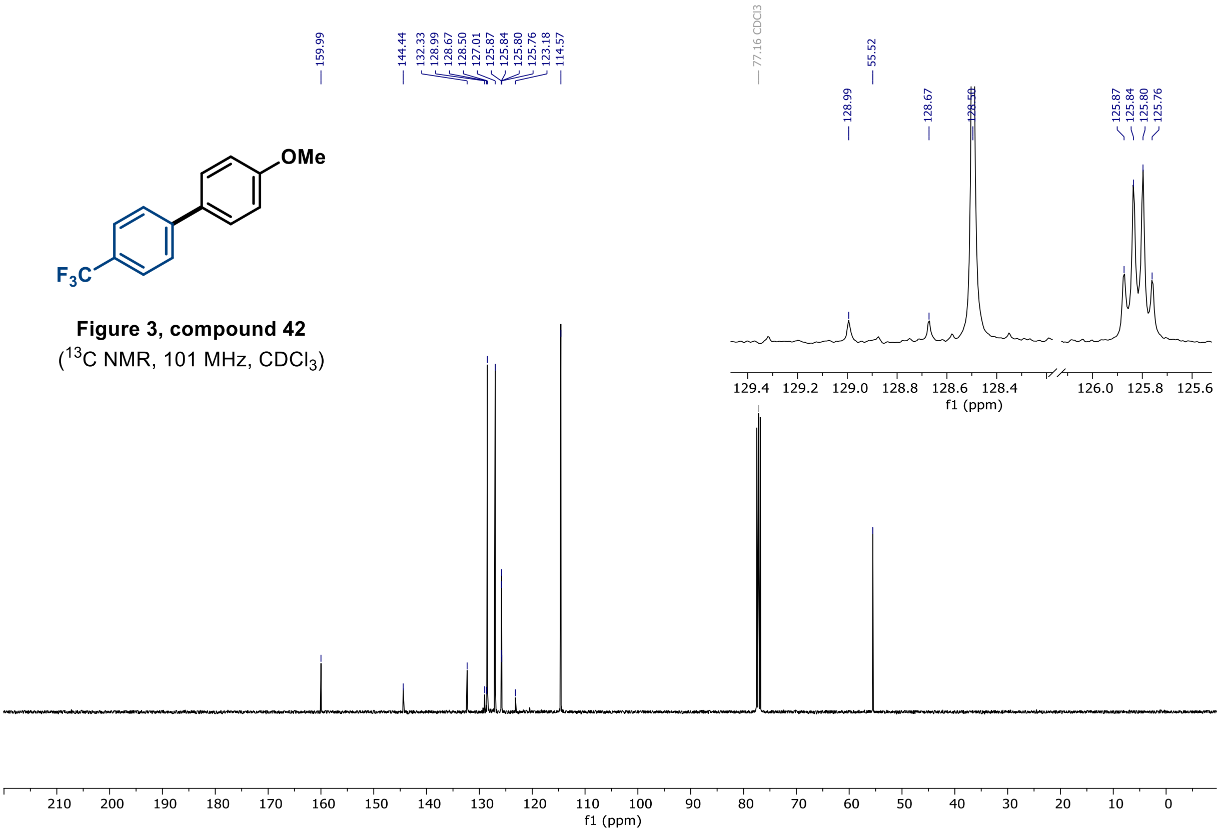

Figure 3, compound 42
(^{13}C NMR, 101 MHz, $CDCl_3$)

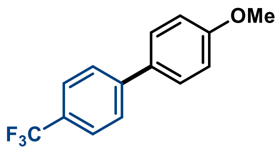

Figure 3, compound 42
(^{19}F NMR, 282 MHz, $CDCl_3$)

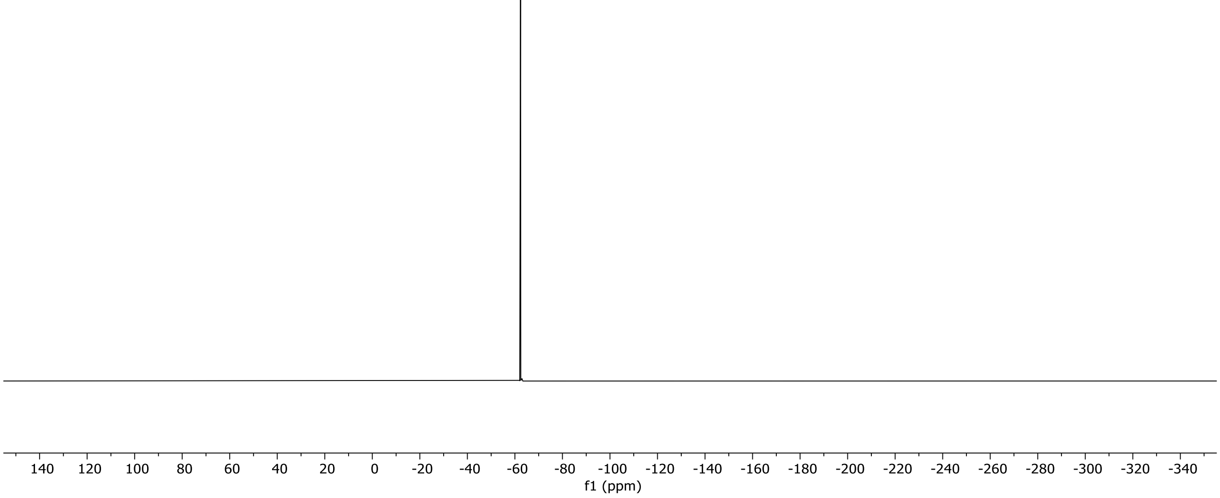

4-(*tert*-Butyl)-4'-methoxy-1,1'-biphenyl (compound 43)

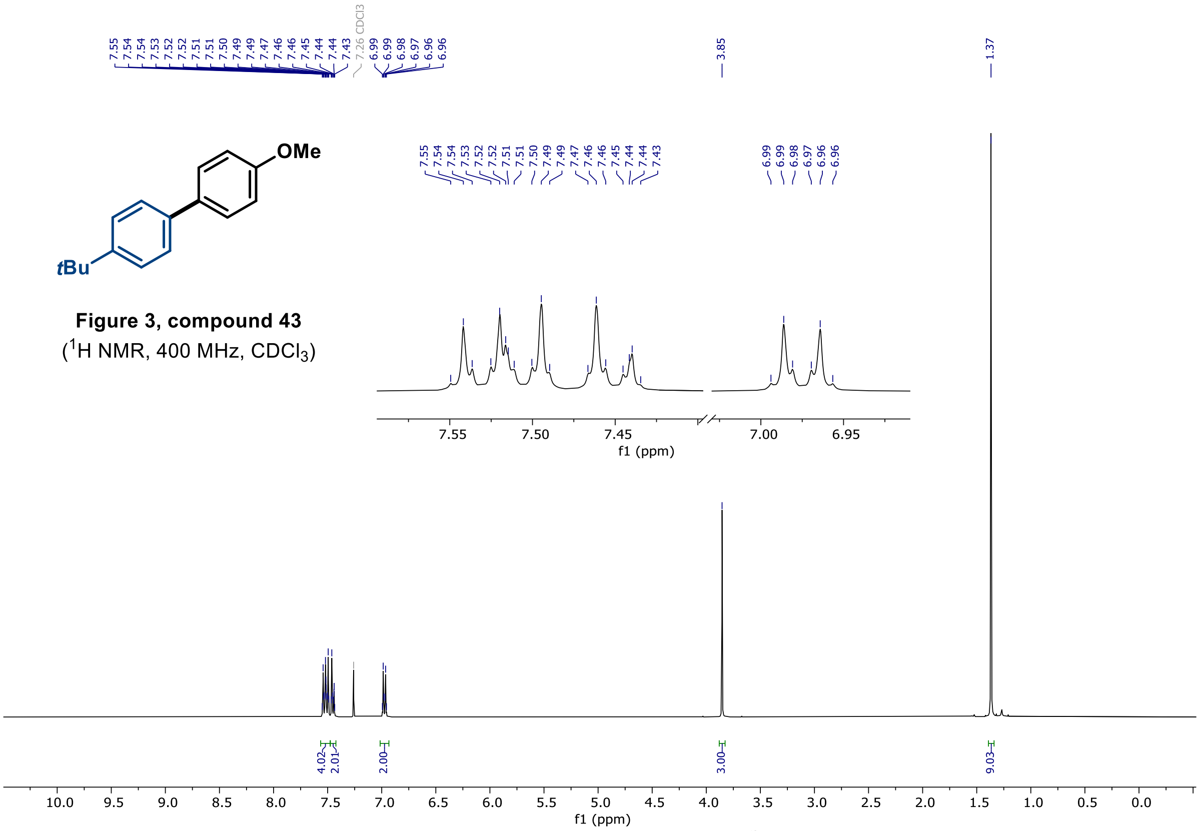

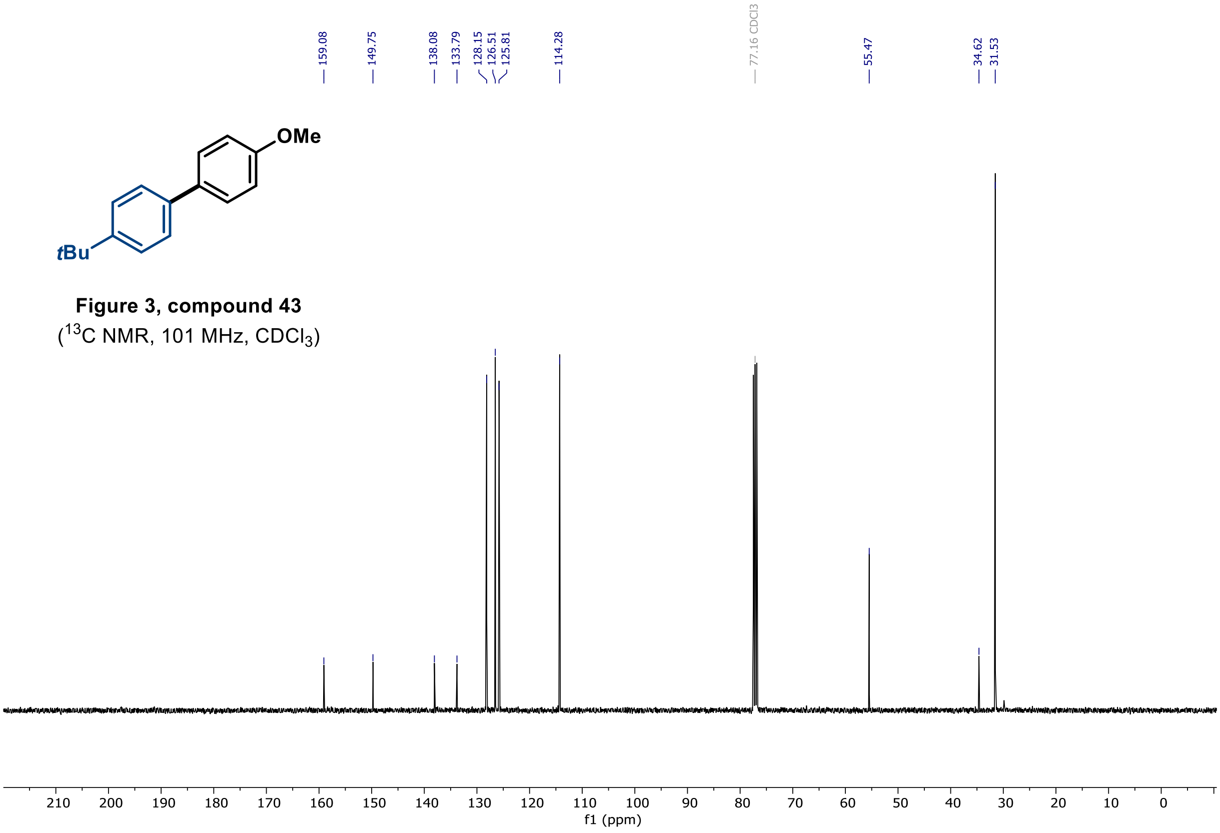

3,5-Dibromo-4'-methoxy-1,1'-biphenyl (compound 44)

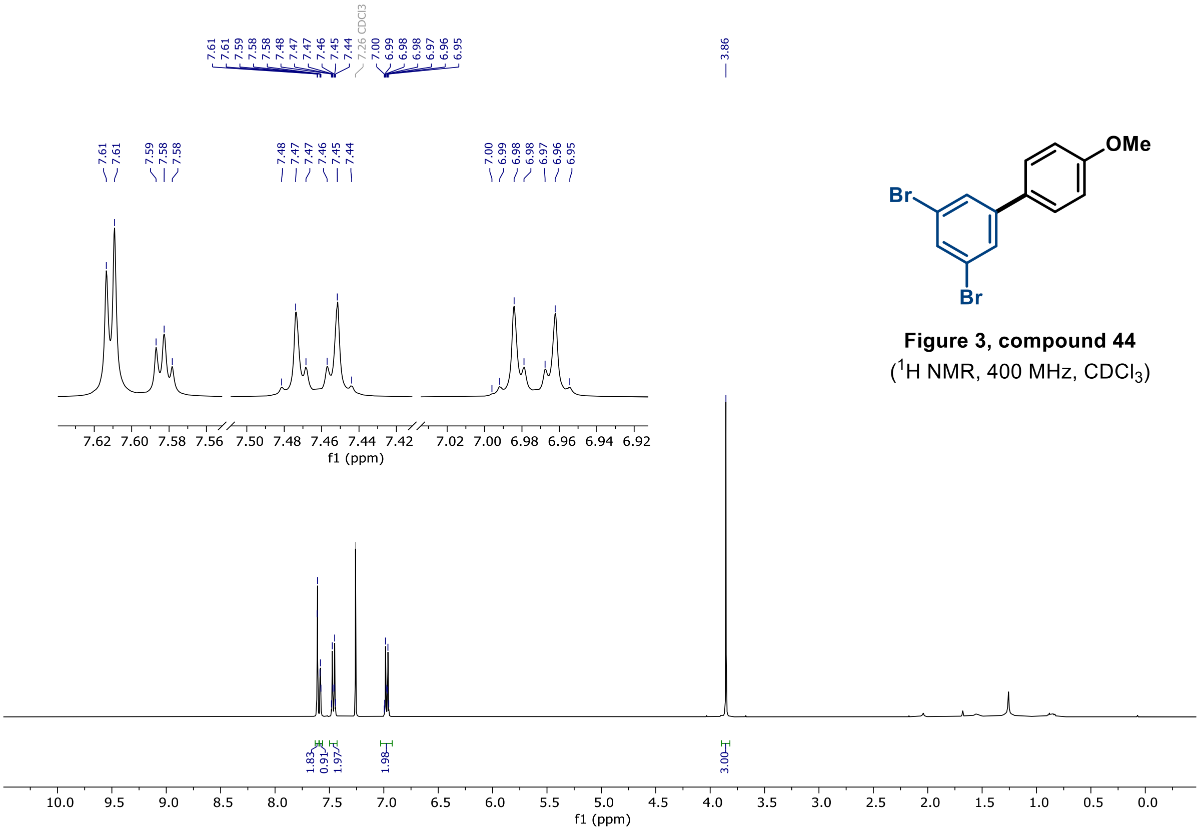

Figure 3, compound 44
(^{1}H NMR, 400 MHz, $CDCl_3$)

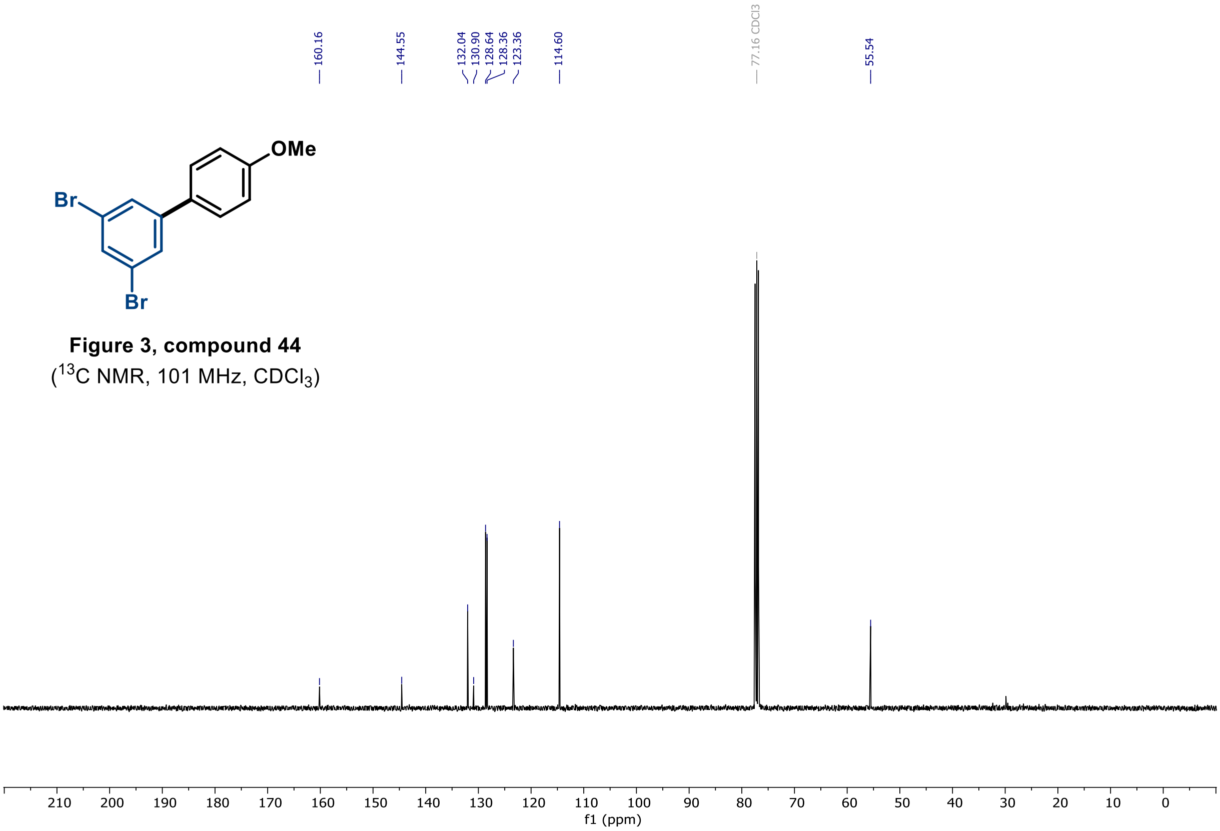

Figure 3, compound 44
(^{13}C NMR, 101 MHz, $CDCl_3$)

2-(4-Methoxyphenyl)benzo[b]thiophene (compound 45)

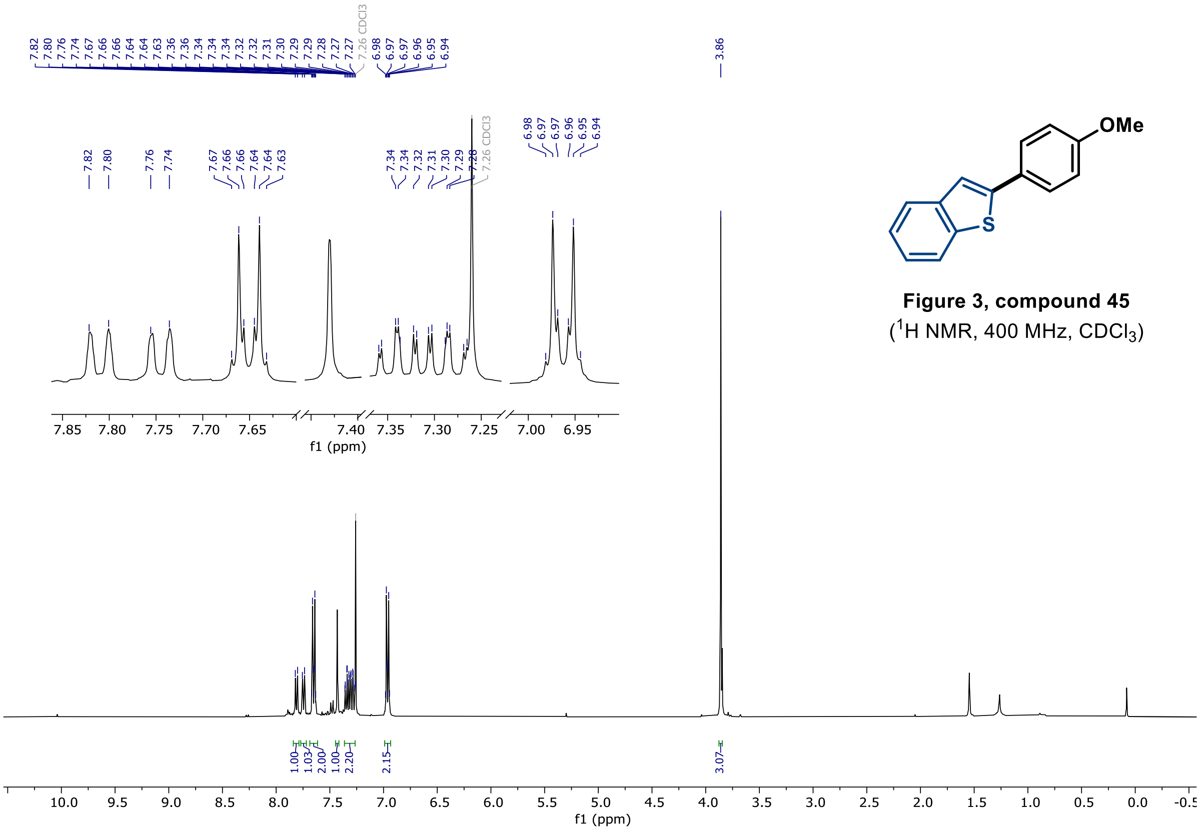

Figure 3, compound 45
(^{1}H NMR, 400 MHz, $CDCl_3$)

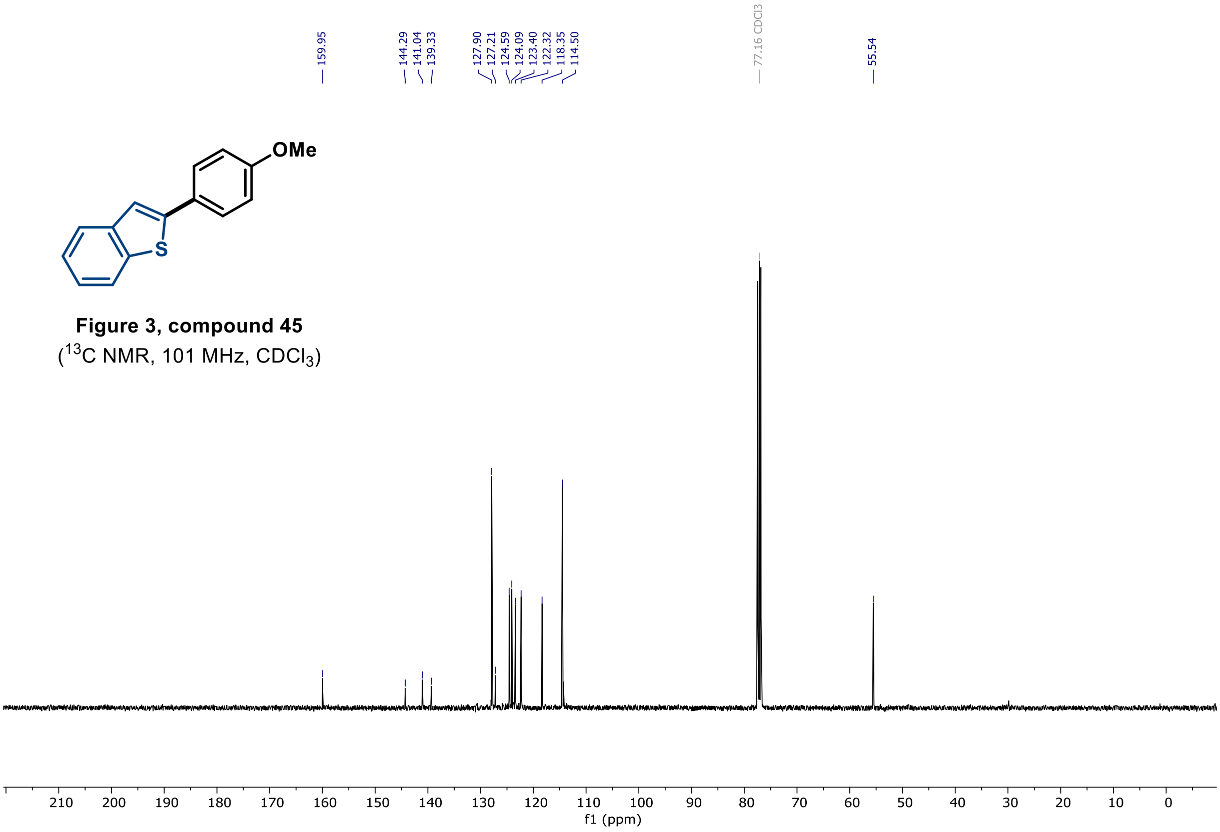

Figure 3, compound 45
(^{13}C NMR, 101 MHz, $CDCl_3$)

6-(4-Methoxyphenyl)quinoline (compound 46)

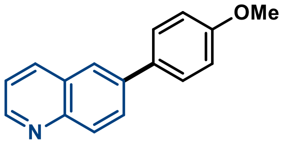

Figure 3, compound 46
(^{1}H NMR, 400 MHz, $CDCl_3$)

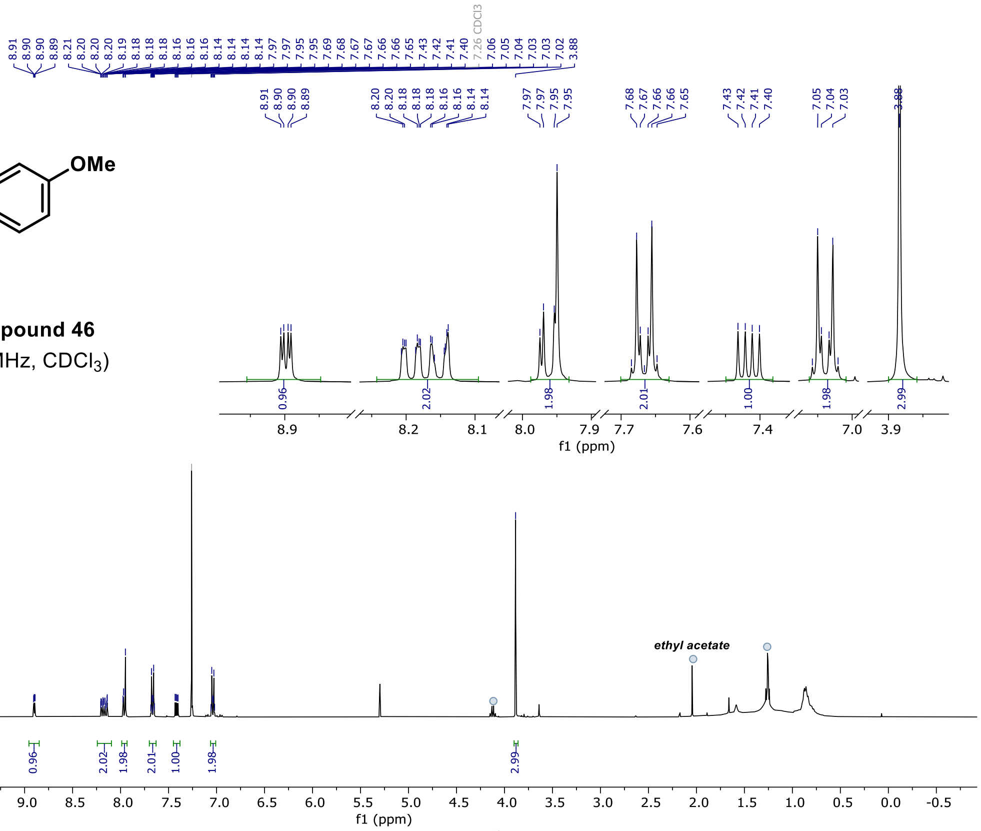

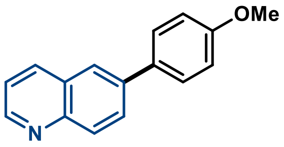

Figure 3, compound 46
(^{13}C NMR, 101 MHz, $CDCl_3$)

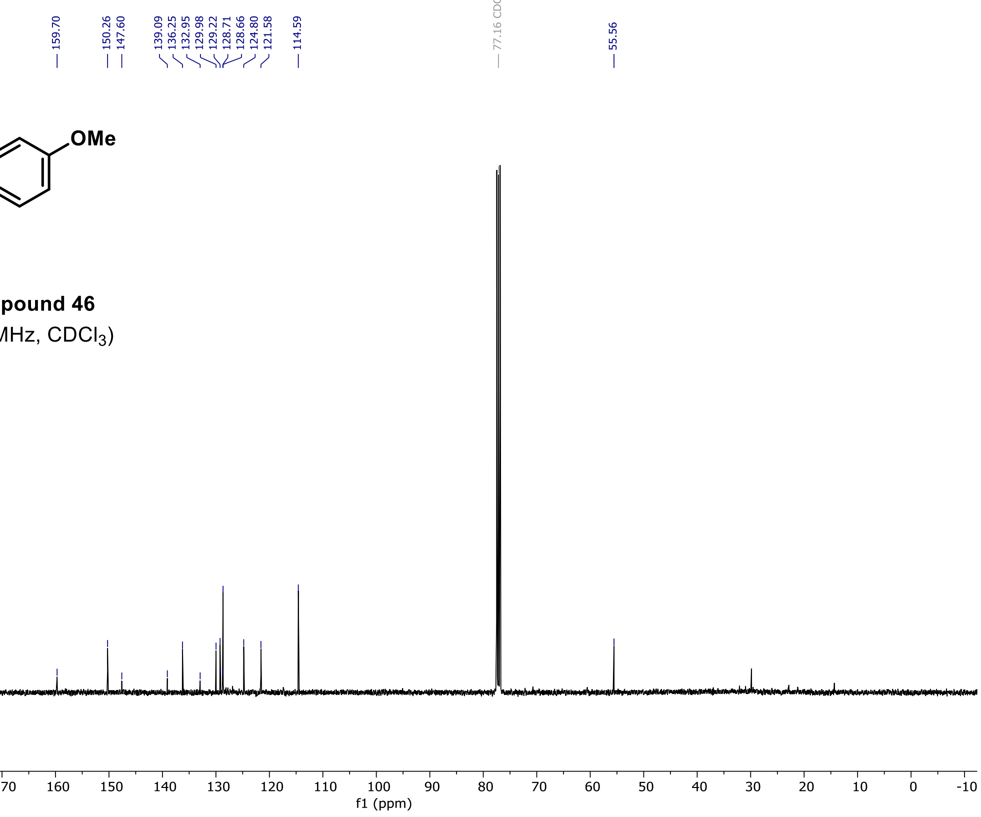

5-(4-(4-(2-(Pyridin-2-yloxy)propoxy)phenoxy)phenyl)-1H-indole (compound 47)

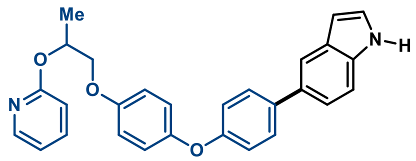

Figure 3, compound 47
(^{1}H NMR, 400 MHz, $CDCl_3$)

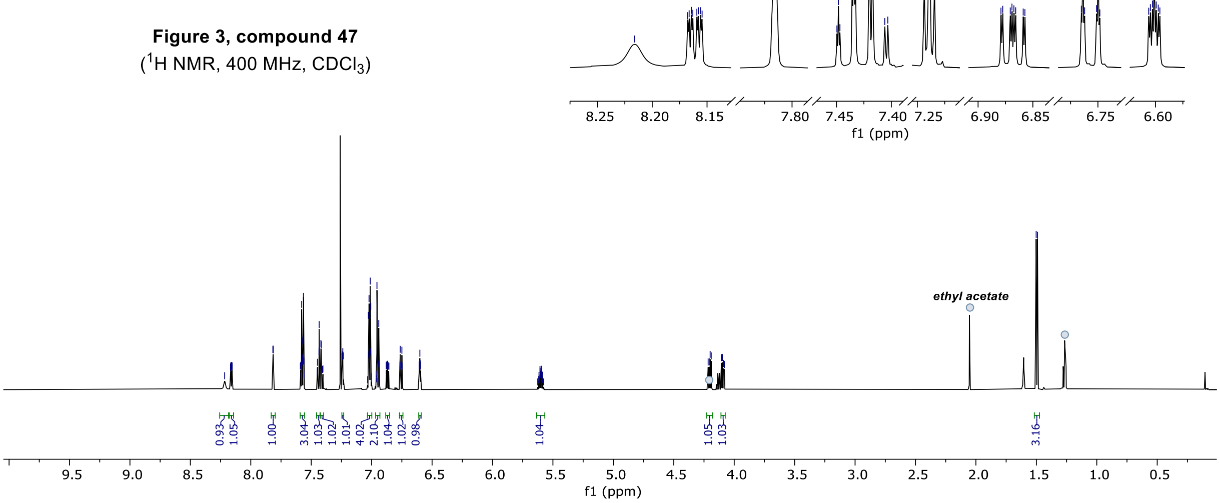

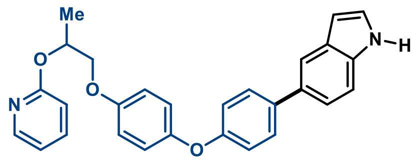

Figure 3, compound 47
(^{13}C NMR, 101 MHz, $CDCl_3$)

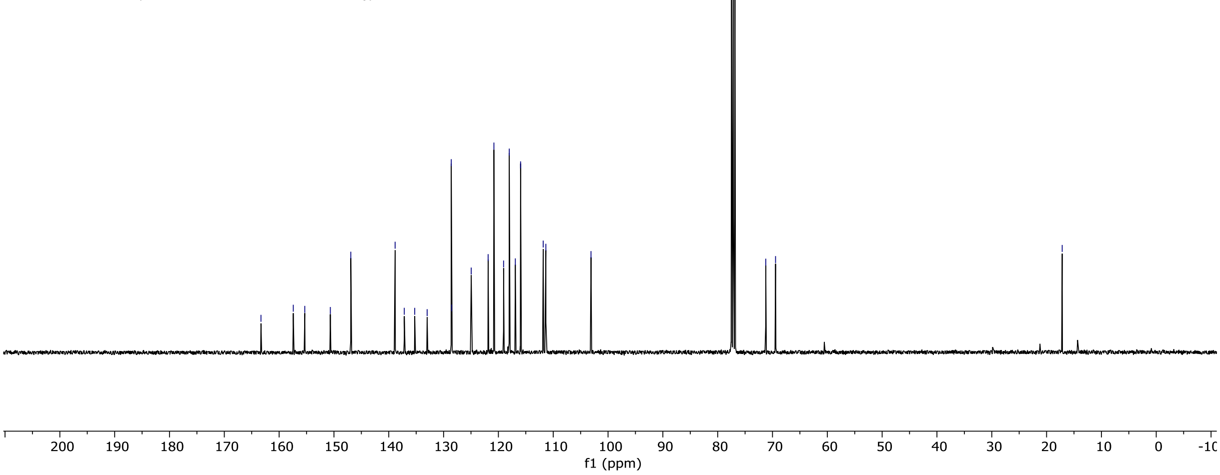

(*S*)-*N*-(3-(4-(Dibenzo[b,d]furan-2-yl)-2-methylphenoxy)-3-phenylpropyl)-2,2,2-trifluoro-*N*-methylacetamide (compound 48)

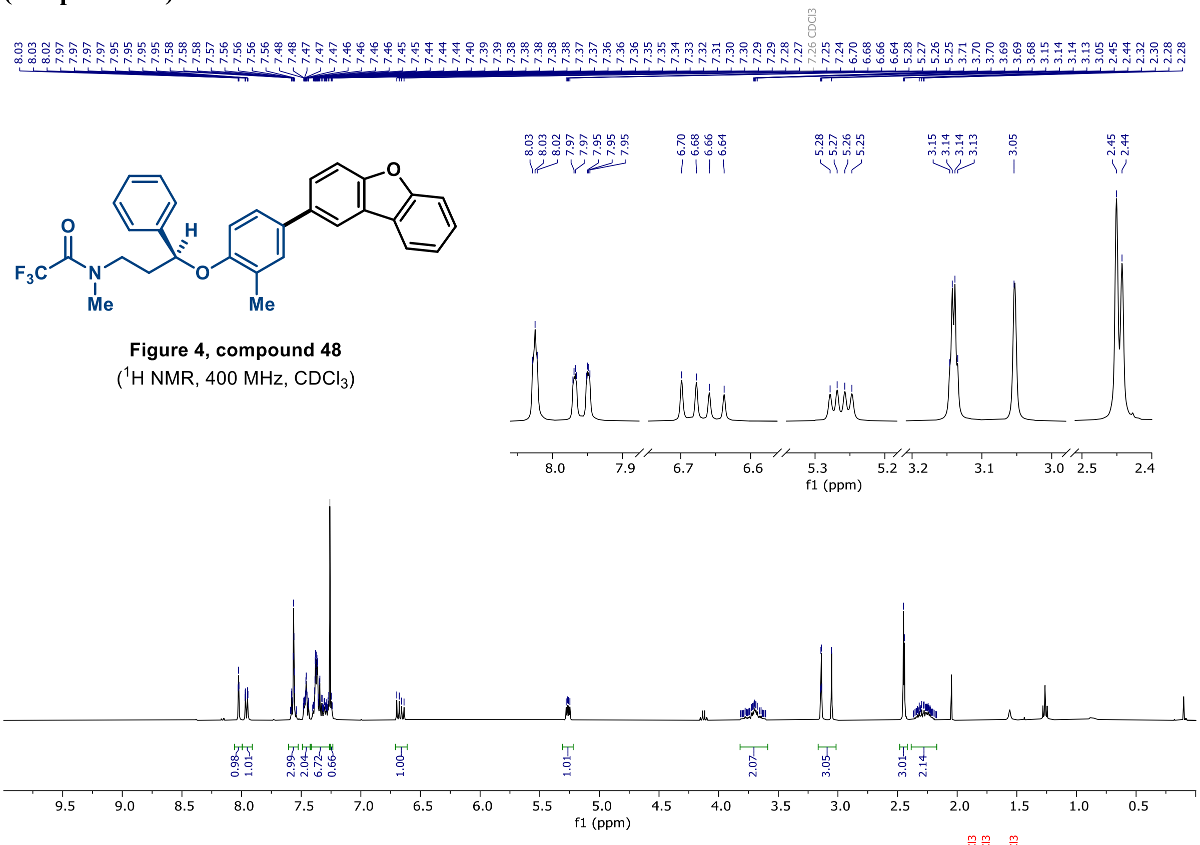

Figure 4, compound 48
(^{1}H NMR, 400 MHz, $CDCl_3$)

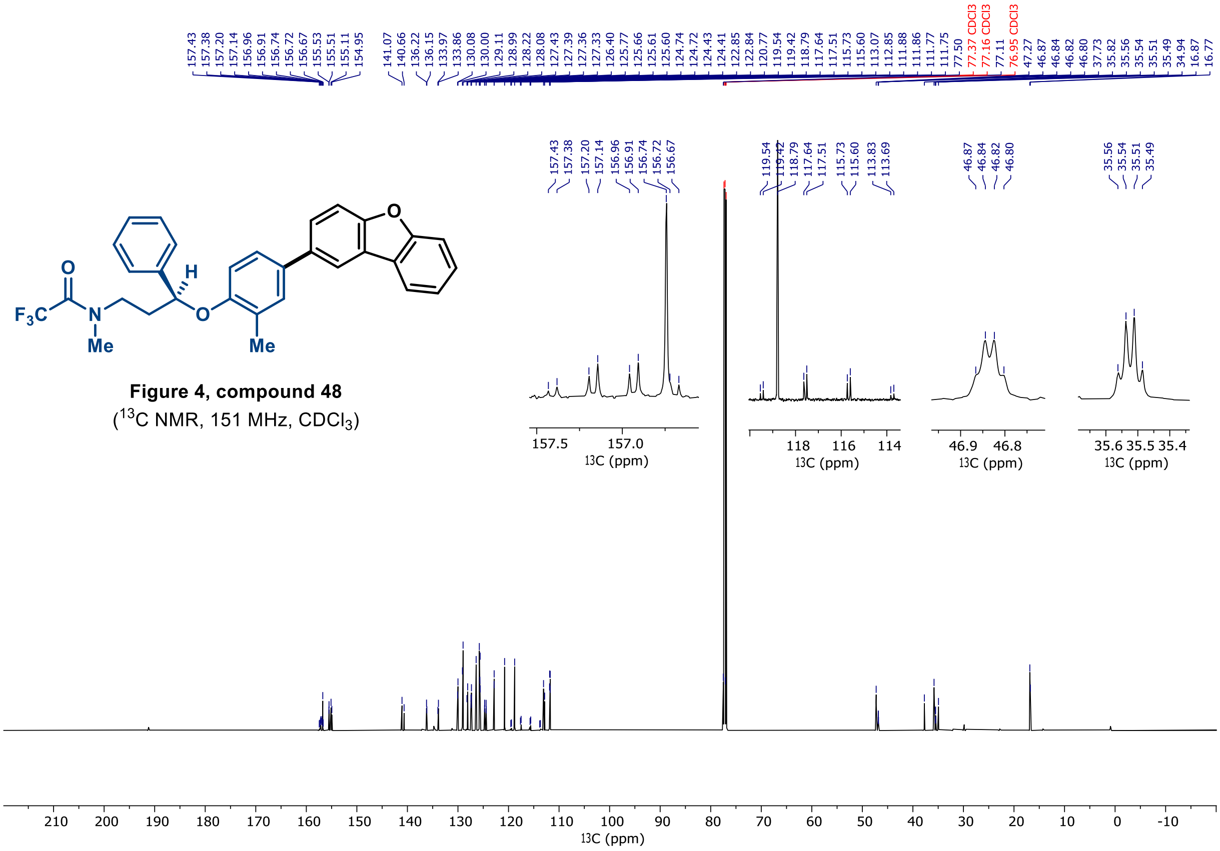

Figure 4, compound 48
(^{13}C NMR, 151 MHz, $CDCl_3$)

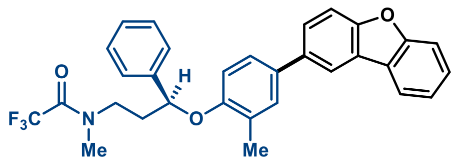

Figure 4, compound 48

(^{19}F NMR, 565 MHz, $CDCl_3$)

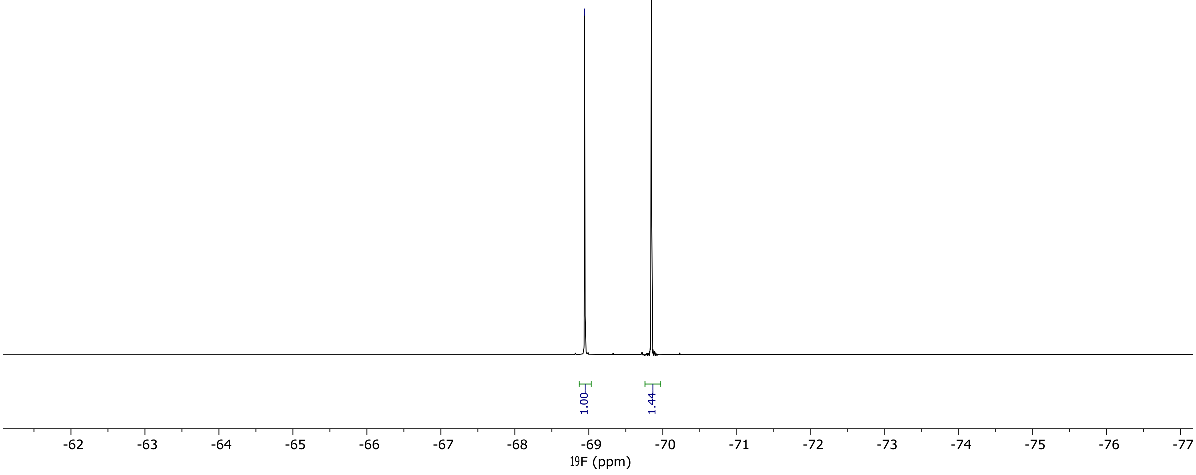

(*S*)-2,2,2-trifluoro-*N*-methyl-*N*-(3-(2-methyl-4-(6-(trifluoromethyl)pyridin-3-yl)phenoxy)-3-phenylpropyl)acetamide (compound 49)

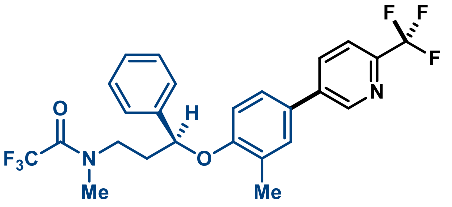

Figure 4, compound 49
(^{1}H NMR, 600 MHz, $CDCl_3$)

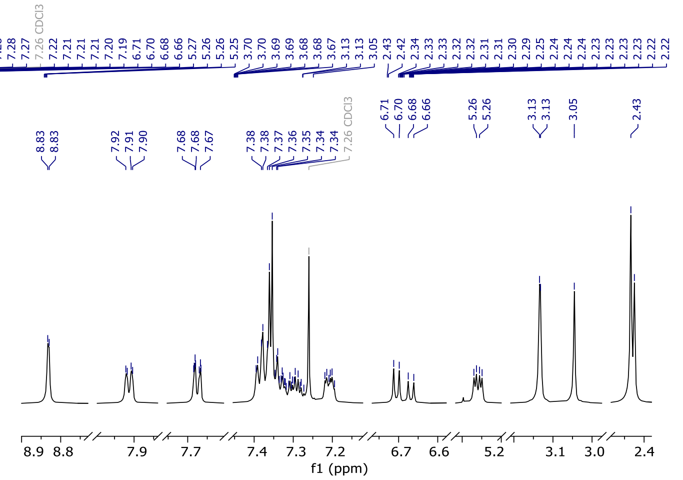

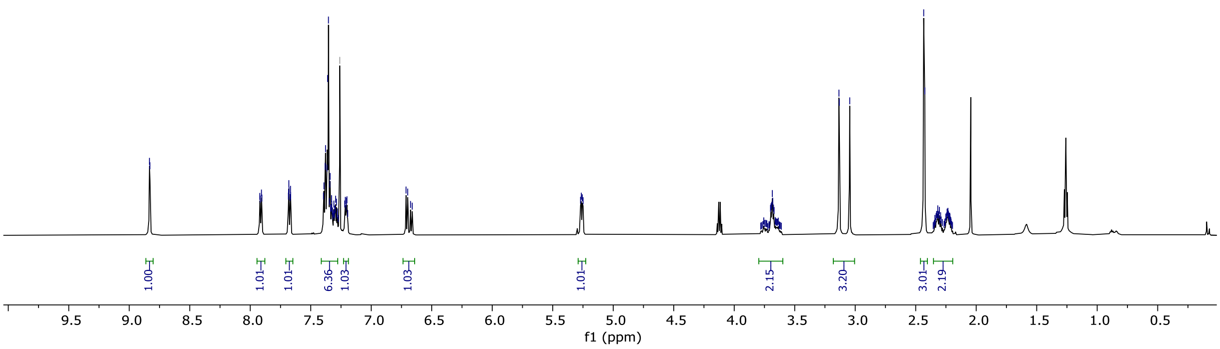

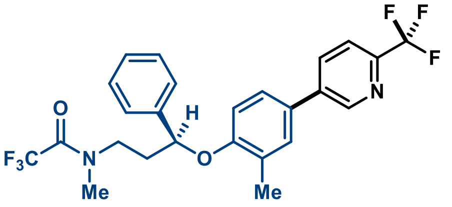

Figure 4, compound 49
(^{13}C NMR, 151 MHz, $CDCl_3$)

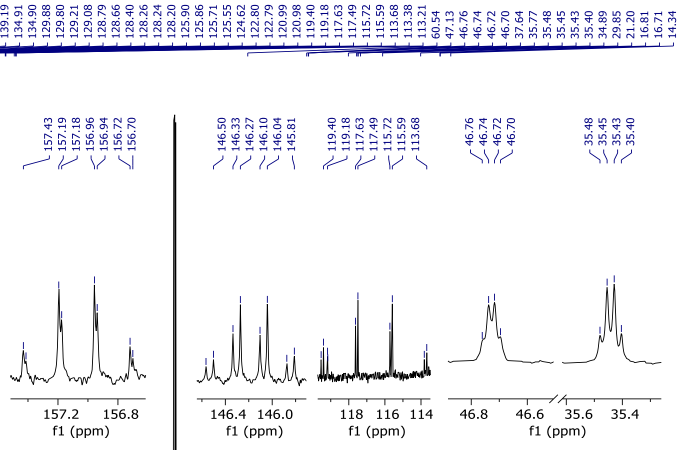

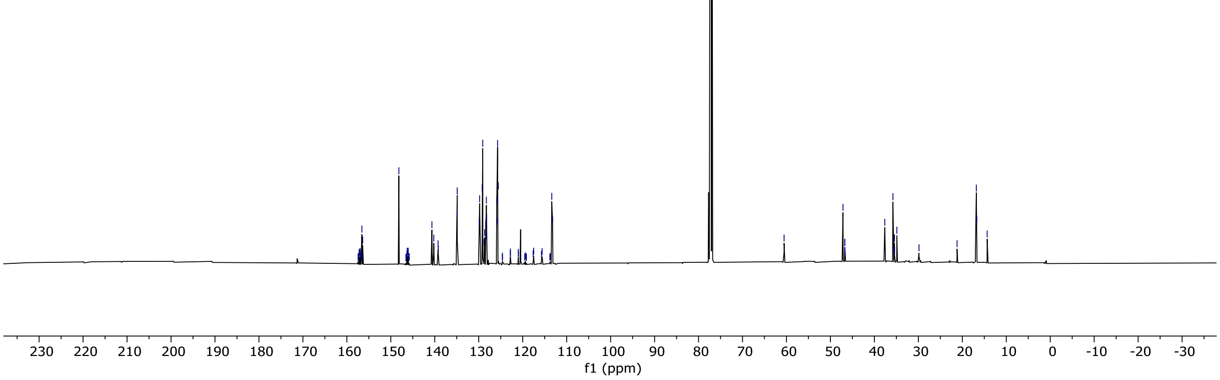

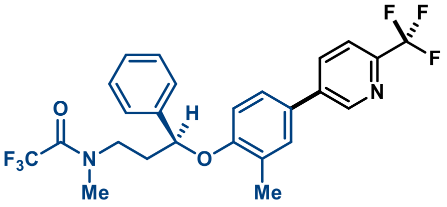

Figure 4, compound 49

(^{19}F NMR, 565 MHz, $CDCl_3$)

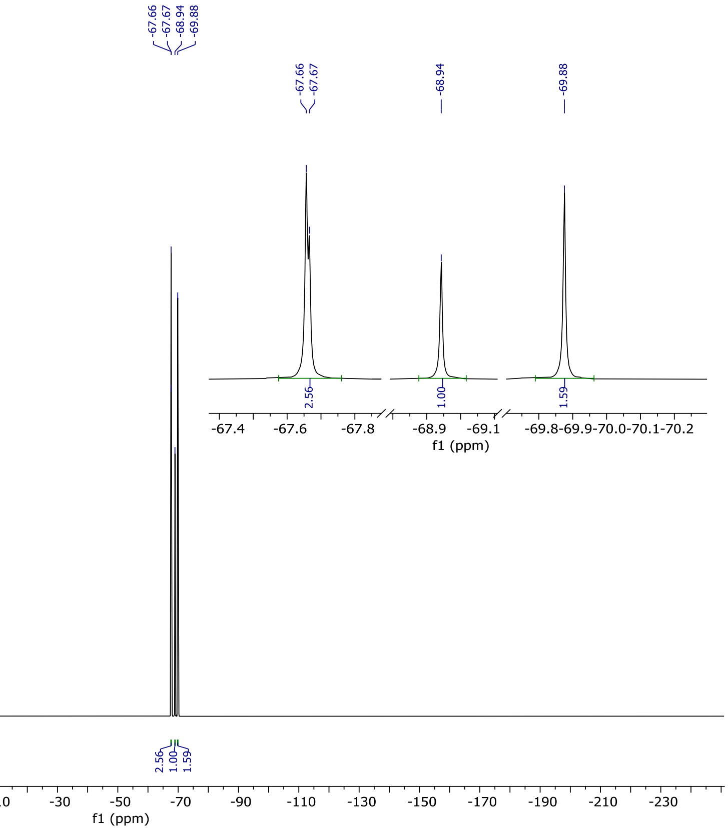

(*S*)-*N*-(3-(4-(2-bromopyridin-4-yl)-2-methylphenoxy)-3-phenylpropyl)-2,2,2-trifluoro-*N*-methylacetamide (compound 50)

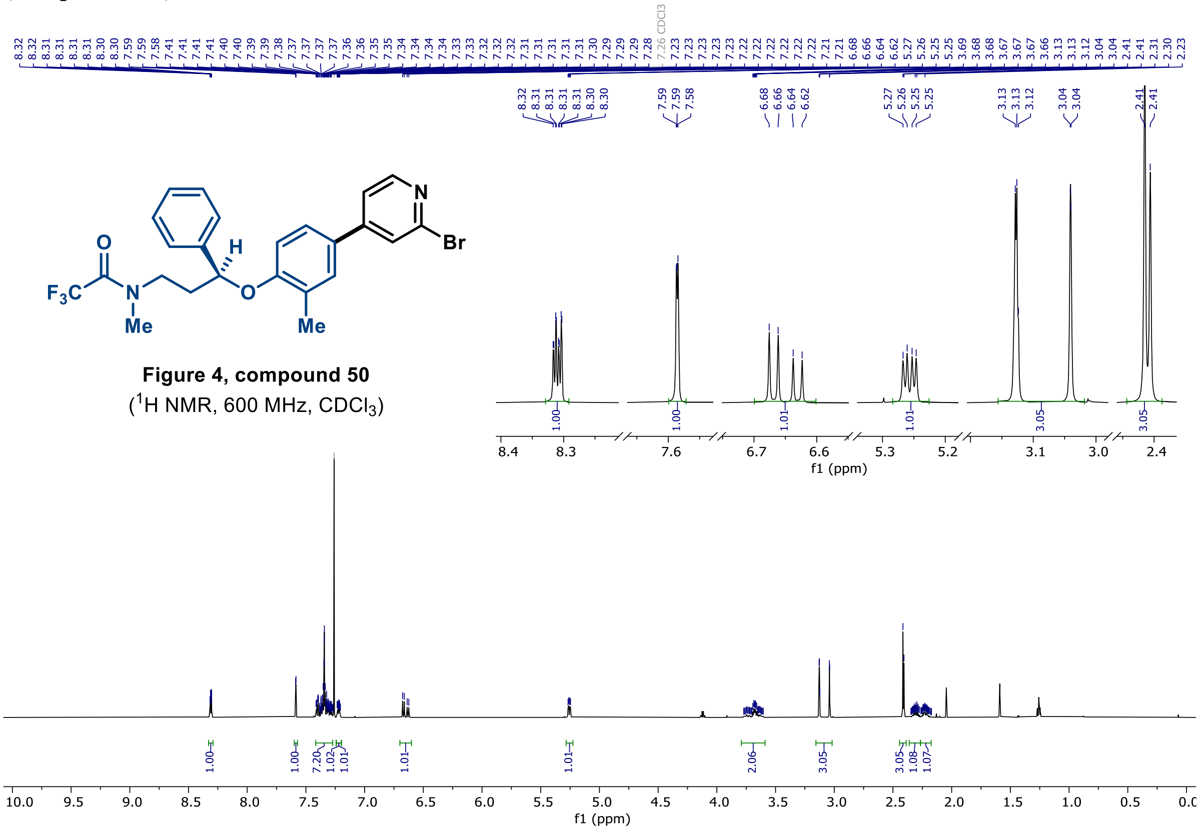

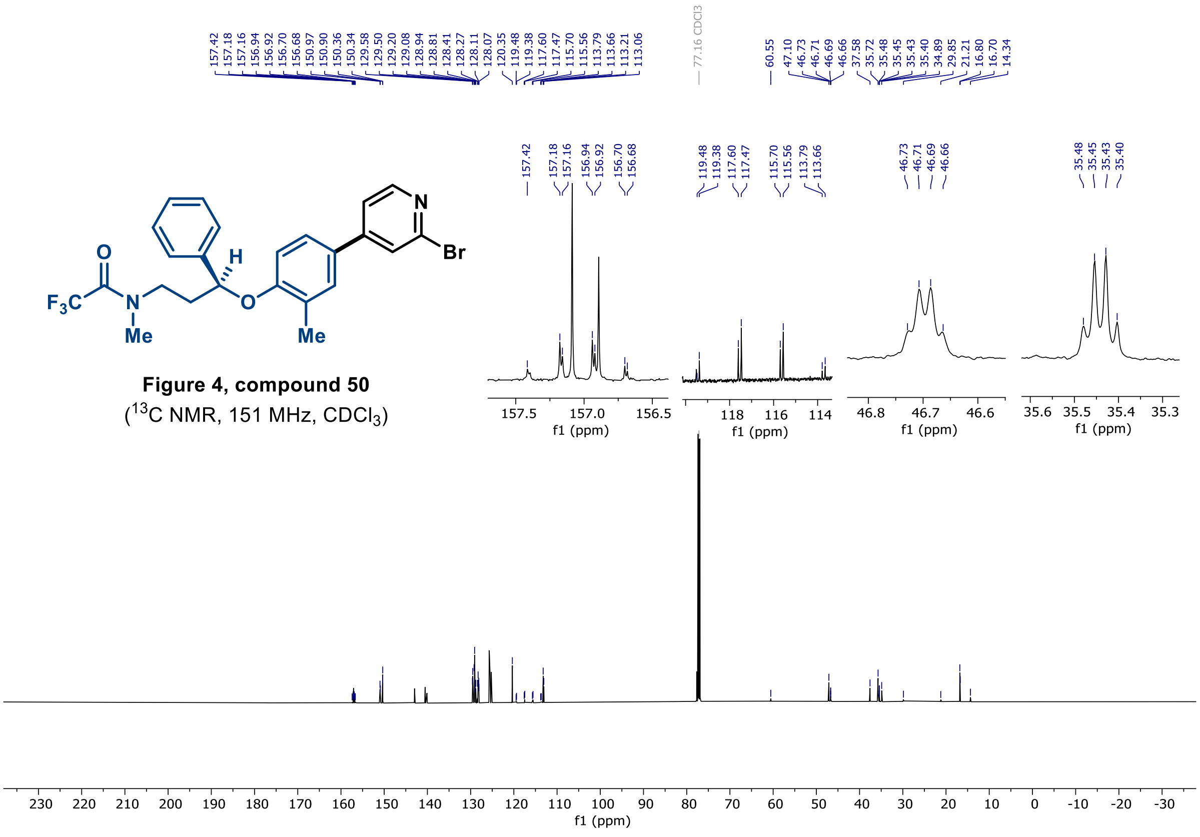

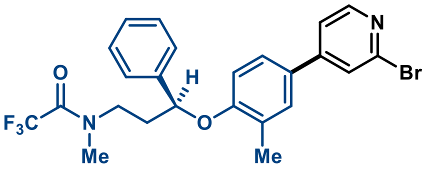

Figure 4, compound 50
(^{19}F NMR, 565 MHz, $CDCl_3$)

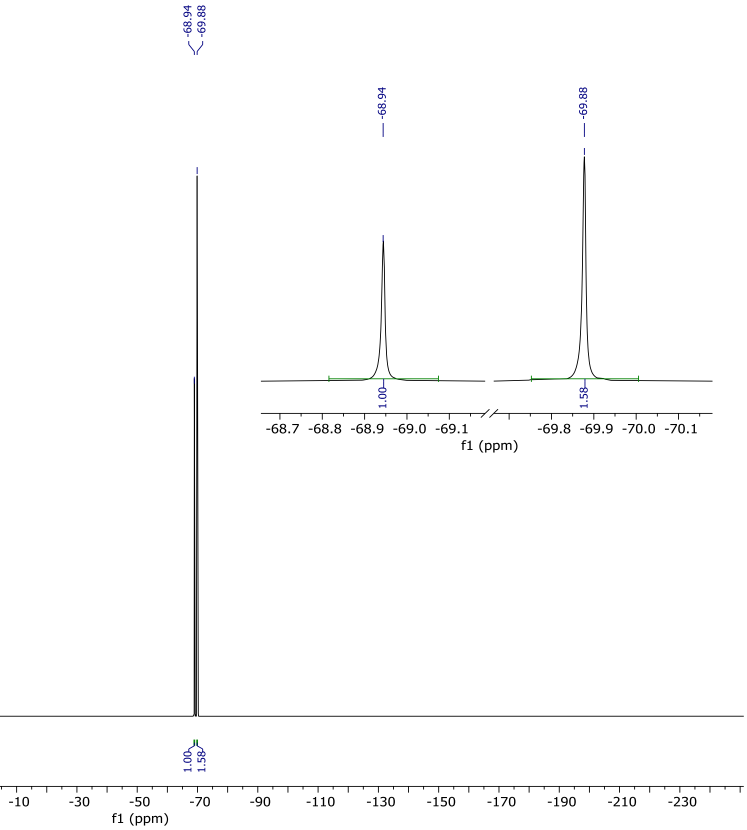

(*S*)-2,2,2-trifluoro-*N*-methyl-*N*-(3-((3-methyl-4'-(trifluoromethyl)-[1,1'-biphenyl]-4-yl)oxy)-3-phenylpropyl)acetamide (compound 51)

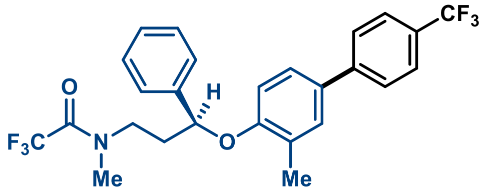

Figure 4, compound 51
(^{1}H NMR, 600 MHz, $CDCl_3$)

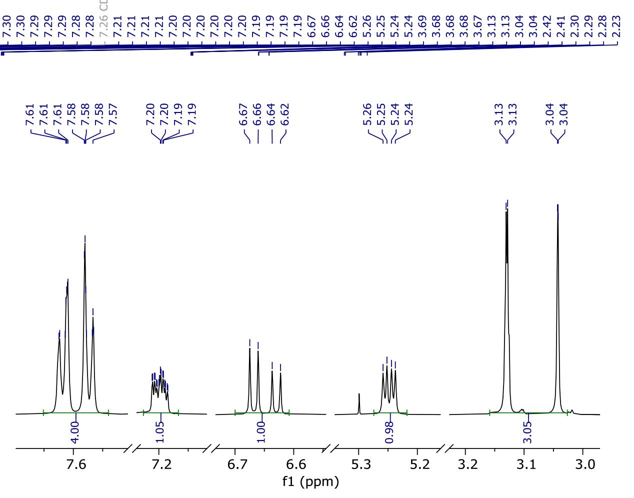

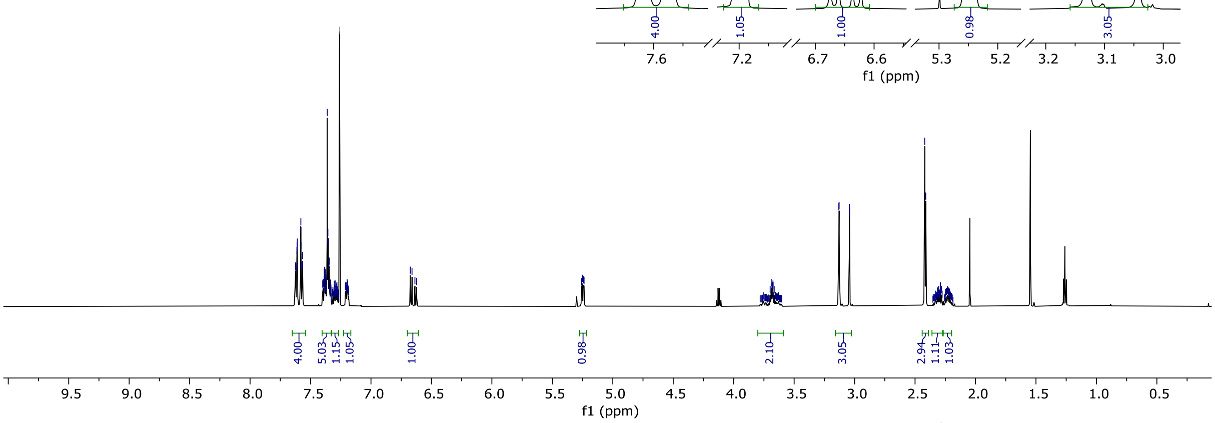

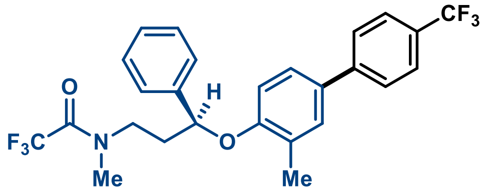

Figure 4, compound 51
(^{13}C NMR, 151 MHz, $CDCl_3$)

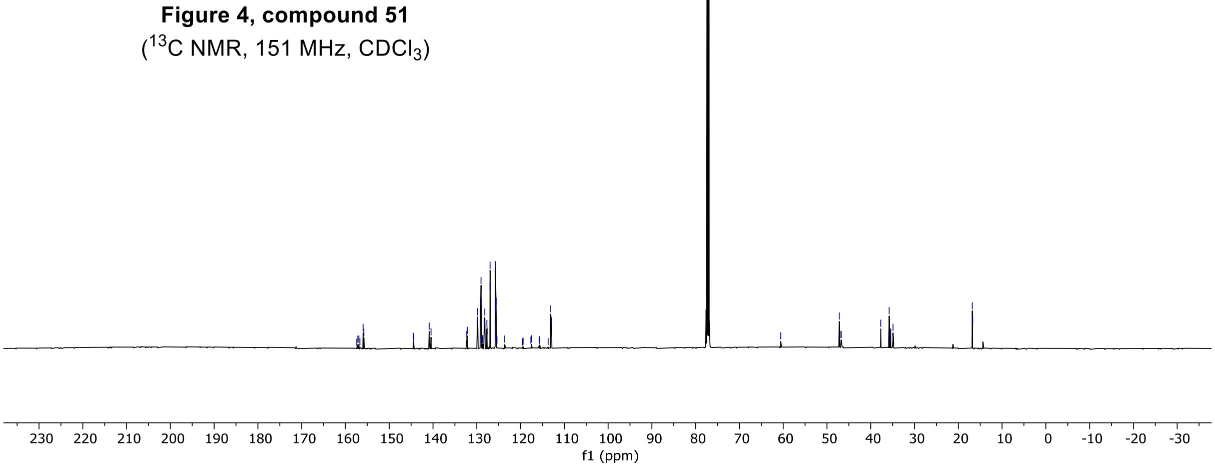

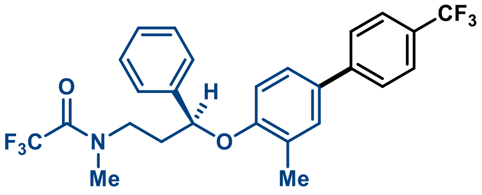

Figure 4, compound 51
(^{19}F NMR, 565 MHz, $CDCl_3$)

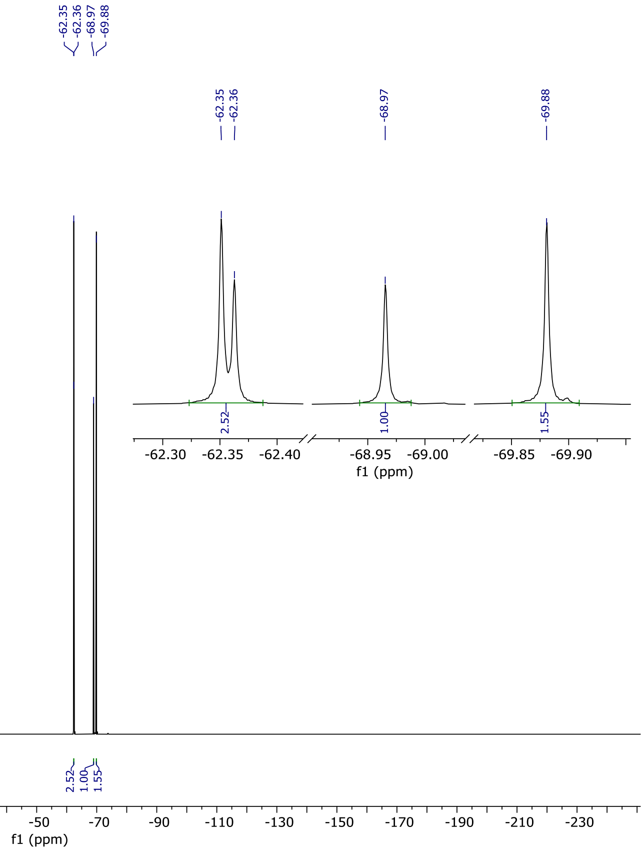

4-methoxy-4'-(trifluoromethyl)-1,1'-biphenyl

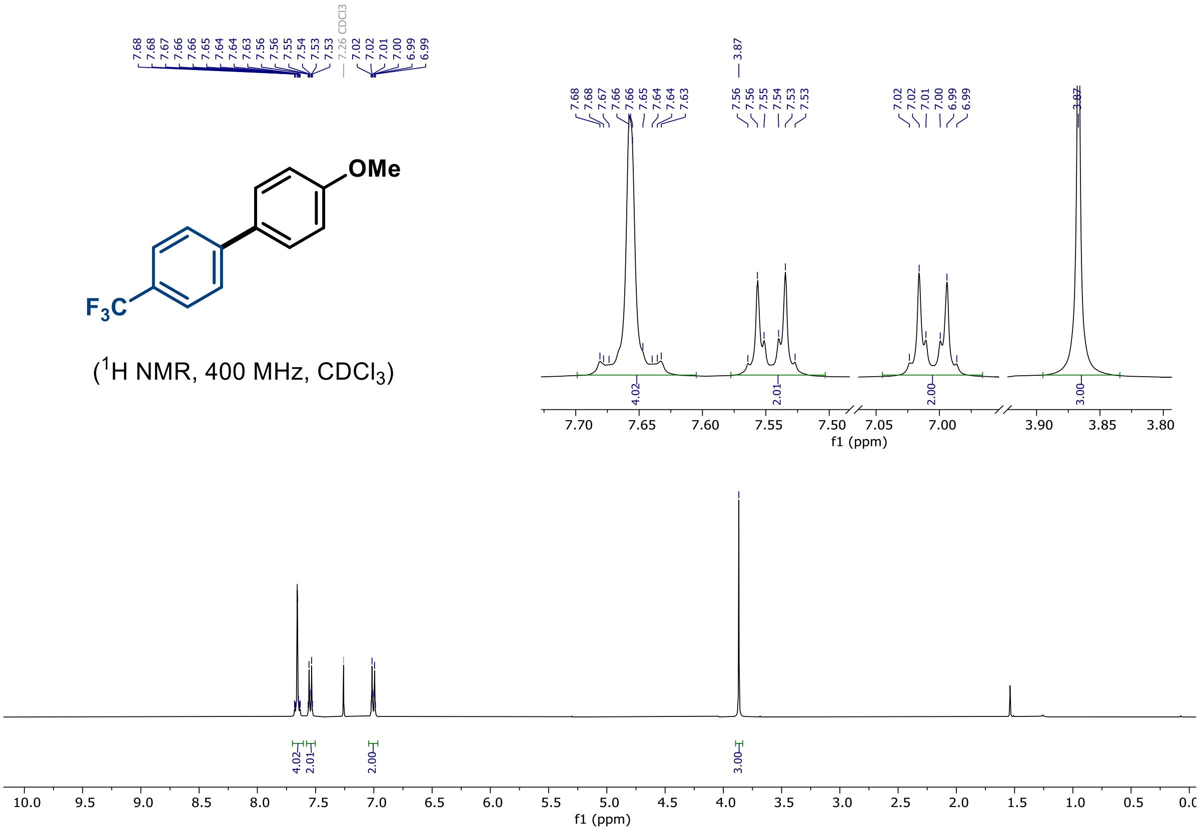

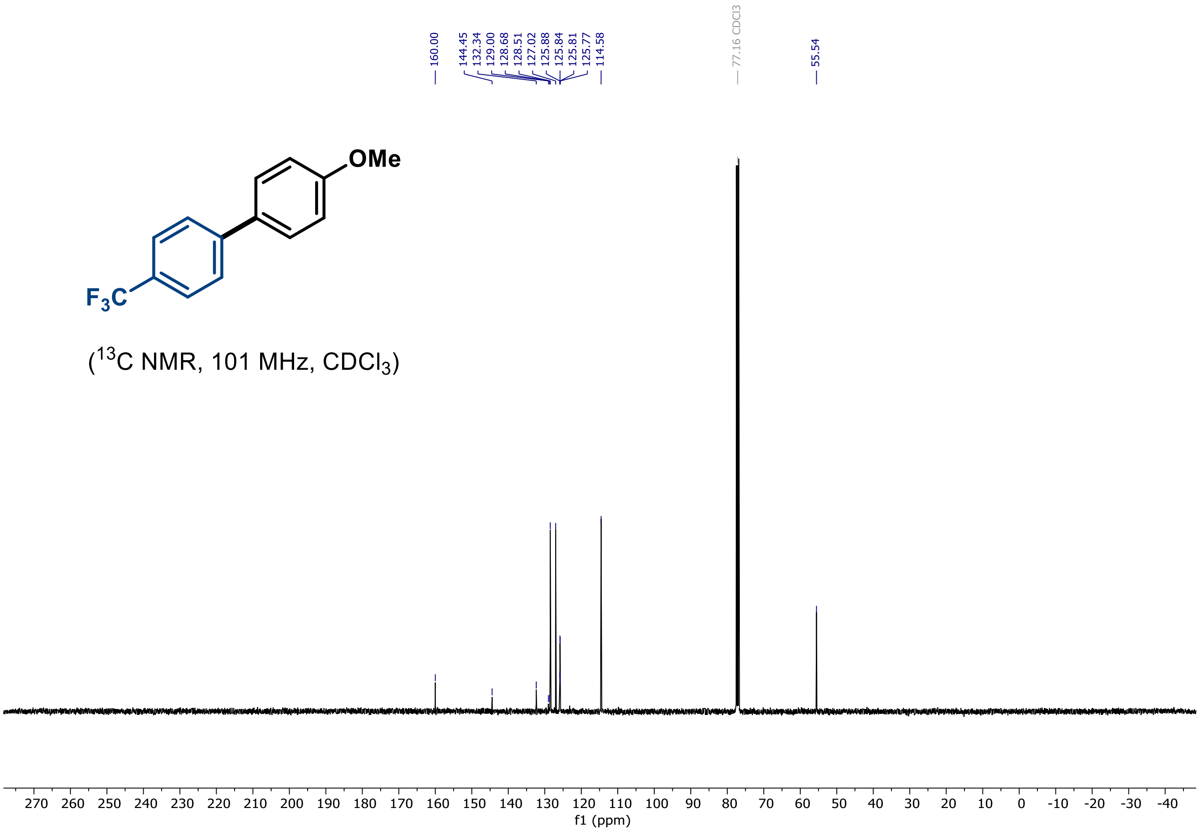

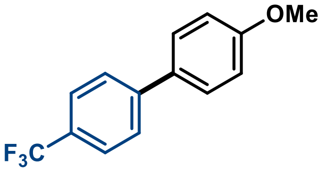

(${}^{19}F$ NMR, 376 MHz, $CDCl_3$)

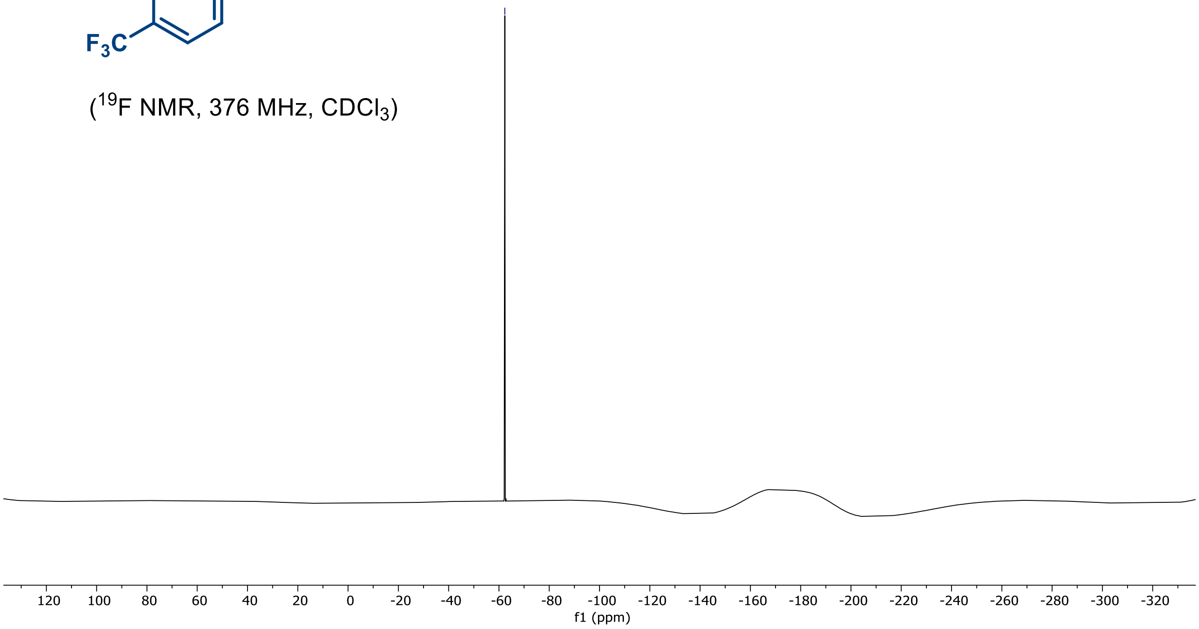

(*S*)-2,2,2-trifluoro-*N*-methyl-*N*-(3-(2-methyl-4-(1-tosyl-1,2,3,6-tetrahydropyridin-4-yl)phenoxy)-3-phenylpropyl)acetamide (Figure 4, compound 52)

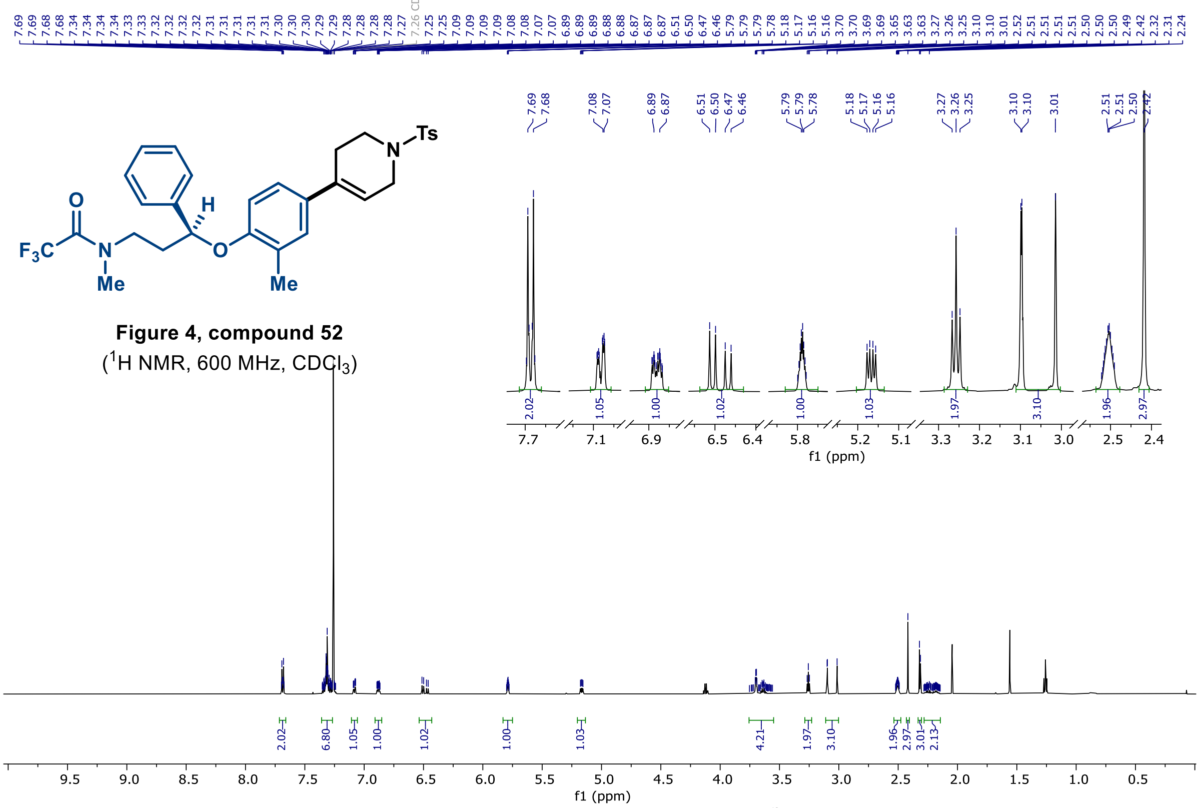

Figure 4, compound 52

(^{1}H NMR, 600 MHz, $CDCl_3$)

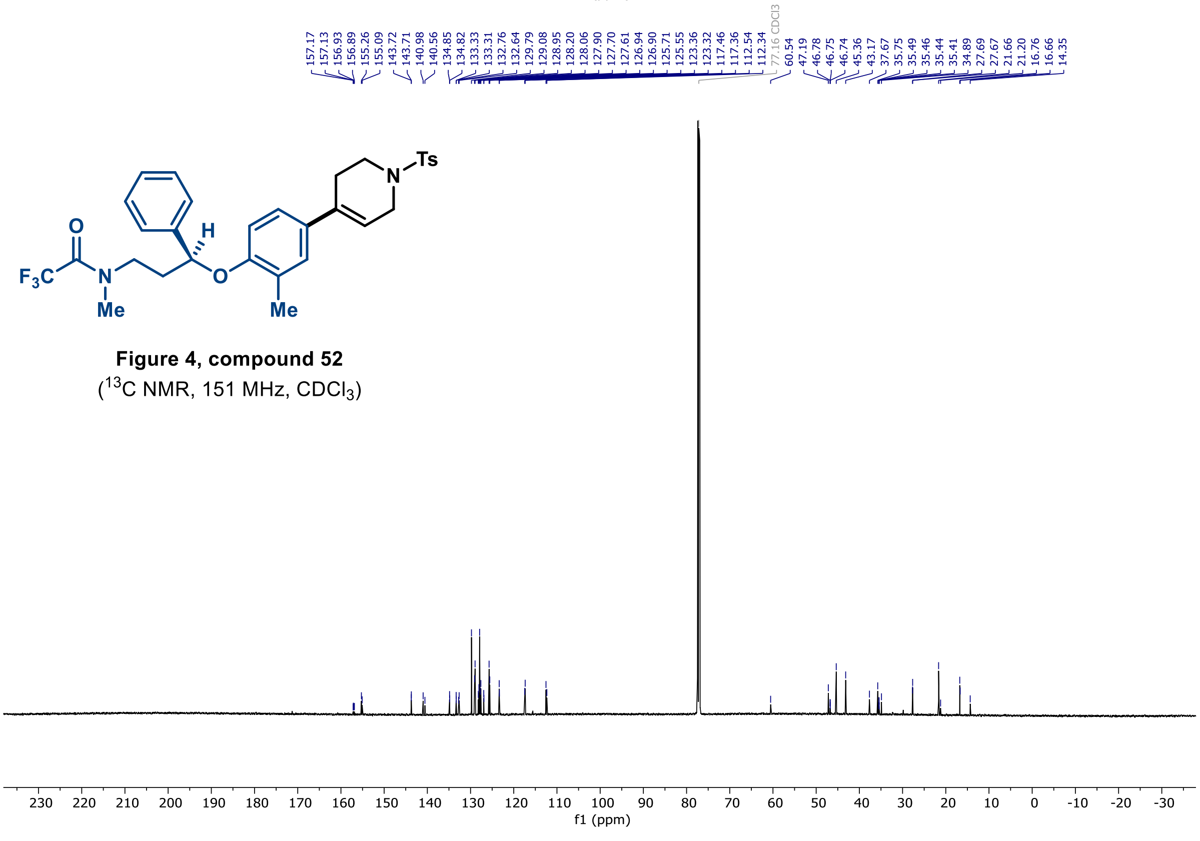

Figure 4, compound 52

(^{13}C NMR, 151 MHz, $CDCl_3$)

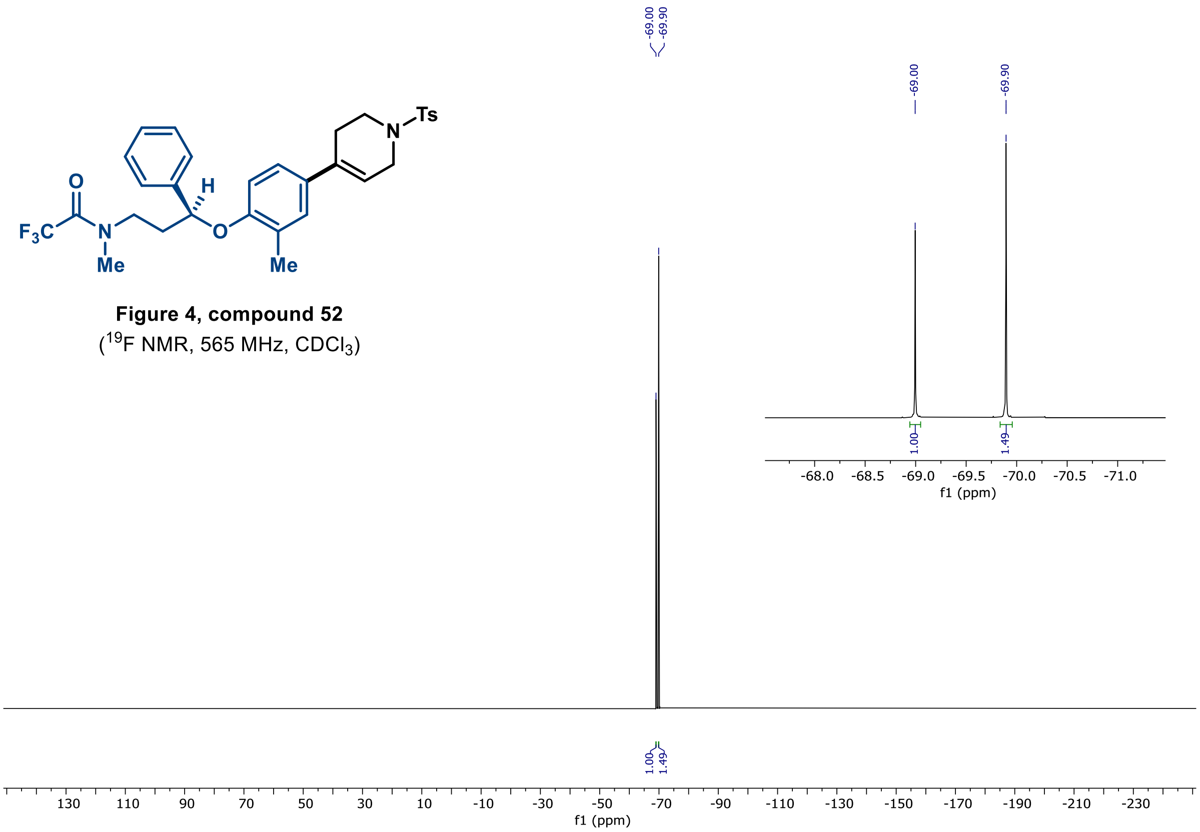

Figure 4, compound 52

(^{19}F NMR, 565 MHz, $CDCl_3$)

(*S*)-*N*-(3-(4-(Benzo[b]thiophen-3-yl)-2-methylphenoxy)-3-phenylpropyl)-2,2,2-trifluoro-*N*-methylacetamide (compound 53)

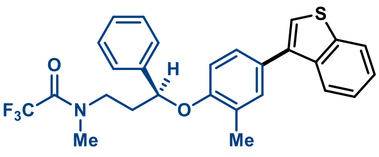

Figure 4, compound 53
(^{1}H NMR, 400 MHz, $CDCl_3$)

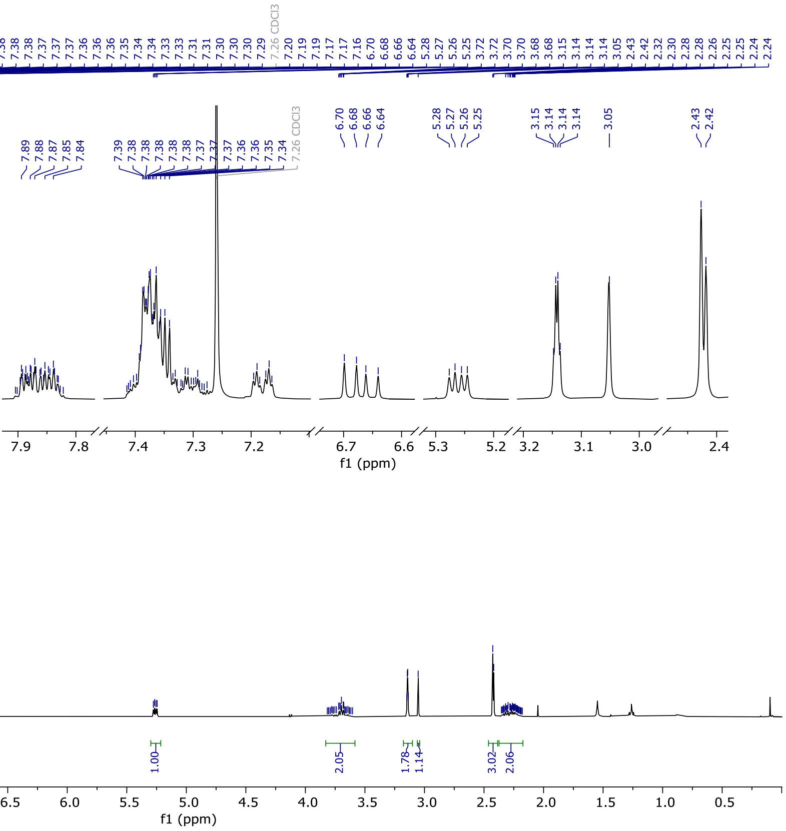

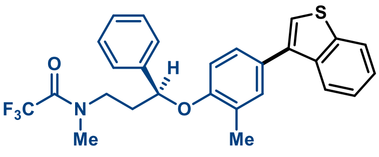

Figure 4, compound 53
(^{13}C NMR, 151 MHz, $CDCl_3$)

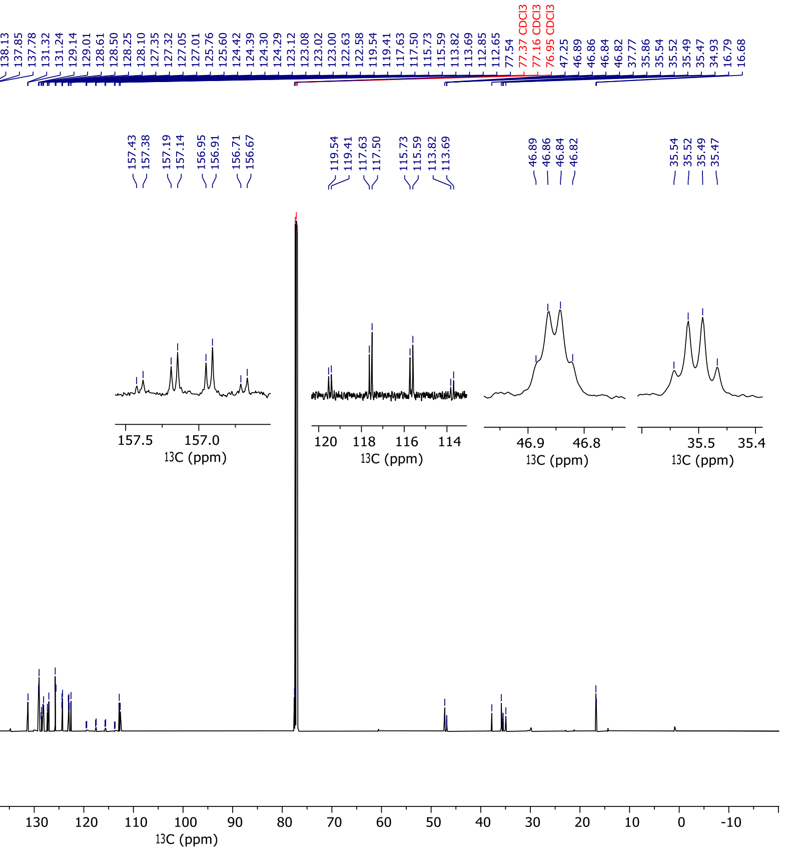

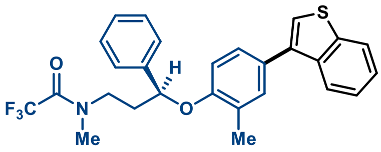

Figure 4, compound 53

(^{19}F NMR, 565 MHz, $CDCl_3$)

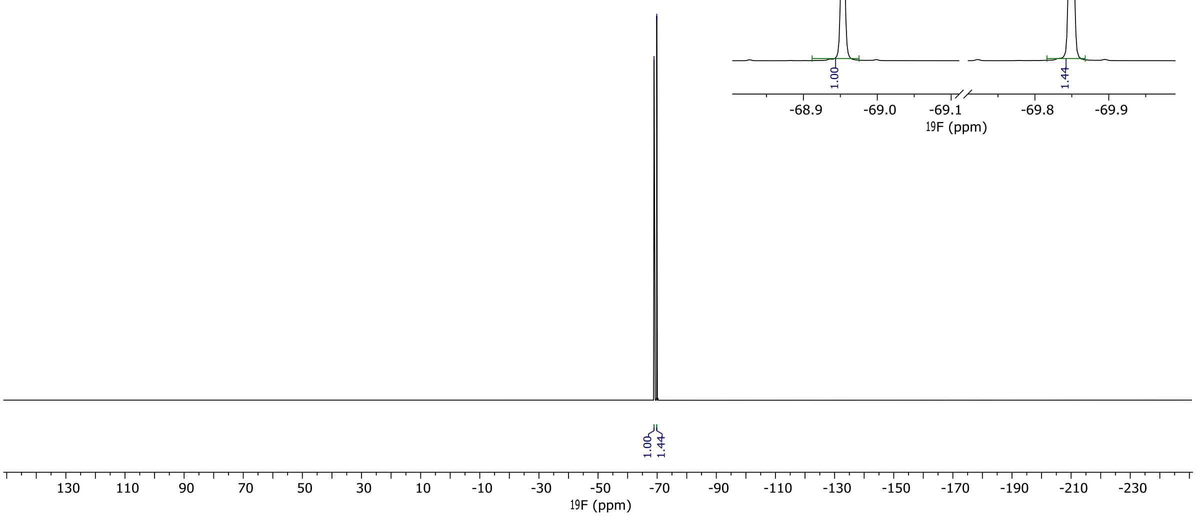

(*S*)-*N*-(3-((3'-cyano-3-methyl-[1,1'-biphenyl]-4-yl)oxy)-3-phenylpropyl)-2,2,2-trifluoro-*N*-methylacetamide (compound 54)

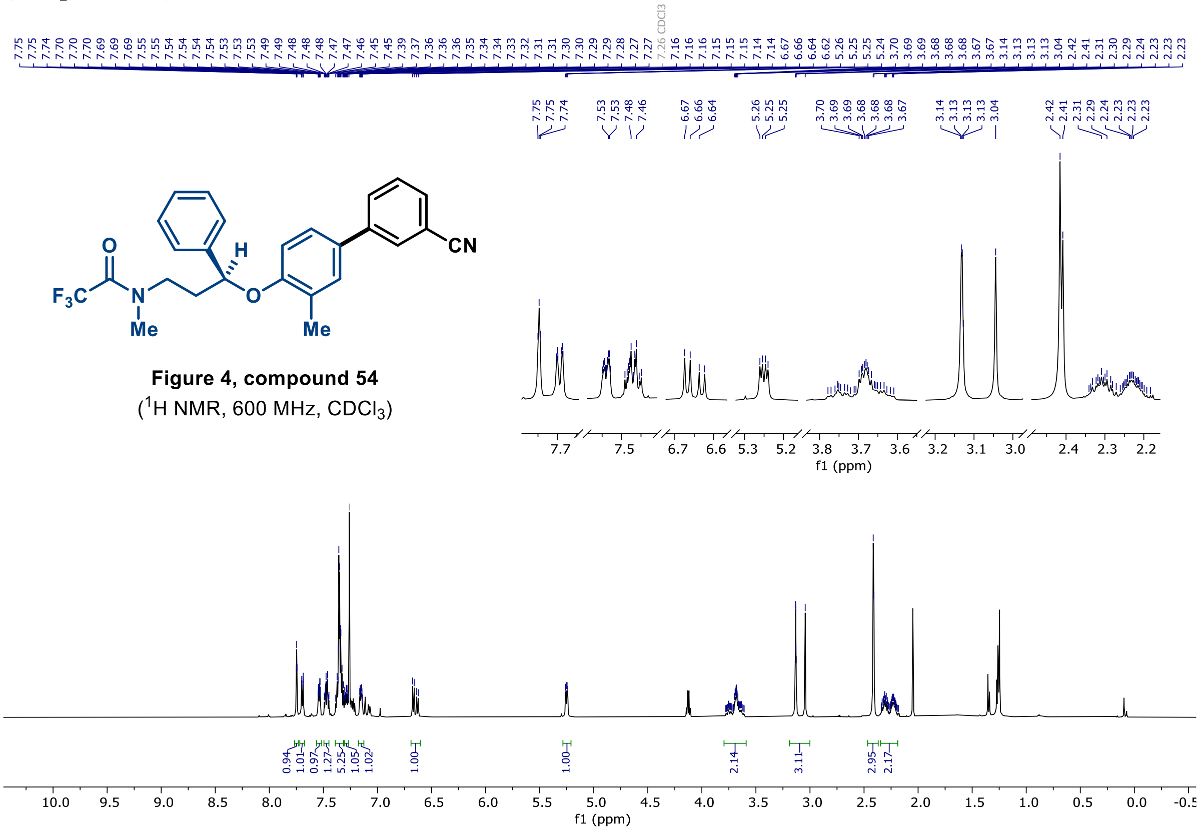

Figure 4, compound 54
(^{1}H NMR, 600 MHz, $CDCl_3$)

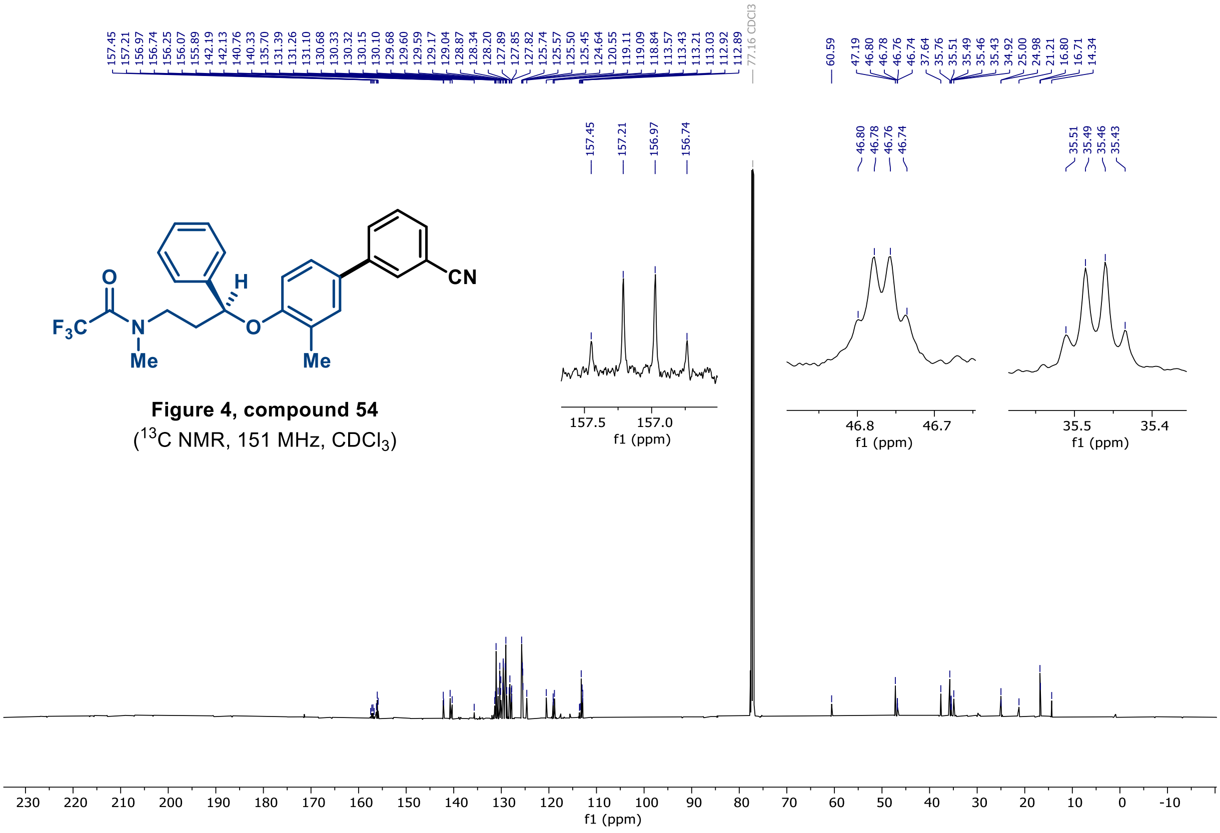

Figure 4, compound 54
(^{13}C NMR, 151 MHz, $CDCl_3$)

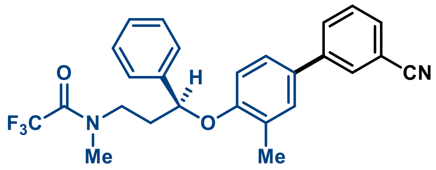

Figure 4, compound 54
(^{19}F NMR, 565 MHz, $CDCl_3$)

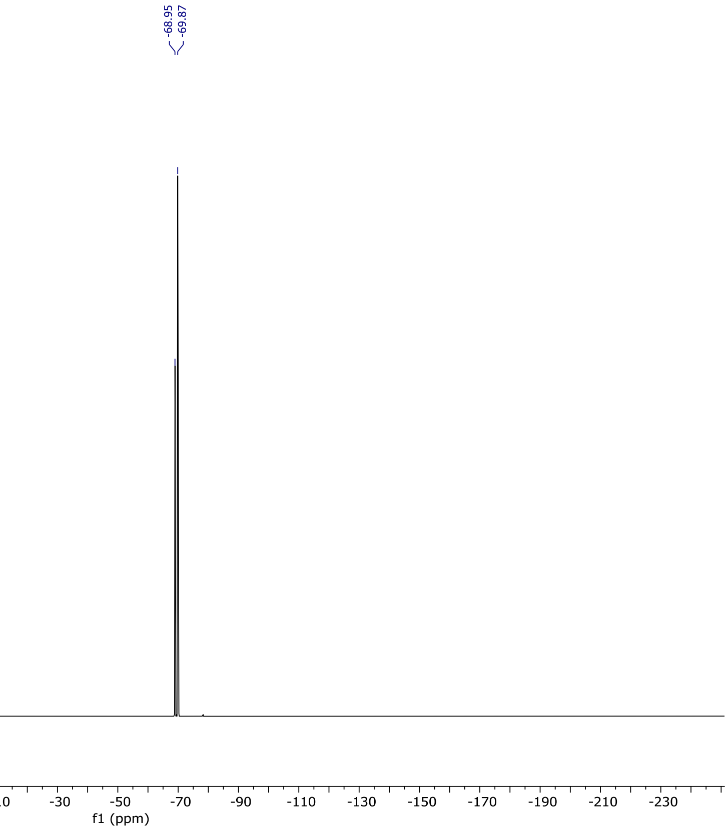

(*S*)-*N*-(3-((3',5'-dimethoxy-3-methyl-[1,1'-biphenyl]-4-yl)oxy)-3-phenylpropyl)-2,2,2-trifluoro-*N*-methylacetamide (compound 55)

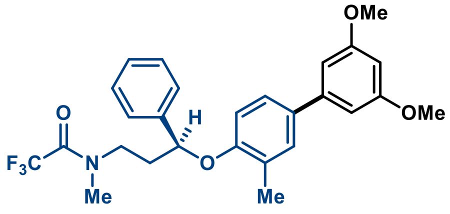

Figure 4, compound 55
(^{1}H NMR, 600 MHz, $CDCl_3$)

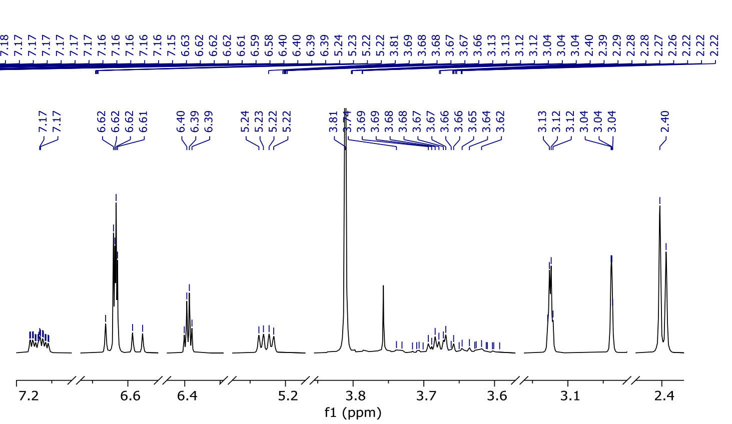

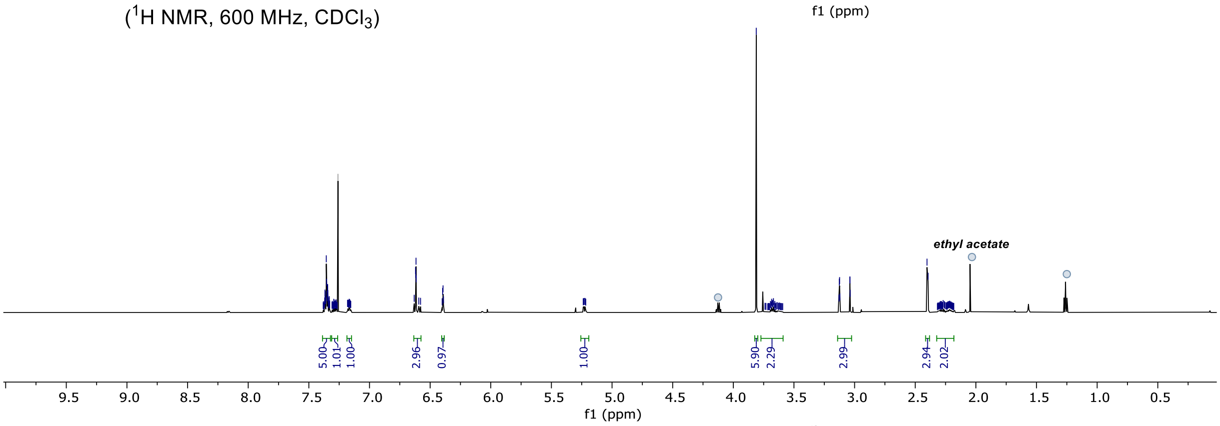

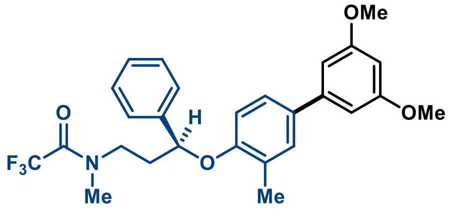

Figure 4, compound 55
(^{13}C NMR, 151 MHz, $CDCl_3$)

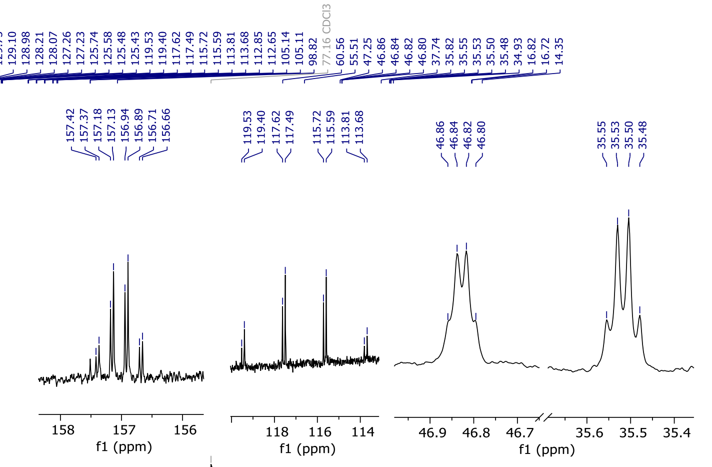

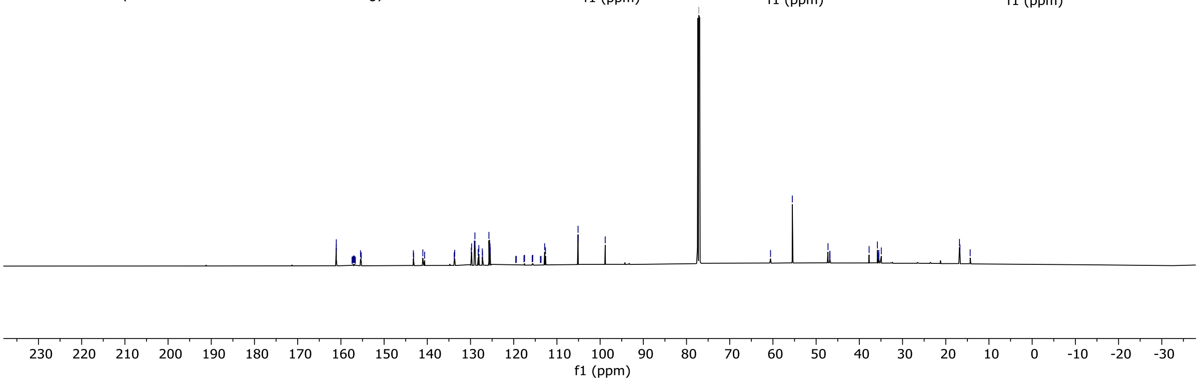

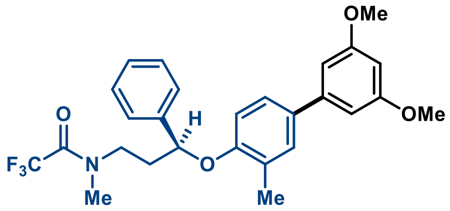

Figure 4, compound 55
(^{19}F NMR, 565 MHz, $CDCl_3$)

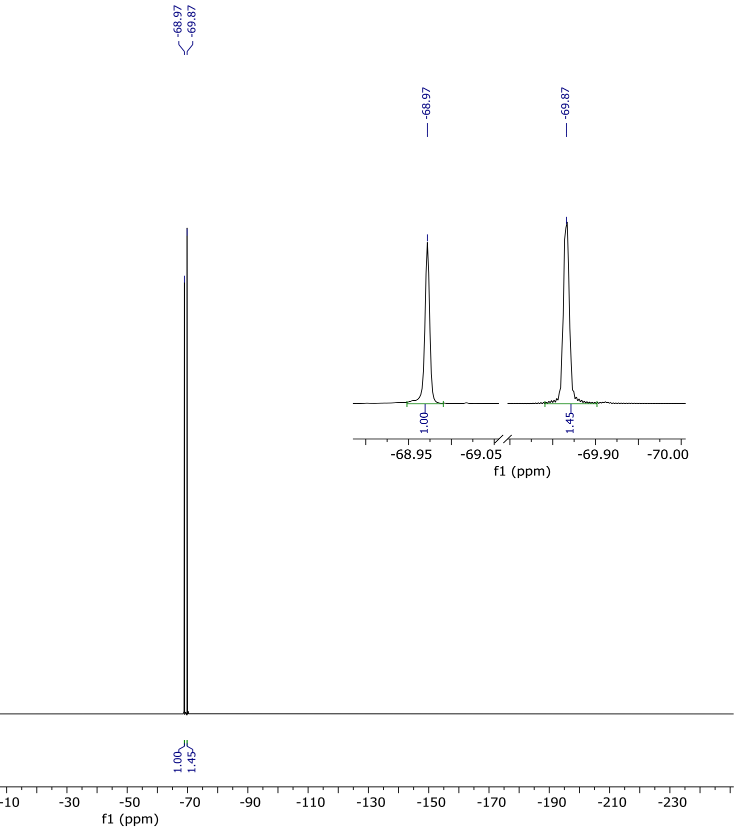

***tert*-Butyl 2-phenyl-1H-pyrrole-1-carboxylate (compound 56)**

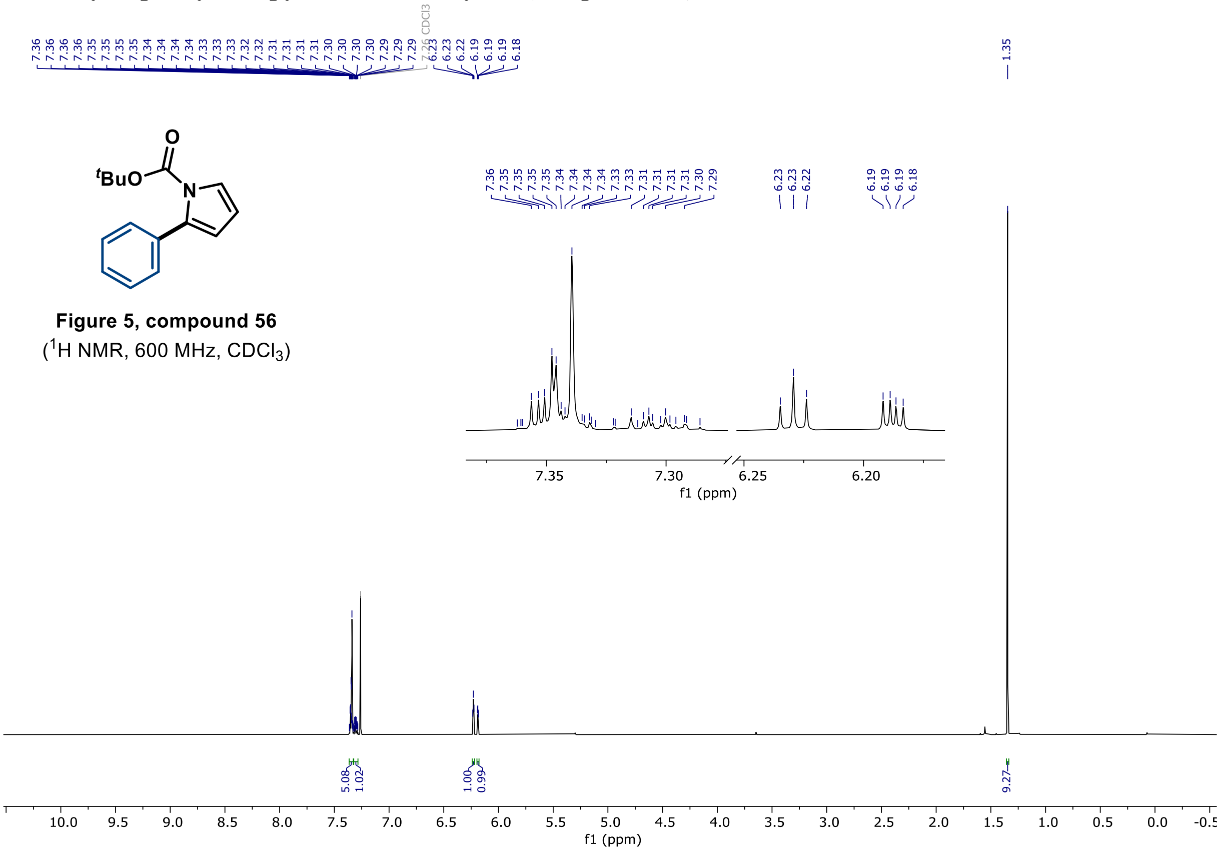

Figure 5, compound 56
(^{1}H NMR, 600 MHz, $CDCl_3$)

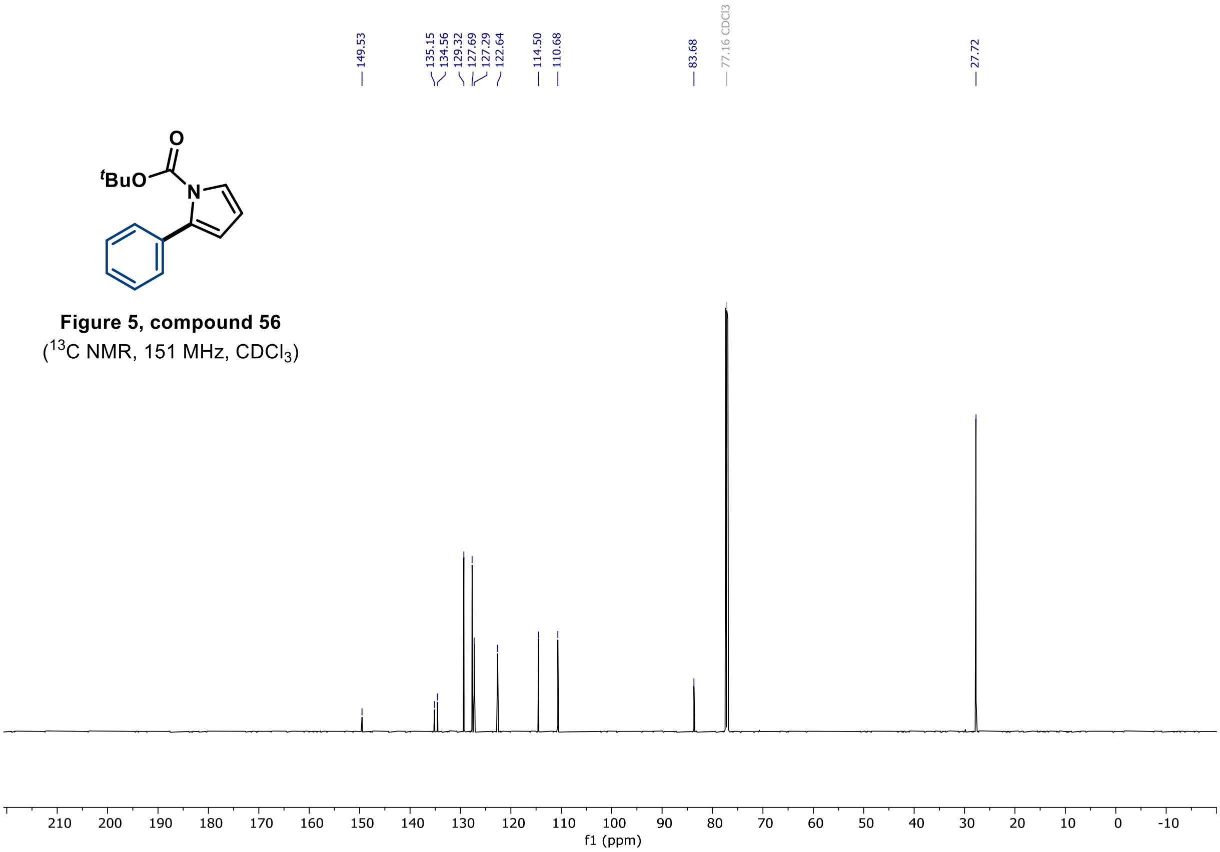

Figure 5, compound 56
(^{13}C NMR, 151 MHz, $CDCl_3$)

2-Phenylfuran (compound 57)

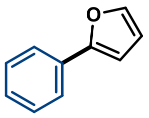

Figure 5, compound 57
(^{1}H NMR, 600 MHz, $CDCl_3$)

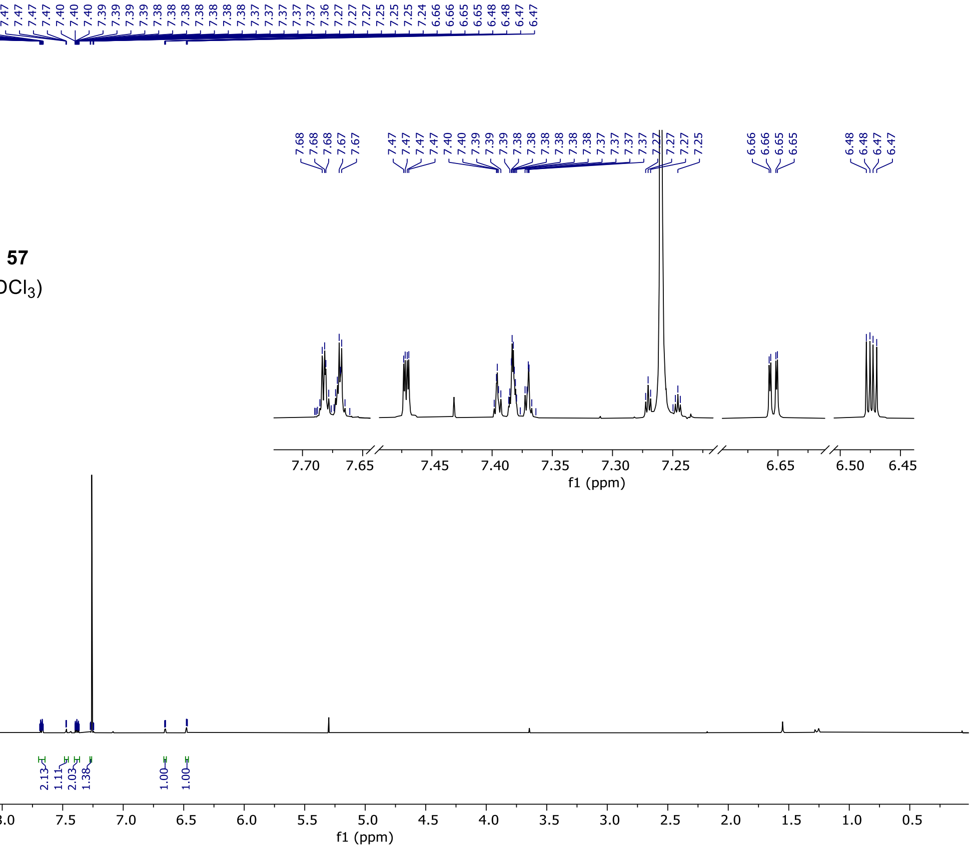

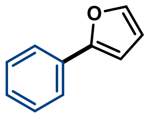

Figure 5, compound 57
(^{13}C NMR, 151 MHz, $CDCl_3$)

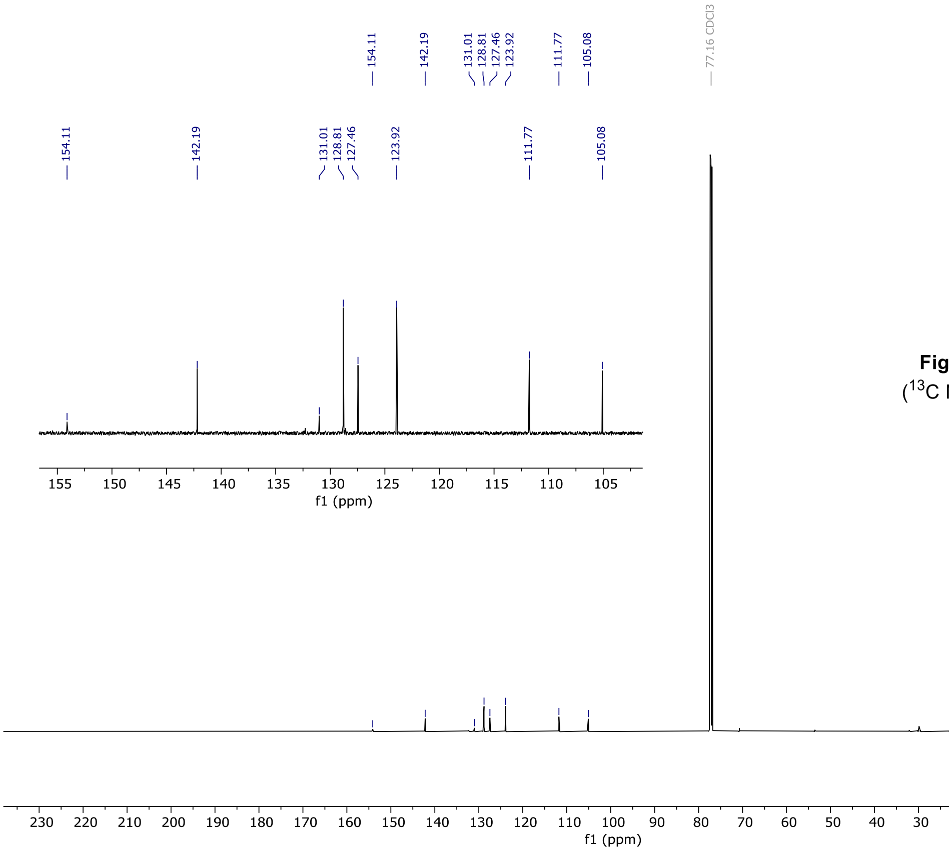

4-Methoxy-1,1'-biphenyl (compound 58)

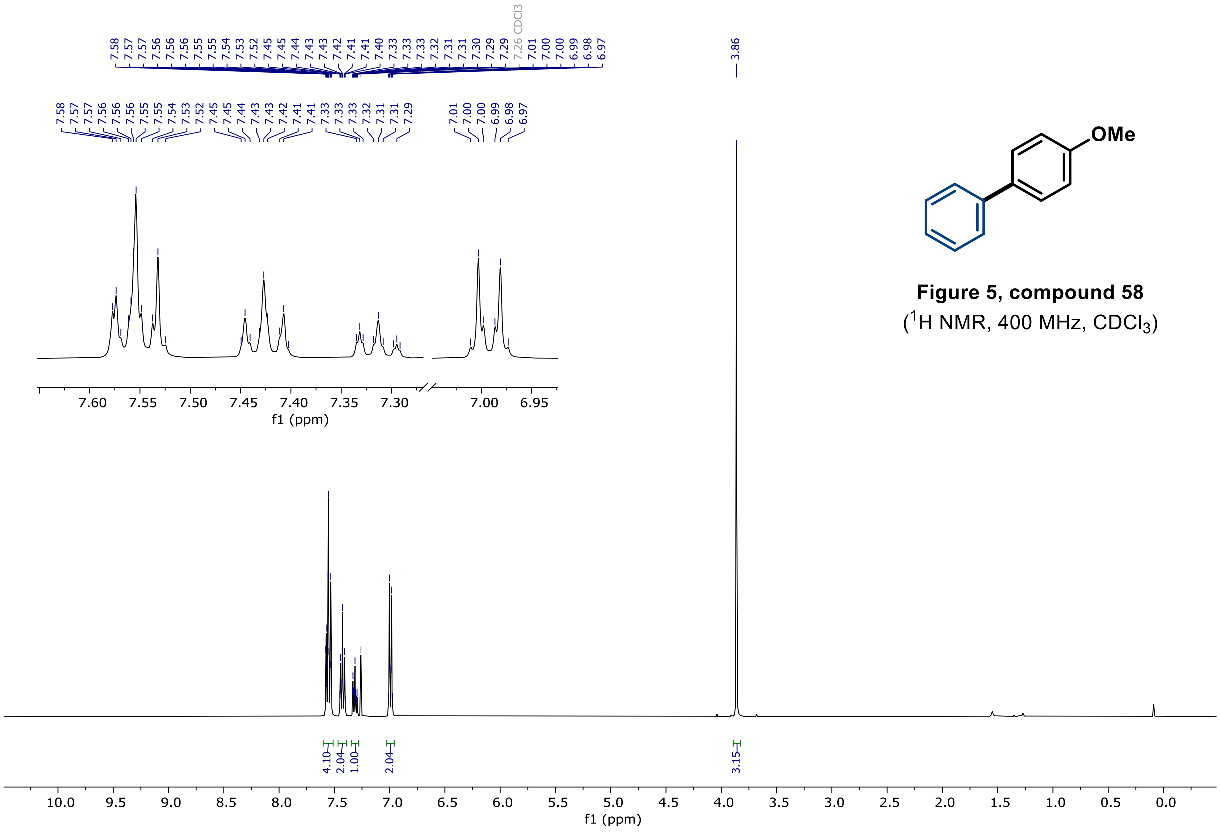

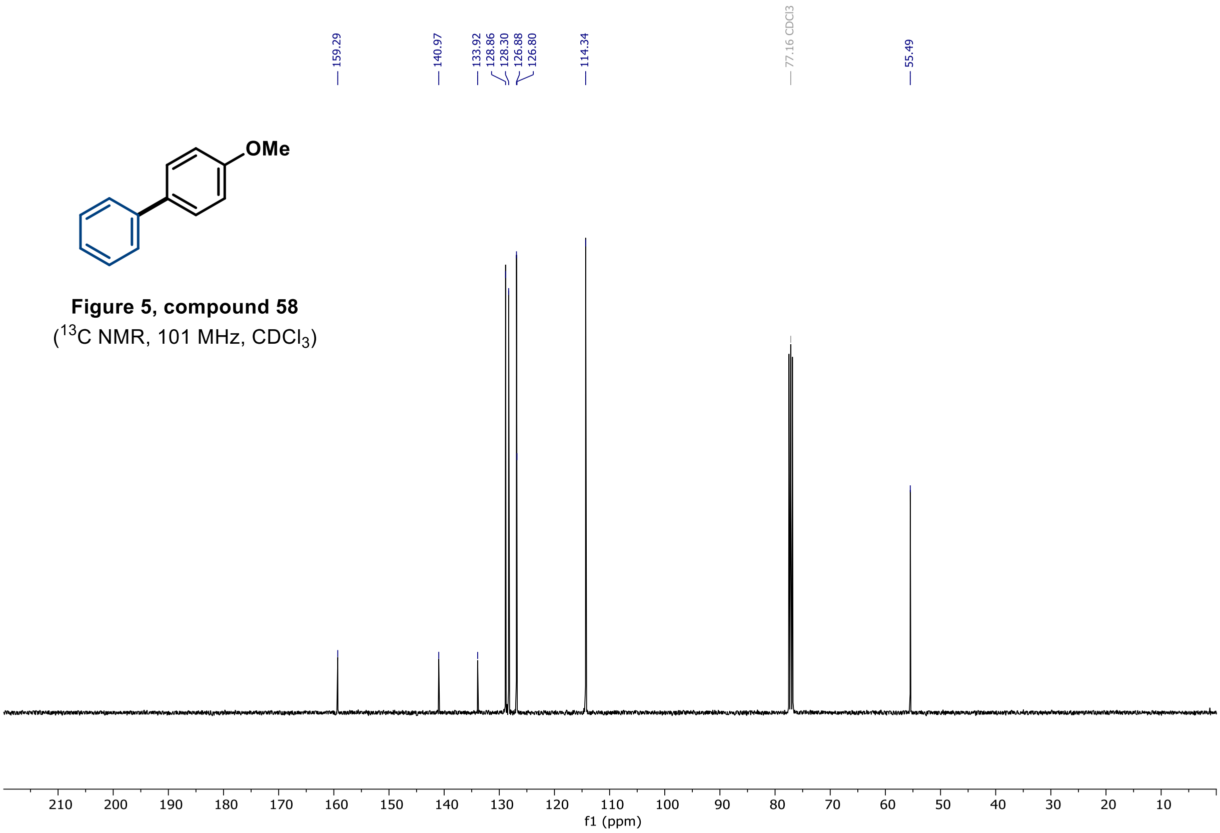

4'-Methoxy-3,5-bis(trifluoromethyl)-1,1'-biphenyl (compound 59)

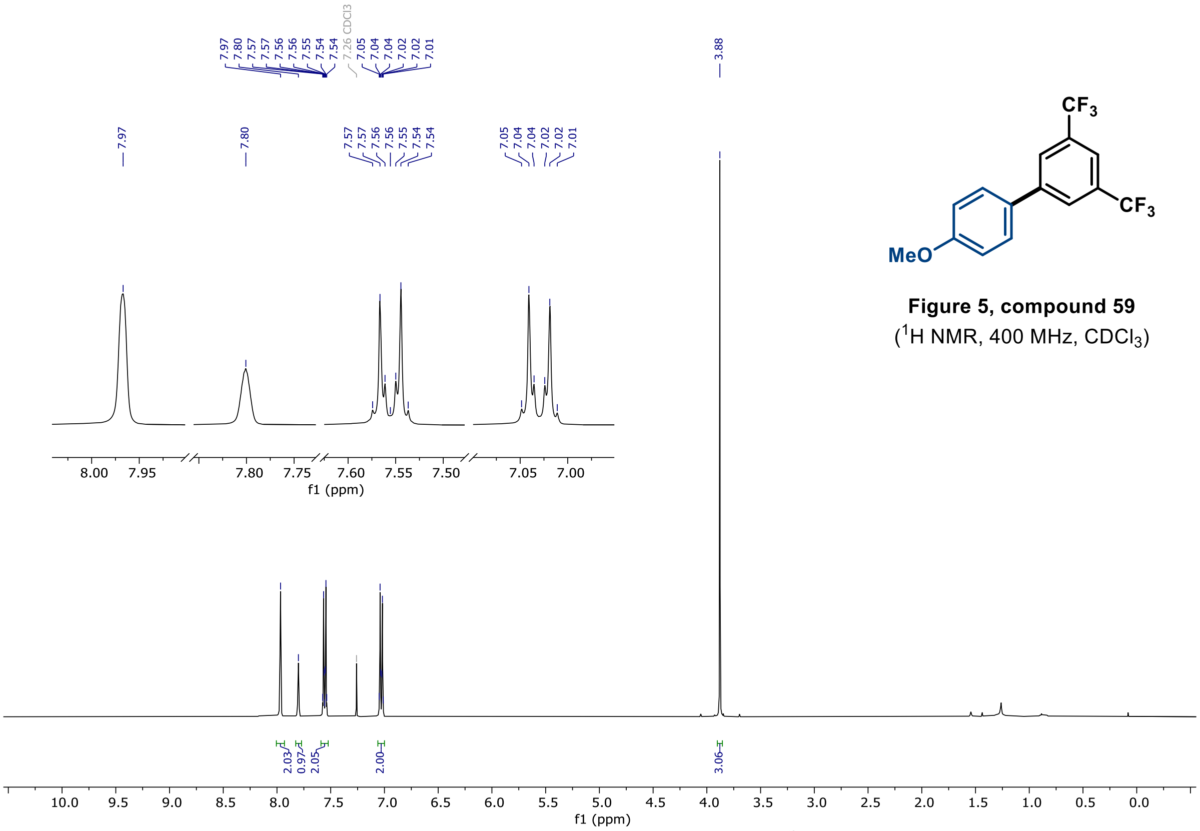

Figure 5, compound 59
(^{1}H NMR, 400 MHz, $CDCl_3$)

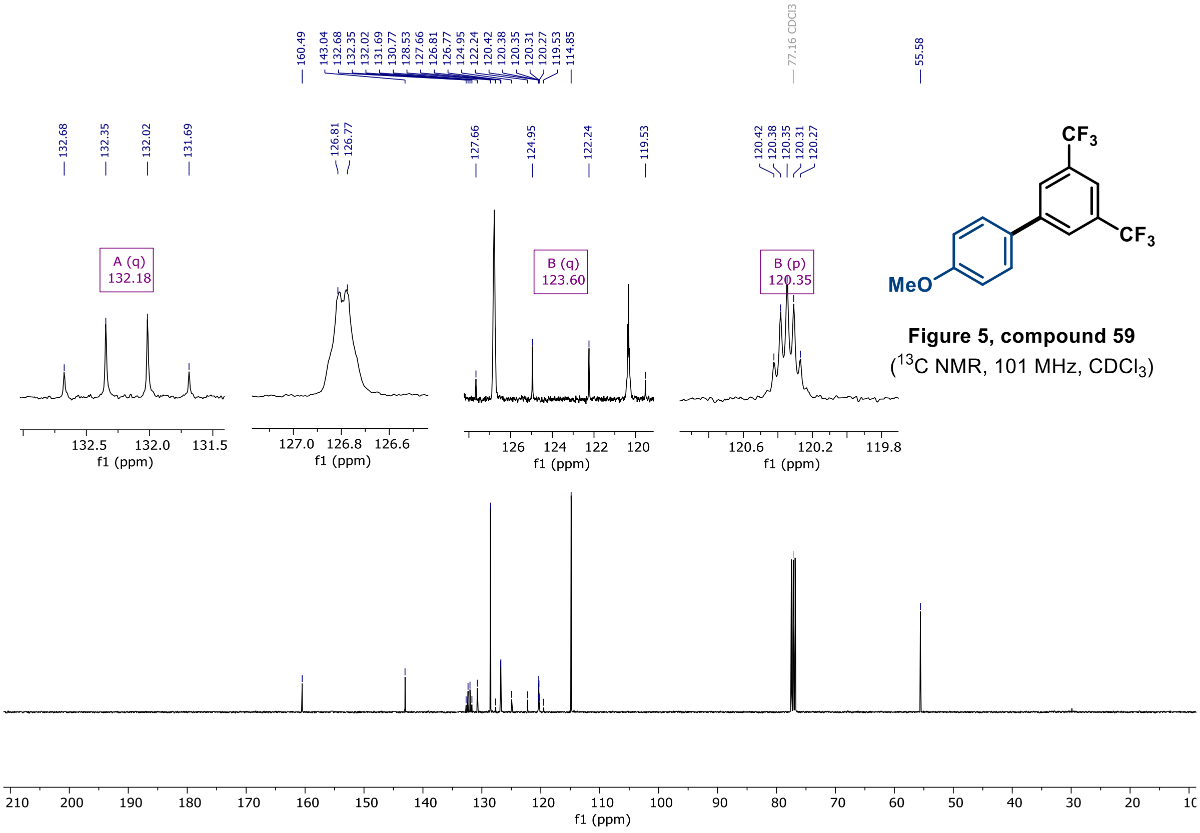

Figure 5, compound 59
(^{13}C NMR, 101 MHz, $CDCl_3$)

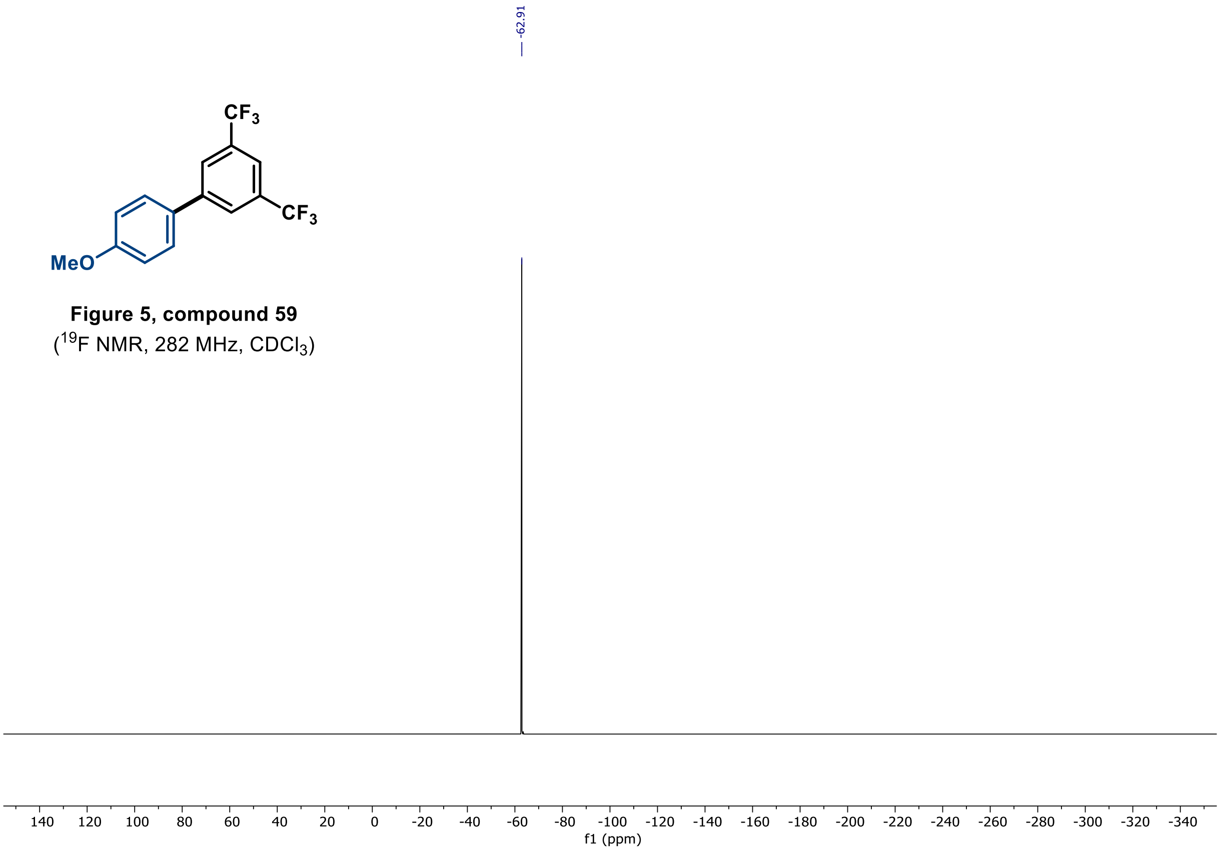

Figure 5, compound 59
(^{19}F NMR, 282 MHz, $CDCl_3$)

2-Phenylquinoxaline (compound 60)

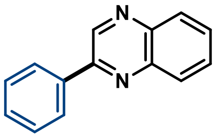

Figure 5, compound 60
(^{1}H NMR, 400 MHz, $CDCl_3$)

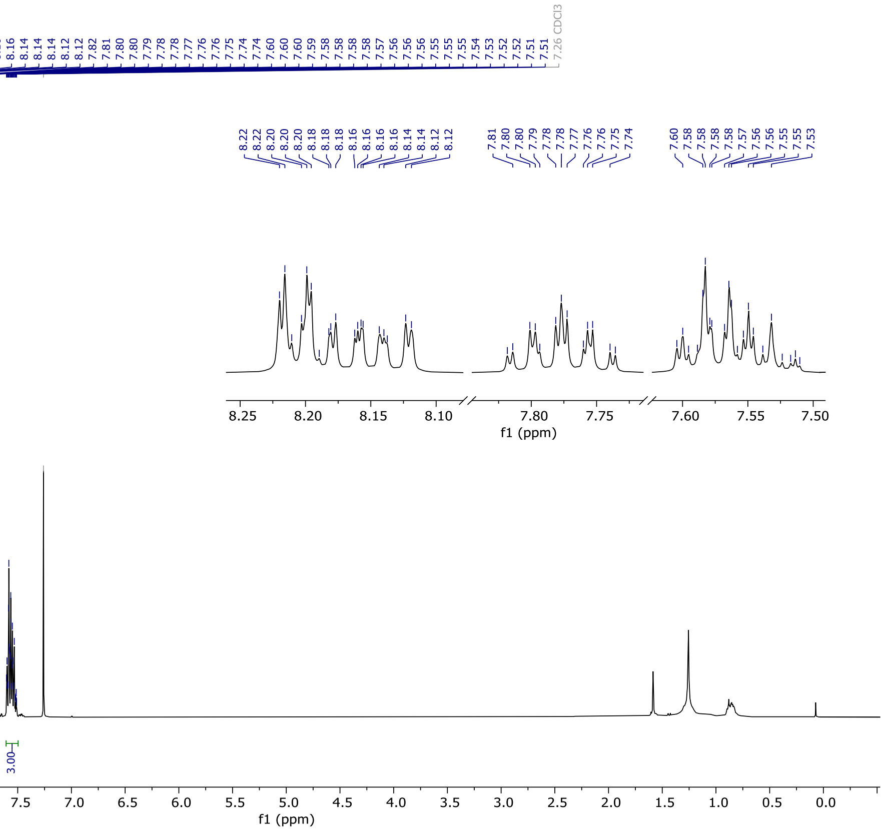

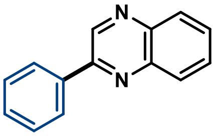

Figure 5, compound 60
(^{13}C NMR, 101 MHz, $CDCl_3$)

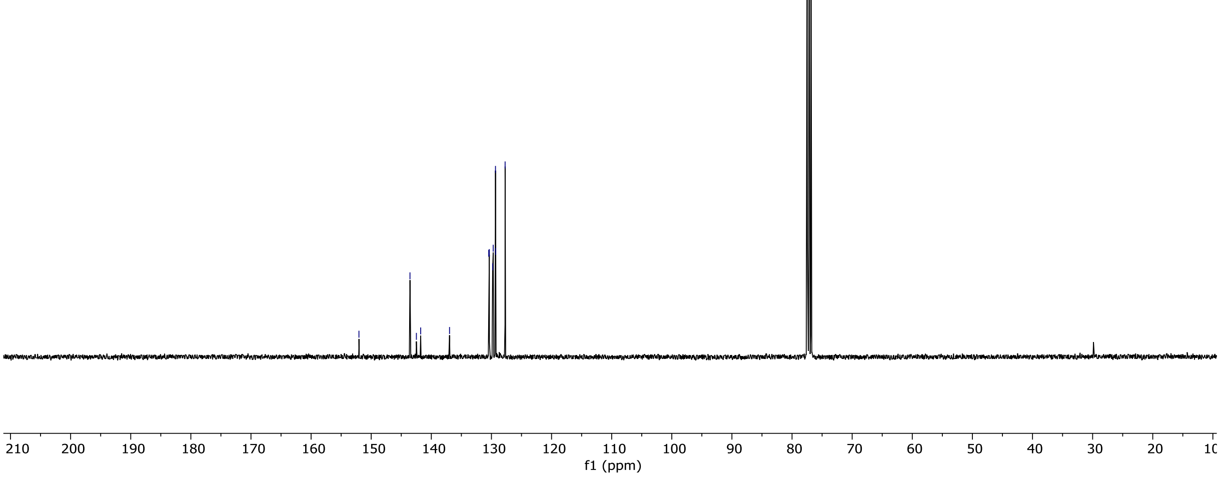

2-Phenylpyrazine (compound 61)

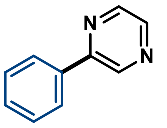

Figure 5, compound 61
(^{1}H NMR, 400 MHz, $CDCl_3$)

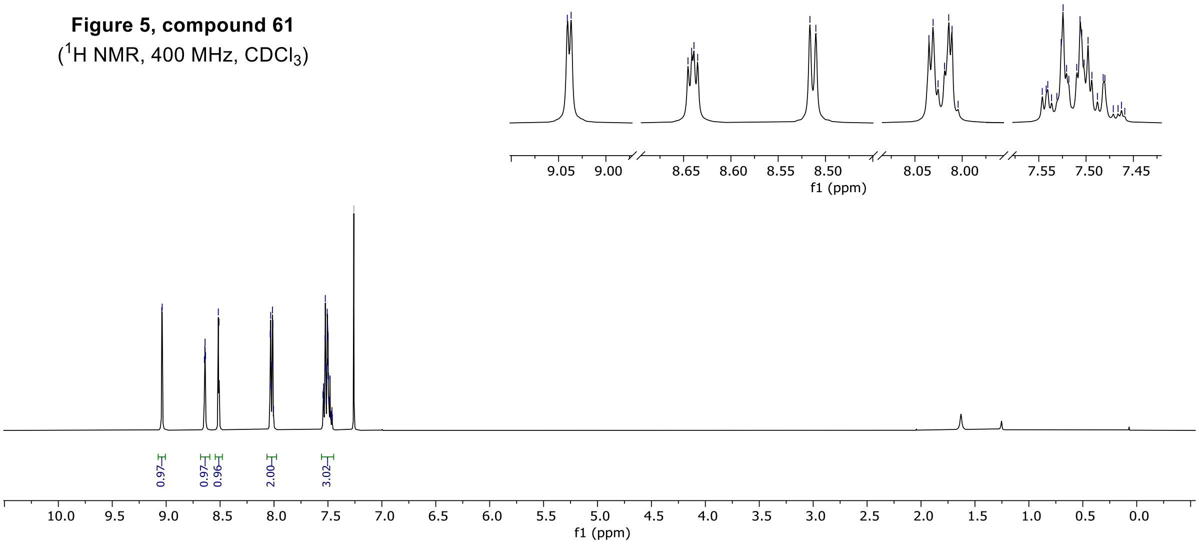

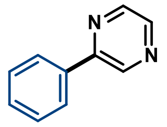

Figure 5, compound 61
(^{13}C NMR, 101 MHz, $CDCl_3$)

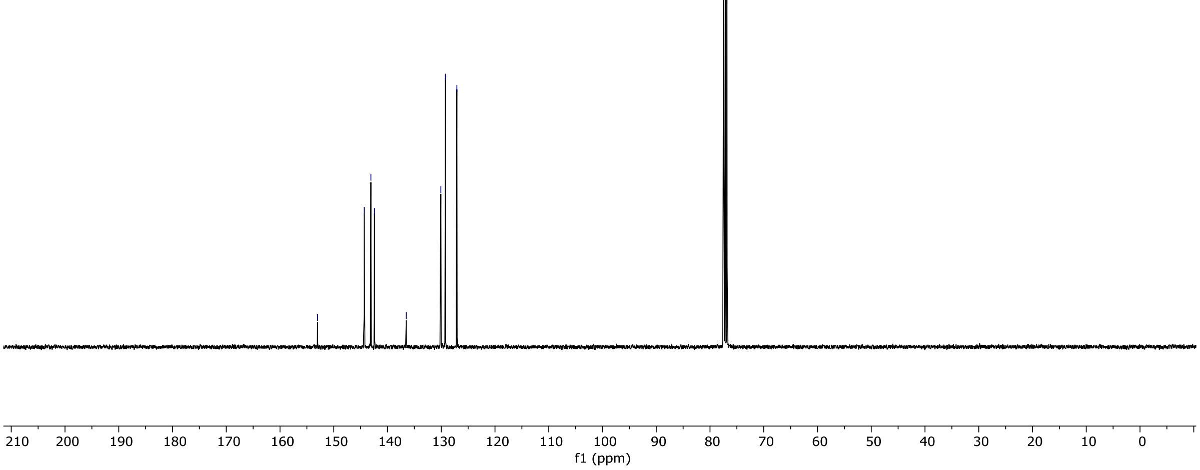

(PPh₃)₂Pd(II)(4-F-Ph)(I) (OAC-1)

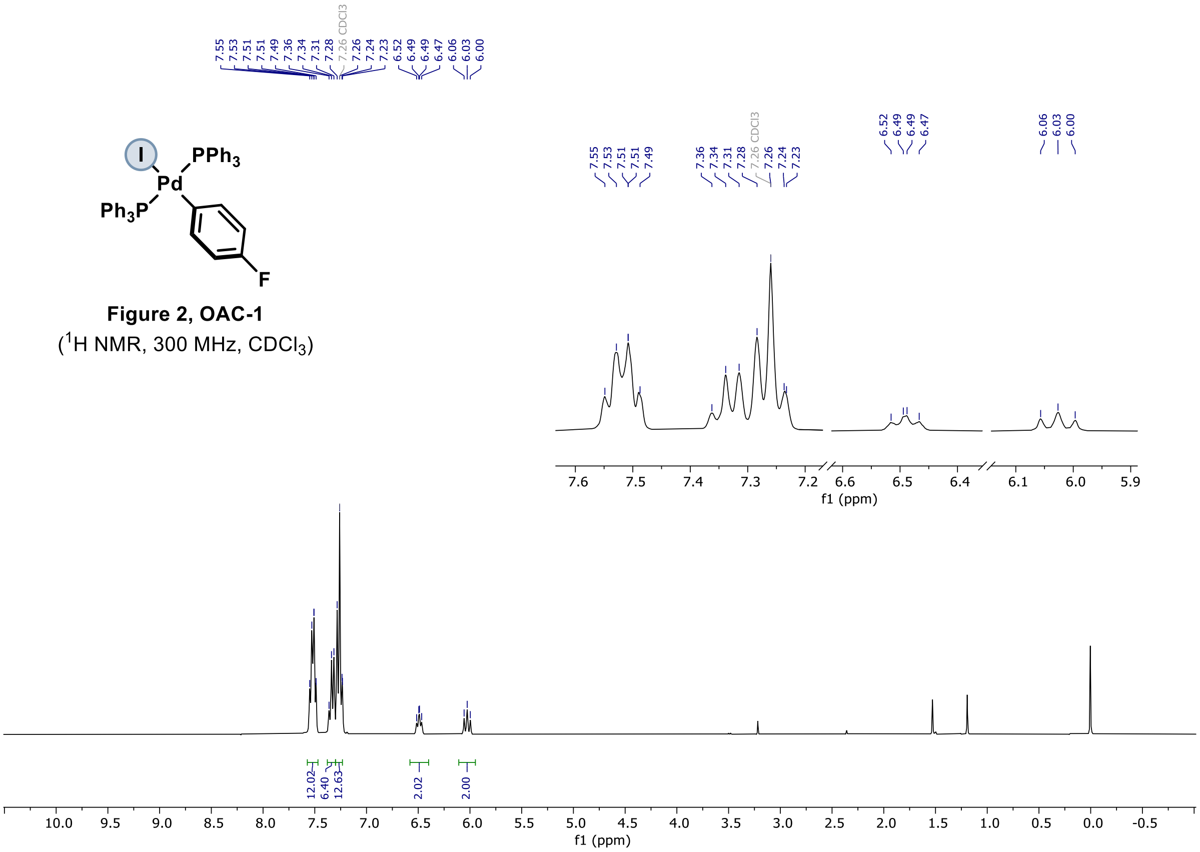

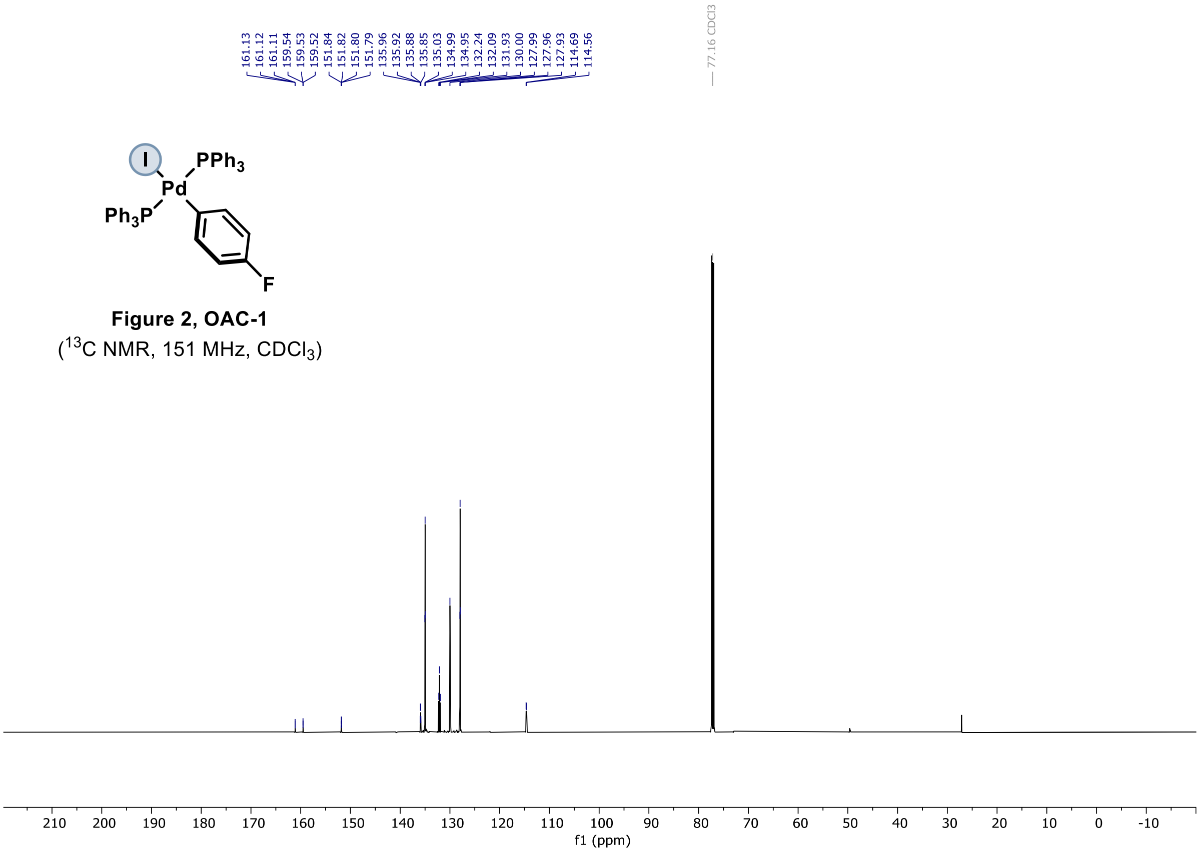

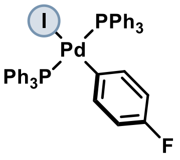

Figure 2, OAC-1
(^{19}F NMR, 282 MHz, $CDCl_3$)

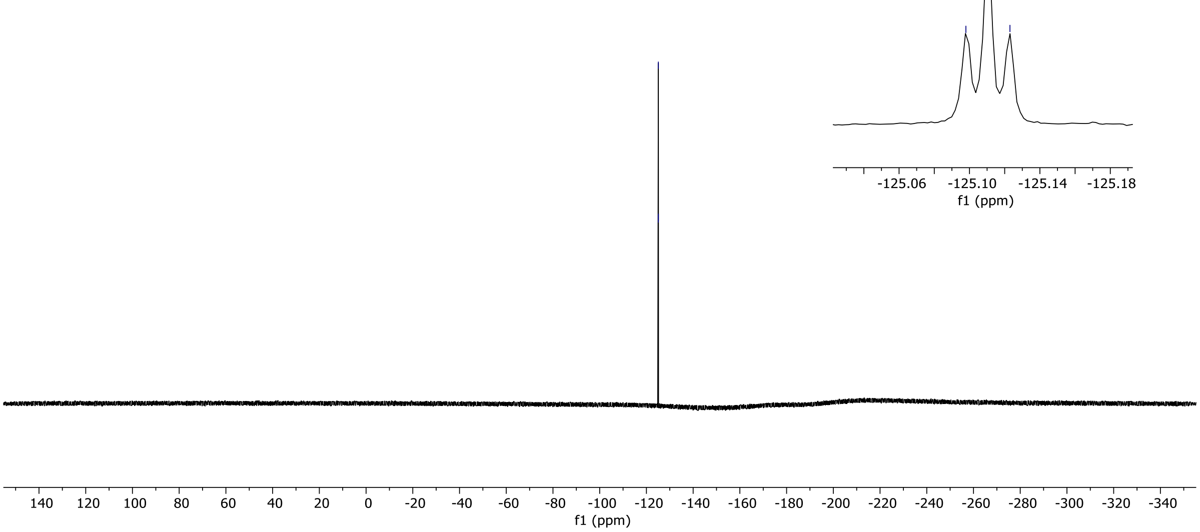

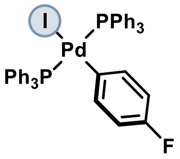

Figure 2, OAC-1
(^{31}P NMR, 122 MHz, $CDCl_3$)

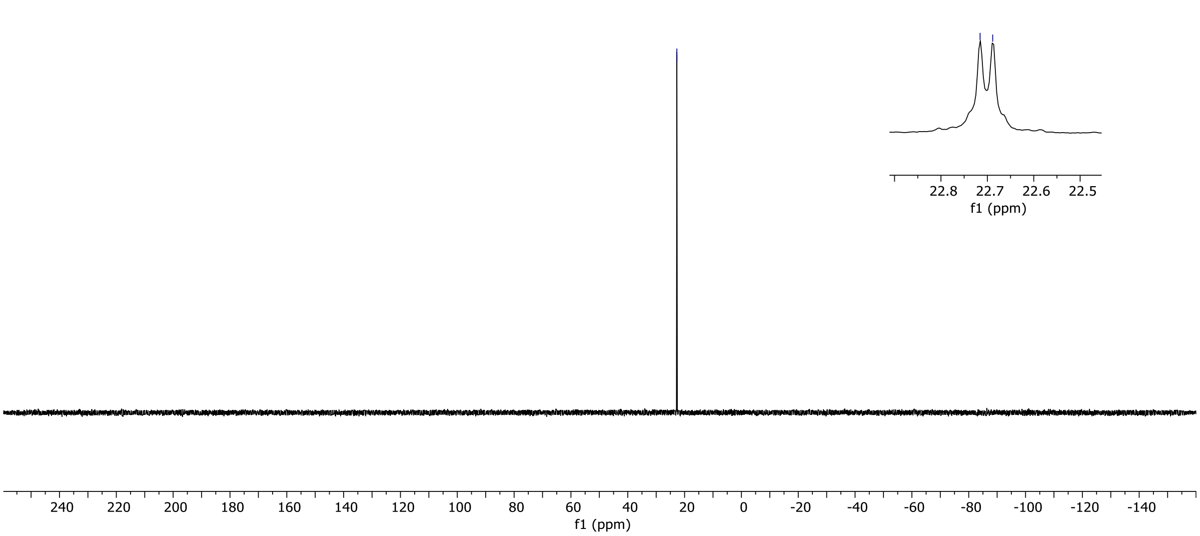

[(2,6-(*t*BuNCH)$_2$C$_6$H$_3$)Bi(4-fluorophenyl)(Cl)], ArBi(Cl)-1

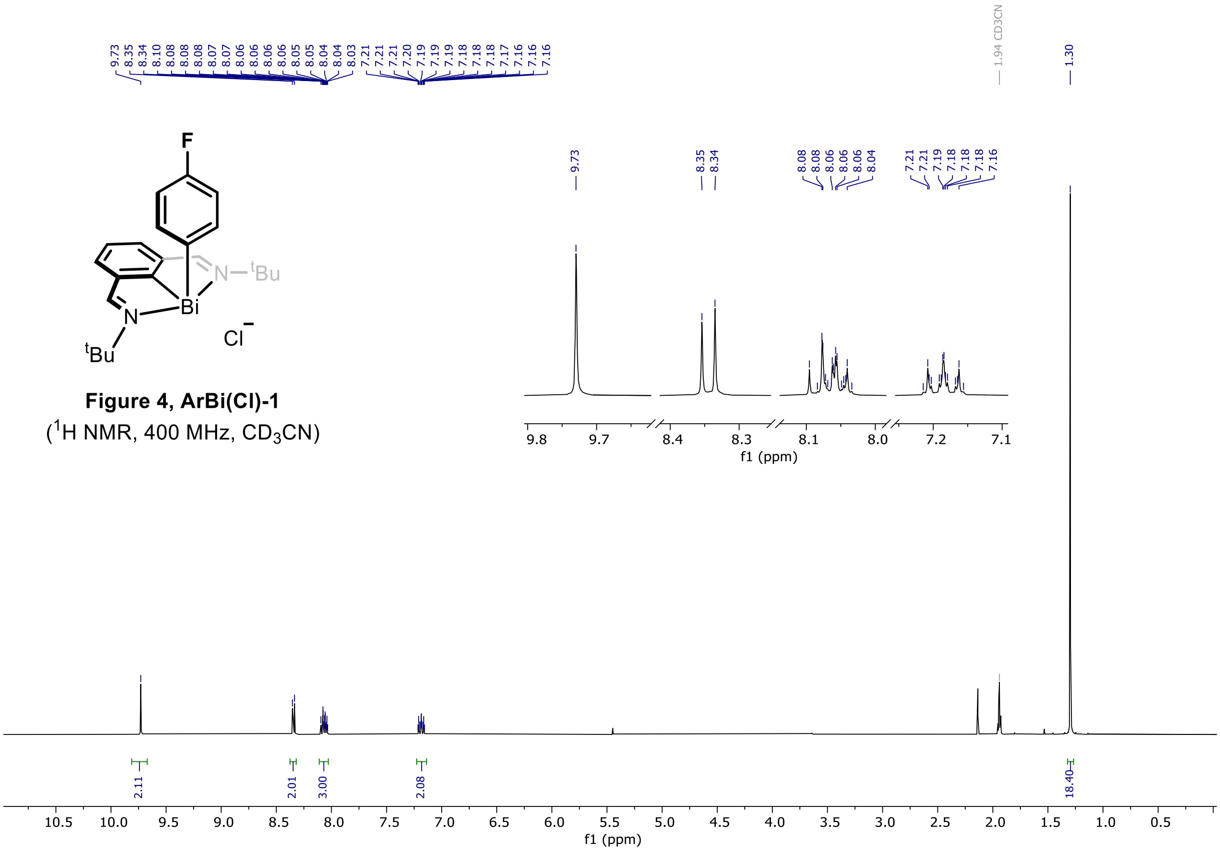

Figure 4, ArBi(Cl)-1
(^{1}H NMR, 400 MHz, CD$_3$CN)

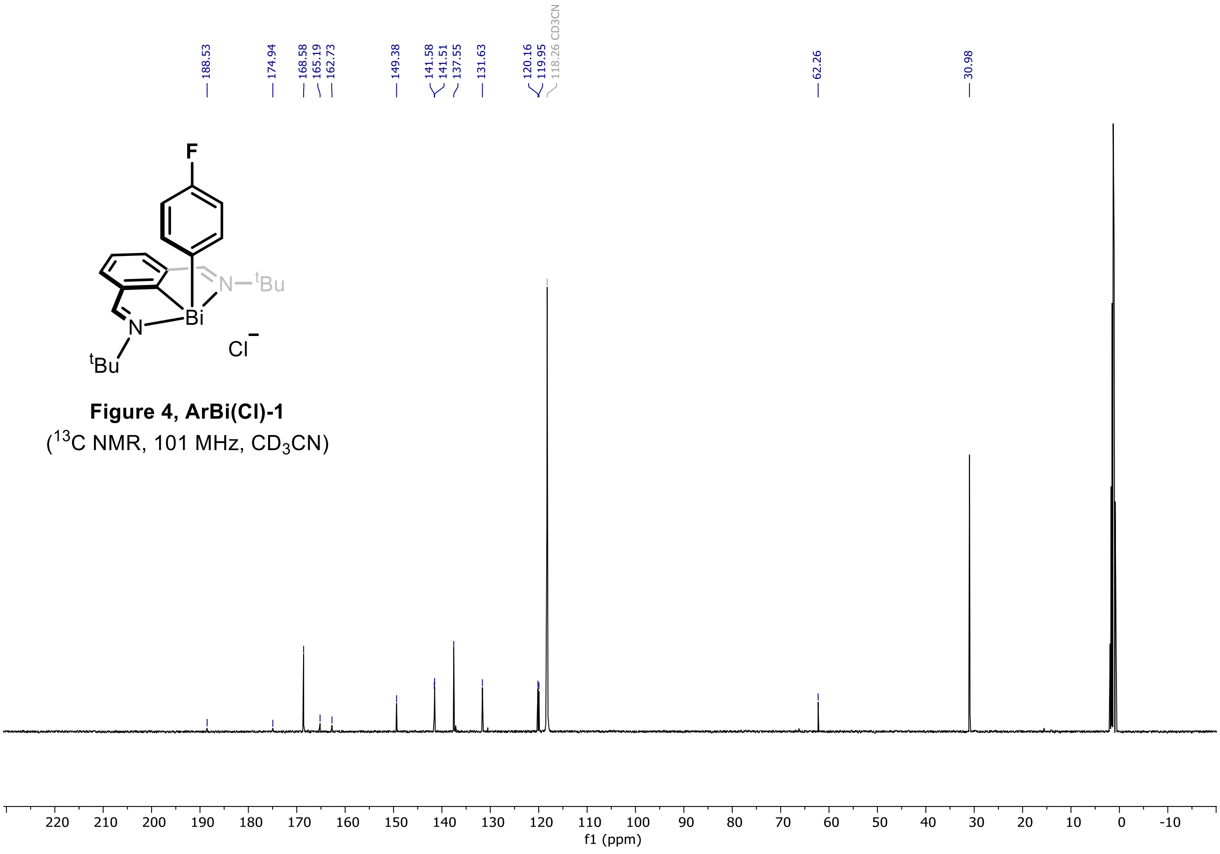

Figure 4, ArBi(Cl)-1
(^{13}C NMR, 101 MHz, CD$_3$CN)

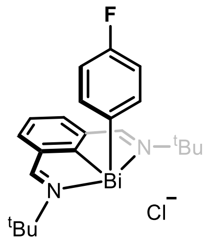

Figure 4, ArBi(Cl)-1
(^{19}F NMR, 282 MHz, $CDCl_3$)

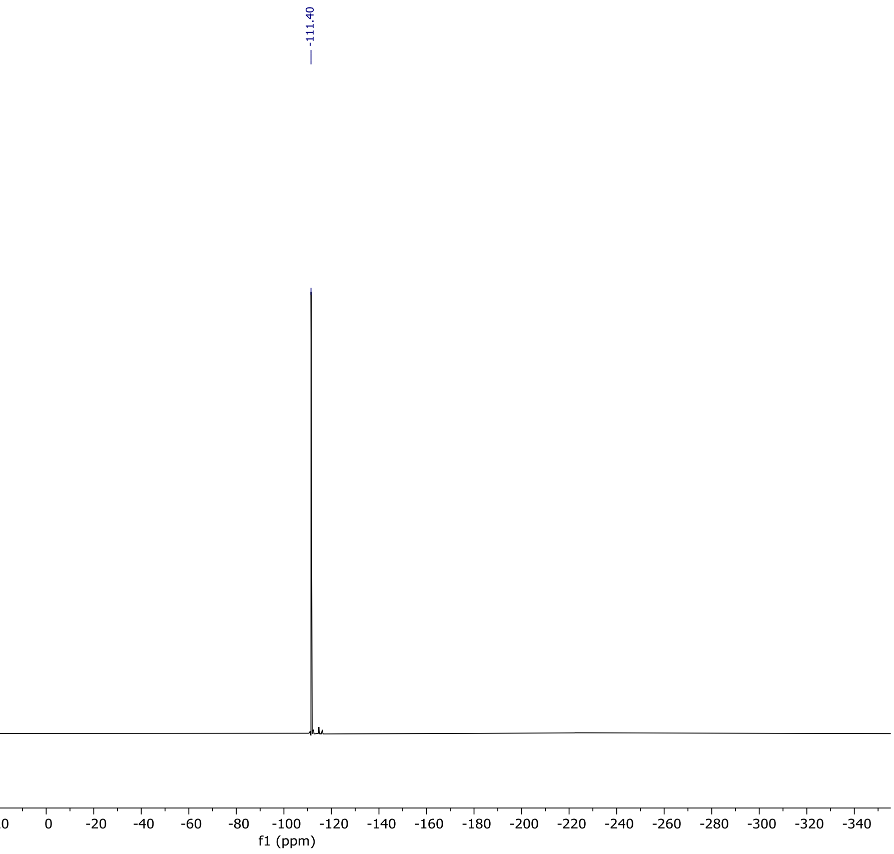

[(2,6-(*t*BuNCH)$_2$C$_6$H$_3$)Bi(4-methoxyphenyl)(Cl)], ArBi(Cl)-2

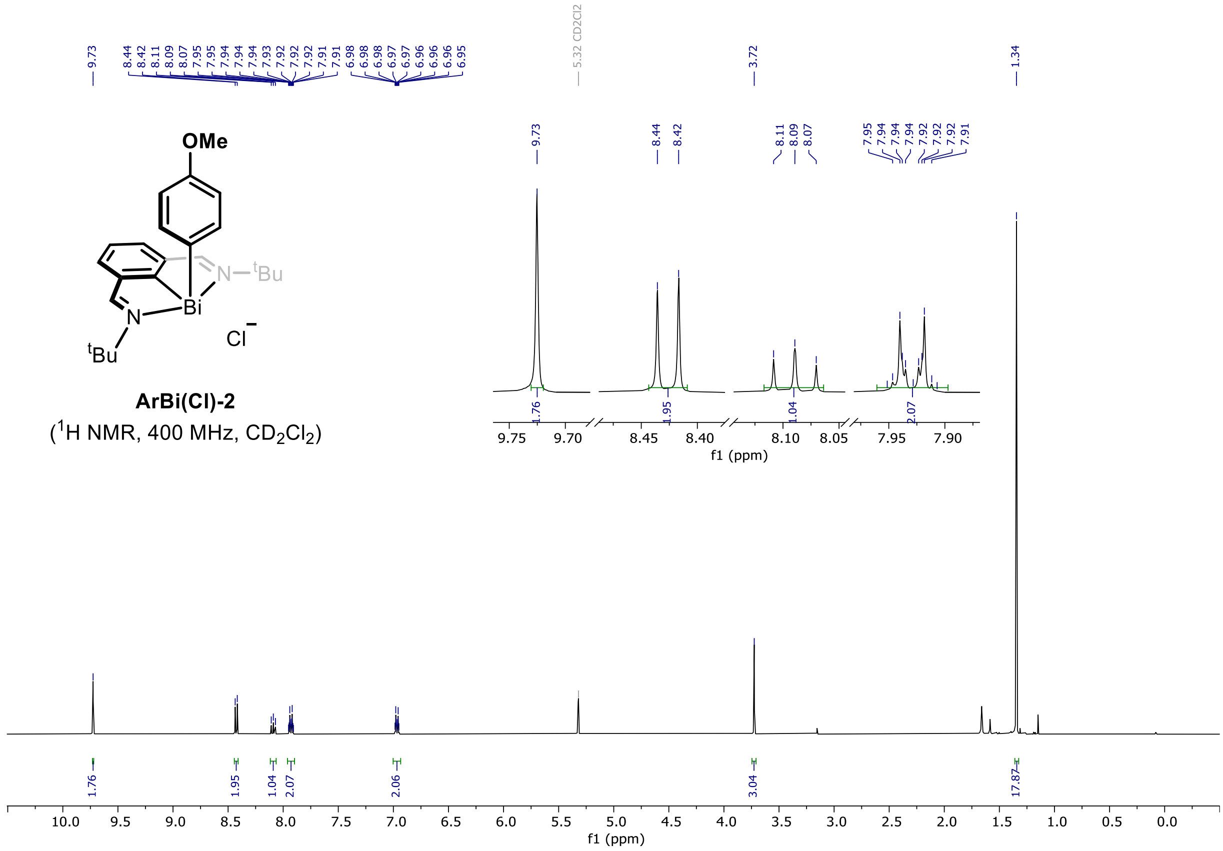

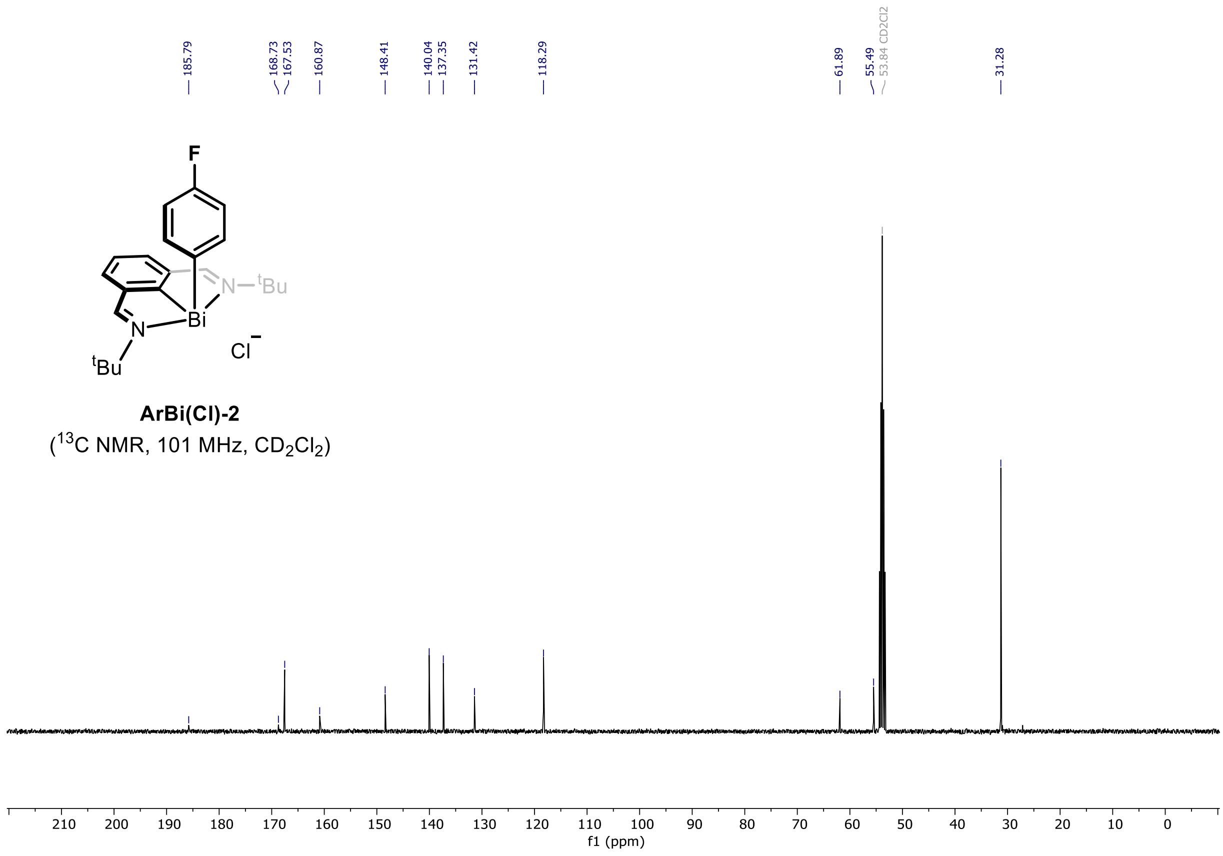

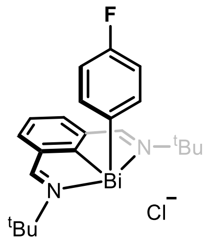

Figure 4, ArBi(Cl)-1
(^{19}F NMR, 282 MHz, $CDCl_3$)

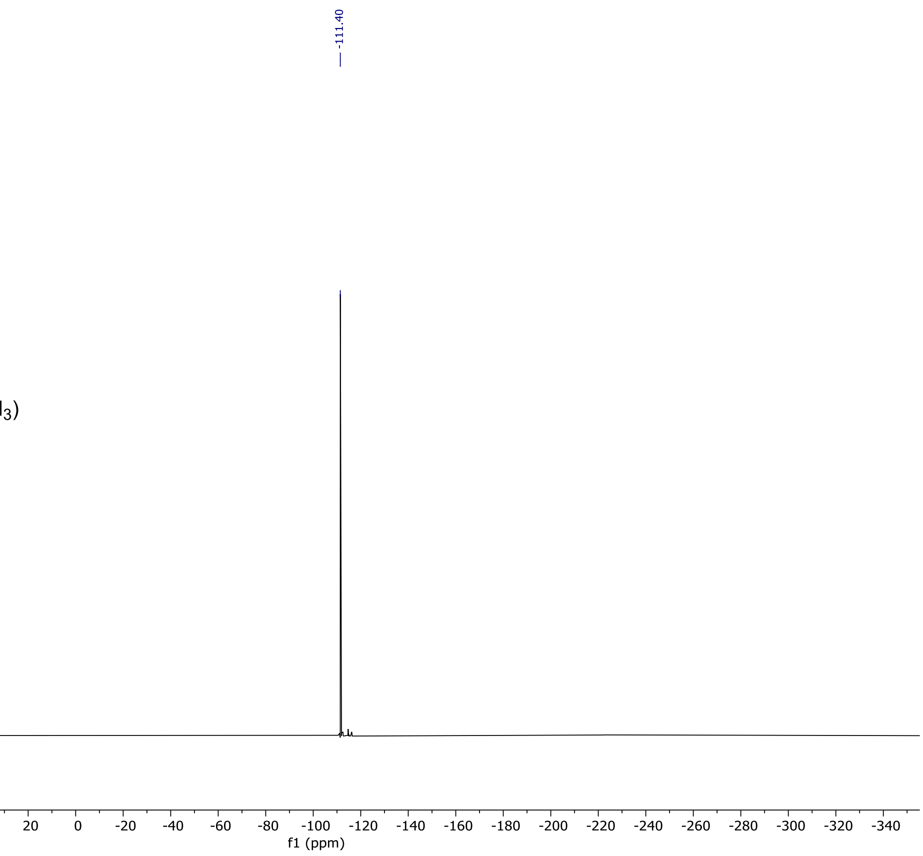

[(2,6-(^{t}BuNCH)$_2$C$_6$H$_3$)Bi(2-methoxyphenyl)(Cl)], ArBi(Cl)-3 (together with 2% of **Bi(III)·Cl$_2$** and 8% **(*n*Bu)Bi(III)·Cl**)

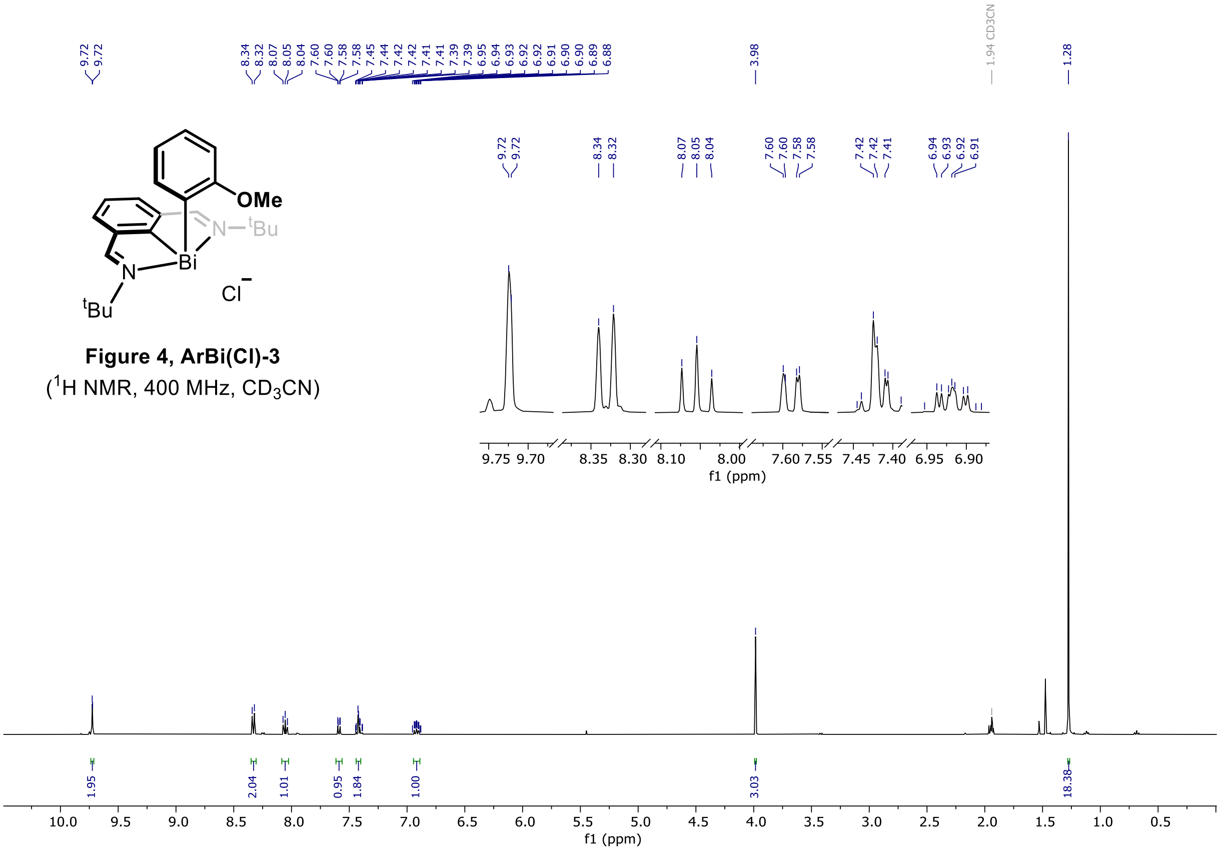

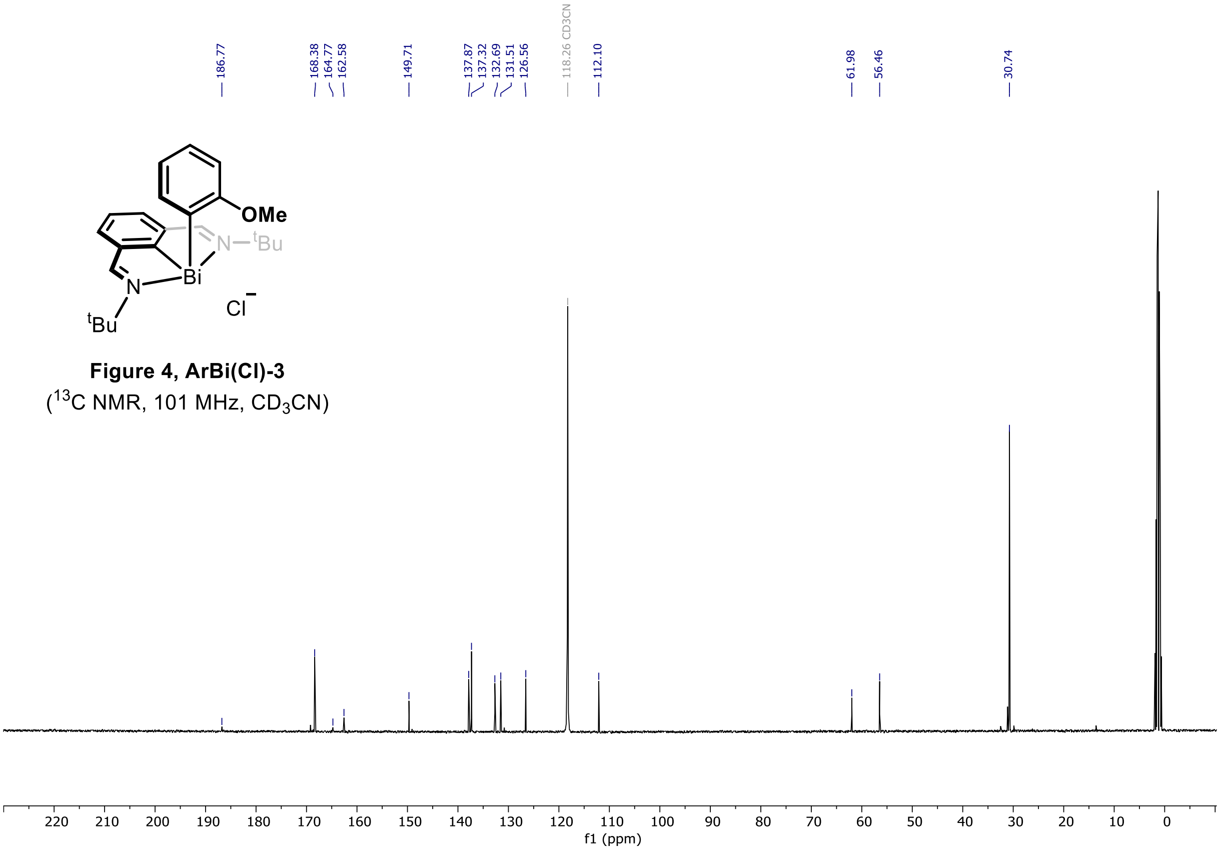

[(2,6-(^{t}BuNCH)$_2$C$_6$H$_3$)Bi(2-methoxyphenyl)(Cl)], ArBi(Cl)-3

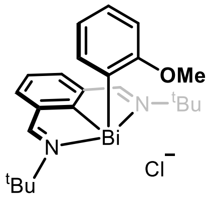

Figure 4, ArBi(Cl)-3
(^{1}H NMR, 400 MHz, CD$_2$Cl$_2$)

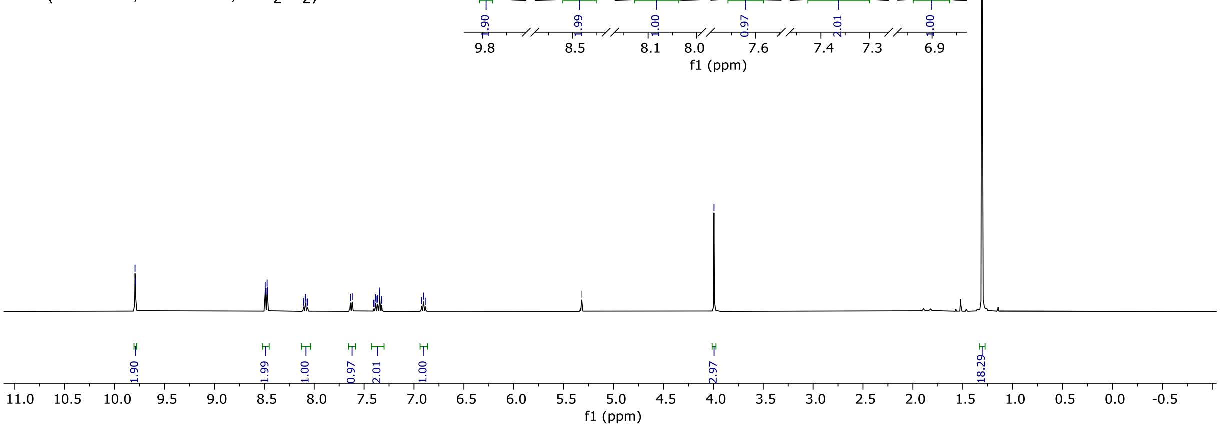

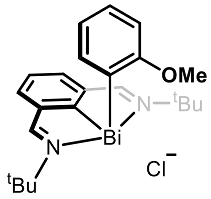

Figure 4, ArBi(Cl)-3
(^{13}C NMR, 101 MHz, CD$_2$Cl$_2$)

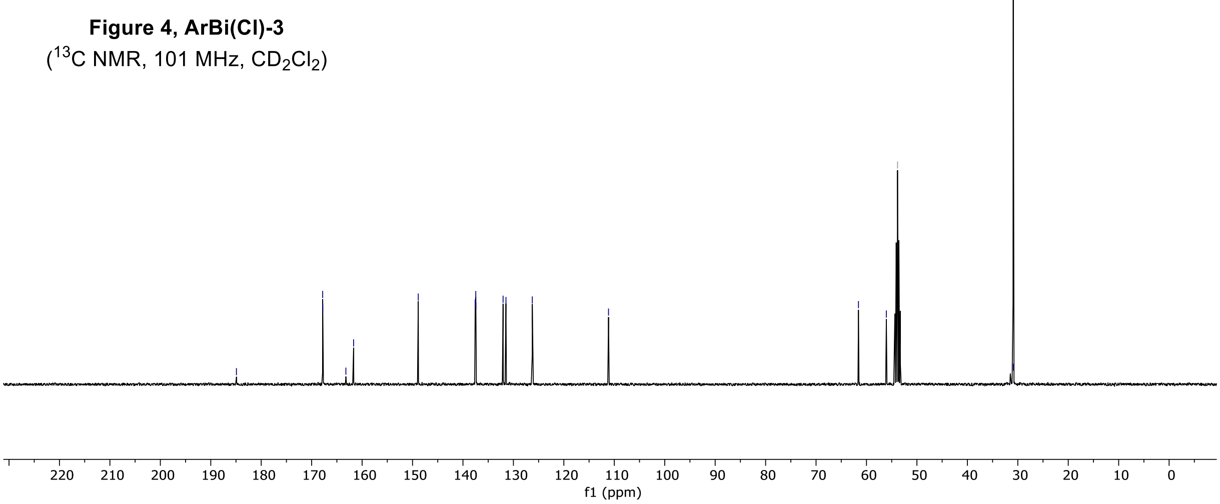

Supplement: Supplementary file 1 — Supplementary Information containing the following sections: 1. General experimental details; 2. Procedures for the preparation of starting materials; 3. Optimization of reaction conditions; 4. General procedures for ambiphilic cross-coupling; 5. Synthesis and characterization of the products; 6. Mechanistic investigations; 7. Convergent synthesis of ambiphilic N,C,N-pincer aryl-bismuth compounds; 8. References; and 9. NMR spectra for isolated products and substrates. [file 41586_2026_10486_MOESM1_ESM.pdf]
